# Stereoselective synthesis, VCD study and conformational analysis of *C*-glycosyl isochromans

**DOI:** 10.1038/s41598-026-46290-7

**Published:** 2026-04-24

**Authors:** Nawar Ahmad, Ágnes Homolya, Roland A. Barta, Mihály Herczeg, Attila Bényei, Nika Iurgenson, Ilona Bereczki, Gergely M. Fedics, Attila Mándi, Tibor Kurtán, Anikó Borbás

**Affiliations:** 1 Department of Pharmaceutical Chemistry, University of Debrecen, Egyetem tér 1, Debrecen, 4032 Hungary; 2 Doctoral School of Pharmaceutical Sciences, University of Debrecen, Egyetem tér 1, Debrecen, 4032 Hungary; 3 Department of Organic Chemistry, University of Debrecen, P.O. Box 400, Debrecen, 4002 Hungary; 4 Doctoral School of Chemistry, University of Debrecen, Egyetem tér 1, Debrecen, 4032 Hungary; 5 Department of Physical Chemistry, University of Debrecen, Egyetem tér 1, Debrecen, 4032 Hungary; 6 Present Address: Department of Chemistry, Eszterházy Károly Catholic University, Leányka utca 12, Eger, 3300 Hungary

**Keywords:** Isochroman, Carbohydrate, C-glycoside, Stereocontrolled synthesis, Vibrational circular dichroism (VCD) spectroscopy, DFT-based conformational analysis, Oxa-Pictet–Spengler cyclization, Biochemistry, Chemical biology, Chemistry, Computational biology and bioinformatics, Drug discovery

## Abstract

Isochromans and aryl-*C*-glycosides are important structural motifs in many natural products and drug candidates with diverse therapeutic potentials. Here we present the first application of pyranosyl aldehydes in the oxa-Pictet–Spengler cyclization to conjugate the two pharmacophore motifs. Enantiomeric pairs of aryl-2-propanols were reacted with pyranosyl dialdoses and 1-formyl sugars in a BF_3_·Et_2_O-mediated oxa-Pictet–Spengler reaction. The effects of the substitution pattern, configuration, and protecting groups of the reactants on the efficiency and stereochemical outcome of the reactions were studied, and the reaction conditions were optimized. A series of 1,3-*cis*-substituted 1-(*C*-glycosyl)isochromans was prepared in high yields with good to complete stereoselectivity from glucosyldialdoses and 1-formyl glucopyranosides. The reaction efficiency dropped significantly with *galacto*-dialdose derivatives due to steric hindrance, leading to lower yields and anomerization, which slightly limits the universality of the method. ROESY-NMR, X-ray diffraction, and VCD methods were used to determine the structure and the absolute configuration of the compounds. The method developed represents a straightforward and stereocontrolled route to a new chemotype of isochroman *C*-glycosides.

**Supplementary Information:**

The online version contains supplementary material available at 10.1038/s41598-026-46290-7.

## Introduction

**Fig. 1:**
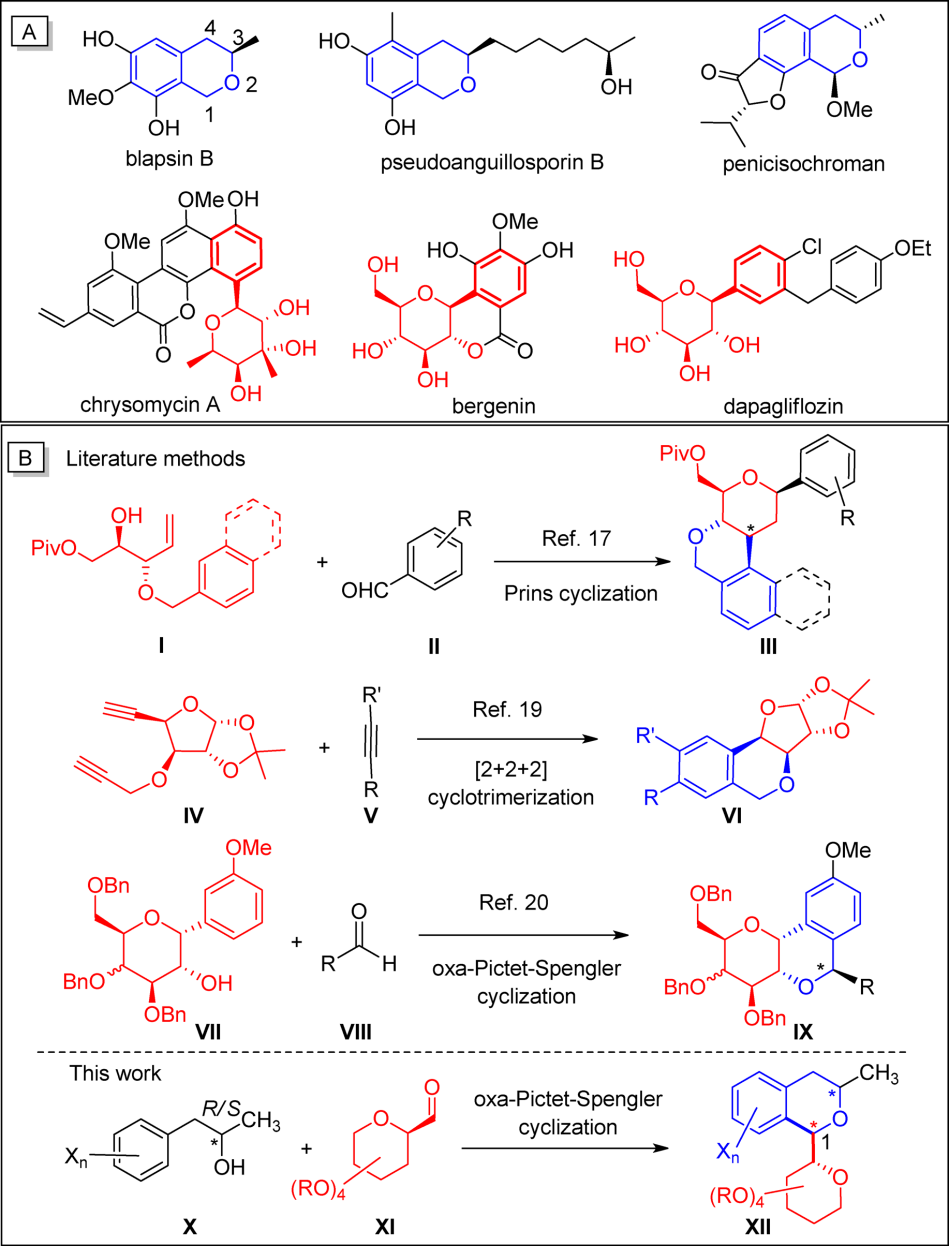
(**A**) Examples for bioactive isochroman natural products and bioactive aryl-*C*-glycosides. The isochroman core is shown in blue, the aryl-*C*-glycosidic motif is shown in red. (**B**) Application of sugar skeletons for the synthesis of isochroman scaffolds.

The benzene-fused *O*-heterocycle isochroman (3,4-dihydro-l*H*-benzo[c]pyran) constitutes the backbone of many bioactive natural products. For example, blapsin B, isolated from the insect *Blaps japanensis*, has been identified as a 14-3-3 protein–protein interaction inhibitor^1^, pseudoanguillosporin B shows antibacterial and antifungal effects^2^, while penicisochroman exerts potent anticancer activity^3^ (Fig. 1A). Furthermore, numerous synthetic isochroman-based compounds have been published as promising drug candidates with various therapeutic activities^4^.

Another important class of biorelevant organic compounds is the aryl-*C*-glycosides, in which the anomeric carbon atom of the carbohydrate moiety is linked to an aromatic aglycone unit through a carbon–carbon bond^5^. Unlike the more common *O*-glycosides in nature, which are sensitive to enzymatic hydrolysis, *C*-glycosides have enhanced chemical and metabolic stability, which gives them unique advantages as potential therapeutic agents. Aryl-*C*-glycosides are important motifs in many natural compounds that exhibit diverse biological activities, e.g.antibacterial (chrysomycin)^6^, and anti-inflammatory effects (bergenin)^7^, while the synthetic aryl-*C*-glycoside derivatives gliflozins (e.g. dapagliflozin) are used in the treatment of type 2 diabetes^8^ (Fig. 1A).

Due to the mentioned broad biological activities, the synthesis of both *C*-glycosides^9–11^ and isochroman scaffolds^12–14^ has received considerable attention, leading to the development of several efficient methods, which, however, still do not provide sufficient structural diversity for drug discovery. Therefore, there is a continuous need to develop new approaches, e.g., the application of known methods in innovative synthetic strategies to generate new structures of these valuable scaffolds^15,16^.

The most straightforward and efficient method for the preparation of isochroman core is the oxa-Pictet–Spengler reaction, which is an acid-catalyzed ring-closure reaction between a 2-arylethanol derivative and a carbonyl compound^14^. We envisioned that by innovatively employing an aldehyde derivative of a carbohydrate as the oxo component in the oxa-Pictet–Spengler cyclization, we could generate novel *C*-1 glycosyl isochromans, in which the isochroman motif is combined with a homo-aryl-*C*-glycoside motif (Fig. 1B).

There are only sporadic examples of the use of carbohydrate skeletons in the synthesis of isochromans, which always lead to sugar-fused isochromans by Prins reaction (**III**)^17^ or 2 + 2 + 2 tricyclization (**VI**)^18,19^. To the best of our knowledge, only one method has been reported to prepare carbohydrate isochromans based on oxa-Pictet–Spengler cyclization^20^. In this approach, Vankar and co-workers used the carbohydrate scaffold as a 2-arylethanol reactant (**VII**), which was reacted with various aldehydes to give sugar-fused 1,2-annulated isochromans (**IX**)^20^. The advantages of this method lie in its efficiency and high stereoselectivity. However, it requires the synthesis of starting aryl C-glycosides (**VII**), which is often challenging, and its applicability is limited to the preparation of bergenin-type sugar-fused isochroman skeletons (Fig. 1B).

In our new approach to the synthesis of sugar isochromans, in contrast to the Vankar method, carbohydrates are used as aldehyde components in the oxa-Pictetet–Spengler reaction, not as alcohol components. Cyclization of sugar aldehydes (**XI**) with arylethanols (**X**) leads to a new chemotype of glycosyl isochromans, in which the sugar moiety is uniquely attached to the C1 position of the isochroman backbone (**XII**) and is not fused to the isochroman ring system. Here we describe the oxa-Pictet–Spengler reaction of variously substituted optically active 1-methyl-2-arylethanols and various sugar aldehydes, including pyranosyl dialdoses and 1-formyl pyranosides. We studied the stereochemical outcome of the reactions and determined the absolute configuration of the compounds by NMR, VCD, and X-ray diffraction methods.

## Results and discussion

### Synthesis

In the initial experiments, we reacted (*S*) and (*R*) isomers of 1-(3,4-dimethoxyphenyl)propan-2-ol (**1**) and trimethylsilyl ether-protected 6-aldehydes, α-Me-glucoside and galactoside derivatives (**2** and **6**). By using alcohols (*S*)-**1**^21^ and (*R*)-**1**^22^, both stereoisomeric forms of 3-methylisochromans, characteristic of bioactive derivatives, can be formed. The silylated dialdoses were chosen because they can be efficiently prepared from commercially available α-methyl glycosides using a two-step literature method^23^. Moreover, under the conditions of the oxa-Pictet–Spengler reaction, the acid-sensitive silyl groups are presumably cleaved, thus generating the free glycosyl isochroman final product in a single step. The optimization reactions performed with the Lewis acid BF_3_^.^Et_2_O, most commonly used in the oxa-Pictet–Spengler cyclization, are shown in Table 1.

**Table 1:** Optimization of the oxa-Pictet–Spengler cyclization of (*S*)-**1** and (*R*)-**1** with dialdose **2**.

Entry	Alcohol	Acid equiv	Acid equiv	Temp. (°C)	Time (h)	Product (Yield %)^a^
1	(*S*)	BF_3_^.^Et_2_O	0.2 equiv	0 °C to rt	24 h	**3** (32%)
2	(*S*)	BF_3_^.^Et_2_O	0.2 equiv	0 °C to rt	48 h	**3** (33%)
3	(*S*)	BF_3_^.^Et_2_O	2 equiv	0 °C to rt	5 h	**3** (53%)
4	(*S*)	BF_3_^.^Et_2_O	3 equiv	0 °C to rt	12 h	**3** (57%)
5	(*S*)	CSA	3 equiv	50 °C	72 h	**3** (8%)
6	(*R*)	BF_3_^.^Et_2_O	0.2 equiv	0 °C to rt	7 h	**4** (23%) **5** (0%)
7	(*R*)	BF_3_^.^Et_2_O	2 equiv	0 °C to rt	5 h	**4** (24%) **5** (22%)
8	(*R*)	BF_3_^.^Et_2_O	3 equiv	0 °C to rt	24 h	**4** (21%) **5** (35%)

Alcohol (**1**) and aldehyde (**2**) were used in a 1:1 ratio.

^a^Isolated yield.

**Fig. 2:**
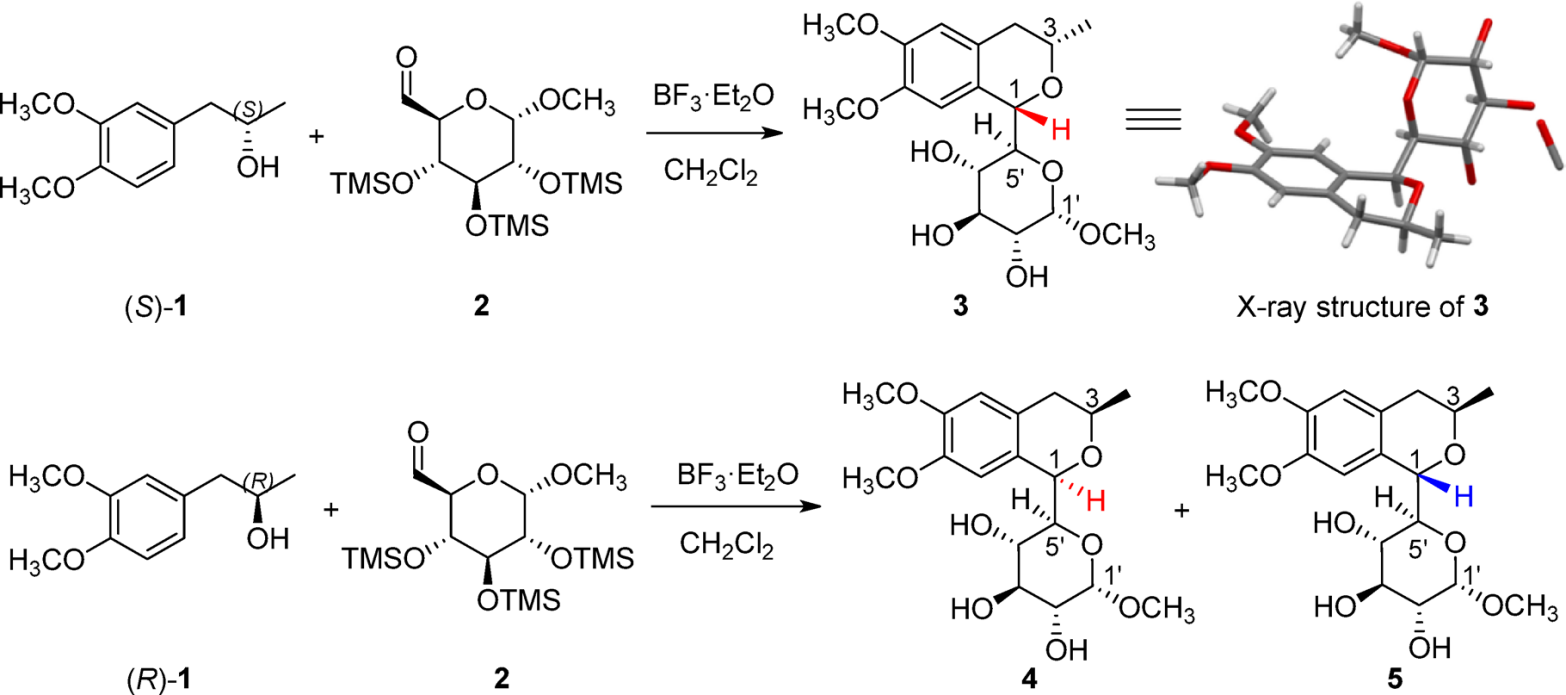
Oxa-Pictet–Spengler cyclization of arylpropan-2-ols (*S*)-**1** and (*R*)-**1** with *gluco*dialdose **2** and single crystal X-ray structure of **3**.

The reaction of alcohol (*S*)-**1** and *gluco*-dialdose **2**^23^ with catalytic amounts of BF_3_^.^Et_2_O gave the 1,3-*cis*-isochroman product **3** with moderate conversion after 24 h (Fig. 2, entry 1, Table 1), and prolonging the reaction hardly increased the yield (entry 2). The conversion was significantly improved by using excess acid, and thus, in the presence of three equivalents of BF_3_^.^Et_2_O, the yield of **3** reached 57% (entry 4). TLC monitoring of these reactions showed rapid cleavage of the silyl ether groups of the sugar in agreement with our initial hypothesis, followed by sluggish ring closure, as indicated by the slow consumption of the alcohol reactant.

In the oxa-Pictet–Spengler (oPS) reaction, the acid catalyst coordinates with the aldehyde, and the hydroxyl group of the alcohol reactant attacks the activated aldehyde, forming a hemiacetal-type intermediate. This intermediate rapidly rearranges to an oxocarbenium ion, whose carbon atom is attached to the aromatic ring in an aromatic electrophilic substitution reaction, forming the isochroman ring system^14,20^. However, the Lewis acid used, BF_3_^.^Et_2_O, preferentially coordinates to hydroxyl groups, and since the rapid cleavage of the acid-sensitive silyl groups from dialdose **2** resulted in three liberated hydroxyls on the sugar moiety, the catalytic amount of Lewis acid coordinating to these OH groups is unable to promote the initial steps of the oPS reaction, leading to a slow reaction (entries 1 and 2). Thus, the coordination of the Lewis acid to the free hydroxyls of the sugar necessitated the use of a large excess of Lewis acid in the reaction of **1** and **2**.

The oPS cyclisation was also carried out through camphorsulfonic acid (CSA) activation (entry 5), which proved to be inferior to the Lewis acid-mediated reaction. This finding is consistent with the results of the Vankar group, which showed that BF_3_^.^Et_2_O was a superior catalyst to protic acids (TfOH, HCl, H_2_SO_4_) in the oxa-Pictet–Spengler ring closure step of Bergenin-type isochromans^20^.

Regardless of the amount and type of acid, the ring closure proceeded with complete stereoselectivity, yielding exclusively the 1,3-*cis*-substituted product, the poor to moderate yields of **3** being due to insufficient conversion. The *cis* orientation of the C-1 glycosyl and C-3 methyl substituents of **3** was determined using ROESY NMR by the crosspeak detected between H-1 and H-3 protons, and it was also confirmed by single crystal X-ray diffraction analysis^24^.

The reaction of enantiomeric alcohol, (*R*)-**1** with dialdose **2** using catalytic BF_3_^.^Et_2_O provided the 1,3-*cis*-substituted isochroman product **4** in a stereoselective manner with low conversion (entry 6, Table 1), analogously to the previous reactions. However, the use of excess acid not only improved the conversion but also resulted in the formation of the C-1 epimeric product **5** (entries 7 and 8). In the presence of two equivalents of BF_3_^.^Et_2_O, in a 5-h reaction, the 1,3-*cis*- and 1,3-*trans*-substituted products were formed in a 1:1 ratio, while when using three equivalents of BF_3_^.^Et_2_O after 24 h, the amount of 1,3-*trans* product slightly exceeded that of 1,3-*cis* product. These results suggest that the 1,3-*cis*-substituted stereoisomer is the kinetic product, but the formation of the diastereomeric mixtures at higher acid concentrations indicates that an equilibrium exists between *cis*- and *trans*-products, and the two distereoisomers may have similar thermodynamic stability.

**Fig. 3:**
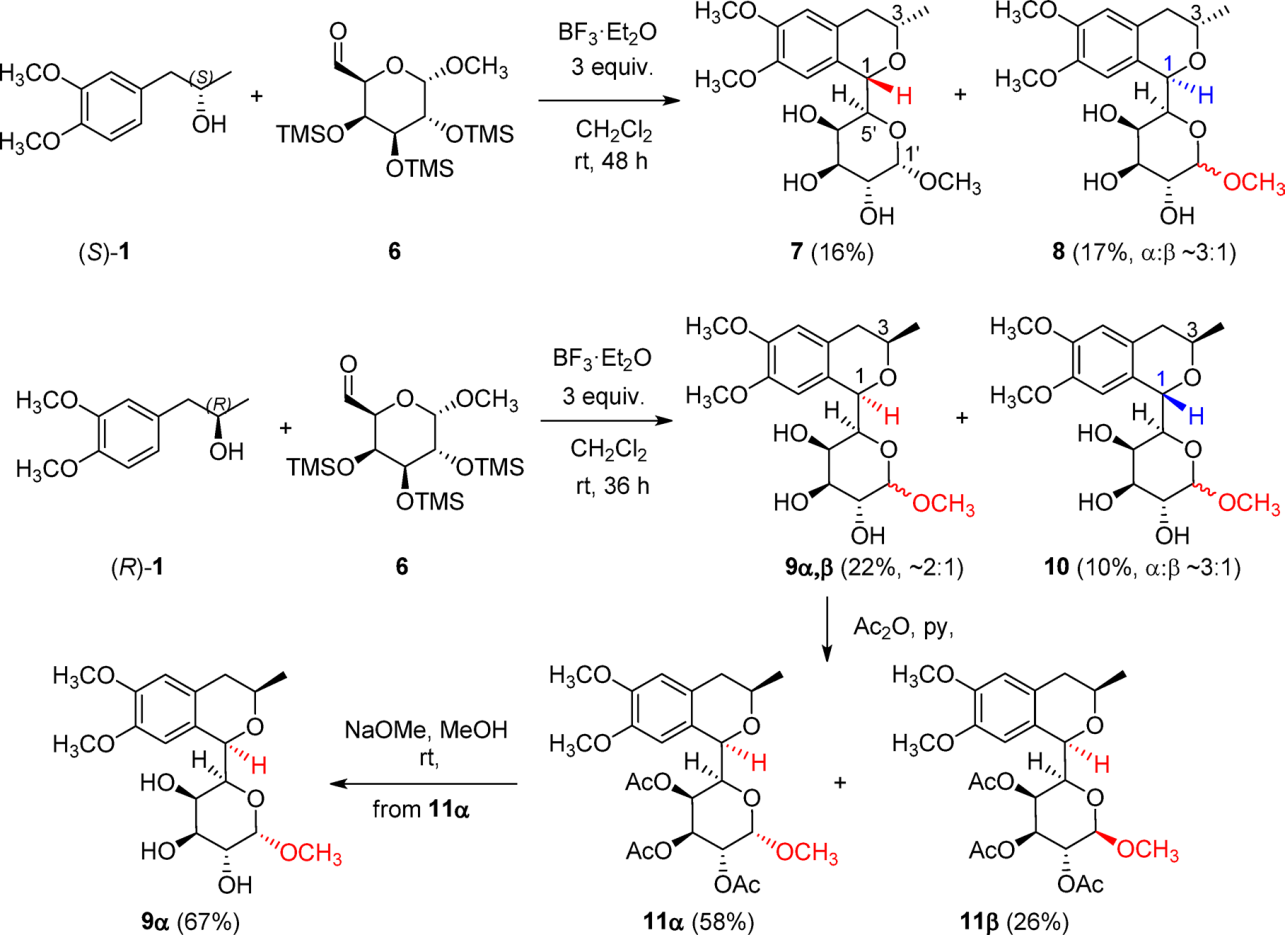
Oxa-Pictet–Spengler cyclization of arylpropan-2-ol (*S*)-**1** and (*R*)-**1** with *galacto*-dialdose **6.**

In order to study the dialdose scope of the cyclization reaction, the *galacto*-dialdopyranose **6** was reacted with the aryl-2-propanols (*S*)-**1** and (*R*)-**1** (Fig. 3). The galactose derivative **6** showed much lower reactivity in the reactions than its glucose counterpart, therefore, we used a large excess of acid and a long reaction time to increase the conversion, thus achieving moderate yields. In the reaction with alcohol (*S*)-**1**, two products were isolated in a nearly 1:1 ratio. Compound **7** was the expected 1,3-*cis*-substituted isochroman, the structure of which was confirmed by X-ray crystallography (see Supporting Information, Figure S2)^24^. The chromatographically uniform product **8** contained the 1,3-*trans* stereoisomer as a ~ 3:1 α:β anomeric mixture according to NMR analysis.

The ring-closure reaction of **6** with alcohol (*R*)-**1** also produced the 1,3-*cis*- (**9**) and the 1,3-*trans*-substituted (**10**) isochroman derivatives, both in the form of an inseparable α,β anomeric mixture, in a ratio of 2:1 and 3:1 in favor of the α-anomer, respectively. After acetylation of the major 1,3-*cis* product **9α,β**, the **11α **and **11β** anomers were separated, and pure **9α** was obtained by deacetylation of **11α**. These results indicate that the *galacto* configuration of the dialdose is not favorable for the oxa-Pictet–Spengler reaction, leading to low yields and undesirable anomerization. It is hypothesized that prolonged exposure of compound **6** to Lewis acid resulted in anomerization via cleavage of the pyranosyl C-1 ring-oxygen bond^25,26^. The low efficiency of cyclization could be attributed to steric hindrance of the pyranosyl C-4 hydroxyl group, which is in *cis*-arrangement with the adjacent aldehyde group.

**Fig. 4:**
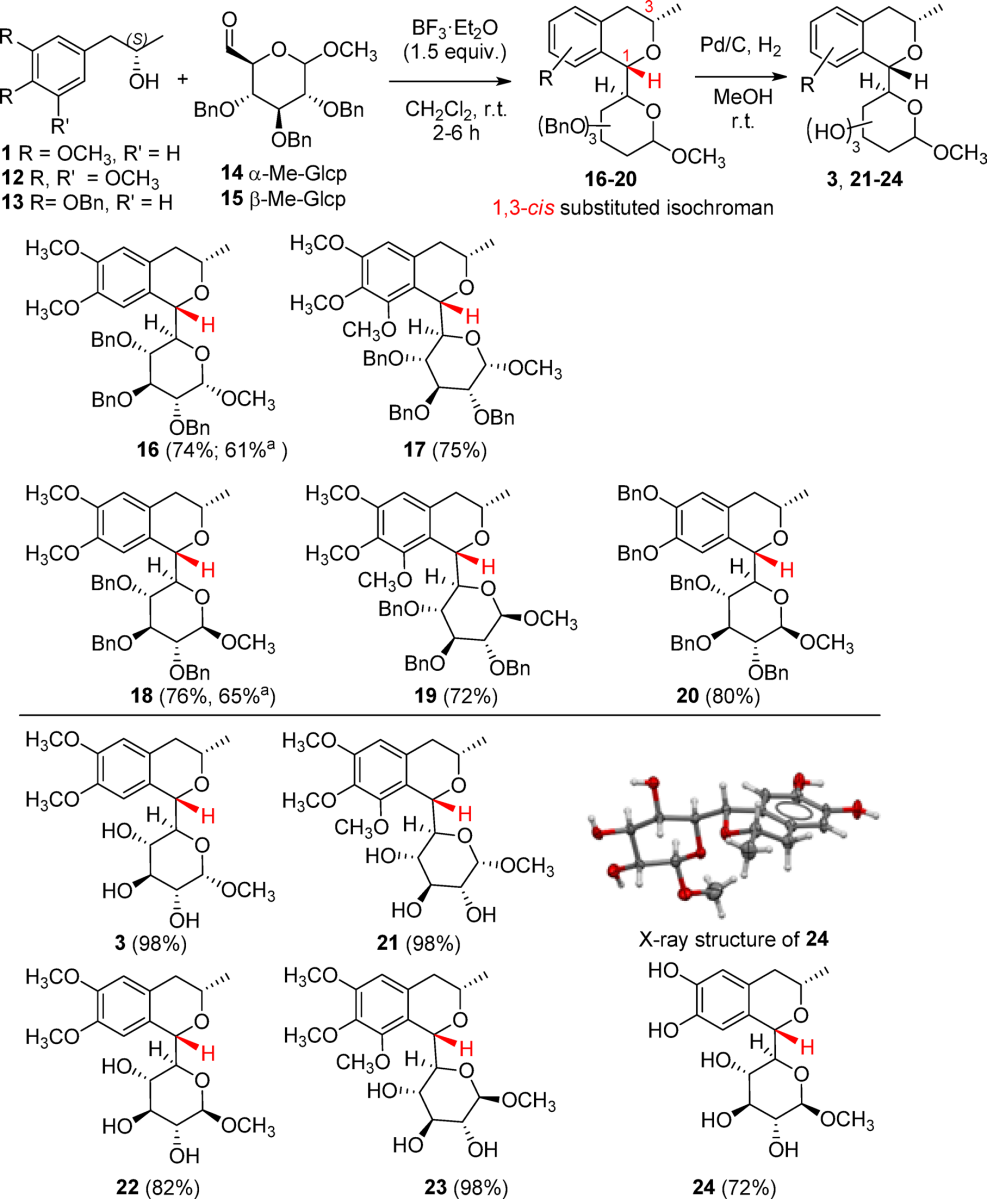
Oxa-Pictet–Spengler cyclization of (*S*)-aryl-2-propanols (*S*)-**1**, (*S*)-**12,** and (*S*)-**13** with benzyl-protected α- and β-methyl *gluco*dialdose derivatives **14** and **15**, and deprotection procedure. ^a^The reaction was performed with 0.5 equiv. of BF_3_^.^Et_2_O.

**Fig. 5:**
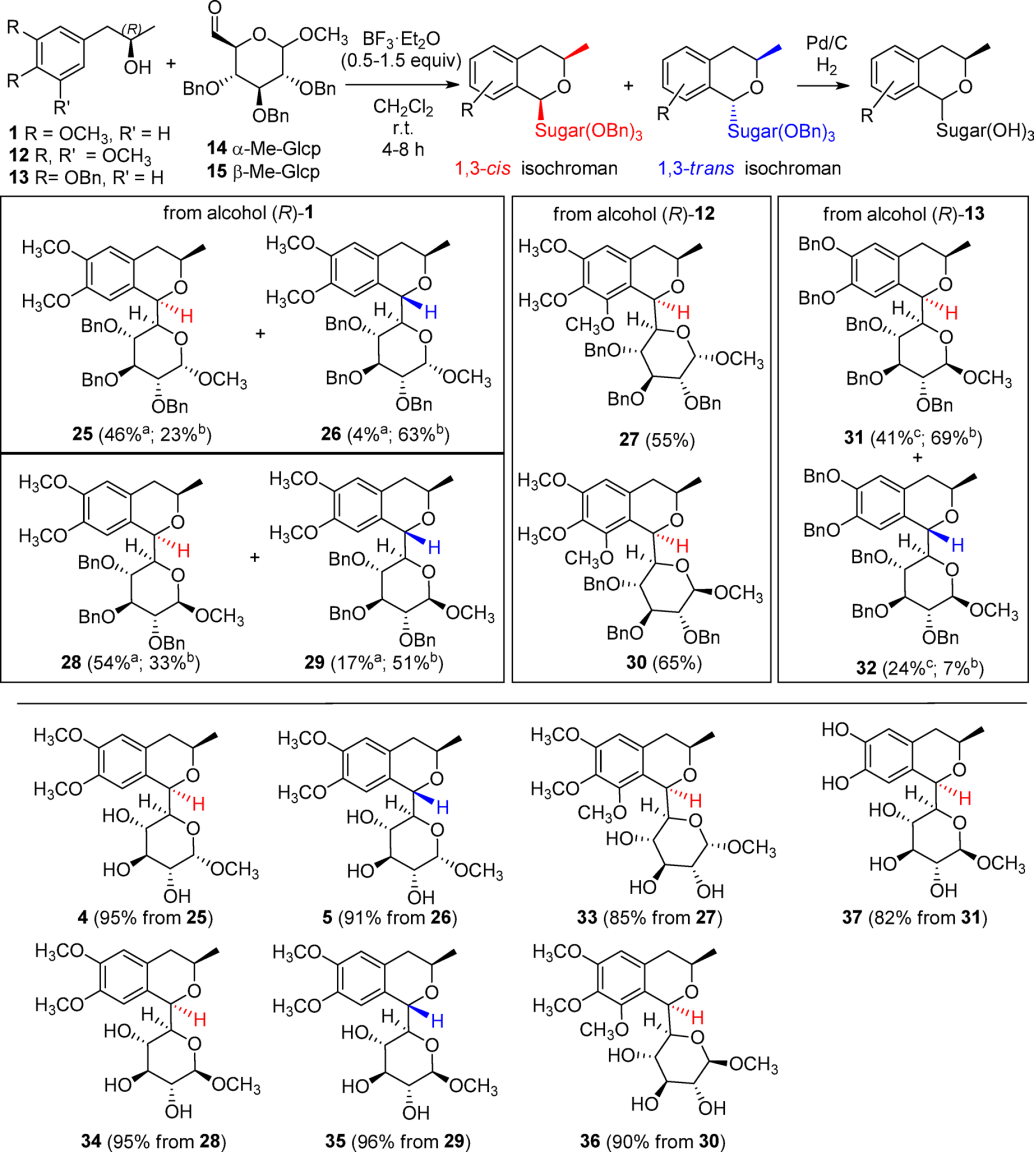
Oxa-Pictet–Spengler cyclization of (*R*)-aryl-2-propanols (*R*)-**1**, (*R*)-**12,** and (*R*)-**13** with benzyl-protected α- and β-methyl *gluco*dialdose derivatives **14** and **15,** followed by deprotection. ^a^The reaction was performed using 0.5 equiv. of BF_3_^.^Et_2_O; ^b^the reaction was performed using 1.5 equiv. of BF_3_^.^Et_2_O; ^c^the reaction was performed using 0.75 equiv. of BF_3_^.^Et_2_O.

Although the trimethylsilyl-protected dialdoses did indeed yield the free sugar-isochroman conjugates in one step, as expected, we were not satisfied with the yields. Therefore, in the hope of higher yields, we continued the study of the oxa-Pictet–Spengler reactions with benzyl-protected α- and β-methyl glucodialdoses **14**^27^ and **15**^28^, which were prepared by literature methods. In addition to the previously used dimethoxy-substituted alcohols, 1-(3,4,5-trimethoxyphenyl)propan-2-ols (*S*)-**12**^21^ and (*R*)-**12**^21^, and dibenzyloxy-substituted aryl-2-propanols (*S*)-**13**^29^ and (*R*)-**13**^30^ were also included in these studies. Using these starting materials, the free glycosyl isochroman final products were obtained in a two-step process involving cyclization followed by catalytic hydrogenolysis of the benzyl protecting groups. (Figs. 4 and 5). From the (*S*)-alcohols, in the presence of 1.5 equivalents of Lewis acid, complete conversion was observed with both Me-α and β-*gluco*dialdoses and, as expected, the corresponding 1,3-*cis*-substituted isochroman-sugar conjugates were formed with full stereoselectivity (Fig. 4). To our delight, complete conversion was also achieved with trimethoxy alcohol (*S*)-**12**, and the yields of the cyclized products **17** (75%) and **19** (72%) were similarly high as those of the isochromans formed by the disubstituted alcohols (**16**, **18,** and **20**, 74–80%). These prove that the 5-methoxy substituent of the alcohol does not interfere with the ring-closure reactions of benzyl-protected aldehydes.

During the reactions between alcohol (*S*)-**1** and dialdoses **14** and **15**, we investigated whether the amount of Lewis acid could be reduced without a significant decrease in conversion. We found that reducing the Lewis acid to 1 equivalent did not cause a noticeable change in the yield, but when only 0.5 equivalents of BF_3_^.^Et_2_O were used, the reaction did not proceed to completion, and the yields decreased by ~ 10% (61% for **16** and 65% for **18**). These results are consistent with those observed during the synthesis of bergenin-type sugar isochromans, where stoichiometric or slight excess BF_3_^.^Et_2_O was required to efficiently perform the oPS cyclization step^20^.

Removal of the benzyl protecting groups of **16**–**20** by catalytic hydrogenolysis upon Pd/C catalysis resulted in the final isochroman-sugar conjugates **3** and **21**–**24** in excellent yields. The overall yield of this two-step route was 73% for compound **3**, representing an increase of 16% compared to the silyl-protected dialdose-based route.

Reactions of the disubstituted alcohols (*R*)-**1** and (*R*)-**13** with dialdoses **14** and **15** afforded diastereomeric mixtures of 1,3-*cis* and 1,3-*trans*-substituted isochromans, **25** + **26**, **28** + **29**, and **31** + **32**, respectively (Fig. 5). Since we found in the reactions between alcohol (*R*)-**1** and silyl-protected dialdose **2** that the stereoselectivity of the cyclization could be reversed by increasing the amount of acid used (see Table 1), we investigated in these cases whether the amount of BF_3_^.^Et_2_O affects the stereochemical outcome of the ring-closure reactions. When substoichiometric amounts (0.5 or 0.75 equiv) of acid were used, a predominance of the 1,3-*cis* isomers (**25**, **28,** and **31**) was observed (**25**:**26** 46% *versus* 4%, **28**:**29** 54% *vs* 17% and **31**:**32** 41% vs 24%). When the amount of BF_3_^.^Et_2_O was increased to 1.5 equivalents, the stereoselectivity in the reaction with dimethoxy alcohol (*R*)-**1** was reversed, and the formation of 1,3-*trans*-substituted products (**26** and **29**) became predominant (**25**:**26** 23% vs 63%, **28**:**29** 33% vs 51%). This is consistent with the results of the reaction between (*R*)-**1** and dialdose **2** and supports our assumption that the 1,3-*cis* and -*trans* isomers may have similar thermodynamic stability and by fine-tuning the conditions the reaction can be shifted towards the formation of one or the other product. In contrast, in the reaction of dibenzyloxy alcohol (*R*)-**13** and dialdose **15**, the use of excess acid further increased the proportion of 1,3-*cis*-substituted product (**31**:**32** 69% vs 7%). This suggests that the bulky benzyloxy substituents on the aromatic core hinder the β-side (*trans*) cyclization, and the steric congestion in the 1,3-*trans*-substituted product exceeds that in the 1,3-*cis*-substituted product.

The cyclization reaction of trimethoxy alcohol (*R*)-**12** with both aldehydes **14** and **15** resulted in exclusively the corresponding 1,3-*cis*-substituted isochroman derivatives **27** and **30**. The exclusive formation of a single diastereomer can be explained by the higher steric congestion of the starting alcohol, which results in complete facial selectivity during the cyclization.

Deprotection of compounds **25**–**31** by catalytic hydrogenolysis proceeded smoothly, providing the final products **4**, **5**, and **33**–**37** in excellent yields of 82–96%.

**Fig. 6:**
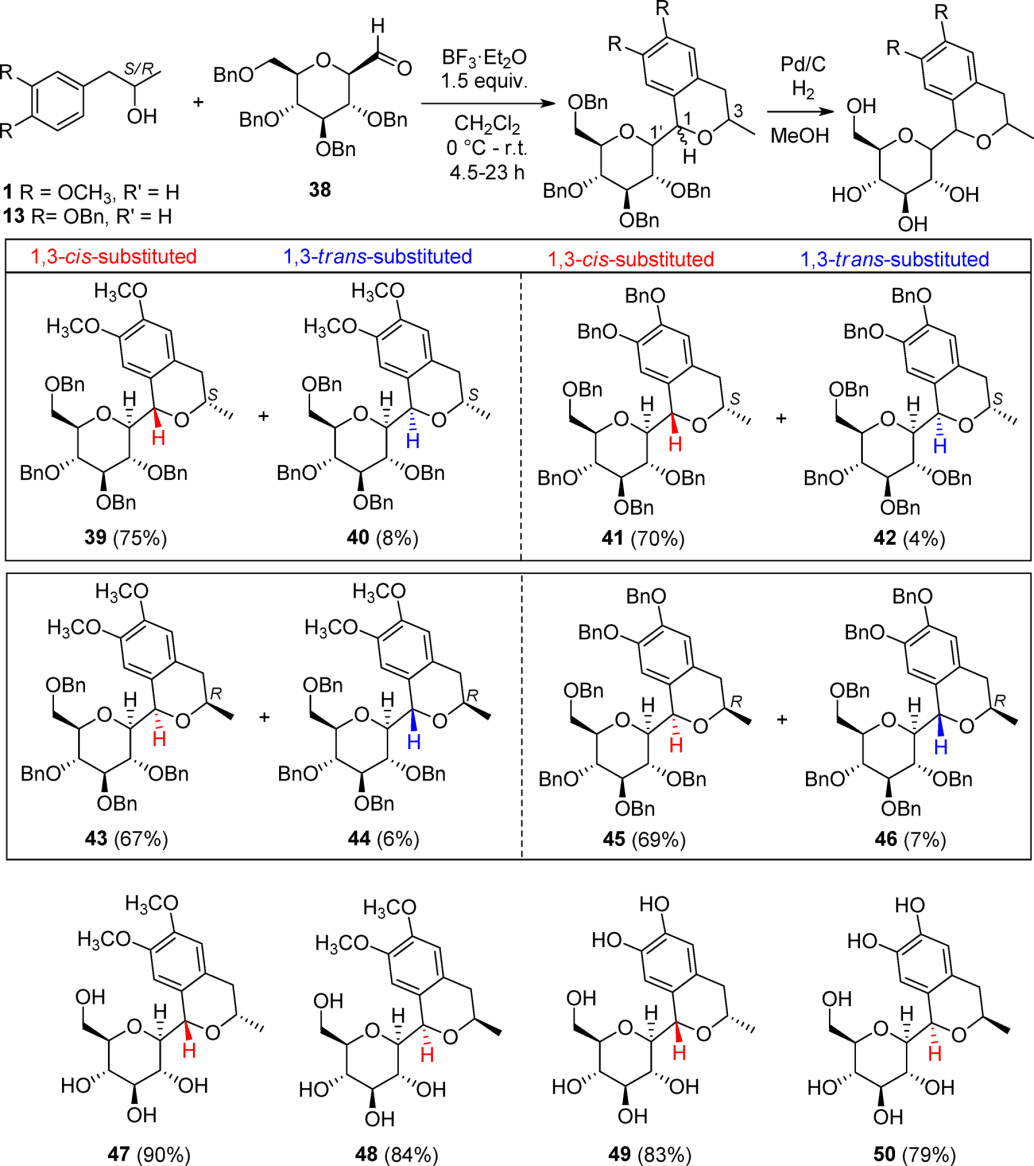
Synthesis of 1-(β-*C*-glucosyl)-isochroman derivatives by oxa-Pictet–Spengler cyclization of aryl-2-propanols (*S*)-**1**, (*R*)-**1**, (*S*)-**13**, and (*R*)-**13** with benzyl-protected β-formyl glucose **38**.

With the optimized conditions for the oxa-Pictet–Spengler cyclization of sugar dialdoses in our hands, we extended the approach to the synthesis of *C*-glycosyl isochromane derivatives, using 1-formyl glycoside as the oxo component. The benzyl-protected β-*C*-glucopyranosyl-formaldehyde **38** was prepared via a four-step literature procedure from tetra-*O*-benzyl-glucose with complete stereoselectivity in 37% overall yield (see SI for details)^31^. Compound **38** was reacted with both enantiomers of the dimethoxy and dibenzyloxy aryl-2-propanols (*S*)-**1**, (*R*)-**1**, (*S*)-**13,** and (*R*)-**13** using 1.5 equiv. of BF_3_·Et_2_O to produce the 1-(β-*C*-glucosyl)-isochroman derivatives **39**–**46** (Fig. 6).

Unexpectedly, the ring-closure reaction of the benzyl-protected β-formyl sugar with (*S*)- and (*R*)-alcohols led to completely identical stereochemical outcomes. In all reactions, the corresponding 1,3-*cis*-substituted isochromans were formed as the main products in high yields (67–75%) from both alcohol enantiomers. However, 1,3-*trans*-substituted isochroman glycosides were also formed in small amounts (4–8%) from both (*R*)- and (*S*)-alcohols, the latter being surprising, since *trans* products were previously only observed in the reactions with (*R*)-alcohols. The explanation for the high 1,3-*cis* selectivity observed in the reactions of (*R*)-alcohols with 1-formyl sugar is probably the preferential ring-closure with the bulky dialdose **38** on the alcohol from the *cis* side, similar to that observed in the reactions between (*R*)-**13** alcohol and dialdoses **14** and **15**.

Finally, the catalytic hydrogenation of the 1,3-*cis*-substituted isochroman derivatives **39**, **41**, **43,** and **45** gave the final product 1-(β-*C*-glucosyl)-isochromans **47**–**50** in high yields of 79–90%. The structure of **48** was confirmed by X-ray diffraction analysis (Figure S4).

### Stereochemical outcome and mechanism

Regarding the stereochemical outcomes of the oxa-Pictet–Spengler reactions, the complete stereoselectivity of the cyclizations with (*S*)- and (*R*)-alcohols can be explained by the principle of double stereodifferentiation^32,33^. The absolute configuration of the newly established C-1 chirality center is affected by the configurations of both the optically active alcohol and the sugar aldehyde. Our results show that the investigated chiral d-carbohydrate derivatives and chiral alcohols with (*S*)-configuration form sterically matched pairs in the transition state leading to the *cis* product with high selectivity, in which both the C-3 methyl group and the C-1 glycosidic bond adopt *equatorial* orientation (For details, see the *DFT conformational analysis* section). In the reaction of (*S*)-alcohols and d-sugar aldehydes, the *trans* products appear with *quasi-axial* C-1 glycosyl bond, which suggests a mismatch differentiation.

**Fig. 7:**
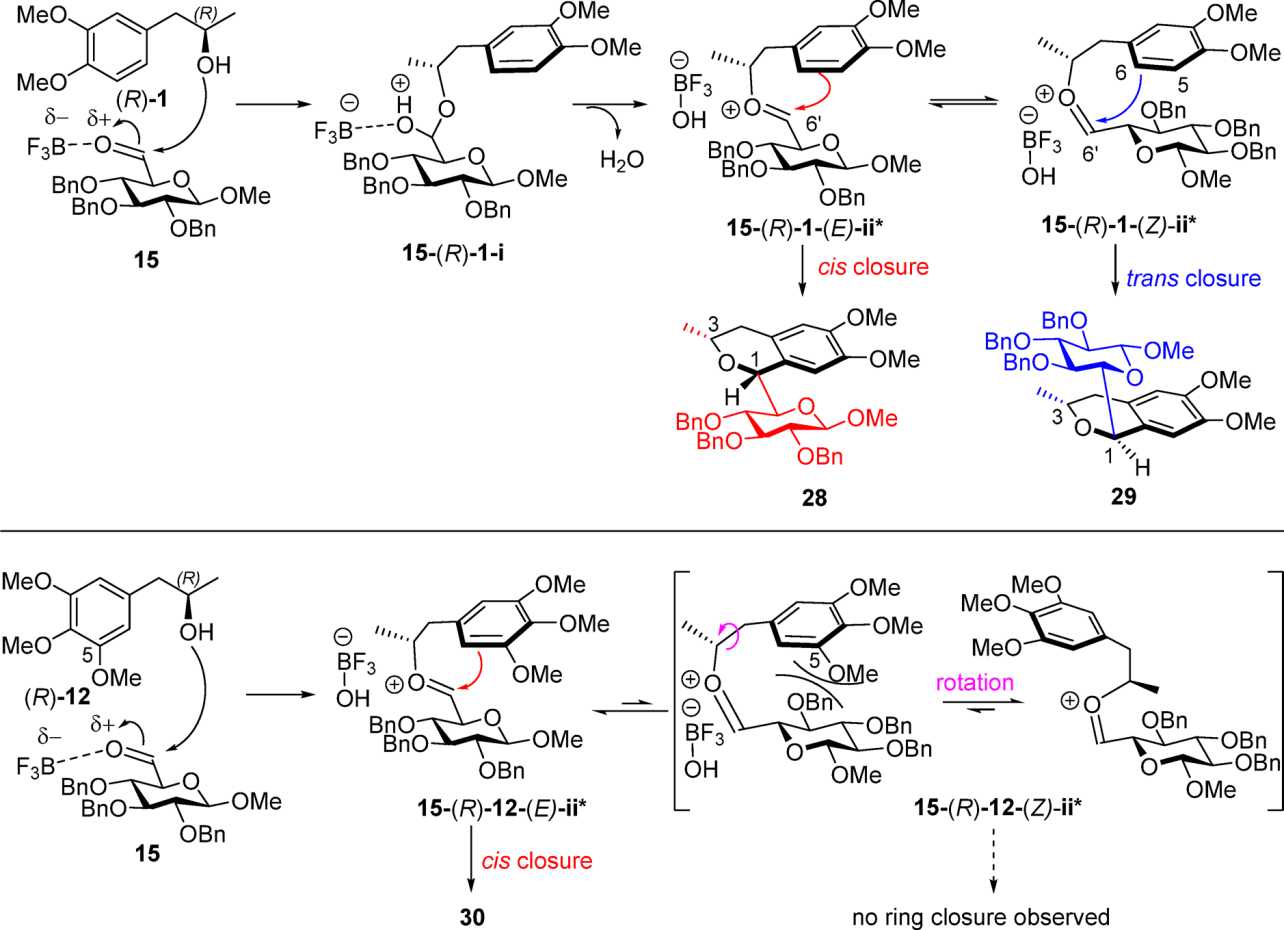
Mechanism of *cis* and *trans* ring closures via (*E*) and (*Z*) oxocarbenium ion intermediates in the reactions of **15** with disubsituted (top panel) and trisubstituted (bottom panel) (*R*)-alcohols **1** and **12**.

It is known from the literature that the *trans* product arises through the formation and ring closure of the (*Z*)-oxocarbenium ion, while the *cis* product arises through the (*E*)-oxocarbenium ion^34–36^. With an excess of an acid and prolonged reaction time, the isochroman ring can also undergo a ring-opening reaction and subsequent cyclization can induce isomerization at the C-1 favoring the thermodynamic product. In the reactions of dimethoxy- and dibenzyloxy-substituted (*R*)-alcohols, diastereomeric mixtures are formed, and the geometry of the intermediate oxocarbenium ions plays an important role in the ratio of stereoisomers. As demonstrated in the examples of **1**-(*R*) alcohol and benzyl-protected β-glucoside **15** (Fig. 7, top panel), the sugar ring is *exo*-positioned to the aromatic ring in the (*E*)-oxocarbenium ion. Consequently, the electrophilic 6’-C atom of the glucose unit can initiate an intramolecular S_E_Ar reaction with the aromatic ring without steric hindrance, and the ring closure can easily occur, generating the 1,3-*cis* isochroman product. The (*Z*)-oxocarbenium ion, despite the *endo*-position of the sugar ring, can adopt a conformation with the appropriate geometry to bring the reactive centers close to each other, allowing cyclization, leading to 1,3-*trans* products.

In contrast, in the reactions of trimethoxy (*R*)-**12** alcohol (Fig. 7, bottom panel), the 5-methoxy substituent of the aromatic ring in the **12**-(*Z*)-oxocarbenium ion sterically hinders the proximity of the reactive centers, which prevents cyclization. The same steric hindrance may occur in the (*Z*)-oxocarbenium ions formed from the (*S*)-**12** alcohol, which explains why we did not observe *trans*-cyclization in the reactions of either (*R*)- and (*S*)-trimethoxy alcohol. However, in the **12**-(*E*)-oxocarbenium ion, the two reactive centers easily come close to each other without steric hindrance, allowing ring closure and yielding the 1,3-*cis* isochroman product with complete stereoselectivity.

### VCD analysis

Although the absolute configuration of the compounds under discussion was apparent from the X-ray analysis and NMR measurements, the novel 1-(*C*-glycosyl)isochromans appeared to be interesting targets for VCD studies. VCD is a powerful tool for the elucidation of absolute configuration of carbohydrates^37–40^, and due to the higher number of transitions, it can also be applied to distinguish stereoisomers^41–43^.

The VCD spectra of six novel isochroman-carbohydrate hybrids were recorded in DMSO-d_6_ or MeOH-d_4_ and were compared pairwise to check the effect of different absolute configuration (AC) in the two blocks of chirality.

**Fig. 8:**
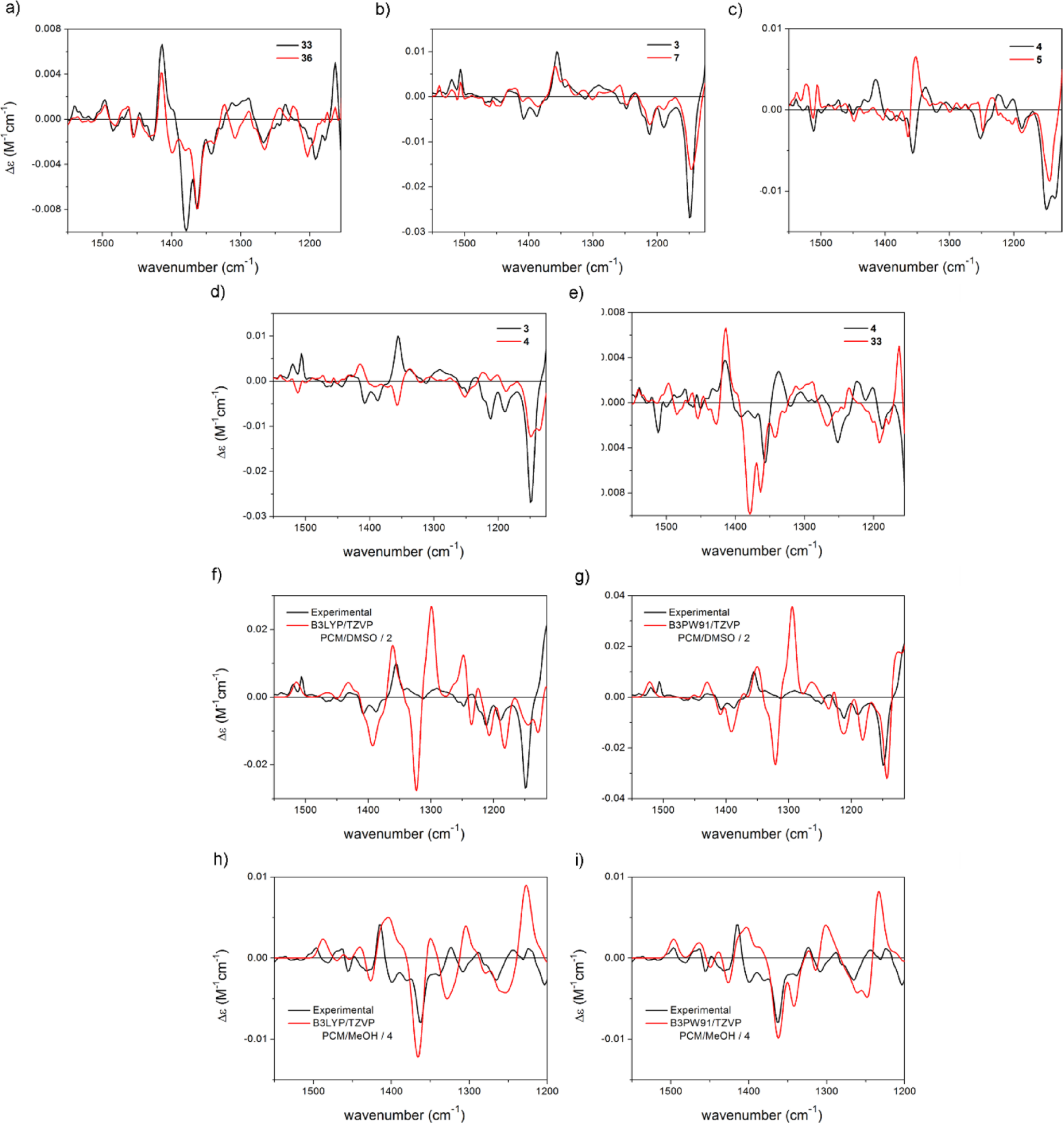
Comparison of the experimental VCD spectra of epimeric isochroman-carbohydrate conjugates, (**a**) C-1’ epimeric α- and β-D-*gluco*pyranosides **33** (black) and **36** (red), (**b**) C-4’ epimeric α-D-*gluco*pyranoside **3** (black) vs α-D-*galacto*pyranoside **7** (red), and (**c**) C-1 epimers **4** [(1*R*), black] and **5** [(1*S*), red]. Comparison of the experimental VCD spectra of related isochroman-carbohydrate conjugates; (**d**) **3** (black) and **4** (red) differing in the AC of two stereocenters of the isochroman unit, (**e**) dimethoxy **4** (black) vs trimethoxy **33** (red) derivatives with identical stereochemistry, differing only in the degree of substitution. Comparison of the experimental and calculated VCD spectra of **3** (**f** and **g**) and **36** (h and i); calculations were performed at the B3LYP/TZVP PCM (**f** and **h**) and the B3PW91/TZVP PCM (**g** and **i**) levels.

The experimental VCD spectra of the anomeric methyl α- and β-D-glucopyranosides **33** and **36** were found quite similar for the 1150–1550 region but significant differences could be identified around 1380 and 1305 cm^−1^ (Fig. 8a). Furthermore, the negative transition of **33** at 1192 cm^−1^ shifted to 1204 nm in **36**. The change of the AC at the anomeric C-1’ has relatively small effect on the VCD spectra, which might be reproduced with calculations. The cosine similarity index (CSI) computed for **33** and **36** was in line with this observation with a value of 0.74.

The C-4’ epimers **3** and **7**, containing a *gluco* and *galacto* carbohydrate moiety, respectively, had near congruent VCD spectra (Fig. 8b). The 1125–1550 cm^−1^ range looks very similar, with only minor intensity differences at 1190 nm or the 1380–1410 cm^−1^ region, and two weak oppositely signed transitions were identified at 1312 cm^−1^ and 1300 cm^−1^. The CSI value computed for **3** and **7** was found to be 0.89. Consequently, the *gluco–galacto* configuration of **3** and **7** could be very challenging to distinguish and assign by VCD spectra.

Larger differences were found in the VCD spectra of the C-1 epimers **4** and **5**. The intense negative 1358 cm^−1^ transition of **4** corresponds to an intense positive one in **5**, while further oppositely signed VCD transitions can be found with moderate intensity at 1256, 1232 or 1202 cm^−1^ (Fig. 8c). Accordingly, the CSI value computed for **4** and **5** was 0.56.

Comparison of the VCD spectra of (1*R*,3*S*)-**3** and (1*S*,3*R*)-**4** with different AC at C-1 and C-3 of the isochroman units indicated oppositely signed transitions at 1130, 1200, 1220, 1235, 1310, 1355, 1410, 1442, 1470, 1498 and 1510 cm^−1^ (Fig. 8d). The CSI value computed for **3** and **4** was found to be 0.51.

A difference in a single OMe group on the aromatic ring at C-8 resulted in a sign change of several VCD transitions and a considerable blue shift in the 1175–1390 cm^−1^ region when comparing the VCD spectra of the trimethoxy and dimethoxy derivatives **33** and **4** with identical absolute configuration (Fig. 8e). The CSI value computed for **4** and **33** was 0.37, the lowest one in the studied pairs.

In order to reproduce the experimental VCD spectrum of **3**, a Merck Molecular Force Field (MMFF) conformational search was performed first, yielding 90 conformers in a 21 kJ/mol energy window. B3LYP/TZVP PCM/DMSO re-optimization of the MMFF conformers resulted in 10 low-energy conformers over 1% Boltzmann population. VCD spectra computed for these conformers gave moderate agreement with the experimental VCD spectrum,with CSI = 0.33 (Fig. 8f). To improve the agreement, the DFT re-optimization of the MMFF conformers and the VCD calculations were repeated with the more advanced B3PW91 functional^44–46^, which increased the similarity index to 0.56 (Fig. 8g). The moderate agreement is attributed to the strong solute–solvent interactions of the DMSO and the hydroxyl groups of **3** that are hard to model with the classical conformational analysis-based approaches^47^. The calculations systematically overestimated the negative VCD couplet around 1300 cm^−1^.

A similar VCD calculation approach was applied to **36**, the VCD spectra of which was measured in MeOH-d_6_. The B3LYP/TZVP PCM/MeOH re-optimization of the initial 65 MMFF conformers resulted in 13 low-energy conformers over 1% Boltzmann population. The Boltzmann averaged B3LYP/TZVP PCM/MeOHVCD spectrum computed for these conformers reproduced the major transitions of the experimental VCD spectrum with a CSI value of 0.46 (Fig. 8h). The use of the advanced B3PW91 functional improved the overall agreement as justified by the higher CSI value of 0.58 (Fig. 8i) However, the difference between the experimental and the calculated VCD spectra is still larger than that found between experimental VCD spectra of epimers.

The experimental VCD spectra of the isochroman-carbohydrate conjugates were found useful to distinguish epimers or diastereomers in the presence of several matching stereogenic elements. However, in most cases, the identified differences were found difficult to be reproduced by VCD calculations, when strongly interacting solvents such as DMSO-d_6_ or MeOH-d_4_ were utilized.

### DFT conformational analysis of C-1 epimers

In order to rationalize the observed *cis/trans* selectivity, conformational analysis was performed for some selected 1,3-*cis* and -*trans* cyclization product pairs such as **3**/*trans-***3** (C-1epimer of **3**), **4**/**5**, **31mod**/**32mod** and **34**/**35**. The epimeric pairs **31mod**/**32mod** and **34**/**35** model the cyclization epimeric products **31**/**32** and **28**/**29** but the benzyloxy groups of the carbohydrate moieties were replaced by hydroxyl groups to reduce the conformational flexibility and the size of the molecules. The initial few hundreds MMFF conformers were re-optimized at the ωB97X/TZVP PCM/CH_2_Cl_2_ level to mimic the reaction conditions. The energies of the lowest-energy conformers of the epimeric pairs differed by 0.33–2.86 kJ/mol, and with the exception of the **34**/**35** pair, the 1,3-*trans* products had higher energy. For the pair **3**/*trans-***3**, the *trans-***3** had 2.00 kJ/mol higher energy corresponding to populations with 69% and 31%, respectively. For the pair **31mod**/ **32mod**, the difference is 2.86 kJ/mol corresponding to 76% and 24% populations. These computed energy values of the products are consistent with the exclusive or dominant formation of the 1,3-*cis* products (**3**, **16** and **31**) observed in the related reactions. For the pair **4**/ **5**, the energy difference is only 0.33 kJ/mol corresponding to a 53% to 47% ratio, while in the case of **34**/**35** the former 1,3-*cis* product had 2.30 kJ/mol higher energy corresponding to a 28% to 72% ratio. These results are in line with the observation that in the related reactions, in addition to the kinetic 1,3-*cis* products, 1,3-*trans* products are also formed, and the proportion of the latter increases with increasing acid excess and reaction time (see the formation of **4**/**5**, **25**/**26** and **28**/**29** epimeric mixtures, Figs. 2 and 5).

**Fig. 9:**
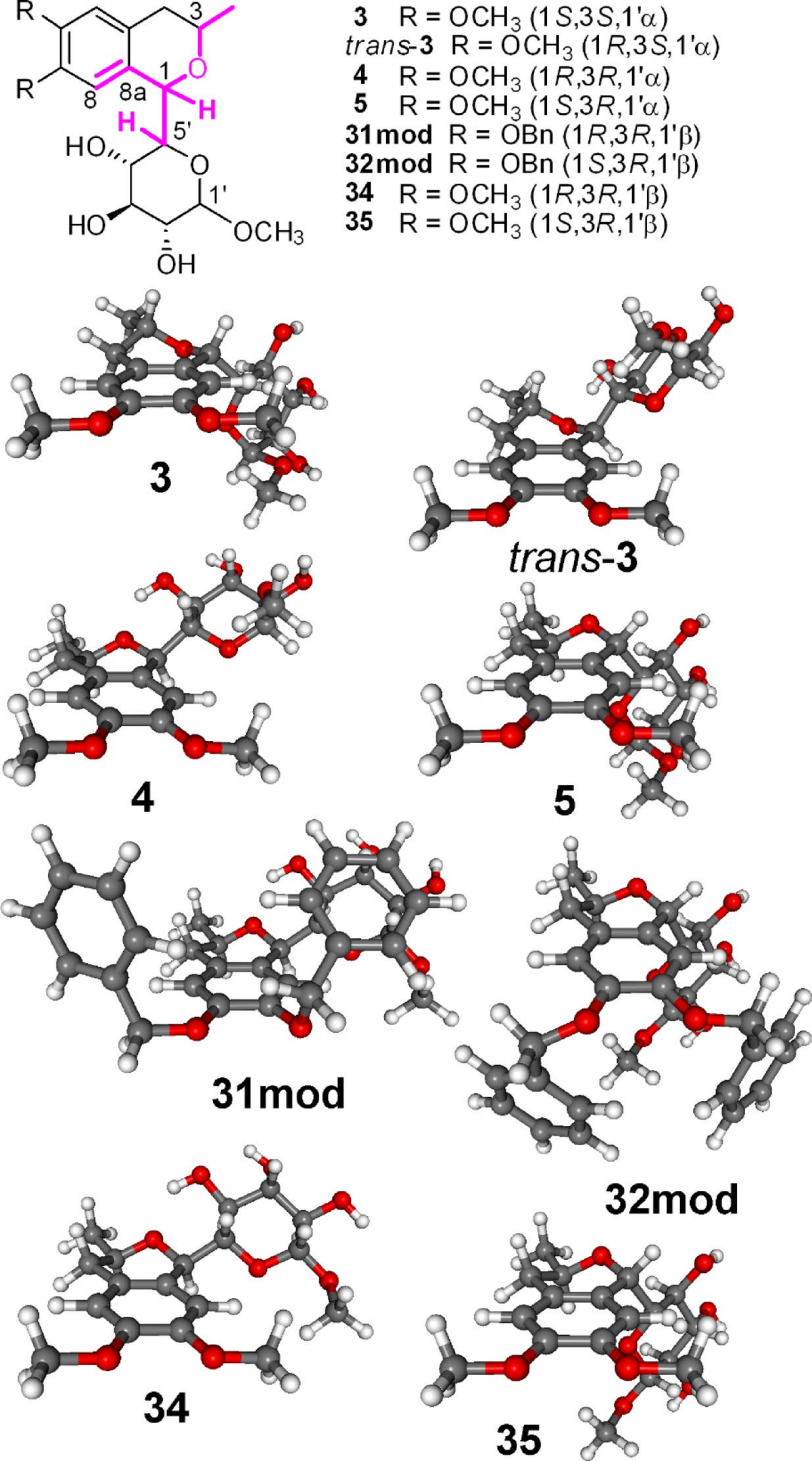
Lowest-energy conformers of four epimeric pairs calculated at the ωB97X/TZVP PCM/CH_2_Cl_2_ level.

The carbohydrate moieties show a ^4^C_1_ conformation for all studied derivatives as expected (Fig. 9). For all six studied derivatives with (3*R*) absolute configuration, the heteroring of the isochroman moiety had *M*-helicity, while the two investigated derivatives with (3*S*) absolute configuration contained a heteroring with *P* helicity. The C-3 methyl group adopted an equatorial position in both the *cis* and *trans* derivatives. The C-1 carbohydrate moiety of the *cis* derivatives **3**, **4**, **31mod** and **34** adopted an equatorial or pseudoequatorial position with an axial orientation of the 1-H (for relevant bond angles, see Table S2 in the SI). In the trans derivatives *trans*-**3**, **5**, **32mod** and **35**, the C-1 carbohydrate moiety had a pseudoaxial orientation, which is attributed to the distortion of the half-chair conformation of the condensed heteroring toward an envelop conformation, in which the oxygen atom shifts to the plane of the benzene ring.

## Conclusion

The oxa-Pictet–Spengler cyclization reaction was applied to the efficient synthesis of new isochroman-carbohydrate conjugates, 1-(C-glycosyl)isochromans. The 1-aryl-2-propanol enantiomers containing methoxy or benzyloxy substituents in the aromatic core reacted readily with sugar aldehydes, including glucopyranosyl dialdoses and 1-formylglucopyranosides to afford 1,3-*cis* 1-(C-glycosyl)isochromans in high yields with good to complete stereoselectivity. When the reaction was extended to galacto-dialdopyranose **6**, moderate yields, significantly reduced stereoselectivity, and anomerization side reactions were observed, probably due to steric hindrance by the axial C4-OH group of galactose, which represents a slight limitation to the universality of the method.

Among the acids tested, BF_3_^.^Et_2_O was much more efficient than CSA. Although persilylated pyranosyl dialdoses provided the deprotected glycosyl isochroman final products in one step, the reactions of perbenzylated pyranosyl aldehydes were still more advantageous because they resulted in complete conversions and higher overall yields without isomerization. The (*S*)-alcohols provided 1,3-*cis* isochromans with complete or high stereoselectivity regardless of the substitution of the aromatic nucleus, and exclusive stereoselectivity was also observed in the ring closure reactions of trisubstituted (*R*)-alcohols. A mixture of 1,3-*cis* and 1,3-*trans* isochromans was formed from dimethoxy- and dibenzyloxy-(*R*)-alcohols, the diastereomeric ratio depended on the steric congestion of the reactants and the amount of acid used. Moreover, the stereoselectivity could be increased or reversed by increasing the excess of acid. A total of 21 benzyl-protected and 20 free 1-(C-glycosyl)isochroman derivatives were prepared, which contain five chirality centers on the sugar moiety and two on the isochroman backbone. The absolute configuration of the C-1 chiral center formed in the oPS reaction was determined by ROESY NMR measurements, and in the case of four derivatives (**3**, **7**, **24** and **48**) planar structure and stereochemistry were also confirmed by single crystal X-ray diffraction analysis^24^.

Conformational analysis have shown that the heteroring of isochromans has a distorted half-chair conformation, when the C-1 pyranose ring is equatorially oriented in the 1,3-*cis* products and axially oriented in the 1,3-*trans* products. The former ones are generally more stable, which may explain the observed prevalence of the 1,3-*cis* isomers. The computed relative energies of the epimeric products corroborated well the isolated yields of the epimeric products. Our VCD measurement and calculation experiments on diastereomeric pairs have shown the scope and limitations of the VCD method to distinguish epimers or stereoisomeric derivatives containing seven chirality centers.

## Supplementary Information

Below is the link to the electronic supplementary material.

Supplementary Material 1

## Data Availability

All data generated or analysed during this study are included in this published article and its Supplementary Information files. The supplementary crystallographic data for each compound can be obtained free of charge from the Cambridge Crystallographic Data Centre via http://www.ccdc.cam.ac.uk/data_request/cif using reference deposition numbers: 2494726 for compound **3**, 2494727 for compound **7**, 2494728 for compound **24** and 2494729 for compound **48**. Before publication of this paper, the supplementary crystallographic data are available via this link: https://www.ccdc.cam.ac.uk/structures/search?Ccdc=2494726,2494727,2494728,2494729&Author=Benyei&Access=referee
