# Supplementary material for: Stereoselective synthesis, VCD study and conformational analysis of C-glycosyl isochromans
Source: Sci Rep. 2026 Apr 24;16:19090. doi: 10.1038/s41598-026-46290-7 (PMC13280479; doi:10.1038/s41598-026-46290-7)
Supplement: Supplementary file 1 — Supplementary Material 1 [file 41598_2026_46290_MOESM1_ESM.pdf]

SUPPORTING INFROMATION

C-glycosyl isochromans – Stereoselective synthesis, VCD study and conformational analysis

Nawar Ahmad,[1,2] Ágnes Homolya,[1] Roland A. Barta,[3,4] Mihály Herczeg,[1] Attila Bényei,[5] Nika Iurgenson,[5] Ilona Bereczki,[1] Gergely M. Fedics,[3] Attila Mándi,[3]* Tibor Kurtán,[3]* Anikó Borbás[1]*

[1] Department of Pharmaceutical Chemistry, University of Debrecen, Egyetem tér 1, Debrecen, H-4032, Hungary

[2] Doctoral School of Pharmaceutical Sciences, University of Debrecen, Egyetem tér 1, Debrecen, H-4032, Hungary

[3] Department of Organic Chemistry, University of Debrecen, P.O. Box 400, 4002 Debrecen, Hungary

[4] Doctoral School of Chemistry, University of Debrecen, Egyetem tér 1, Debrecen, H-4032, Hungary

[5] Department of Physical Chemistry, University of Debrecen, Egyetem tér 1, Debrecen, H-4032, Hungary

Table of Content

Experimental Section 2

General information 2

Synthesis of trisilylated hexodialdoses from methyl α-D-glucoside and -galactoside 2

Synthesis of tribenzylated hexodialdoses from methyl α- and β-D-glucosides 4

General method for the oxa-Pictet–Spengler cyclization reaction with silyl protecting groups (Method A): 6

General method for the oxa-Pictet–Spengler cyclization reaction with benzyl protecting groups (Method B): 6

General method for debenzylation (Method C): 6

Synthesis of isochroman-sugar hybrids using hexodialdo-1,5-pyranosides 8

Synthesis of tetra-*O*-benzyl-β-*C*-glucopyranosyl formaldehyde (**38**) 25

Synthesis of isochroman-sugar hybrids from tetra-*O*-benzyl-β-*C*-glucopyranosyl-formaldehyde 27

Determination of the C-1 configuration by ^{1}H-^{1}H ROESY NMR experiments 33

X-ray diffraction experiments 37

X-ray structures of compounds **3, 7, 24, 48** 39

Computational Section 40

Quantitative comparison of VCD spectra 41

Cartesian coordinates and energies of the low-energy conformers 42

References 71
COPY of NMR SPECTRA.. 73

Experimental Section

General information

The solvents were purified by distillation. THF was freshly distillied from benzophenone and sodium. TLC analysis was performed on Kieselgel 60 F_{254} (Merck) silicagel plates with visualization by immersing in a sulfuric acid solution (5% in EtOH) followed by heating. Column chromatography was performed on silica gel 60 (Merck 0.063–0.200 mm), and flash column chromatography was performed on Silica gel 60 (Merck 0.040-0.063 mm). The 1D (^{1}H, ^{13}C) and 2D (COSY, CLIP-COSY, HSQC, HMBC, ROESY) spectra were recorded with Bruker Avance I 400 MHz (400/101 MHz for ^{1}H/^{13}C), Bruker Avance II 500 MHz (500/126 MHz for ^{1}H/^{13}C), and Bruker Avance Neo 700 MHz (700/176 MHz for ^{1}H/^{13}C) spectrometers at 25 ºC. Chemical shifts values (δ) are given in ppm are referenced to $SiMe_4$ or the residual solvent signal ($SiMe_4$: 0.00 ppm, CD_3SOCD_3: 2.50 ppm for ^{1}H) and residual solvent signals ($CDCl_3$: 77.16 ppm, CD_3OD: 49.00 ppm, and CD_3SOCD_3: 39.52 ppm for ^{13}C) while homonuclear coupling constants ($^3J_{H\text{-}H}$) are given in Hz. For the MALDI-ToF MS measurements a Bruker Autoflex Speed mass spectrometer equipped with a time-of-flight (ToF) mass analyzer was used. During the measurements in all cases 19 kV (ion source voltage 1) and 16.65 kV (ion source voltage 2) were used. For reflectron mode 21 kV and 9.55 kV were applied as reflector voltage 1 and reflector voltage 2. To produce laser desorption a solid phase laser (355 nm, ≥100 µJ/pulse) operating at 500 Hz was applied. 2,5-Dihydroxybenzoic acid (DHB) was used as matrix and CF_3COONa as cationising agent in DMF [1]. Optical rotations were measured at room temperature on a Perkin-Elmer 241 automatic polarimeter. Melting points were measured in open capillary tubes on a Büchi Melting Point B-540 apparatus.

VCD spectra were recorded on a BioTools ChiralIR-2X spectrophotometer at a resolution of 4 cm^{-1} under ambient temperature for 18 x 3000 scans, respectively. Samples were dissolved in CD_3SOCD_3 and CD_3OD, and the concentrations were between 0.135 M and 0.150 M, and the solution was placed in a 100 µm BaF_2 cell.

Synthesis of trisilylated hexodialdoses from methyl α-D-glucoside and -galactoside

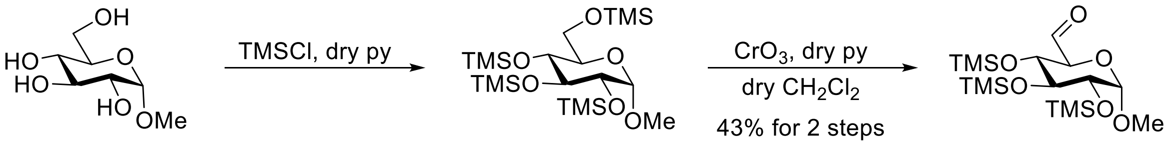

2.1 2.2 2

Scheme S1. Synthesis of glucose-derived 6-aldehyde using silyl protecting groups.

Methyl 2,3,4-tris-*O*-(trimethylsilyl)-α-D-*gluco*-hexodialdo-1,5-pyranoside (2) [2, 3]
Step 1. To a solution of methyl α-D-glucopyranoside (5.0 g, 26 mmol, 1 equiv.) in dry pyridine (25 mL) at 0 °C, TMSCl (15.5 mL, 0.124 mol, 1.2 equiv./OH group) was added, and the reaction mixture was stirred overnight. The progress of the reaction was monitored by TLC (hexane: EtOAc 95:5). After the starting material was consumed, the reaction mixture was diluted with diethyl ether (200 mL) and washed with water (3×100 mL). The organic phase was dried over $MgSO_4$ and concentrated in vacuo to afford the per-trimethylsilylated sugar as a colorless syrup, which was used in the next step without further purification.

Step 2. CrO_3 was dried over P_2O_5. CrO_3 (15.5 g, 0.155 mol, 6 equiv.) was suspended in dry CH_2Cl_2 (520 mL) and dry pyridine (24.8 mL) at 0 °C and it was stirred for 15 min. The persilylated sugar from the previous step (~12 g, 26 mmol, 1 equiv.) was dissolved in dry CH_2Cl_2 (35 mL) and was added to the suspension dropwise at 0 °C. The reaction mixture was stirred at room temperature for 2 h, filtered through a silica bed, washed with CH_2Cl_2, and then evaporated. The reaction mixture was purified by flash column chromatography (CH_2Cl_2: acetone 98:2) to afford compound **2** (6 g, 56%) as a colorless syrup. R_f = 0.18 (CH_2Cl_2: acetone 98:2). $[\alpha]_D$ +29.0 (c 0.21, $CHCl_3$). ^1H NMR (500 MHz, $CDCl_3$) δ (ppm): 9.70 (d, J = 1.2 Hz, 1H, H-6), 4.69 (d, J = 3.4 Hz, 1H, H-1), 4.10 (dd, J = 10.0, 1.6 Hz, 1H, H-5), 3.83 (t, J = 8.7 Hz, 1H, H-3/H-4), 3.56 (t, J = 9.1 Hz, 1H, H-3/H-4), 3.49 (dd, J = 9.1, 3.5 Hz, 1H, H-2), 3.35 (s, 3H, OCH_3), 0.15 – 0.11 (m, 27H, 3 x $(CH_3)_3Si$). ^{13}C NMR (126 MHz, $CDCl_3$) δ (ppm): 198.6 (1C, C-6), 100.4 (1C, C-1), 75.8, 74.9, 73.4, 72.9 (4C, C-2 – C-5), 55.6 (1C, OCH_3), 1.3, 1.0, 0.5 (9C, 3 x $(CH_3)_3Si$). The characteristic analytical data of the compound are consistent with those reported in the literature.

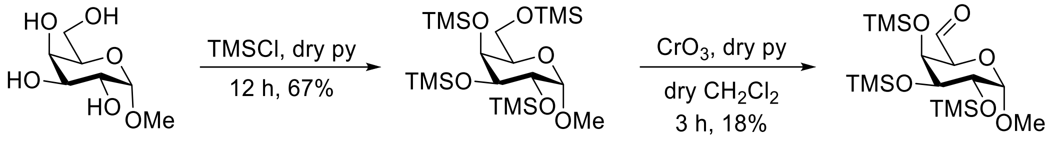

6.1 **6.2** **6**

Scheme S2. Synthesis of α-D-galactose-derived 6-aldehyde using silyl protecting groups.

Methyl 2,3,4-tris-*O*-(trimethylsilyl)-α-D-*galacto*-hexodialdo-1,5-pyranoside (6)

Step1: To a solution of methyl α-D-galactopyranoside (5.0 g, 26 mmol, 1 equiv.) in dry pyridine (30 mL) at 0 °C, TMSCl (15.5 mL, 122 mmol, 4.76 equiv., 1.2 equiv./OH group) was added dropwise, and the reaction mixture was allowed to warm up and stirred overnight at room temperature. The reaction mixture was diluted with diethyl ether (200 mL) and extracted with water (3×50 mL). The organic layer was dried over $MgSO_4$, concentrated in vacuum, and then dried over P_2O_5 to afford the corresponding persilylated sugar (8.3 g, 67%) as a colorless syrup, which was used in the next step without further purification. ^1H NMR (700 MHz, $CDCl_3$) δ (ppm): 4.63 (d, J = 3.7 Hz, 1H, H-1), 3.93 (dd, J = 9.5, 3.7 Hz, 1H, H-2), 3.90 (d, J = 2.8 Hz, 1H, H-4), 3.81 (dd, J = 9.5, 2.9 Hz, 1H, H-3), 3.72 (t, J = 6.7 Hz, 1H, H-5), 3.64 (dd, J = 9.7, 7.4 Hz, 1H, H-6a), 3.59 (dd, J = 9.7, 6.0 Hz, 1H, H-6b), 3.37 (s, 3H, OCH_3), 0.15 (s, 9H, $(CH_3)_3Si$), 0.14 (s, 9H, $(CH_3)_3Si$), 0.14 (s, 9H, $(CH_3)_3Si$), 0.12 (s, 9H, $(CH_3)_3Si$). ^{13}C NMR (176 MHz, $CDCl_3$) δ (ppm): 100.8 (1C, C-1), 72.4, 71.1, 69.9 (4C, C-2 – C-5), 61.4 (1C, C-6), 55.4 (1C, OCH_3), 0.7, 0.7, 0.4, -0.3 (12C, 4 x $(CH_3)_3Si$).

Step2: CrO_3 was dried over P_2O_5. CrO_3 (8.6 g, 86 mmol, 5 equiv) was suspended in dry CH_2Cl_2 (290 mL) and dry pyridine (16 mL) at 0 °C with continuous stirring for 15 min. The persilylated sugar **6.2** (8.3 g, 18 mmol, 1 equiv.) from the previous step was dissolved in dry CH_2Cl_2 (22 mL) and was added to the suspension dropwise at 0 °C. The reaction mixture was stirred at rt for 3 h, filtered through a silica bed, washed with CH_2Cl_2, and then evaporated. The reaction mixture was purified by flash column chromatography (CH_2Cl_2: acetone 98:2) to afford compound **6** (1.2 g, 18%) as a colorless syrup. R_f = 0.40 (CH_2Cl_2: acetone 98:2). $[\alpha]_D$ +55.8 (c 0.095, $CHCl_3$). ^1H NMR (700 MHz, $CDCl_3$) δ (ppm): 9.60 (s, 1H, CHO), 4.80 (d, J = 3.5 Hz, 1H, H-1), 4.25 (dd, J = 2.8, 1.7 Hz, 1H, H-4), 4.08 (d, J = 1.6 Hz, 1H, H-5), 3.99 (dd, J = 9.4, 3.5 Hz, 1H, H-2), 3.83 (dd, J = 9.4, 2.9 Hz, 1H, H-3), 3.43 (s, 3H, OCH_3), 0.16 (s, 9H, $(CH_3)_3Si$), 0.15 (s, 9H, $(CH_3)_3Si$), 0.10 (s, 9H, $(CH_3)_3Si$). ^{13}C NMR (176 MHz, $CDCl_3$) δ (ppm): 201.3 (1C, CHO), 101.2 (1C, C-1), 76.9, 76.5, 73.5, 70.3, 69.4 (5C, C-2 – C-6), 56.1 (OCH_3), 0.6, 0.6, 0.4 (9C, 3 x $(CH_3)_3Si$). MALDI-ToF HRMS: m\z calcd for $C_{16}H_{36}NaO_6Si_3^+$ $[M+Na]^+$ 431.1712, found 431.1715.

Synthesis of tribenzylated hexodialdoses from methyl α- and β-D-glucosides

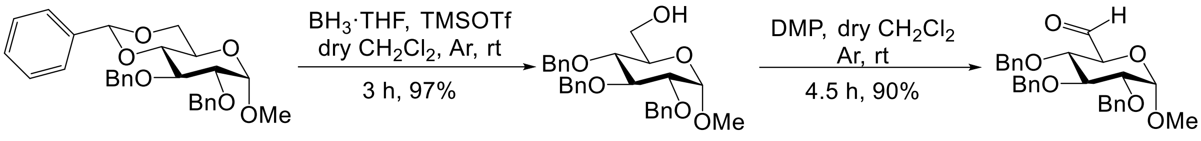

14.1 **14.2** **14**

Scheme S3. Synthesis of methyl α-D-glucoside-derived 6-aldehyde using benzyl protecting groups. (DMP: Dess-Martin periodinane)

Methyl 2,3,4-tri-*O*-benzyl-α-D-glucopyranoside (14.2): This product was prepared according to the literature with some modifications [4]. To a solution of methyl 2,3-di-*O*-benzyl-4,6-*O*-benzylidene-α-D-glucopyranoside **14.1** (2 g, 4.3 mmol, 1 equiv.) in dry CH_2Cl_2 (45 mL), BH_3·THF complex (1M in THF, 22 mL, 22 mmol, 5 equiv.) and TMSOTf (118 µL, 0.65 mmol, 0.15 equiv.) were added. The reaction mixture was monitored by TLC and was stirred at room temperature under argon atmosphere for 3 h. After complete conversion, Et_3N (3 mL) was added, followed by the careful addition of MeOH until the H_2 gas evolution ceased, and the residue was co-evaporated with MeOH (3×20 mL). The crude product was purified by flash column chromatography on silica gel (hexane: EtOAc 3:2) to afford compound **14.2** (2.00 g, 97%) as white crystals. R_f = 0.40 (hexane: EtOAc 1:1). M.p. 50-52 °C. $[\alpha]_D$ +19.6 (c 0.24, $CHCl_3$). M.p., $[\alpha]_D$, ^{1}H, ^{13}C NMR, and MALDI-ToF MS data of the compound are consistent with those reported in the literature [5].

Methyl 2,3,4-tri-*O*-benzyl-α-D-*gluco*-hexodialdo-1,5-pyranoside (14): Methyl 2,3,4-tri-*O*-benzyl-α-D-glucopyranoside **14.2** (505 mg, 1.08 mmol, 1 equiv.) was dissolved in dry CH_2Cl_2 (14 mL), and Dess-Martin periodinane (DMP, 682 mg, 1.61 mmol, 1.5 equiv.) was added, followed by stirring at room temperature. The reaction mixture was monitored by TLC (CH_2Cl_2: acetone 95:5). After 4.5 hours, the reaction mixture was diluted with CH_2Cl_2, and aq. NaOH solution (1.3 M, 6 mL) was added with continuous stirring for 10 min. The organic layer was extracted with water to neutral pH. The organic phase was dried over $MgSO_4$, filtered, and concentrated in vacuum, then co-evaporated with toluene. The crude product was used in the cyclization reactions without further purification as a colorless syrup (450 mg, 90%). R_f = 0.57 (CH_2Cl_2: acetone 95:5). $[\alpha]_D$ +14.0 (c 0.05, $CHCl_3$), lit. $[\alpha]_D$ +13.6 (c 2.6, $CHCl_3$) [6]. ^{1}H NMR (500 MHz, $CDCl_3$) δ (ppm): 9.64 (s, 1H, CHO), 7.36 – 7.24 (m, 15H, aromatic), 5.00 (d, J = 10.9 Hz, 1H, Ph-**CH**), 4.86 (d, J = 11.0 Hz, 1H, Ph-**CH**), 4.84 (d, J = 11.0 Hz, 1H, Ph-**CH**), 4.79 (d, J = 12.1 Hz, 1H, Ph-**CH**), 4.66 – 4.61 (m, 3H), 4.16 (d, J = 10.4 Hz, 1H), 4.08 (t, J = 9.2 Hz, 1H), 3.57 (dd, J = 10.5, 8.7 Hz, 1H), 3.50 (dd, J = 9.7, 3.5 Hz, 1H), 3.38 (s, 3H, OCH_3). ^{13}C NMR (126 MHz, $CDCl_3$) δ (ppm): 197.5 (1C, CHO), 138.4, 137.9, 137.5, 128.6, 128.5, 128.5, 128.2, 128.2, 128.1, 128.0, 127.8, 127.7 (15C, aromatic), 98.4 (1C, C-1), 81.8, 79.3, 77.8 (3C, C-2 – C-5), 76.0 (1C, Ph-**C**H_2), 75.1 (1C, Ph-**C**H_2), 74.2 (1C, C-2 – C-5), 73.7 (1C, Ph-**C**H_2), 55.8 (1C, OCH_3). MALDI-ToF MS: m\z calcd for $C_{28}H_{30}NaO_6^+$ $[M+Na]^+$ 485.204, found 485.230.

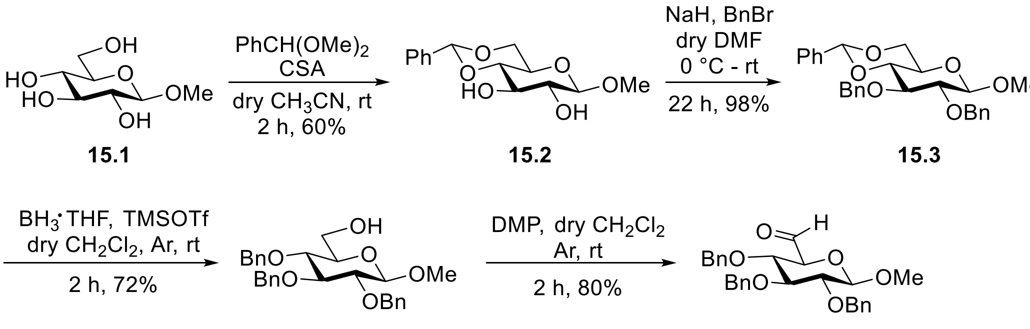

15.1 **15.2** **15.3** **15.4** **15**

Scheme S4. Synthesis of β-D-glucoside-derived 6-aldehyde using benzyl protecting groups. (DMP: Dess-Martin periodinane)

Methyl 4,6-*O*-benzylidene-β-D-glucopyranoside (15.2): To a suspension of methyl β-D-glucopyranoside **15.1** (10 g, 52 mmol, 1 equiv.) in dry acetonitrile (165 mL), camphorsulfonic acid (CSA, 3.6 g, 16 mmol, 0.3 equiv.) and benzaldehyde dimethyl acetal (11.6 mL, 78 mmol, 1.5 equiv.) were added, stirred at room temperature for 3.5 h, monitored by TLC (CH_2Cl_2: acetone 8:2). The reaction mixture was quenched with Et_3N (7 mL), filtered and evaporated, and it was purified by crystallization using hexane, filtered and washed with this system again to afford methyl 4,6-*O*-benzylidene-β-D-glucopyranoside as white crystals (8.7 g, 60%). R_f = 0.45 (CH_2Cl_2: MeOH 9:1). $[\alpha]_D$ -67.2 (c 0.18, $CHCl_3$), lit. $[\alpha]_D$ -61.5 (c 0.2, $CHCl_3$) [7]. The ^{1}H and ^{13}C NMR data of the compound are consistent with those reported in the literature [7]. MALDI-ToF MS: m\z calcd for $C_{14}H_{18}NaO_6^+$ $[M+Na]^+$ 305.110, found 305.112.

Methyl 2,3-di-*O*-benzyl-4,6-*O*-benzylidene-β-D-glucopyranoside (15.3): To a stirred solution of methyl 4,6-*O*-benzylidene-β-D-glucopyranoside **15.2** (5 g, 18 mmol, 1 equiv.) in dry DMF (127 mL) at 0 °C, sodium hydride (60%, 1.77 g, 44 mmol, 2.4 equiv., 1.2 equiv./OH) was added in small portions. The reaction mixture was stirred at 0°C for 30 min, after that BnBr (5.3 mL, 44 mmol, 2.4 equiv.) was added, followed by continuous stirring for 22 h at room temperature. The progress of the reaction was monitored by TLC (hexane: EtOAc 2:1). After complete conversion, the reaction mixture was diluted with MeOH (50 mL), stirred for 30 min, and then evaporated. The residue was dissolved in CH_2Cl_2 (500 mL) and washed with water (3×100 mL) until neutral pH. The organic layer was dried over $MgSO_4$, filtered, concentrated under vacuum, and crystallized with MeOH to afford the title compound as white crystals (8.0 g, 98%), which were used in the next step without further purification. R_f =0.18 (hexane: EtOAc 8:2). $[\alpha]_D$ -19.6 (c 0.24, MeOH). M.p. 119–121°C. ^{1}H NMR (400 MHz, $CDCl_3$) δ (ppm): 7.52 – 7.46 (m, 5H, aromatic), 7.41 – 7.32 (m, 7H, aromatic), 7.31 – 7.23 (m, 3H, aromatic), 5.57 (s, 1H, H-7), 4.91 (d, J = 11.4 Hz, 1H, Ph-**CH**), 4.87 (d, J = 11.0 Hz, 1H, Ph-**CH**), 4.80 (d, J = 11.4 Hz, 1H, Ph-**CH**), 4.76 (d, J = 11.0 Hz, 1H, Ph-**CH**), 4.42 (d, J = 7.6 Hz, 1H, H-1), 4.36 (dd, J = 10.5, 5.0 Hz, 1H), 3.79 (t, J = 10.3 Hz, 1H, overlap with triplet at 3.75 ppm), 3.75 (t, J = 9.1 Hz, 1H, overlap with triplet at 3.79 ppm), 3.68 (t, J = 9.2 Hz, 1H), 3.58 (s, 3H, OCH_3), 3.48 – 3.43 (m, 1H), 3.45 – 3.36 (m, 1H). ^{13}C NMR (101 MHz, $CDCl_3$) δ (ppm): 138.6, 138.5, 137.4 (3C, aromatic), 129.1, 128.4, 128.4, 128.3, 128.2, 128.1, 127.8, 127.7, 126.1 (15C, aromatic), 105.3 (1C, C-7), 101.2 (1C, C-1), 82.3, 81.6, 80.9, 75.4 (4C, sugar), 75.2 (2C, Ph-**CH_2**), 68.9 (1C, C-6), 66.1, 57.6 (2C, OCH_3, C_{sugar}). MALDI-ToF MS: m\z calcd for $C_{28}H_{30}NaO_6^+$ $[M+Na]^+$ 485.204, found 485.221. These characterization data are consistent with those reported in the literature [8].

Methyl 2,3,4-tri-*O*-benzyl-β-D-glucopyranoside (15.4): To a solution of methyl 2,3-di-*O*-benzyl-4,6-*O*-benzylidene-β-D-glucopyranoside **15.3** (1.7 g, 3.7 mmol, 1 equiv.) in dry CH_2Cl_2 (40 mL), BH_3·THF complex (1M in THF, 18.5 mL, 18.5 mmol, 5 equiv.) and TMSOTf (101 µL, 0.55 mmol, 0.15 equiv.) were added. The reaction mixture was stirred at room temperature under argon atmosphere. The reaction mixture was monitored by TLC (hexane: EtOAc 1:1). After complete conversion (2 h), Et_3N (7.5 mL) was added, followed by the careful addition of MeOH until the H_2 gas evolution ceased, and the residue was co-evaporated with MeOH (3×20 mL). The crude product was purified by column chromatography on silica gel (hexane: EtOAc 3:2) to afford the title compound as white crystals (1.2 g, 72%). R_f = 0.39 (hexane: EtOAc 1:1). ^{1}H NMR (500 MHz, $CDCl_3$) δ (ppm): 7.39 – 7.19 (m, 15H, aromatic), 4.93 (d, J = 11.1 Hz, 1H, Ph-**CH**), 4.90 (d, J = 11.2 Hz, 1H, Ph-**CH**), 4.86 (d, J = 10.9 Hz, 1H, Ph-**CH**), 4.81 (d, J = 11.0 Hz, 1H, Ph-**CH**), 4.71 (d, J = 11.0 Hz, 1H, Ph-**CH**), 4.64 (d, J = 11.0 Hz, 1H, Ph-**CH**), 4.35 (d, J = 7.8 Hz, 1H, H-1), 3.87 (d, J = 11.9 Hz, 1H, H-6a), 3.72 (dd, J = 11.5, 5.7 Hz, 1H, H-6b), 3.67 (t, J = 9.1 Hz, 1H, H-3), 3.58 (d, J = 9.5 Hz, 1H, H-4), 3.56 (s, 3H, OCH_3), 3.40 (t, J = 8.6 Hz, 1H, H-2), 3.36 (ddd, J = 9.7,

4.5, 2.8 Hz, 1H, H-5). ^{13}C NMR (126 MHz, $CDCl_3$) δ (ppm): 138.5, 138.4, 138.0, 128.5, 128.4, 128.1, 127.9, 127.9, 127.7, 127.6 (18C, aromatic), 104.8 (C-1), 84.4, 82.4, 77.6 (3C, sugar), 75.7, 75.1 (2C, 2 x Ph-**CH_2**), 75.0 (1C, sugar), 74.8 (1C, Ph-**CH_2**), 62.0 (1C, C-6), 57.3 (1C, OCH_3) MALDI-ToF MS: m\z calcd for $C_{28}H_{32}NaO_6^+$ $[M+Na]^+$ 487.219, found 487.284. These characterization data are consistent with those reported in the literature [9].

Methyl 2,3,4-tri-*O*-benzyl-β-D-*gluco*-hexodialdo-1,5-pyranoside (15): Methyl 2,3,4-tri-*O*-benzyl-β-D-glucopyranoside **15.4** (500 mg, 1.07 mmol, 1 equiv.) was dissolved in dry CH_2Cl_2 (10 mL), and Dess-Martin periodinane (DMP, 687 mg, 1.61 mmol, 1.5 equiv.) was added, followed by stirring at room temperature for 2 h. The reaction mixture was monitored by TLC (hexane: acetone 3:1). The reaction mixture was diluted with CH_2Cl_2, and NaOH solution (1.25M, 13 mL) was added with continuous stirring for 10 min. The organic layer was extracted with water to neutral pH. The organic phase was dried over $MgSO_4$, filtered, and concentrated under vacuum, then co-evaporated with toluene. The crude product, as a colorless syrup (400 mg, ~80%), was used in the cyclization reactions without further purification. R_f = 0.35 (CH_2Cl_2: acetone 95:5) **[α]**$_D$ -4.41 (c 0.204, $CHCl_3$). ^{1}H NMR (500 MHz, $CDCl_3$) δ (ppm): 9.62 (d, J = 1.4 Hz, 1H, CHO), 7.34 – 7.24 (m, 15H, aromatic), 4.90 (d, J = 7.8 Hz, 1H, Ph-**CH**), 4.88 (d, J = 8.1 Hz, 1H, Ph-**CH**), 4.78 (d, J = 10.9 Hz, 2H, Ph-**CH**), 4.69 (d, J = 11.0 Hz, 1H, Ph-**CH**), 4.61 (d, J = 10.7 Hz, 1H, Ph-**CH**), 4.42 (d, J = 7.3 Hz, 1H, H-1), 3.85 (dd, J = 9.1, 1.2 Hz, 1H), 3.76 – 3.66 (m, 2H), 3.57 (s, 3H, OCH_3), 3.44 (dd, J = 8.4, 7.3 Hz, 1H). ^{13}C NMR (126 MHz, $CDCl_3$) δ (ppm): 138.3, 137.4 (3C, aromatic), 128.5, 128.5, 128.4, 128.3, 128.1, 127.9, 127.8 (15C, aromatic), 104.6 (1C, C-1), 83.7, 81.7, 78.3, 77.2 (4C, C-2 – C-5), 75.6, 74.9, 74.7 (3C, 3 x Ph-**C**H_2), 57.4 (1C, OCH_3). MALDI-ToF MS: m\z calcd for $C_{28}H_{30}$ NaO_6^+ $[M+Na]^+$ 487.204, found 485.317.

General method for the oxa-Pictet–Spengler cyclization reaction with silyl protecting groups (Method A):

In a flame-dried round-bottom flask, the corresponding alcohol and the silylated sugar 6-aldehyde were dissolved in dry CH_2Cl_2. The reaction mixture was cooled down to 0 °C. Under argon atmosphere, the appropriate amount of $BF_3 \cdot Et_2O$ was added slowly, followed by continuous stirring at room temperature. The reaction mixture was monitored by TLC till complete conversion or no further changes were observed. The reaction was quenched with saturated aqueous $NaHCO_3$ solution and stirred for a further 5 min at room temperature. The solvent was evaporated under vacuum, and the crude product was purified by flash column chromatography.

General method for the oxa-Pictet–Spengler cyclization reaction with benzyl protecting groups (Method B):

In a flame-dried round-bottom flask, the corresponding alcohol and the benzylated sugar 6-aldehyde or glucopyranosyl formaldehyde were dissolved in dry CH_2Cl_2. The reaction mixture was cooled down to 0 °C. Under argon atmosphere, the appropriate amount of $BF_3 \cdot Et_2O$ was added slowly. The reaction mixture was monitored by TLC till complete conversion or no further changes were observed. The reaction was quenched with saturated aqueous $NaHCO_3$ solution and stirred for a further 5 min at room temperature. Next, the organic layer was washed with distilled water, dried over $MgSO_4$, and the solution was concentrated under vacuum. The crude product was purified by flash column chromatography to afford the cyclized product.

General method for debenzylation (Method C):

The corresponding compound was dissolved in MeOH (~2 mL/mmol) under argon atmosphere. Pd/C (catalytic amount of 10% on activated charcoal) and a few drops of acetic acid were added. The reaction mixture was stirred overnight under H_2 atmosphere (1 atm), then filtered through a Celite pad, and the solvent was evaporated in vacuum.

Synthesis of isochroman-sugar hybrids using hexodialdo-1,5-pyranosides

1. (1*S*,3*S*)-1-(Methyl α-D-xylopyranoside-5-yl)-7,8-dimethoxy-3-methylisochroman (3)

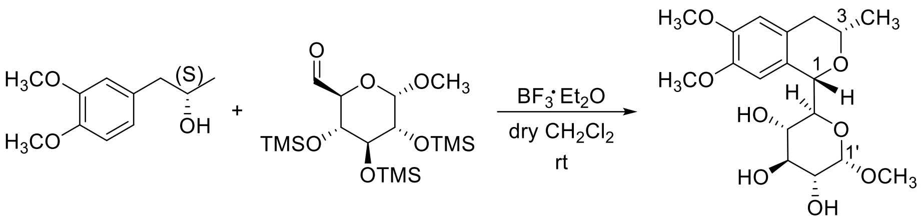

(*S*)-1 2 3

I. Alcohol (*S*)-**1** (100 mg, 0.5 mmol, 1 equiv.), dialdose **2** (204 mg, 0.5 mmol, 1 equiv), and $BF_3 \cdot Et_2O$ (13 μL, 0.1 mmol, 0.2 equiv.) in dry CH_2Cl_2 (10 mL) were reacted according to the general method A. Reaction time: 24 h. The reaction mixture was quenched with saturated $NaHCO_3$ solution (1.5 mL) and stirred for a further 5 min at room temperature. The crude product was purified twice by flash column chromatography on silica gel (CH_2Cl_2: acetone 6:4 and then CH_2Cl_2: MeOH 95:5) to afford compound **3** (58.1 mg, 32 %) as white crystals.

II. Alcohol (*S*)-**1** (100 mg, 0.5 mmol, 1 equiv.), dialdose **2** (204 mg, 0.5 mmol, 1 equiv) and $BF_3 \cdot Et_2O$ (130 μL, 1 mmol, 2 equiv.) in dry CH_2Cl_2 (10 mL) were reacted for 12 h at rt. The reaction mixture was quenched with Et_3N, concentrated, and purified by flash column chromatography (CH_2Cl_2: MeOH 95:5) to give compound **3** as white crystals (104 mg, 57%).

R_f = 0.40 (CH_2Cl_2: MeOH 9:1). $[\alpha]_D$ +154.0 (c 0.2, DMSO). M.p: 245-248 °C. ^{1}H NMR (500 MHz, CD_3SOCD_3) δ (ppm): 6.71 (s, 1H, H-9), 6.65 (s, 1H, H-6), 5.68 (s, 1H, OH), 5.01 (s, 1H, H-1), 4.42 (d, J = 3.7 Hz, 1H, H-1'), 4.11 (s, 2H, 2 x OH), 3.93 (d, J = 9.7 Hz, 1H, H-5'), 3.73 (s, 6H, 2 x OCH_3), 3.71-3.67 (m, 1H, H-3), 3.62-3.59 (m, 1H, H-4'), 3.51 (t, J = 9.2 Hz, 1H, H-3'), 3.23 (dd, J = 9.6, 3.7 Hz, 1H, H-2'), 3.11 (s, 3H, OCH_3, anomeric), 2.53 – 2.50 (m, 2H, H-4a,b), 1.27 (d, J = 6.1 Hz, 3H, CH_3). ^{13}C NMR (125 MHz, CD_3SOCD_3) δ (ppm): 147.6, 147.3 (2C, C-7, C-8), 127.7 (2C, C-5, C-10), 112.2 (1C, C-6), 107.7 (1C, C-9), 100.3 (1C, C-1'), 74.0 (1C, C-3'), 73.6 (1C, C-1), 72.7 (1C, C-5'), 72.0 (1C, C-2'), 70.5 (1C, C-3), 69.5 (1C, C-4'), 55.7, 55.6 (2C, 2 x OCH_3), 55.0 (1C, OCH_3, anomeric), 35.9 (1C, C-4), 21.7 (1C, CH_3). MALDI-ToF HRMS: m\z calcd for $C_{18}H_{26}NaO_8^+$ $[M+Na]^+$ 393.1520, found 393.1515.

2. (1*R*,3*R*)-1-(Methyl α-D-xylopyranoside-5-yl)-7,8-dimethoxy-3-methylisochroman (4) and **(1*S*,3*R*)-1-(methyl α-D-xylopyranoside-5-yl)-7,8-dimethoxy-3-methylisochroman (5)**

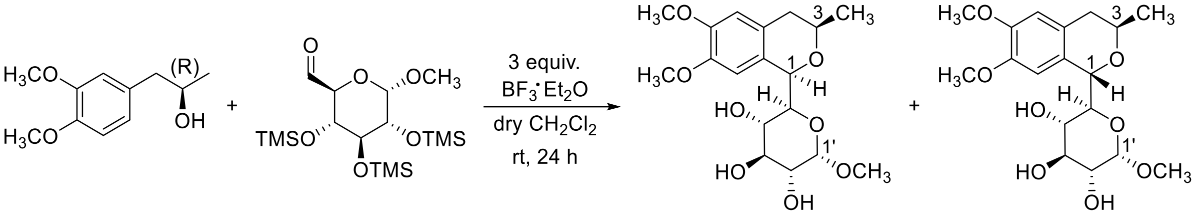

(*R*)-1 **2** **4** **5**

Alcohol (*R)*-**1** (100 mg, 0.5 mmol, 1 equiv.), dialdose **2** (210 mg, 0.5 mmol, 1 equiv.), dry CH_2Cl_2 (10 mL) and $BF_3 \cdot Et_2O$ (190 μL, 1.5 mmol, 3 equiv.) were used according to method A. Reaction time: 24 h. The reaction mixture was quenched with saturated $NaHCO_3$ solution (3.5 mL) and stirred for a further 5 min at room temperature. The crude product was purified by flash column chromatography twice on silica gel (CH_2Cl_2: acetone 7:3 and then CH_2Cl_2: MeOH 95:5) to afford compound **4** (48 mg, 27%) as a white solid and compound **5** (39 mg, 22%) as a white solid.
The reaction was also carried out using 0.2 equiv of $BF_3 \cdot Et_2O$. After 7 h, compound **4** was isolated as the sole product with 23% yield.
Compound **4**: R_f = 0.40 (CH_2Cl_2: MeOH 9:1). $[\alpha]_D$ +16.7 (c 0.21, DMSO). M.p: 106-108 °C. ^1H NMR (500 MHz, CD_3SOCD_3) δ (ppm): 6.74 (s, 1H, H-9), 6.66 (s, 1H, H-6), 5.00 (s, 1H, H-1), 4.90 (d, *J* = 5.0 Hz, 1H, H-4'-OH), 4.78 (d, *J* = 4.6 Hz, 1H, H-3'-OH), 4.65 (d, *J* = 6.5 Hz, 1H, H-2'-OH), 4.45 (d, *J* = 3.7 Hz, 1H, H-1'), 3.72 (s, 3H, OCH_3), 3.71 (s, 3H, OCH_3), 3.69 (dd, *J* = 10.4, 1.9 Hz, 1H, H-5'), 3.59 (ddd, *J* = 9.3, 6.1, 2.9 Hz, 1H, H-3), 3.40-3.38 (m, 1H, H-3'), 3.35 (s, 3H, OCH_3), 3.33-3.31 (m, 1H, H-4'), 3.07 (ddd, *J* = 9.6, 6.5, 3.8 Hz, 1H, H-2'), 2.53 (dd, *J* = 14.9, 10.8 Hz, 1H, H-4a), 2.47 (dd, *J* = 15.3, 2.4 Hz, 1H, H-4b), 1.22 (d, *J* = 5.8 Hz, 3H, CH_3). ^{13}C NMR (125 MHz, CD_3SOCD_3) δ (ppm): 147.3 (1C, C-8), 146.7 (1C, C-7), 128.3 (1C, C-10), 125.8 (1C, C-5), 111.6 (1C, C-6), 110.0 (1C, C-9), 99.6 (1C, C-1'), 74.9 (1C, C-1), 74.4 (1C, C-5'), 73.9 (1C, C-3'), 72.0 (1C, C-2'), 71.0 (1C, C-4'), 69.5 (1C, C-3), 55.8, 55.4 (2C, 2 x OCH_3), 54.1 (1C, OCH_3, anomeric), 36.2 (1C, C-4), 21.9 (1C, CH_3). MALDI-ToF HRMS: m\z calcd for $C_{18}H_{26}NaO_8^+$ $[M+Na]^+$ 393.1520, found 393.1523.
Compound **5**: R_f = 0.34 (CH_2Cl_2: MeOH 9:1). $[\alpha]_D$ +65.0 (c 0.12, DMSO). M.p: 199-201 °C. ^1H NMR (500 MHz, CD_3SOCD_3) δ (ppm): 6.70 (s, 1H, H-9), 6.65 (s, 1H, H-6), 5.00 (d, *J* = 5.5 Hz, 1H, H-4'-OH), 4.95 (s, 1H, H-1), 4.77 (d, *J* = 4.8 Hz, 1H, H-3'-OH), 4.67 (d, *J* = 6.4 Hz, 1H, H-2'-OH), 4.37 (d, *J* = 3.6 Hz, 1H, H-1'), 4.27 (ddd, *J* = 9.6, 6.2, 3.2 Hz, 1H, H-3), 3.80 (dd, *J* = 9.5, 1.9 Hz, 1H, H-5'), 3.71 (s, 3H, OCH_3), 3.70 (s, 3H, OCH_3), 3.52 (td, *J* = 9.1, 5.7 Hz, 1H, H-4'), 3.46 (td, *J* = 9.0, 4.9 Hz, 1H, H-3'), 3.17 (ddd, *J* = 9.7, 6.4, 3.7 Hz, 1H, H-2'), 2.94 (s, 3H, OCH_3, anomeric), 2.61 (dd, *J* = 15.8, 2.8 Hz, 1H, H-4a), 2.37 (dd, *J* = 15.7, 10.2 Hz, 1H, H-4b), 1.18 (d, *J* = 6.2 Hz, 3H, CH_3). ^{13}C NMR (101 MHz, CD_3SOCD_3) δ (ppm): 147.0, 146.8 (2C, C-7, C-8), 126.4 (2C, C-5, C-10), 111.8 (1C, C-6), 107.7 (1C, C-9), 99.5 (1C, C-1'), 75.5 (1C, C-5'), 73.5 (1C, C-3'), 71.8 (1C, C-2'), 70.4 (1C, C-1), 69.8 (1C, C-4'), 66.2 (1C, C-3), 55.4, 55.3 (2C, 2 x OCH_3, aromatic), 54.0 (1C, OCH_3, anomeric), 35.1 (1C, C-4), 21.3 (1C, CH_3). MALDI-ToF HRMS: m\z calcd for $C_{18}H_{26}NaO_8^+$ $[M+Na]^+$ 393.1520, found 393.1522.

3. **(1*S*,3*S*)-1-(Methyl α-D-lyxopyranoside-5-yl)-7,8-dimethoxy-3-methylisochroman (7)** and **(1*S*,3*R*)-1-(Methyl α,β-D-lyxopyranoside-5-yl)-7,8-dimethoxy-3-methylisochroman (8)**

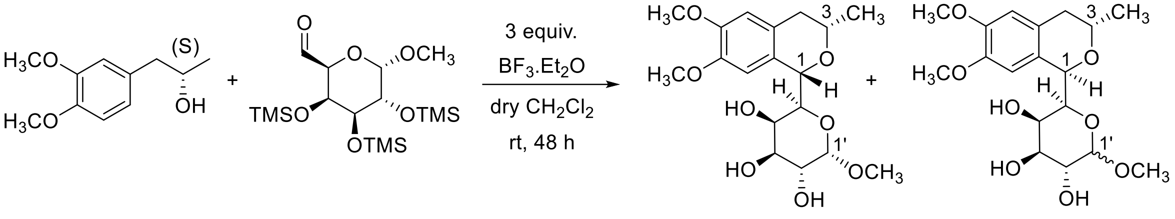

(*S*)-1 **6** **7** (16%) **8** (17%, α:β ~3:1)

Alcohol (*S*)-**1** (100 mg, 0.5 mmol, 1 equiv.), dialdose **6** (306 mg, 0.74 mmol, 1.5 equiv.), dry CH_2Cl_2 (10 mL) and $BF_3·Et_2O$ (185 µL, 1.5 mmol, 3 equiv.) were used according to method A. Reaction time: 48 h. The reaction mixture was quenched with saturated $NaHCO_3$ solution (3 mL) and stirred for a further 5 min at room temperature. The crude product was purified twice by flash column chromatography on silica gel (CH_2Cl_2: acetone 6:4, and then CH_2Cl_2: MeOH 97:3) to afford compound **7** (30 mg, 16 %) as a white solid and compound **8** (31 mg, 17%) as a white foam.

Compound **7**: R_f = 0.34 (CH_2Cl_2: MeOH 9:1). $[\alpha]_D$ +133.6 (c 0.147, $CHCl_3$). M.p.: 139-143°C. ^1H NMR (400 MHz, $CDCl_3$) δ (ppm): 6.63 (s, 1H, H-9), 6.60 (s, 1H, H-6), 5.19 (s, 1H, H-1), 4.77 (d, *J* = 4.0 Hz, 1H, H-1'), 4.34 (d, *J* = 3.1 Hz, 1H, H-4'), 4.23 (s, 1H, H-5'), (3.97 dd, *J* = 9.7, 4.0 Hz, 1H, H-2'), 3.87 (s, 4H, OCH_3 (aromatic), H-3), 3.86 (s, 3H, OCH_3, aromatic), 3.77 (dd, *J* = 9.7, 3.2 Hz, 1H, H-3'), 3.22 (s, 3H, OCH_3, anomeric), 2.83 (dd, *J* = 15.8, 11.0 Hz, 1H, H-4a), 2.56 (dd, *J* = 16.0, 2.7 Hz, 1H, H-4b), 1.41 (d, J = 6.1 Hz, 3H, CH_3). ^{13}C NMR (101 MHz, $CDCl_3$) δ (ppm): 148.1, 147.6 (2C, C-7, C-8), 127.2, 125.3 (2C, C-5, C-10), 111.7 (1C, C-6), 106.8 (1C, C-9), 100.2 (1C, C-1'), 79.7 (1C, C-1), 72.5 (1C, C-4'), 72.0 (1C, C-3'), 71.7 (1C, C-3), 69.7 (1C, C-2'), 69.7 (1C, C-5'), 56.1, 56.0, 55.8 (3C, 3 x OCH_3), 35.6 (1C, C-4), 21.5 (1C, CH_3). MALDI-ToF HRMS: m\z calcd for $C_{18}H_{26}NaO_8^+$ $[M+Na]^+$ 393.1520, found 393.1522.

Compound **8α:** R_f = 0.34 (CH_2Cl_2: MeOH 9:1). (NMR assignment was made from the spectrum of the α:β 3:1 mixture): ^1H NMR (700 MHz, CD_3SOCD_3) δ (ppm): 6.94 (s, 1H, H-6/9), 6.65 (s, 1H, H-6/9), 4.68 (d, *J* = 9.6 Hz, 1H), 4.62 (d, *J* = 3.9 Hz, 1H, H-1'), 4.57 – 4.48 (m, 3H, 3 x OH), 3.95 (t, *J* = 3.9 Hz, 1H), 3.86 (ddd, *J* = 9.9, 5.8, 3.4 Hz, 1H, H-3), 3.71 (s, 3H, OCH_3), 3.70 – 3.66 (m, 5H, H-2', OCH_3), 3.48 (d, *J* = 10.1 Hz, 1H), 3.36 – 3.34 (m, 1H, overlap with CD_3SOCD_3 signal), 2.89 (s, 3H, OCH_3), 2.65 (dd, *J* = 16.3, 3.2 Hz, 1H, H-4a), 2.45 (dd, *J* = 16.1, 10.6 Hz, 1H, H-4b), 1.22 – 1.17 (m, 3H, CH_3). ^{13}C NMR (176 MHz, CD_3SOCD_3) δ (ppm): 147.4, 146.1, 127.5, 125.2 (4C, C-5, C-7, C-8, C-10), 111.3, 111.2 (2C, C-6, C-9), 100.5 (1C, C-1'), 70.9, 70.5, 70.0, 68.4, 67.9, 65.1 (6C, C-1, C-3, C-2' – C-5'), 55.4, 55.3, 54.4 (3C, OCH_3), 35.11 (1C, C-4), 21.7 (1C, CH_3). MALDI-ToF HRMS: m\z calcd for $C_{18}H_{26}NaO_8^+$ $[M+Na]^+$ 393.1520, found 393.1514.

4. **(1*R*,3*R*)-1-(Methyl α,β-D-lyxopyranoside-5-yl)-7,8-dimethoxy-3-methylisochroman (9α,β)** and **(1*S*,3*R*)-1-(methyl α,β-D-lyxopyranoside-5-yl)-7,8-dimethoxy-3-methylisochroman (10α,β)**

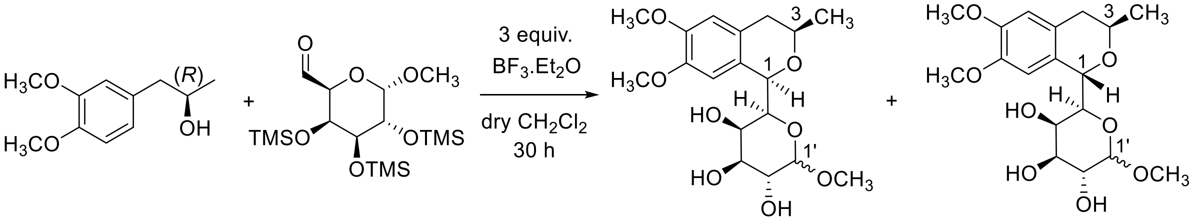

(*R*)-1 **6** **9**α,β (22%, α:β ~2:1) **10**α,β (10%, α:β ~3:1)

Alcohol (*R*)-**1** (100 mg, 0.5 mmol, 1 equiv.), dialdose **6** (310 mg, 0.75 mmol, 1.5 equiv.), dry CH_2Cl_2 (10 mL) and $BF_3·Et_2O$ (190 µL, 1.5 mmol, 3 equiv.) were used according to method A. Reaction time: 30 h. The reaction mixture was quenched with saturated $NaHCO_3$ solution (3 mL) and stirred for a further 5 min at room temperature. The crude product was purified twice by flash column chromatography on silica gel (CH_2Cl_2: acetone 6:4, and then CH_2Cl_2: MeOH 97:3) to afford **9α,β** (41 mg, 22 %) and **10α,β** (18 mg, 10%).
Compound **9α,β** (α:β 2:1 mixture): R_f = 0.35 (CH_2Cl_2: MeOH 97:3). To separate the anomers, the anomeric mixture was acetylated.
Compound **10α** (α:β 3:1 mixture): R_f = 0.35 (CH_2Cl_2: MeOH 97:3). (NMR assignment was made from the spectrum of the α:β 3:1 mixture): ^{1}H NMR (700 MHz, $CDCl_3$) δ (ppm): 6.69 (s, 1H, H-6/9), 6.60 (s, 1H, H-6/9), 5.14 – 5.09 (m, 1H), 4.75 (d, *J* = 3.9 Hz, 1H), 4.57 (s, 1H), 4.47 (ddd, *J* = 9.8, 6.3, 3.5 Hz, 1H), 4.30 (d, *J* = 3.4 Hz, 1H), 4.11 (d, *J* = 3.4 Hz, 1H), 3.96 – 3.89 (m, 1H), 3.87 (s, 3H, OCH_3), 3.85 (s, 3H, OCH_3), 3.76 – 3.71 (m, 2H), 3.31 (s, 1H), 3.16 (s, 3H, OCH_3), 2.72 (dd, *J* = 15.9, 3.4 Hz, 1H, H-4a), 2.57 (dd, *J* = 15.8, 10.0 Hz, 1H, H-4b), 1.31 (d, *J* = 6.3 Hz, 3H, CH_3). ^{13}C NMR (176 MHz, $CDCl_3$) δ (ppm): 148.2, 147.6, 126.6, 124.8 (4C, C-5, C-7, C-8, C-10), 111.6, 107.7 (2C, C-6, C-9), 99.6 (1C, C-1'), 76.5, 71.9, 71.9, 71.5, 70.0, 67.76 (6C, C-1, C-3, C-2' – C-5'), 56.2, 56.0, 55.6 (3C, OCH_3), 35.2 (1C, C-4), 21.4 (1C, CH_3). MALDI-ToF HRMS: m\z calcd for $C_{18}H_{26}NaO_8^+$ $[M+Na]^+$ 393.1520, found 393.1525.

4.1. (1*R*,3*R*)-1-(Methyl 2,3,4-tri-*O*-acetyl-α-D-lyxopyranoside-5-yl)-7,8-dimethoxy-3-methylisochroman (11α) and (1*R*,3*R*)-1-(methyl 2,3,4-tri-*O*-acetyl-β-D-lyxopyranoside-5-yl)-7,8-dimethoxy-3-methylisochroman (11β)

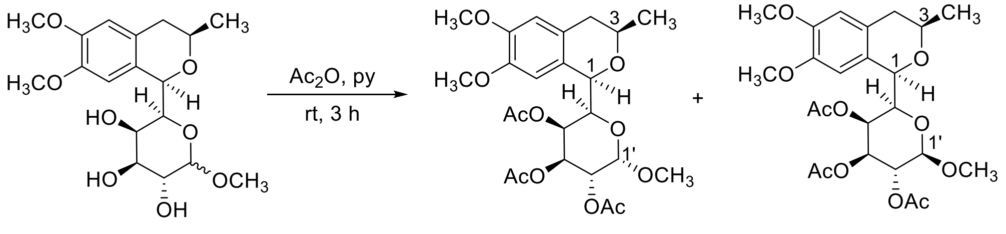

The anomeric mixture **9α,β** (29 mg, 0.08 mmol, 1 equiv.) was dissolved in dry pyridine (2 mL), and acetic anhydride (1 mL) was added at 0 °C. The reaction mixture was allowed to warm up to room temperature and stirred for 3 h. The reaction mixture was concentrated and co-evaporated with toluene. The crude product was purified by column chromatography on silica gel (hexane: acetone 8:1) to give **11α** (23 mg, 58%) as a white solid and **11β** (10 mg, 26%) as a white solid.
Compound **11α**: R_f = 0.30 (hexane: acetone 6:2). $[\alpha]_D$ +32.5 (c 0.12, $CHCl_3$). ^{1}H NMR (500 MHz, $CDCl_3$) δ (ppm): 7.16 (s, 1H, H-9), 6.57 (s, 1H, H-6), 5.82 (d, *J* = 2.4 Hz, 1H, H-4'), 5.36 (dd, *J* = 10.9, 3.3 Hz, 1H, H-3'), 5.20 (dd, *J* = 10.9, 3.5 Hz, 1H, H-2'), 5.10 (d, *J* = 3.4 Hz, 1H, H-1'), 4.73 (d, *J* = 8.7 Hz, 1H, H-1), 3.86 (s, 3H, OCH_3), 3.82 (s, 3H, OCH_3), 3.79 (d, *J* = 8.7 Hz, 1H, H-5', overlap with OCH_3), 3.62 (ddd, *J* = 9.5, 6.1, 3.0 Hz, 1H, H-3), 3.18 (s, 3H, OCH_3, anomeric), 2.58 (dd, *J* = 15.4, 10.4 Hz, 1H, H-4a), 2.52 (dd, *J* = 15.4, 2.2 Hz, 1H, H-4b), 2.17 (s, 3H, CH_3CO), 2.09 (s, 3H, CH_3CO), 2.00 (s, 3H, CH_3CO), 1.23 (d, *J* = 6.0 Hz, 3H, CH_3). ^{13}C NMR (126 MHz, $CDCl_3$) δ (ppm): 170.7, 170.1, 169.8 (3C, 3 x C=O), 147.9 (1C, C-8), 147.1 (1C, C-7), 127.3, 127.2 (2C, C-5, C-10), 111.2 (1C, C-6), 109.6 (1C, C-9), 97.5 (1C, C-1'), 72.8 (1C, C-1), 72.4 (1C, C-5'), 70.3 (1C, C-3), 68.7 (1C, C-2'), 68.7 (1C, C-4'), 67.9 (1C, C-3'), 55.9, 55.9, 55.8 (3C, 3 x OCH_3), 36.5 (1C, C-4), 21.5 (1C, CH_3), 21.0, 20.9, 20.8 (3C, 3 x CH_3CO). MALDI-ToF HRMS: m\z calcd for $C_{24}H_{32}NaO_{11}^+$ $[M^+Na]^+$ 519.1840, found 519.1849.
Compound **11β**: R_f = 0.30 (hexane: acetone 6:2). $[\alpha]_D$ -26.1 (c 0.088, $CHCl_3$). ^{1}H NMR (500 MHz, $CDCl_3$) δ (ppm): 7.07 (s, 1H, H-9), 6.58 (s, 1H, H-6), 5.78 (dd, *J* = 3.5, 1.2 Hz, 1H, H-4'), 5.27 (dd, *J* = 10.5, 8.0 Hz, 1H, H-2'), 5.01 (dd, *J* = 10.5, 3.5 Hz, 1H, H-3'), 4.76 (d, *J* = 8.3 Hz, 1H, H-1),

4.37 (d, $J = 8.0$ Hz, 1H, H-1'), 3.86 (s, 3H, OCH_3), 3.83 (s, 3H, OCH_3), 3.64 (ddd, $J = 9.2, 6.1, 4.2$ Hz, 1H, H-3), 3.58 (s, 3H, OCH_3, anomeric), 3.57 (dd, $J = 8.5, 1.4$ Hz, 1H, H-5', overlap with OCH_3), 2.59 – 2.53 (m, 2H, H-4a,b), 2.17 (s, 3H, CH_3CO), 2.06 (s, 3H, CH_3CO), 2.00 (s, 3H, CH_3CO), 1.23 (d, $J = 6.1$ Hz, 3H, CH_3). ^{13}C NMR (126 MHz, $CDCl_3$) δ(ppm): 170.4, 170.0, 169.7 (3C, 3 x C=O), 148.2, 147.4 (2C, C-7, C-8), 127.5, 127.3 (2C, C-5, C-10), 111.3 (1C, C-6), 109.3 (1C, C-9), 102.6 (1C, C-1'), 77.5 (1C, C-5'), 73.1 (1C, C-1), 71.3 (1C, C-3'), 70.6 (1C, C-3), 69.2 (1C, C-2'), 67.7 (1C, C-4'), 57.5 (1C, OCH_3, anomeric), 56.2, 55.9 (2C, 2 x OCH_3), 36.5 (1C, C-4), 21.5 (1C, CH_3), 21.0, 20.9, 20.8 (3C, 3 x CH_3CO). MALDI-ToF HRMS: m\z calcd for $C_{24}H_{32}NaO_{11}^+$ $[M+Na]^+$ 519.1840, found 519.1848.

4.2. (1*R*,3*R*)-1-(Methyl α-D-lyxopyranoside-5-yl)-7,8-dimethoxy-3-methylisochroman (9α)

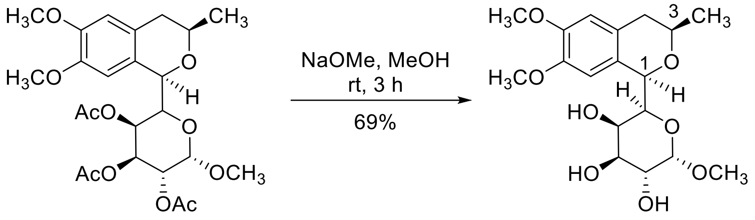

11α **9**α

To a stirred solution of **11α** (22 mg, 0.04 mmol) in dry MeOH (2 mL), a catalytic amount of NaOMe was added (pH = 9-10). The reaction mixture was stirred at room temperature for 3 h. Then, the mixture was neutralized with Amberlite IR-120 H^+ ion exchange resin, filtered, and concentrated under reduced pressure to give **9α** (13 mg, 69%) as a white solid. $R_f = 0.50$ (CH_2Cl_2: methanol 9:1). $[\alpha]_D$ +26.6 (c 0.101, DMSO). M.p: 160-162 °C. ^1H NMR (500 MHz, CD_3SOCD_3) δ (ppm): 7.19 (s, 1H, H-9), 6.67 (s, 1H, H-6), 4.78 (d, $J = 8.7$ Hz, 1H, H-1), 4.66 (d, $J = 3.8$ Hz, 1H, H-1'), 4.58 (s, 1H, OH), 4.43 (s, 1H, OH), 4.01 (d, $J = 3.1$ Hz, 1H, H-4'), 3.72 (s, 3H, OCH_3), 3.72 – 3.70 (m, 1H, H-2', overlap with OCH_3), 3.69 (s, 3H, OCH_3), 3.62 (ddd, $J = 10.7, 6.1, 2.6$ Hz, 1H, H-3), 3.46 (dd, $J = 10.0, 3.2$ Hz, 1H, H-3'), 3.37 – 3.34 (m, 1H, H-5', overlap with CD_3SOCD_3 signal), 3.03 (s, 3H, OCH_3, anomeric), 2.59 (dd, $J = 15.5, 1.9$ Hz, 1H, H-4a), 2.50 – 2.45 (m, 1H, H-4b, overlap with CD_3SOCD_3 signal), 1.24 (d, $J = 5.9$ Hz, 3H, CH_3). ^{13}C NMR (126 MHz, CD_3SOCD_3) δ (ppm): 147.2, 146.4 (2C, C-7, C-8), 127.8, 126.7 (2C, C-5, C-10), 111.4 (1C, C-6), 110.5 (1C, C-9), 100.5 (1C, C-1'), 73.3 (1C, C-5'), 72.4 (1C, C-1), 69.8 (1C, C-3'), 69.4 (1C, C-3), 68.2, 68.2 (2C, C-2', C-4'), 55.4, 55.3 (2C, 2 x OCH_3), 54.8 (1C, OCH_3, anomeric), 36.0 (1C, C-4), 21.5 (1C, CH_3). MALDI-ToF HRMS: m\z calcd for $C_{18}H_{26}NaO_8^+$ $[M+Na]^+$ 393.1520, found 393.1526.

5. (1*S*,3*S*)-1-(Methyl 2,3,4-tri-*O*-benzyl-*α*-D-xylopyranoside-5-yl)-7,8-dimethoxy-3-methylisochroman (16)

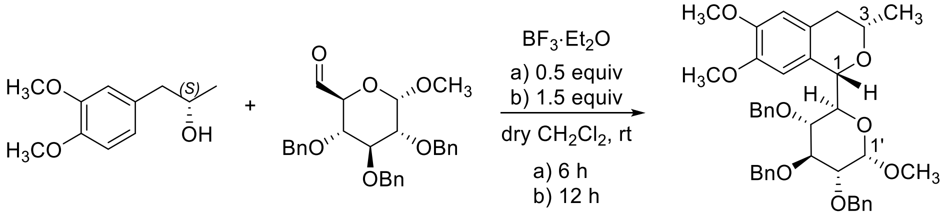

(*S*)-**1** **14** **16**

a) Alcohol (*S*)-**1** (50 mg, 0.256 mmol, 1 equiv.), dialdose **14** (143 mg, 0.31 mmol, 1.2 equiv.), dry CH_2Cl_2 (5 mL) and $BF_3 \cdot Et_2O$ (16 µL, 0.13 mmol, 0.5 equiv.) were used according to method B. Reaction time: 6 h. The reaction mixture was quenched with saturated $NaHCO_3$ solution (1 mL) and stirred for 5 min at room temperature. The crude product was purified by flash column chromatography on silica gel (CH_2Cl_2: EtOAc 95:5) to afford compound **16** (100 mg, 61%) as a white solid.

b) The reaction was repeated on the same scale using 1.5 equiv of $BF_3·Et_2O$ to give compound **16** with 74% yield.

R_f = 0.38 (hexane: acetone 2:1). $[\alpha]_D$ +80.0 (c 0.12, $CHCl_3$). M.p: 141-147 °C. ^1H NMR (400 MHz, $CDCl_3$) δ (ppm): 7.46 – 7.39 (m, 2H, aromatic), 7.39 – 7.21 (m, 13H, aromatic), 6.66 (s, 1H, H-9), 6.57 (s, 1H, H-6), 5.09 (s, 1H, H-1), 5.03 (d, *J* = 10.9 Hz, 1H, Ph-**CH**), 4.94 (d, *J* = 10.8 Hz, 1H, Ph-**CH**), 4.91 (d, *J* = 11.1 Hz, 1H, Ph-**CH**), 4.79 (d, *J* = 12.3 Hz, 1H, Ph-**CH**), 4.72 (d, *J* = 10.6 Hz, 1H, Ph-**CH**), 4.64 (d, *J* = 12.3 Hz, 1H, Ph-**CH**), 4.52 (d, *J* = 3.6 Hz, 1H, H-1'), 4.20 (dd, *J* = 9.3, 1.4 Hz, 1H, H-5'), 4.04 (m, 2H, H-3', H-4'), 3.83 (s, 3H, OCH_3, aromatic), 3.81 (s, 3H, OCH_3, aromatic), 3.74 (ddd, *J* = 11.0, 6.1, 2.8 Hz, 1H, H-3), 3.58 (dd, *J* = 9.1, 3.6 Hz, 1H, H-2'), 3.24 (s, 3H, OCH_3, anomeric), 2.77 (dd, *J* = 15.4, 11.3 Hz, 1H, H-4a), 2.53 (dd, *J* = 15.9, 1.6 Hz, 1H, H-4b), 1.34 (d, *J* = 6.1 Hz, 3H, CH_3). ^{13}C NMR (101 MHz, $CDCl_3$) δ (ppm): 147.5, 147.2 (2C, C-7, C-8), 138.8, 138.3, 138.2, 128.5, 128.4, 128.2, 128.0, 128.0, 127.9, 127.8, 127.6 (18C, aromatic), 127.4, 126.6 (2C, C-5, C-10), 111.6 (1C, C-6), 106.5 (1C, C-9), 98.5 (1C, C-1'), 82.3 (1C, C-3'), 79.6 (1C, C-2'), 77.6 (1C, C-4'), 75.8, 75.6 (2C, 2 x Ph-CH_2), 73.7 (1C, C-1), 73.4 (1C, Ph-**C**H_2), 71.4 (1C, C-5'), 71.0 (1C, C-3), 55.9, 55.8 (3C, 3 x OCH_3), 35.8 (1C, C-4), 21.6 (1C, CH_3). MALDI-ToF HRMS: m/z calculated for $C_{39}H_{44}NaO_8^+$ $[M+Na]^+$ 663.2928, found 663.2930.

6. (1*S*,3*S*)-1-(Methyl 2,3,4-tri-*O*-benzyl-α-D-xylopyranoside-5-yl)-7,8,9-trimethoxy-3-methylisochroman (17)

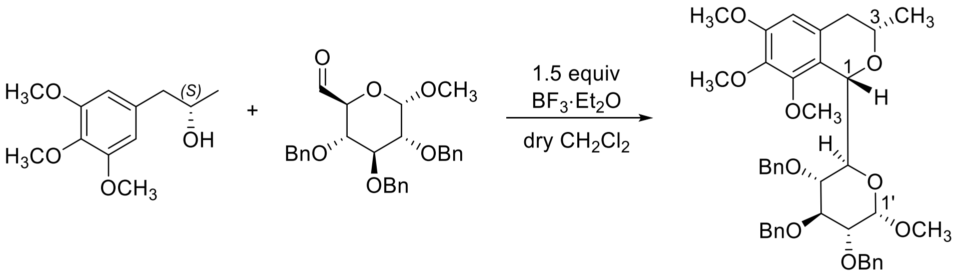

(*S*)-**12** **14** **17**

Alcohol (*S*)-**12** (50 mg, 0.22 mmol, 1 equiv.), dialdose **14** (122 mg, 0.26 mmol, 1.2 equiv.), dry CH_2Cl_2 (5 mL) and $BF_3·Et_2O$ (45 µL, 0.33 mmol, 1.5 equiv.) were used according to method B. Reaction time 4 h. The reaction mixture was quenched with saturated $NaHCO_3$ solution (1.5 mL) and stirred for a further 5 min at room temperature. The crude product was purified by flash column chromatography on silica gel (hexane: EtOAc 5:1) to afford compound **17** (110 mg, 75%) as a colorless syrup. R_f = 0.44 (hexane: acetone 7:3). $[\alpha]_D$ +42.2 (c 0.09, $CHCl_3$). ^1H NMR (500 MHz, $CDCl_3$) δ (ppm): 7.40 – 7.36 (m, 4H, aromatic), 7.34 – 7.30 (m, 6H, aromatic), 7.29 – 7.21 (m, 5H, aromatic), 6.36 (s, 1H, H-6), 5.24 (s, 1H, H-1), 4.98 (d, *J* = 10.9 Hz, 1H, Ph-**CH**), 4.95 (d, *J* = 11.8 Hz, 1H, Ph-**CH**), 4.87 (d, *J* = 10.9 Hz, 1H, Ph-**CH**), 4.84 (d, *J* = 11.3 Hz, 1H, Ph-**CH**), 4.77 (d, *J* = 12.3 Hz, 1H, Ph-**CH**), 4.65 (d, *J* = 12.4 Hz, 1H, Ph-**CH**), 4.50 (d, *J* = 3.9 Hz, 1H, H-1', overlap with H-5'), 4.48 (dd, *J* = 9.4 Hz, 1.9 Hz, 1H, H-5', overlap with H-1'), 4.03 (t, *J* = 9.1 Hz, 1H, H-3'), 3.99 (t, *J* = 9.3 Hz, 1H, H-4'), 3.84 (s, 3H, OCH_3), 3.80 (s, 3H, OCH_3), 3.77 (s, 3H, OCH_3), 3.61 (ddd, *J* = 12.3, 6.1, 1.9 Hz, 1H, H-3), 3.52 (dd, *J* = 9.2, 3.6 Hz, 1H, H-2'), 3.09 (s, 3H, OCH_3), 2.65 (dd, *J* = 15.3, 10.7 Hz, 1H, H-4a), 2.43 (dd, *J* = 15.6, 2.2 Hz, 1H, H-4b), 1.28 (d, *J* = 6.1 Hz, 3H, CH_3). ^{13}C NMR (126 MHz, $CDCl_3$) δ (ppm): 151.9, 150.1, 139.9 (3C, C-7 – C-9), 139.2, 139.1, 138.6 (3C, aromatic), 132.3 (1C, C-5/C-10), 128.3, 128.0, 127.9, 127.7, 127.7, 127.5, 127.5 (15C, aromatic), 120.3 (1C, C-5/C-10), 107.1 (C-6), 98.1 (C-1'), 82.6 (C-3'), 80.1 (C-2'), 78.2 (C-4'), 75.8, 74.9, 73.2 (3C, 3 x Ph-**C**H_2), 71.5 (1C, C-1), 71.0 (1C, C-5'), 69.6 (1C, C-3), 60.6, 60.3, 55.8, 54.7 (4C, 4 x OCH_3), 37.0 (1C, C-4), 21.5 (1C, CH_3). MALDI-ToF HRMS: m\z calcd for $C_{40}H_{46}NaO_9^+$ $[M+Na]^+$ 693.3034, found 693.3037.

7. (1*S*,3*S*)-1-(Methyl 2,3,4-tri-*O*-benzyl-β-D-xylopyranoside-5-yl)-7,8-dimethoxy-3-methylisochroman (18)

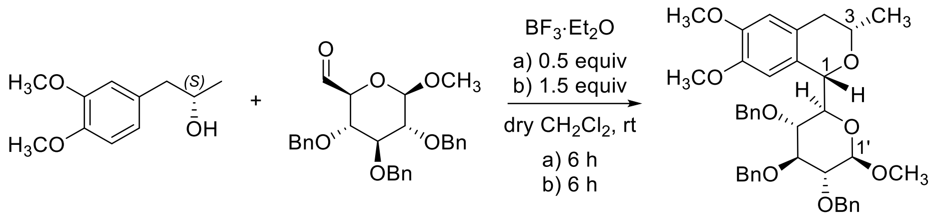

(*S*)-**1** **15** **18**

a) Alcohol (*S*)-**1** (50 mg, 0.25 mmol, 1 equiv.), dialdose **15** (142 mg, 0.31 mmol, 1.2 equiv.), dry CH_2Cl_2 (5 mL), and $BF_3 \cdot Et_2O$ (16 µL, 0.13 mmol, 0.5 equiv.) were used according to method B. Reaction time: 6 h. The reaction mixture was quenched with saturated $NaHCO_3$ solution (1 mL) and stirred for a further 5 min at room temperature. The crude product was purified by flash column chromatography on silica gel (hexane: acetone 5:1) to afford **18** (106 mg, 65%) as a white amorphous solid.
b) The reaction was repeated on the same scale using 1.5 equiv of $BF_3 \cdot Et_2O$ to give compound **18** with 76% yield.
R_f = 0.18 (hexane: acetone 3:1). $[\alpha]_D$ +79.0 (c 0.10, $CHCl_3$). ^1H NMR (400 MHz, $CDCl_3$) δ (ppm): 7.38 – 7.21 (m, 15H, aromatic), 6.58 (s, 1H, H-9), 6.57 (s, 1H, H-6), 5.10 (d, J = 2.4 Hz, 1H, H-1), 4.95 (d, J = 11.0 Hz, 1H, Ph-**CH**), 4.91 (d, J = 10.6 Hz, 1H, Ph-**CH**), 4.87 (d, J = 11.4 Hz, 1H, Ph-**CH**, overlap with Ph-CH), 4.85 (d, J = 11.0 Hz, 1H, Ph-**CH**, overlap with Ph-CH), 4.76 (d, J = 10.6 Hz, 1H, Ph-**CH**), 4.68 (d, J = 11.2 Hz, 1H, Ph-**CH**), 4.19 (d, J = 7.8 Hz, 1H, H-1'), 4.09 (t, J = 9.3 Hz, 1H, H-4'), 3.84 (s, 3H, OCH_3, aromatic), 3.81 (m, 1H, H-3, overlap with aromatic OCH_3), 3.80 (s, 1H, OCH_3, aromatic), 3.74 (dd, J = 9.8, 2.4 Hz, 1H, H-5', overlap with H-3'), 3.71 (t, J = 9.1 Hz, 1H, H-3', overlap with H-5'), 3.46 (dd, J = 9.0, 7.8 Hz, 1H, H-2'), 3.19 (s, 1H, OCH_3, anomeric), 2.78 (dd, J = 15.8, 10.9 Hz, 1H, H-4a), 2.55 (dd, J = 16.0, 1.9 Hz, 1H, H-4b), 1.35 (d, J = 6.1 Hz, 3H, CH_3). ^{13}C NMR (101 MHz, $CDCl_3$) δ (ppm): 147.6, 147.3 (2C, C-7, C-8), 138.8, 138.64 138.6, 128.5, 128.4, 128.3, 128.1, 128.0, 127.9 (aromatic), 127.8 (1C, C-5/C-10), 127.6 (aromatic, overall 18C aromatic), 126.6 (1C, C-5/C-10), 111.4 (1C, C-9), 106.8 (1C, C-6), 105.2 (1C, C-1'), 84.9 (1C, C-3'), 82.3 (1C, C-2'), 77.8 (1C, C-4'), 75.7 (1C, C-5'), 75.7, 75.3, 74.6 (3C, 3 x Ph-**C**H_2), 73.9 (1C, C-1), 71.2 (1C, C-3), 56.7 (1C, OCH_3, anomeric), 56.0, 55.8 (2C, 2 x OCH_3, aromatic), 35.8 (1C, C-4), 21.7 (1C, CH_3). MALDI-ToF HRMS: m\z calcd for $C_{18}H_{26}NaO_8^+$ $[M+Na]^+$ 663.2928, found 663.2921.

8. (1*S*,3*S*)-1-(Methyl 2,3,4-tri-*O*-benzyl-β-D-xylopyranoside-5-yl)-7,8,9-trimethoxy-3-methylisochroman (19)

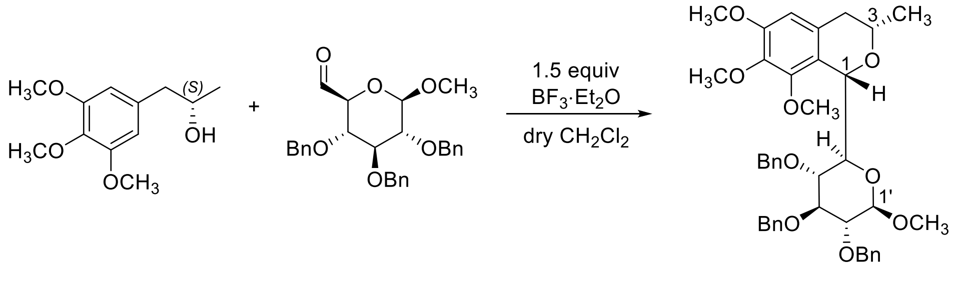

(*S*)-**12** **15** **19**

(*S*)-**12** (113 mg, 0.5 mmol, 1 equiv.), dialdose **15** (278 mg, 0.6 mmol, 1.2 equiv.), dry CH_2Cl_2 (10 mL), and $BF_3 \cdot Et_2O$ (93 µL, 0.75 mmol, 1.5 equiv.) were used according to method B. Reaction time: 4 h. The reaction mixture was quenched with saturated $NaHCO_3$ solution (2 mL) and stirred

for a further 5 min at room temperature. The crude product was purified by flash column chromatography on silica gel (hexane: acetone 9:1) to afford (239 mg, 72%) **19** as a colorless syrup. Compound **19**: R_f = 0.45 (hexane: acetone 7:3). $[\alpha]_D$ +53.1 (c 0.26, $CHCl_3$). ^{1}H NMR (500 MHz, $CDCl_3$) δ (ppm): 7.38 – 7.22 (m, 15H, aromatic), 6.38 (s, 1H, H-6), 5.25 (s, 1H, H-1), 4.92 (d, *J* = 10.8 Hz, 1H, Ph-**CH**, overlap with Ph-CH), 4.92 (d, *J* = 10.8 Hz, 1H, Ph-**CH**, overlap with Ph-CH), 4.88 (d, *J* = 11.3 Hz, 1H, Ph-**CH**), 4.86 (d, *J* = 11.7 Hz, 1H, Ph-**CH**), 4.84 (d, *J* = 11.2 Hz, 1H, Ph-**CH**), 4.67 (d, *J* = 11.3 Hz, 1H, Ph-**CH**), 4.16 (d, J = 7.8 Hz, 1H, H-1'), 4.09 – 4.04 (m, 2H, H-4', H-5'), 3.85 (s, 3H, OCH_3, aromatic), 3.82 (s, 3H, OCH_3, aromatic), 3.79 (s, 3H, OCH_3, aromatic), 3.73 – 3.67 (m, 2H, H-3', H-3), 3.40 (dd, *J* = 8.9, 8.0 Hz, 1H, H-2'), 3.14 (s, 3H, OCH_3, anomeric), 2.75 (dd, J = 15.4, 10.9 Hz, 1H, H-4a), 2.47 (d, J = 14.3 Hz, 1H, H-4b), 1.30 (d, *J* = 6.0 Hz, 3H, CH_3). ^{13}C NMR (126 MHz, $CDCl_3$) δ (ppm): 152.0, 150.1, 139.9 (3C, C-7 – C-9), 139.2, 139.0, 138.8 (3C, aromatic), 132.5 (1C, C-5/C-10), 128.4, 128.4, 128.3, 128.1, 128.0, 127.6, 127.6 (15C, aromatic), 120.1 (1C, C-5/C-10), 107.0 (1C, C-6), 104.7 (1C, C-1'), 85.3 (1C, C-3'), 82.6 (1C, C-2'), 78.3 (1C, C-4'), 75.8, 75.1 (2C, 2 x Ph-CH_2), 75.0 (1C, C-5'), 74.6 (1C, Ph-CH_2), 71.9 (1C, C-1), 69.8 (1C, C-3), 60.8, 60.6, 56.2, 56.0 (4C, 4 x OCH_3), 25.7 (1C, C-4), 21.6 (1C, CH_3). MALDI-ToF HRMS: m\z calcd for $C_{40}H_{46}NaO_9^+$ $[M+Na]^+$ 693.3034, found 693.3037.

9. (1S,3S)-1-(Methyl 2,3,4-tri-*O*-benzyl-β-D-xylopyranoside-5-yl)-7,8-dibenzeloxy-3-methylisochroman (20)

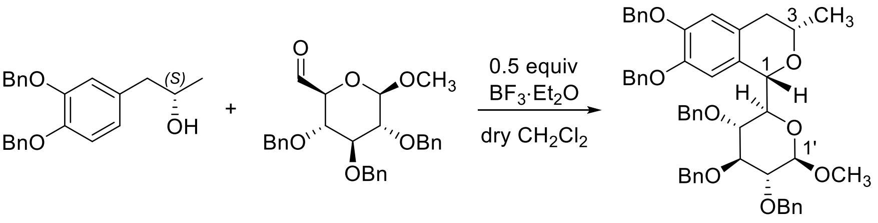

(*S*)-**13** **15** **20**

(*S*)-**13** (101 mg, 0.29 mmol, 1 equiv.), dialdose **15** (188 mg, 0.40 mmol, 1.4 equiv.), dry CH_2Cl_2 (12 mL) and $BF_3{\cdot}Et_2O$ (18 µL, 0.14 mmol, 0.5 equiv.) were used according to method B. Reaction time 6 h. The reaction mixture was quenched with saturated $NaHCO_3$ solution (3 mL) and stirred for a further 5 min at room temperature. The crude product was purified by flash column chromatography on silica gel (hexane: EtOAc 7:1) to afford compound **20** (183 mg, 80%) as a white powder. R_f = 0.20 (hexane: acetone 7:1). $[\alpha]_D$ +51.2 (c 0.08, $CHCl_3$). ^{1}H NMR (500 MHz, $CDCl_3$) δ (ppm): 7.42 (d, *J* = 7.8 Hz, 4H, aromatic), 7.37 – 7.23 (m, 21H, aromatic), 6.67 (s, 1H, H-9), 6.65 (s, 1H, H-6), 5.15 – 5.07 (m, 3H, 3 x Ph-**CH**), 5.05 (s, 1H, H-1, overlap with Ph-CH), 5.03 (d, *J* = 12.3 Hz, 1H, Ph-**CH**, overlap with H-1), 4.94 (d, *J* = 11.0 Hz, 1H, Ph-**CH**), 4.90 – 4.81 (m, 3H, 3 x Ph-**CH**), 4.72 (d, *J* = 10.6 Hz, 1H, Ph-**CH**), 4.66 (d, *J* = 11.2 Hz, 1H, Ph-**CH**), 4.03 (t, *J* = 9.4 Hz, 1H, H-4', overlap with H-1'), 4.02 (d, *J* = 7.8 Hz, 1H, H-1', overlap with H-4'), 3.76 (ddd, *J* = 10.9, 6.1, 2.8 Hz, 1H, H-3), 3.67 (t, *J* = 9.1 Hz, 1H, H-3'), 3.59 (dd, *J* = 9.6, 2.0 Hz, 1H, H-5'), 3.41 (dd, *J* = 9.0, 7.9 Hz, 1H, H-2'), 3.06 (s, 3H, OCH_3), 2.71 (dd, *J* = 15.4, 11.2 Hz, 1H, H-4a), 2.48 (dd, *J* = 15.7, 1.7 Hz, 1H, H-4b), 1.31 (d, *J* = 6.1 Hz, 3H, CH_3). ^{13}C NMR (126 MHz, $CDCl_3$) δ (ppm): 147.5, 147.3 (2C, C-7, C-8), 138.8, 138.7, 138.5, 137.5, 137.3 (5C, aromatic), 128.9 (2C, C-5, C-10), 128.6, 128.5, 128.5, 128.4, 128.3, 128.2, 128.1, 127.9, 127.9, 127.9, 127.8, 127.6, 127.4, 127.2 (25C, aromatic), 115.2 (1C, C-6), 110.9 (1C, C-9), 104.9 (1C, C-1'), 84.9 (1C, C-3'), 82.3 (1C, C-2'), 77.8 (1C, C-4'), 75.7 (1C, C-5'), 75.7, 75.3, 74.5 (3C, 3 x Ph-CH_2), 73.9 (1C, C-1), 71.8, 71.3 (2C, 2 x Ph-CH_2), 71.0 (1C, C-3), 56.5 (1C, OCH_3), 35.8 (1C, C-4), 21.6 (1C, CH_3). MALDI-ToF HRMS: m\z calcd for $C_{51}H_{52}NaO_8^+$ $[M+Na]^+$ 815.3554, found 815.3550.

10. (1*S*,3*S*)-1-(Methyl α-D-xylopyranoside-5-yl)-7,8,9-trimethoxy-3-methylisochroman (21)

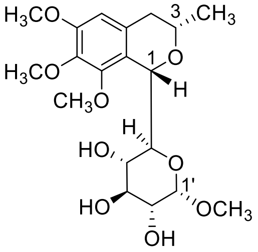

21

Compound **17** (90 mg, 0.13 mmol) was deprotected according to the general method C to give compound **21** (52 mg, 98% yield) as a white amorphous solid. R_f = 0.67 (CH_2Cl_2: MeOH 9:1). $[\alpha]_D$ +193.3 (c 0.09, MeOH). ^{1}H NMR (700 MHz, MeOD) δ (ppm): 6.51 (s, 1H, H-6), 5.31 (s, 1H, H-1), 4.46 (d, *J* = 3.8 Hz, 1H, H-1'), 4.25 (dd, *J* = 9.8, 1.8 Hz, 1H, H-5'), 3.84 (s, 3H, OCH_3, aromatic), 3.81 (s, 3H, OCH_3, aromatic), 3.78 (t, *J* = 9.7 Hz, 1H, H-4'), 3.75 (s, 3H, OCH_3, aromatic), 3.64 (t, *J* = 9.2 Hz, 2H, H-3, H-3'), 3.38 (dd, *J* = 9.6, 3.8 Hz, 1H, H-2'), 3.00 (s, 3H, OCH_3, anomeric), 2.61 (dd, *J* = 15.1, 10.7 Hz, 1H, H-4a), 2.49 (d, *J* = 15.0 Hz, 1H, H-4b), 1.29 (d, *J* = 6.1 Hz, 3H, CH_3). ^{13}C NMR (176 MHz, MeOD) δ (ppm): 153.3, 151.1, 141.2 (3C, C-7 – C-9), 134.3 (1C, C-5), 121.5 (1C, C-10), 108.5 (1C, C-6), 101.0 (1C, C-1'), 75.5 (1C, C-3'), 73.6 (1C, C-2'), 73.3 (1C, C-5'), 72.2 (1C, C-1), 71.0, 71.0 (2C, C-4', C-3), 61.0, 60.9, 56.4 (3C, 3 x OCH_3, aromatic), 55.0 (1C, OCH_3, anomeric), 38.0 (1C, C-4), 21.7 (1C, CH_3). MALDI-ToF HRMS: m\z calcd for $C_{19}H_{28}NaO_9^+$ $[M+Na]^+$ 423.1626, found 423.1620.

11. (1*S*,3*S*)-1-(Methyl β-D-xylopyranoside-5-yl)-7,8-dimethoxy-3-methylisochroman (22)

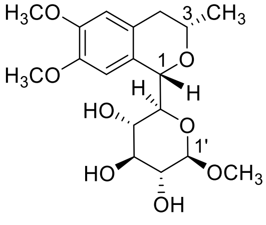

22

Compound **18** (53 mg, 0.15 mmol, 1 equiv.) was deprotected according to the general method C to give compound **22** (38 mg, 82%) as a pale yellow solid. R_f = 0.24 (CH_2Cl_2: methanol 9:1). $[\alpha]_D$ +62.4 (c 0.101, DMSO). M.p: 222-224 °C. ^{1}H NMR (700 MHz, CD_3SOCD_3) δ (ppm): 6.73 (s, 1H, H-9), 6.63 (s, 1H, H-6), 5.11 (s, 2H, 2 x OH), 4.88 (s, 1H, H-1), 4.03 (d, *J* = 7.9 Hz, 1H, H-1'), 3.74 (d, *J* = 9.7 Hz, 1H, H-5'), 3.70 (s, 7H, 2 x OCH_3, H-3), 3.56 (t, *J* = 9.1 Hz, 1H, H-4'), 3.25 (t, *J* = 8.8 Hz, 1H, H-3'), 3.02 (s, 3H, OCH_3, anomeric), 2.92 (t, *J* = 8.3 Hz, 1H, H-2'), 2.60 – 2.55 (m, 1H, H-4a), 2.54 – 2.50 (m, 1H, H-4b, overlap with CD_3SOCD_3 signal), 1.25 (d, *J* = 6.0 Hz, 3H, CH_3). ^{13}C NMR (126 MHz, CD_3SOCD_3) δ (ppm): 147.0, 146.9 (2C, C-7, C-8), 127.4, 126.8 (2C, C-5, C-10), 111.6 (1C, C-6), 107.7 (1C, C-9), 104.2 (1C, C-1'), 77.2 (1C, C-3'), 75.2 (1C, C-5'), 73.5 (1C, C-1), 73.4 (1C, C-2'), 70.4 (1C, C-3), 68.9 (1C, C-4'), 55.7, 55.6, 55.3 (3C, 3 x OCH_3), 35.4 (1C, C-4), 21.7 (1C, CH_3). MALDI-ToF HRMS: m\z calcd for $C_{18}H_{26}NaO_8^+$ $[M+Na]^+$ 393.1520, found 393.1521.

12. (1*S*,3*S*)-1-(Methyl β-D-xylopyranoside-5-yl)-7,8,9-trimethoxy-3-methylisochroman (23)

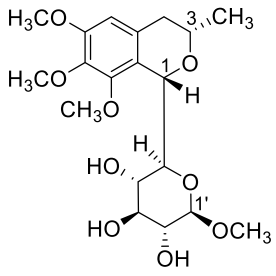

23

Compound **19** (170 mg, 0.25 mmol) was deprotected according to method C to afford compound **23** as a white solid (99 mg, 98%). R_f = 0.50 (CH_2Cl_2: methanol 9:1). $[\alpha]_D$ +74.2 (c 0.12, MeOH). M.p: 153-155 °C. ^{1}H NMR (500 MHz, MeOD) δ (ppm): 6.51 (s, 1H, H-6), 5.26 (s, 1H, H-1), 3.95 (d, J = 7.8 Hz, 1H, H-1'), 3.93 (dd, J = 9.7, 2.2 Hz, 1H, H-5'), 3.85 (s, 3H, OCH_3, aromatic), 3.86 – 3.80 (m, 1H, H-4'), 3.80 (s, 3H, OCH_3, aromatic), 3.76 (s, 3H, OCH_3, aromatic), 3.65 (dtd, J = 12.0, 6.0, 2.2 Hz, 1H, H-3), 3.40 (t, J = 9.1 Hz, 1H, H-3'), 3.15 (dd, J = 9.2, 7.8 Hz, 1H, H-2'), 3.09 (s, 3H, OCH_3, anomeric), 2.64 (ddt, J = 15.5, 10.8, 1.3 Hz, 1H, H-4a), 2.50 (dd, J = 15.2, 2.3 Hz, 1H, H-4b), 1.29 (d, J = 6.1 Hz, 3H, CH_3). ^{13}C NMR (126 MHz, MeOD) δ (ppm): 153.3, 151.1, 141.2 (3C, C-7 – C-9), 134.0, 121.2 (2C, C-5, C-10), 108.4 (1C, C-6), 105.3 (1C, C-1'), 78.5 (1C, C-3'), 77.3 (1C, C-5'), 75.1 (1C, C-2'), 72.5 (1C, C-1), 71.0 (1C, C-3), 70.8 (1C, C-4'), 61.1, 61.1 (2C, 2 x OCH_3, aromatic), 56.5 (1C, OCH_3, anomeric), 56.4 (1C, OCH_3, aromatic), 37.7 (1C, C-4), 21.7 (1C, CH_3). MALDI-ToF HRMS: m\z calcd for $C_{19}H_{28}NaO_9^+$ $[M+Na]^+$ 423.1626, found 423.1627.

13. (1*S*,3*S*)-1-(Methyl β-D-xylopyranoside-5-yl)-7,8-diol-3-methylisochroman (24)

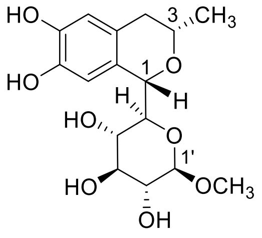

24

Compound **20** (159 mg, 0.2 mmol, 1 equiv.) was converted to compound **24** according to method C. The crude product was purified by flash column chromatography on silica gel (CH_2Cl_2: methanol 7:1) to afford compound **24** (49 mg, 72%) as white crystals. R_f = 0.15 (CH_2Cl_2: methanol 7:1). $[\alpha]_D$ -46.0 (c 0.05, MeOH). M.p: 148-150°C. ^{1}H NMR (500 MHz, MeOD) δ (ppm): 6.61 (s, 1H, H-9), 6.50 (s, 1H, H-6), 4.99 (s, 1H, H-1), 4.06 (d, J = 7.9 Hz, 1H, H-1'), 3.80 (t, J = 9.3 Hz, 1H, H-4'), 3.75 (ddd, J = 11.0, 6.1, 3.0 Hz, 1H, H-3), 3.63 (dd, J = 9.7, 2.0 Hz, 1H, H-5'), 3.43 (t, J = 9.1 Hz, 1H, H-3'), 3.20 (s, 3H, OCH_3), 3.19 (dd, J = 9.2, 7.9 Hz, 1H, H-2'), 2.59 (dd, J = 15.6, 11.0 Hz, 1H, H-4a), 2.47 (dd, J = 15.5, 2.8 Hz, 1H, H-4b), 1.30 (d, J = 6.1 Hz, 3H, CH_3). ^{13}C NMR (126 MHz, MeOD) δ (ppm): 144.8, 144.6 (2C, C-7, C-8), 127.9, 127.1 (2C, C-5, C-10), 116.0 (1C, C-6), 111.5 (1C, C-9), 105.6 (1C, C-1'), 78.5 (1C, C-3'), 77.8 (1C, C-5'), 75.0 (2C, C-1, C-2'), 72.5 (1C, C-3), 70.7 (1C, H-4'), 57.0 (1C, OCH_3), 36.6 (1C, C-4), 21.8 (1C, CH_3). MALDI-ToF HRMS: m\z calcd for $C_{16}H_{22}NaO_8^+$ $[M+Na]^+$ 365.1207, found 365.1213.

14. (1*R*,3*R*)-1-(Methyl 2,3,4-tri-*O*-benzyl-α-D-xylopyranoside-5-yl)-7,8-dimethoxy-3-methylisochroman (25) and **(1*S*,3*R*)-1-(methyl 2,3,4-tri-*O*-benzyl-α-D-xylopyranoside-5-yl)-7,8-dimethoxy-3-methylisochroman (26)**

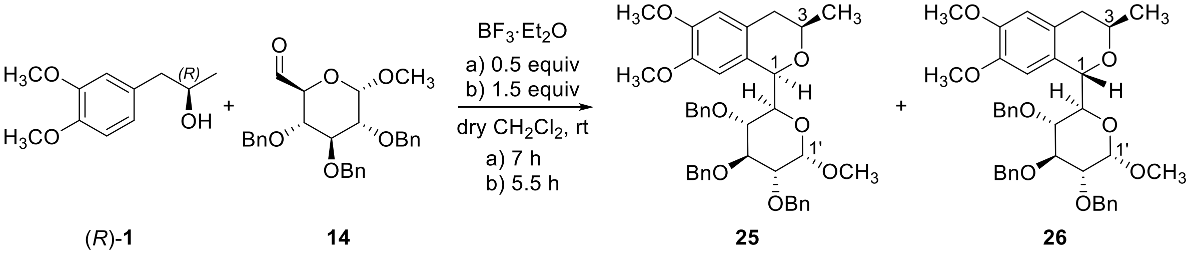

a) Alcohol (*R*)-**1** (51 mg, 0.26 mmol, 1 equiv.), dialdose **14** (140 mg, 0.31 mmol, 1.2 equiv.), dry CH_2Cl_2 (5 mL) and $BF_3{\cdot}Et_2O$ (16 µL, 0.13 mmol, 0.5 equiv.) were used according to method B. Reaction time: 7 h. The reaction mixture was quenched with saturated $NaHCO_3$ solution (2 mL) and stirred for a further 5 min at room temperature. The crude product was purified by flash column chromatography on silica gel (CH_2Cl_2: EtOAc 99:1) to afford compound **25** (76 mg, 46%) as a pale yellow amorphous and compound **26** (7 mg, 4%) as a white amorphous.
b) Alcohol (*R*)-**1** (51 mg, 0.26 mmol, 1 equiv.), dialdose **14** (140 mg, 0.31 mmol, 1.2 equiv.), dry CH_2Cl_2 (5 mL) and $BF_3{\cdot}Et_2O$ (50 µL, 0.4 mmol, 1.5 equiv.) were used according to method B. Reaction time: 5.5 h. The reaction mixture was quenched with saturated $NaHCO_3$ solution (2 mL) and stirred for a further 5 min at room temperature. The crude product was purified by flash column chromatography on silica gel (CH_2Cl_2: EtOAc 99:1) to afford compound **26** (105 mg, 63%) as a white amorphous and compound **25** (37 mg, 23%) as a pale yellow amorphous.
Compound **25**: R_f = 0.34 (CH_2Cl_2: acetone 99:1). $[\alpha]_D$ -53.3 (c 0.21, $CHCl_3$). ^1H NMR (500 MHz, $CDCl_3$) δ (ppm): 7.42 – 7.20 (m, 10H, aromatic), 7.14 – 7.04 (m, 3H, aromatic), 6.64 (s, 1H, H-9), 6.58 (dd, J = 7.3, 2.2 Hz, 2H, aromatic), 6.38 (s, 1H, H-6), 5.01 (s, 1H, H-1), 4.92 (d, J = 10.7 Hz, 1H, Ph-**CH**), 4.81 (d, J = 12.2 Hz, 1H, Ph-**CH**), 4.73 – 4.65 (m, 4H, H-1', 3 x Ph-**CH**), 4.42 (d, J = 10.9 Hz, 1H, Ph-**CH**), 4.34 – 4.23 (m, 1H, H-5'), 4.05 – 3.97 (m, 2H, H-3', H-4'), 3.83 (s, 3H, OCH_3, aromatic), 3.83 – 3.79 (m, 1H, H-3, overlap with aromatic OCH_3), 3.77 (s, 3H, OCH_3, aromatic), 3.58 (dd, J = 9.2, 3.8 Hz, 1H, H-2'), 3.49 (s, 3H, OCH_3, anomeric), 2.58 (dd, J = 15.1, 11.2 Hz, 1H, H-4a,), 2.48 (dd, J = 15.5, 2.3 Hz, 1H, H-4b), 1.35 (d, J = 6.0 Hz, 3H, CH_3). ^{13}C NMR (101 MHz, $CDCl_3$) δ (ppm): 147.9, 147.6 (2C, C-7, C-8), 138.8, 138.5, 138.3, 128.5, 128.4, 128.2, 128.0, 127.9, 127.8, 127.6, 126.9 (aromatic), 126.8 (1C, C-5), 126.7 (aromatic, overall 18C aromatic), 126.2 (1C, C-10), 111.6 (1C, C-6), 108.5 (1C, C-9), 98.5 (1C, C-1'), 82.8 (1C, C-3'), 79.7 (1C, C-2'), 77.5 (1C, C-1), 77.4 (1C, C-4'), 75.9, 74.0, 73.4 (3C, 3 x Ph-$\mathbf{CH_2}$), 73.1 (1C, C-5'), 70.6 (1C, C-3), 56.3, 55.8 (2C, 2 x OCH_3, aromatic), 55.4 (1C, OCH_3, anomeric), 36.4 (1C, C-4), 21.9 (1C, CH_3). MALDI-ToF HRMS: m\z calcd for $C_{39}H_{44}NaO_8^+$ $[M+Na]^+$ 663.2928, found 663.2930.
Compound **26**: R_f = 0.40 (CH_2Cl_2: acetone 99:1). $[\alpha]_D$ +15.9 (c 0.22, $CHCl_3$). ^1H NMR (500 MHz, $CDCl_3$) δ (ppm): 7.40 (d, *J* = 7.0 Hz, 2H, aromatic), 7.38 – 7.24 (m, 13H, aromatic), 6.63 (s, 1H, H-9), 6.59 (s, 1H, H-6), 5.15 (d, *J* = 2.0 Hz, 1H, H-1), 5.00 (d, *J* = 10.8 Hz, 1H, Ph-**CH**), 4.95 (d, *J* = 10.2 Hz, 1H, Ph-**CH**), 4.91 (d, *J* = 10.8 Hz, 1H, Ph-**CH**), 4.85 (d, *J* = 10.3 Hz, 1H, Ph-**CH**), 4.79 (d, *J* = 12.2 Hz, 1H, Ph-**CH**), 4.64 (d, *J* = 12.2 Hz, 1H, Ph-**CH**), 4.44 (d, *J* = 3.5 Hz, 1H, H-1'), 4.33 (ddd, *J* = 9.4, 6.2, 3.3 Hz, 1H, H-3), 4.07 (dd, *J* = 9.9, 2.2 Hz, 1H, H-5', overlap with H-3'), 4.05 (t, *J* = 9.3 Hz, 1H, H-3', overlap with H-5'), 3.89 (t, *J* = 9.5 Hz, 1H, H-4'), 3.85 (s, 3H, OCH_3, aromatic), 3.80 (s, 3H, OCH_3, aromatic), 3.52 (dd, *J* = 9.7, 3.6 Hz, 1H, H-2'), 3.05 (s, 3H, OCH_3, anomeric), 2.68 (dd, *J* = 15.9, 3.2 Hz, 1H, H-4a), 2.51 (dd, *J* = 15.7, 9.4 Hz, 1H, H-4b), 1.28 (d, *J* = 6.2 Hz, 3H, CH_3). ^{13}C NMR (126 MHz, $CDCl_3$) δ (ppm): 147.5, 147.3 (2C, C-7, C-8), 138.9, 138.5, 138.4, 128.6, 128.5, 128.4, 128.2, 128.0, 127.9, 127.8 (18C, aromatic), 126.8, 126.0 (2C, C-5, C-10), 111.5 (1C, C-6), 107.2 (1C, C-9), 97.9 (1C, C-1'), 82.5 (1C, C-3'), 80.4 (1C, C-2'), 78.5

(1C, C-4'), 76.1, 75.7 (2C, 2 x Ph-**C**H_2), 74.8 (1C, C-5'), 73.5 (1C, Ph-**C**H_2), 70.9 (1C, C-1), 67.2 (1C, C-3), 56.0, 55.9 (2C, 2 x OCH_3, aromatic), 55.1 (1C, OCH_3, anomeric), 35.4 (1C, C-4), 21.3 (1C, CH_3). MALDI-ToF HRMS: m\z calcd for $C_{39}H_{44}NaO_8^+$ $[M+Na]^+$ 663.2930, found 663.2941.

15. (1*R*,3*R*)-1-(Methyl 2,3,4-tri-*O*-benzyl-α-D-xylopyranoside-5-yl)-7,8,9-trimethoxy-3-methylisochroman (27)

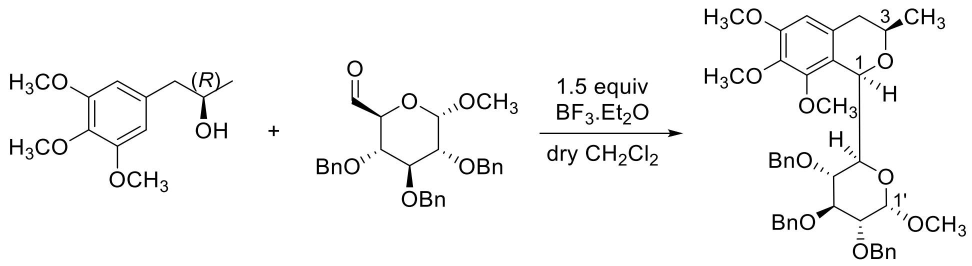

(*R*)-**12** **14** **27**

Alcohol (*R*)-**12** (50 mg, 0.22 mmol, 1 equiv.), dialdose **14** (123 mg, 0.26 mmol, 1.2 equiv.), dry CH_2Cl_2 (5 mL), and $BF_3·Et_2O$ (45 μL, 0.33 mmol, 1.5 equiv.) were used according to method B. Reaction time 4 h. The reaction mixture was quenched with saturated $NaHCO_3$ solution (1.5 mL) and stirred for a further 5 min at room temperature. The crude product was purified by flash column chromatography on silica gel (hexane: EtOAc 5:1) to afford compound **27** (80 mg, 55%) as a colorless syrup. R_f = 0.18 (hexane: EtOAc 5:1). $[\alpha]_D$ -70 (c 0.06, $CHCl_3$). ^1H NMR (500 MHz, $CDCl_3$) δ (ppm): 7.37 (d, J = 6.8 Hz, 2H, aromatic), 7.35 – 7.25 (m, 3H, aromatic), 7.26 – 7.18 (m, 5H, aromatic), 7.09 (dd, J = 4.9, 1.6 Hz, 3H, aromatic), 6.63 (dd, J = 6.4, 2.9 Hz, 2H, aromatic), 6.05 (s, 1H, H-6), 5.09 (s, 1H, H-1), 4.92 (d, J = 10.7 Hz, 1H, Ph-**CH**), 4.79 (d, J = 12.2 Hz, 1H, Ph-**CH**), 4.76 (d, J = 12.1 Hz, 1H, Ph-**CH**, overlap with H-1'), 4.74 (d, J = 3.8 Hz, 1H, H-1', overlap with Ph-CH), 4.73 – 4.70 (m, 1H, H-5'), 4.68 (d, J = 12.2 Hz, 1H, Ph-**CH**), 4.64 (d, J = 10.7 Hz, 1H, Ph-**CH**), 4.32 (d, J = 11.3 Hz, 1H, Ph-**CH**), 4.03 – 3.94 (m, 2H, H-3', H-4'), 3.91 (s, 3H, OCH_3), 3.78 (s, 3H, OCH_3), 3.72 (ddd, J = 10.6, 6.0, 2.1 Hz, 1H, H-3), 3.66 (s, 3H, OCH_3), 3.60 (dd, J = 9.2, 3.6 Hz, 1H, H-2'), 3.51 (s, 3H, OCH_3), 2.55 (dd, J = 15.3, 11.0 Hz, 1H, H-4a), 2.40 (d, J = 15.0 Hz, 1H, H-4b), 1.34 (d, J = 6.0 Hz, 3H, CH_3). ^{13}C NMR (126 MHz, $CDCl_3$) δ (ppm): 152.0, 150.2, 140.4 (3C, C-7 – C-9), 139.0, 138.8, 138.3 (3C, aromatic), 129.8 (1C, C-5/C-10), 128.4, 128.3, 128.1, 128.0, 127.8, 127.5, 127.5, 126.5, 126.1 (15C, aromatic), 120.3 (1C, C-5/C-10), 107.2 (1C, C-6), 98.1 (1C, C-1'), 83.2 (1C, C-3'), 79.9 (1C, C-2'), 77.0 (1C, C-4'), 75.7 (1C, Ph-**C**H_2), 75.5 (1C, C-1), 73.2 (2C, Ph-**C**H_2), 71.0 (1C, C-5'), 69.7 (1C, C-3), 60.7, 60.2, 55.6, 54.9 (4C, 4 x OCH_3), 37.4 (1C, C-4), 21.76 (1C, CH_3). MALDI-ToF HRMS: m\z calcd for $C_{40}H_{46}NaO_9^+$ $[M+Na]^+$ 693.3034, found 693.3040.

16. (1*R*,3*R*)-1-(Methyl 2,3,4-tri-*O*-benzyl-β-D-xylopyranoside-5-yl)-7,8-dimethoxy-3-methylisochroman (28) and **(1*S*,3*R*)-1-(methyl 2,3,4-tri-*O*-benzyl-β-D-xylopyranoside-5-yl)-7,8-dimethoxy-3-methylisochroman (29)**

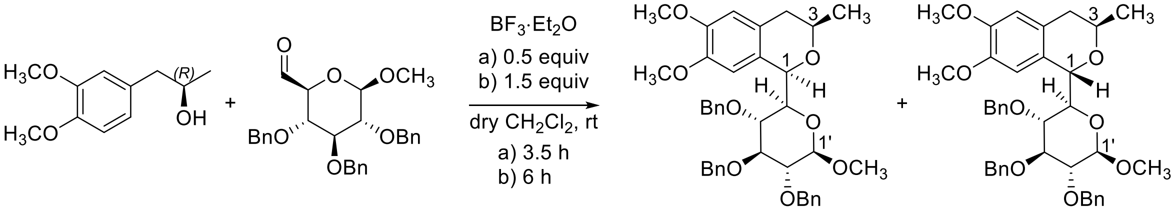

(*R*)-**1** **15** **28** **29**

a) Alcohol (*R*)-**1** (51 mg, 0.26 mmol, 1 equiv.), dialdose **15** (143 mg, 0.31 mmol, 1.2 equiv.) dry CH_2Cl_2 (5 mL) and $BF_3·Et_2O$ (16 μL, 0.13 mmol, 0.5 equiv.) were used according to method B.

Reaction time: 3.5 h. The reaction mixture was quenched with saturated $NaHCO_3$ solution (1 mL) and stirred for a further 5 min at room temperature. The crude product was purified by flash column chromatography on silica gel (hexane: EtOAc 5.5:1) to afford compound **28** as white solid (90 mg, 54%) and compound **29** as white solid (29 mg, 17%).

b) The reaction was repeated in the same scale using 1.5 equiv of $BF_3 \cdot Et_2O$ to give compound **28** with 33% yield and compound **29** with 51% yield.

Compound **28**: R_f = 0.15 (hexane: EtOAc 1:1). $[\alpha]_D$ -18.1 (c 0.16, $CHCl_3$). ^{1}H NMR (400 MHz, $CDCl_3$) δ (ppm): 7.41 – 7.19 (m, 10H, aromatic), 7.16 – 7.04 (m, 3H, aromatic), 6.68 (s, 1H, H-9), 6.56 (dd, *J* = 7.6, 1.7 Hz, 2H, aromatic), 6.41 (s, 1H, H-6), 5.09 (s, 1H, H-1), 4.93 (d, *J* = 11.2 Hz, 1H, Ph-**CH**), 4.87 (d, *J* = 10.9 Hz, 1H, Ph-**CH**), 4.73 (d, *J* = 11.2 Hz, 1H, Ph-**CH**), 4.69 (d, *J* = 11.0 Hz, 1H, Ph-**CH**), 4.65 (d, *J* = 10.8 Hz, 1H, Ph-**CH**), 4.53 (d, *J* = 11.0 Hz, 1H, Ph-**CH**), 4.42 (d, *J* = 7.8 Hz, 1H, H-1'), 4.09 (t, *J* = 9.2 Hz, 1H, H-4'), 3.96 (dd, *J* = 9.4, 1.2 Hz, 1H, H-5'), 3.86 (s, 3H, OCH_3, aromatic), 3.87 – 3.79 (m, 1H, H-3, overlap with aromatic OCH_3), 3.80 (s, 3H, OCH_3, aromatic), 3.67 (t, *J* = 9.0 Hz, 1H, H-3', overlap with anomeric OCH_3), 3.64 (s, 3H, OCH_3, anomeric), 3.47 (dd, *J* = 9.1, 7.8 Hz, 1H, H-2'), 2.58 (dd, *J* = 15.3, 11.2 Hz, 1H, H-4a), 2.49 (dd, *J* = 15.8, 2.1 Hz, 1H, H-4b), 1.35 (d, *J* = 6.0 Hz, 3H, CH_3). ^{13}C NMR (101 MHz, $CDCl_3$) δ (ppm): 147.7, 147.5 (2C, C-7, C-8), 138.7, 138.7, 138.4 (3C, aromatic), 128.4, 128.4, 128.1, 127.9, 127.6, 127.6, 127.0 (aromatic), 126.9 (1C, C-5/C-10), 126.8 (aromatic, overall 18C aromatic), 126.3 (1C, C-5/C-10), 111.6 (1C, C-6), 108.1 (1C, C-9), 104.9 (1C, C-1'), 85.3 (1C, C-3'), 82.1 (1C, C-2'), 77.6 (1C, C-5'), 77.5 (1C, C-1), 77.2 (1C, C-4'), 75.7, 74.6, 73.9 (3C, 3 x Ph-$\mathbf{CH_2}$), 70.5 (1C, C-3), 57.4 (1C, OCH_3, anomeric), 56.2, 55.8 (2C, 2 x OCH_3, aromatic), 36.4 (1C, C-4), 22.0 (1C, CH_3). MALDI-ToF HRMS: m\z calcd for $C_{39}H_{44}NaO_8^+$ $[M+Na]^+$ 663.2928, found 663.2934.

Compound **29**: R_f =0.22 (hexane: EtOAc 1:1). $[\alpha]_D$ -17.2 (c 0.18, $CHCl_3$). ^{1}H NMR (500 MHz, $CDCl_3$) δ (ppm): 7.39 – 7.23 (m, 15H, aromatic), 6.59 (s, 1H, H-9), 6.57 (s, 1H, H-6), 5.14 (d, *J* = 2.6 Hz, 1H, H-1), 4.95 (d, *J* = 10.8 Hz, 1H, Ph-**CH**), 4.93 (d, *J* = 10.2 Hz, 1H, Ph-**CH**), 4.91 – 4.83 (m, 3H, Ph-**CH**), 4.68 (d, *J* = 11.1 Hz, 1H, Ph-**CH**), 4.40 (ddd, *J* = 9.8, 6.3, 3.5 Hz, 1H, H-3), 4.03 (d, *J* = 7.8 Hz, 1H, H-1'), 3.93 (t, *J* = 9.4 Hz, 1H, H-4'), 3.85 (s, 3H, OCH_3, aromatic), 3.80 (s, 3H, OCH_3, aromatic), 3.73 (t, *J* = 9.1 Hz, 1H, H-3'), 3.63 (dd, *J* = 9.7, 2.7 Hz, 1H, H-5'), 3.43 (dd, *J* = 9.1, 7.8 Hz, 1H, H-2'), 3.08 (s, 3H, OCH_3, anomeric), 2.66 (dd, *J* = 15.8, 3.4 Hz, 1H, H-4a), 2.54 (dd, *J* = 15.8, 9.9 Hz, 1H, H-4b), 1.30 (d, *J* = 6.2 Hz, 3H, CH_3). ^{13}C NMR (126 MHz, $CDCl_3$) δ (ppm): 147.6, 147.4 (2C, C-7, C-8), 138.6, 138.6, 138.4, 128.5, 128.5, 128.4, 128.1, 128.1, 127.9, 127.7, 127.7 (18C, aromatic), 127.2, 125.9 (2C, C-5, C-10), 111.3 (1C, C-9), 107.0 (1C, C-6), 105.2 (1C, C-1'), 84.9 (1C, C-3'), 82.6 (1C, C-2'), 79.3 (1C, C-5'), 78.6 (1C, C-4'), 75.9, 75.5, 74.7 (3C, 3 x Ph-$\mathbf{CH_2}$), 71.4 (1C, C-1), 67.3 (1C, C-3), 56.4 (1C, OCH_3, anomeric), 56.1, 55.9 (2C, 2 x OCH_3, aromatic), 35.5 (1C, C-4), 21.6 (1C, CH_3). MALDI-ToF HRMS: m\z calcd for $C_{39}H_{44}NaO_8^+$ $[M+Na]^+$ 663.2928, found 663.2918.

17. (1*R*,3*R*)-1-(Methyl 2,3,4-tri-*O*-benzyl-β-D-xylopyranoside-5-yl)-7,8,9-trimethoxy-3-methylisochroman (30)

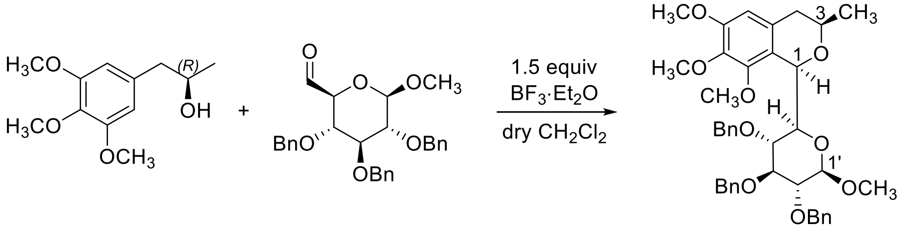

(*R*)-**12** **15** **30**

Alcohol (*R*)-**12** (50 mg, 0.2 mmol, 1 equiv.), dialdose **15** (125 mg, 0.24 mmol, 1.2 equiv.), dry CH_2Cl_2 (5 mL) and $BF_3 \cdot Et_2O$ (45 µL, 0.3 mmol, 1.5 equiv.) were used according to method B. Reaction time 1.5 h. The reaction mixture was quenched with saturated $NaHCO_3$ solution (1.5 mL)

and stirred for a further 5 min at room temperature. The crude product was purified by flash column chromatography on silica gel (hexane: EtOAc 5:1) to afford compound **30** (96 mg, 65%) as a colorless syrup. R_f = 0.39 (hexane: acetone 7:3). $[\alpha]_D$ -54.5 (c 0.11, $CHCl_3$). ^{1}H NMR (500 MHz, $CDCl_3$) δ (ppm): 7.35 (d, J = 6.9 Hz, 2H, aromatic), 7.32 – 7.16 (m, 8H, aromatic), 7.10 (m, 3H, aromatic), 6.67 (dd, J = 6.4, 2.6 Hz, 2H, aromatic), 6.11 (s, 1H, H-6), 5.18 (d, J = 1.1 Hz, 1H, H-1), 4.94 (d, J = 11.3 Hz, 1H, Ph-**CH**), 4.87 (d, J = 10.8 Hz, 1H, Ph-**CH**), 4.78 (d, J = 11.4 Hz, 1H, Ph-**CH**), 4.72 (d, J = 11.2 Hz, 1H, Ph-**CH**), 4.61 (d, J = 10.8 Hz, 1H, Ph-**CH**), 4.46 (d, J = 11.5 Hz, 1H, Ph-**CH**), 4.39 (d, J = 7.8, 4.4 Hz, 1H, H-1', overlap with H-5'), 4.38 (dd, J = 9.7, 1.1 Hz, 1H, H-5', overlap with H-1'), 4.03 (t, J = 9.2 Hz, 1H, H-4'), 3.89 (s, 3H, OCH_3, aromatic), 3.81 (s, 3H, OCH_3, aromatic), 3.71 (s, 4H, OCH_3, H-3), 3.66 (t, J = 9.0 Hz, 1H, H-3'), 3.62 (s, 3H, OCH_3, anomeric), 3.46 (dd, J = 9.1, 8.0 Hz, 1H, H-2'), 2.53 (dd, J = 15.4, 11.0 Hz, 1H, H-4a), 2.39 (d, J = 14.8 Hz, 1H, H-4b), 1.33 (d, J = 6.0 Hz, 3H, CH_3). ^{13}C NMR (126 MHz, $CDCl_3$) δ (ppm): 152.1, 150.2, 140.4 (3C, C-7 – C-9), 139.1, 138.8, 138.7 (aromatic), 130.4 (1C, C-5/C-10), 128.4, 128.3, 128.0, 127.9, 127.6, 127.5, 127.5, 126.6, 126.2 (aromatic, overall 18C aromatic), 120.7 (1C, C-5/C-10), 107.4 (1C, C-6), 105.1 (1C, C-1'), 85.9 (1C, C-3'), 82.3 (1C, C-2'), 77.0 (1C, C-4'), 76.1 (1C, C-5'), 75.7 (1C, C-1), 75.6, 74.5, 73.2 (3C, 3 x Ph-**CH_2**), 69.7 (1C, C-3), 60.8, 60.4, 57.2, 55.7 (4C, 4 x OCH_3), 37.3 (1C, C-4), 21.8 (1C, CH_3). MALDI-ToF HRMS: m\z calcd for $C_{40}H_{46}NaO_9^+$ $[M+Na]^+$ 693.3034, found 693.3041.

18. (1*R*,3*R*)-1-(Methyl 2,3,4-tri-*O*-benzyl-β-D-xylopyranoside-5-yl)-7,8-dibenzyloxy-3-methylisochroman (31) and **(1*S*,3*R*)-1-(methyl 2,3,4-tri-*O*-benzyl-β-D-xylopyranoside-5-yl)-7,8-dibenzyloxy-3-methylisochroman (32)**

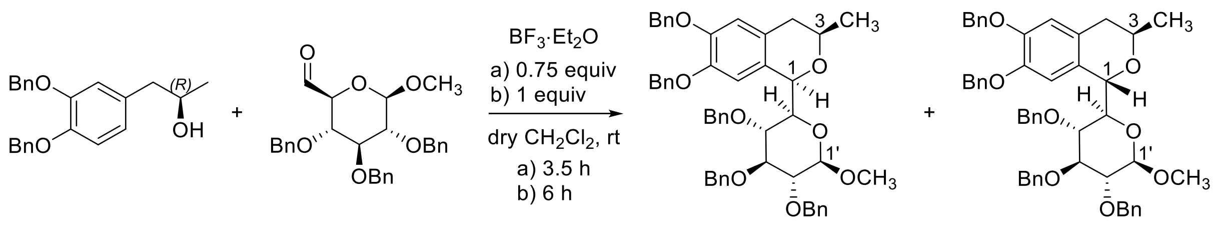

(*R*)-**13** **15** **31** **32**

a) Alcohol (*R*)-**13** (100 mg, 0.29 mmol, 1 equiv.), dialdose **15** (200 mg, 0.43 mmol, 1.5 equiv.), dry CH_2Cl_2 (10 mL) and $BF_3·Et_2O$ (27 µL, 0.22 mmol, 0.75 equiv.) were used according to method B. Reaction time: 4 h. The reaction mixture was quenched with saturated $NaHCO_3$ solution (1 mL) and stirred for a further 5 min at room temperature. The crude product was purified by flash column chromatography on silica gel (hexane: acetone 10:1) to afford **31** as a white solid (93 mg, 41%) and **32** as a white solid (54 mg, 24%).

b) Alcohol (*R*)-**13** (100 mg, 0.29 mmol, 1 equiv.), dialdose **15** (200 mg, 0.43 mmol, 1.5 equiv.), dry CH_2Cl_2 (10 mL) and $BF_3·Et_2O$ (36 µL, 0.29 mmol, 1 equiv.) were used according to method B. Reaction time: 6 h. The reaction mixture was quenched with saturated $NaHCO_3$ solution (1 mL) and stirred for a further 5 min at room temperature. The crude product was purified by flash column chromatography on silica gel (hexane: acetone 10:1) to afford **31** (155 mg, 69%) as a colorless syrup and **32** (15 mg, 7%) as a white amorphous.

Compound **31**: R_f = 0.13 (hexane: acetone 10:1). $[\alpha]_D$ -72.5 (c 0.12, $CHCl_3$). ^{1}H NMR (500 MHz, $CDCl_3$) δ (ppm): 7.47 – 7.39 (m, 4H, aromatic), 7.39 – 7.19 (m, 17H, aromatic), 7.11 – 7.06 (m, 2H, aromatic), 6.76 (s, 1H, H-9), 6.66 – 6.58 (m, 2H, aromatic), 6.48 (s, 1H, H-6), 5.11 (d, J = 12.1 Hz, 1H, Ph-**CH**), 5.07 (d, J = 12.1 Hz, 1H, Ph-**CH**), 5.02 (s, 1H, H-1, overlap with Ph-CH), 5.02 (d, J = 12.1 Hz, 1H, Ph-**CH**, overlap with H-1), 4.99 (d, J = 12.0 Hz, 1H, Ph-**CH**), 4.93 (d, J = 11.2 Hz, 1H, Ph-**CH**), 4.86 (d, J = 11.0 Hz, 1H, Ph-**CH**), 4.73 (d, J = 11.2 Hz, 1H, Ph-**CH**), 4.66 (d, J = 11.1 Hz, 1H, Ph-**CH**, overlap with Ph-CH), 4.65 (d, J = 11.0 Hz, 1H, Ph-**CH**, overlap with Ph-**CH**), 4.47 (d, J = 11.1 Hz, 1H, Ph-**CH**), 4.36 (d, J = 7.8 Hz, 1H, H-1'), 4.00 (t, J = 9.2 Hz, 1H, H-

4'), 3.81 (dd, J = 9.4, 1.3 Hz, 1H, H-5', overlap with H-3), 3.79 (ddd, J = 10.8, 5.9, 3.1 Hz, 1H, H-3, overlap with H-5'), 3.61 (s, 3H, OCH_3, overlap with H-3'), 3.60 (t, J = 9.1 Hz, 1H, H-3', overlap with OCH_3), 3.44 (dd, J = 9.1, 7.8 Hz, 1H, H-2'), 2.53 (dd, J = 15.5, 11.0 Hz, 1H, H-4a), 2.43 (dd, J = 15.8, 2.2 Hz, 1H, H-4b), 1.32 (d, J = 6.1 Hz, 3H, CH_3). ^{13}C NMR (126 MHz, $CDCl_3$) δ (ppm): 148.1, 147.2 (2C, C-7, C-8), 138.7, 138.7, 137.6, 137.4, 128.5, 128.5, 128.4, 128.3, 128.1, 127.8, 127.8, 127.8 (aromatic), 127.8, 127.6 (2C, C-5, C-10), 127.6, 127.6, 127.5, 127.3, 126.9 (aromatic, overall 30C aromatic), 114.9 (1C, C-6), 113.1 (1C, C-9), 105.0 (1C, C-1'), 85.2 (1C, C-3'), 82.1 (1C, C-2'), 77.6 (1C, C-5'), 77.4 (1C, C-1), 77.2 (1C, C-4'), 75.6, 74.6, 73.7, 72.2, 71.2 (5C, 5 x Ph-CH_2), 70.5 (1C, C-3), 57.3 (1C, OCH_3), 36.4 (1C, C-4), 21.9 (1C, CH_3). MALDI-ToF HRMS: m\z calcd for $C_{51}H_{52}NaO_8^+$ $[M+Na]^+$ 815.3554, found 815.3554.

Compound **32**: R_f = 0.20 (hexane: acetone 10:1). $[\alpha]_D$ -21.8 (c 0.11, $CHCl_3$). ^{1}H NMR (700 MHz, $CDCl_3$) δ (ppm): 7.42 (dd, J = 15.6, 7.4 Hz, 4H, aromatic), 7.35 – 7.25 (m, 21H, aromatic), 6.67 (s, 1H, H-9), 6.64 (s, 1H, H-6), 5.18 – 5.11 (m, 3H, Ph-**CH**), 5.08 (s, 1H, H-1), 5.04 (d, J = 12.4 Hz, 1H, Ph-**CH**), 4.94 (d, J = 10.8 Hz, 1H, Ph-**CH**), 4.90 (d, J = 10.3 Hz, 1H, Ph-**CH**), 4.87 – 4.82 (m, 3H, Ph-**CH**), 4.66 (d, J = 11.3 Hz, 1H, Ph-**CH**), 4.34 (ddd, J = 9.7, 6.4, 3.4 Hz, 1H, H-3), 3.86 (t, J = 9.4 Hz, 1H, H-4'), 3.81 (d, J = 7.8 Hz, 1H, H-1'), 3.67 (t, J = 9.0 Hz, 1H, H-3'), 3.45 (dd, J = 9.9, 2.2 Hz, 1H, H-5'), 3.37 (t, J = 8.4 Hz, 1H, H-2'), 2.94 (s, 3H, OCH_3), 2.58 (dd, J = 15.9, 2.4 Hz, 1H, H-4a), 2.48 (dd, J = 15.8, 10.2 Hz, 1H, H-4b), 1.26 (d, J = 5.9 Hz, 3H, CH_3). ^{13}C NMR (126 MHz, $CDCl_3$) δ (ppm): 147.5, 147.2 (2C, C-7, C-8), 138.7, 138.4, 137.5, 137.4 (5C, aromatic), 128.6, 128.6, 128.5, 128.4, 128.4 (aromatic), 128.2 (1C, C-5/C-10), 128.1, 127.9, 127.9, 127.8, 127.8, 127.7, 127.4 (aromatic, overall 25C aromatic), 126.9 (1C, C-5/C-10), 115.3 (1C, C-9), 111.2 (1C, C-6), 105.0 (1C, C-1'), 84.9 (1C, C-3'), 82.7 (1C, C-2'), 79.2 (1C, C-5'), 78.6 (1C, C-4'), 76.0, 75.5, 74.6 (4C, 4 x Ph-CH_2), 71.5 (1C, C-1), 71.4 (1C, Ph-CH_2), 67.2 (1C, C-3), 56.3 (1C, OCH_3), 35.4 (1C, C-4), 21.7 (1C, CH_3). MALDI-ToF HRMS: m\z calcd for $C_{51}H_{52}NaO_8^+$ $[M+Na]^+$ 815.3554, found 815.3563.

19. (1*R*,3*R*)-1-(Methyl α-D-xylopyranoside-5-yl)-7,8,9-trimethoxy-3-methylisochroman (33)

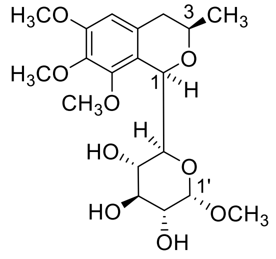

33

Compound **27** (60 mg, 0.09 mmol) was converted to **33** according to method C. The crude product was purified by flash column chromatography on silica gel (CH_2Cl_2: MeOH 9:1) to afford **33** (30 mg, 85%) as a white solid. R_f = 0.18 (CH_2Cl_2: MeOH 9:1). $[\alpha]_D$ +24 (c 0.1, MeOH). M.p: 82.4-85.7 °C. ^{1}H NMR (700 MHz, MeOD) δ (ppm): 6.48 (s, 1H, H-6), 5.16 (d, J = 1.6 Hz, 1H, H-1), 4.66 (d, J = 3.8 Hz, 1H, H-1'), 4.14 (dd, J = 9.9, 1.8 Hz, 1H, H-5'), 3.89 (s, 3H, OCH_3, aromatic), 3.81 (s, 3H, OCH_3, aromatic), 3.80 (s, 3H, OCH_3, aromatic) 3.67 (t, J = 9.3 Hz, 1H, H-4', overlap with H-3), 3.69 – 3.62 (m, 1H, H-3, overlap with H-4'), 3.55 (t, J = 9.3 Hz, 1H, H-3'), 3.45 (s, 3H, OCH_3, anomeric), 3.35 (dd, J = 9.6, 3.8 Hz, 1H, H-2'), 2.71 (ddt, J = 15.1, 10.7, 1.3 Hz, 1H, H-4a), 2.50 (dd, J = 15.0, 2.0 Hz, 1H, H-4b), 1.32 (d, J = 6.1 Hz, 3H, CH_3). ^{13}C NMR (126 MHz, MeOD) δ (ppm): 153.6, 151.1, 141.3 (3C, C-7 – C-9), 133.3, 120.9 (2C, C-5, C-10), 108.4 (1C, C-6), 101.2 (1C, C-1'), 75.6, 75.5 (2C, C-1, C-3'), 74.8 (1C, C-5'), 73.4 (1C, C-2'), 71.7, 71.1 (2C, C-3, C-4'), 61.0, 60.9, 56.4, 55.4 (4C, 4 x OCH_3), 38.3 (1C, C-4), 21.8 (1C, CH_3). MALDI-ToF HRMS: m\z calcd for $C_{19}H_{28}NaO_9^+$ $[M+Na]^+$ 423.1626, found 423.1621.

20. (1*R*,3*R*)-1-(Methyl β-D-xylopyranoside-5-yl)-7,8-dimethoxy-3-methylisochroman (34)

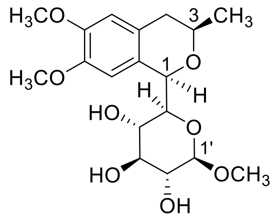

34

Compound **28** (65 mg, 0.10 mmol, 1 equiv.) was converted to **34** according to method C. The crude product was purified by flash column chromatography on silica gel (CH_2Cl_2: MeOH 12:1) to afford **34** (35 mg, 95%) as a white solid. R_f = 0.35 (CH_2Cl_2: MeOH 9:1). $[\alpha]_D$ -25.6 (c 0.09, MeOH). M.p: 148-151 °C. ^{1}H NMR (500 MHz, MeOD) δ (ppm): 6.90 (s, 1H, H-9), 6.67 (s, 1H, H-6), 5.03 (d, *J* = 3.8 Hz, 1H, H-1), 4.21 (d, *J* = 7.8 Hz, 1H, H-1'), 3.80 (s, 3H, OCH_3, aromatic), 3.80 (s, 3H, OCH_3, aromatic), 3.75 (ddd, *J* = 10.7, 6.1, 2.6 Hz, 1H, H-3), 3.63 (t, *J* = 9.2 Hz, 1H, H-4'), 3.56 (dd, *J* = 9.6, 3.9 Hz, 1H, H-5'), 3.53 (s, 3H, OCH_3, anomeric), 3.37 (t, *J* = 9.0 Hz, 1H, H-3'), 3.13 (dd, *J* = 9.3, 7.8 Hz, 1H, H-2'), 2.67 (dd, *J* = 15.3, 10.7 Hz, 1H, H-4a), 2.57 (dd, *J* = 15.3, 2.6 Hz, 1H, H-4b), 1.33 (d, *J* = 6.1 Hz, 3H, CH_3). ^{13}C NMR (126 MHz, MeOD) δ (ppm): 149.3, 148.7 (2C, C-7, C-8), 129.1, 127.7 (2C, C-5, C-10), 112.9 (1C, C-6), 110.9 (1C, C-9), 105.4 (1C, C-1'), 79.8 (1C, C-5'), 78.6 (1C, C-1), 77.9 (1C, C-3'), 74.8 (1C, C-2'), 72.9 (1C, C-4'), 72.0 (1C, C-3), 57.4, 56.7, 56.4 (3C, 3 x OCH_3), 37.4 (1C, C-4), 21.9 (1C, CH_3). MALDI-ToF HRMS: m\z calcd for $C_{18}H_{26}NaO_8^+$ $[M+Na]^+$ 393.1520, found 393.1516.

21. (1*S*,3*R*)-1-(Methyl β-D-xylopyranoside-5-yl)-7,8-dimethoxy-3-methylisochroman (35)

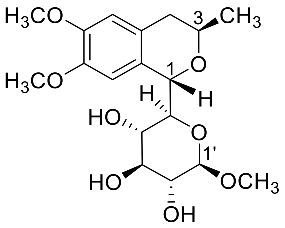

35

Compound **29** (60 mg, 0.093 mmol, 1 equiv.) was deprotected according to method C to afford compound **35** (33 mg, 96%) as a white amorphous. R_f = 0.50 (CH_2Cl_2: methanol 9:1). $[\alpha]_D$ -9.23 (c 0.13, MeOH). ^{1}H NMR (500 MHz, CD_3SOCD_3) δ (ppm): 6.71 (s, 1H, H-9), 6.65 (s, 1H, H-6), 5.23 (s, 2H, OH), 4.92 (s, 1H, H-1), 4.28 – 4.15 (m, 1H, H-3), 3.85 (d, *J* = 7.8 Hz, 1H, H-1'), 3.71 (s, 3H, OCH_3, aromatic), 3.71 (s, 3H, OCH_3, aromatic), 3.62 – 3.55 (m, 1H, H-5'), 3.50 (t, *J* = 9.1 Hz, 1H, H-4'), 3.24 (t, *J* = 8.8 Hz, 1H, H-3'), 2.99 – 2.95 (m, 1H, H-2', overlap with OCH_3), 2.95 (s, 3H, OCH_3, anomeric), 2.61 (dd, *J* = 15.9, 3.1 Hz, 1H, H-4a), 2.36 (dd, *J* = 15.8, 10.1 Hz, 1H, H-4b), 1.17 (d, *J* = 6.2 Hz, 3H, CH_3). ^{13}C NMR (126 MHz, CD_3SOCD_3) δ (ppm): 147.1, 147.0 (2C, C-7, C-8), 126.6, 126.4 (2C, C-5, C-10), 111.5 (1C, C-6), 107.9 (1C, C-9), 104.0 (1C, C-1'), 79.4 (1C, C-5'), 77.1 (1C, C-3'), 73.2 (1C, C-2'), 70.5 (1C, C-1), 69.6 (1C, C-4'), 66.5 (1C, C-3), 55.6, 55.3 (2C, 2 x OCH_3, aromatic), 54.8 (1C, OCH_3, anomeric), 35.1 (1C, C-4), 21.3 (1C, CH_3). MALDI-ToF HRMS: m\z calcd for $C_{18}H_{26}NaO_8^+$ $[M+Na]^+$ 393.1520, found 393.1525.

22. (1*R*,3*R*)-1-(Methyl β-D-xylopyranoside-5-yl)-7,8,9-trimethoxy-3-methylisochroman (36)

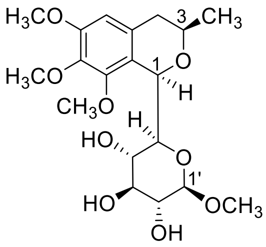

36

Compound **30** (88 mg, 0.13 mmol) was deprotected according to method C to afford **36** as a pale yellow solid (47 mg, 90%). R_f = 0.50 (CH_2Cl_2: methanol 9:1). $[\alpha]_D$ -74 (0.1, MeOH.). M.p: 58-60 °C. ^{1}H NMR (500 MHz, MeOD) δ (ppm): 6.50 (s, 1H, H-6), 5.23 (s, 1H, H-1), 4.14 (d, J = 7.9 Hz, 1H, H-1'), 3.89 (s, 3H, OCH_3, aromatic), 3.81 (s, 3H, OCH_3, aromatic), 3.80 (s, 3H, OCH_3, aromatic), 3.78 (dd, J = 9.8, 1.9 Hz, 1H, H-5'), 3.69 – 3.59 (m, 1H, H-3, overlap with H-4'), 3.62 (t, J = 9.3, 1H, H-4', overlap with H-3) 3.52 (s, 3H, OCH_3, anomeric), 3.29 (t, J = 9.1 Hz, 1H, H-3', overlap with MeOD signal), 3.11 (dd, J = 9.3, 7.8 Hz, 1H, H-2'), 2.72 (dd, J = 15.0, 10.8 Hz, 1H, H-4a), 2.51 (dd, J = 15.0, 1.8 Hz, 1H, H-4b), 1.31 (d, J = 6.0 Hz, 3H, CH_3). ^{13}C NMR (126 MHz, MeOD) δ (ppm): 153.71, 151.23, 141.29 (3C, C-7 – C-9), 133.58, 120.58 (2C, C-5, C-10), 108.47 (1C, C-6), 105.65 (1C, C-1'), 79.76 (1C, C-5'), 78.32 (1C, C-3'), 75.31 (1C, C-1), 74.89 (1C, C-2'), 71.70, 71.16 (2C, C-3, C-4'), 61.13, 61.12 (2C, 2 x OCH_3, aromatic), 57.29 (1C, OCH_3, anomeric), 56.45 (1C, OCH_3, aromatic), 38.24 (1C, C-4), 21.92 (1C, CH_3). MALDI-ToF HRMS: m\z calcd for $C_{19}H_{28}NaO_9^+$ $[M+Na]^+$ 423.1626, found 423.1627.

23. (1*R*,3*R*)-1-(Methyl β-D-xylopyranoside-5-yl)-7,8-diol-3-methylisochroman (37)

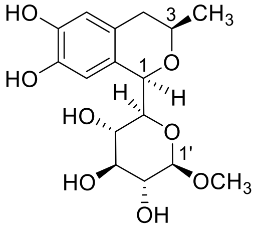

37

Compound **31** (129 mg, 0.16 mmol) was converted to **37** according to method C. The crude product was purified by flash column chromatography on silica gel (CH_2Cl_2: methanol 8:1) to afford compound **37** (45 mg, 82%) as a colorless syrup. R_f = 0.15 (CH_2Cl_2: methanol 7:1). $[\alpha]_D$ -110 (c 0.05, MeOH). ^{1}H NMR (500 MHz, MeOD) δ (ppm): 6.78 (s, 1H, H-9), 6.50 (s, 1H, H-6), 4.94 (d, J = 4.1 Hz, 1H, H-1), 4.17 (d, J = 7.8 Hz, 1H, H-1'), 3.72 (ddd, J = 10.8, 6.1, 2.5 Hz, 1H, H-3), 3.63 (t, J = 9.2 Hz, 1H, H-4'), 3.53 (s, 3H, OCH_3), 3.46 (dd, J = 9.6, 4.3 Hz, 1H, H-5'), 3.37 (t, J = 9.1 Hz, 1H, H-3'), 3.15 (dd, J = 9.1, 8.0 Hz, 1H, H-2'), 2.58 (dd, J = 15.1, 10.9 Hz, 1H, H-4a), 2.46 (dd, J = 15.2, 1.9 Hz, 1H, H-4b), 1.30 (d, J = 6.0 Hz, 3H, CH_3). ^{13}C NMR (126 MHz, MeOD) δ (ppm): 145.2, 144.4 (2C, C-7, C-8), 127.6, 126.6 (2C, C-5, C-10), 115.8 (1C, C-6), 113.7 (1C, C-9), 105.3 (1C, C-1'), 79.7 (1C, C-5'), 78.7 (1C, C-1), 77.9 (1C, C-3'), 74.7 (1C, C-2'), 73.2 (1C, C-4'), 72.1 (1C, C-3), 57.5 (1C, OCH_3), 37.2 (1C, C-4), 21.9 (1C, CH_3). MALDI-ToF-HRMS: m\z calcd for $C_{16}H_{22}NaO_8^+$ $[M+Na]^+$ 365.1207, found 365.1212.

Synthesis of tetra-*O*-benzyl-β-*C*-glucopyranosyl formaldehyde (38)

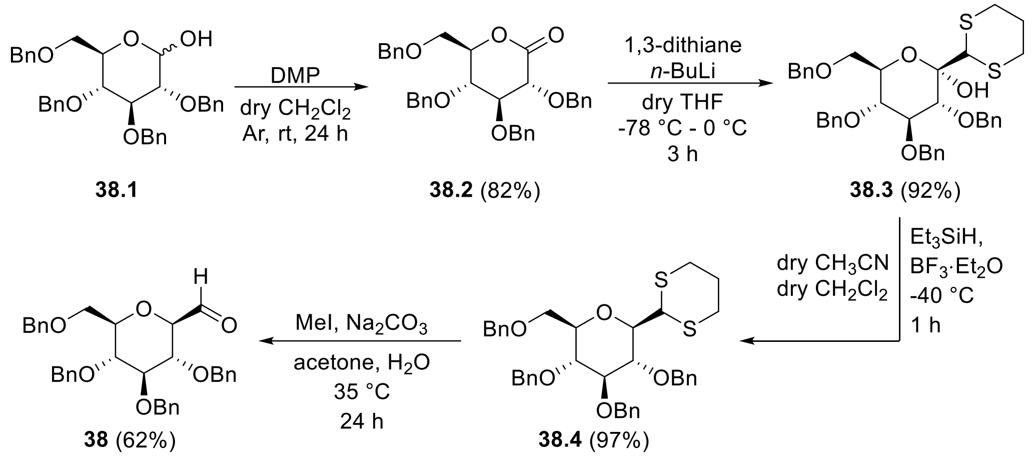

Scheme S5. Synthesis of β-*C*-glucopyranosyl formaldehyde **38**. (DMP: Dess-Martin periodinane)

2,3,4,6-Tetra-*O*-benzyl-D-glucono-1,5-lactone (38.2): 2,3,4,6-tetra-*O*-benzyl-D-glucopyranose **38.1** (5.4 g, 10.0 mmol, 1 equiv.) was dissolved in dry CH_2Cl_2 (200 mL), and Dess-Martin periodinane (DMP, 5.2 g, 12.0 mmol, 1.2 equiv.) was added at 0 °C, followed by stirring at room temperature for 24 h. The reaction mixture was monitored by TLC (hexane: EtOAc 7:3). The reaction mixture was diluted with CH_2Cl_2, and NaOH solution (1.3M, 46 mL) was added with continuous stirring for 10 min. The organic layer was extracted with water to a neutral pH. The organic phase was dried over Na_2SO_4, filtered, and concentrated under vacuum. The crude product was purified by flash column chromatography on silica gel (hexane: EtOAc 4:1) to afford the title compound as a white solid (4.50 g, 82%). R_f = 0.42 (hexane: EtOAc 7:3). ^{1}H NMR (500 MHz, $CDCl_3$) δ (ppm): 7.40 – 7.35 (m, 2H, aromatic), 7.35 – 7.21 (m, 16H, aromatic), 7.19 – 7.14 (m, 2H, aromatic), 4.97 (d, J = 11.4 Hz, 1H, Ph-**CH**), 4.72 (d, J = 11.8 Hz, 1H, Ph-**CH**), 4.70 (d, J = 12.2 Hz, 1H, Ph-**CH**), 4.62 (d, J = 11.4 Hz, 1H, Ph-**CH**), 4.58 (d, J = 11.4 Hz, 1H, Ph-**CH**), 4.54 (d, J = 12.0 Hz, 1H, Ph-**CH**), 4.50 (d, J = 11.2 Hz, 1H, Ph-**CH**), 4.48 – 4.42 (m, 2H, Ph-**CH**, H-5), 4.12 (d, J = 6.5 Hz, 1H, H-2), 3.94 (dd, J = 8.4, 6.7 Hz, 1H, H-4), 3.90 (t, J = 6.7 Hz, 1H, H-3), 3.71 (dd, J = 11.0, 2.4 Hz, 1H, H-6a), 3.65 (dd, J = 11.0, 3.3 Hz, 1H, H-6b). ^{13}C NMR (126 MHz, $CDCl_3$) δ (ppm): 169.4 (C-1), 137.7, 137.6, 137.6, 137.0 (4C, aromatic), 128.5, 128.4, 128.2, 128.0, 128.0, 127.9 (20C, aromatic), 81.0, 78.2, 77.4, 76.1 (4C, C-2 – C-5), 73.9, 73.8, 73.7, 73.6 (4C, 4 x Ph-**C**H_2), 68.31 (1C, C-6). MALDI-ToF MS: m\z calcd for $C_{34}H_{34}NaO_6^+$ [M+Na]$^+$ 561.225, found 561.320.

2,3,4,6-Tetra-*O*-benzyl-1-*C*-(2'-dithianyl)-α-D-glucopyranose (38.3) [10]: To a stirred solution of 1,3-dithiane (1.33 g, 11.1 mmol, 1.5 equiv.) in dry THF (45 mL), BuLi solution (4.44 mL, 2.5M in hexane, 11.1 mmol, 1.5 mol) was added at -78 °C under argon atmosphere. The mixture was stirred at -78 °C for 15 min, after that, the solution of 2,3,4,6-tetra-*O*-benzyl-D-glucono-1,5-lactone **38.2** (3.98 g, 7.4 mmol, 1 equiv.) in dry THF (30 mL) was added at -78 °C. The mixture was stirred for 2 h at 0 °C. The progress of the reaction was monitored by TLC (hexane: acetone 3:1). After complete conversion, saturated NH_4Cl solution (11 mL) and Et_2O (15 mL) were added to the reaction mixture, and the mixture was allowed to warm up to room temperature. The aqueous phase was separated, extracted with Et_2O (3 x 100 mL), the combined organic phases were washed with water (1 x 100 mL) and brine (1 x 100 mL). The organic layer was dried over Na_2SO_4, filtered, and concentrated under vacuum. The crude product was purified by flash column chromatography on silica gel (hexane: acetone 8:1) to afford the title compound as a colorless syrup (4.50 g, 92%). R_f = 0.33 (hexane: acetone 3:1). ^{1}H NMR (500 MHz, $CDCl_3$) δ (ppm): 7.36 – 7.22 (m, 20H, aromatic),

4.92 (d, J = 10.8 Hz, 3H, 3 x Ph-**CH**), 4.88 (d, J = 11.0 Hz, 1H, Ph-**CH**), 4.67 (d, J = 11.3 Hz, 1H, Ph-**CH**), 4.60 (d, J = 11.0 Hz, 1H, Ph-**CH**), 4.48 (s, 2H), 4.31 (d, J = 9.6 Hz, 1H), 4.10 (t, J = 9.2 Hz, 1H), 4.06 (ddd, J = 10.1, 4.6, 2.1 Hz, 1H), 3.70 – 3.65 (m, 2H), 3.65 – 3.60 (m, 2H), 3.41 (td, J = 13.0, 2.7 Hz, 1H), 3.35 (td, J = 13.0, 2.5 Hz, 1H), 2.76 (tdd, J = 14.3, 9.5, 2.8 Hz, 1H), 2.34 – 2.21 (m, 1H), 2.00 (ddq, J = 9.2, 6.1, 3.3 Hz, 1H), 1.92 (tt, J = 13.7, 3.1 Hz, 1H). ^{13}C NMR (126 MHz, $CDCl_3$) δ (ppm): 138.8, 138.4, 138.1, 128.6, 128.6, 128.5, 128.5, 128.1, 128.0, 127.9, 127.9, 127.9, 127.8, 127.7, 102.2, 84.5, 78.8, 78.1, 75.6, 75.5, 75.2, 73.4, 71.3, 69.5, 44.4, 25.8, 25.3, 24.9. MALDI-ToF MS: m\z calcd for $C_{38}H_{42}NaO_6S_2{}^+$[M+Na]$^+$ 681.232, found 681.315.

2-(2',3',4',6'-Tetra-*O*-benzyl-1'-*C*-β-D-glucopyranosyl)dithiane (38.4) [10]: 2,3,4,6-tetra-*O*-benzyl-1-*C*-(2'-dithianyl)-α-D-glucopyranose **38.3** (3.20 g, 4.90 mmol, 1 equiv.) was dissolved in a dry acetonitrile: CH_2Cl_2 (1:1, 70 mL) solvent mixture, and cooled down to -40 °C. Et_3SiH (3.88 mL, 24.3 mmol, 5 equiv.) was added to the solution under argon atmosphere, and it was stirred at -40 °C for 1 hour. After that $BF_3{\cdot}Et_2O$ (1.78 mL, 14.6 mmol, 3 equiv.) was added dropwise to the reaction mixture, and it was stirred for 1 hour at -40 °C. The progress of the reaction was monitored by TLC (hexane: acetone 3:1). After complete conversion (1+1 hour), Et_3N (2 mL) was added to the reaction mixture, and it was allowed to warm up to room temperature. The solution was concentrated under vacuum, the residue was dissolved in CH_2Cl_2 (100 mL), and it was washed with water to neutral pH (1 x 50 mL). The organic phase was extracted with brine (1 x 50 mL) then it was dried over Na_2SO_4, filtered, and concentrated under vacuum. The crude product was purified by flash column chromatography on silica gel (hexane: acetone 10:1) to afford the title compound as a yellowish syrup (3.00 g, 97%). R_f = 0.39 (hexane: acetone 3:1). MALDI-ToF MS: m\z calcd for $C_{38}H_{42}NaO_5S_2{}^+$[M+Na]$^+$ 665.237 found 665.277.

2,3,4,6-Tetra-*O*-benzyl-1-*C*-β-D-glucopyranosyl formaldehyde (38) [10]: To a solution of 2-(2',3',4',6'-tetra-*O*-benzyl-1'-*C*-β-D-glucopyranosyl)dithiane **38.4** (1.25 g, 1.95 mmol, 1 equiv.) in aceton: water solvent mixture (9:1, 25 mL) Na_2CO_3 (248 mg, 2.3 mmol, 1.2 equiv.) and CH_3I (491 μl, 7.8 mmol, 4 equiv.) were added. The reaction was stirred at 35 °C for overnight. The solvent was evaporated under reduced pressure, the residue was dissolved in EtOAc (50 mL), and it was dried over Na_2SO_4, filtered, and concentrated under vacuum. The crude product was purified by flash column chromatography on silica gel (hexane: CH_2Cl_2: acetone 5:1:1) to afford the title compound as a colorless syrup (664 mg, 62%) beside the starting material **38.4** (174 mg, conversion: 86%). R_f = 0.16 (hexane: CH_2Cl_2: acetone 5:1:1).

Synthesis of isochroman-sugar hybrids from tetra-*O*-benzyl-β-*C*-glucopyranosyl-formaldehyde

1. (1*S*,3*S*)-1-(2',3',4',6'-Tetra-*O*-benzyl-1'-*C*-β-D-glucopyranosyl)-7,8-dimethoxy-3-methylisochroman (39) and (1*R*,3*S*)-1-(2',3',4',6'-tetra-*O*-benzyl-1'-*C*-β-D-glucopyranosyl)-7,8-dimethoxy-3-methylisochroman (40)

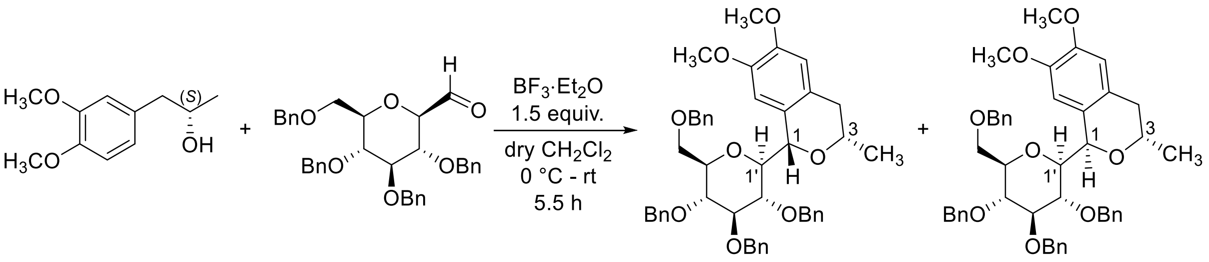

Alcohol (*S*)-**1** (50 mg, 0.26 mmol, 1 equiv.), glucopyranosyl formaldehyde **38** (211 mg, 0.38 mmol, 1.5 equiv.), dry CH_2Cl_2 (8.7 mL), and $BF_3{\cdot}Et_2O$ (47 µL, 0.38 mmol, 1.5 equiv.) were used according to method B. Reaction time: 5.5 h. The reaction mixture was quenched with saturated $NaHCO_3$ solution (2 mL) and stirred for a further 5 min at room temperature. The crude product was purified by flash column chromatography on silica gel (hexane: EtOAc 4:1) to afford compound **39** (140 mg, 75%) as a colorless syrup and compound **40** (15 mg, 8%) as a colorless syrup.

Compound **39**: R_f = 0.33 (hexane: EtOAc 2:1). $[\alpha]_D$ +80.0 (c 0.07, $CHCl_3$). ^{1}H NMR (500 MHz, $CDCl_3$) δ (ppm): 7.38 – 7.32 (m, 4H, aromatic), 7.31 – 7.22 (m, 9H, aromatic), 7.19 – 7.12 (m, 2H, aromatic), 7.12 – 7.05 (m, 3H, aromatic), 6.73 (s, 1H, H-9), 6.58 – 6.53 (m, 2H, aromatic), 6.38 (s, 1H, H-6), 5.10 (s, 1H, H-1), 4.83 (d, *J* = 11.1 Hz, 1H, Ph-**CH**), 4.82 (d, *J* = 10.9 Hz, 1H, Ph-**CH**), 4.75 (d, *J* = 11.0 Hz, 1H, Ph-**CH**), 4.68 (d, *J* = 10.9 Hz, 1H, Ph-**CH**), 4.62 – 4.53 (m, 4H, 4 x Ph-**CH**), 4.08 (t, *J* = 9.2 Hz, 1H, H-2'), 3.96 (dd, *J* = 9.4, 1.4 Hz, 1H, H-1'), 3.89 – 3.86 (m, 1H, H-3, overlap with OCH_3), 3.85 (s, 3H, OCH_3, overlap with H-3), 3.81 – 3.76 (m, 4H, H-6'a, OCH_3), 3.74 (t, *J* = 9.0 Hz, 1H, H-3'), 3.71 (dd, *J* = 6.7, 3.9 Hz, 1H, H-6'b), 3.63 – 3.59 (m, 1H, H-5', overlap with H-4'), 3.59 – 3.53 (m, 1H, H-4', overlap with H-5'), 2.60 (dd, *J* = 15.4, 11.4 Hz, 1H, H-4a), 2.48 (dd, *J* = 15.6, 1.9 Hz, 1H, H-4b), 1.36 (d, *J* = 6.1 Hz, 3H, CH_3). ^{13}C NMR (126 MHz, $CDCl_3$) δ (ppm): 147.73, 147.69 (2C, C-7, C-8), 138.82, 138.50, 138.22 (4C, aromatic), 128.51, 128.46, 127.92, 127.86, 127.79, 127.65, 127.60 (aromatic), 127.36 (1C, C-5/C-10), 127.05, 126.87 (aromatic, altogether 24C aromatic), 126.02 (1C, C-5/C-10), 111.69 (1C, C-6), 108.35 (1C, C-9), 87.94 (1C, C-3'), 81.75 (1C, C-1'), 79.24 (1C, C-5'), 78.44 (1C, C-4'), 78.07 (1C, C-1), 77.81 (1C, C-2'), 75.62 , 75.06, 73.99, 73.63 (4C, 4 x Ph-CH_2), 70.71 (1C, C-3), 69.46 (1C, C-6'), 56.32, 55.91 (2C, 2 x OCH_3), 36.47 (1C, C-4), 22.00 (1C, CH_3). MALDI-ToF HRMS: m/z calcd for $C_{46}H_{50}NaO_8^+$ [M+Na]$^+$ 753.3398, found 753.3380.

Compound **40**: R_f = 0.41 (hexane: EtOAc = 2:1). $[\alpha]_D$ +32.8 (c 0.07, $CHCl_3$). ^{1}H NMR (500 MHz, $CDCl_3$) δ (ppm): 7.38 – 7.23 (m, 18H, aromatic), 7.11 – 7.05 (m, 2H, aromatic), 6.60 (s, 1H, H-9), 6.55 (s, 1H, H-6), 5.11 (d, *J* = 2.3 Hz, 1H, H-1), 4.99 (d, *J* = 10.9 Hz, 1H, Ph-**CH**), 4.95 (d, *J* = 10.3 Hz, 1H, Ph-**CH**), 4.94 (d, *J* = 10.8 Hz, 1H, Ph-**CH**), 4.90 (d, *J* = 10.4 Hz, 1H, Ph-**CH**), 4.86 (d, *J* = 10.9 Hz, 1H, Ph-**CH**), 4.62 (d, *J* = 11.1 Hz, 1H, Ph-**CH**), 4.50 (ddd, *J* = 9.9, 6.2, 3.6 Hz, 1H, H-3), 4.25 (d, *J* = 12.0 Hz, 1H, Ph-**CH**), 4.21 (d, *J* = 12.0 Hz, 1H, Ph-**CH**), 3.95 (t, *J* = 9.4 Hz, 1H, H-2'), 3.88 – 3.81 (m, 1H, H-3', overlap with OCH_3), 3.80 (s, 3H, OCH_3, overlap with H-3'), 3.79 (s, 3H, OCH_3), 3.69 (dd, *J* = 9.6, 2.4 Hz, 1H, H-1'), 3.62 (dd, *J* = 11.3, 1.8 Hz, 1H, H-6'a), 3.60 (t, *J* = 9.4 Hz, 1H, H-4'), 3.53 (dd, *J* = 11.6, 4.8 Hz, 1H, H-6'b), 3.27 (ddd, *J* = 9.8, 4.9, 1.8 Hz, 1H, H-5'), 2.62 (dd, *J* = 15.9, 3.5 Hz, 1H, H-4a), 2.54 (dd, *J* = 15.8, 9.8 Hz, 1H, H-4b), 1.28 (d, *J* = 6.1 Hz, 3H, CH_3). ^{13}C NMR (126 MHz, $CDCl_3$) δ (ppm): 147.7, 147.7 (2C, C-7, C-8), 138.7, 138.5, 138.4 (4C, aromatic), 128.7, 128.6, 128.5, 128.3, 128.0, 128.0, 127.9, 127.5 (20C, aromatic), 127.1, 126.3 (2C, C-5, C-10), 111.4 (1C, C-6), 107.4 (1C, C-9), 87.4 (1C, C-3'), 84.0 (1C, C-1'),

80.6 (1C, C-5’), 79.1 (1C, C-2’), 78.9 (1C, C-4’), 75.9, 75.6, 75.2, 73.3 (4C, 4 x Ph-**C**H_2), 71.7 (1C, C-1), 69.3 (1C, C-6’), 67.4 (1C, C-3), 56.2, 55.9 (2C, 2 x OCH_3), 35.7 (1C, C-4), 21.8 (1C, CH_3). MALDI-ToF HRMS: m/z calcd for $C_{46}H_{50}NaO_8^+$ $[M+Na]^+$ 753.3398, found 753.3388.

2. (1*S*,3*S*)-1-(2’,3’,4’,6’-Tetra-*O*-benzyl-1’-*C*-β-D-glucopyranosyl)-7,8-dibenzyloxy-3-methylisochroman (41) and (1*R*,3*S*)-1-(2’,3’,4’,6’-tetra-*O*-benzyl-1’-*C*-β-D-glucopyranosyl)-7,8-dibenzyloxy-3-methylisochroman (42)

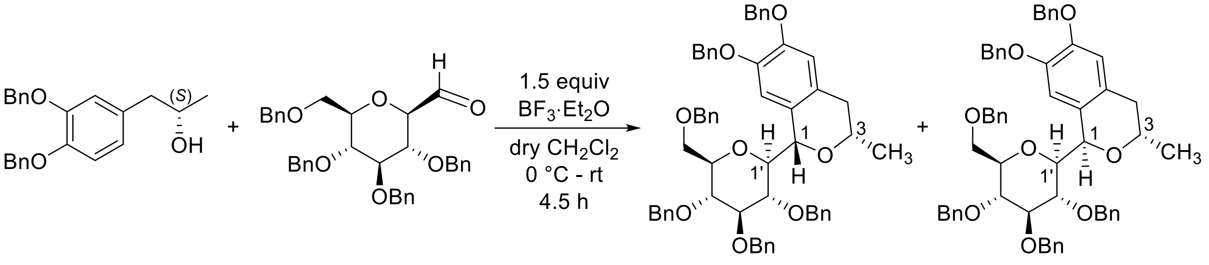

(*S*)-**13** **38** **41** **42**

Alcohol (*S*)-**13** (45 mg, 0.13 mmol, 1 equiv.), glucopyranosyl formaldehyde **38** (107 mg, 0.19 mmol, 1.5 equiv.), dry CH_2Cl_2 (4.5 mL), and $BF_3 \cdot Et_2O$ (24 µL, 0.19 mmol, 1.5 equiv.) were used according to method B. Reaction time: 4.5 h. The reaction mixture was quenched with saturated $NaHCO_3$ solution (2 mL) and stirred for a further 5 min at room temperature. The crude product was purified by flash column chromatography on silica gel (hexane: EtOAc 5.5:1) to afford compound **41** (80 mg, 70%) as a colorless foam and compound **42** (4.9 mg, 4%) as a colorless foam.

Compound **41**: R_f = 0.27 (hexane: EtOAc 4:1). $[\alpha]_D$ +26.1 (c 0.18, $CHCl_3$). ^1H NMR (500 MHz, $CDCl_3$) δ (ppm): 7.46 – 7.40 (m, 4H, aromatic), 7.38 – 7.29 (m, 9H, aromatic), 7.31 – 7.21 (m, 10H, aromatic), 7.19 – 7.13 (m, 2H, aromatic), 7.10 – 7.05 (m, 3H, aromatic), 6.80 (s, 1H, H-9), 6.60 (m, 2H, aromatic), 6.46 (s, 1H, H-6), 5.10 (d, *J* = 12.3 Hz, 1H, Ph-**CH**$_{(isochroman)}$), 5.10 (d, *J* = 12.4 Hz, 1H, Ph-**CH**$_{(isochroman)}$), 5.04 (s, 1H, H-1), 5.00 (d, *J* = 12.1 Hz, 1H, Ph-**CH**$_{(isochroman)}$), 4.97 (d, *J* = 12.1 Hz, 1H, Ph-**CH**$_{(isochroman)}$), 4.82 (d, *J* = 11.1 Hz, 1H, Ph-**CH**$_{(sugar)}$), 4.82 (d, *J* = 10.6 Hz, 1H, Ph-**CH**$_{(sugar)}$), 4.74 (d, *J* = 11.1 Hz, 1H, Ph-**CH**$_{(sugar)}$), 4.65 (d, *J* = 11.0 Hz, 1H, Ph-**CH**$_{(sugar)}$), 4.60 – 4.54 (m, 3H, 3 x Ph-**CH**$_{(sugar)}$), 4.51 (d, *J* = 11.1 Hz, 1H, Ph-**CH**$_{(sugar)}$), 3.99 (t, *J* = 9.2 Hz, 1H, H-2’), 3.82 (dd, *J* = 9.5, 1.4 Hz, 1H, H-1’, overlap with H-3), 3.84 – 3.78 (m, 1H, H-3, overlap with H-1’), 3.77 (dd, *J* = 10.8, 1.3 Hz, 1H, H-6’a), 3.71 (dd, *J* = 10.8, 4.6 Hz, 1H, H-6’b), 3.65 (t, *J* = 9.0 Hz, 1H, H-3’), 3.58 – 3.53 (m, 2H, H-4’, H-5’), 2.54 (dd, *J* = 15.4, 10.9 Hz, 1H, H-4a), 2.43 (dd, *J* = 16.0, 1.8 Hz, 1H, H-4b), 1.33 (d, *J* = 6.1 Hz, 3H, CH_3). ^{13}C NMR (126 MHz, $CDCl_3$) δ (ppm): 148.0 (1C, C-8), 147.2 (1C, C-7), 138.8, 138.7, 138.3, 138.3, 137.6, 137.6 (6C, aromatic), 128.6, 128.5, 128.5, 128.4, 128.1, 128.1, 127.9, 127.8, 127.7, 127.7, 127.6, 127.3, 127.1, 126.9, 126.8 (32C, aromatic, C-5, C-10), 114.9 (1C, C-6), 112.8 (1C, C-9), 87.9 (1C, C-3’), 81.7 (1C, C-1’), 79.3 (1C, C-5’), 78.4 (1C, C-4’), 77.9 (1C, C-1), 77.8 (1C, C-2’), 75.6, 75.0, 73.8, 73.6 (4C, 4 x Ph-**CH**$_{2(sugar)}$), 71.9, 71.3 (2C, 2 x Ph-**CH**$_{2(isochroman)}$), 70.6 (1C, C-3), 69.4 (1C, C-6’), 36.4 (1C, C-4), 22.0 (1C, CH_3). MADI-ToF HRMS: m/z calcd for $C_{58}H_{58}NaO_8^+$ $[M+Na]^+$ 905.4024, found 905.4034.

Compound **42**: R_f = 0.39 (hexane: EtOAc 4:1). $[\alpha]_D$ +15.5 (c 0.20, $CHCl_3$). ^1H NMR (700 MHz, $CDCl_3$) δ (ppm): 7.42 (d, *J* = 7.6 Hz, 2H, aromatic), 7.40 – 7.22 (m, 26H, aromatic), 7.08 (d, *J* = 6.9 Hz, 2H, aromatic), 6.67 (s, 1H, H-9), 6.66 (s, 1H, H-6), 5.12 (d, *J* = 12.3 Hz, 1H, Ph-**CH**$_{(isochroman)}$), 5.09 – 5.02 (m, 4H, 3 x Ph-**CH**$_{(isochroman)}$, H-1), 4.98 (d, *J* = 10.8 Hz, 1H, Ph-**CH**$_{(sugar)}$), 4.93 (d, *J* = 10.6 Hz, 1H, Ph-**CH**$_{(sugar)}$), 4.91 (d, *J* = 10.3 Hz, 1H, Ph-**CH**$_{(sugar)}$), 4.87 (d, *J* = 10.4 Hz, 1H, Ph-**CH**$_{(sugar)}$), 4.84 (d, *J* = 11.0 Hz, 1H, Ph-**CH**$_{(sugar)}$), 4.62 (d, *J* = 10.9 Hz, 1H, Ph-**CH**), 4.41 (ddd, *J* = 9.7, 6.2, 3.6 Hz, 1H, H-3), 4.22 (d, *J* = 12.1 Hz, 1H, Ph-**CH**$_{(sugar)}$), 4.19 (d, *J* = 12.0 Hz, 1H, Ph-**CH**$_{(sugar)}$), 3.89 (t, *J* = 9.4 Hz, 1H, H-2’), 3.76 (t, *J* = 9.1 Hz, 1H, H-3’), 3.57 (t, *J* = 9.4 Hz,

1H, H-4'), 3.54 (dd, J = 9.6, 2.4 Hz, 1H, H-1'), 3.53 – 3.47 (m, 2H, H-6'a, H-6'b), 3.10 (ddd, J = 9.8, 3.9, 2.3 Hz, 1H, H-5'), 2.55 (dd, J = 15.9, 3.4 Hz, 1H, H-4a), 2.49 (dd, J = 15.8, 9.9 Hz, 1H, H-4b), 1.25 (d, J = 6.5 Hz, 3H, CH_3). ^{13}C NMR (176 MHz, $CDCl_3$) δ (ppm): 147.9 (1C, C-8), 147.2 (1C, C-7), 138.8, 138.8, 138.5, 138.4, 137.6, 137.5 (6C, aromatic), 128.6, 128.6, 128.6, 128.6, 128.5, 128.3 (aromatic), 128.1 (1C, C-10), 128.0, 128.0, 128.0, 127.9, 127.9, 127.8, 127.5, 127.4, 127.4 (aromatic, altogether 30C aromatic), 127.2 (1C, C-5), 115.0 (1C, C-6), 111.8 (1C, C-9), 87.4 (1C, C-3'), 83.8 (1C, C-1'), 80.5 (1C, C-5'), 79.1 (1C, C-2'), 78.8 (1C, C-4'), 75.9, 75.6, 75.1, 73.2 (4C, 4 x Ph-**CH$_{2}$**$_{(sugar)}$), 71.8 (1C, C-1), 71.6, 71.4 (2C, 2 x Ph-**CH$_{2}$**$_{(isochroman)}$), 69.0 (1C, C-6'), 67.3 (1C, C-3), 35.7 (1C, C-4), 21.7 (1C, CH_3). MALDI-ToF HRMS: m/z calcd for $C_{58}H_{58}NaO_8^+$ $[M+Na]^+$ 905.4024, found 905.4020.

3. (1*R*,3*R*)-1-(2',3',4',6'-Tetra-*O*-benzyl-1'-*C*-β-D-glucopyranosyl)-3-methyl-7,8-dimetyoxyisochroman (43) and (1*S*,3*R*)-1-(2',3',4',6'-tetra-*O*-benzyl-1'-*C*-β-D-glucopyranosyl)-3-methyl-7,8-dimethoxyisochroman (44)

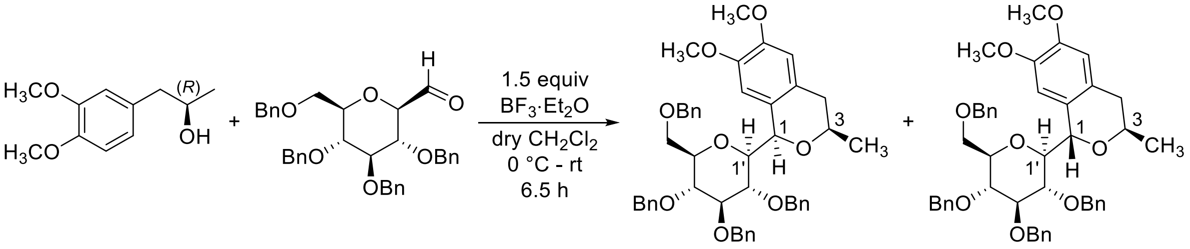

Alcohol (*R*)-**1** (51 mg, 0.26 mmol, 1 equiv.), glucopyranosyl formaldehyde **38** (211 mg, 0.38 mmol, 1.5 equiv.), dry CH_2Cl_2 (8.7 mL) and $BF_3 \cdot Et_2O$ (47 µL, 0.38 mmol, 1.5 equiv.) were used according to method B. Reaction time: 6.5 h. The reaction mixture was quenched with saturated $NaHCO_3$ solution (2 mL) and stirred for a further 5 min at room temperature. The crude product was purified by flash column chromatography on silica gel (hexane: EtOAc 5.5:1) to afford compound **43** (126 mg, 67%) as a white foam and compound **44** (11 mg, 6%) as a colorless syrup.

Compound **43**: R_f = 0.24 (hexane: EtOAc 3:1). $[\alpha]_D$ -32.0 (c 0.050, $CHCl_3$). ^{1}H NMR (500 MHz, $CDCl_3$) δ (ppm): 7.36 (m, 2H, aromatic), 7.35 – 7.28 (m, 6H, aromatic), 7.30 – 7.19 (m, 10H, aromatic), 7.14 – 7.09 (m, 2H, aromatic), 6.68 (s, 1H, H-9), 6.54 (s, 1H, H-6), 5.11 (s, 1H, H-1), 4.97 (d, J = 11.1 Hz, 1H, Ph-**CH**), 4.92 (d, J = 11.2 Hz, 1H, **Ph-CH**), 4.88 (d, J = 10.7 Hz, 1H, **Ph-CH**), 4.84 (d, J = 11.0 Hz, 1H, Ph-**CH**), 4.73 (d, J = 10.6 Hz, 1H, **Ph-CH**), 4.60 (d, J = 10.9 Hz, 1H, **Ph-CH**), 4.35 (d, J = 12.0 Hz, 1H, Ph-**CH**), 4.28 (d, J = 11.9 Hz, 1H, **Ph-CH**), 4.04 (t, J = 9.3 Hz, 1H, H-2'), 3.79 (m, 6H, OCH_3, H-1', H-3', H-3), 3.73 (s, 3H, OCH_3), 3.64 (dd, J = 11.5, 1.5 Hz, 1H, H-6'a), 3.58 (t, J = 9.6 Hz, 1H, H-4'), 3.56 (dd, J = 7.8, 3.7 Hz, 1H, H-6'b), 3.41 (dd, J = 9.8, 3.3 Hz, 1H, H-5'), 2.69 (dd, J = 15.3, 11.1 Hz, 1H, H-4a), 2.53 (dd, J = 15.6, 2.0 Hz, 1H, H-4b), 1.35 (d, J = 6.1 Hz, 3H, CH_3). ^{13}C NMR (126 MHz, $CDCl_3$) δ (ppm): 147.6, 147.6 (2C, C-7, C-8), 138.8, 138.6, 138.4, 138.2 (4C, aromatic), 128.5, 128.5, 128.4, 128.2, 128.1, 127.9, 127.9, 127.8, 127.8, 127.7, 127.6 (19C, aromatic), 127.5 (1C, C-10), 127.4 (1C, aromatic), 126.9 (1C, C-5), 111.3 (1C, C-6), 107.3 (1C, C-9), 87.5 (1C, C-3'), 80.6 (1C, C-1'), 80.5 (1C, C-5'), 78.6 (1C, C-4'), 78.2 (1C, C-2'), 75.6, 75.2, 75.0 (3C, 3 x Ph-**C**H_2), 74.5 (1C, C-1), 73.2 (1C, Ph-**C**H_2), 71.0 (1C, C-3), 68.6 (1C, C-6'), 55.9, 55.8 (2C, 2 x OCH_3), 36.0 (1C, C-4), 21.8 (1C, CH_3). MALDI-ToF HRMS m/z: calcd for $C_{46}H_{50}NaO_8^+$ $[M+Na]^+$ 753.3398, found 753.3403.

Compound **44**: R_f = 0.40 (hexane: EtOAc 3:1). $[\alpha]_D$ +22.5 (c 0.040, $CHCl_3$). ^{1}H NMR (700 MHz, $CDCl_3$) δ (ppm): 7.40 – 7.35 (m, 4H, aromatic), 7.34 – 7.31 (m, 4H, aromatic), 7.31 – 7.20 (m, 10H, aromatic), 7.12 (m, 2H, aromatic), 6.74 (d, J = 8.1 Hz, 1H, H-6), 6.69 (d, J = 8.1 Hz, 1H, H-9), 5.35 (s, 1H, H-1), 4.99 – 4.89 (m, 4H, 4xPh-**CH**), 4.84 (d, J = 11.0 Hz, 1H, Ph-**CH**), 4.61 (d, J = 10.9 Hz, 1H, Ph-**CH**), 4.28 (d, J = 12.0 Hz, 1H, Ph-**CH**), 4.18 (d, J = 12.1 Hz, 1H, Ph-**CH**), 4.09 (t, J = 9.2 Hz, 1H, H-2', overlap with H-1'), 4.06 (dd, J = 9.4, 1.3 Hz, 1H, H-1', overlap with H-

2’), 3.80 (s, 3H, OCH_3), 3.80 – 3.75 (m, 4H, H-3’, OCH_3), 3.69 – 3.63 (m, 1H, H-3), 3.60 (d, *J* = 11.1 Hz, 1H, H-6’a), 3.55 – 3.49 (m, 2H, H-4’, H-6’b), 3.35 (dd, *J* = 9.2, 4.4 Hz, 1H, H-5’), 2.65 (dd, *J* = 14.3, 11.3 Hz, 1H, H-4a), 2.48 (d, *J* = 14.4 Hz, 1H, H-4b), 1.29 (d, *J* = 5.9 Hz, 3H, CH_3). ^{13}C NMR (176 MHz, $CDCl_3$) δ (ppm): 150.7 (1C, C-7), 145.5 (1C, C-8), 139.3, 139.2, 139.1, 138.5 (4C, aromatic), 130.2 (1C, C-10), 128.7 (1C, C-5), 128.6, 128.5, 128.5, 128.2, 128.1, 128.0, 128.0, 127.8, 127.7, 127.3 (20C, aromatic), 123.2 (1C, C-6), 110.9 (1C, C-9), 87.6 (1C, C-3’), 80.2 (1C, C-5’), 80.0 (1C, C-1’), 78.9 (1C, C-4’), 78.8 (1C, C-2’), 75.8, 75.1, 75.1, 73.3 (4C, 4 x Ph-**C**H_2), 71.9 (1C, C-1), 69.9 (1C, C-3), 69.1 (1C, C-6’), 60.2, 55.9 (2C, 2 x OCH_3), 36.8 (1C, C-4), 21.9 (1C, CH_3). MALDI-ToF HRMS m/z: calcd for $C_{46}H_{50}NaO_8^+$ $[M+Na]^+$ 753.3398, found 753.3406.

4. (1*R*,3*R*)-1-(2’,3’,4’,6’-Tetra-*O*-benzyl-1’-*C*-β-D-glucopyranosyl)-7,8-dibenzyloxy-3-methylisochroman (45) and (1*S*,3*R*)-1-(2’,3’,4’,6’-tetra-*O*-benzyl-1’-*C*-β-D-glucopyranosyl)-7,8-dibenzyloxy-3-methylisochroman (46)

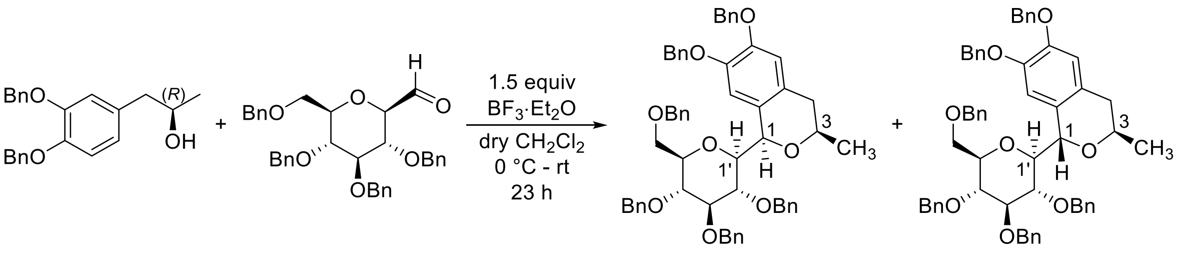

Alcohol (*R*)-**13** (51 mg, 0.14 mmol, 1 equiv.), glucopyranosyl formaldehyde **38** (120 mg, 0.22 mmol, 1.5 equiv.), dry CH_2Cl_2 (5 mL), and $BF_3 \cdot Et_2O$ (26.5 µL, 0.21 mmol, 1.5 equiv.) were used according to method B. Reaction time: 23 h. The reaction mixture was quenched with saturated $NaHCO_3$ solution (2 mL) and stirred for a further 5 min at room temperature. The crude product was purified by flash column chromatography on silica gel (hexane: EtOAc 8:1) to afford compound **45** (88 mg, 69%) as a white amorphous solid and compound **46** (9 mg, 7%) as a colorless syrup.

Compound **45**: R_f = 0.32 (hexane: EtOAc 4:1). $[\alpha]_D$ -65.7 (c 0.070, $CHCl_3$). ^{1}H NMR (500 MHz, $CDCl_3$) δ (ppm): 7.45 – 7.40 (m, 2H, aromatic), 7.39 – 7.34 (m, 4H, aromatic), 7.34 – 7.31 (m, 3H, aromatic), 7.31 – 7.24 (m, 13H, aromatic), 7.24 – 7.16 (m, 6H, aromatic), 7.14 – 7.09 (m, 2H, aromatic), 6.72 (s, 1H, H-9), 6.64 (s, 1H, H-6), 5.12 – 5.04 (m, 4H, 4 x Ph-**CH**), 5.03 (s, 1H, H-1, overlap with Ph-**CH**), 4.95 (d, *J* = 11.1 Hz, 1H, Ph-**CH**), 4.91 (d, *J* = 11.1 Hz, 1H, Ph-**CH**), 4.85 (d, *J* = 10.6 Hz, 1H, Ph-**CH**), 4.82 (d, *J* = 10.9 Hz, 1H, Ph-**CH**), 4.70 (d, *J* = 10.4 Hz, 1H, Ph-**CH**), 4.59 (d, *J* = 10.9 Hz, 1H, Ph-**CH)**, 4.34 (d, *J* = 12.1 Hz, 1H, Ph-**CH**), 4.27 (d, *J* = 12.1 Hz, 1H, Ph-**CH**), 4.00 (t, *J* = 9.2 Hz, 1H, H-2’), 3.76 (m, 1H, H-3, overlap with H-3’), 3.74 (t, *J* = 9.2 Hz, 1H, H-3’, overlap with H-3), 3.62 (dd, *J* = 9.6, 1.9 Hz, 1H, H-1’), 3.54 (t, *J* = 9.4 Hz, 1H, H-4’), 3.50 (d, *J* = 3.1 Hz, 2H, H-6’), 3.21 (dt, *J* = 9.7, 3.1 Hz, 1H, H-5’), 2.62 (dd, *J* = 15.3, 11.2 Hz, 1H, H-4a), 2.48 (dd, *J* = 15.5, 1.8 Hz, 1H, H-4b), 1.31 (d, *J* = 6.1 Hz, 3H, CH_3). ^{13}C NMR (126 MHz, $CDCl_3$) δ (ppm): 147.7 (1C, C-8), 147.4 (1C, C-7), 138.9, 138.6, 138.6, 138.4, 137.6, 137.5 (6C, aromatic), 128.6 (aromatic), 128.5 (aromatic), 128.5 (aromatic), 128.4 (1C, C-10), 128.3 (aromatic), 128.2 (aromatic), 128.0 (aromatic), 128.0 (aromatic), 127.9 (1C, C-5), 127.8, 127.7, 127.4, 127.4, 127.2 (aromatic, overall 30C), 115.0 (1C, C-6), 111.3 (1C, C-9), 87.5 (1C, C-3’), 80.4 (2C, C-1’, C-5’), 78.6 (1C, C-4’), 78.3 (1C, C-2’), 75.6, 75.3, 75.0 (3C, 3 x Ph-**C**H_2)), 74.4 (1C, C-1), 73.2 (1C, Ph-**C**H_2), 71.5, 71.4 (2C, 2 x Ph-**C**H_2), 71.0 (1C, C-3), 68.4 (1C, C-6’), 36.0 (1C, C-4), 21.9 (1C, CH_3). MALDI-ToF HRMS m/z: calcd for $C_{58}H_{58}NaO_8^+$ $[M+Na]^+$ 905.4024, found 905.4037.

Compound **46**: R_f = 0.40 (hexane: EtOAc 4:1). $[\alpha]_D$ +10.0 (c 0.050, $CHCl_3$). ^{1}H NMR (700 MHz, $CDCl_3$) δ (ppm): 7.43 – 7.40 (m, 2H, aromatic), 7.37 – 7.34 (m, 4H, aromatic), 7.31 – 7.22 (m, 20H, aromatic), 7.19 – 7.15 (m, 2H, aromatic), 7.15 – 7.11 (m, 2H, aromatic), 6.78 (d, *J* = 8.3 Hz, 1H, H-9), 6.73 (d, *J* = 8.3 Hz, 1H, H-6), 5.30 (s, 1H, H-1), 5.04 (d, *J* = 10.8 Hz, 1H, Ph-**CH**), 5.01 (d, *J* = 10.8 Hz, 1H, Ph-**CH**), 4.98 (d, *J* = 11.6 Hz, 1H, Ph-**CH**, overlap with Ph-CH), 4.97 (d, *J* = 11.9 Hz, 1H, Ph-**CH**, overlap with Ph-CH), 4.95 – 4.87 (m, 3H, 3 x Ph-**CH**), 4.85 (d, *J* = 10.8 Hz, 1H, Ph-**CH**), 4.82 (d, *J* = 11.0 Hz, 1H, Ph-**CH**), 4.58 (d, *J* = 11.0 Hz, 1H, Ph-**CH**), 4.27 (d, *J* = 12.1 Hz, 1H, Ph-**CH**), 4.17 (d, *J* = 12.0 Hz, 1H, Ph-**CH**), 4.09 (dd, *J* = 9.6, 1.7 Hz, 1H, H-1'), 4.03 (t, *J* = 9.3 Hz, 1H, H-2'), 3.65 (t, *J* = 9.1 Hz, 1H, H-3'), 3.60 – 3.54 (m, 2H, H-3, H-6'a), 3.51 (dd, *J* = 11.9, 4.9 Hz, 1H, H-6'b), 3.48 (t, *J* = 9.4 Hz, 1H, H-4'), 3.30 (dd, *J* = 9.8, 3.9 Hz, 1H, H-5'), 2.65 (dd, *J* = 14.5, 11.0 Hz, 1H, H-4a), 2.46 (d, *J* = 14.0 Hz, 1H, H-4b), 1.28 (d, *J* = 6.0 Hz, 3H, CH_3). ^{13}C NMR (176 MHz, $CDCl_3$) δ (ppm): 149.9 (1C, C-7), 144.6 (1C, C-8), 139.6, 139.2, 139.0, 138.6, 137.5, 137.3 (6C, aromatic), 131.0 (1C, C-10), 129.5 (1C, C-5), 128.6, 128.6, 128.5, 128.5, 128.4, 128.4, 128.2, 128.1, 128.0, 127.9, 127.8, 127.7, 127.7, 127.7, 127.6, 127.4, 127.3 (30C, aromatic), 123.3 (1C, C-6), 113.0 (1C, C-9), 87.5 (1C, C-3'), 80.1 (1C, C-5'), 79.9 (1C, C-1'), 78.9 (1C, C-4'), 78.7 (1C, C-2'), 75.7, 75.0, 74.7, 74.3, 73.2 (5C, 5 x Ph-**C**H_2), 71.8 (1C, C-1), 71.2 (1C, Ph-**C**H_2), 69.8 (1C. C-3), 69.2 (1C, C-6'), 36.9 (1C, C-4), 21.9 (1C, CH_3). MALDI-ToF HRMS m/z: calcd for $C_{58}H_{58}NaO_8^+$ $[M+Na]^+$ 905.4024, found 905.4013.

5. (1*S*,3*S*)-1-(1'-*C*-β-D-Glucopyranosyl)-3-methyl-7,8-dimethoxyisochroman (47)

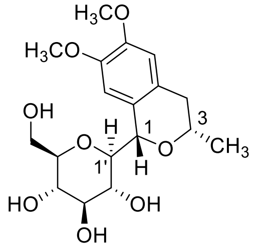

47

Compound **39** (170 mg, 0.23 mmol, 1 equiv.) was converted to **47** according to method C. The crude product was purified by flash column chromatography on silica gel (CH_2Cl_2: MeOH 12:1) to afford **47** (78 mg, 90%) as a white amorphous. R_f = 0.59 (CH_2Cl_2: MeOH 4:1). $[\alpha]_D$ +20.0 (c 0.16, MeOH). ^{1}H NMR (500 MHz, MeOD) δ (ppm): 7.12 (s, 1H, H-9), 6.65 (s, 1H, H-6), 4.94 (d, *J* = 4.8 Hz, 1H, H-1), 3.91 (dd, *J* = 11.9, 1.8 Hz, 1H, H-6'a), 3.81 (s, 3H, OCH_3), 3.80 (m, 1H, H-3, overlap with the two OCH_3 signals), 3.79 (s, 3H, OCH_3), 3.71 (t, *J* = 9.1 Hz, 1H, H-2'), 3.66 (dd, *J* = 11.8, 5.8 Hz, 1H, H-6'b), 3.50 (dd, *J* = 9.4, 5.1 Hz, 1H, H-1'), 3.39 (t, *J* = 8.6 Hz, 1H, H-3'), 3.34 (dd, *J* = 9.6, 2.0 Hz, 1H, H-5'), 3.30 (t, *J* = 8.4 Hz, 1H, H-4'), 2.67 (dd, *J* = 15.5, 10.8 Hz, 1H, H-4a), 2.59 (dd, *J* = 15.6, 2.3 Hz, 1H, H-4b), 1.35 (d, *J* = 6.0 Hz, 3H, CH_3). ^{13}C NMR (126 MHz, MeOD) δ (ppm): 149.1, 148.8 (2C, C-7, C-8), 129.1, 127.7 (2C, C-5, C-10), 112.7 (1C, C-6), 110.2 (1C, C-9), 83.3 (1C, C-1'), 81.7 (1C, C-5'), 79.9 (1C, C-1), 79.5 (1C, C-3'), 73.7 (1C, C-2'), 72.4 (1C, C-3), 71.5 (1C, C-4'), 63.1 (1C, C-6'), 56.6, 56.4 (2C, 2 x OCH_3), 37.0 (1C, C-4), 21.8 (1C, CH_3). MALDI-ToF HRMS: m\z calcd for $C_{18}H_{26}NaO_8^+$ $[M+Na]^+$ 393.1520, found 393.1526.

6. (1*R*,3*R*)-1-(1'-*C*-β-D-Glucopyranosyl)-3-methyl-7,8-dimethoxyisochroman (48)

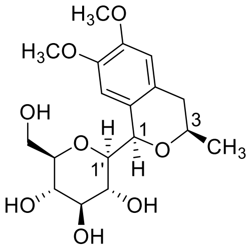

48

Compound **43** (126 mg, 0.17 mmol, 1 equiv.) was converted to **48** according to method C. The crude product was purified by flash column chromatography on silica gel (CH_2Cl_2: MeOH 12.5:1) to afford compound **48** (53 mg, 84%) as a white solid. R_f = 0.22 (CH_2Cl_2: MeOH 9:1). M.p.: No sharp melting point could be detected because of the solvent content of the lattice. $[\alpha]_D$ -12.5 (c 0.040, MeOH). ^{1}H NMR (500 MHz, MeOD) δ (ppm): 6.81 (s, 1H, H-9), 6.68 (s, 1H, H-6), 5.04 (d, *J* = 2.1 Hz, 1H, H-1), 3.86 – 3.82 (m, 1H, H-3, overlap with H-1' and OCH_3), 3.81 (m, 1H, H-1', overlap with H-3 and OCH_3), 3.79 (s, 3H, OCH_3, overlap with H-1' and H-3), 3.79 (s, 3H, OCH_3, overlap with H-1' and H-3), 3.71 (t, *J* = 9.2 Hz, 1H, H-2'), 3.66 (dd, *J* = 12.2, 2.4 Hz, 1H, H-6'a), 3.60 (dd, *J* = 12.2, 4.5 Hz, 1H, H-6'b), 3.47 (t, *J* = 8.9 Hz, 1H, H-3'), 3.38 (t, *J* = 9.3 Hz, 1H, H-4'), 3.21 (ddd, *J* = 9.6, 4.5, 2.4 Hz, 1H, H-5'), 2.70 – 2.59 (m, 2H, H-4), 1.34 (d, *J* = 6.1 Hz, 3H, CH_3). ^{13}C NMR (126 MHz, MeOD) δ (ppm): 149.3, 148.9 (2C, C-7, C-8), 129.0 (1C, C-5), 128.4 (1C, C-10), 113.2 (1C, C-6), 109.1 (1C, C-9), 82.1 (1C, C-5'), 81.0 (1C, C-1'), 80.1 (1C, C-3'), 76.0 (1C, C-1), 73.0 (1C, C-3), 71.2 (1C, C-4'), 70.9 (1C, C-2'), 62.5 (1C, C-6'), 56.7, 56.4 (2C, 2 x OCH_3), 36.9 (1C, C-4), 21.8 (1C, CH_3). MALDI-ToF HRMS m/z: calcd for $C_{18}H_{26}NaO_8^+$ $[M+Na]^+$ 393.1520, found 393.1524.

7. (1*S*,3*S*)-1-(1'-*C*-β-D-Glucopyranosyl)-7,8-dihydroxy-3-methylisochroman (49)

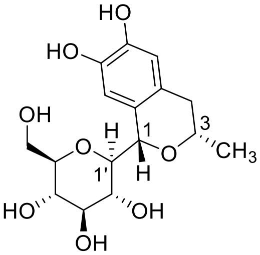

49

Compound **41** (155 mg, 0.18 mmol, 1 equiv.) was converted to **49** according to method C. The crude product was purified by flash column chromatography on silica gel (CH_2Cl_2: MeOH 6:1) to afford **49** (50 mg, 83%) as a white amorphous. R_f= 0.71 (CH_2Cl_2: MeOH 7:3). $[\alpha]_D$ +46.7 (c 0.090, MeOH). ^{1}H NMR (500 MHz, MeOD) δ (ppm): 6.87 (s, 1H, H-9), 6.48 (s, 1H, H-6), 4.88 (d, *J* = 4.2 Hz, 1H, H-1), 3.92 (d, *J* = 11.9 Hz, 1H, H-6'a), 3.75 (ddd, *J* = 10.7, 6.1, 2.6 Hz, 1H, H-3), 3.74 – 3.65 (m, 2H, H-2', H-6'b), 3.49 (dd, *J* = 9.4, 4.4 Hz, 1H, H-1'), 3.39 (t, *J* = 8.3 Hz, 1H, H-3'), 3.34 – 3.29 (m, 2H, H-4', H-5'), 2.59 (dd, *J* = 15.1, 11.1 Hz, 1H, H-4a), 2.47 (dd, *J* = 15.6, 1.9 Hz, 1H, H-4b), 1.31 (d, *J* = 6.0 Hz, 3H, CH_3). ^{13}C NMR (126 MHz, MeOD) δ (ppm): 145.1, 144.6 (2C, C-7, C-8), 127.6, 126.7 (2C, C-5, C-10), 115.9 (1C, C-6), 113.2 (1C, C-9), 83.5 (1C, C-1'), 81.6 (1C, C-5'), 79.6, 79.6 (2C, C-1, C-3'), 73.4 (1C, C-2'), 72.5 (1C, C-3), 71.3 (1C, C-4'), 63.0 (1C, C-6'), 36.9 (1C, C-4), 21.9 (1C, CH_3). MALDI-ToF HRMS: m\z calcd for $C_{16}H_{22}NaO_8^+$ $[M+Na]^+$ 365.1207, found 365.1208.

8. (1*R*,3*R*)-1-(1'-*C*-β-D-Glucopyranosyl)-7,8-dihydroxy-3-methylisochroman (50)

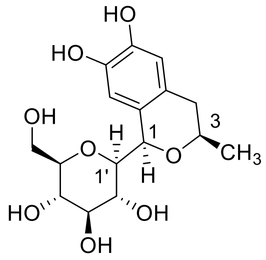

50

Compound **45** (159 mg, 0.18 mmol, 1 equiv.) was converted to **50** according to method C. The crude product was purified by flash column chromatography on silica gel (CH_2Cl_2: MeOH 6:1) to afford **50** (49 mg, 79%) as a white amorphous. R_f = 0.32 (CH_2Cl_2: MeOH = 3:1). $[\alpha]_D$ -65.8 (c 0,12, MeOH). ^{1}H NMR (500 MHz, MeOD) δ (ppm): 6.65 (s, 1H, H-9), 6.51 (s, 1H, H-6), 4.97 (s, 1H, H-1), 3.80 (ddd, J = 10.2, 7.9, 4.9 Hz, 1H, H-3), 3.75 – 3.69 (m, 2H, H-1', H-2'), 3.67 (dd, J = 12.1, 1.8 Hz, 1H, H-6'a), 3.62 (dd, J = 12.2, 4.1 Hz, 1H, H-6'b), 3.45 (t, J = 8.0 Hz, 1H, H-4'), 3.40 (t, J = 9.1 Hz, 1H, H-3'), 3.18 (ddd, J = 9.3, 3.8, 2.4 Hz, 1H), H-5', 2.58 (dd, J = 15.9, 10.7 Hz, 1H, H-4a), 2.53 (dd, J = 15.9, 3.7 Hz, 1H, H-4b), 1.32 (d, J = 6.1 Hz, 3H, CH_3). ^{13}C NMR (126 MHz, MeOD) δ (ppm): 145.1 (1C, C-8), 144.7 (1C, C-7), 127.5 (1C, C-10), 127.2 (1C, C-5), 116.3 (1C, C-6), 111.7 (1C, C-9), 82.0 (1C, C-5'), 81.0 (1C, C-1'/C-2'), 80.1 (1C, C-4'), 75.8 (1C, C-1), 73.2 (1C, C-3), 71.0 (1C, C-3'), 70.8 (1C, C-1'/C-2'), 62.4 (1C, C-6'), 36.8 (1C, C-4), 21.9 (1C, CH_3). MALDI-ToF HRMS m/z: calcd for $C_{16}H_{22}NaO_8^+$ $[M+Na]^+$ 365.1207, found 365.1211.

Determination of the C-1 configuration by ^{1}H-^{1}H ROESY NMR experiments

During the oxa-Pictet-Spengler reaction a new stereogenic center (isochroman C-1) was formed, and the configuration of the newly formed C-1 was determined using ^{1}H-^{1}H ROESY NMR experiments. These measurements provide the opportunity to investigate hydrogen atoms that are close to each other in space, with the maximum distance of 5 Å. In those cases where the H-1 and H-3 atoms are located on the same side of the isochroman ring (compound **25**, **41**, **49**), the appearance of ROESY correlations is expected.

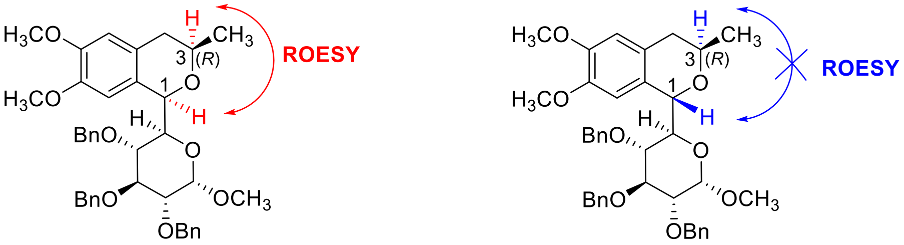

25 **26**

Scheme S6. Structural elucidation of compound **25** and **26** based on ROESY correlation

In contrast, in the case of the epimeric compounds **26** and **42**, H-1 and H-3 are located on the opposite side of the isochroman ring, so we expected to observe the lack of the correlation peaks.

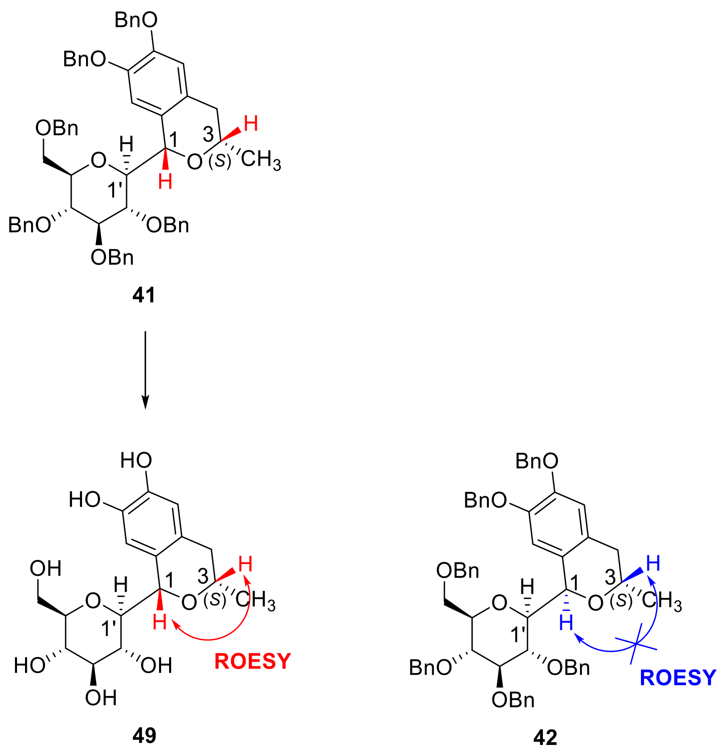

Scheme S7. Structural elucidation of compound **41, 42** and **49** based on the ROESY correlations

The ROESY experiments proved our expectations, ROESY correlations were observed only in the case of compound **25** and **49** (Scheme 8 and scheme 10). It allowed the determination of the *cis* relative configuration of H-1 and H-3 atoms, which afforded the absolute configuration of the C-1 atom based on the known C-3 absolute configuration.

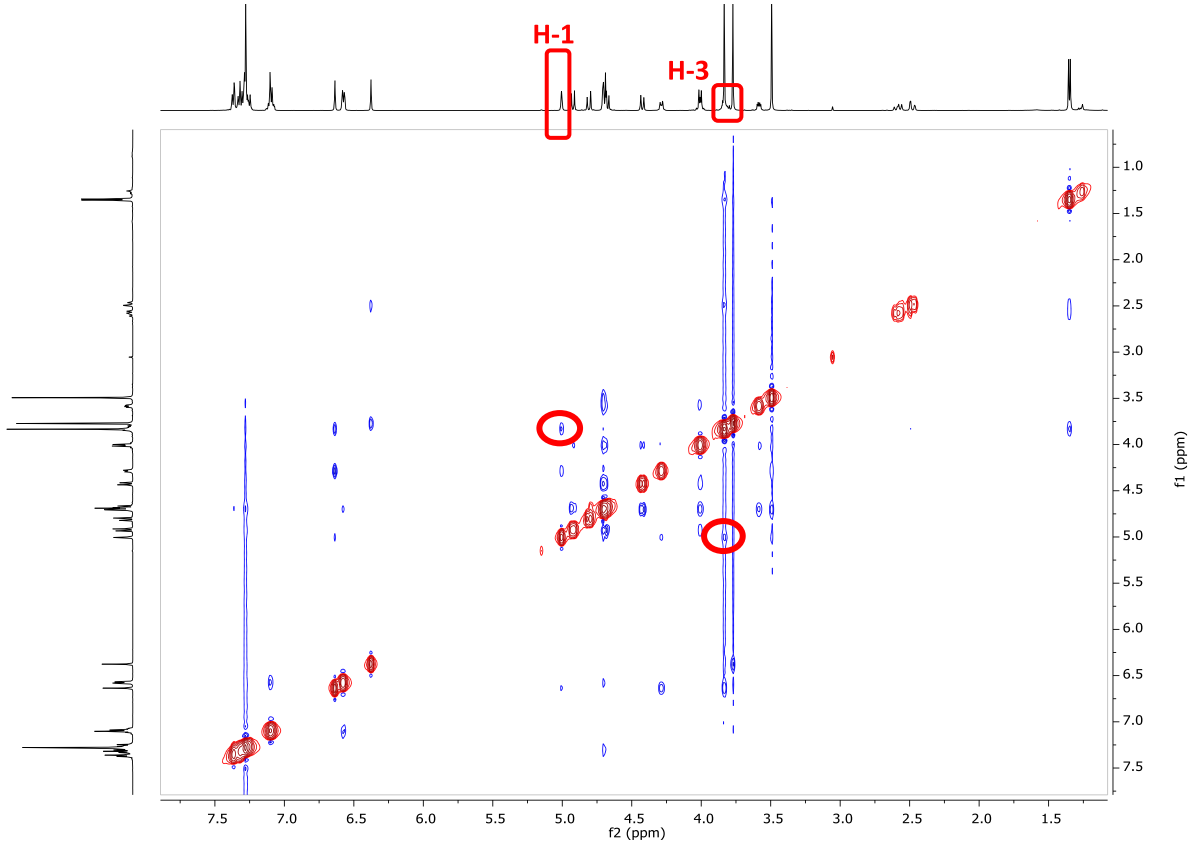

Scheme S8. ROESY NMR spectra of compound **25** with correlation peaks between H-1 and H-3

Although the absence of ROESY correlation peak is indirect evidence for the relative configuration of the H-1 and H-3 atoms, during the structural analysis of the isomer pairs we always found that the ROESY cross peak could be detected just for one isomer, while not for the other.

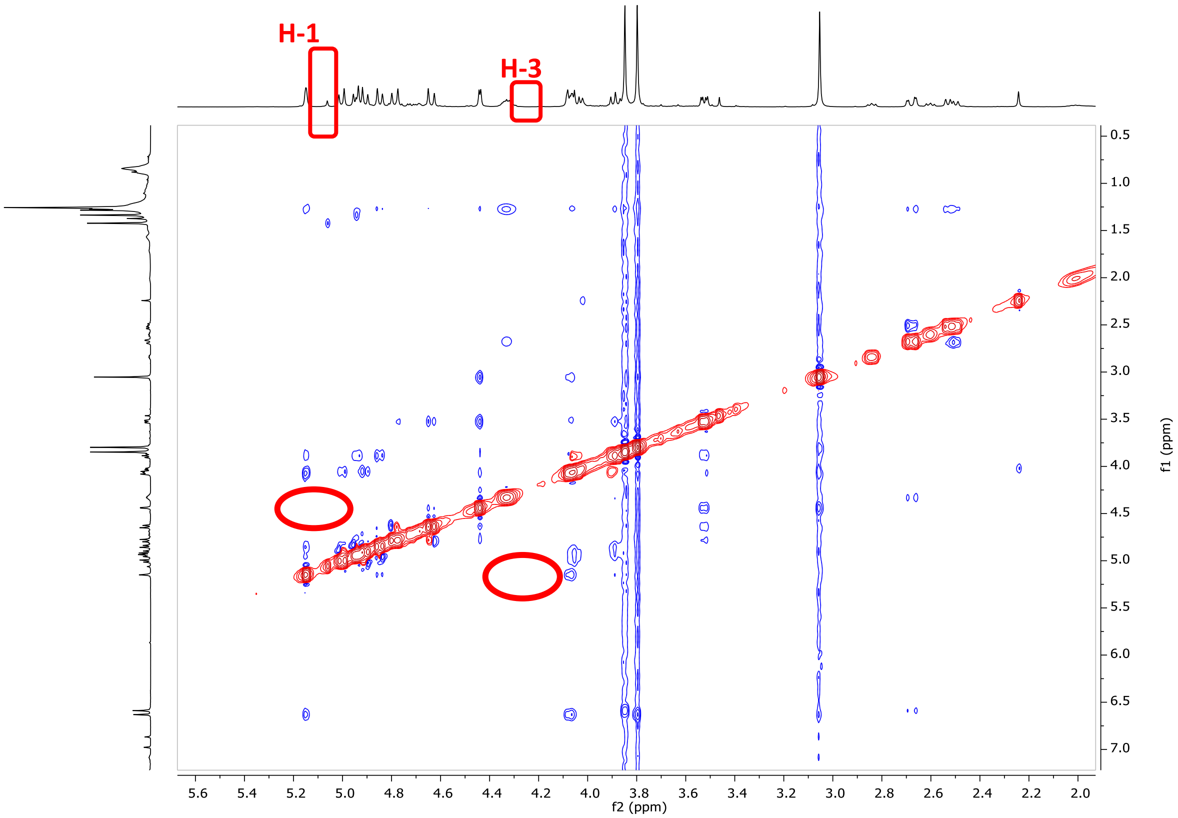

Scheme S9. ^1H-^1H ROESY NMR spectra of compound **26**

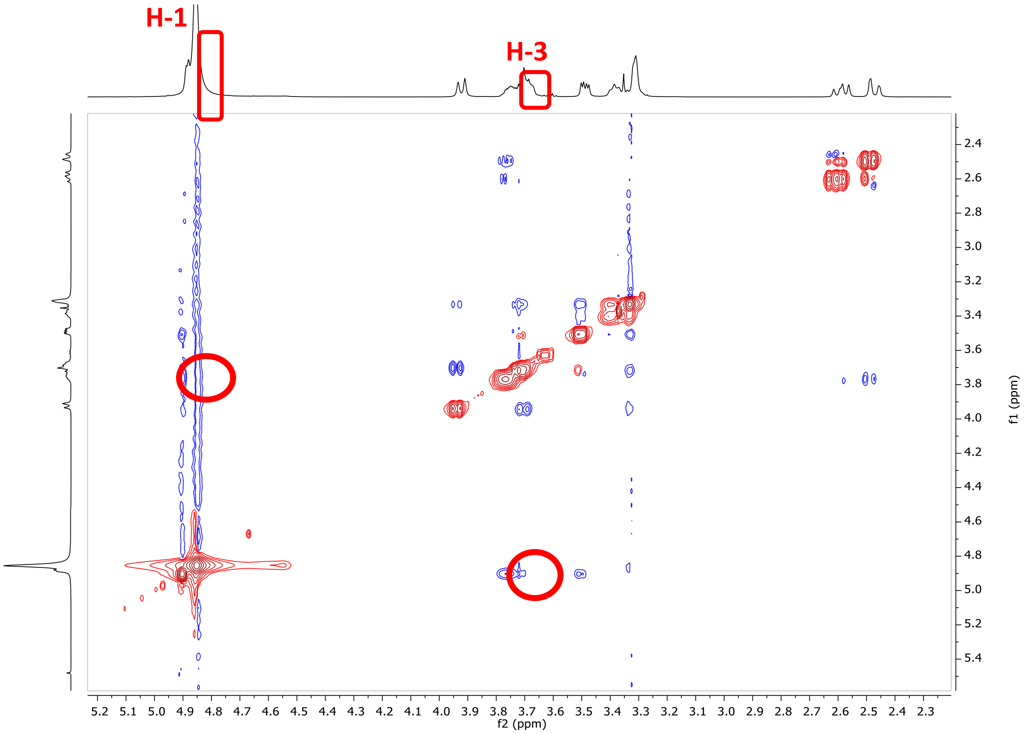

Scheme S10. ROESY correlations from compound **49**

Beside the NMR experiments, in some cases the structures were confirmed by X-Ray analysis. These results were consistent with the NMR data, so it proved the absolute configuration from an other point.

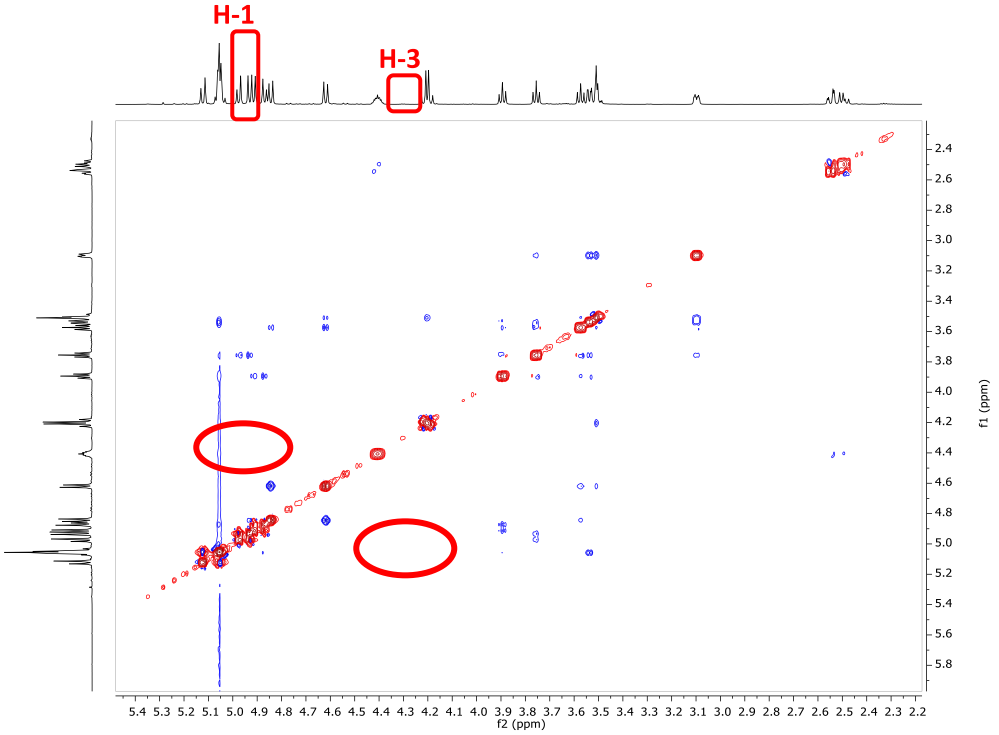

Scheme S11. ^{1}H-^{1}H ROESY NMR spectra of compound **42** and the lack of the correlation

X-ray diffraction experiments

During the workup and after evaporation of the solvent, X-ray quality crystals could be grown. In the asymmetric unit together with the carbohydrate moiety in **3** there is a solvent methanol, in **7** a solvent chloroform, in **24** two methanol molecules while in **48** two carbohydrate molecules crystallized together with two water and one methanol molecules. For data collection a properly chosen single crystal was fixed onto a Mitegen loop using high-density oil. Diffraction Intensity data was collected at low or ambient temperature (150 or 294 K) on a Bruker-D8 Venture diffractometer (Bruker AXS GmbH, Karlsruhe,Germany) equipped with INCOATEC IµS 3.0 (Incoatec GmbH, Geesthacht, Germany) dual (Cu and Mo) sealed tube micro sources and a Photon II Charge-Integrating Pixel Array detector (Bruker AXS GmbH, Karlsruhe, Germany) using Mo Kα (λ = 0.71073 Å) radiation. High-multiplicity data collection and integration were performed using APEX3 (version 2017.3-0, Bruker AXS Inc., 2017, Madison, WI, USA) software. Data reduction and multiscan absorption correction were performed using SAINT (version 8.38A, Bruker AXS Inc., 2017, Madison, WI, USA). The structures could be solved using direct methods and refined on F^2 using SHELXL program incorporated into the APEX4 suite. In all cases the space group is allowed for enantiopure compound as it is expected. For data collection Mo Kα radiation was used as the assignment of absolute configuration was not an issue and the syntehsis i.e. use of carbohydrate unambigously indicated the configuration of all of the stereogenic centers. Refinement was performed anisotropicaly for all non-hydrogen atoms. Hydrogens were placed usually into geometric positions, however, in some cases O-H protons could be found at the

difference electron density map and O-H distances were constrained. Tables were extracted from the edited CIF file using publ CIF [11, 12].

Table S1. Experimental details and results of single crystal X-ray diffraction studies

**Compound**	**3**	**7**	**24**	**48**
Crystal data				
Chemical formula	$C_{18}H_{26}O_8·CH_4O$	$C_{18}H_{26}O_8·CHCl_3$	$C_{16}H_{22}O_8·2(CH_4O)$	$2(C_{18}H_{26}O_8)·CH_4O·3(H_2O)$
M_r	402.43	489.75	406.42	826.86
Crystal system, space group	Monoclinic, $P2_1$	Monoclinic, $P2_1$	Orthorhombic, $P2_12_12_1$	Triclinic, $P1$
Temperature (K)	294	294	293	293
a, b, c (Å)	8.4789 (4), 6.9513 (3), 16.9764 (9)	11.485 (5), 7.518 (3), 13.937 (5)	8.836 (2), 10.441 (2), 21.975 (5)	7.5257 (17), 11.220 (3), 13.808 (3)
α, β, γ (°)	90, 101.958 (2), 90	90, 94.994 (13), 90	90, 90, 90	104.104 (9), 101.501 (10), 106.042 (9)
V (Å^3)	978.87 (8)	1198.8 (8)	2027.3 (8)	1041.3 (4)
Z	2	2	4	1
Radiation type	Mo $K\alpha$			
μ (mm^{-1})	0.11	0.42	0.11	0.11
Crystal size (mm)	0.26 × 0.20 × 0.10	0.36 × 0.11 × 0.06	0.31 × 0.22 × 0.21	0.30 × 0.20 × 0.11
Data collection				
Diffractometer	Bruker D8 VENTURE			
Absorption correction	Multi-scan *SADABS2016*/2 - Bruker AXS area detector scaling and absorption correction			
T_{min}, T_{max}	0.97, 0.99	0.41, 0.98	0.97, 0.98	0.50, 0.99
No. of measured, independent and observed [$I > 2\sigma(I)$] reflections	16032, 3748, 3021	24210, 4378, 2989	10457, 3846, 2880	17580, 6747, 4616
R_{int}	0.055	0.158	0.074	0.124

$(\sin \theta/\lambda)_{max}$ (Å^{-1})	0.611	0.604	0.613	0.614
Refinement				
$R[F^2 > 2\sigma(F^2)]$, $wR(F^2)$, S	0.043, 0.126, 1.10	0.119, 0.333, 1.18	0.080, 0.201, 1.07	0.173, 0.452, 1.22
No. of reflections	3748	4378	3846	6747
No. of parameters	271	285	276	453
No. of restraints	5	227	10	429
H-atom treatment	H atoms treated by a mixture of independent and constrained refinement			
$(\Delta/\sigma)_{max}$	< 0.001	< 0.001	0.001	0.434
$\Delta\rangle_{max}$, $\Delta\rangle_{min}$ (e Å^{-3})	0.29, -0.27	0.89, -0.64	1.01, -0.44	0.87, -1.54
Absolute structure parameter	0.0 (6)	-0.06 (19)	0.5 (10)	-0.6 (10)

X-ray structures of compounds 3, 7, 24, 48

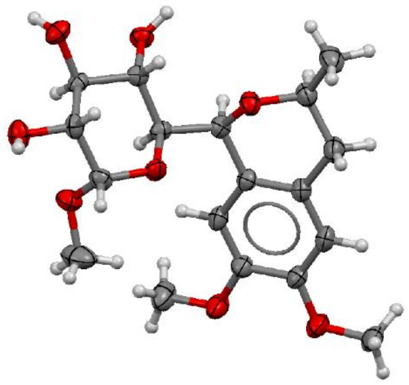

Figure S1. ORTEP view of **3** at a 40 % probability level. Solvent molecules are omitted for clarity.

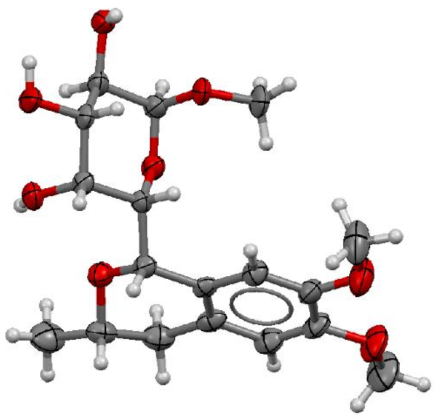

Figure S2. ORTEP view of **7** at a 40 % probability level. Solvent molecules are omitted for clarity.

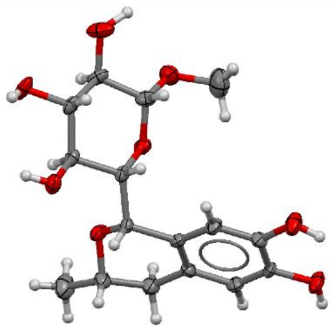

Figure S3. ORTEP view of **24** at 40 % probability level. Solvent molecules are omitted for clarity.

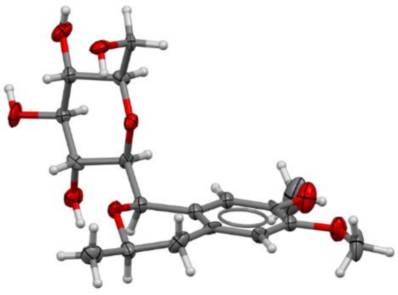

Figure S4. ORTEP view of **48** at 40 % probability level. Solvent molecules are omitted for clarity, only one molecule from the asymmetric unit is shown.

Computational Section

Mixed torsional/low-frequency mode conformational searches were carried out by means of the Macromodel 10.8.011 software, using the Merck Molecular Force Field (MMFF) with an implicit solvent model for $CHCl_3$ [13]. All quantum chemical calculations were carried out with the Gaussian 16 software package [14]. The B3LYP, B3PW91 [15] and ωB97X [16] functionals with the TZVP basis set and the PCM solvent model for DMSO, MeOH or CH_2Cl_2 were used to re-optimize the MMFF geometries. VCD calculations were performed at the B3LYP/TZVP and

B3PW91/TZVP level with the same solvent model as in the preceding DFT optimization step. VCD spectra were gained by applying an 8 cm^{-1} half-height width and scaled by a factor of 0.98 [17, 18]. Boltzmann distributions were estimated from the B3LYP, B3PW91 and ωB97X energies. The MOLEKEL software package was used for visualization of the results [19].

Table S2. C8-C8a-C1-C5' and C8-C8a-C1-1H angle values of the investigated derivatives.

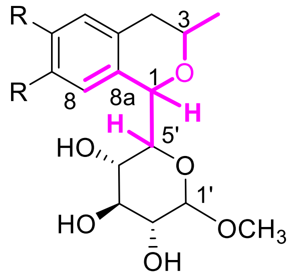

3 R = OCH_3 (1*S*,3*S*,1'α)
trans-**3** R = OCH_3 (1*R*,3*S*,1'α)
4 R = OCH_3 (1*R*,3*R*,1'α)
5 R = OCH_3 (1*S*,3*R*,1'α)
31mod R = OBn (1*R*,3*R*,1'β)
32mod R = OBn (1*S*,3*R*,1'β)
34 R = OCH_3 (1*R*,3*R*,1'β)
35 R = OCH_3 (1*S*,3*R*,1'β)

derivative / angle	C8-C8a-C1-C5' angle (°)	C8-C8a-C1-1H angle (°)
**3**	51	-67
trans-**3**	-68	50
**4**	-56	64
**5**	61	-55
**31mod**	-40	78
**32mod**	61	-55
**34**	-44	75
**35**	62	-54

Quantitative comparison of VCD spectra

To quantitatively evaluate the agreement between experimental and calculated VCD spectra, the cosine similarity index (CSI) was used as a shape-based similarity metric. The CSI measures the angular similarity between two vectors thus it is independent of the absolute intensity and therefore also suitable for comparing VCD data of different scales.

Before comparison, both spectra were baseline-corrected by subtraction of their mean Δε values, ensuring that constant offsets did not influence the results. Each spectrum was then normalized so that the analysis reflected only the relative spectral features and not their magnitude. The result of the CSI analysis is between −1 and +1, where +1 corresponds to identical spectral shapes, 0 to no similarity, and −1 to perfect mirror-image spectra.

For the comparison of experimental and calculated spectra, the calculated dataset was linearly interpolated onto the wavenumber grid of the experimental spectrum to enable pointwise comparison. For comparisons between two experimental spectra, both datasets were instead interpolated onto a shared uniform grid with 1 cm^{-1} increments, providing a consistent basis for comparison.

The cosine similarity index was calculated as:

$$CSI(A,B) = cos(\theta) = \frac{A \cdot B}{\|A\|\|B\|} = \frac{\sum_{i=1}^{n} A_i B_i}{\sqrt{\sum_{i=1}^{n} A_i^2}\sqrt{\sum_{i=1}^{n} B_i^2}}$$

where A_i and B_i represent the normalized Δε values of the two spectra at wavenumber i.

All mathematical operations, including interpolation, baseline correction, normalization, and dot-product computation, were performed with an in-house script written in Python 3 using the NumPy library (np.interp, np.mean, np.linalg.norm, and np.dot) [20].

Cartesian coordinates and energies of the low-energy conformers

Table S3. Cartesian coordinates and energies of the low-energy conformers calculated at the B3LYP/TZVP PCM/DMSO level.

3, Conf A

C	0.218564	1.139831	0.769263
H	0.262474	1.323995	1.852067
C	2.287904	-1.079773	-1.383229
H	2.159957	-1.117773	-2.468817
C	3.710890	-0.638784	-1.019282
H	3.946168	0.259318	-1.592234
C	-0.548842	3.308183	0.073280
H	-0.902133	3.453491	1.104015
C	3.804533	-0.309668	0.469536
H	3.660469	-1.227869	1.051262
C	-1.688171	2.749284	-0.770807
H	-2.555458	3.411990	-0.709609
H	-1.372365	2.732751	-1.820208
C	2.728954	0.691903	0.866321
H	2.910880	1.634667	0.341193
C	-3.340932	0.849014	-0.575524
H	-4.030921	1.455756	-1.146165
C	-2.063824	1.362901	-0.303815
C	1.351586	0.155204	0.464150
H	1.168658	-0.760931	1.032296
C	-3.733719	-0.398732	-0.119508
C	-2.828366	-1.162862	0.653750
C	-1.568002	-0.653156	0.919898
H	-0.883639	-1.234728	1.520711
C	0.014540	4.612825	-0.456606
H	-0.766404	5.374766	-0.491440
H	0.409016	4.477356	-1.466059
H	0.819188	4.974481	0.185331
C	-1.165374	0.599301	0.430444
C	0.988484	-3.084778	-1.356240
H	1.087210	-3.205653	-2.439209
H	0.049028	-2.577308	-1.130039
H	0.996818	-4.062726	-0.880121
C	-5.906408	-0.229040	-1.112549
H	-6.789909	-0.859919	-1.170352
H	-5.534714	-0.030961	-2.121277
H	-6.166099	0.715632	-0.627624
C	-2.427536	-3.167654	1.903917
H	-2.167645	-2.650185	2.831188
H	-1.513548	-3.441818	1.370807
H	-2.991451	-4.067103	2.138368
H	4.329785	-2.474862	-1.112334
H	5.755371	-0.364045	0.569943
H	3.633072	1.121775	2.532659
O	2.104545	-2.360549	-0.827974
O	0.521628	2.352787	0.087237
O	4.675622	-1.616395	-1.397744
O	5.067228	0.273816	0.798441
O	2.726270	0.907384	2.277547
O	1.331241	-0.136876	-0.941751
O	-4.954713	-0.963337	-0.343574
O	-3.286761	-2.372342	1.088777

B3LYP Energy = -1303.78876061 a.u.

3, Conf B

C	-0.092552	1.198654	-0.930260
H	-0.185898	1.180401	-2.024714
C	-2.985558	-1.123198	-0.674543
H	-3.012887	-2.075639	-1.211078
C	-3.112094	-1.358420	0.838892
H	-2.386458	-2.121666	1.124318
C	1.204690	3.232373	-0.778433
H	1.440594	3.067413	-1.837408
C	-2.800826	-0.074775	1.603892
H	-3.585992	0.663058	1.399947
C	2.287523	2.604803	0.086974
H	3.264669	3.000591	-0.201240
H	2.122381	2.899186	1.129814
C	-1.466073	0.514721	1.152000
H	-0.665850	-0.174748	1.445753
C	3.429967	0.368331	0.282375
H	4.293002	0.904733	0.652528
C	2.279902	1.099810	-0.049752
C	-1.437831	0.689762	-0.369966
H	-2.186129	1.437562	-0.651160
C	3.485876	-1.007465	0.131408
C	2.357523	-1.685549	-0.386043
C	1.222183	-0.963854	-0.713881
H	0.357704	-1.473543	-1.108317
C	0.996712	4.712305	-0.525952
H	0.715959	4.890080	0.514455
H	0.211817	5.107810	-1.171913
H	1.918798	5.259324	-0.730389
C	1.159838	0.427947	-0.529892
C	-4.318556	-0.270897	-2.463731
H	-4.486826	-1.277390	-2.858459
H	-3.483600	0.192191	-2.993387
H	-5.214732	0.328983	-2.604880
C	5.731261	-1.142395	0.952820
H	5.518111	-0.633522	1.896419

H	6.144423	-0.426724	0.237329
H	6.452242	-1.937461	1.125569
C	1.379800	-3.766639	-1.057488
H	1.132799	-3.436995	-2.070100
H	0.498169	-3.671487	-0.418365
H	1.696909	-4.806250	-1.083239
H	-5.045229	-1.331308	0.710395
H	-2.534239	0.442542	3.457969
H	-0.735113	2.321700	1.289491
O	-4.073380	-0.317821	-1.054516
O	-0.042981	2.565475	-0.496601
O	-4.391696	-1.893779	1.149805
O	-2.771428	-0.373967	2.999478
O	-1.308805	1.759565	1.837483
O	-1.734836	-0.553828	-1.019132
O	4.565579	-1.781826	0.433395
O	2.485630	-3.036558	-0.528645

B3LYP Energy = -1303.78816782 a.u.

3, Conf C

C	0.305203	1.123397	0.739065
H	0.332699	1.334468	1.817429
C	2.357013	-1.206606	-1.309453
H	2.240456	-1.283377	-2.394285
C	3.784061	-0.779644	-0.944614
H	4.043218	0.092285	-1.546934
C	-0.406846	3.296234	0.016376
H	-0.751413	3.457818	1.047625
C	3.866879	-0.398374	0.532545
H	3.699845	-1.292445	1.144859
C	-1.563427	2.760319	-0.819464
H	-2.416305	3.440853	-0.755217
H	-1.254550	2.733697	-1.870530
C	2.805359	0.635639	0.881342
H	3.010871	1.556224	0.326431
C	-3.246705	0.891299	-0.648619
H	-3.915940	1.511490	-1.229574
C	-1.967921	1.380680	-0.355123
C	1.423424	0.110399	0.480268
H	1.216593	-0.782613	1.076400
C	-3.661658	-0.361115	-0.210581
C	-2.777685	-1.131442	0.570920
C	-1.515810	-0.643301	0.857780
H	-0.871311	-1.267986	1.464059
C	0.185054	4.582811	-0.526061
H	0.573047	4.429514	-1.535466
H	0.999832	4.930690	0.110591
H	-0.577895	5.362542	-0.565142
C	-1.082047	0.600650	0.386427
C	1.012933	-3.180691	-1.215158
H	1.001867	-4.142344	-0.707011
H	1.111958	-3.338875	-2.293278
H	0.082419	-2.648966	-1.008567
C	-5.807907	-0.169609	-1.262675

H	-5.407158	0.037677	-2.257966
H	-6.078402	0.769627	-0.773860
H	-6.690248	-0.798157	-1.351686
C	-4.067725	-2.432666	2.087268
H	-4.225636	-3.484274	2.318384
H	-5.015752	-1.974785	1.801625
H	-3.660481	-1.924471	2.965662
H	4.370500	-2.628788	-0.960167
H	5.815205	-0.483240	0.658474
H	3.697832	1.103998	2.543458
O	2.141424	-2.460633	-0.707682
O	0.639202	2.314267	0.033634
O	4.734188	-1.788320	-1.275358
O	5.135995	0.174414	0.855376
O	2.789855	0.899904	2.284084
O	1.414981	-0.227924	-0.915407
O	-4.878101	-0.915103	-0.475409
O	-3.125597	-2.393911	1.002416

B3LYP Energy = -1303.78604732 a.u.

3, Conf D

C	0.307763	1.193678	0.725986
H	0.373154	1.469870	1.787883
C	2.302177	-1.245054	-1.253697
H	2.158593	-1.372638	-2.330482
C	3.737309	-0.801727	-0.945210
H	3.979656	0.043233	-1.591361
C	-0.406951	3.326352	-0.110128
H	-0.717150	3.558701	0.918487
C	3.856025	-0.357107	0.511402
H	3.702269	-1.223206	1.165876
C	-1.594321	2.750398	-0.872861
H	-2.436793	3.445524	-0.832382
H	-1.316359	2.645945	-1.927695
C	2.804578	0.693904	0.838399
H	2.999600	1.588273	0.238626
C	-3.302041	0.935186	-0.496294
H	-3.990271	1.535641	-1.075696
C	-2.000802	1.413054	-0.299783
C	1.411147	0.157609	0.494024
H	1.212303	-0.702558	1.139215
C	-3.724645	-0.267551	0.057825
C	-2.813954	-1.012883	0.833150
C	-1.529335	-0.535608	1.024442
H	-0.870052	-1.130141	1.645012
C	0.180016	4.567765	-0.753716
H	1.018454	4.946295	-0.167251
H	-0.575900	5.352548	-0.819264
H	0.533561	4.344642	-1.762749
C	-1.097015	0.667713	0.456104
C	0.966508	-3.214178	-1.035488
H	1.040408	-3.423268	-2.106934
H	0.040658	-2.672928	-0.832906
H	0.968299	-4.150565	-0.482072

C	-5.940284	-0.056132	-0.835867
H	-6.851612	-0.647804	-0.805298
H	-5.614406	0.057014	-1.872777
H	-6.131741	0.928573	-0.402497
C	-3.402932	-3.311275	0.616838
H	-4.205893	-3.129906	-0.098362
H	-3.675382	-4.137547	1.270462
H	-2.480991	-3.558675	0.083924
H	4.326123	-2.649497	-0.894658
H	5.806687	-0.440890	0.597669
H	3.736346	1.232958	2.456942
O	2.104688	-2.470230	-0.589102
O	0.629158	2.335254	-0.062019
O	4.679908	-1.823622	-1.256230
O	5.132914	0.226649	0.779318
O	2.822182	1.020233	2.228020
O	1.368052	-0.250169	-0.882325
O	-4.980222	-0.782381	-0.067099
O	-3.200306	-2.172541	1.470376

B3LYP Energy = -1303.78586531 a.u.

3, Conf E

C	0.282484	1.221785	0.730086
H	0.338628	1.440702	1.805686
C	2.160905	-1.237550	-1.334106
H	2.027612	-1.306445	-2.417517
C	3.615247	-0.898571	-0.985535
H	3.920139	-0.043174	-1.590114
C	-0.300339	3.421542	-0.042550
H	-0.640023	3.630401	0.981740
C	3.737463	-0.525253	0.490844
H	3.520275	-1.407616	1.104421
C	-1.481706	2.932252	-0.871574
H	-2.290852	3.666379	-0.837997
H	-1.166110	2.851734	-1.917959
C	2.746822	0.573387	0.850584
H	3.003698	1.479515	0.293372
C	-3.283016	1.176240	-0.610244
H	-3.950183	1.793567	-1.201217
C	-1.973762	1.599908	-0.358183
C	1.330114	0.136260	0.464611
H	1.073129	-0.740347	1.065419
C	-3.776407	-0.013524	-0.106931
C	-2.948531	-0.818982	0.700616
C	-1.643251	-0.407910	0.945630
H	-1.008427	-1.015092	1.574985
C	0.372702	4.654677	-0.613726
H	0.754984	4.451884	-1.616552
H	1.204774	4.967503	0.018751
H	-0.340266	5.478838	-0.676895
C	-1.142525	0.784630	0.407888
C	0.703494	-3.128799	-1.227907
H	-0.192039	-2.542329	-1.014759
H	0.639081	-4.087757	-0.718598

H	0.784514	-3.293636	-2.306520
C	-5.300928	-1.436320	-1.250961
H	-6.378271	-1.565107	-1.334242
H	-4.850432	-2.357075	-0.878035
H	-4.886929	-1.192283	-2.233154
C	-2.724014	-2.785910	2.056541
H	-2.414336	-2.246689	2.955249
H	-1.842418	-3.170132	1.537635
H	-3.367977	-3.615530	2.336915
H	4.087756	-2.780014	-1.009347
H	5.677421	-0.734062	0.595713
H	3.683080	0.987594	2.503199
O	1.876480	-2.476739	-0.729830
O	0.684623	2.378949	0.005683
O	4.498287	-1.962712	-1.327874
O	5.043623	-0.034783	0.800707
O	2.762049	0.837202	2.253348
O	1.284573	-0.203737	-0.929723
O	-5.093704	-0.348951	-0.334142
O	-3.510350	-1.956480	1.200496

B3LYP Energy = -1303.78559439 a.u.

3, Conf F

C	0.215995	1.157629	0.707310
H	0.197742	1.335485	1.791723
C	2.337985	-1.116469	-1.333090
H	2.276095	-1.141058	-2.424763
C	3.749261	-0.725154	-0.878069
H	4.047819	0.169918	-1.425672
C	-0.430269	3.355944	-0.017515
H	-0.839812	3.507368	0.991236
C	3.761394	-0.412734	0.617254
H	3.550788	-1.330634	1.178860
C	-1.534770	2.843731	-0.934192
H	-2.379825	3.536916	-0.923477
H	-1.154451	2.819056	-1.961677
C	2.697366	0.621453	0.957358
H	2.942989	1.561782	0.454279
C	-3.262627	0.999418	-0.837461
H	-3.924791	1.607551	-1.443459
C	-1.989362	1.470611	-0.499902
C	1.330513	0.135090	0.465368
H	1.081397	-0.777718	1.013161
C	-3.722631	-0.233358	-0.411997
C	-2.895589	-1.039659	0.395175
C	-1.632218	-0.573010	0.740126
H	-0.999141	-1.182097	1.369297
C	0.212000	4.641922	-0.500821
H	-0.536620	5.432814	-0.575962
H	0.661779	4.498374	-1.485689
H	0.988934	4.968656	0.191917
C	-1.164318	0.665952	0.284017
C	0.980154	-3.080845	-1.411914
H	0.044022	-2.543312	-1.250642

H	0.922806	-4.059483	-0.940654
H	1.151206	-3.202667	-2.485702
C	-5.984534	-0.658466	0.188111
H	-6.143001	0.358611	0.557064
H	-5.725598	-1.316888	1.018334
H	-6.894118	-1.015470	-0.290835
C	-2.623565	-3.081711	1.626067
H	-3.227547	-3.967044	1.807642
H	-2.404944	-2.589591	2.577128
H	-1.688891	-3.373854	1.141261
H	4.314637	-2.579395	-0.951973
H	5.699443	-0.533439	0.836945
H	3.509070	1.006086	2.681375
O	2.079269	-2.394766	-0.803125
O	0.601564	2.361027	0.052268
O	4.703685	-1.730074	-1.207574
O	5.019697	0.124785	1.030162
O	2.613429	0.824983	2.367724
O	1.386604	-0.146192	-0.941463
O	-4.962851	-0.672289	-0.823067
O	-3.408735	-2.243978	0.776992

B3LYP Energy = -1303.78556961 a.u.

3, Conf G

C	-0.199054	1.324069	-0.822533
H	-0.314658	1.439400	-1.908733
C	-3.008121	-1.102948	-0.877373
H	-3.012862	-1.958157	-1.559033
C	-3.057232	-1.563983	0.579959
H	-2.280596	-2.316783	0.722294
C	1.036537	3.365719	-0.453146
H	1.246281	3.339666	-1.530027
C	-2.796026	-0.400793	1.539856
H	-3.623175	0.315301	1.465219
C	2.163864	2.675847	0.301170
H	3.119421	3.138278	0.042058
H	2.017528	2.831545	1.375951
C	-1.507530	0.325373	1.179290
H	-0.663738	-0.345565	1.379185
C	3.386267	0.481044	0.196156
H	4.242865	1.006364	0.596205
C	2.203136	1.198628	-0.016194
C	-1.515772	0.698522	-0.314168
H	-2.290891	1.455552	-0.470359
C	3.482959	-0.869054	-0.119515
C	2.358265	-1.516687	-0.668169
C	1.192015	-0.805142	-0.880769
H	0.351795	-1.327821	-1.315195
C	0.790899	4.795752	-0.015128
H	1.689749	5.394569	-0.172199
H	0.533382	4.834766	1.045527
H	-0.023262	5.240492	-0.588730
C	1.084849	0.551026	-0.545077
C	-4.429516	-0.036471	-2.472955

H	-5.350420	0.541764	-2.492629
H	-4.574789	-0.973383	-3.018999
H	-3.628839	0.538104	-2.942861
C	5.770579	-1.010088	0.585833
H	5.582708	-0.631402	1.593447
H	6.125082	-0.196410	-0.051527
H	6.524788	-1.791666	0.627308
C	2.411539	-3.797281	0.003613
H	1.489207	-3.712118	0.584680
H	3.274247	-3.668678	0.658784
H	2.453859	-4.777895	-0.466360
H	-4.998436	-1.642495	0.545668
H	-3.482224	-1.421374	3.041572
H	-0.856724	2.133567	1.505527
O	-4.137522	-0.296745	-1.095864
O	-0.180557	2.627087	-0.223960
O	-4.290914	-2.208661	0.887101
O	-2.695647	-0.883189	2.879821
O	-1.405516	1.495140	1.991741
O	-1.786264	-0.437728	-1.146186
O	4.603596	-1.625972	0.037979
O	2.423291	-2.835786	-1.064618

B3LYP Energy = -1303.78528919 a.u.

3, Conf H

C	-0.221632	1.184751	-0.903218
H	-0.299372	1.154123	-1.998429
C	-2.997147	-1.275908	-0.741106
H	-2.967719	-2.212492	-1.305174
C	-3.097686	-1.544639	0.761105
H	-2.321484	-2.263326	1.027899
C	0.967196	3.281861	-0.765880
H	1.215579	3.118304	-1.822284
C	-2.880502	-0.264215	1.571418
H	-3.709025	0.426809	1.374893
C	2.077661	2.720885	0.110433
H	3.034590	3.163039	-0.177237
H	1.891943	3.015872	1.149350
C	-1.584606	0.423868	1.166127
H	-0.743943	-0.207887	1.476374
C	3.330985	0.554572	0.354874
H	4.158612	1.143133	0.726798
C	2.150160	1.215437	-0.003714
C	-1.545859	0.604835	-0.361893
H	-2.325336	1.321891	-0.638556
C	3.454686	-0.824952	0.238625
C	2.369241	-1.559161	-0.279411
C	1.204511	-0.904184	-0.634124
H	0.388989	-1.496075	-1.024765
C	0.683212	4.751777	-0.529626
H	1.577147	5.343186	-0.735339
H	0.388105	4.924965	0.507555
H	-0.117575	5.099866	-1.183284
C	1.062705	0.481392	-0.481259

C	-4.362957	-0.444158	-2.514186
H	-5.293922	0.102884	-2.644034
H	-4.464674	-1.445652	-2.942750
H	-3.554717	0.087389	-3.020440
C	5.686642	-0.833526	1.114964
H	6.085192	-0.121100	0.388531
H	6.436117	-1.591672	1.326859
H	5.427753	-0.309638	2.038247
C	3.236787	-3.430639	-1.462264
H	2.849734	-3.081910	-2.423682
H	3.172175	-4.516074	-1.418461
H	4.276934	-3.121826	-1.348678
H	-5.035574	-1.650515	0.666209
H	-3.614984	-1.091942	3.165773
H	-0.963705	2.268475	1.278381
O	-4.125307	-0.521974	-1.104690
O	-0.245469	2.555195	-0.481534
O	-4.337404	-2.158757	1.104364
O	-2.827203	-0.568938	2.964862
O	-1.516575	1.687408	1.827781
O	-1.772710	-0.633111	-1.049637
O	4.561176	-1.537717	0.586880
O	2.427089	-2.933081	-0.384144

B3LYP Energy = -1303.78522900 a.u.

3, Conf I

C	-0.132920	1.328031	-0.850240
H	-0.246529	1.375976	-1.941627
C	-2.889210	-1.160700	-0.773467
H	-2.877618	-2.049694	-1.410343
C	-2.926560	-1.546302	0.705921
H	-2.133528	-2.273664	0.885342
C	1.043759	3.422961	-0.599694
H	1.256112	3.339902	-1.673200
C	-2.689251	-0.329080	1.603125
H	-3.531437	0.364452	1.492535
C	2.188553	2.811114	0.194107
H	3.131489	3.281458	-0.095470
H	2.038507	3.030668	1.257288
C	-1.416669	0.404379	1.202852
H	-0.558846	-0.237285	1.436205
C	3.455470	0.630269	0.223237
H	4.319817	1.162373	0.604071
C	2.265825	1.319448	-0.034447
C	-1.434263	0.698415	-0.308138
H	-2.226132	1.428618	-0.502447
C	3.581950	-0.726218	-0.012586
C	2.489024	-1.438898	-0.544427
C	1.300668	-0.763682	-0.796020
H	0.455218	-1.293241	-1.205913
C	0.753614	4.867871	-0.246171
H	1.634442	5.483513	-0.436823
H	0.492111	4.960440	0.810167
H	-0.072087	5.253628	-0.845454

C	1.171492	0.606419	-0.527488
C	-4.333098	-0.206142	-2.418685
H	-5.266876	0.349359	-2.465945
H	-4.457383	-1.172684	-2.915991
H	-3.545974	0.361737	-2.918678
C	4.850554	-2.227427	1.327823
H	4.630219	-1.686129	2.251935
H	5.868130	-2.610908	1.366805
H	4.151382	-3.055906	1.208338
C	1.619558	-3.524991	-1.348645
H	0.752693	-3.534963	-0.683402
H	2.001702	-4.536488	-1.460232
H	1.325515	-3.137339	-2.327167
H	-4.866010	-1.667124	0.683606
H	-3.352596	-1.283333	3.157793
H	-0.808989	2.243011	1.430513
O	-4.034274	-0.389427	-1.030650
O	-0.151809	2.662822	-0.327991
O	-4.145395	-2.200626	1.049358
O	-2.577375	-0.738576	2.966093
O	-1.338469	1.616944	1.952870
O	-1.680759	-0.485714	-1.079011
O	4.797943	-1.337128	0.200122
O	2.692099	-2.764444	-0.791321

B3LYP Energy = -1303.78500171 a.u.

3, Conf J

C	-0.096118	1.251159	-0.852581
H	-0.128680	1.223762	-1.950124
C	-2.875234	-1.212922	-0.813698
H	-2.825010	-2.144392	-1.384632
C	-3.028788	-1.494783	0.681473
H	-2.260633	-2.213818	0.969679
C	1.075408	3.355164	-0.664090
H	1.363143	3.196400	-1.711199
C	-2.844163	-0.220980	1.510034
H	-3.667336	0.469456	1.290554
C	2.155716	2.797259	0.250518
H	3.120260	3.244939	-0.001292
H	1.930707	3.089481	1.282561
C	-1.536789	0.475008	1.157660
H	-0.706049	-0.154779	1.497053
C	3.404946	0.622098	0.512254
H	4.245104	1.178039	0.912641
C	2.238301	1.293226	0.131529
C	-1.439440	0.665349	-0.366778
H	-2.207882	1.384081	-0.668317
C	3.535362	-0.748743	0.387695
C	2.467396	-1.497987	-0.144375
C	1.309392	-0.837598	-0.537540
H	0.484435	-1.395557	-0.951314
C	0.772408	4.822208	-0.434179
H	1.669449	5.420105	-0.604607
H	0.437806	4.990019	0.591808

H	-0.005777	5.167248	-1.116164	H	1.375424	-3.369977	-1.796573
C	1.175703	0.549886	-0.385810	H	-4.962296	-1.602875	0.519614
C	-4.178388	-0.367900	-2.627282	H	-3.633363	-1.061338	3.071580
H	-5.100846	0.186145	-2.785003	H	-0.926971	2.321227	1.305300
H	-4.272367	-1.366247	-3.064813	O	-3.988975	-0.455362	-1.210918
H	-3.350134	0.161180	-3.102806	O	-0.142272	2.619060	-0.426532
C	5.559914	-1.856663	-0.193120	O	-4.278083	-2.115110	0.974775
H	5.067020	-2.622853	-0.792605	O	-2.838553	-0.537994	2.901782
H	6.417839	-2.288580	0.318192	O	-1.500437	1.735378	1.827825
H	5.894385	-1.039090	-0.837513	O	-1.639905	-0.567494	-1.071953
C	1.599462	-3.646780	-0.763401	O	4.685941	-1.359760	0.834757
H	0.693709	-3.563757	-0.157829	O	2.654218	-2.846184	-0.228535
H	1.962055	-4.671104	-0.733653				

B3LYP Energy = -1303.78492462 a.u.

Table S4. Cartesian coordinates and energies of the low-energy conformers calculated at the B3LYP/TZVP PCM/MeOH level.

36, Conf A

C	3.168065	0.439486	-0.974370
C	2.885825	-0.663772	-0.149147
C	1.786440	-0.614486	0.709704
C	0.921519	0.488179	0.719253
C	1.233586	1.587321	-0.081480
C	2.348909	1.564645	-0.916964
C	-0.316139	0.512111	1.589705
O	-0.707113	1.825116	2.026112
C	0.036675	2.968848	1.571833
C	0.397726	2.826629	0.092902
C	-1.568754	-0.117513	0.941688
C	-1.443492	-1.606924	0.586555
C	-2.765421	-2.083874	-0.001992
C	-3.202338	-1.221222	-1.179775
C	-3.184848	0.251615	-0.777483
O	-1.898503	0.592218	-0.254268
O	4.262993	0.318151	-1.771782
O	3.717814	-1.757722	-0.134604
C	4.592470	1.400389	-2.645607
C	3.497482	-2.694592	-1.204266
C	-0.816193	4.187667	1.873923
O	-3.423536	1.026989	-1.901079
H	-0.122032	-0.075719	2.489757
C	-3.769555	2.387592	-1.625238
O	-4.518012	-1.564978	-1.602429
O	-1.165071	-2.429972	1.715302
O	-2.683791	-3.427531	-0.476947
H	-2.377053	0.003610	1.675588
O	1.528602	-1.693648	1.539957
C	2.461117	-1.867683	2.631387
H	2.577873	2.439597	-1.508949
H	0.969854	3.031561	2.147869
H	-0.523970	2.764289	-0.490966
H	0.950464	3.707551	-0.238878
H	-0.661017	-1.726224	-0.169829
H	-3.532316	-2.020205	0.781730
H	-2.489214	-1.351334	-2.002562
H	-3.946371	0.442430	-0.004762
H	5.475575	1.078966	-3.191583
H	4.823852	2.307463	-2.082380
H	3.781324	1.601239	-3.349395
H	4.208765	-3.504000	-1.053045
H	2.479692	-3.089881	-1.164921
H	3.676881	-2.226174	-2.173418
H	-1.742429	4.165041	1.296399
H	-1.070297	4.222297	2.934527
H	-0.270995	5.099725	1.623444
H	-4.636982	2.438349	-0.959893
H	-4.020837	2.843047	-2.580634
H	-2.934533	2.926844	-1.174593
H	-4.536699	-2.524523	-1.714448
H	-0.202656	-2.385709	1.846305
H	-2.394509	-3.975299	0.264583
H	3.460543	-2.063184	2.247638
H	2.464792	-0.980132	3.267311
H	2.106766	-2.725120	3.199244

B3LYP Energy = -1418.33926476 a.u.

36, Conf B

C	3.162815	0.447622	-0.988205
C	2.892063	-0.650272	-0.152178
C	1.796724	-0.601183	0.712167
C	0.923666	0.495191	0.715057
C	1.224952	1.589899	-0.095926
C	2.336725	1.567968	-0.936145
C	-0.310635	0.517479	1.590230
O	-0.707874	1.831646	2.018343
C	0.025111	2.976926	1.551116
C	0.382268	2.825519	0.072145
C	-1.563340	-0.121308	0.949346
C	-1.434181	-1.624643	0.631882
C	-2.734619	-2.128778	0.015867
C	-3.167319	-1.243978	-1.148647
C	-3.181536	0.226219	-0.742249
O	-1.895882	0.588527	-0.245682
O	4.254173	0.326463	-1.790709
O	3.731486	-1.738648	-0.134155
C	4.572960	1.404198	-2.673884
C	3.503492	-2.690170	-1.189108
C	-0.836416	4.191635	1.845425
O	-3.466474	0.978157	-1.874874
H	-0.109934	-0.061145	2.494100
C	-3.845943	2.334844	-1.618981
O	-4.455780	-1.698521	-1.557477
O	-1.164044	-2.379240	1.806762
O	-2.549585	-3.472814	-0.424417
H	-2.369636	0.006547	1.684306
O	1.549663	-1.673484	1.553670
C	2.495509	-1.839332	2.634774
H	2.557284	2.439619	-1.536140
H	0.959816	3.052187	2.123242
H	-0.540806	2.753915	-0.508373
H	0.929103	3.707162	-0.267524
H	-0.633824	-1.766080	-0.101805
H	-3.516090	-2.097798	0.787201
H	-2.445441	-1.353696	-1.965895
H	-3.935125	0.405864	0.040879
H	4.801755	2.316706	-2.118390
H	3.757259	1.595335	-3.375142
H	5.455273	1.084050	-3.221903

H	3.667728	-2.232733	-2.166237
H	4.221730	-3.493269	-1.037061
H	2.488829	-3.091215	-1.132019
H	-1.763880	4.156748	1.270401
H	-1.088081	4.233135	2.906367
H	-0.299115	5.105858	1.585884
H	-4.701467	2.374090	-0.938132
H	-4.126437	2.764543	-2.578035
H	-3.016442	2.902938	-1.194752
H	-4.696098	-1.227183	-2.364931
H	-0.202451	-2.330952	1.934070
H	-3.358405	-3.730616	-0.885949
H	3.491193	-2.033478	2.240912
H	2.503560	-0.948872	3.266722
H	2.150445	-2.695350	3.210399

B3LYP Energy = -1418.33871277 a.u.

36, Conf C

C	-3.819479	-0.144760	0.240916
C	-2.970629	-1.188627	-0.166923
C	-1.667794	-0.897063	-0.571655
C	-1.161329	0.410973	-0.514044
C	-2.026066	1.440678	-0.143914
C	-3.341951	1.161129	0.232351
C	0.274795	0.697769	-0.932738
O	0.610572	2.082938	-0.902941
C	-0.437814	2.994478	-1.256665
C	-1.551472	2.870880	-0.223660
C	1.346079	-0.035421	-0.101311
C	1.659883	0.581936	1.267071
C	2.832065	-0.150931	1.916002
C	4.032779	-0.228620	0.986180
C	3.591990	-0.811308	-0.354518
O	2.544166	-0.015787	-0.897283
O	-5.079759	-0.509870	0.606287
O	-3.436004	-2.482265	-0.229429
C	-5.983398	0.509446	1.036345
C	-3.401161	-3.188615	1.022335
C	0.177852	4.379064	-1.324737
O	4.681200	-0.779931	-1.216368
H	0.408894	0.325579	-1.957619
C	4.522386	-1.551977	-2.412121
O	5.013595	-1.041799	1.624834
O	0.506851	0.488050	2.101052
O	3.134701	0.547306	3.126137
H	1.020276	-1.069541	0.032343
O	-0.838889	-1.917284	-0.989596
C	-1.169097	-2.502206	-2.262998
H	-3.991437	1.982712	0.500645
H	-0.831984	2.720650	-2.245196
H	-1.168916	3.211702	0.744618
H	-2.386595	3.523463	-0.489125
H	1.936740	1.629800	1.121879
H	2.512706	-1.173319	2.155325
H	4.423804	0.778634	0.804686
H	3.232523	-1.844997	-0.223402
H	-6.174780	1.231903	0.239159
H	-5.603300	1.029793	1.918886
H	-6.908091	-0.003436	1.288343
H	-2.377558	-3.253827	1.398938
H	-4.037553	-2.699434	1.762259
H	-3.779621	-4.189053	0.821610
H	0.597020	4.660560	-0.356344
H	0.974422	4.408856	-2.069614
H	-0.579009	5.115558	-1.600804
H	4.289882	-2.593628	-2.170510
H	5.472791	-1.506266	-2.939104
H	3.734514	-1.139458	-3.044552
H	5.803162	-1.046389	1.069201
H	0.780464	0.768426	2.984575
H	3.813967	0.048374	3.596699
H	-2.144941	-2.985534	-2.227762
H	-1.157481	-1.740990	-3.047244
H	-0.397182	-3.241952	-2.464929

B3LYP Energy = -1418.33782985 a.u.

36, Conf D

C	-3.787013	-0.109834	0.223757
C	-2.970393	-1.124156	-0.310899
C	-1.670377	-0.820774	-0.711367
C	-1.131774	0.465448	-0.528411
C	-1.967555	1.472216	-0.038869
C	-3.281479	1.179386	0.339322
C	0.310637	0.763324	-0.925383
O	0.671619	2.126459	-0.718816
C	-0.348600	3.092312	-1.007588
C	-1.478532	2.902088	-0.003780
C	1.356435	-0.081212	-0.172173
C	1.539450	0.285777	1.309441
C	2.706238	-0.485322	1.930013
C	3.965344	-0.360530	1.090868
C	3.640886	-0.709635	-0.363194
O	2.602739	0.126241	-0.855026
O	-5.044898	-0.485576	0.577306
O	-3.465955	-2.392768	-0.496084
C	-5.918426	0.500222	1.132524
C	-3.440446	-3.222466	0.679010
C	0.297817	4.462627	-0.944669
O	4.792512	-0.495276	-1.111915
H	0.438344	0.517051	-1.988096
C	4.768894	-1.064796	-2.425725
O	4.953878	-1.243338	1.610332
O	0.404566	-0.060735	2.111679
O	2.974796	-0.007470	3.246621
H	1.065332	-1.131140	-0.253533
O	-0.872302	-1.805935	-1.248648
C	-1.241144	-2.259701	-2.565365
H	-3.908328	1.982425	0.700948

H	-0.732062	2.913435	-2.021349
H	-1.112392	3.173441	0.992483
H	-2.307067	3.575987	-0.233740
H	1.743832	1.356341	1.385367
H	2.427005	-1.546637	1.974242
H	4.320455	0.676404	1.116749
H	3.324727	-1.763051	-0.437394
H	-6.111340	1.304705	0.418951
H	-5.507873	0.917652	2.054987
H	-6.847464	-0.019656	1.351263
H	-2.417114	-3.342016	1.042332
H	-4.067010	-2.799609	1.466368
H	-3.836290	-4.190595	0.379010
H	0.713109	4.645535	0.048671
H	1.102224	4.541424	-1.677256
H	-0.439911	5.238074	-1.158726
H	4.570286	-2.139689	-2.376207
H	5.754819	-0.897986	-2.853658
H	4.014347	-0.582351	-3.049244
H	5.723028	-1.178713	1.029147
H	-0.336031	0.512360	1.878476
H	2.133095	-0.023174	3.721575
H	-1.231628	-1.426196	-3.271964
H	-0.486961	-2.988595	-2.854150
H	-2.224547	-2.728097	-2.553919

B3LYP Energy = -1418.33781119 a.u.

36, Conf E

C	-3.784652	-0.100646	0.222495
C	-2.973848	-1.099375	-0.349144
C	-1.672723	-0.788500	-0.740101
C	-1.126858	0.487393	-0.511019
C	-1.957078	1.480470	0.015185
C	-3.272309	1.180983	0.383611
C	0.317076	0.791784	-0.898318
O	0.684835	2.144623	-0.643014
C	-0.330293	3.125348	-0.896428
C	-1.460730	2.905616	0.100827
C	1.358545	-0.083681	-0.175081
C	1.526094	0.212559	1.325320
C	2.691436	-0.599520	1.877637
C	3.966779	-0.419481	1.064386
C	3.668814	-0.692674	-0.406191
O	2.608369	0.158315	-0.836866
O	-5.043898	-0.482421	0.564254
O	-3.475888	-2.358230	-0.578259
C	-5.911072	0.486778	1.157480
C	-3.453479	-3.228520	0.567220
C	0.323482	4.489143	-0.785349
O	4.795614	-0.403615	-1.160709
H	0.443004	0.583145	-1.969283
C	4.740356	-0.873525	-2.511373
O	4.977229	-1.318199	1.509934
O	0.390807	-0.184552	2.101911
O	3.005161	-0.238347	3.224066
H	1.069552	-1.129450	-0.305233
O	-0.880351	-1.757738	-1.313288
C	-1.252787	-2.162042	-2.645045
H	-3.894854	1.973715	0.774183
H	-0.715407	2.984889	-1.915661
H	-1.092899	3.139605	1.105859
H	-2.285989	3.591332	-0.104852
H	1.727404	1.277920	1.460567
H	2.409923	-1.660219	1.842672
H	4.310638	0.618112	1.150199
H	3.367963	-1.744006	-0.544442
H	-6.842021	-0.036249	1.359951
H	-6.102008	1.317543	0.474145
H	-5.495877	0.868537	2.093236
H	-2.430492	-3.365108	0.925486
H	-4.077884	-2.830648	1.369182
H	-3.853679	-4.183867	0.233888
H	0.740515	4.634333	0.213477
H	1.127740	4.589599	-1.515442
H	-0.410216	5.275677	-0.970918
H	4.551850	-1.951512	-2.537846
H	5.713833	-0.667286	-2.950901
H	3.966637	-0.355109	-3.080593
H	5.019802	-1.236355	2.472001
H	-0.362484	0.372337	1.869304
H	2.220195	-0.404971	3.761234
H	-0.502742	-2.884107	-2.960504
H	-2.238645	-2.625374	-2.649277
H	-1.239406	-1.303825	-3.321350

B3LYP Energy = -1418.33735128 a.u.

36, Conf F

C	-3.795346	-0.082060	0.239126
C	-2.990006	-1.116344	-0.274639
C	-1.687228	-0.835489	-0.682077
C	-1.134718	0.448910	-0.525101
C	-1.959128	1.474590	-0.055029
C	-3.275993	1.203807	0.329075
C	0.308638	0.722394	-0.935956
O	0.682148	2.089054	-0.779984
C	-0.335361	3.055713	-1.074791
C	-1.452005	2.898000	-0.050234
C	1.355238	-0.104847	-0.161393
C	1.523914	0.302373	1.318878
C	2.723817	-0.404705	1.938454
C	3.962154	-0.301794	1.056720
C	3.638054	-0.747144	-0.364015
O	2.589414	0.067621	-0.872619
O	-5.056548	-0.437175	0.600599
O	-3.499504	-2.383005	-0.432159
C	-5.918800	0.568061	1.138680
C	-3.481639	-3.187827	0.760386
C	0.322753	4.421754	-1.052307

O	4.774751	-0.566598	-1.142221
H	0.428916	0.437664	-1.989627
C	4.726760	-1.193673	-2.428738
O	4.964860	-1.115407	1.661749
O	0.384069	-0.077251	2.094439
O	2.975917	0.163269	3.223472
H	1.054847	-1.154660	-0.205161
O	-0.898135	-1.838246	-1.198202
C	-1.277082	-2.326208	-2.499986
H	-3.893764	2.020549	0.675203
H	-0.735868	2.857831	-2.078392
H	-1.065987	3.179806	0.935410
H	-2.275111	3.578918	-0.278634
H	1.682557	1.382278	1.375714
H	2.471349	-1.467624	2.049797
H	4.290738	0.742528	1.010771
H	3.321916	-1.802924	-0.379857
H	-6.104368	1.360991	0.410432
H	-5.502185	0.998072	2.052558
H	-6.852823	0.062071	1.368396
H	-2.459228	-3.311004	1.124996
H	-4.102377	-2.741363	1.539233
H	-3.888628	-4.157582	0.481342
H	0.753025	4.624061	-0.069176
H	1.117646	4.476951	-1.797348
H	-0.411688	5.198068	-1.274392
H	4.532580	-2.265856	-2.328073
H	5.703641	-1.044158	-2.883085
H	3.958815	-0.741181	-3.058430
H	5.786272	-0.990429	1.170300
H	-0.389774	0.389539	1.753961
H	3.777467	-0.256138	3.563002
H	-2.261853	-2.790662	-2.469753
H	-1.269136	-1.511921	-3.228625
H	-0.526884	-3.064997	-2.773480

B3LYP Energy = -1418.33731005 a.u.

36, Conf G

C	-3.792092	-0.345057	0.337348
C	-2.820370	-1.326697	0.071173
C	-1.553093	-0.939004	-0.362171
C	-1.200659	0.416952	-0.472670
C	-2.188346	1.377659	-0.248787
C	-3.469156	0.994999	0.159475
C	0.205281	0.815418	-0.910920
O	0.385618	2.234251	-0.902346
C	-0.709482	2.984329	-1.454787
C	-1.898446	2.837183	-0.514982
C	1.341962	0.205492	-0.049350
C	2.038648	1.198851	0.888735
C	3.136574	0.492522	1.681819
C	4.098773	-0.213382	0.740102
C	3.318847	-1.081122	-0.253857
O	2.338950	-0.313134	-0.944629
O	-5.006690	-0.805025	0.743223
O	-3.131458	-2.662430	0.172293
C	-6.032068	0.147407	1.031838
C	-3.044197	-3.196290	1.504829
C	-0.239456	4.415451	-1.626341
O	4.234216	-1.584888	-1.170678
H	0.372607	0.447408	-1.930837
C	3.748609	-2.668908	-1.970892
O	4.991310	-1.014501	1.507106
O	1.144171	1.769599	1.843600
O	3.865362	1.428901	2.472359
H	0.936046	-0.617291	0.542387
O	-0.605073	-1.898691	-0.642958
C	-0.835858	-2.666528	-1.838915
H	-4.214799	1.762097	0.315616
H	-0.963702	2.559729	-2.434821
H	-1.680509	3.369565	0.417990
H	-2.781123	3.312678	-0.949306
H	2.497902	1.987290	0.288448
H	2.670941	-0.258243	2.334495
H	4.652187	0.538624	0.165557
H	2.816264	-1.903435	0.280323
H	-6.291728	0.730510	0.145048
H	-5.731872	0.820241	1.838789
H	-6.894370	-0.434493	1.346855
H	-2.028738	-3.092680	1.894521
H	-3.749925	-2.696147	2.170712
H	-3.299641	-4.251447	1.430143
H	0.058063	4.839891	-0.664978
H	0.612484	4.461739	-2.305938
H	-1.042973	5.028115	-2.038983
H	3.396119	-3.488045	-1.336947
H	4.588442	-3.011120	-2.571379
H	2.940532	-2.341393	-2.626988
H	5.577026	-1.463477	0.883582
H	0.611827	2.415866	1.360555
H	3.208269	1.947051	2.956643
H	-0.889975	-2.009205	-2.710303
H	0.018742	-3.332008	-1.939810
H	-1.752170	-3.250125	-1.755019

B3LYP Energy = -1418.33709406 a.u.

36, Conf H

C	-3.790560	-0.338191	0.343394
C	-2.817894	-1.319750	0.080737
C	-1.550207	-0.932385	-0.351827
C	-1.198306	0.423492	-0.465393
C	-2.186813	1.384104	-0.244159
C	-3.467949	1.001631	0.163358
C	0.207773	0.822209	-0.903603
O	0.387452	2.241247	-0.893437
C	-0.706507	2.990680	-1.448670
C	-1.897538	2.843540	-0.511584
C	1.345599	0.212165	-0.043021

C	2.055252	1.209482	0.883442
C	3.141649	0.471266	1.652368
C	4.101529	-0.245247	0.707874
C	3.318978	-1.113553	-0.275434
O	2.330686	-0.323464	-0.939242
O	-5.005547	-0.798022	0.748412
O	-3.128556	-2.655440	0.184242
C	-6.032524	0.154308	1.031471
C	-3.040897	-3.186983	1.517615
C	-0.236775	4.421937	-1.619998
O	4.193432	-1.613651	-1.228190
H	0.374825	0.455775	-1.924120
C	3.652961	-2.663292	-2.037007
O	5.003731	-1.073806	1.433828
O	1.173205	1.785170	1.845962
O	3.932157	1.355146	2.449108
H	0.936691	-0.599788	0.561763
O	-0.601753	-1.892701	-0.628696
C	-0.830247	-2.662195	-1.823981
H	-4.214079	1.768725	0.317211
H	-0.958271	2.565455	-2.429098
H	-1.682081	3.376979	0.421339
H	-2.779638	3.317773	-0.948486
H	2.517145	1.992733	0.278573
H	2.665243	-0.276120	2.300406
H	4.652526	0.503197	0.126016
H	2.816709	-1.934439	0.261196
H	-6.290225	0.734656	0.142301
H	-5.735223	0.829739	1.837330
H	-6.895197	-0.427450	1.345751
H	-2.025377	-3.082544	1.906939
H	-3.746590	-2.685851	2.182823
H	-3.296142	-4.242326	1.444837
H	0.058611	4.846980	-0.658242
H	0.616515	4.468293	-2.297899
H	-1.039732	5.034053	-2.034556
H	3.292093	-3.486019	-1.411802
H	4.466034	-3.018800	-2.666220
H	2.837925	-2.300125	-2.665504
H	5.382428	-0.529262	2.136516
H	0.631734	2.425014	1.364405
H	3.335547	1.808138	3.058743
H	-0.878139	-2.006522	-2.696977
H	0.022138	-3.331188	-1.919859
H	-1.749066	-3.242231	-1.742778

B3LYP Energy = -1418.33654373 a.u.

36, Conf I

C	-3.744937	0.015713	0.490733
C	-2.959822	-1.125290	0.235180
C	-1.666016	-0.959643	-0.268177
C	-1.126149	0.318508	-0.489586
C	-1.945329	1.430321	-0.306206
C	-3.243969	1.274894	0.182039
C	0.299169	0.465206	-1.004806
O	0.665974	1.812674	-1.295082
C	-0.378079	2.663000	-1.786707
C	-1.442019	2.796041	-0.703699
C	1.382910	-0.093877	-0.061395
C	1.742514	0.794479	1.136109
C	2.925739	0.197537	1.894941
C	4.098641	-0.093255	0.972335
C	3.614120	-0.942951	-0.200150
O	2.558065	-0.267347	-0.872897
O	-4.971044	-0.211089	1.042376
O	-3.391840	-2.390339	0.553894
C	-5.801556	0.911221	1.343870
C	-4.551823	-2.879955	-0.145546
C	0.265020	3.981448	-2.172852
O	4.678227	-1.111546	-1.077686
H	0.389307	-0.129956	-1.923493
C	4.480648	-2.121651	-2.073348
O	5.091030	-0.764809	1.743835
O	0.613882	0.896505	2.001958
O	3.268591	1.131125	2.921867
H	1.046402	-1.068718	0.298737
O	-0.851704	-2.047289	-0.495030
C	-1.261076	-2.975043	-1.513274
H	-3.856570	2.154595	0.321958
H	-0.827428	2.200983	-2.676772
H	-1.004392	3.319058	0.153689
H	-2.273294	3.406304	-1.064786
H	2.024740	1.783723	0.765110
H	2.603501	-0.746297	2.353120
H	4.492220	0.846377	0.568964
H	3.251044	-1.919760	0.158965
H	-6.703029	0.498937	1.789892
H	-6.064558	1.465369	0.439614
H	-5.316096	1.581894	2.056844
H	-4.657523	-3.923043	0.145645
H	-5.444190	-2.323775	0.135725
H	-4.404097	-2.817420	-1.226689
H	0.739985	4.445018	-1.305507
H	1.022565	3.828600	-2.942948
H	-0.488003	4.668734	-2.562850
H	4.251357	-3.084102	-1.605897
H	5.415052	-2.200681	-2.624491
H	3.675942	-1.847297	-2.757555
H	5.864259	-0.898945	1.181435
H	0.917854	1.347570	2.800877
H	3.955260	0.732511	3.470809
H	-1.554094	-2.444586	-2.422376
H	-0.391814	-3.595846	-1.721263
H	-2.081770	-3.602544	-1.169402

B3LYP Energy = -1418.33632650 a.u.

36, Conf J

C	-3.791828	-0.352571	0.341770

C	-2.811752	-1.326869	0.080009
C	-1.545423	-0.930195	-0.348132
C	-1.201971	0.428206	-0.457963
C	-2.198048	1.381499	-0.238209
C	-3.477660	0.989621	0.165078
C	0.202249	0.838448	-0.892772
O	0.371477	2.258578	-0.875106
C	-0.725283	3.002485	-1.432949
C	-1.919036	2.843789	-0.501437
C	1.346796	0.227918	-0.039099
C	2.048095	1.228484	0.899365
C	3.155537	0.510815	1.658954
C	4.081913	-0.220810	0.691186
C	3.290399	-1.104781	-0.268755
O	2.316067	-0.317176	-0.947207
O	-5.004882	-0.821351	0.742257
O	-3.113781	-2.664843	0.179573
C	-6.039040	0.123574	1.024074
C	-3.026740	-3.198738	1.512063
C	-0.264596	4.437415	-1.597687
O	4.181992	-1.629849	-1.195896
H	0.371082	0.479823	-1.915618
C	3.663488	-2.705647	-1.986049
O	4.993540	-0.977673	1.485053
O	1.128546	1.774030	1.840884
O	3.891565	1.464065	2.423506
H	0.939182	-0.582841	0.568144
O	-0.590834	-1.884686	-0.624304
C	-0.810055	-2.649102	-1.824445
H	-4.229270	1.751464	0.318289
H	-0.969407	2.578272	-2.415697
H	-1.712062	3.376915	0.433606
H	-2.802959	3.311963	-0.941128
H	2.496572	2.025846	0.302090
H	2.698583	-0.227613	2.331327
H	4.621484	0.519368	0.089702
H	2.780961	-1.913080	0.279803
H	-6.898774	-0.464543	1.334553
H	-6.297887	0.703977	0.135265
H	-5.748700	0.799338	1.832181
H	-3.274373	-4.255677	1.436118
H	-2.013269	-3.088010	1.904946
H	-3.738059	-2.704005	2.176059
H	0.023693	4.861429	-0.633367
H	0.591238	4.491741	-2.271775
H	-1.070014	5.045205	-2.013850
H	3.296037	-3.512696	-1.345043
H	4.489848	-3.071928	-2.591102
H	2.858533	-2.362348	-2.637987
H	5.666090	-1.346166	0.898844
H	0.568543	2.378420	1.334747
H	4.638964	0.992464	2.814310
H	-1.729366	-3.229640	-1.752673
H	-0.851394	-1.989859	-2.695108
H	0.042933	-3.317830	-1.916721

B3LYP Energy = -1418.33630293 a.u.

36, Conf K

C	-3.811765	-0.075252	0.431635
C	-3.006456	-1.167162	0.061095
C	-1.693045	-0.945320	-0.357555
C	-1.166793	0.358294	-0.435167
C	-2.005922	1.432481	-0.141304
C	-3.311186	1.214364	0.305411
C	0.250107	0.588183	-0.949044
O	0.595555	1.966138	-1.075800
C	-0.460331	2.852513	-1.465961
C	-1.520011	2.842536	-0.372046
C	1.368745	-0.061006	-0.110625
C	1.750381	0.681356	1.176376
C	2.965733	0.020546	1.823699
C	4.114015	-0.134744	0.839144
C	3.606741	-0.846746	-0.412712
O	2.520607	-0.114210	-0.969111
O	-5.070365	-0.381968	0.852582
O	-3.511030	-2.447165	0.121781
C	-5.946654	0.689154	1.206298
C	-4.323862	-2.819748	-1.004574
C	0.162738	4.215709	-1.699226
O	4.645193	-0.890620	-1.334182
H	0.327634	0.114729	-1.937097
C	4.429241	-1.776367	-2.438781
O	5.140084	-0.875138	1.494730
O	0.646195	0.656254	2.078450
O	3.324900	0.831132	2.945029
H	1.063558	-1.078153	0.140479
O	-0.903589	-1.979283	-0.803918
C	-0.682931	-3.105625	0.067019
H	-3.933521	2.068553	0.532593
H	-0.906168	2.489345	-2.402442
H	-1.083868	3.263307	0.540515
H	-2.360587	3.481237	-0.653078
H	2.005879	1.713119	0.920317
H	2.673620	-0.977544	2.174448
H	4.480309	0.853813	0.540753
H	3.268207	-1.865778	-0.163579
H	-6.138904	1.343396	0.352338
H	-5.540460	1.276402	2.033363
H	-6.875870	0.219180	1.518210
H	-5.204401	-2.178898	-1.076756
H	-3.747427	-2.763654	-1.931262
H	-4.635763	-3.847939	-0.831492
H	0.631399	4.585260	-0.784581
H	0.921807	4.162367	-2.481051
H	-0.600867	4.931373	-2.009491
H	4.225974	-2.791502	-2.084325
H	5.347185	-1.773761	-3.022227
H	3.600567	-1.433987	-3.060823
H	5.896796	-0.926734	0.897297
H	0.965442	1.015678	2.916932

H	4.033196	0.382475	3.423323
H	-1.554287	-3.754891	0.103140
H	0.168022	-3.639851	-0.351320
H	-0.440342	-2.767644	1.077255

B3LYP Energy = -1418.33629345 a.u.

36, Conf L

C	-3.754476	-0.039207	0.432440
C	-3.000728	-1.113088	-0.080504
C	-1.695773	-0.890864	-0.520160
C	-1.127768	0.400380	-0.477561
C	-1.922891	1.467419	-0.054916
C	-3.219498	1.241862	0.417082
C	0.295281	0.632323	-0.978456
O	0.679358	2.005638	-0.954042
C	-0.348291	2.953174	-1.271630
C	-1.409391	2.883104	-0.181668
C	1.376324	-0.125660	-0.183005
C	1.604102	0.387534	1.249633
C	2.809029	-0.316181	1.862294
C	4.042706	-0.247053	0.971486
C	3.684066	-0.746453	-0.424233
O	2.591169	0.017807	-0.930044
O	-5.002456	-0.352738	0.871613
O	-3.546928	-2.374437	-0.130030
C	-5.832624	0.699193	1.368792
C	-4.436466	-2.598289	-1.238816
C	0.314695	4.311967	-1.390504
O	4.770260	-0.564430	-1.266419
H	0.365699	0.260648	-2.009143
C	4.660478	-1.232164	-2.527483
O	5.089591	-1.053419	1.501260
O	0.511249	0.091007	2.125339
O	3.176580	0.251049	3.121326
H	1.096148	-1.180432	-0.142529
O	-0.944049	-1.882680	-1.093798
C	-0.810108	-3.152946	-0.425841
H	-3.807889	2.086374	0.746939
H	-0.801383	2.680592	-2.234605
H	-0.970340	3.234565	0.758316
H	-2.240455	3.552002	-0.416615
H	1.787944	1.464083	1.218892
H	2.547039	-1.372971	2.003755
H	4.369490	0.795630	0.880602
H	3.395657	-1.809705	-0.386579
H	-5.376382	1.187035	2.233347
H	-6.762091	0.223024	1.669808
H	-6.037994	1.441462	0.593820
H	-5.296183	-1.928303	-1.188146
H	-3.912000	-2.458604	-2.187267
H	-4.773236	-3.629773	-1.157085
H	0.796411	4.587450	-0.449899
H	1.069871	4.302841	-2.177674
H	-0.427834	5.073916	-1.634492
H	4.493915	-2.304433	-2.383620
H	5.607292	-1.080636	-3.041221
H	3.849634	-0.815526	-3.127649
H	5.172517	-0.824377	2.436581
H	-0.269333	0.578305	1.832711
H	2.423350	0.153056	3.717539
H	-1.660840	-3.798476	-0.629002
H	0.100912	-3.598056	-0.821991
H	-0.710110	-3.012381	0.651870

B3LYP Energy = -1418.33597767 a.u.

36, Conf M

C	3.712258	0.037669	-0.474716
C	2.960999	-1.098879	-0.110327
C	1.669345	-0.926719	0.393085
C	1.095277	0.354360	0.505999
C	1.884514	1.469441	0.221083
C	3.182072	1.305053	-0.271839
C	-0.334144	0.511149	1.013828
O	-0.728313	1.874228	1.152949
C	0.288682	2.777989	1.605629
C	1.367100	2.855026	0.532485
C	-1.395543	-0.154947	0.116724
C	-1.632449	0.541464	-1.234446
C	-2.813366	-0.109817	-1.944423
C	-4.052737	-0.191035	-1.062393
C	-3.685378	-0.859655	0.258312
O	-2.617878	-0.136890	0.868157
O	4.935284	-0.206242	-1.019677
O	3.426097	-2.371557	-0.330505
C	5.736653	0.905009	-1.426788
C	4.601402	-2.774572	0.399945
C	-0.384012	4.107251	1.889360
O	-4.780112	-0.823770	1.108658
H	-0.412571	0.013316	1.989511
C	-4.654256	-1.649126	2.270961
O	-5.075435	-0.947591	-1.701836
O	-0.528326	0.399421	-2.134641
O	-3.190913	0.605987	-3.122193
H	-1.088900	-1.188483	-0.060678
O	0.881942	-2.000554	0.729723
C	1.361925	-2.903338	1.741238
H	3.771081	2.184717	-0.490217
H	0.729333	2.384887	2.532012
H	0.943892	3.333550	-0.357532
H	2.193450	3.484466	0.870783
H	-1.850042	1.597972	-1.060605
H	-2.520484	-1.131730	-2.219144
H	-4.407842	0.821871	-0.838311
H	-3.364592	-1.899473	0.082163
H	5.227252	1.500537	-2.187921
H	6.642081	0.475619	-1.847553
H	5.996157	1.539311	-0.576031
H	5.473286	-2.204355	0.085118

H	4.446438	-2.651058	1.474567
H	4.745762	-3.828783	0.173253
H	-0.856438	4.498710	0.985908
H	-1.148105	3.994277	2.659676
H	0.350995	4.835170	2.237745
H	-4.447349	-2.686137	1.987958
H	-5.609319	-1.601108	2.789548
H	-3.863199	-1.287622	2.930467
H	-5.157018	-0.602802	-2.601087
H	0.228956	0.886567	-1.786807
H	-2.431360	0.608843	-3.718470
H	0.490002	-3.456697	2.084227
H	2.097966	-3.594618	1.335491
H	1.791757	-2.349135	2.578544

B3LYP Energy = -1418.33593096 a.u.

Table S5. Cartesian coordinates and energies of the low-energy conformers calculated at the B3PW91/TZVP PCM/DMSO level.

3, Conf A

C	0.219071	1.136509	0.761388
H	0.265225	1.314751	1.846876
C	2.284660	-1.062717	-1.384434
H	2.163592	-1.097248	-2.472683
C	3.703475	-0.629187	-1.013407
H	3.940961	0.276789	-1.575673
C	-0.551310	3.288767	0.077331
H	-0.911520	3.421345	1.108960
C	3.786998	-0.315550	0.474591
H	3.623312	-1.239331	1.045972
C	-1.682156	2.735940	-0.774237
H	-2.551136	3.397777	-0.715901
H	-1.359766	2.723367	-1.822452
C	2.717298	0.687758	0.864945
H	2.904642	1.629028	0.334730
C	-3.330051	0.837259	-0.582998
H	-4.021229	1.440911	-1.158291
C	-2.056316	1.353482	-0.311805
C	1.346918	0.154401	0.452033
H	1.160807	-0.765946	1.016140
C	-3.722095	-0.406873	-0.122036
C	-2.819198	-1.164181	0.657761
C	-1.561254	-0.652306	0.922112
H	-0.877285	-1.229955	1.530260
C	0.002233	4.599320	-0.435933
H	-0.784636	5.356004	-0.464870
H	0.399580	4.477122	-1.446593
H	0.803408	4.960866	0.211332
C	-1.159484	0.595238	0.426397
C	0.993615	-3.054691	-1.377556
H	1.100620	-3.170550	-2.461416
H	0.051583	-2.546659	-1.158030
H	0.991988	-4.037109	-0.908457
C	-5.875260	-0.238506	-1.117615
H	-6.763445	-0.863941	-1.180736
H	-5.497516	-0.044114	-2.126036
H	-6.134977	0.710009	-0.637694
C	-2.418282	-3.139704	1.922522
H	-2.162080	-2.608762	2.844517
H	-1.500386	-3.417146	1.395711
H	-2.975628	-4.040885	2.170164
H	4.306590	-2.457407	-1.115753
H	5.725887	-0.390281	0.584871
H	3.617103	1.095869	2.525631
O	2.100438	-2.340551	-0.837431
O	0.518566	2.344033	0.084626
O	4.661595	-1.601648	-1.393607
O	5.044479	0.249518	0.821303
O	2.707681	0.903614	2.267834
O	1.331138	-0.124839	-0.947853
O	-4.936657	-0.970884	-0.344971
O	-3.275847	-2.364774	1.099482

B3PW91 Energy = -1303.28174846 a.u.

3, Conf B

C	-0.099816	1.202135	-0.927870
H	-0.197192	1.188781	-2.023671
C	-2.982551	-1.104084	-0.675369
H	-3.026872	-2.048612	-1.228000
C	-3.091271	-1.365698	0.830953
H	-2.360635	-2.134100	1.094171
C	1.187114	3.221285	-0.765555
H	1.426276	3.052745	-1.824715
C	-2.766443	-0.097655	1.607685
H	-3.546788	0.650253	1.411140
C	2.267068	2.599590	0.099889
H	3.244354	2.999998	-0.184038
H	2.097444	2.890884	1.143701
C	-1.434489	0.480501	1.148871
H	-0.639386	-0.231195	1.409757
C	3.410338	0.368072	0.288472
H	4.272351	0.901945	0.668905
C	2.263738	1.100694	-0.043635
C	-1.433633	0.682156	-0.364462
H	-2.185438	1.439230	-0.618071
C	3.468311	-1.004501	0.125972
C	2.345393	-1.677969	-0.404884
C	1.213146	-0.954560	-0.732115
H	0.349660	-1.461750	-1.137084
C	0.981582	4.697933	-0.517721
H	0.699857	4.878598	0.522534
H	0.198092	5.094204	-1.165851
H	1.905472	5.242886	-0.721793
C	1.149041	0.432915	-0.535686
C	-4.329319	-0.220544	-2.418137
H	-4.515045	-1.220090	-2.825544
H	-3.496833	0.240262	-2.955892
H	-5.221049	0.391371	-2.542368
C	5.692966	-1.135728	0.955783
H	5.470361	-0.633053	1.901815
H	6.108083	-0.411902	0.247950
H	6.420905	-1.925319	1.130537
C	1.375443	-3.734494	-1.103487
H	1.134916	-3.389332	-2.113678
H	0.488614	-3.646221	-0.468763
H	1.686025	-4.776607	-1.144128
H	-5.013606	-1.324281	0.720990
H	-2.471518	0.402047	3.450355
H	-0.694245	2.271623	1.284908
O	-4.066630	-0.288046	-1.020842
O	-0.054388	2.557962	-0.485351
O	-4.362634	-1.899312	1.144741

O	-2.729965	-0.406726	2.992859
O	-1.243503	1.702261	1.849932
O	-1.739152	-0.541182	-1.028881
O	4.542063	-1.776782	0.426622
O	2.475019	-3.021008	-0.559755

B3PW91 Energy = -1303.28131607 a.u.

3, Conf C

C	0.305789	1.123029	0.729174
H	0.336040	1.331183	1.809836
C	2.350128	-1.196516	-1.305906
H	2.238057	-1.276830	-2.392689
C	3.773685	-0.774327	-0.939846
H	4.033673	0.102108	-1.537847
C	-0.410580	3.279133	0.018576
H	-0.758177	3.429082	1.052136
C	3.849709	-0.399242	0.534503
H	3.664899	-1.295336	1.142327
C	-1.562067	2.749090	-0.820023
H	-2.416591	3.428547	-0.754713
H	-1.251109	2.726865	-1.871351
C	2.794568	0.637793	0.873410
H	3.003571	1.553380	0.307545
C	-3.238120	0.880100	-0.652179
H	-3.909546	1.497430	-1.236421
C	-1.962808	1.372800	-0.360096
C	1.419078	0.112180	0.468077
H	1.211442	-0.782101	1.065644
C	-3.649954	-0.370342	-0.211372
C	-2.766552	-1.134066	0.574545
C	-1.507060	-0.642504	0.858396
H	-0.861369	-1.263276	1.469863
C	0.170147	4.571364	-0.510474
H	0.557414	4.430343	-1.522585
H	0.984020	4.919466	0.128170
H	-0.598659	5.346303	-0.541281
C	-1.076274	0.598161	0.382576
C	1.016300	-3.158750	-1.218484
H	0.990610	-4.118084	-0.704432
H	1.127402	-3.326753	-2.295070
H	0.084008	-2.621516	-1.029156
C	-5.776966	-0.182120	-1.265172
H	-6.049668	0.759315	-0.779312
H	-6.662445	-0.806913	-1.361130
H	-5.370486	0.024481	-2.259523
C	-4.041304	-2.409675	2.097573
H	-3.619549	-1.906962	2.973554
H	-4.217637	-3.458083	2.334499
H	-4.985825	-1.936265	1.821024
H	4.344441	-2.615854	-0.955040
H	5.785957	-0.502674	0.667066
H	3.686595	1.093777	2.525627
O	2.135226	-2.444246	-0.704086
O	0.634685	2.307230	0.025922
O	4.716484	-1.779913	-1.268603
O	5.113739	0.158260	0.869191
O	2.775768	0.910529	2.266146
O	1.411992	-0.220892	-0.920147
O	-4.858885	-0.925495	-0.476139
O	-3.113181	-2.389008	1.010665

B3PW91 Energy = -1303.27841977 a.u.

3, Conf D

C	0.306269	1.186047	0.718145
H	0.371456	1.453691	1.784045
C	2.309535	-1.218272	-1.261185
H	2.179363	-1.335063	-2.342607
C	3.738359	-0.782626	-0.934080
H	3.986285	0.075330	-1.563187
C	-0.411247	3.303617	-0.108001
H	-0.735103	3.524301	0.920535
C	3.838252	-0.362786	0.526376
H	3.661623	-1.239128	1.164585
C	-1.584736	2.729744	-0.885025
H	-2.429564	3.423604	-0.855758
H	-1.292470	2.625255	-1.936740
C	2.790034	0.686482	0.847380
H	2.992795	1.582547	0.248879
C	-3.291563	0.919953	-0.509937
H	-3.979301	1.517190	-1.096006
C	-1.992803	1.398166	-0.314132
C	1.405773	0.153514	0.481501
H	1.200558	-0.714842	1.116975
C	-3.717085	-0.274747	0.054567
C	-2.810479	-1.012901	0.839033
C	-1.527275	-0.535192	1.027818
H	-0.869938	-1.123757	1.658567
C	0.167994	4.552788	-0.733331
H	0.999750	4.933277	-0.137643
H	-0.594528	5.331940	-0.796586
H	0.529992	4.342518	-1.742762
C	-1.092718	0.659792	0.449146
C	0.987767	-3.182012	-1.093077
H	1.092277	-3.393463	-2.162653
H	0.056559	-2.637496	-0.920267
H	0.965732	-4.119654	-0.540338
C	-5.913402	-0.060599	-0.841942
H	-6.831793	-0.643166	-0.813301
H	-5.581839	0.041840	-1.879337
H	-6.100951	0.930560	-0.418827
C	-3.401747	-3.296225	0.648098
H	-4.190054	-3.114868	-0.084965
H	-3.697327	-4.114310	1.303614
H	-2.473793	-3.563309	0.133708
H	4.311934	-2.622963	-0.902254
H	5.776147	-0.465171	0.634252
H	3.708442	1.193631	2.469906
O	2.108440	-2.446214	-0.615017

O	0.626079	2.324778	-0.060446
O	4.676741	-1.796812	-1.248564
O	5.107694	0.203078	0.824383
O	2.793270	1.004643	2.230577
O	1.374798	-0.232962	-0.892486
O	-4.968771	-0.783805	-0.065578
O	-3.200979	-2.157323	1.488385

B3PW91 Energy = -1303.27812249 a.u.

3, Conf E

C	0.282697	1.219973	0.722181
H	0.340502	1.433447	1.800572
C	2.156617	-1.221597	-1.334619
H	2.030210	-1.288367	-2.420731
C	3.606734	-0.890726	-0.979397
H	3.915553	-0.028531	-1.574845
C	-0.304564	3.403377	-0.040966
H	-0.653318	3.601435	0.983993
C	3.718275	-0.530114	0.496208
H	3.480008	-1.415763	1.100754
C	-1.475941	2.917579	-0.878208
H	-2.287121	3.650656	-0.849278
H	-1.152371	2.839192	-1.923080
C	2.734886	0.571133	0.848221
H	2.997382	1.474019	0.284290
C	-3.271768	1.161742	-0.621002
H	-3.939126	1.773989	-1.219184
C	-1.966227	1.589384	-0.368268
C	1.324911	0.136584	0.452924
H	1.064481	-0.743309	1.051037
C	-3.764036	-0.024221	-0.112141
C	-2.939098	-0.819364	0.706084
C	-1.636534	-0.405034	0.949852
H	-1.002333	-1.005697	1.589015
C	0.359216	4.643336	-0.596736
H	0.746351	4.452455	-1.600679
H	1.187175	4.957534	0.041392
H	-0.360277	5.462715	-0.655374
C	-1.136940	0.781474	0.403975
C	0.706860	-3.099583	-1.248510
H	-0.190109	-2.509413	-1.046206
H	0.628451	-4.060452	-0.742741
H	0.798621	-3.264195	-2.327438
C	-5.261045	-1.449013	-1.252992
H	-6.336431	-1.599462	-1.338131
H	-4.794748	-2.364213	-0.882208
H	-4.851939	-1.199063	-2.236899
C	-2.712575	-2.752403	2.080830
H	-2.405803	-2.197782	2.972524
H	-1.827146	-3.141082	1.569632
H	-3.350196	-3.583042	2.376064
H	4.061029	-2.764154	-1.010716
H	5.644349	-0.760395	0.612707
H	3.666995	0.963535	2.494779

O	1.870380	-2.457525	-0.737882
O	0.681191	2.371779	0.002614
O	4.482044	-1.950998	-1.322250
O	5.018312	-0.058137	0.823855
O	2.744551	0.837888	2.242134
O	1.283867	-0.192397	-0.935444
O	-5.073820	-0.364728	-0.340650
O	-3.498899	-1.945783	1.215890

B3PW91 Energy = -1303.27793496 a.u.

3, Conf F

C	0.215257	1.153667	0.701686
H	0.197891	1.322952	1.789249
C	2.338299	-1.091151	-1.337011
H	2.285930	-1.107661	-2.431070
C	3.743882	-0.709496	-0.870698
H	4.045754	0.195271	-1.403220
C	-0.439246	3.334795	-0.013039
H	-0.860694	3.472934	0.994309
C	3.742168	-0.418046	0.624229
H	3.509852	-1.342708	1.169872
C	-1.529425	2.824788	-0.940858
H	-2.377242	3.515699	-0.938424
H	-1.137485	2.802567	-1.964801
C	2.684453	0.617330	0.959860
H	2.936964	1.557750	0.455794
C	-3.250009	0.979309	-0.849096
H	-3.912572	1.582458	-1.461743
C	-1.981418	1.455681	-0.510182
C	1.326356	0.135648	0.453415
H	1.073883	-0.783384	0.993187
C	-3.709078	-0.249411	-0.416204
C	-2.884385	-1.046571	0.399850
C	-1.624941	-0.574767	0.744899
H	-0.992431	-1.177615	1.383570
C	0.190373	4.628671	-0.478255
H	-0.566627	5.412451	-0.550154
H	0.647060	4.499710	-1.462614
H	0.960408	4.957436	0.222072
C	-1.158749	0.659119	0.280817
C	0.990347	-3.041803	-1.447131
H	0.051778	-2.504772	-1.290854
H	0.922980	-4.027242	-0.989359
H	1.171390	-3.152690	-2.521605
C	-5.956744	-0.652535	0.181928
H	-6.119836	0.374878	0.522173
H	-5.696290	-1.285032	1.033214
H	-6.868308	-1.026906	-0.282011
C	-2.610164	-3.056648	1.649978
H	-3.206451	-3.945554	1.844874
H	-2.398774	-2.548581	2.595557
H	-1.669800	-3.350524	1.174935
H	4.293032	-2.555297	-0.962170
H	5.666003	-0.562810	0.857685

H	3.488449	0.971628	2.681009	O	2.591408	0.816600	2.361960
O	2.078107	-2.368928	-0.821797	O	1.389449	-0.127770	-0.947877
O	0.595555	2.353754	0.054558	O	-4.942145	-0.693376	-0.824339
O	4.692281	-1.708130	-1.204022	O	-3.393594	-2.243013	0.788885
O	4.993099	0.097560	1.059557	B3PW91 Energy = -1303.27789441 a.u.			

Table S6. Cartesian coordinates and energies of the low-energy conformers calculated at the B3PW91/TZVP PCM/MeOH level.

36, Conf A

C	3.149813	0.414565	-0.990570
C	2.865285	-0.675141	-0.150964
C	1.771975	-0.607657	0.711854
C	0.913025	0.496873	0.708793
C	1.229703	1.583511	-0.103658
C	2.339704	1.544370	-0.941978
C	-0.319000	0.537649	1.577791
O	-0.706180	1.849188	1.993274
C	0.060277	2.969856	1.546627
C	0.409600	2.827322	0.069475
C	-1.564726	-0.096637	0.933855
C	-1.431973	-1.586056	0.604332
C	-2.746842	-2.072350	0.019305
C	-3.175019	-1.230033	-1.170231
C	-3.166573	0.241640	-0.778490
O	-1.886893	0.592573	-0.266107
O	4.235077	0.277910	-1.788121
O	3.689000	-1.768157	-0.126698
C	4.558857	1.347731	-2.666642
C	3.430308	-2.721453	-1.162161
C	-0.758064	4.205109	1.855468
O	-3.418498	1.006209	-1.900157
H	-0.125849	-0.045516	2.483878
C	-3.783496	2.352844	-1.620868
O	-4.476355	-1.585329	-1.601418
O	-1.155378	-2.388723	1.738110
O	-2.665866	-3.417416	-0.427301
H	-2.377846	0.031908	1.664141
O	1.519763	-1.668225	1.558070
C	2.451578	-1.803797	2.644995
H	2.573265	2.412948	-1.544176
H	0.999716	3.008202	2.117673
H	-0.517583	2.772921	-0.508193
H	0.969384	3.702651	-0.267588
H	-0.647328	-1.709114	-0.152329
H	-3.518368	-1.988544	0.799455
H	-2.447354	-1.362449	-1.982245
H	-3.927090	0.428149	-0.000625
H	5.436757	1.022143	-3.220440
H	4.799332	2.259059	-2.111917
H	3.742424	1.548722	-3.366004
H	4.142281	-3.533192	-1.019888
H	2.412153	-3.112378	-1.082492
H	3.579094	-2.276268	-2.148701
H	-1.687639	4.206855	1.281828
H	-1.006438	4.243768	2.917872
H	-0.191586	5.104323	1.604116
H	-4.661273	2.389678	-0.966428
H	-4.028605	2.815352	-2.575598
H	-2.962175	2.901870	-1.154467
H	-4.488726	-2.546662	-1.681628
H	-0.193940	-2.332900	1.869559
H	-2.370204	-3.945360	0.324124
H	3.455840	-1.986313	2.263770
H	2.439913	-0.905302	3.267063
H	2.117901	-2.658870	3.230437

B3PW91 Energy = -1417.78544507 a.u.

36, Conf B

C	3.145185	0.423595	-1.004571
C	2.872822	-0.661694	-0.155421
C	1.783657	-0.595768	0.713047
C	0.916078	0.502020	0.704811
C	1.221281	1.585200	-0.116690
C	2.327621	1.548197	-0.959939
C	-0.312201	0.539911	1.579107
O	-0.705107	1.851858	1.988822
C	0.049002	2.975362	1.528902
C	0.393504	2.824655	0.051440
C	-1.558669	-0.101980	0.941941
C	-1.423504	-1.604407	0.648081
C	-2.719154	-2.115958	0.039198
C	-3.141742	-1.249720	-1.136756
C	-3.162944	0.219347	-0.741988
O	-1.884197	0.590682	-0.255110
O	4.226685	0.288180	-1.807631
O	3.704632	-1.748717	-0.129385
C	4.540047	1.355066	-2.693352
C	3.435950	-2.717280	-1.147928
C	-0.779543	4.205652	1.830623
O	-3.458566	0.958449	-1.873818
H	-0.112128	-0.035343	2.488036
C	-3.857710	2.301101	-1.617630
O	-4.416468	-1.711169	-1.552920
O	-1.151925	-2.338893	1.825884
O	-2.542897	-3.461185	-0.374325
H	-2.368836	0.032192	1.674555
O	1.542684	-1.650415	1.569104
C	2.488318	-1.779530	2.644757
H	2.552143	2.414172	-1.569283
H	0.990501	3.027272	2.095536
H	-0.535435	2.760516	-0.522249
H	0.946464	3.701365	-0.293304
H	-0.622933	-1.749243	-0.087941
H	-3.503524	-2.061831	0.809184
H	-2.405806	-1.363531	-1.943324
H	-3.916224	0.396761	0.044966
H	4.777599	2.270843	-2.144694
H	3.719345	1.547513	-3.390149
H	5.417368	1.031745	-3.249417
H	3.567255	-2.284295	-2.142452
H	4.154881	-3.522933	-1.005932

H	2.421538	-3.113092	-1.047754
H	-1.710825	4.193913	1.259799
H	-1.025013	4.250563	2.893463
H	-0.222242	5.108036	1.570234
H	-4.720077	2.327252	-0.943049
H	-4.137744	2.733432	-2.576760
H	-3.040787	2.882385	-1.184169
H	-4.650792	-1.240720	-2.360716
H	-0.191246	-2.276988	1.951707
H	-3.344430	-3.709682	-0.850442
H	3.488361	-1.961296	2.252395
H	2.482094	-0.878745	3.263695
H	2.163754	-2.633192	3.237316

B3PW91 Energy = -1417.78489065 a.u.

36, Conf C

C	-3.762183	-0.116994	0.235016
C	-2.950226	-1.123365	-0.317827
C	-1.656285	-0.813975	-0.728033
C	-1.118399	0.468715	-0.533222
C	-1.949438	1.467626	-0.024861
C	-3.257569	1.169681	0.361225
C	0.315844	0.771198	-0.934509
O	0.674927	2.125940	-0.724975
C	-0.347735	3.081733	-1.001181
C	-1.460601	2.891639	0.015229
C	1.355130	-0.075127	-0.184674
C	1.516447	0.282620	1.296517
C	2.668909	-0.494113	1.925054
C	3.934086	-0.358313	1.104745
C	3.621709	-0.701315	-0.349353
O	2.599945	0.137569	-0.849411
O	-5.010947	-0.495703	0.592975
O	-3.444906	-2.384939	-0.510241
C	-5.868794	0.483941	1.163585
C	-3.387599	-3.219496	0.650843
C	0.289188	4.452223	-0.950139
O	4.779524	-0.497692	-1.081607
H	0.440063	0.524110	-1.999217
C	4.761769	-1.070819	-2.385398
O	4.918949	-1.232649	1.623886
O	0.376431	-0.058056	2.077996
O	2.913801	-0.037573	3.245546
H	1.062048	-1.126213	-0.274740
O	-0.866547	-1.786548	-1.284328
C	-1.254883	-2.214549	-2.595560
H	-3.883072	1.969081	0.737277
H	-0.746896	2.898839	-2.009696
H	-1.078924	3.156617	1.008145
H	-2.292031	3.568314	-0.198849
H	1.729682	1.353592	1.376379
H	2.388185	-1.557764	1.945310
H	4.279037	0.683772	1.136699
H	3.297927	-1.754584	-0.425216
H	-6.067112	1.297088	0.459663
H	-5.446955	0.892407	2.086273
H	-6.799836	-0.031357	1.389320
H	-2.354349	-3.344729	0.986028
H	-3.991270	-2.803451	1.460985
H	-3.793317	-4.186518	0.357059
H	0.716078	4.640087	0.037973
H	1.083988	4.534973	-1.693523
H	-0.456507	5.222822	-1.156088
H	4.555237	-2.145220	-2.333710
H	5.751967	-0.914447	-2.809897
H	4.015210	-0.587259	-3.019564
H	5.680431	-1.172161	1.034582
H	-0.373412	0.472652	1.783850
H	2.061054	-0.048140	3.697382
H	-1.249602	-1.370682	-3.291351
H	-0.510983	-2.945225	-2.909002
H	-2.242260	-2.677258	-2.579548

B3PW91 Energy = -1417.78467850 a.u.

36, Conf D

C	-3.760522	-0.111460	0.233658
C	-2.951875	-1.102820	-0.350325
C	-1.656738	-0.785147	-0.750374
C	-1.114034	0.488797	-0.514548
C	-1.942148	1.474329	0.024298
C	-3.251507	1.168867	0.400178
C	0.321457	0.799084	-0.906225
O	0.684679	2.145140	-0.653118
C	-0.334257	3.112585	-0.900152
C	-1.448954	2.894845	0.108731
C	1.357919	-0.073463	-0.183074
C	1.508506	0.224021	1.313805
C	2.660708	-0.593100	1.873357
C	3.939578	-0.412976	1.073872
C	3.647851	-0.689352	-0.393445
O	2.604571	0.166938	-0.831789
O	-5.010558	-0.496820	0.579656
O	-3.450622	-2.356141	-0.582211
C	-5.864551	0.467176	1.181929
C	-3.400033	-3.225386	0.553493
C	0.307474	4.478558	-0.805639
O	4.778143	-0.420841	-1.140558
H	0.444751	0.586047	-1.978381
C	4.717281	-0.900331	-2.479261
O	4.942562	-1.302327	1.529555
O	0.369117	-0.158323	2.075257
O	2.962302	-0.246128	3.218125
H	1.066148	-1.120834	-0.313666
O	-0.870506	-1.742359	-1.337239
C	-1.260048	-2.126274	-2.661734
H	-3.874592	1.958240	0.800587
H	-0.733083	2.962929	-1.914310
H	-1.067940	3.128047	1.109816

H	-2.278131	3.580232	-0.085632
H	1.721441	1.289955	1.444822
H	2.374821	-1.654054	1.823293
H	4.276825	0.628901	1.156818
H	3.334012	-1.739969	-0.525293
H	-6.061060	1.302666	0.504162
H	-5.440210	0.845130	2.116409
H	-6.796972	-0.051984	1.392592
H	-2.368301	-3.364125	0.888066
H	-4.005088	-2.831829	1.373756
H	-3.807918	-4.181764	0.229433
H	0.734021	4.633845	0.188263
H	1.103357	4.581566	-1.545327
H	-0.435179	5.257983	-0.988016
H	4.516084	-1.977324	-2.497402
H	5.692847	-0.710874	-2.924146
H	3.949321	-0.379160	-3.055923
H	4.956232	-1.229555	2.491820
H	-0.389891	0.358671	1.779599
H	2.173709	-0.418419	3.745042
H	-1.250895	-1.260452	-3.329974
H	-0.519088	-2.849608	-2.998279
H	-2.249365	-2.585097	-2.660834

B3PW91 Energy = -1417.78419987 a.u.

36, Conf E

C	-3.772978	-0.094351	0.250489
C	-2.968350	-1.122233	-0.273209
C	-1.671763	-0.834469	-0.690490
C	-1.123807	0.449219	-0.528832
C	-1.947598	1.468280	-0.048637
C	-3.258588	1.191461	0.342824
C	0.311255	0.728817	-0.943636
O	0.679007	2.089084	-0.791416
C	-0.342675	3.041691	-1.081344
C	-1.444553	2.887304	-0.046564
C	1.353546	-0.093748	-0.168244
C	1.509332	0.319656	1.306236
C	2.694907	-0.394049	1.934508
C	3.935766	-0.293894	1.063605
C	3.615906	-0.743261	-0.352773
O	2.583341	0.074543	-0.869967
O	-5.024538	-0.453610	0.617351
O	-3.473127	-2.384625	-0.430145
C	-5.874910	0.547066	1.162375
C	-3.417970	-3.188233	0.752792
C	0.302218	4.409435	-1.076478
O	4.755130	-0.582096	-1.121800
H	0.429772	0.437885	-1.997623
C	4.703368	-1.221076	-2.393516
O	4.930307	-1.099081	1.674522
O	0.364350	-0.038260	2.068679
O	2.939672	0.160756	3.217949
H	1.048726	-1.144912	-0.207898
O	-0.887452	-1.826201	-1.218630
C	-1.282441	-2.296960	-2.513346
H	-3.877994	2.005479	0.696888
H	-0.755620	2.833373	-2.079323
H	-1.046913	3.169675	0.935112
H	-2.271495	3.567718	-0.265708
H	1.683534	1.399848	1.353608
H	2.434199	-1.458244	2.033135
H	4.260015	0.753459	1.013236
H	3.287752	-1.797668	-0.361086
H	-6.809731	0.044765	1.401346
H	-6.067152	1.343055	0.437524
H	-5.449710	0.975951	2.074143
H	-3.832713	-4.159371	0.486520
H	-2.384517	-3.312898	1.087456
H	-4.015025	-2.745602	1.553681
H	0.741191	4.622875	-0.098921
H	1.089052	4.466357	-1.830703
H	-0.441572	5.178076	-1.296162
H	4.498322	-2.291264	-2.281825
H	5.682371	-1.088433	-2.850901
H	3.940955	-0.769918	-3.032798
H	5.750111	-0.978107	1.182610
H	-0.406687	0.386942	1.672639
H	3.745526	-0.254719	3.547878
H	-2.270228	-2.757500	-2.477718
H	-1.279092	-1.476428	-3.236470
H	-0.540580	-3.038394	-2.805537

B3PW91 Energy = -1417.78418928 a.u.

36, Conf F

C	-3.804699	-0.160929	0.244752
C	-2.950545	-1.195022	-0.171323
C	-1.652435	-0.891823	-0.577818
C	-1.154187	0.416433	-0.507300
C	-2.024402	1.436318	-0.130356
C	-3.336049	1.146262	0.244580
C	0.274399	0.716064	-0.922788
O	0.601491	2.094411	-0.875612
C	-0.447645	2.991287	-1.234009
C	-1.560300	2.864718	-0.206681
C	1.344771	-0.022490	-0.104392
C	1.653156	0.578584	1.267153
C	2.816441	-0.166706	1.907605
C	4.015990	-0.229724	0.982480
C	3.571578	-0.794898	-0.360147
O	2.536211	0.009185	-0.894429
O	-5.056735	-0.534190	0.607243
O	-3.406134	-2.485855	-0.240616
C	-5.953687	0.477803	1.042782
C	-3.338293	-3.193892	0.999881
C	0.152157	4.377951	-1.305657
O	4.658395	-0.768377	-1.216785
H	0.408415	0.353411	-1.953240

C	4.490005	-1.532987	-2.406563
O	4.994117	-1.043581	1.606256
O	0.506148	0.485743	2.095046
O	3.111051	0.502374	3.126819
H	1.017895	-1.059832	0.018710
O	-0.824657	-1.899597	-1.008799
C	-1.161177	-2.454982	-2.284850
H	-3.991805	1.962986	0.518402
H	-0.839503	2.710035	-2.223009
H	-1.182010	3.208796	0.762904
H	-2.399298	3.512438	-0.474805
H	1.941406	1.626478	1.128269
H	2.491251	-1.195199	2.120606
H	4.400581	0.783678	0.810883
H	3.202365	-1.828114	-0.234288
H	-6.149251	1.205416	0.249741
H	-5.571351	0.995525	1.927301
H	-6.879704	-0.033779	1.296787
H	-2.305875	-3.254591	1.355457
H	-3.961697	-2.713867	1.758420
H	-3.714002	-4.197746	0.805802
H	0.566550	4.666937	-0.336748
H	0.950195	4.414322	-2.049497
H	-0.612156	5.106070	-1.585067
H	4.249268	-2.574509	-2.167468
H	5.438616	-1.496330	-2.939517
H	3.703225	-1.114229	-3.038256
H	5.775017	-1.047915	1.041226
H	0.791174	0.736396	2.982404
H	3.785785	-0.007853	3.588229
H	-2.142400	-2.930815	-2.257993
H	-1.144448	-1.680575	-3.057443
H	-0.397799	-3.199818	-2.504894

B3PW91 Energy = -1417.78416165 a.u.

36, Conf G

C	3.721611	-0.041247	-0.450373
C	2.978994	-1.112990	0.079637
C	1.680336	-0.891791	0.533524
C	1.112736	0.397749	0.493546
C	1.899894	1.463430	0.059000
C	3.187778	1.238352	-0.430246
C	-0.302428	0.626730	1.000032
O	-0.685092	1.992253	0.990288
C	0.344775	2.928324	1.301276
C	1.385657	2.871923	0.196862
C	-1.374372	-0.122648	0.193624
C	-1.577991	0.402544	-1.233028
C	-2.769840	-0.296544	-1.864893
C	-4.010855	-0.231507	-0.991624
C	-3.664314	-0.747725	0.396766
O	-2.589858	0.015420	0.923033
O	4.956376	-0.353994	-0.907382
O	3.529529	-2.366099	0.129990
C	5.766294	0.697179	-1.417369
C	4.422829	-2.567827	1.230031
C	-0.308659	4.284336	1.446983
O	-4.757760	-0.592892	1.226107
H	-0.371566	0.239622	2.026899
C	-4.647221	-1.279351	2.468036
O	-5.049816	-1.020127	-1.541965
O	-0.477294	0.121402	-2.089497
O	-3.119783	0.268777	-3.121149
H	-1.090306	-1.178660	0.145706
O	0.936629	-1.874798	1.116104
C	0.820018	-3.148918	0.470025
H	3.771712	2.083300	-0.771432
H	0.815810	2.642944	2.253477
H	0.929555	3.226770	-0.734543
H	2.218603	3.543022	0.421922
H	-1.769641	1.479635	-1.189932
H	-2.504479	-1.355209	-2.000786
H	-4.329596	0.814352	-0.888271
H	-3.363293	-1.809027	0.342854
H	5.983252	1.442181	-0.646649
H	5.291526	1.183587	-2.274126
H	6.694544	0.228814	-1.737263
H	3.902242	-2.430150	2.182082
H	4.777194	-3.594985	1.155790
H	5.273668	-1.885290	1.172525
H	-0.804179	4.573171	0.516948
H	-1.051662	4.269342	2.246321
H	0.441234	5.040622	1.687906
H	-5.598321	-1.150701	2.982189
H	-3.844459	-0.865662	3.083003
H	-4.466698	-2.348018	2.306701
H	-5.099070	-0.792274	-2.478544
H	0.310171	0.550355	-1.731998
H	-2.361468	0.165546	-3.707161
H	1.664252	-3.794162	0.705335
H	-0.101462	-3.589940	0.849149
H	0.747530	-3.028834	-0.613185

B3PW91 Energy = -1417.78278772 a.u.

36, Conf H

C	-3.680021	0.048269	0.484152
C	-2.944040	-1.090148	0.099700
C	-1.658667	-0.924484	-0.417995
C	-1.077283	0.352682	-0.521873
C	-1.852072	1.469832	-0.215879
C	-3.143775	1.312183	0.288503
C	0.343180	0.501326	-1.039422
O	0.741967	1.853176	-1.193958
C	-0.276653	2.755544	-1.621321
C	-1.323339	2.845634	-0.523801
C	1.394676	-0.159399	-0.134949
C	1.602457	0.541000	1.213460
C	2.769399	-0.104679	1.941267

C	4.018688	-0.178578	1.080610
C	3.669344	-0.856640	-0.235743
O	2.619040	-0.139324	-0.864826
O	-4.891098	-0.190448	1.041707
O	-3.415685	-2.353998	0.321552
C	-5.665501	0.922315	1.469600
C	-4.588249	-2.738707	-0.407517
C	0.391653	4.076931	-1.928368
O	4.772895	-0.836030	-1.066420
H	0.414112	-0.012532	-2.009154
C	4.653248	-1.671603	-2.212315
O	5.033748	-0.915626	1.736979
O	0.488723	0.404661	2.088314
O	3.123849	0.602058	3.122450
H	1.084542	-1.194501	0.042826
O	-0.882303	-1.988167	-0.778808
C	-1.409185	-2.927342	-1.720245
H	-3.723648	2.195389	0.523286
H	-0.747535	2.361183	-2.533862
H	-0.868377	3.309085	0.359422
H	-2.148132	3.491617	-0.835639
H	1.826514	1.597713	1.033985
H	2.475064	-1.131715	2.202621
H	4.363555	0.838529	0.850952
H	3.340413	-1.894675	-0.051015
H	-5.932780	1.568526	0.628889
H	-5.134897	1.505897	2.227085
H	-6.570819	0.504260	1.904260
H	-5.450079	-2.140767	-0.112589
H	-4.422360	-2.641490	-1.484189
H	-4.765135	-3.784917	-0.162962
H	0.888664	4.471396	-1.038836
H	1.135664	3.957617	-2.717949
H	-0.349795	4.806127	-2.261389
H	4.434397	-2.704494	-1.919053
H	5.613955	-1.640653	-2.723826
H	3.872139	-1.314195	-2.887628
H	5.077965	-0.575775	2.639273
H	-0.283304	0.808101	1.672489
H	2.359060	0.588724	3.708993
H	-1.953306	-2.412602	-2.516344
H	-0.547523	-3.438241	-2.147981
H	-2.060615	-3.652851	-1.234757

B3PW91 Energy = -1417.78273111 a.u.

36, Conf I

C	-3.731222	0.003991	0.493811
C	-2.942589	-1.130140	0.227500
C	-1.651164	-0.954662	-0.274620
C	-1.117778	0.326314	-0.482736
C	-1.942170	1.430820	-0.293651
C	-3.237963	1.265951	0.192954
C	0.300989	0.485147	-0.996499
O	0.660934	1.829845	-1.267012
C	-0.382854	2.664663	-1.763522
C	-1.448102	2.795272	-0.687699
C	1.383868	-0.083157	-0.066050
C	1.738065	0.786746	1.140522
C	2.912989	0.174465	1.891432
C	4.084525	-0.098420	0.968969
C	3.595848	-0.927734	-0.211394
O	2.552414	-0.239904	-0.875338
O	-4.948519	-0.230731	1.046487
O	-3.367424	-2.393927	0.536331
C	-5.772645	0.885550	1.351201
C	-4.518322	-2.872475	-0.169533
C	0.245621	3.984149	-2.153371
O	4.657477	-1.097210	-1.083543
H	0.390204	-0.101528	-1.923167
C	4.449910	-2.095018	-2.078277
O	5.074303	-0.776181	1.723293
O	0.616069	0.884724	2.001567
O	3.248027	1.079105	2.935814
H	1.047319	-1.064669	0.282009
O	-0.836258	-2.031314	-0.513782
C	-1.255460	-2.950169	-1.524991
H	-3.856111	2.142623	0.338851
H	-0.828471	2.194177	-2.652855
H	-1.015872	3.322693	0.170636
H	-2.282682	3.400158	-1.052327
H	2.030828	1.778616	0.778477
H	2.584636	-0.781871	2.323264
H	4.471833	0.850547	0.576622
H	3.222775	-1.906200	0.139622
H	-6.674212	0.475707	1.801638
H	-6.040824	1.442428	0.448777
H	-5.284379	1.556759	2.063504
H	-4.632687	-3.917975	0.113296
H	-5.412849	-2.315511	0.108224
H	-4.365088	-2.804796	-1.250647
H	0.714529	4.456240	-1.286580
H	1.006265	3.836607	-2.922246
H	-0.514163	4.662625	-2.546857
H	4.212118	-3.059390	-1.616483
H	5.382104	-2.182452	-2.633844
H	3.646206	-1.811639	-2.761819
H	5.836296	-0.914102	1.149293
H	0.932236	1.303802	2.811485
H	3.925869	0.664044	3.480517
H	-1.574699	-2.416899	-2.424709
H	-0.384794	-3.561202	-1.759639
H	-2.061853	-3.591811	-1.169841

B3PW91 Energy = -1417.78253955 a.u.

36, Conf J

C	-3.794298	-0.086681	0.440261
C	-2.988430	-1.172339	0.058556
C	-1.678010	-0.944057	-0.361227

C	-1.158641	0.360272	-0.434358
C	-2.000477	1.428278	-0.135511
C	-3.300762	1.203791	0.315636
C	0.251266	0.598568	-0.947044
O	0.589867	1.970501	-1.066143
C	-0.467159	2.840695	-1.462082
C	-1.523315	2.835049	-0.370748
C	1.367469	-0.051138	-0.114885
C	1.742498	0.684811	1.172106
C	2.949238	0.017383	1.818558
C	4.097049	-0.130324	0.839179
C	3.586896	-0.834978	-0.410955
O	2.513736	-0.097588	-0.966830
O	-5.043332	-0.398435	0.866062
O	-3.489411	-2.448138	0.109806
C	-5.909944	0.668551	1.224759
C	-4.291554	-2.802500	-1.020218
C	0.141063	4.203551	-1.708208
O	4.624030	-0.889802	-1.325503
H	0.329019	0.126311	-1.937884
C	4.399517	-1.775525	-2.418166
O	5.119533	-0.867498	1.486445
O	0.643745	0.665086	2.067607
O	3.300043	0.809795	2.945539
H	1.060520	-1.070922	0.133126
O	-0.889974	-1.968510	-0.813561
C	-0.667995	-3.087200	0.053616
H	-3.926640	2.055727	0.548592
H	-0.913689	2.464951	-2.394968
H	-1.088095	3.263356	0.539585
H	-2.367687	3.468577	-0.654915
H	2.008390	1.715806	0.914355
H	2.650549	-0.986617	2.152647
H	4.457260	0.863042	0.542754
H	3.238501	-1.852329	-0.159082
H	-6.108298	1.325444	0.372783
H	-5.497459	1.255787	2.050237
H	-6.839979	0.202794	1.543898
H	-5.169389	-2.156151	-1.094196
H	-3.709112	-2.741007	-1.943996
H	-4.612864	-3.831305	-0.862491
H	0.607961	4.585595	-0.797110
H	0.899550	4.151100	-2.491442
H	-0.630113	4.909763	-2.022858
H	4.188022	-2.788384	-2.058654
H	5.315731	-1.786399	-3.006083
H	3.572065	-1.431489	-3.042946
H	5.866396	-0.926276	0.879913
H	0.974113	1.002851	2.909226
H	4.001419	0.352247	3.422181
H	-1.541291	-3.735293	0.100456
H	0.177722	-3.628690	-0.368522
H	-0.414974	-2.749018	1.062418

B3PW91 Energy = -1417.78253087 a.u.

Table S7. Cartesian coordinates and energies of the lowest-energy conformers calculated at the ωB97X/TZVP PCM/CH_2Cl_2 level.

3, Conf A

C	0.246793	1.166440	0.779846
H	0.305071	1.355767	1.861985
C	2.187525	-1.131147	-1.356615
H	2.021752	-1.222174	-2.435463
C	3.628903	-0.716567	-1.062280
H	3.868716	0.152051	-1.679322
C	-0.513627	3.315762	0.102065
H	-0.848137	3.453335	1.141278
C	3.780509	-0.331329	0.402569
H	3.628640	-1.223271	1.025430
C	-1.667428	2.774643	-0.725570
H	-2.531963	3.438293	-0.636921
H	-1.369707	2.766925	-1.779927
C	2.743450	0.706343	0.788868
H	2.918736	1.620863	0.210617
C	-3.305442	0.864122	-0.552380
H	-3.999107	1.472419	-1.120207
C	-2.035257	1.385259	-0.270520
C	1.354218	0.171360	0.457807
H	1.191166	-0.730440	1.058711
C	-3.681404	-0.388966	-0.116348
C	-2.771304	-1.152014	0.647743
C	-1.523441	-0.635059	0.925301
H	-0.831788	-1.215684	1.522492
C	0.040266	4.617249	-0.431548
H	-0.740904	5.379532	-0.453987
H	0.417395	4.476958	-1.447084
H	0.856846	4.974918	0.197014
C	-1.136577	0.626953	0.452897
C	0.790954	-3.025005	-1.128854
H	0.789481	-3.201977	-2.209244
H	-0.086463	-2.430175	-0.859452
H	0.761501	-3.978666	-0.605309
C	-5.827827	-0.228592	-1.113845
H	-6.710470	-0.858605	-1.194013
H	-5.443701	-0.012201	-2.115022
H	-6.095203	0.708597	-0.616894
C	-2.336504	-3.157037	1.841154
H	-2.076473	-2.653340	2.776859
H	-1.420607	-3.396482	1.291503
H	-2.872942	-4.076391	2.063331
H	4.180068	-2.564262	-1.087233
H	5.720631	-0.423020	0.408269
H	3.706488	1.190750	2.392655
O	1.989130	-2.370017	-0.732595
O	0.544797	2.363151	0.088296
O	4.543746	-1.734881	-1.420630
O	5.058237	0.226689	0.663042
O	2.792330	0.979378	2.177998
O	1.277254	-0.152849	-0.927722
O	-4.887766	-0.965283	-0.351056
O	-3.215462	-2.368464	1.058689

ωB97X Energy = -1303.46938424 a.u.

4, Conf A

C	-3.725863	-0.501025	0.192486
C	-2.730045	-1.330125	-0.367165
C	-1.486378	-0.804237	-0.644817
C	-1.186283	0.540534	-0.375710
C	-2.176316	1.364048	0.130581
C	-3.438392	0.828522	0.419081
C	-1.930602	2.838997	0.322190
C	-0.774298	3.289881	-0.548456
O	0.341088	2.440880	-0.273067
C	0.196906	1.091810	-0.693637
C	1.348837	0.316583	-0.027955
C	2.695090	1.049656	-0.105524
C	3.841296	0.151005	0.343867
C	3.831543	-1.176529	-0.393464
C	2.439494	-1.799706	-0.297211
O	1.446612	-0.911323	-0.748968
O	5.081449	0.794229	0.113237
O	2.762764	2.182930	0.740841
O	4.809360	-2.076313	0.082135
C	-0.333430	4.710509	-0.281810
H	0.364047	1.041565	-1.780097
H	1.103764	0.124564	1.023798
O	2.260946	-2.182117	1.035293
C	1.114641	-2.987965	1.264210
O	-4.919482	-1.092477	0.446310
C	-5.945837	-0.286986	1.000588
O	-3.093251	-2.619115	-0.597992
C	-2.143888	-3.465990	-1.221325
H	-0.718704	-1.429299	-1.076722
H	-4.200054	1.489077	0.815180
H	-2.831380	3.402903	0.066366
H	-1.691868	3.062972	1.367591
H	-1.043523	3.172976	-1.607239
H	2.871293	1.338984	-1.152017
H	3.711576	-0.041111	1.417587
H	4.056702	-1.000129	-1.447476
H	2.359060	-2.670774	-0.956615
H	5.018859	1.664269	0.521456
H	1.993344	2.727760	0.524035
H	4.519085	-2.372555	0.952353
H	-0.020765	4.820598	0.759298
H	0.500425	4.984481	-0.929601
H	-1.158110	5.400368	-0.468476
H	0.194882	-2.415720	1.110494
H	1.116493	-3.858331	0.598556

H	1.161898	-3.323431	2.297931
H	-6.802966	-0.942291	1.135351
H	-5.647208	0.124628	1.969104
H	-6.216689	0.530400	0.325986
H	-1.859183	-3.085721	-2.206780
H	-1.246547	-3.580900	-0.605504
H	-2.629163	-4.432710	-1.332461

ωB97X Energy = -1303.46926252 a.u.

5, Conf A

C	-3.708858	-0.184558	-0.125955
C	-2.909267	-1.047148	0.656660
C	-1.648584	-0.637160	1.035882
C	-1.146305	0.615043	0.654761
C	-1.927046	1.460755	-0.105069
C	-3.210916	1.049105	-0.489108
C	-1.412902	2.820041	-0.504306
C	0.103940	2.867234	-0.451490
O	0.549067	2.374343	0.818425
C	0.246969	1.019348	1.096825
C	1.316298	0.059078	0.568165
C	2.724843	0.445441	1.001770
C	3.718060	-0.544732	0.421357
C	3.565152	-0.639393	-1.090806
C	2.108107	-0.912300	-1.461281
O	1.249503	0.021388	-0.856764
O	5.016928	-0.105418	0.784969
O	2.771197	0.437314	2.416955
O	4.430130	-1.619644	-1.632076
C	0.653078	4.269026	-0.591046
H	0.305488	0.941685	2.185887
H	1.096514	-0.937378	0.970832
O	1.833429	-2.229900	-1.077172
C	0.578379	-2.713745	-1.539320
O	-4.935651	-0.657576	-0.463996
C	-5.767643	0.181637	-1.246052
O	-3.466269	-2.243352	0.979472
C	-2.683376	-3.144813	1.741933
H	-1.036945	-1.288074	1.648840
H	-3.811731	1.721584	-1.089380
H	-1.751644	3.069284	-1.513667
H	-1.816748	3.584378	0.169747
H	0.511037	2.227529	-1.240741
H	2.949630	1.447815	0.616203
H	3.520447	-1.530969	0.862453
H	3.858169	0.315313	-1.533028
H	1.948637	-0.791550	-2.538314
H	5.652311	-0.730557	0.422804
H	3.691632	0.558315	2.671176
H	4.023999	-2.477123	-1.456285
H	0.290468	4.900949	0.223032
H	1.743867	4.259649	-0.563990
H	0.333900	4.706734	-1.539037
H	0.514292	-2.634721	-2.629430
H	0.514554	-3.759792	-1.246788
H	-0.246688	-2.155391	-1.087509
H	-6.690347	-0.371589	-1.404036
H	-5.990044	1.117198	-0.724540
H	-5.308499	0.405507	-2.213483
H	-1.762412	-3.413966	1.215038
H	-2.432172	-2.724971	2.720434
H	-3.293952	-4.034271	1.877763

ωB97X Energy = -1303.46913821 a.u.

21, Conf A

C	0.240293	1.020209	-0.970241
C	2.161836	-1.937354	0.086469
C	3.618606	-1.539218	-0.161447
C	-0.099797	3.011116	0.347428
C	3.817643	-0.061419	0.137760
C	-1.606669	2.858781	0.381701
C	2.765148	0.784385	-0.566627
C	-3.308688	1.009224	0.408849
C	-2.019925	1.443984	0.070957
C	1.362331	0.292769	-0.200884
C	-3.737858	-0.266705	0.109928
C	-2.858100	-1.148367	-0.555849
C	-1.594343	-0.716222	-0.894281
C	0.347480	4.455216	0.370636
C	-1.157086	0.578023	-0.573134
C	0.727183	-2.513037	1.870582
C	-5.877804	0.096819	1.066257
C	-2.475399	-3.312701	-1.446736
O	1.966304	-1.942165	1.470423
O	0.404975	2.431626	-0.867968
O	4.489660	-2.369329	0.575510
O	5.115358	0.313766	-0.283488
O	3.020229	2.122357	-0.176549
O	1.268284	-1.078619	-0.578663
O	-4.964381	-0.763306	0.406491
O	-3.346283	-2.389226	-0.817279
H	0.394329	0.795593	-2.030049
H	1.960258	-2.931177	-0.328206
H	3.843042	-1.711296	-1.216571
H	0.338095	2.477805	1.200762
H	3.710653	0.101194	1.219502
H	-1.978214	3.154061	1.366484
H	-2.050196	3.544318	-0.349070
H	2.895292	0.673256	-1.653012
H	-3.970979	1.701289	0.914617
H	1.208772	0.394760	0.880198
H	-0.914069	-1.380631	-1.406641
H	1.435641	4.533529	0.406708
H	-0.056387	4.955709	1.252337
H	-0.013037	4.974686	-0.519697
H	0.714506	-2.515578	2.958261
H	0.641975	-3.540872	1.501848
H	-0.115406	-1.924920	1.494771

H	-5.492371	0.412553	2.040108
H	-6.787269	-0.481878	1.208402
H	-6.100078	0.979526	0.459628
H	-2.181516	-2.968173	-2.442560
H	-3.034167	-4.241211	-1.536713
H	-1.577801	-3.484567	-0.843908
H	4.162903	-2.383357	1.482490
H	5.171078	1.270225	-0.185382
H	2.332670	2.661313	-0.590340

ωB97X Energy = -1303.46862139 a.u.

31mod, Conf A

C	2.501330	0.322817	-1.437872
C	1.267568	0.978721	-1.575510
C	0.095307	0.271207	-1.429767
C	0.099329	-1.089676	-1.122427
C	1.316894	-1.743881	-0.990319
C	2.507675	-1.032316	-1.151283
C	1.370320	-3.212288	-0.646124
C	0.016963	-3.875542	-0.832863
O	-0.981972	-3.061018	-0.214506
C	-1.204026	-1.839086	-0.901681
C	-2.236883	-1.020091	-0.111168
C	-3.265964	-1.860523	0.653689
C	-4.309297	-0.929211	1.246427
C	-4.925331	-0.010083	0.207755
C	-3.818156	0.717240	-0.537381
O	-2.916651	-0.235945	-1.083368
O	-5.373133	-1.643839	1.851959
O	-2.703938	-2.556965	1.749515
O	-5.767103	0.942981	0.823873
C	-0.063990	-5.249501	-0.209551
H	-1.668695	-2.069087	-1.871386
H	-1.720526	-0.367295	0.608964
O	-4.370287	1.433709	-1.576891
C	-3.454545	2.303170	-2.229556
O	3.610689	1.091619	-1.612189
C	5.266188	0.169265	-0.048196
C	6.013199	-0.966842	0.241453
C	6.400683	-1.243037	1.546203
C	6.035718	-0.386757	2.575373
C	5.286103	0.748316	2.292795
C	4.908387	1.026971	0.987353
C	4.890861	0.486189	-1.477664
O	1.217790	2.321015	-1.869751
C	0.437394	3.107276	0.312217
C	-0.727766	3.860731	0.194662
C	-1.739510	3.751059	1.138224
C	-1.596698	2.880364	2.211800
C	-0.436770	2.128140	2.340845
C	0.574671	2.244629	1.396130
C	1.501576	3.182117	-0.751238
H	-0.838239	0.810095	-1.540604
H	3.444200	-1.562864	-1.031323
H	2.115491	-3.716831	-1.266701
H	1.684110	-3.334235	0.396350
H	-0.216899	-3.937104	-1.904869
H	-3.751612	-2.555507	-0.044071
H	-3.807097	-0.305762	2.000843
H	-5.483181	-0.608499	-0.524053
H	-3.264539	1.382839	0.148517
H	-4.992798	-2.211850	2.529282
H	-1.999190	-3.108901	1.383325
H	-6.352618	0.457400	1.414393
H	0.696851	-5.902446	-0.640725
H	0.107488	-5.185509	0.867562
H	-1.043754	-5.695963	-0.383502
H	-4.037248	2.924283	-2.906623
H	-2.940229	2.940076	-1.501674
H	-2.713820	1.739465	-2.802306
H	6.291146	-1.646568	-0.558651
H	6.982218	-2.132841	1.759018
H	6.331512	-0.603758	3.595347
H	4.998419	1.421779	3.092233
H	4.327851	1.917138	0.768937
H	4.955482	-0.411096	-2.100833
H	5.580040	1.224345	-1.889695
H	-0.841925	4.539282	-0.645436
H	-2.641218	4.345076	1.037754
H	-2.387552	2.792326	2.948305
H	-0.318463	1.450373	3.178693
H	1.479848	1.652825	1.498780
H	1.552516	4.182914	-1.178804
H	2.481879	2.927180	-0.340149

ωB97X Energy = -1765.59057892 a.u.

32mod, Conf A

C	-2.599668	-0.880993	-1.557441
C	-1.696404	0.052229	-2.097772
C	-0.347647	-0.243774	-2.095330
C	0.131783	-1.433584	-1.545417
C	-0.753635	-2.360754	-1.029551
C	-2.120522	-2.071104	-1.046622
C	-0.241648	-3.659710	-0.461647
C	1.201770	-3.530526	-0.002352
O	1.991198	-2.949110	-1.049686
C	1.629815	-1.639912	-1.445007
C	2.244758	-0.553884	-0.554074
C	3.750286	-0.689910	-0.361127
C	4.240442	0.429372	0.545547
C	3.442176	0.489386	1.836070
C	1.955240	0.539267	1.511869
O	1.614295	-0.577257	0.716561
O	5.619374	0.206312	0.785893
O	4.371860	-0.614367	-1.630261
O	3.870027	1.639852	2.540500
C	1.832897	-4.865375	0.323069
H	2.084234	-1.510117	-2.430528
H	2.050879	0.414231	-1.040572

O	1.249776	0.479035	2.698193
C	-0.152720	0.674444	2.541109
O	-3.922683	-0.537999	-1.596724
C	-4.369455	-0.551076	0.805064
C	-3.921812	0.756156	0.975401
C	-3.578101	1.223234	2.234511
C	-3.685721	0.388780	3.341177
C	-4.130483	-0.915219	3.177941
C	-4.465678	-1.383536	1.913009
C	-4.765987	-1.042978	-0.567401
O	-2.237803	1.205256	-2.583495
C	-0.604086	2.900085	-1.874877
C	0.751927	3.183696	-1.993732
C	1.434405	3.822831	-0.965791
C	0.763955	4.180074	0.195487
C	-0.591374	3.898657	0.321356
C	-1.270542	3.264556	-0.708559
C	-1.355400	2.237518	-3.008496
H	0.359909	0.467961	-2.499580
H	-2.810814	-2.793591	-0.626233
H	-0.864108	-3.975604	0.379785
H	-0.302593	-4.446666	-1.222354
H	1.242030	-2.876242	0.873761
H	3.958432	-1.656587	0.113636
H	4.105474	1.382807	0.014026
H	3.626780	-0.419599	2.421971
H	1.708116	1.464311	0.959630
H	5.948581	0.933709	1.322416
H	5.322611	-0.593377	-1.481888
H	3.423272	1.650519	3.392099
H	1.269709	-5.366329	1.113009
H	1.838812	-5.507533	-0.560799
H	2.861212	-4.731935	0.662677
H	-0.582381	0.697050	3.540644
H	-0.355408	1.623264	2.031697
H	-0.602282	-0.140916	1.968337
H	-3.834316	1.401552	0.107606
H	-3.226459	2.242659	2.354109
H	-3.418951	0.753570	4.326768
H	-4.209577	-1.574078	4.035228
H	-4.800239	-2.408946	1.788261
H	-5.758866	-0.675620	-0.830359
H	-4.807238	-2.135971	-0.587089
H	1.284124	2.900088	-2.897214
H	2.492196	4.036244	-1.071512
H	1.295071	4.674524	1.000873
H	-1.122086	4.178954	1.224808
H	-2.328912	3.047575	-0.609578
H	-2.008986	2.961328	-3.496479
H	-0.657985	1.861282	-3.763490

ωB97X Energy = -1765.59145389 a.u.

34, Conf A

C	3.754692	-0.642390	-0.307197
C	2.644279	-1.484698	-0.079487
C	1.421301	-0.925817	0.226786
C	1.259371	0.465560	0.308305
C	2.349694	1.290618	0.103715
C	3.592154	0.723123	-0.208011
C	-0.099962	1.060047	0.642621
O	-0.139238	2.449962	0.358436
C	0.947737	3.178566	0.933298
C	2.223184	2.790237	0.208952
C	-1.267178	0.390278	-0.103815
C	-2.496494	1.292435	-0.269428
C	-3.676770	0.506288	-0.828732
C	-3.934211	-0.747644	-0.023925
C	-2.634190	-1.534413	0.096785
O	-1.624267	-0.739026	0.687858
O	4.921554	-1.265912	-0.605135
O	2.884461	-2.817221	-0.185272
C	6.057207	-0.451885	-0.844170
C	1.807306	-3.700216	0.066783
C	0.618799	4.649184	0.823619
O	-2.884993	-2.623654	0.909144
H	-0.304380	0.922915	1.714702
C	-1.842726	-3.588085	0.937483
O	-4.918936	-1.520445	-0.677108
O	-2.281101	2.345663	-1.189777
O	-4.841414	1.308426	-0.830512
H	-0.936395	0.063553	-1.100176
H	0.564743	-1.561013	0.409474
H	4.436029	1.383315	-0.367632
H	1.024042	2.890188	1.991035
H	2.212983	3.238194	-0.790888
H	3.086458	3.203179	0.737579
H	-2.775741	1.689454	0.715822
H	-3.426108	0.208568	-1.857283
H	-4.256314	-0.473417	0.988773
H	-2.295475	-1.866175	-0.900908
H	5.895040	0.214112	-1.696665
H	6.874065	-1.133481	-1.068684
H	6.312438	0.142300	0.038120
H	1.441084	-3.594234	1.092743
H	0.982488	-3.532910	-0.633285
H	2.200367	-4.704313	-0.073466
H	0.484938	4.929417	-0.223914
H	-0.296198	4.882794	1.369386
H	1.432369	5.246929	1.238186
H	-1.563831	-3.885146	-0.079625
H	-2.225187	-4.453427	1.474270
H	-0.965230	-3.198425	1.459592
H	-5.040183	-2.322377	-0.158951
H	-1.529360	2.849249	-0.848172
H	-4.606334	2.131266	-1.272606

ωB97X Energy = -1303.46737260 a.u.

35, Conf A

C	3.725779	-0.203119	0.094870
C	2.986063	-1.026678	-0.783029
C	1.727271	-0.627992	-1.180134
C	1.171212	0.576704	-0.728010
C	1.894746	1.387093	0.121781
C	3.174955	0.984832	0.527595
C	-0.216740	0.971325	-1.193040
O	-0.558731	2.301788	-0.852463
C	-0.186846	2.722071	0.466721
C	1.326444	2.701567	0.591995
C	-1.280048	-0.039488	-0.747456
C	-2.684416	0.357306	-1.195495
C	-3.698205	-0.651026	-0.681094
C	-3.553121	-0.854961	0.814969
C	-2.105263	-1.201548	1.133229
O	-1.256417	-0.171831	0.666097
O	4.953452	-0.664382	0.446056
O	3.597644	-2.173895	-1.175847
C	5.727426	0.137112	1.321630
C	2.884252	-3.029959	-2.050548
C	-0.773719	4.101204	0.665161
O	-1.977569	-1.306270	2.504043
C	-0.735321	-1.858682	2.925075
O	-4.437771	-1.893918	1.186526
O	-2.699183	0.405537	-2.609871
O	-4.984763	-0.169688	-1.030220
H	1.160217	-1.248169	-1.863896
H	3.730781	1.628169	1.198954
H	-0.241184	0.950860	-2.285858
H	-0.618009	2.025942	1.192953
H	1.744446	3.521104	-0.004159
H	1.609293	2.883891	1.632397
H	-1.032246	-1.006360	-1.211012
H	-2.917281	1.342639	-0.774216
H	-3.513608	-1.613165	-1.179924
H	-3.799578	0.076998	1.338639
H	-1.823425	-2.151514	0.644089
H	5.939633	1.115615	0.880900
H	5.224539	0.273746	2.283585
H	6.660638	-0.399400	1.475465
H	3.534972	-3.881021	-2.236669
H	1.953282	-3.378492	-1.593074
H	2.658982	-2.532017	-2.998285
H	-1.861686	4.071456	0.587022
H	-0.389109	4.788900	-0.091731
H	-0.507858	4.486143	1.651711
H	-0.590090	-2.852026	2.487763
H	-0.778504	-1.942803	4.008723
H	0.096370	-1.210826	2.639718
H	-4.399419	-1.990318	2.142704
H	-3.608384	0.568168	-2.880476
H	-5.632886	-0.820816	-0.745203

ωB97X Energy = -1303.46628246 a.u.

References

[1] E. B. Lőrincz, M. Herczeg, J. Houser, M. Rievajová, Á. Kuki, L. Malinovská, L. Naesens, M. Wimmerová, A. Borbás, P. Herczegh, I. Bereczki, “Amphiphilic Sialic Acid Derivatives as Potential Dual-Specific Inhibitors of Influenza Hemagglutinin and Neuraminidase,” Int J Mol Sci, 2023 Dec 8;24(24):17268. doi: 10.3390/ijms242417268.

[2] R. Mahrwald, F. Theil, H. Schick, S. Schwarz, H. Palme, and G. Weber, “The Oxidation of Primary Trimethylsilyl Ethers to Aldehydes – a selective conversion of a primary hydroxy group into an aldehyde group in the presence of a secondary hydroxy group,” *J. Prakt. Chem.*, vol. 328, no. 5–6, pp. 777–783, Jan. 1986, doi: 10.1002/prac.19863280517.

[3] L. F. García-Alles, A. Zahn, and B. Erni, “Sugar Recognition by the Glucose and Mannose Permeases of Escherichia coli. Steady-State Kinetics and Inhibition Studies,” *Biochemistry*, vol. 41, no. 31, pp. 10077–10086, Aug. 2002, doi: 10.1021/bi025928d.

[4] K. Daragics and P. Fügedi, “Regio- and chemoselective reductive cleavage of 4,6-*O*-benzylidene-type acetals of hexopyranosides using BH3·THF–TMSOTf,” *Tetrahedron Letters*, vol. 50, no. 24, pp. 2914–2916, Jun. 2009, doi: 10.1016/j.tetlet.2009.03.194.

[5] K. Daragics, P. Szabó, and P. Fügedi, “Some observations on the reductive ring opening of 4,6-O-benzylidene acetals of hexopyranosides with the borane trimethylamine–aluminium chloride reagent,” *Carbohydrate Research*, vol. 346, no. 12, pp. 1633–1637, Sep. 2011, doi: 10.1016/j.carres.2011.04.046.

[6] H. Hashimoto, K. Asano, F. Fujii, and J. Yoshimura, “Synthesis of destomic and epi-destomic acid, and their C-6 epimers,” *Carbohydrate Research*, vol. 104, no. 1, pp. 87–104, Jun. 1982, doi: 10.1016/S0008-6215(00)82223-5.

[7] P. V. Murphy, J. L. O’Brien, L. J. Gorey-Feret, and A. B. Smith, “Synthesis of novel HIV-1 protease inhibitors based on carbohydrate scaffolds,” *Tetrahedron*, vol. 59, no. 13, pp. 2259–2271, Mar. 2003, doi: 10.1016/S0040-4020(03)00208-4.

[8] Y. Yoneda, T. Kawada, T. Rosenau, and P. Kosma, “Synthesis of methyl 4′-O-methyl-13C12-β-d-cellobioside from 13C6-d-glucose. Part 1: Reaction optimization and synthesis,” *Carbohydrate Research*, vol. 340, no. 15, pp. 2428–2435, Oct. 2005, doi: 10.1016/j.carres.2005.08.003.

[9] A. Roën, J. I. Padrón, and J. T. Vázquez, “Hydroxymethyl Rotamer Populations in Disaccharides,” *J. Org. Chem.*, vol. 68, no. 12, pp. 4615–4630, Jun. 2003, doi: 10.1021/jo026913o.

[10] M. E. L. Sánchez, V. Michelet, I. Besmier, J. P. Genet, “Convient Methods for the Synthesis of β-C-Glycosyl Aldehydes,” *Synlett*, vol. 1994, no. 9, pp. 705-708, 1994, doi: 10.1055/s-1994-22978.

[11] G. M. Sheldrick, “A short history of SHELX,” *Acta Crystallographica Section A: Foundations of Crystallography*, vol. 64, no. 1, pp. 112-122, 2008, doi: 10.1107/S0108767307043930.

[12] S. P. Westrip, “publCIF: software for editing, validating and formatting crystallographic information files,” *J. Appl. Cryst.,* vol. 43, no. 4, pp. 920-925, August 2010, doi: 10.1107/S0021889810022120.

[13] MacroModel; Schrödinger LLC.: New York, NY, USA, 2015. Available online: http://www.schrodinger.com/MacroModel

[14] Frisch, M.J.; Trucks, G.W.; Schlegel, H.B.; Scuseria, G.E.; Robb, M.A.; Cheeseman, J.R.; Scalmani, G.; Barone, V.; Petersson, G.A.; Nakatsuji, H.; et al. Gaussian 16, Revision C.02; Gaussian, Inc.: Wallingford, CT, USA, 2019.

[15] A. D. Becke, Density-functional thermochemistry. III. The role of exact exchange, *J. Chem. Phys.* **1993**, *98*, 5648-5652.

[16] Chai, J.-D.; Head-Gordon, M. Systematic optimization of longrange corrected hybrid density functionals. *J. Chem. Phys.* **2008**, *128*, 084106.

[17] Stephens, P.J.; Harada, N. ECD cotton effect approximated by the Gaussian curve and other methods. *Chirality* **2010**, *22*, 229-233.

[18] El-Kashef, D.H.; Daletos, G.; Plenker, M.; Hartmann, R.; Mándi, A.; Kurtán, T.; Weber, H.; Lin, W.; Ancheeva, E.; Proksch, P. Polyketides and a Dihydroquinolone Alkaloid from a Marine-Derived Strain of the Fungus *Metarhizium marquandii*. *J. Nat. Prod.* **2019**, *82*, 2460-2469.

[19] Varetto, U. MOLEKEL 5.4; Swiss National Supercomputing Centre: Manno, Switzerland, 2009.

[20] C. R. Harris, K. J. Millman, S. J. van der Walt, R. Gommers, P. Virtanen, D. Cournapeau, E. Wieser, J. Taylor, S. Berg, N. J. Smith, R. Kern, M. Picus, S. Hoyer, M. H. van Kerkwijk, M. Brett, A. Haldane, J. F. del Río, M. Wiebe, P. Peterson, P. Gérard-Marchant, K. Sheppard, T. Reddy, W. Weckesser, H. Abbasi, C. Gohlke, T. E. Oliphant: Array programming with NumPy, *Nature* **2020**, *585*, 357-362.

COPY of NMR SPECTRA

Compound 2

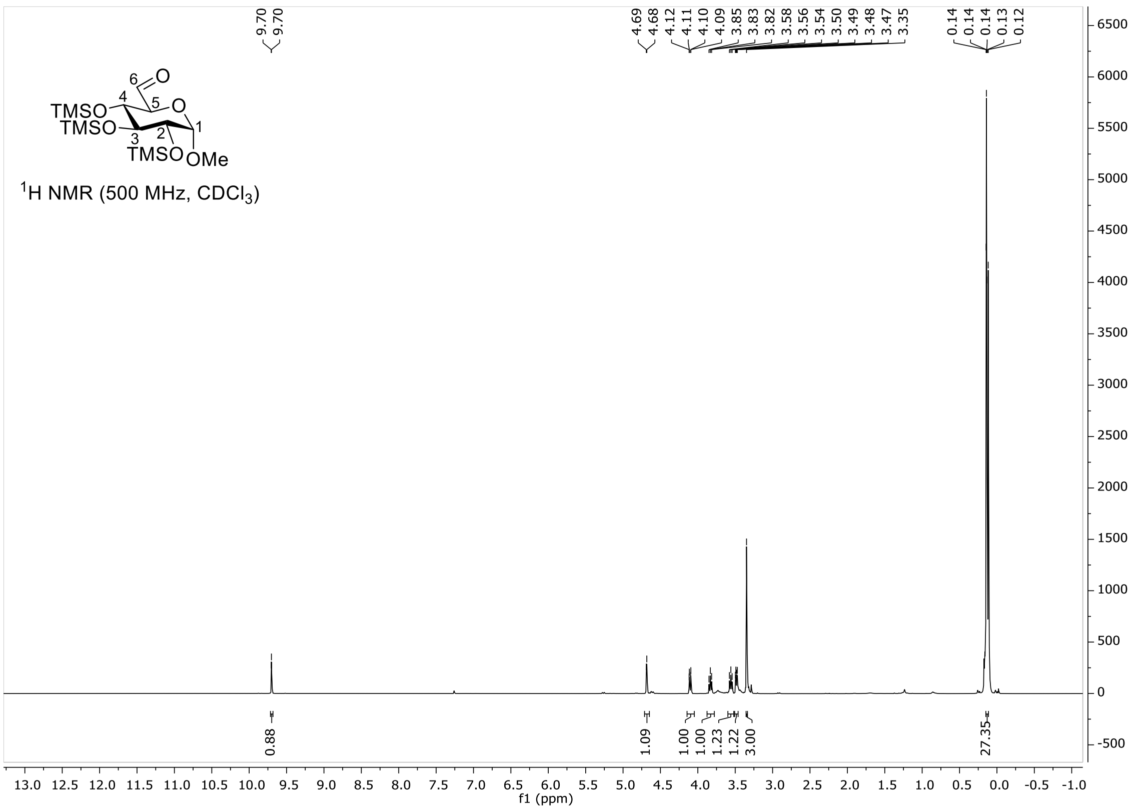

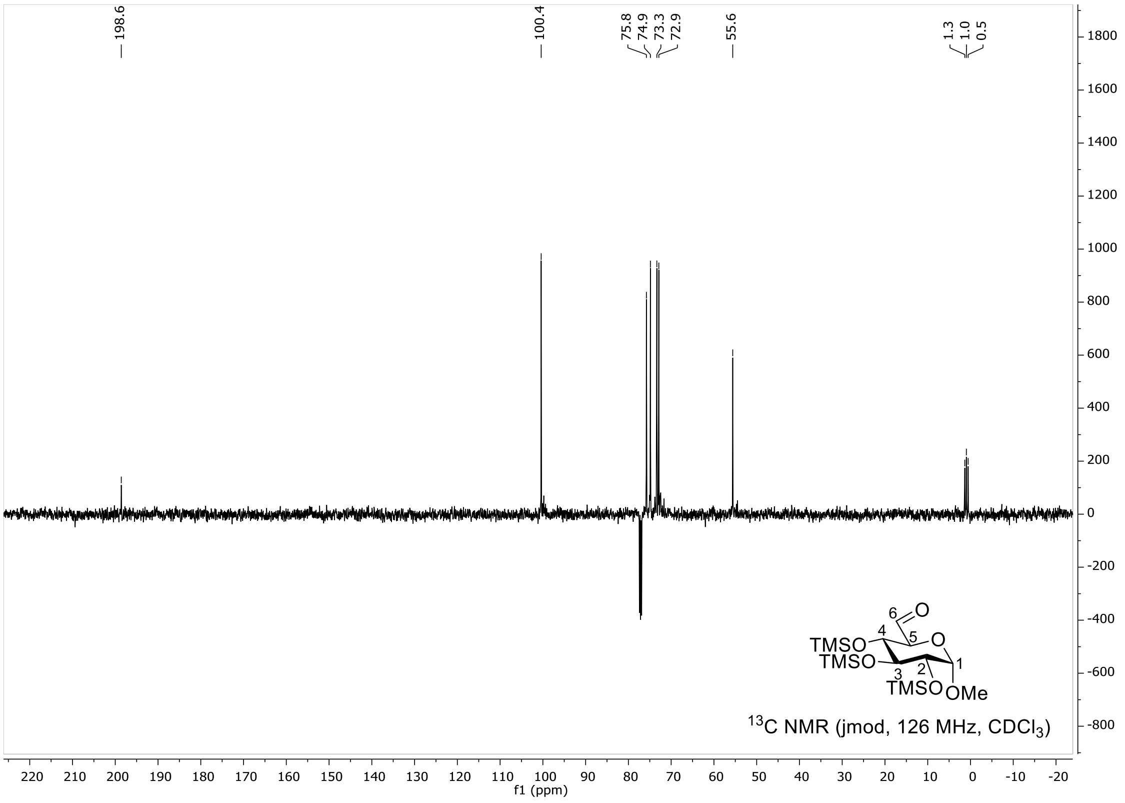

Compound 6.2

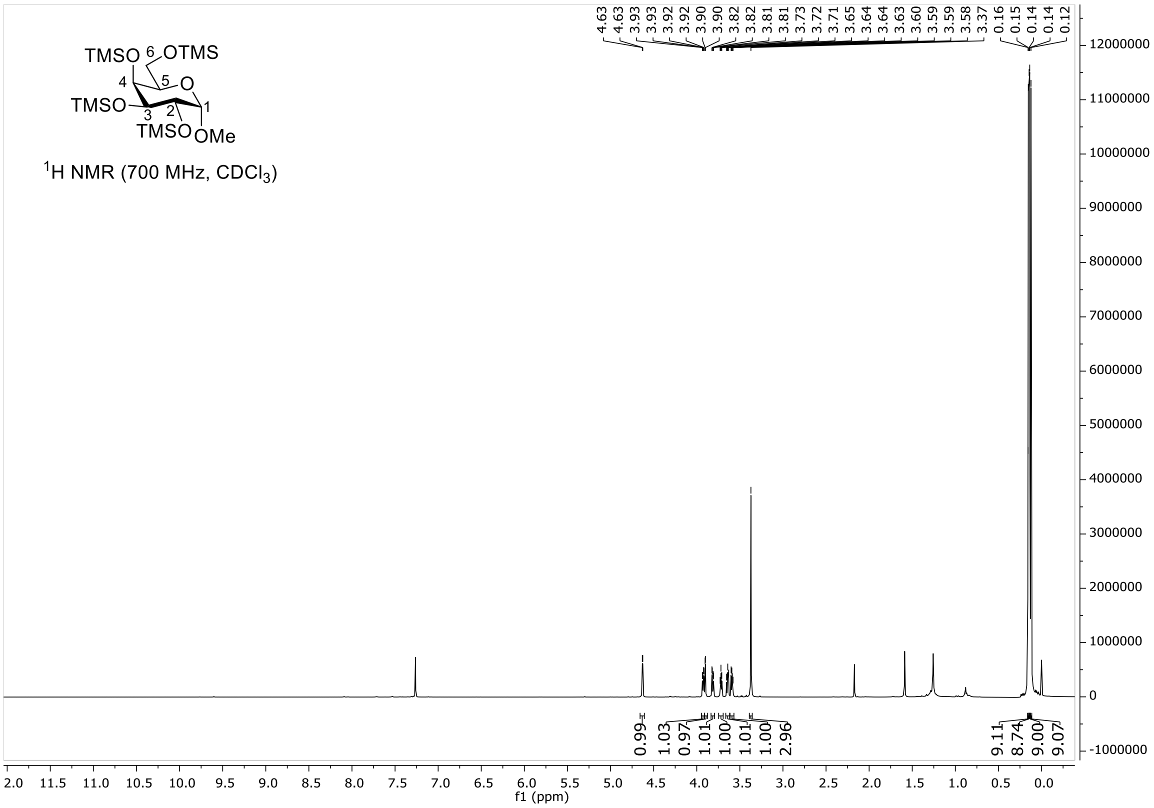

TMSO 6 OTMS
4 5 O
TMSO 3 2 1
TMSO OMe
1H NMR (700 MHz, CDCl3)
f1 (ppm)

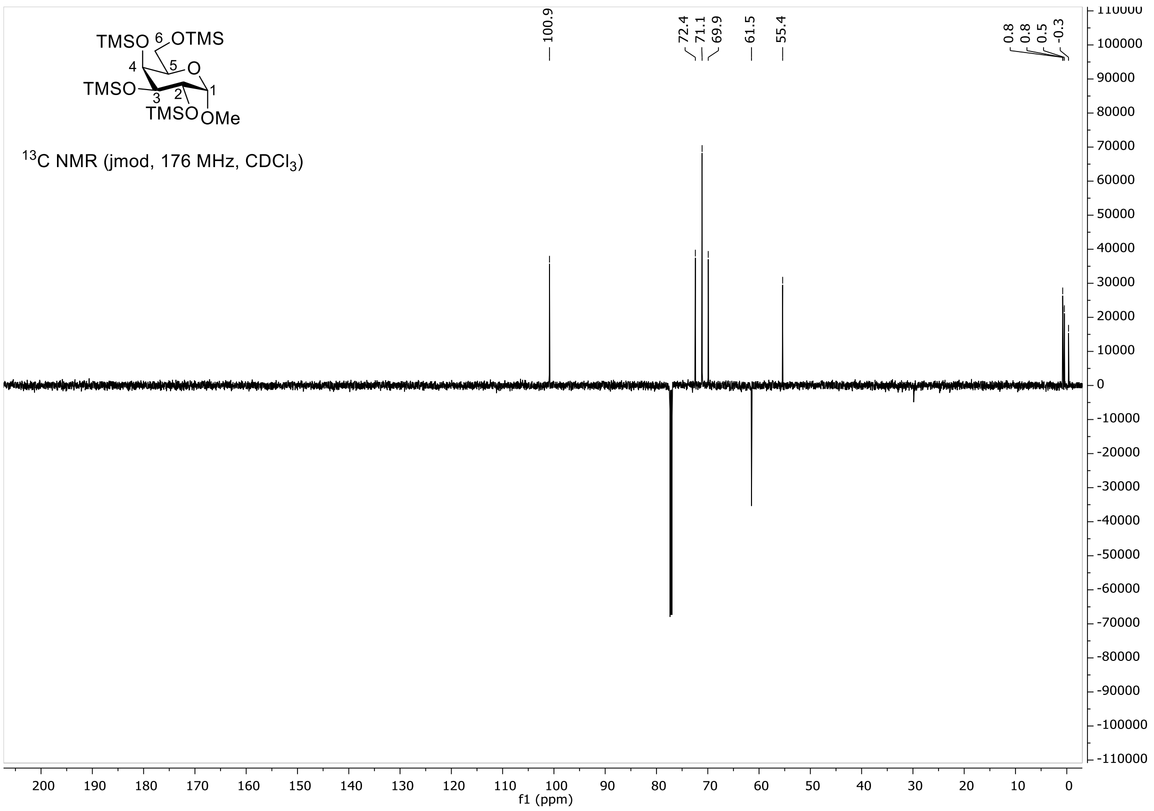

TMSO 6 OTMS
4 5 O
TMSO 3 2 1
TMSO OMe
13C NMR (jmod, 176 MHz, CDCl3)
f1 (ppm)

Compound 6

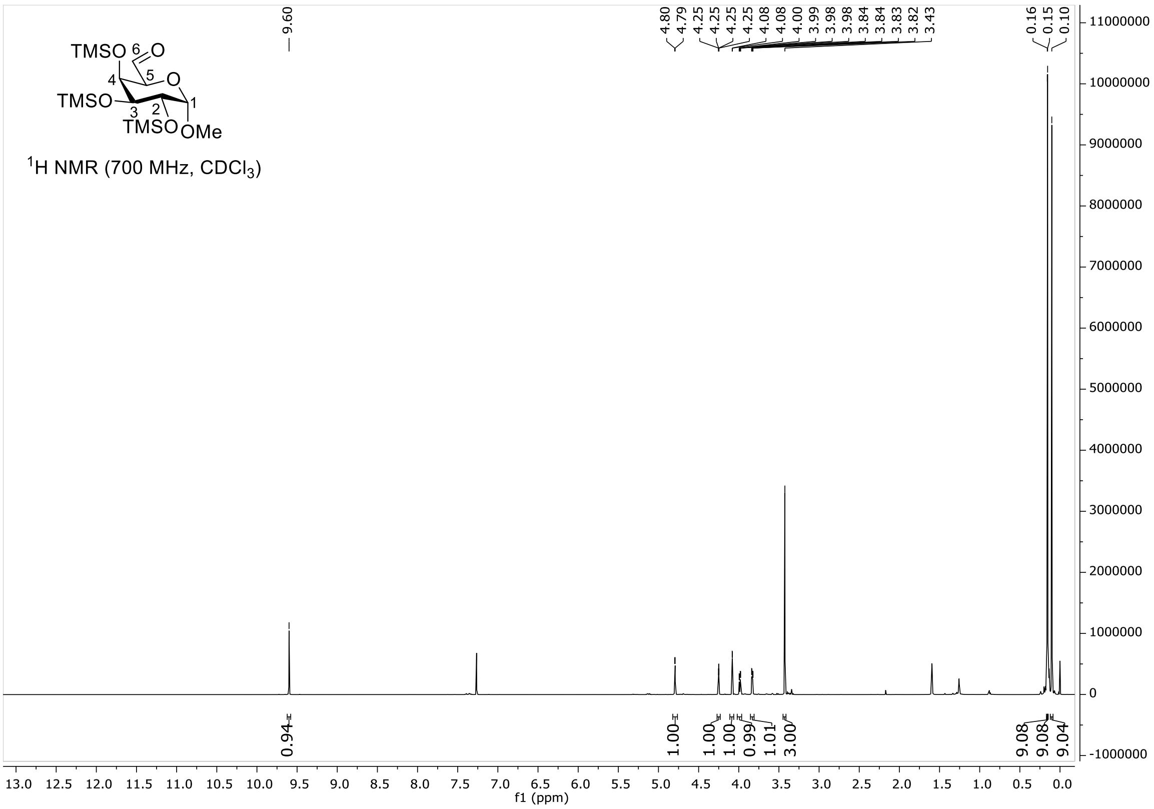
TMSO
O
TMSO
TMSO
OMe
¹H NMR (700 MHz, CDCl₃)
f1 (ppm)

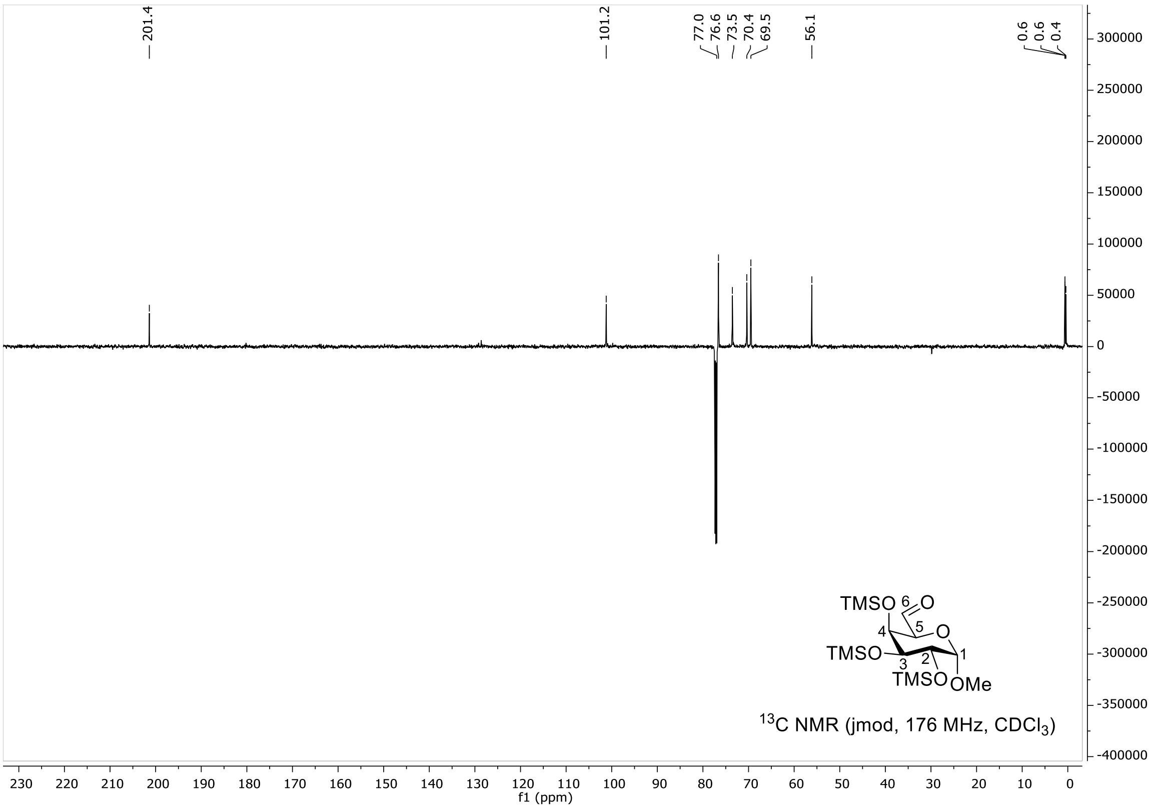
TMSO
O
TMSO
TMSO
OMe
¹³C NMR (jmod, 176 MHz, CDCl₃)
f1 (ppm)

Compound 14

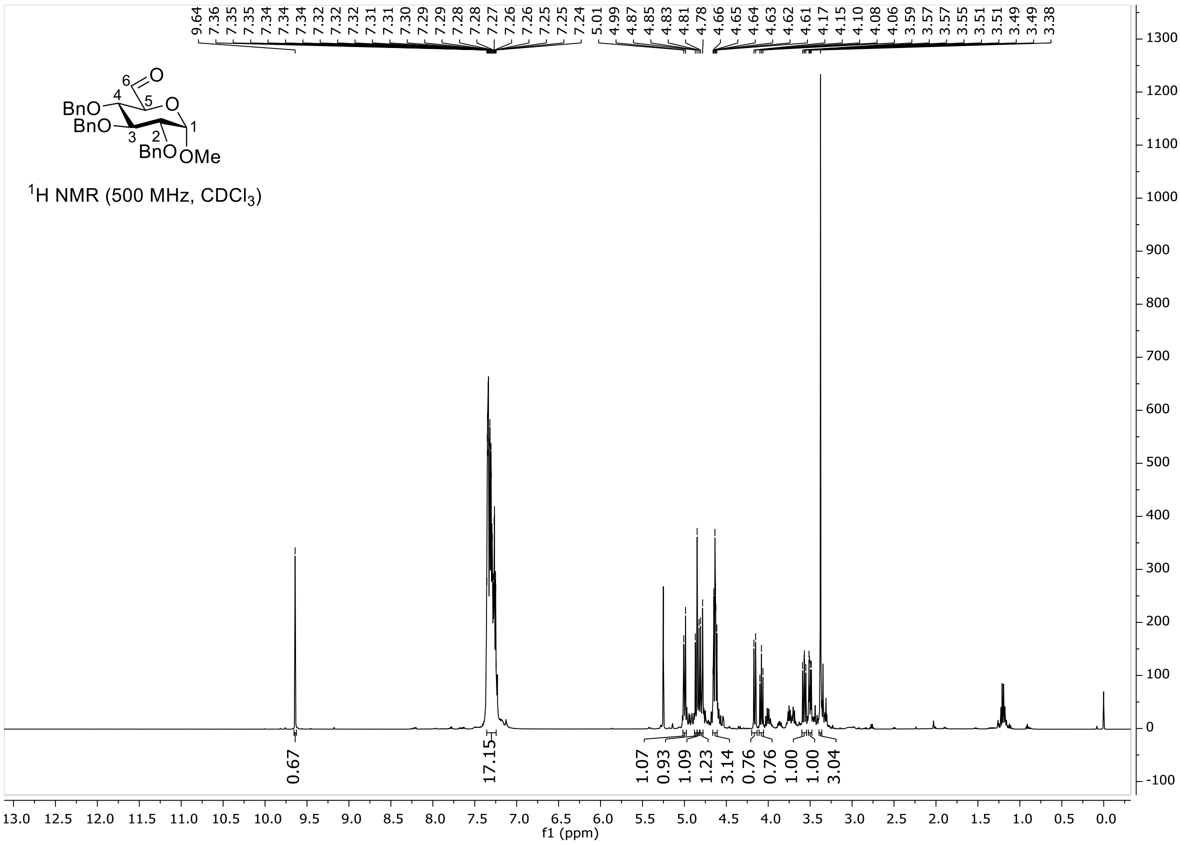
9.64 7.36 7.35 7.35 7.34 7.34 7.34 7.32 7.32 7.32 7.31 7.31 7.30 7.29 7.29 7.28 7.28 7.27 7.26 7.26 7.25 7.25 7.24 5.01 4.99 4.87 4.85 4.83 4.81 4.78 4.66 4.65 4.64 4.63 4.62 4.61 4.17 4.15 4.10 4.08 4.06 3.59 3.57 3.57 3.55 3.51 3.51 3.49 3.38
BnO BnO BnO OMe O 1 2 3 4 5 6
1H NMR (500 MHz, CDCl3)
0.67 17.15 1.07 0.93 1.09 1.23 3.14 0.76 0.76 1.00 1.00 3.04
f1 (ppm)

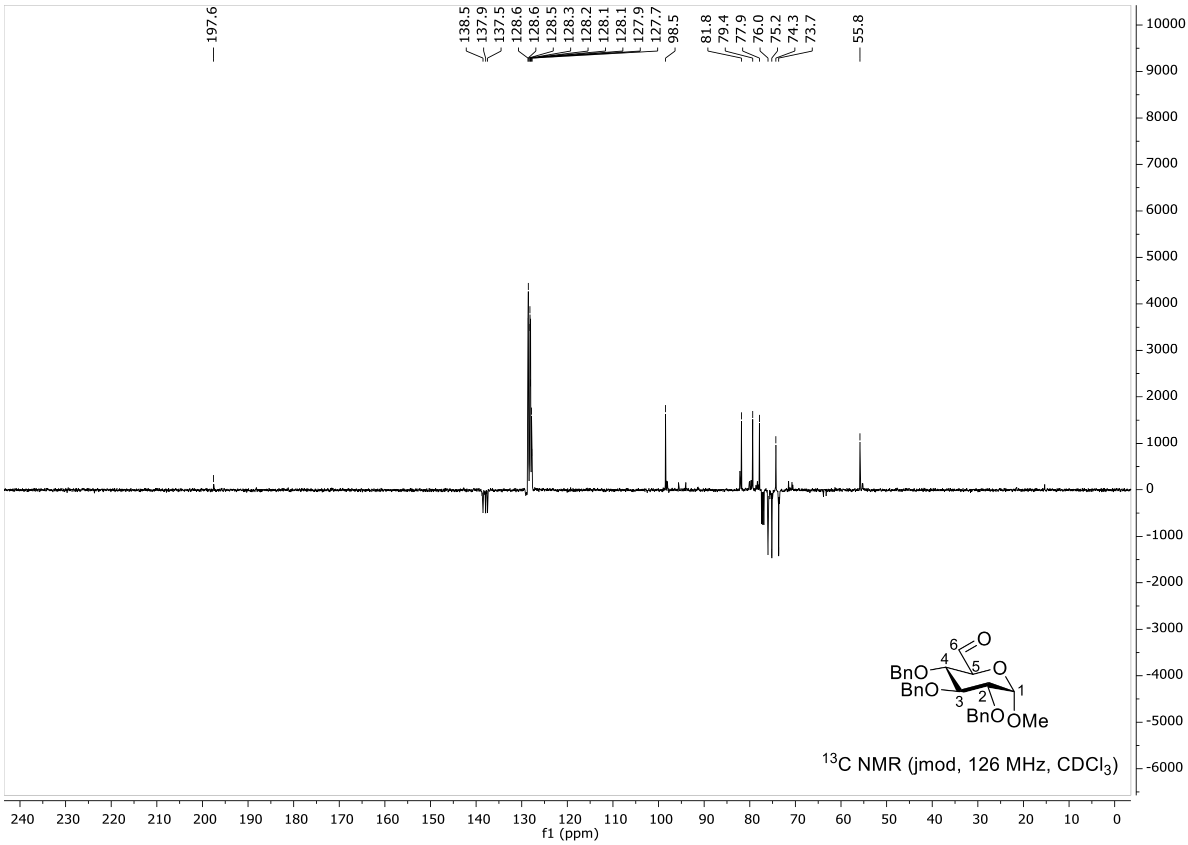
197.6 138.5 137.9 137.5 128.6 128.5 128.3 128.2 128.1 128.1 127.9 127.7 98.5 81.8 79.4 77.9 76.0 75.2 74.3 73.7 55.8
BnO BnO BnO OMe O 1 2 3 4 5 6
13C NMR (jmod, 126 MHz, CDCl3)
f1 (ppm)

Compound 15.3

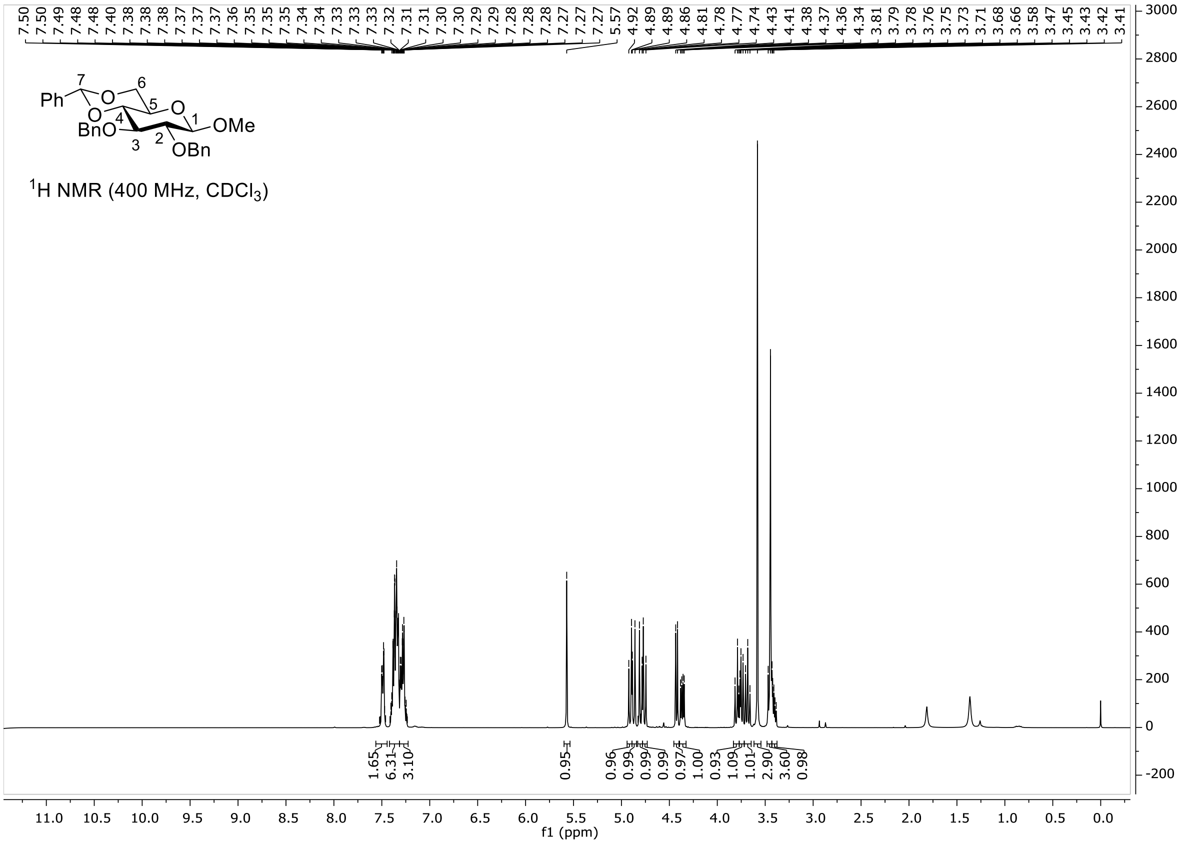

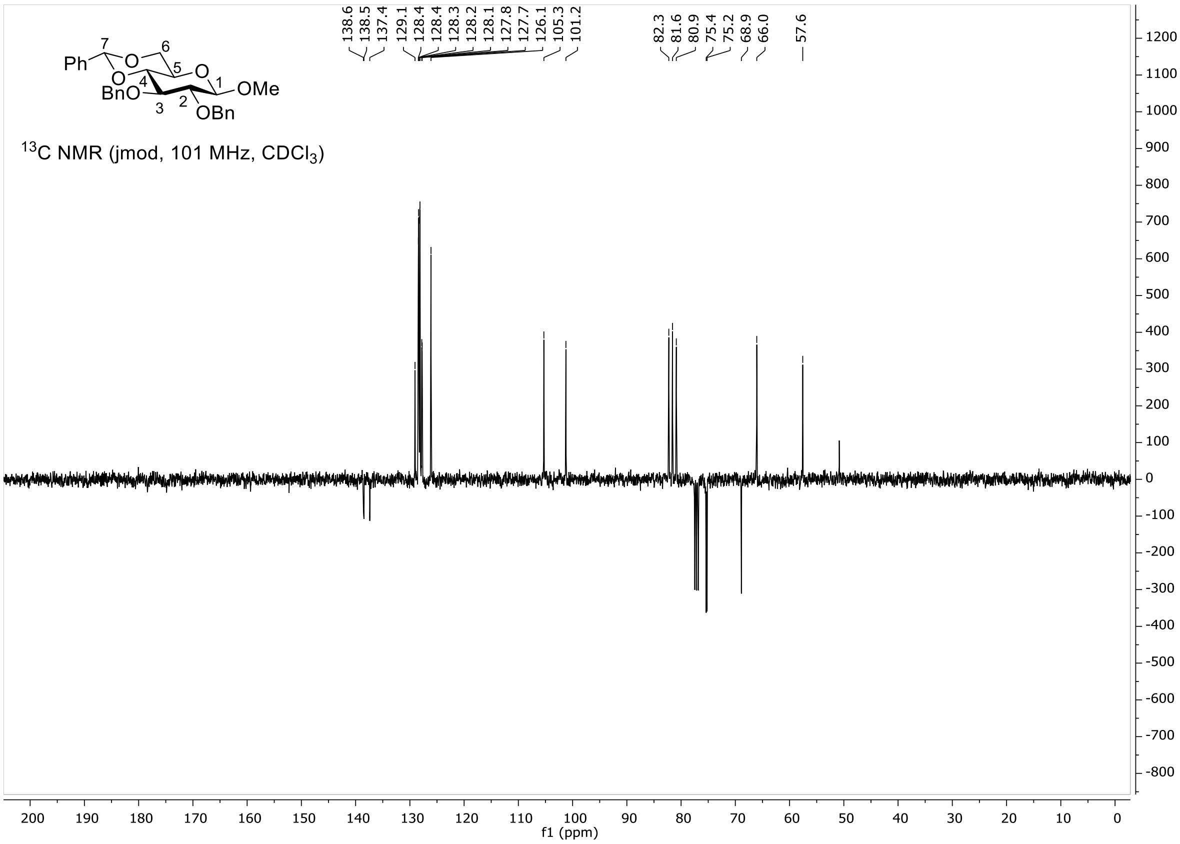

Compound 15.4

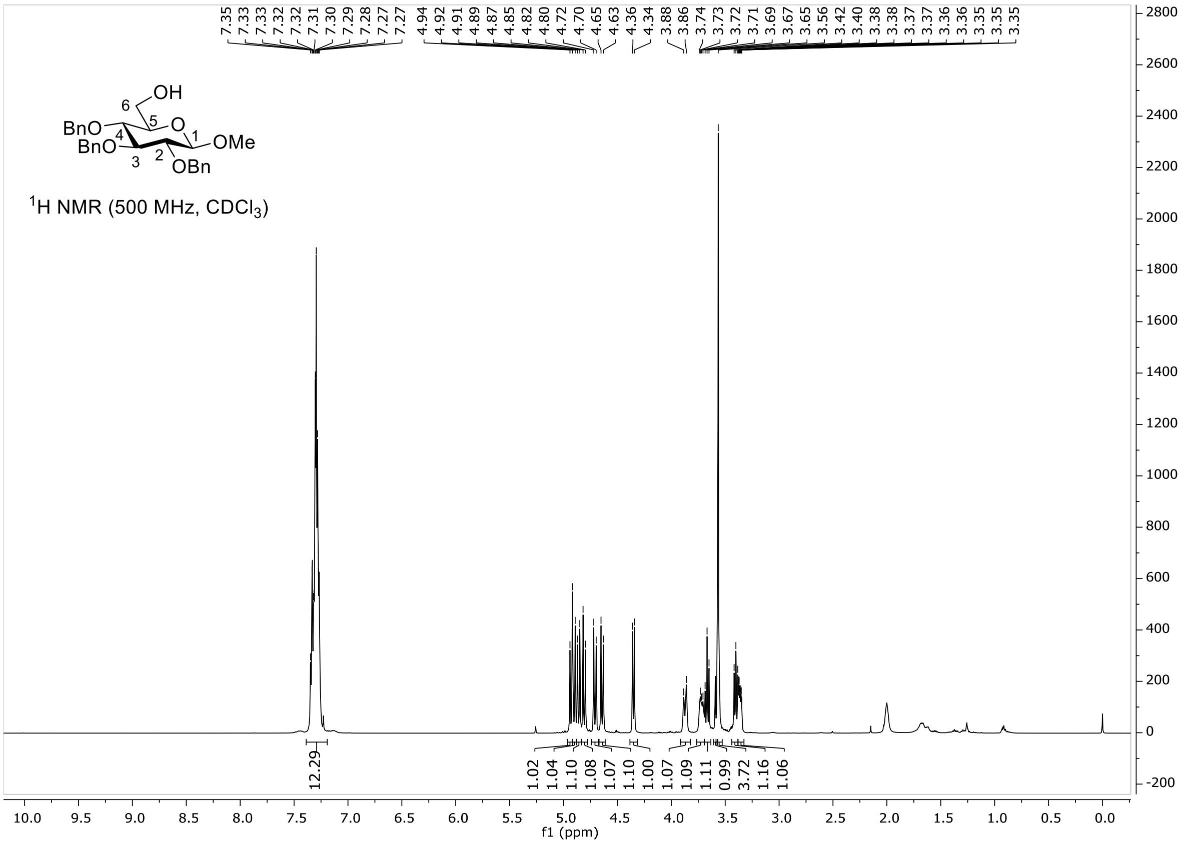

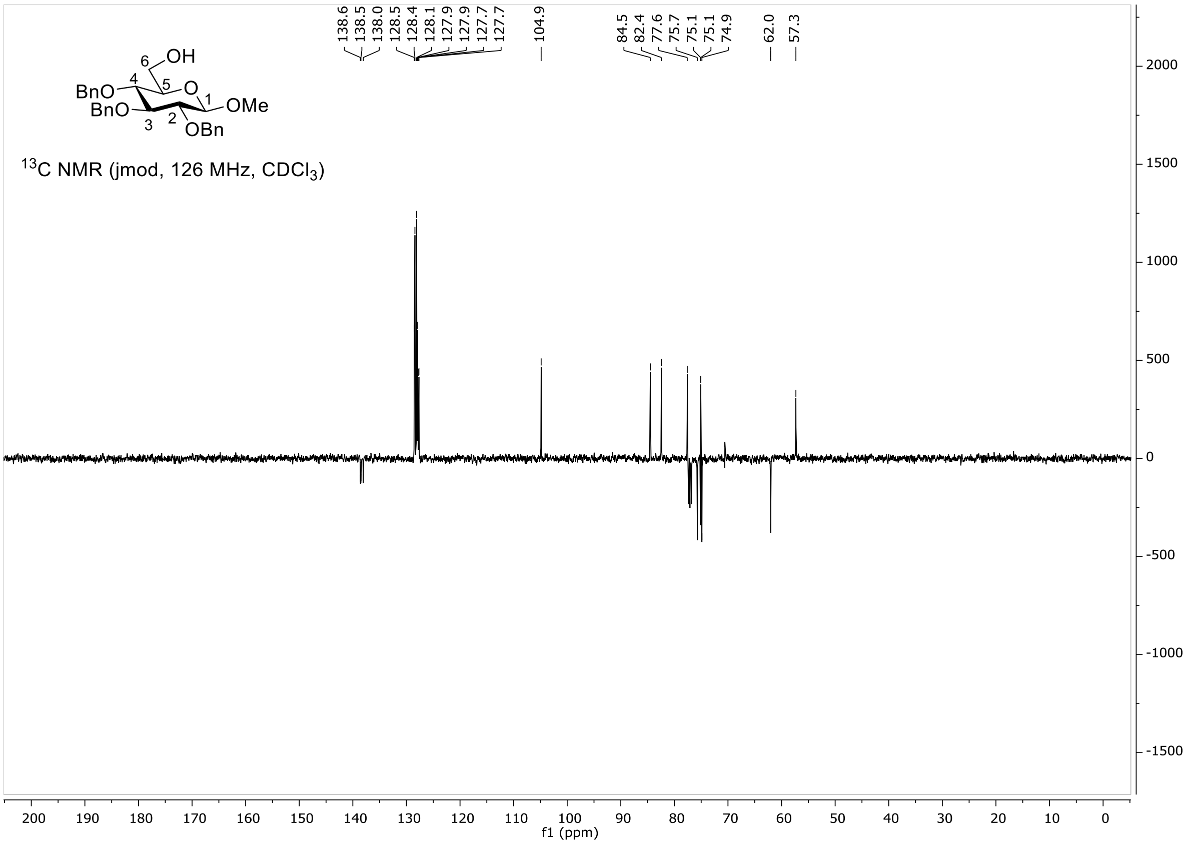

Compound 15

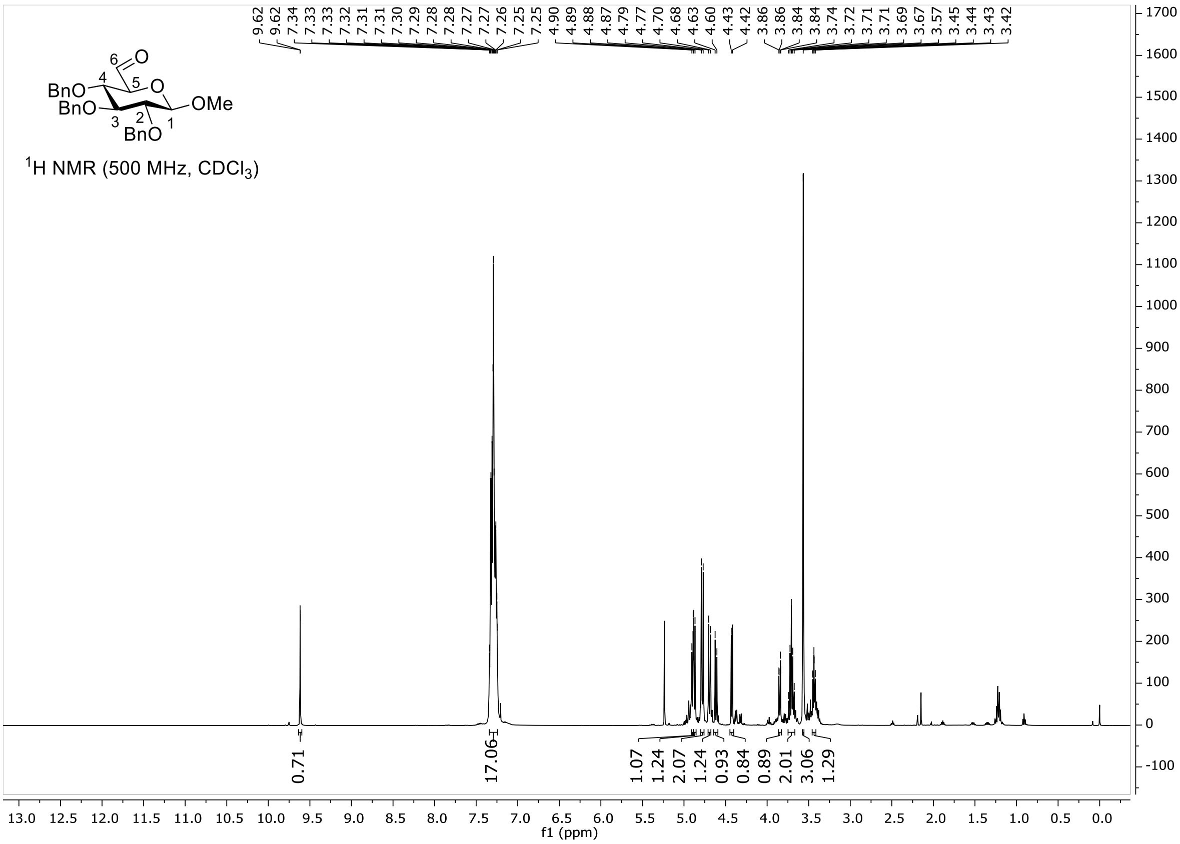

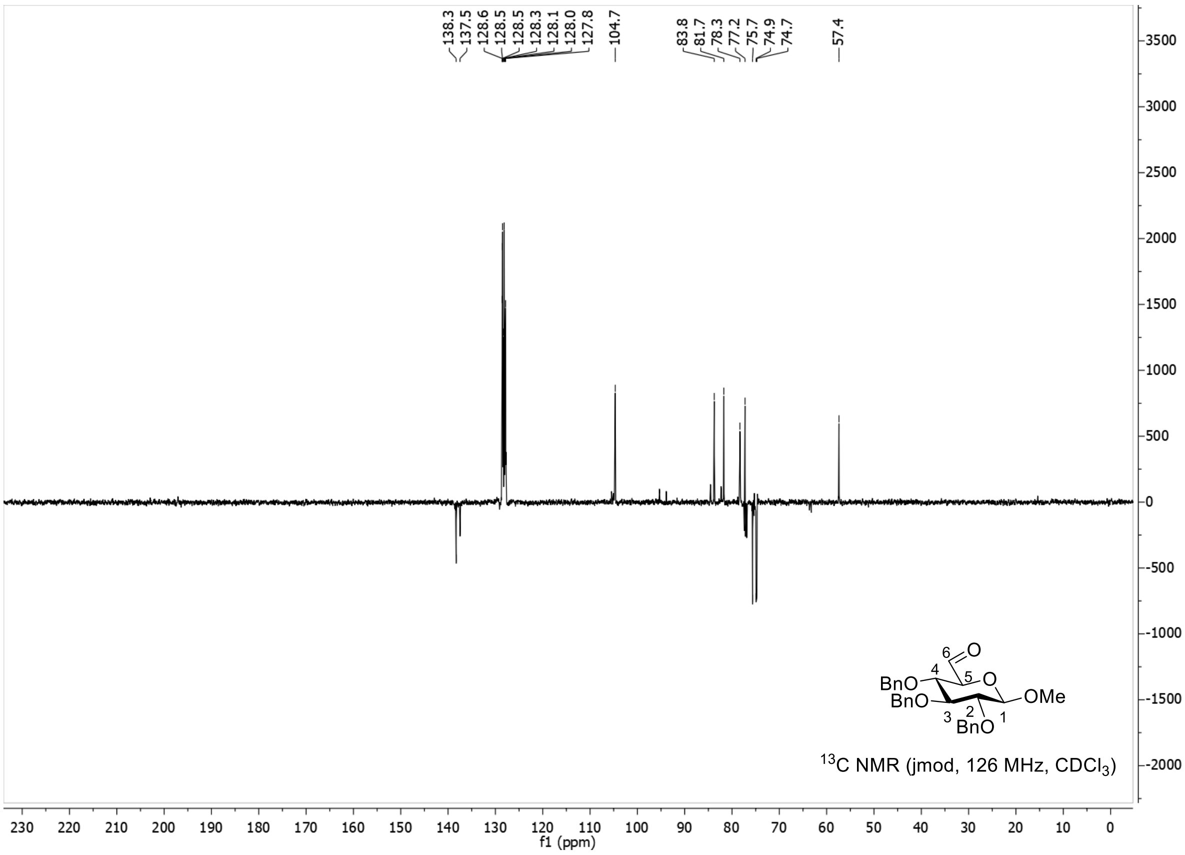

Compound 3

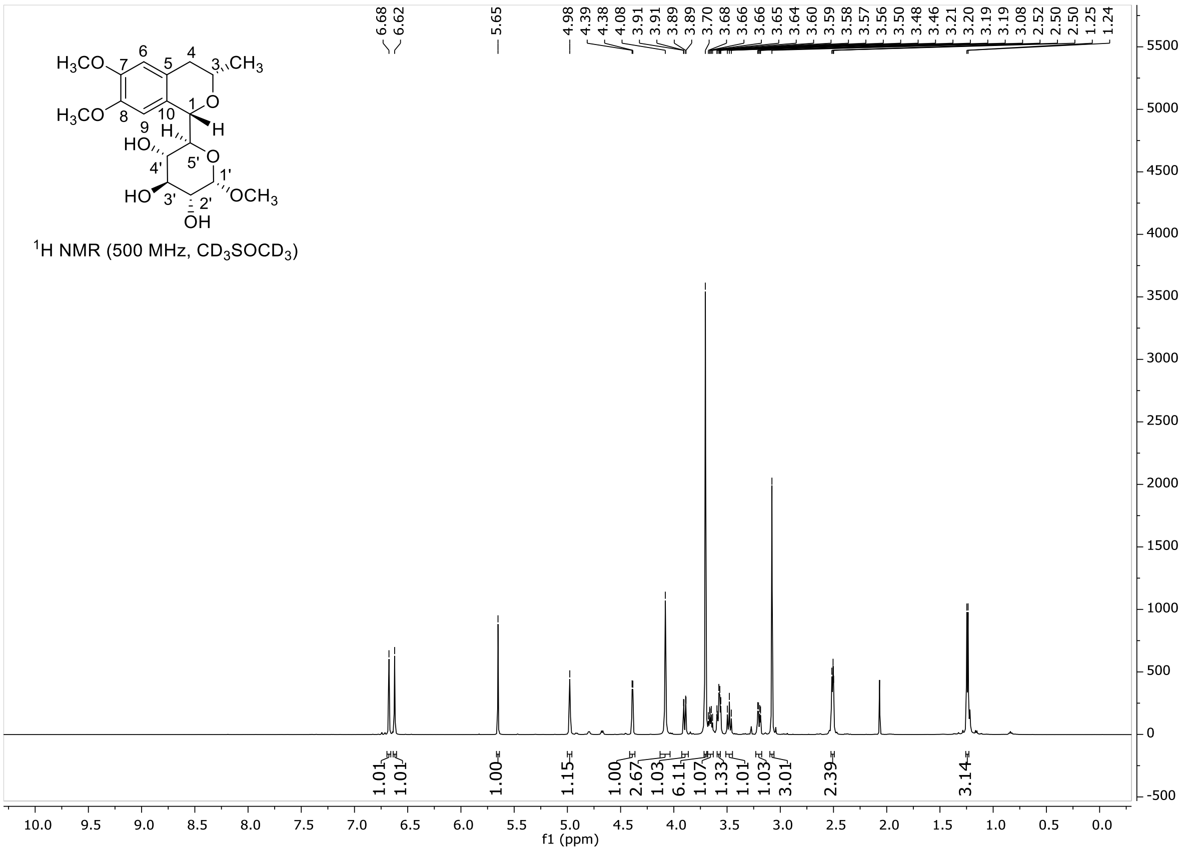
1H NMR (500 MHz, CD3SOCD3)
f1 (ppm)

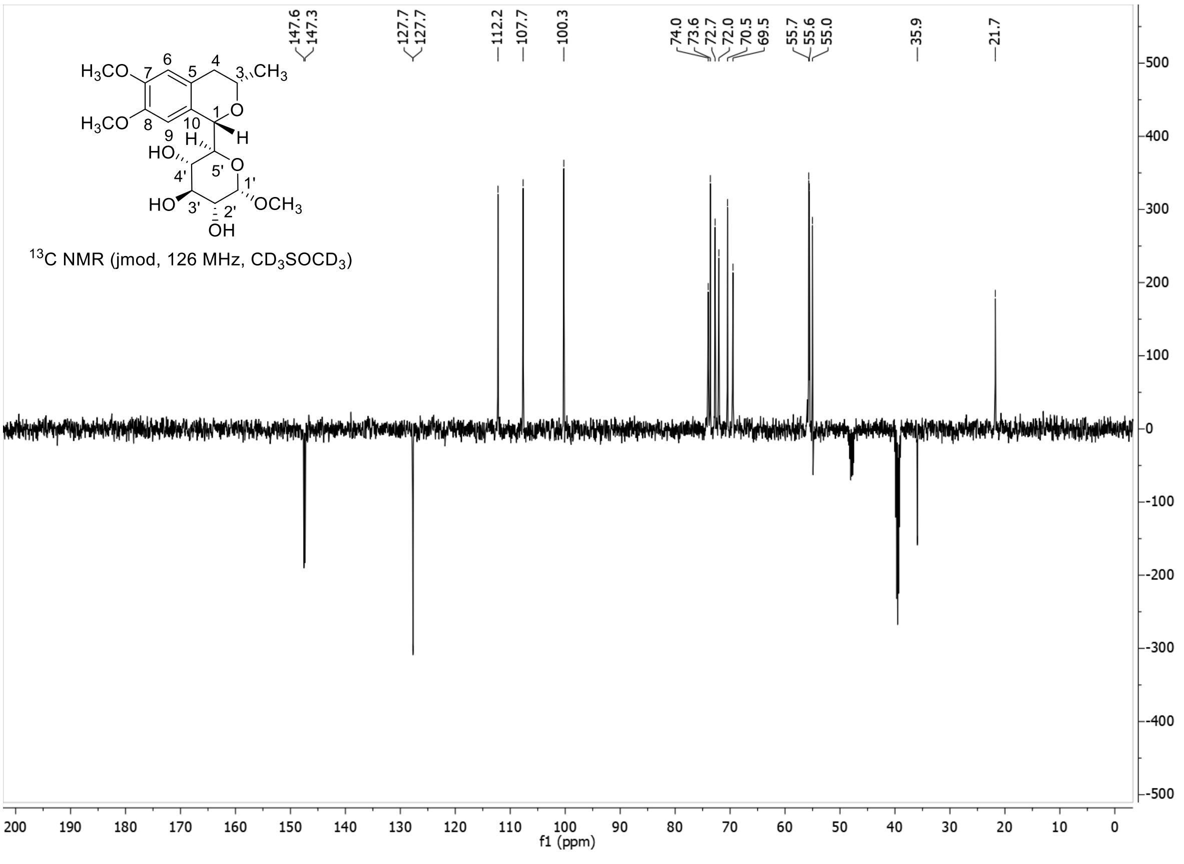
13C NMR (jmod, 126 MHz, CD3SOCD3)
f1 (ppm)

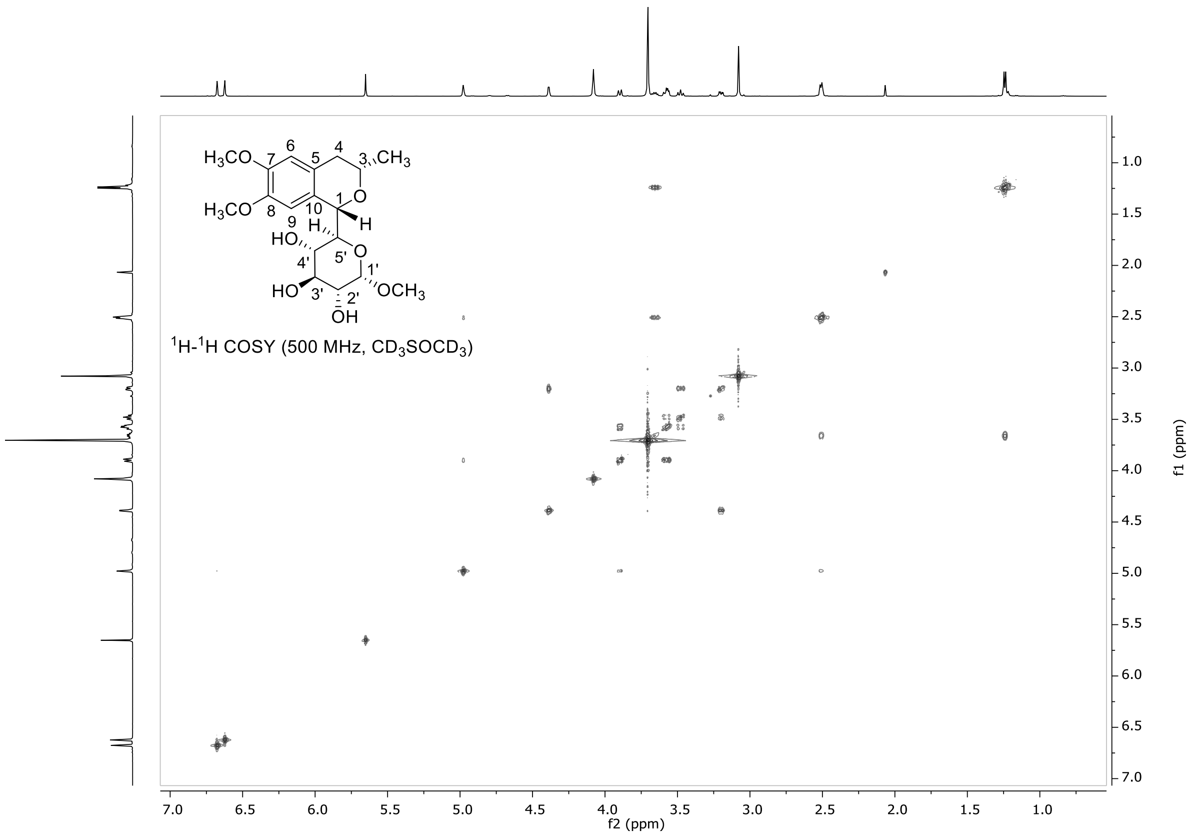
H3CO 7 6 5 4 3 CH3
H3CO 8 9 10 1 O H
H HO 4' 5' O 1'
HO 3' 2' OCH3 OH
1H-1H COSY (500 MHz, CD3SOCD3)
f2 (ppm)
f1 (ppm)

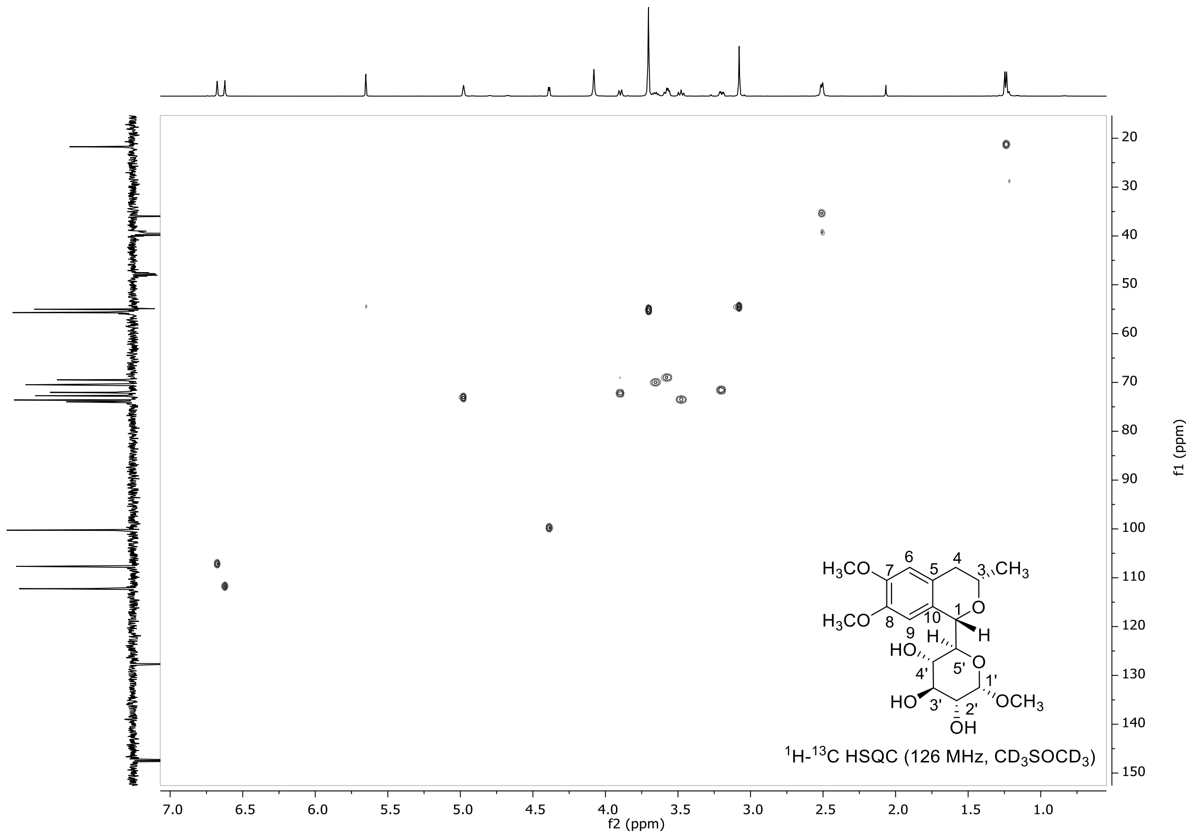
H3CO 7 6 5 4 3 CH3
H3CO 8 9 10 1 O H
H HO 4' 5' O 1'
HO 3' 2' OCH3 OH
1H-13C HSQC (126 MHz, CD3SOCD3)
f2 (ppm)
f1 (ppm)

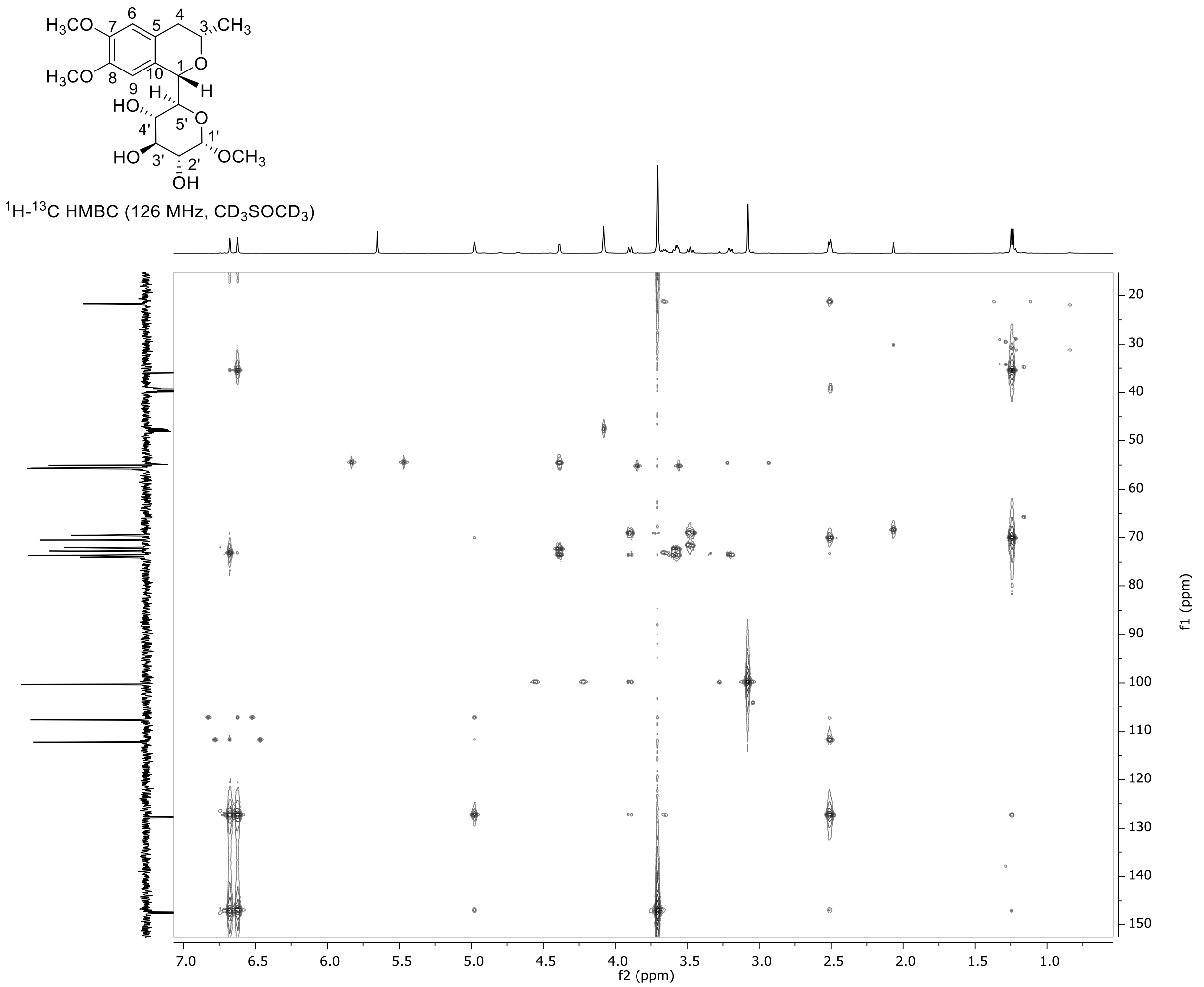

H3CO
H3CO
HO
HO
OH
OCH3
CH3
^{1}H-^{13}C HMBC (126 MHz, CD3SOCD3)
f2 (ppm)
f1 (ppm)

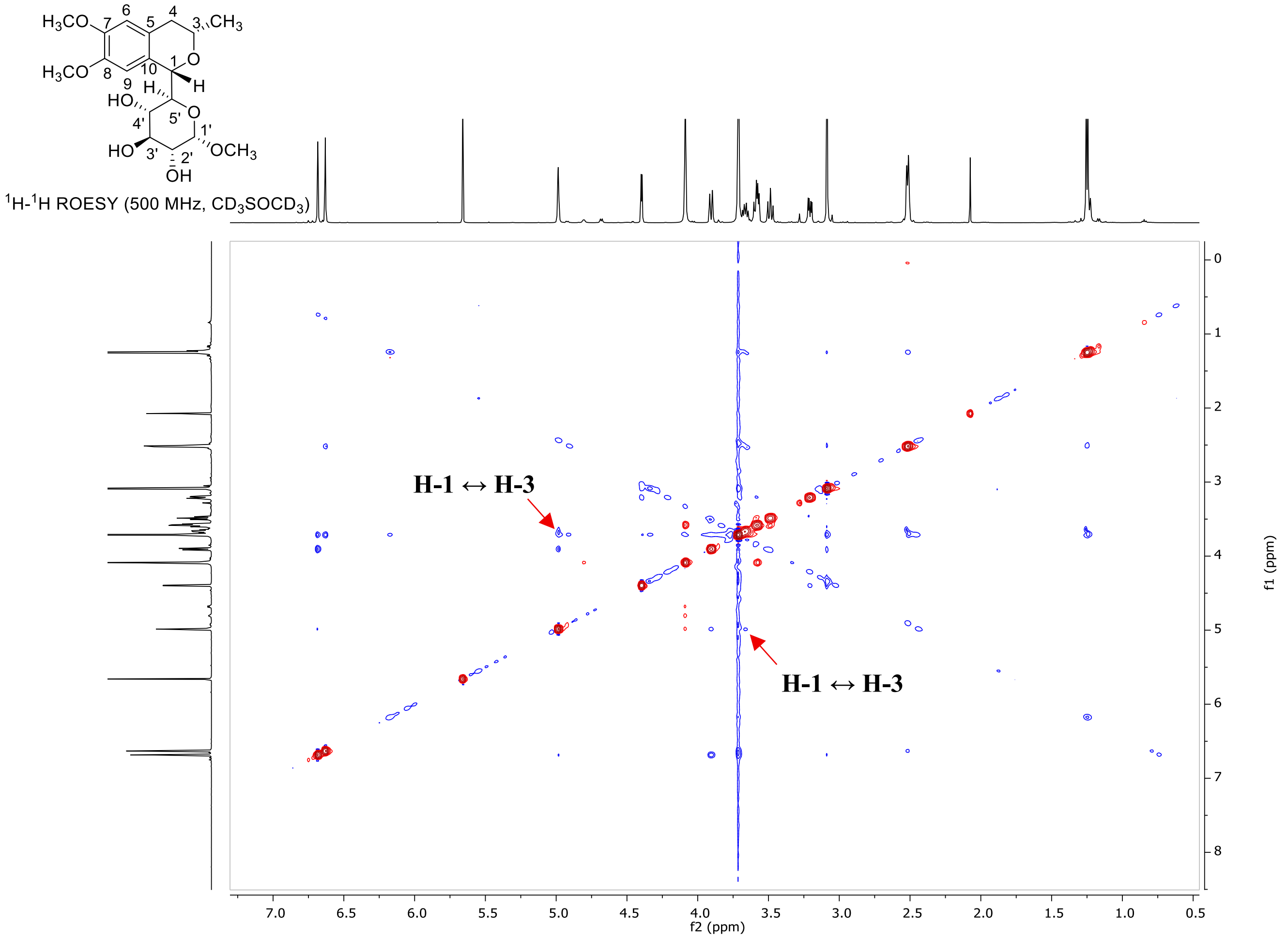

H3CO
H3CO
HO
HO
OH
OCH3
CH3
^{1}H-^{1}H ROESY (500 MHz, CD3SOCD3)
H-1 ↔ H-3
H-1 ↔ H-3
f2 (ppm)
f1 (ppm)

Compound 4

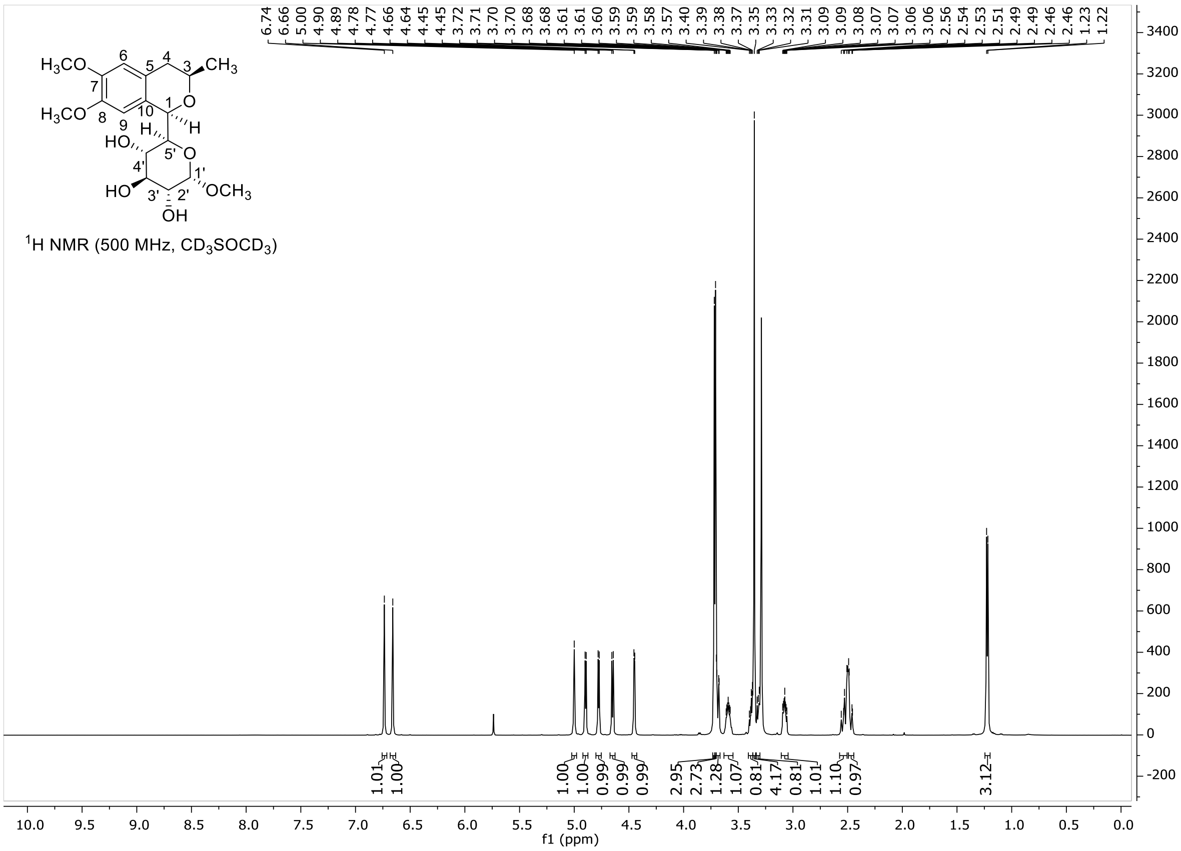
6.74 6.66 5.00 4.90 4.89 4.78 4.77 4.66 4.64 4.45 4.45 3.72 3.71 3.70 3.70 3.68 3.68 3.61 3.61 3.60 3.59 3.59 3.58 3.57 3.40 3.39 3.38 3.37 3.35 3.33 3.32 3.31 3.09 3.09 3.08 3.07 3.07 3.06 3.06 2.56 2.54 2.53 2.51 2.49 2.49 2.46 2.46 1.23 1.22
^{1}H NMR (500 MHz, CD_3SOCD_3)
1.01 1.00 1.00 1.00 0.99 0.99 0.99 2.95 2.73 1.28 1.07 0.81 4.17 0.81 1.01 1.10 0.97 3.12
f1 (ppm)

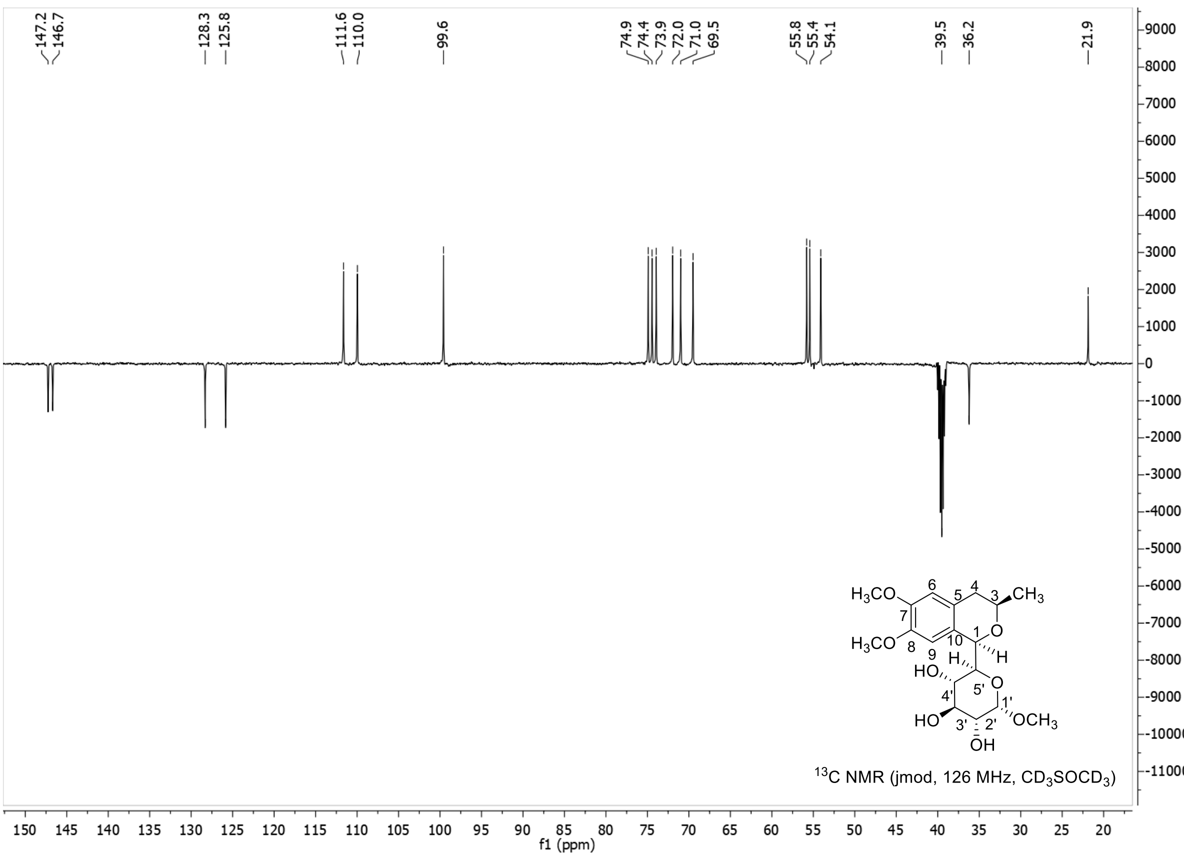
147.2 146.7 128.3 125.8 111.6 110.0 99.6 74.9 74.4 73.9 72.0 71.0 69.5 55.8 55.4 54.1 39.5 36.2 21.9
^{13}C NMR (jmod, 126 MHz, CD_3SOCD_3)
f1 (ppm)

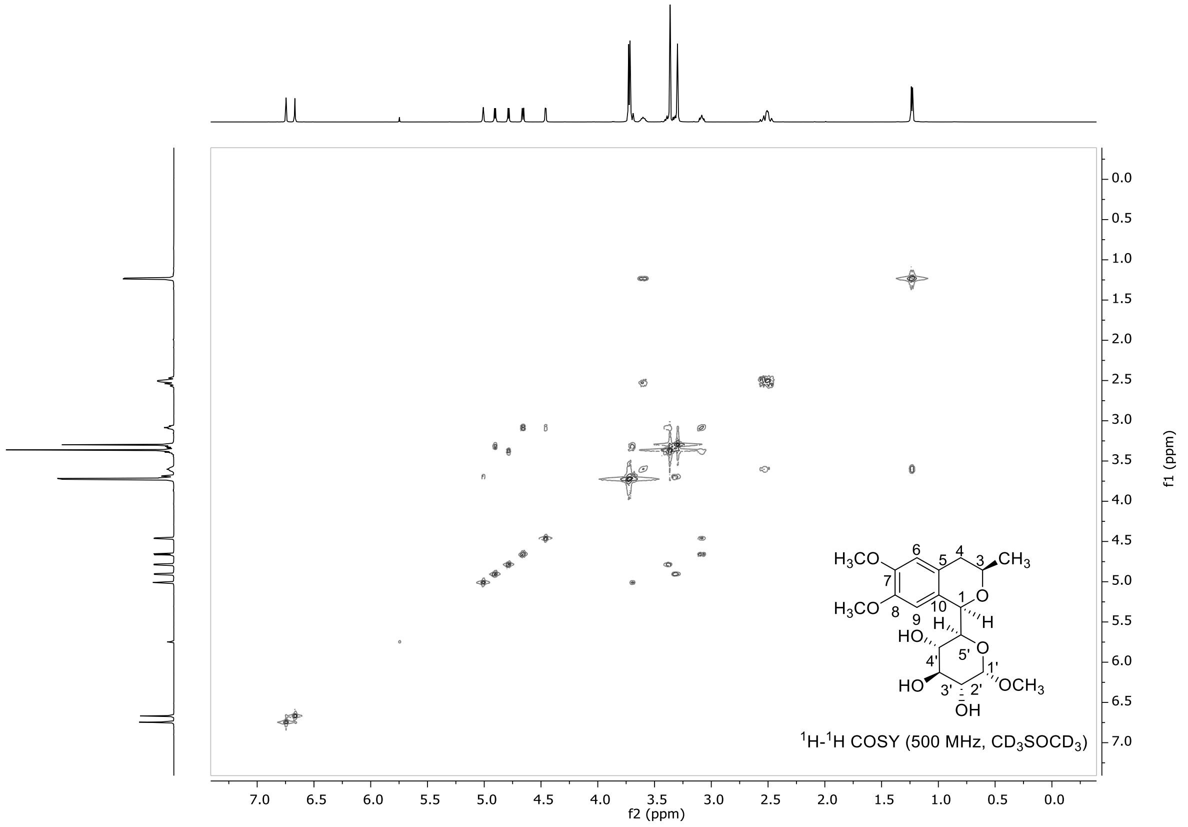
f2 (ppm)
f1 (ppm)
H3CO
H3CO
CH3
HO
HO
OH
OCH3
1H-1H COSY (500 MHz, CD3SOCD3)

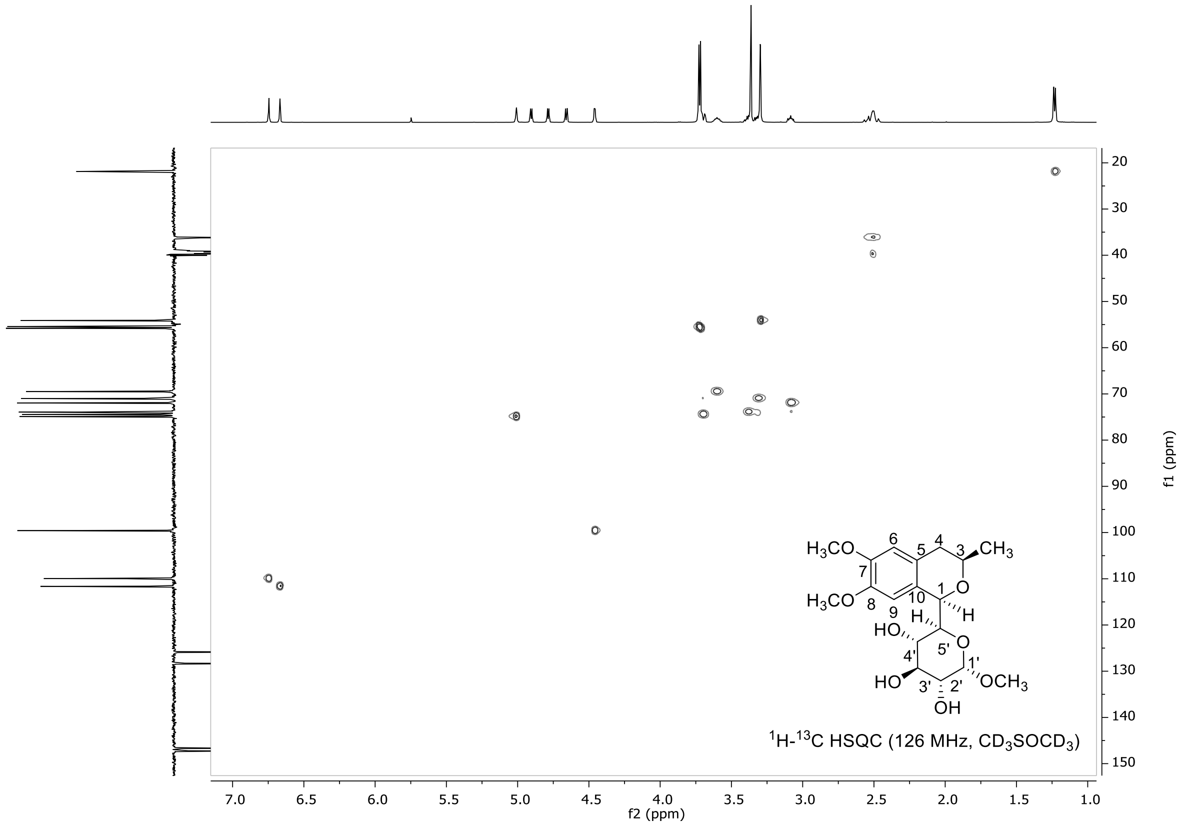
f2 (ppm)
f1 (ppm)
H3CO
H3CO
CH3
HO
HO
OH
OCH3
1H-13C HSQC (126 MHz, CD3SOCD3)

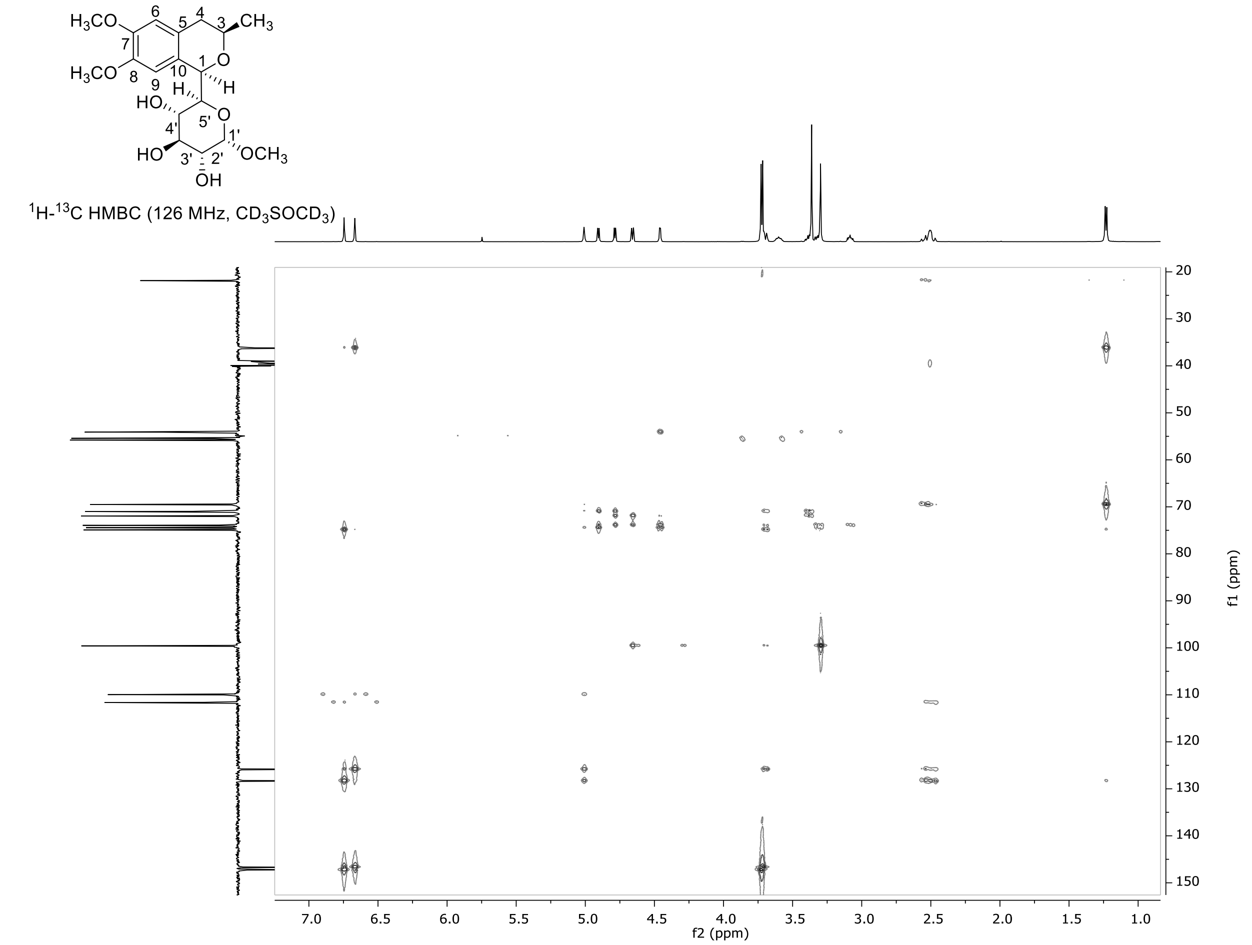
^{1}H-^{13}C HMBC (126 MHz, CD_3SOCD_3)
f2 (ppm)
f1 (ppm)

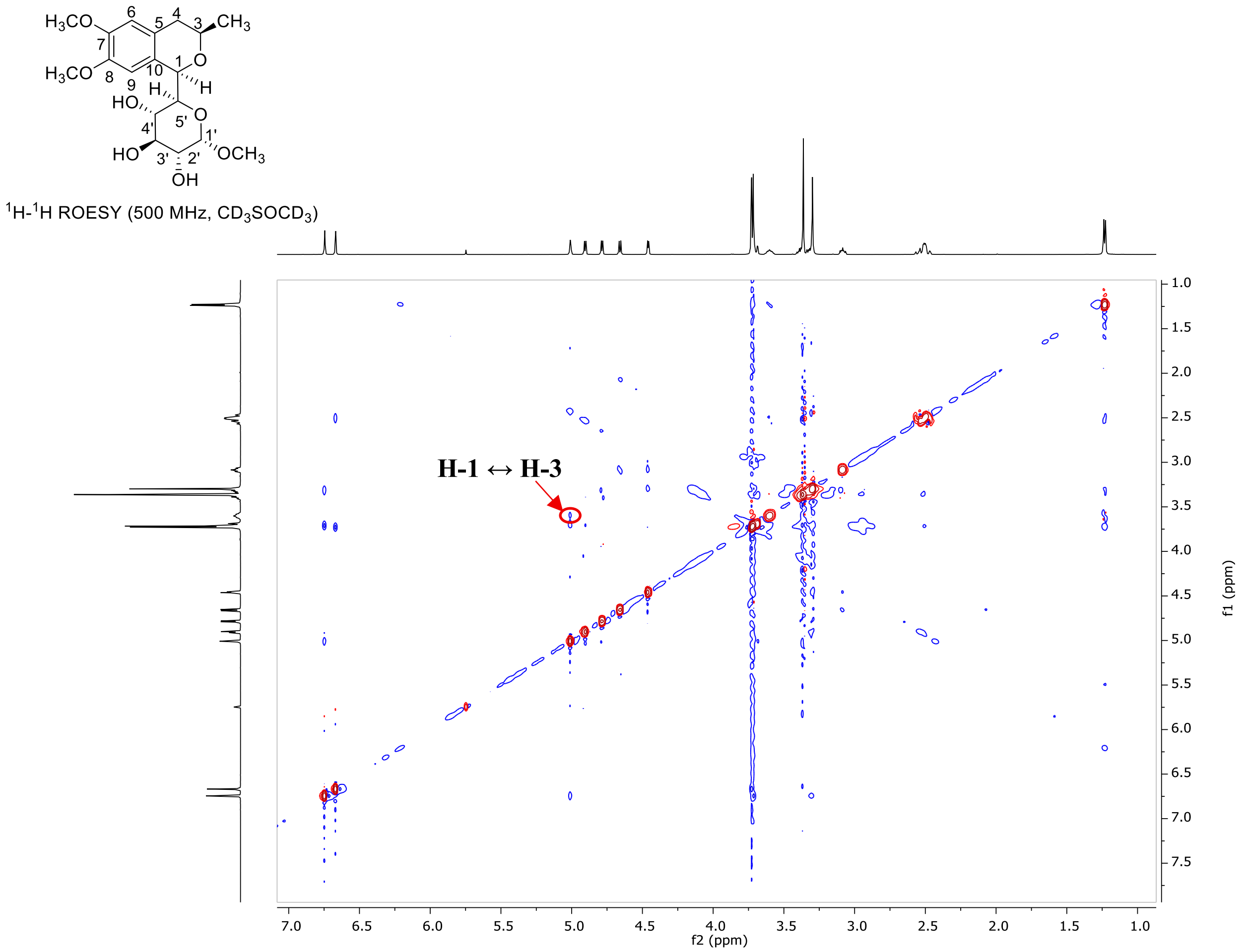
^{1}H-^{1}H ROESY (500 MHz, CD_3SOCD_3)
H-1 ↔ H-3
f2 (ppm)
f1 (ppm)

Compound 5

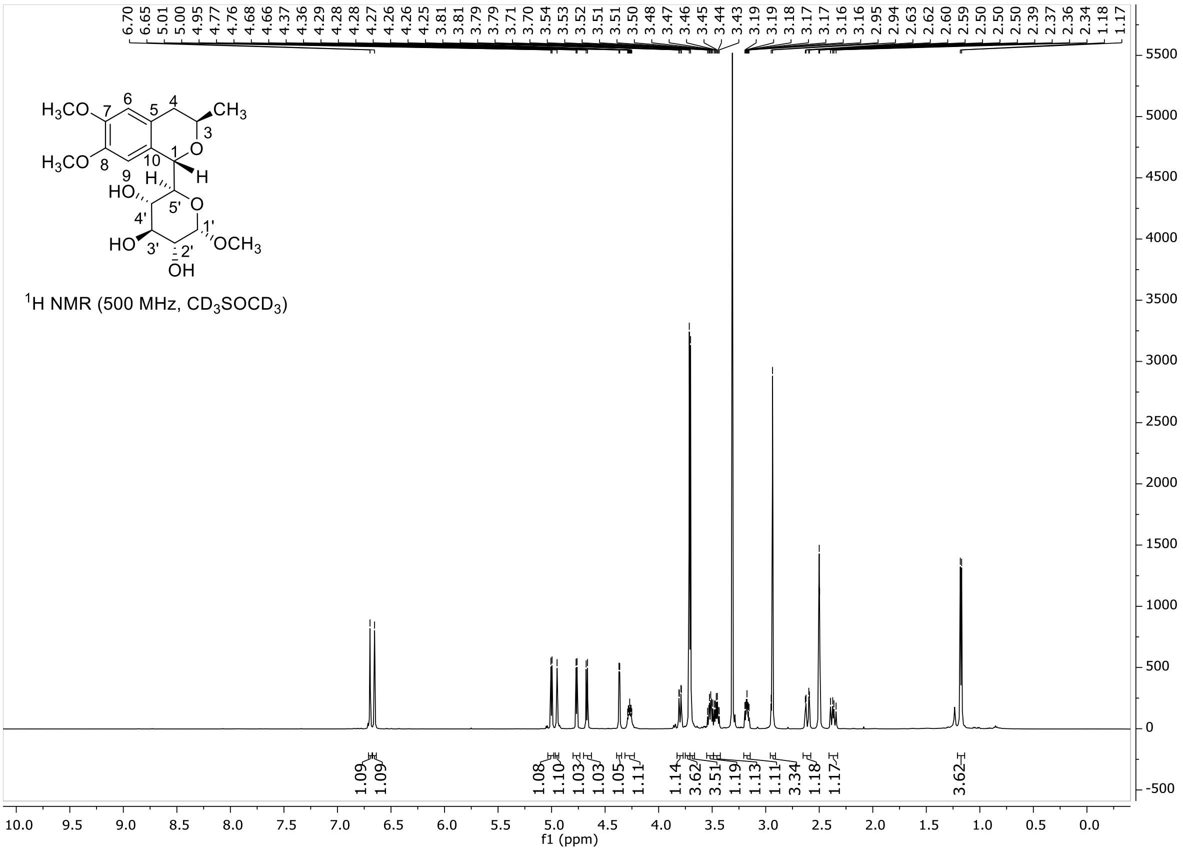

1H NMR (500 MHz, CD3SOCD3)
f1 (ppm)

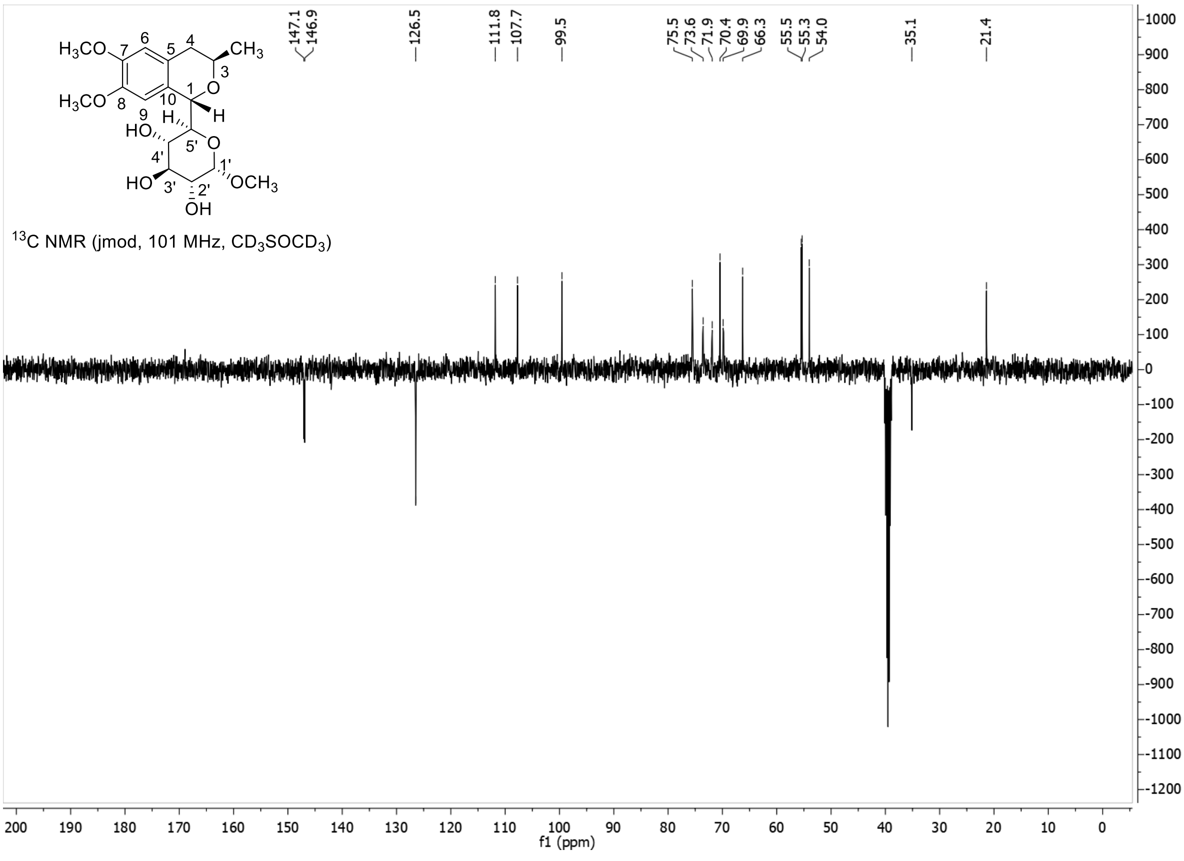

13C NMR (jmod, 101 MHz, CD3SOCD3)
f1 (ppm)

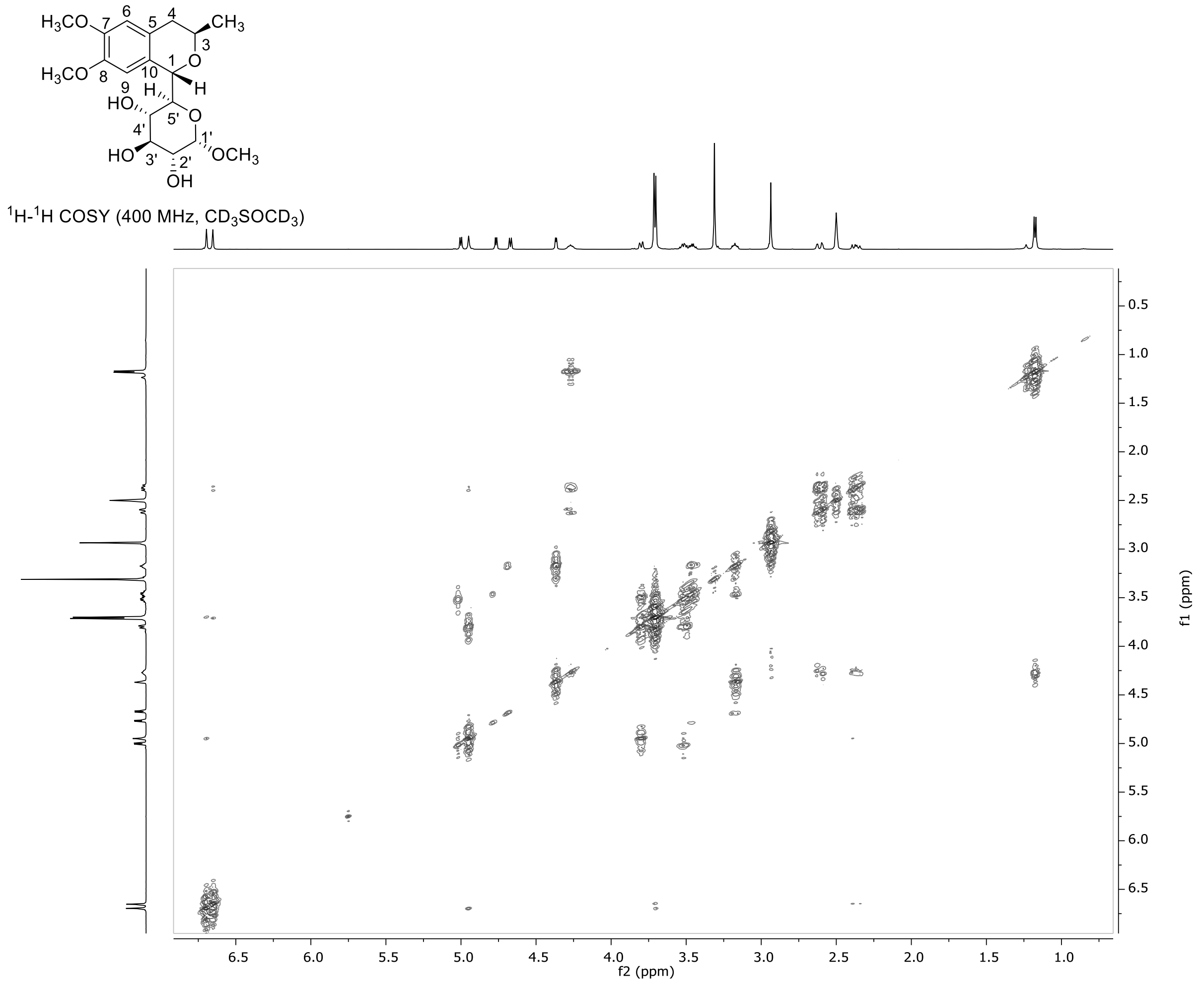

^{1}H-^{1}H COSY (400 MHz, CD_3SOCD_3)

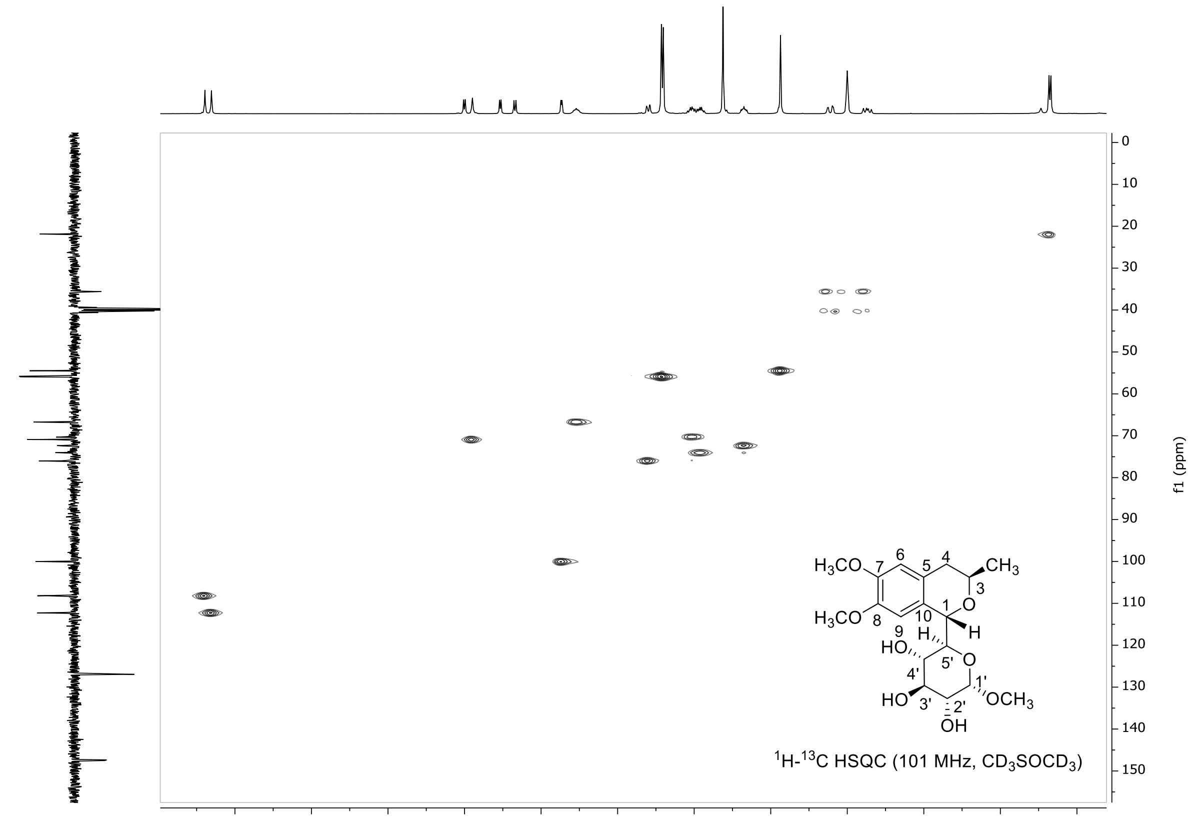

^{1}H-^{13}C HSQC (101 MHz, CD_3SOCD_3)

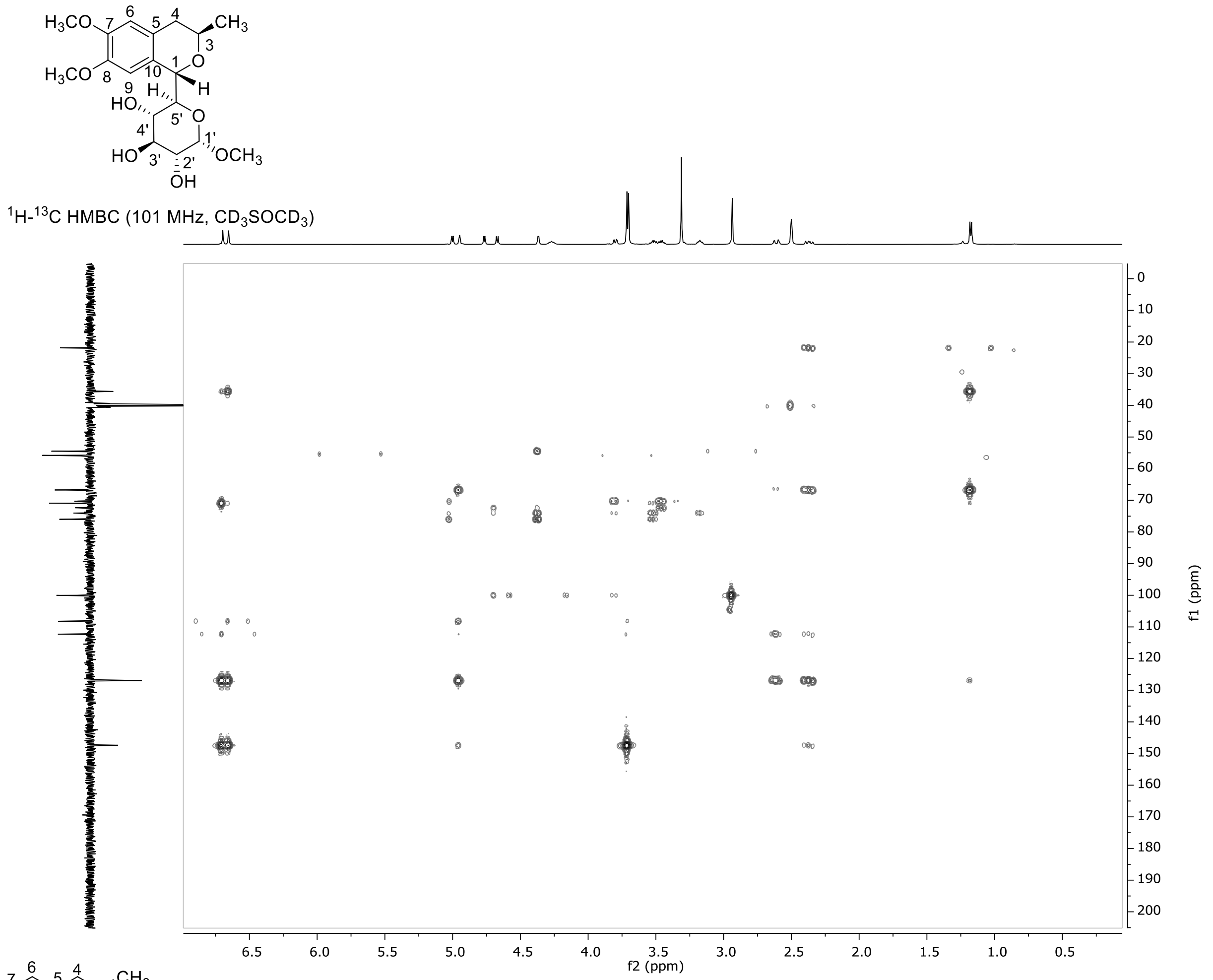

^{1}H-^{13}C HMBC (101 MHz, CD_3SOCD_3)
f2 (ppm)
f1 (ppm)

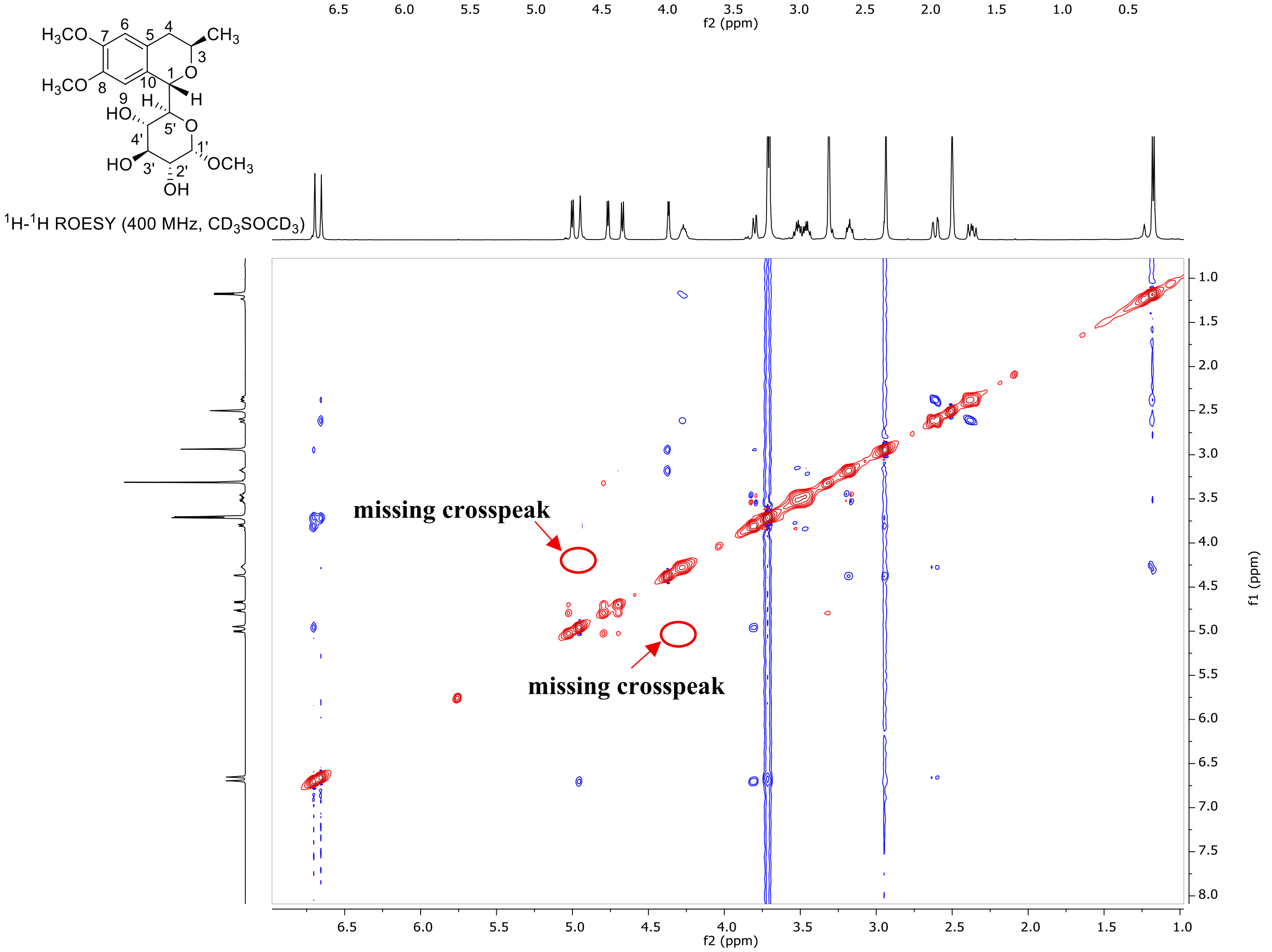

^{1}H-^{1}H ROESY (400 MHz, CD_3SOCD_3)
missing crosspeak
missing crosspeak
f2 (ppm)
f1 (ppm)

Compound 7

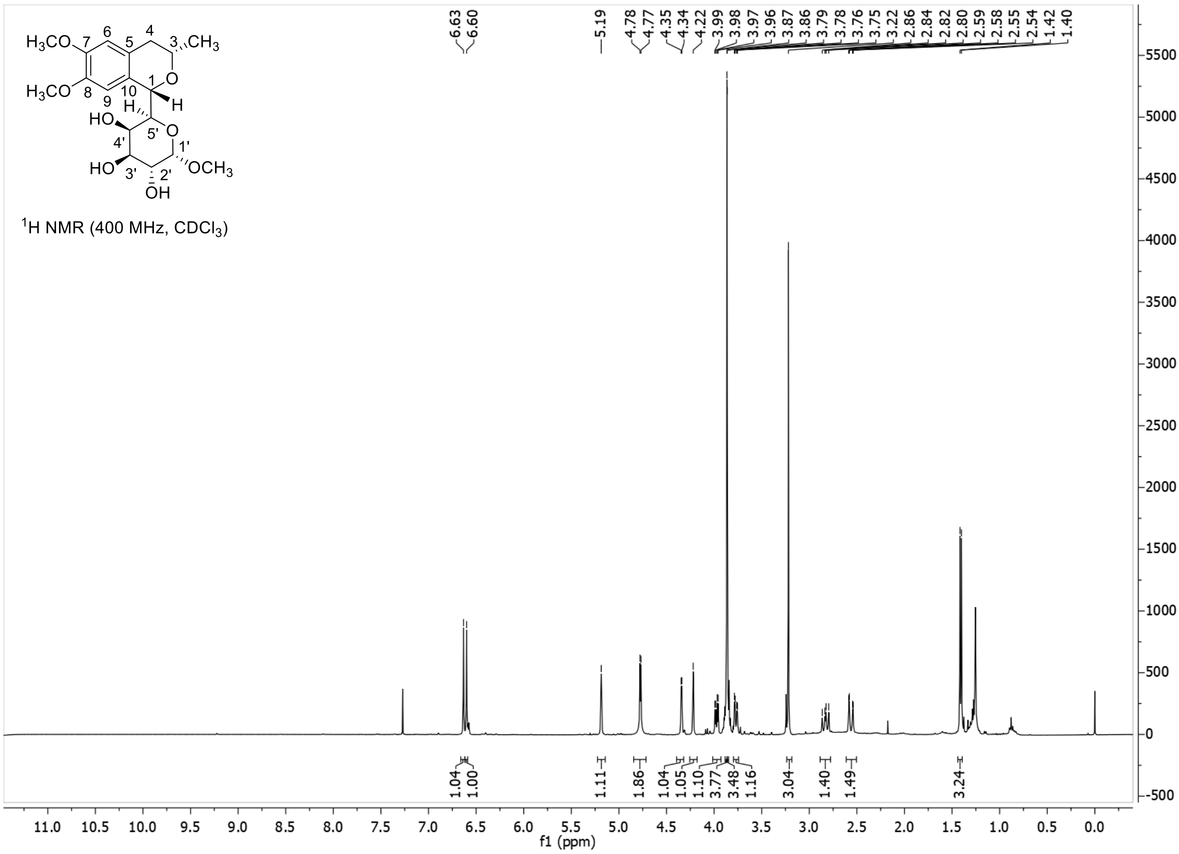

6.63
6.60
5.19
4.78
4.77
4.35
4.34
4.22
3.99
3.98
3.97
3.96
3.87
3.86
3.79
3.78
3.76
3.75
3.22
2.86
2.84
2.82
2.80
2.59
2.58
2.55
2.54
1.42
1.40
1H NMR (400 MHz, CDCl3)
1.04
1.00
1.11
1.86
1.04
1.05
1.10
3.77
3.48
1.16
3.04
1.40
1.49
3.24
f1 (ppm)

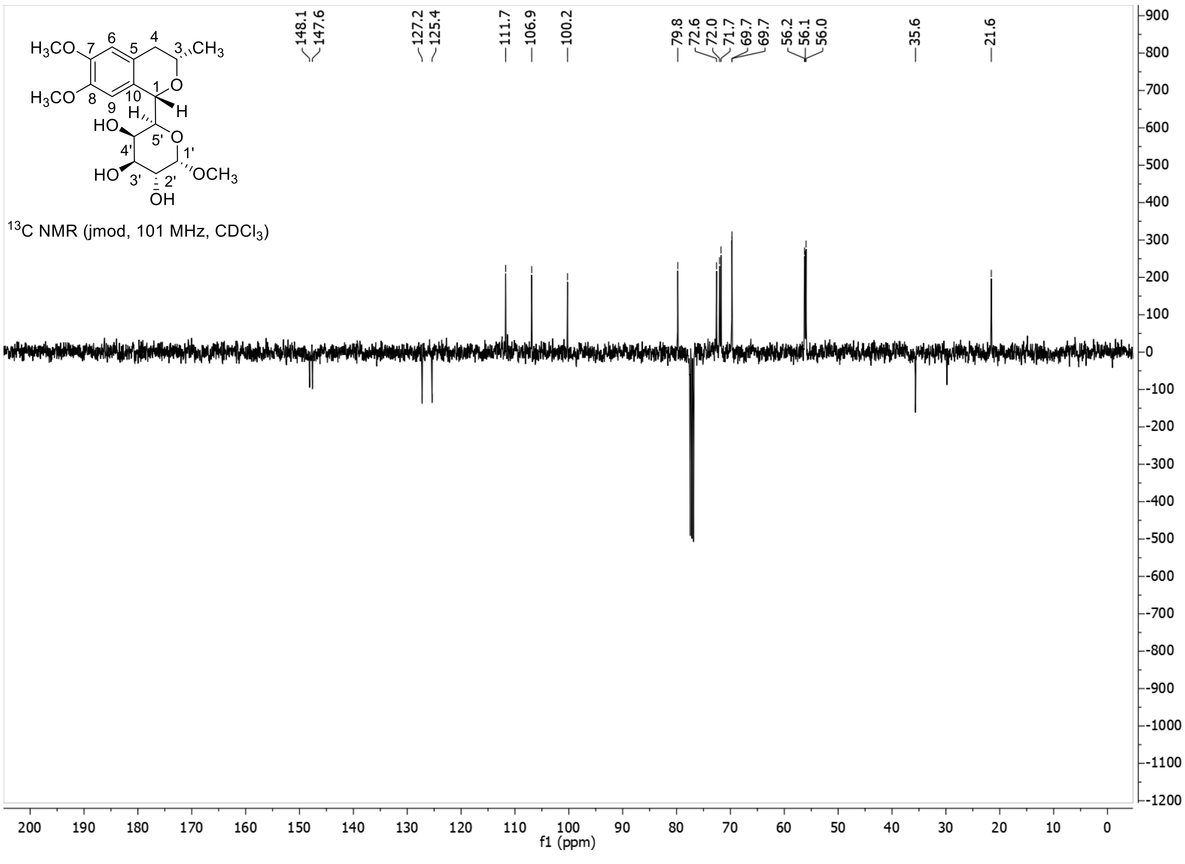

148.1
147.6
127.2
125.4
111.7
106.9
100.2
79.8
72.6
72.0
71.7
69.7
69.7
56.2
56.1
56.0
35.6
21.6
13C NMR (jmod, 101 MHz, CDCl3)
f1 (ppm)

Compound 8

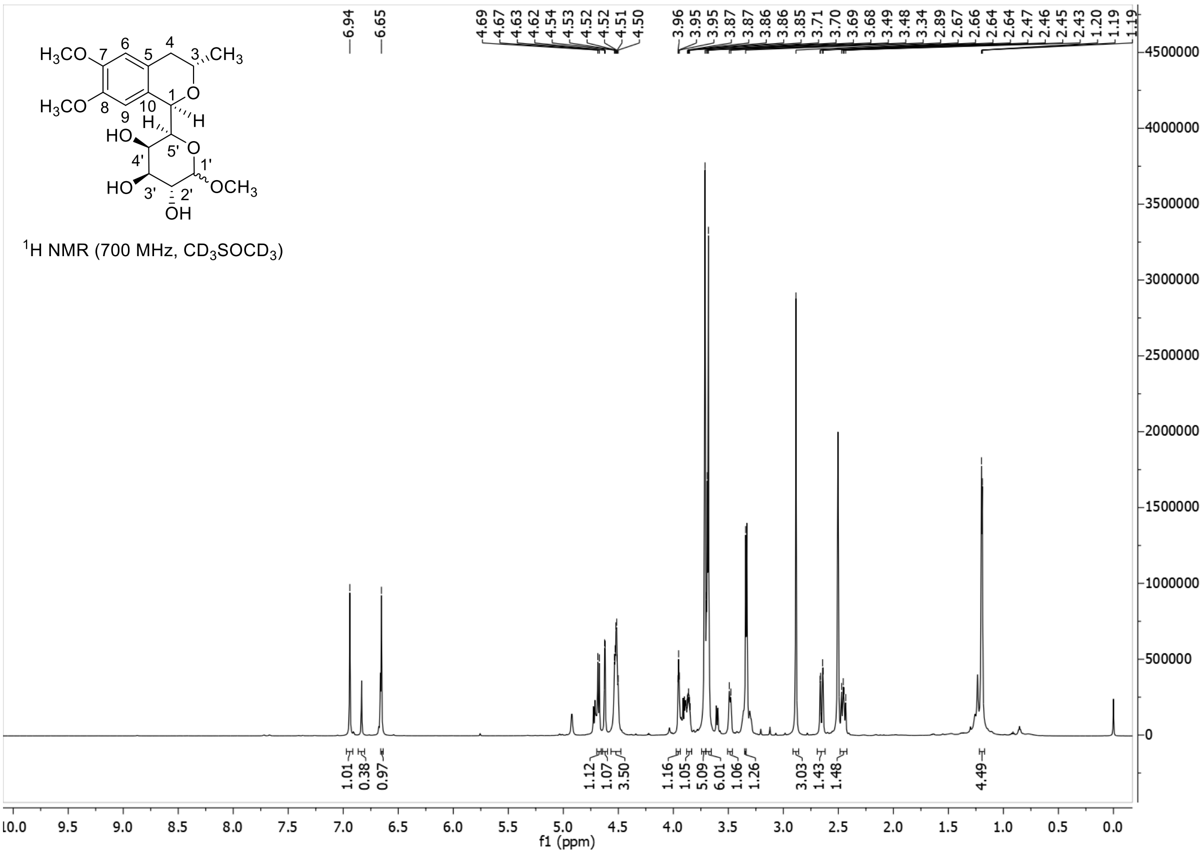

H3CO
H3CO
HO
HO
OH
OCH3
CH3
1H NMR (700 MHz, CD3SOCD3)
6.94
6.65
4.69
4.67
4.63
4.62
4.54
4.53
4.52
4.52
4.51
4.50
3.96
3.95
3.95
3.87
3.87
3.86
3.86
3.85
3.71
3.70
3.69
3.68
3.49
3.48
3.34
2.89
2.67
2.66
2.64
2.64
2.47
2.46
2.45
2.43
1.20
1.19
1.19
1.01
0.38
0.97
1.12
1.07
3.50
1.16
1.05
5.09
6.01
1.06
1.26
3.03
1.43
1.48
4.49
f1 (ppm)

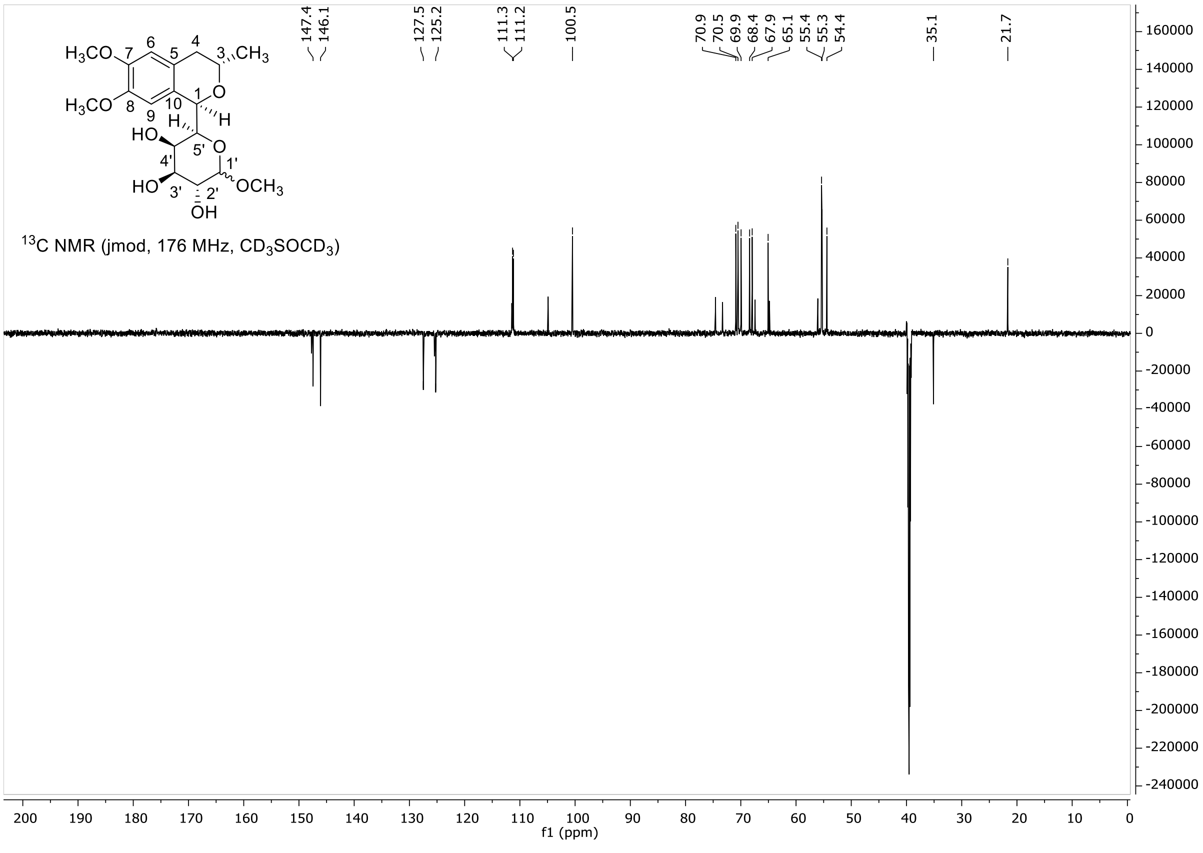

H3CO
H3CO
HO
HO
OH
OCH3
CH3
13C NMR (jmod, 176 MHz, CD3SOCD3)
147.4
146.1
127.5
125.2
111.3
111.2
100.5
70.9
70.5
69.9
68.4
67.9
65.1
55.4
55.3
54.4
35.1
21.7
f1 (ppm)

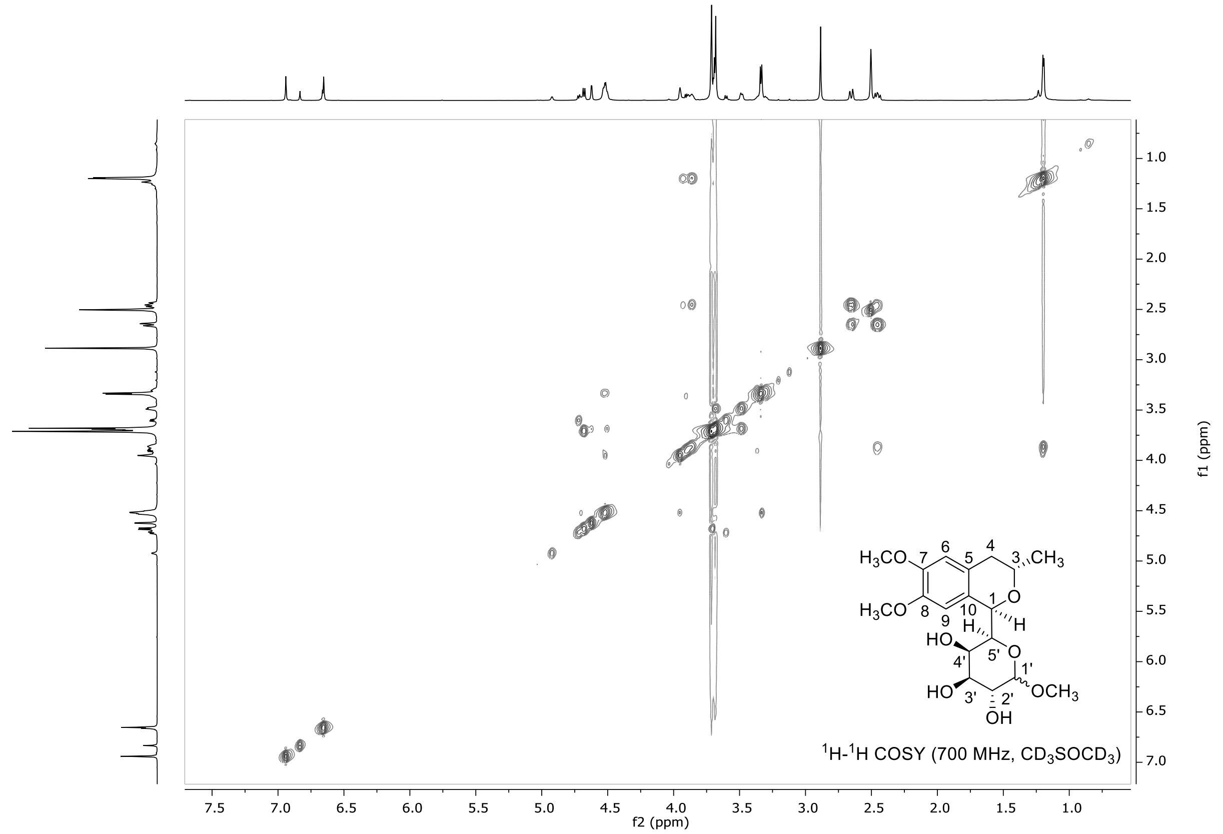
H3CO 7 6 5 4 3 CH3
O
H3CO 8 9 10 1 H
H
HO 4' 5' O
HO 3' 2' 1' OCH3
OH
1H-1H COSY (700 MHz, CD3SOCD3)
f2 (ppm)
f1 (ppm)

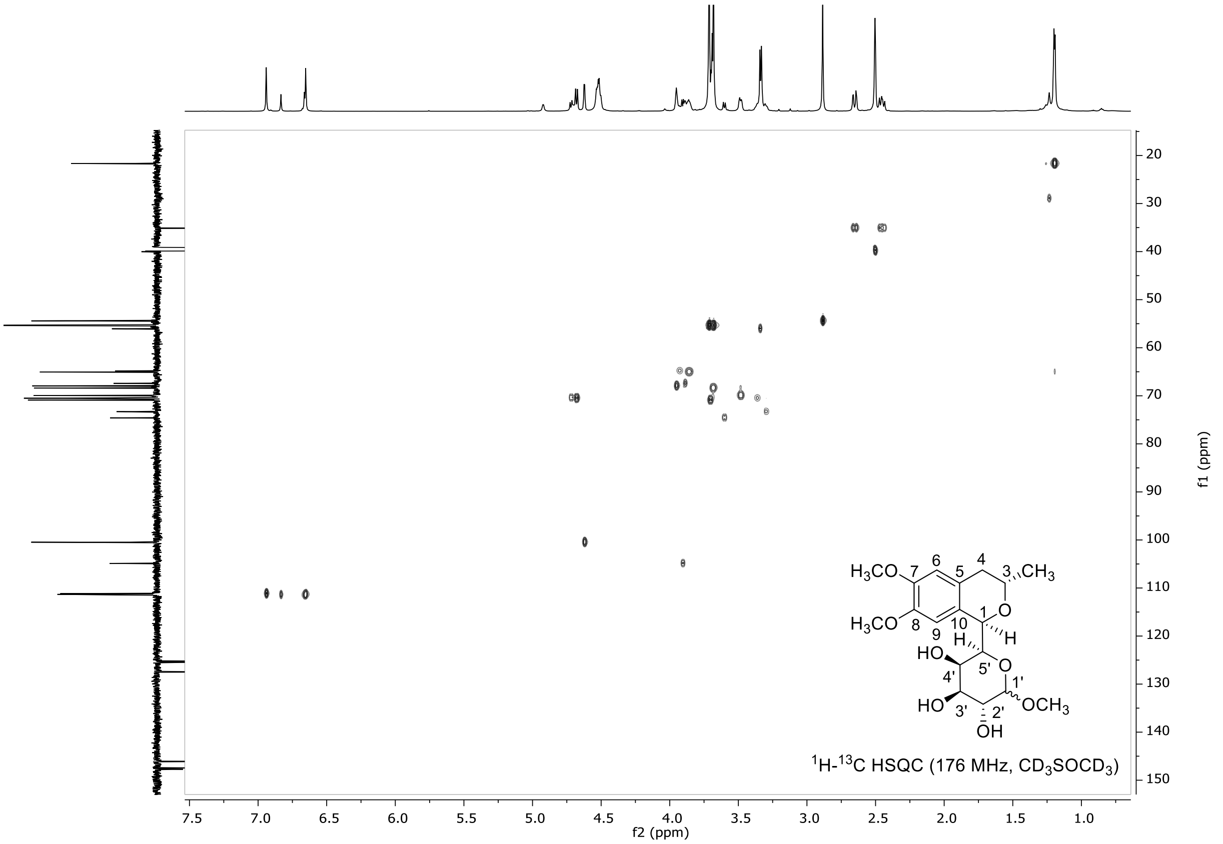
H3CO 7 6 5 4 3 CH3
O
H3CO 8 9 10 1 H
H
HO 4' 5' O
HO 3' 2' 1' OCH3
OH
1H-13C HSQC (176 MHz, CD3SOCD3)
f2 (ppm)
f1 (ppm)

Compound 9α

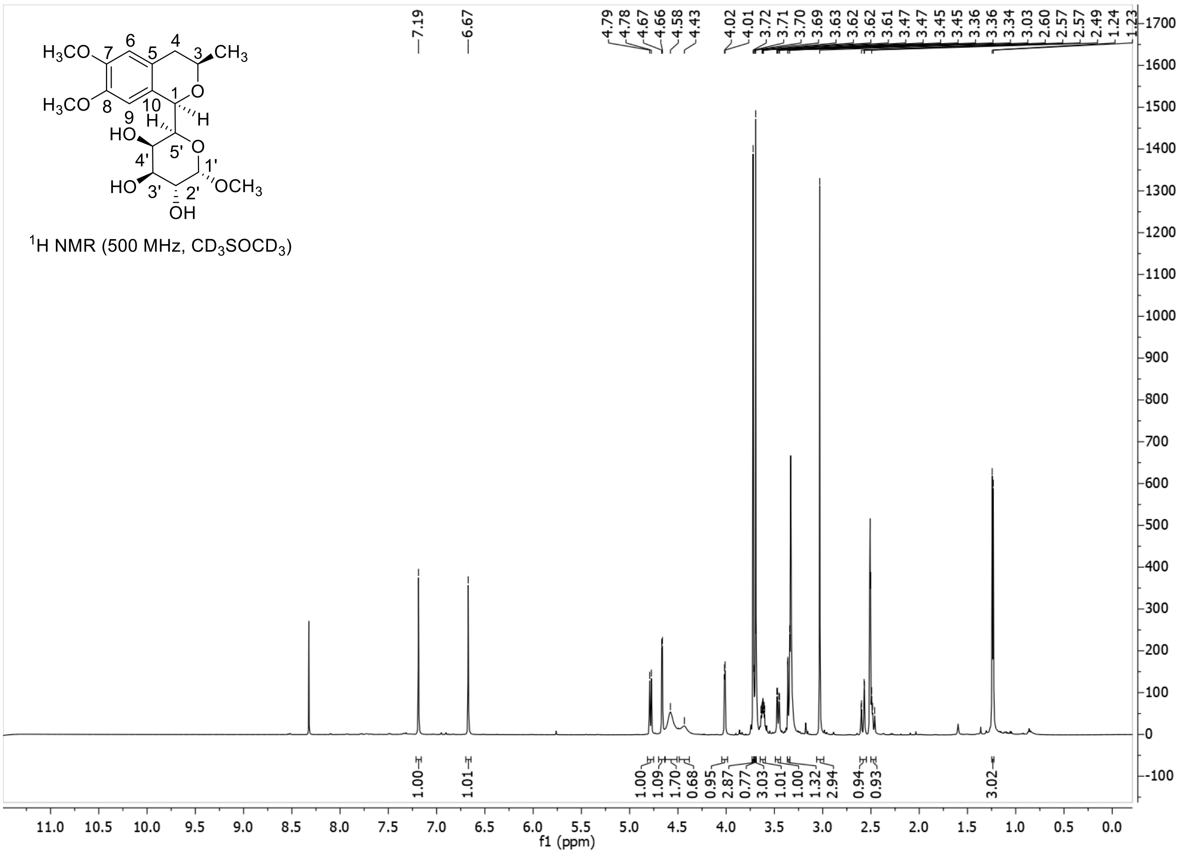

^{1}H NMR (500 MHz, CD_3SOCD_3)
f1 (ppm)

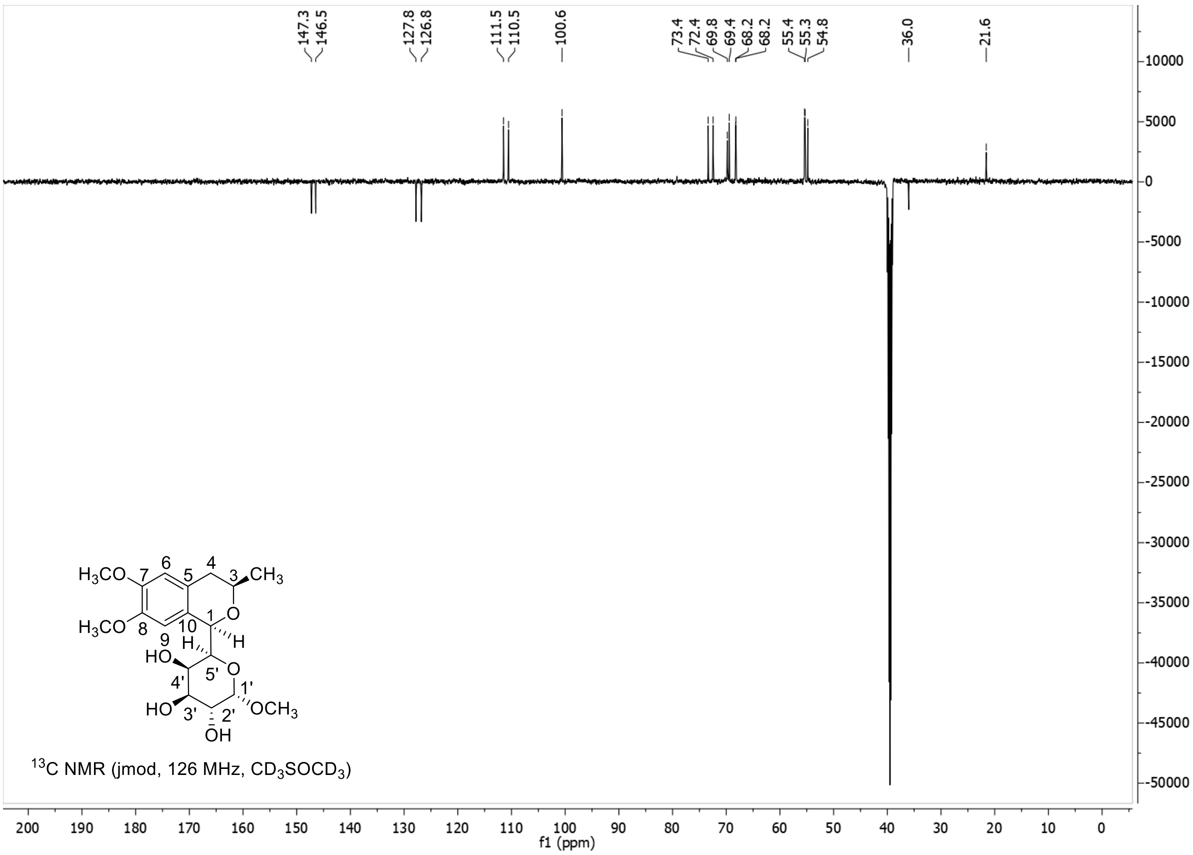

^{13}C NMR (jmod, 126 MHz, CD_3SOCD_3)
f1 (ppm)

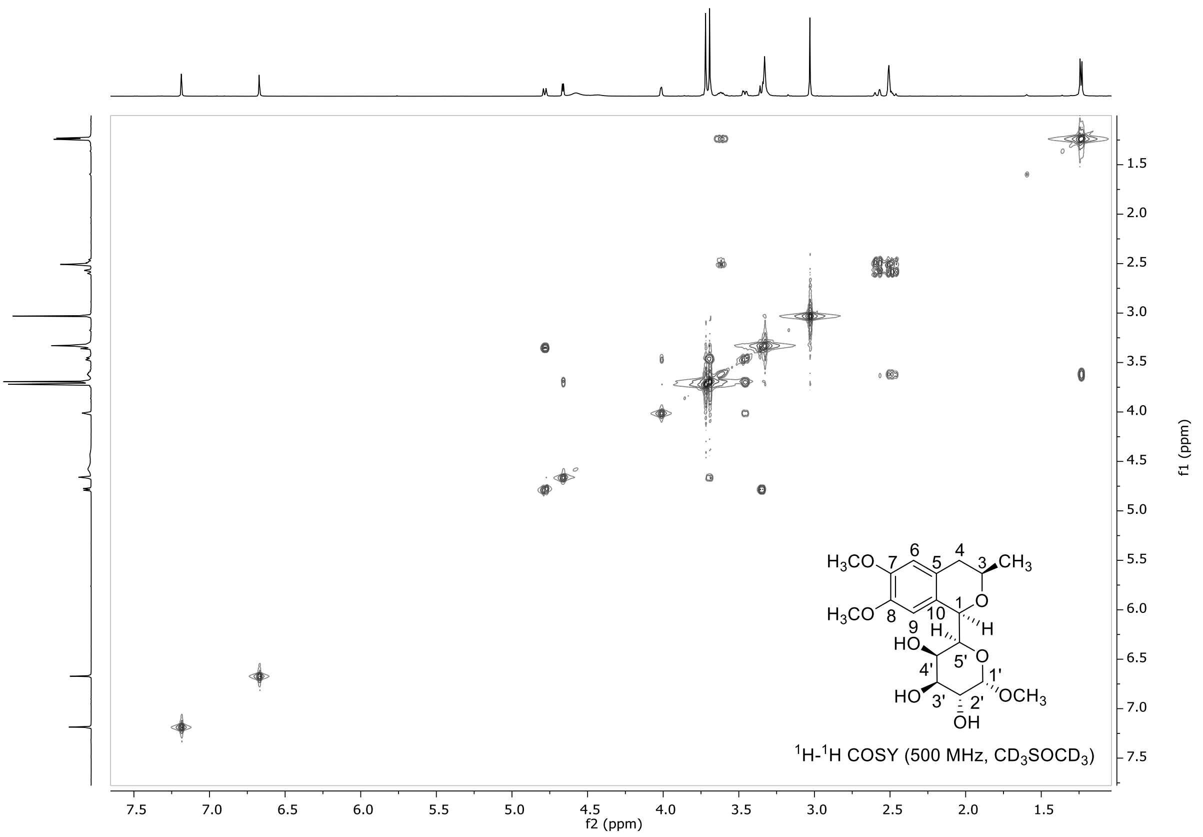
f1 (ppm)
f2 (ppm)
H3CO 7 6 5 4 3 CH3
H3CO 8 9 10 1 O H
H HO 5' O 4' 1' HO 3' 2' OCH3 OH
1H-1H COSY (500 MHz, CD3SOCD3)

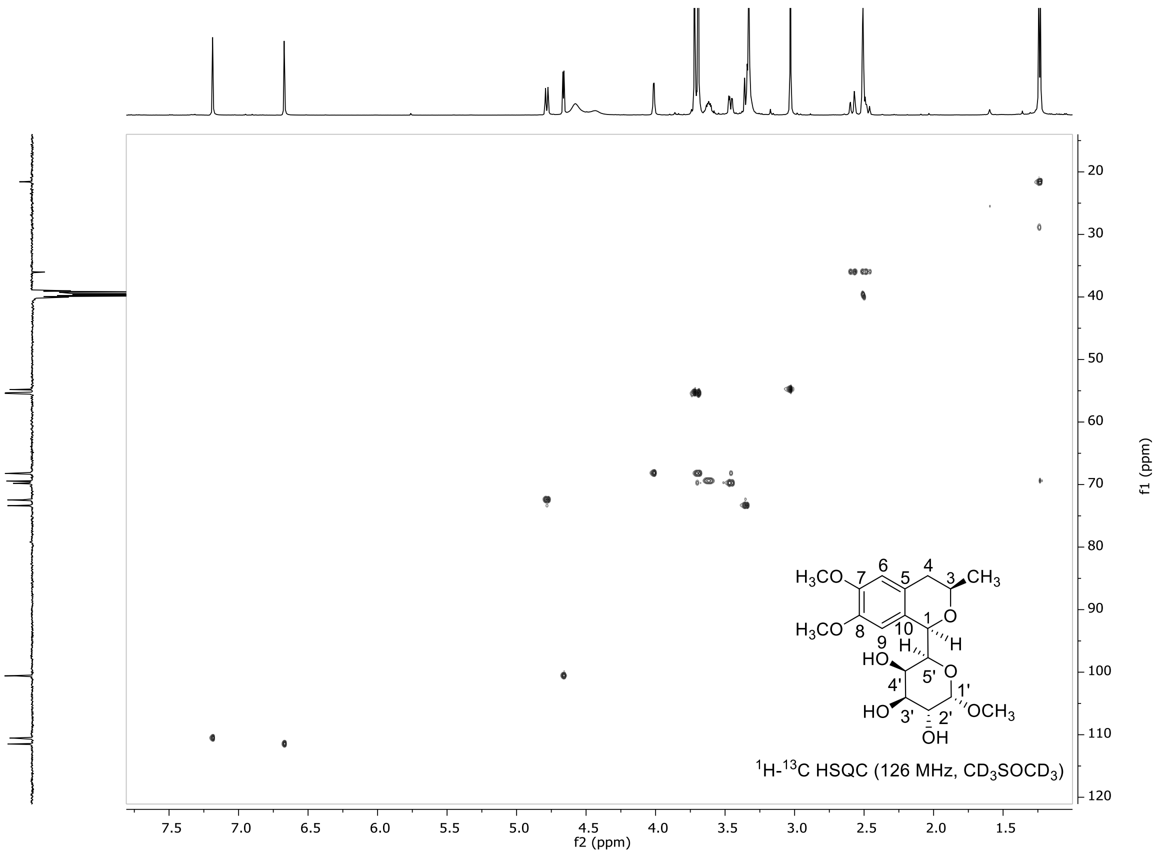
f1 (ppm)
f2 (ppm)
H3CO 7 6 5 4 3 CH3
H3CO 8 9 10 1 O H
H HO 5' O 4' 1' HO 3' 2' OCH3 OH
1H-13C HSQC (126 MHz, CD3SOCD3)

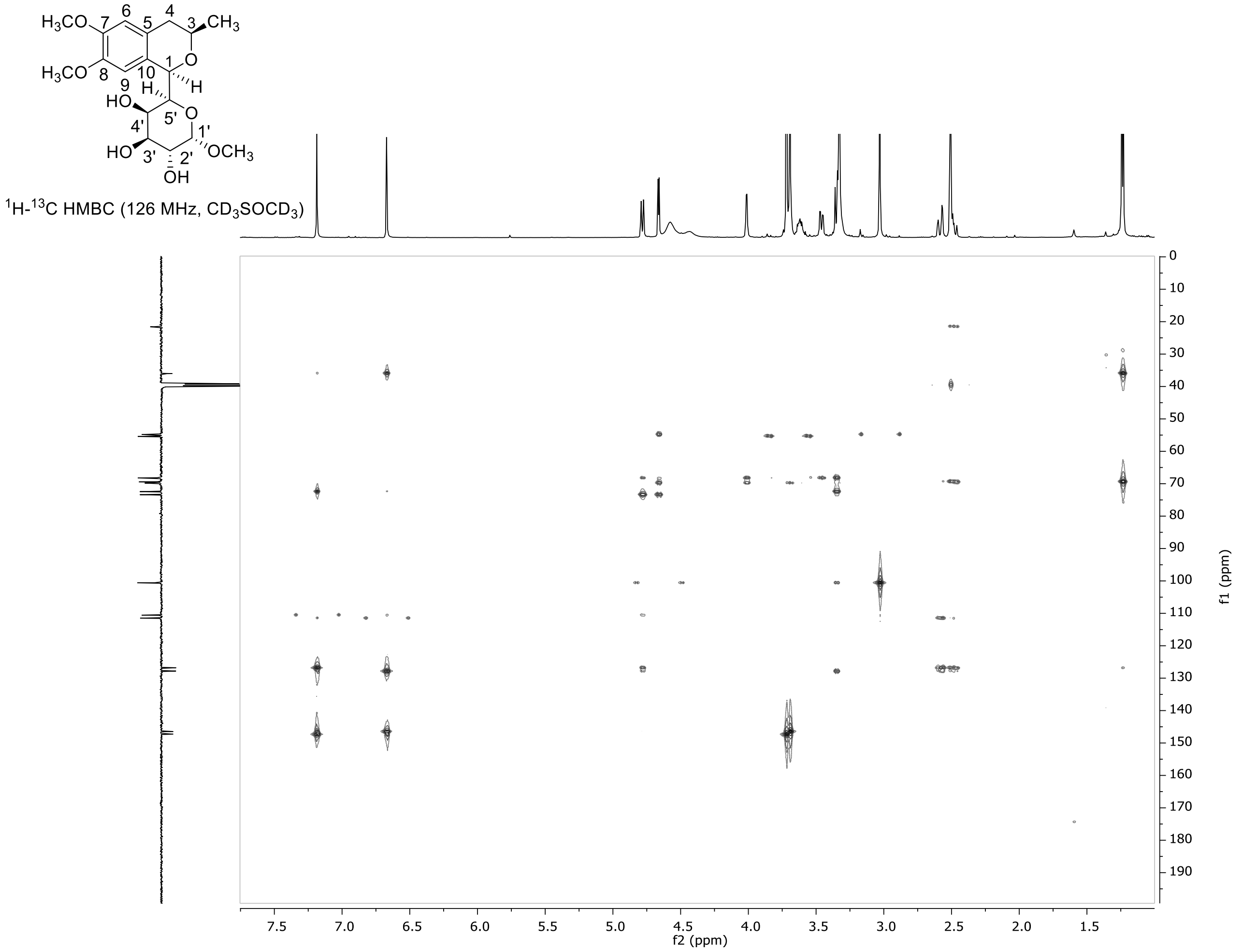
H3CO
H3CO
HO
HO
OH
OCH3
CH3
1H-13C HMBC (126 MHz, CD3SOCD3)
f2 (ppm)
f1 (ppm)

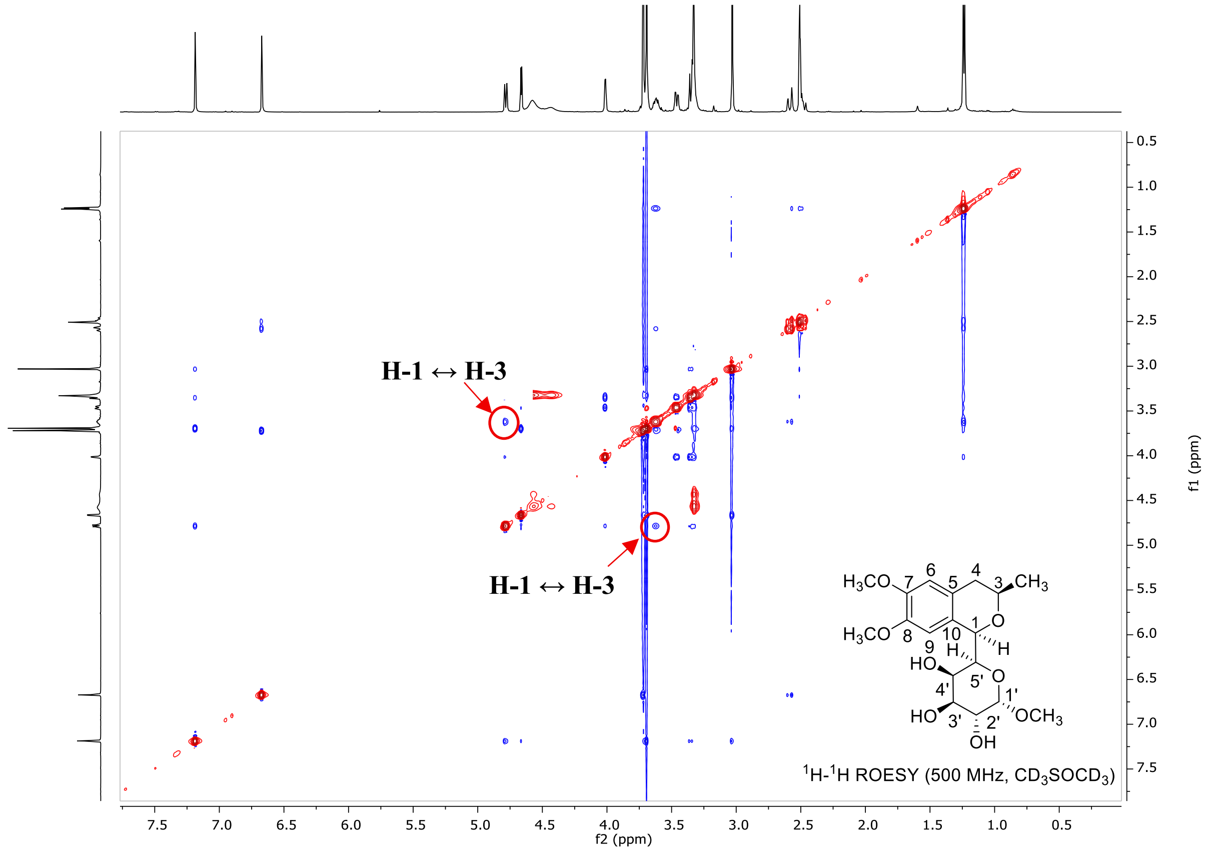
H-1 ↔ H-3
H-1 ↔ H-3
H3CO
H3CO
HO
HO
OH
OCH3
CH3
1H-1H ROESY (500 MHz, CD3SOCD3)
f2 (ppm)
f1 (ppm)

Compound 10α,β

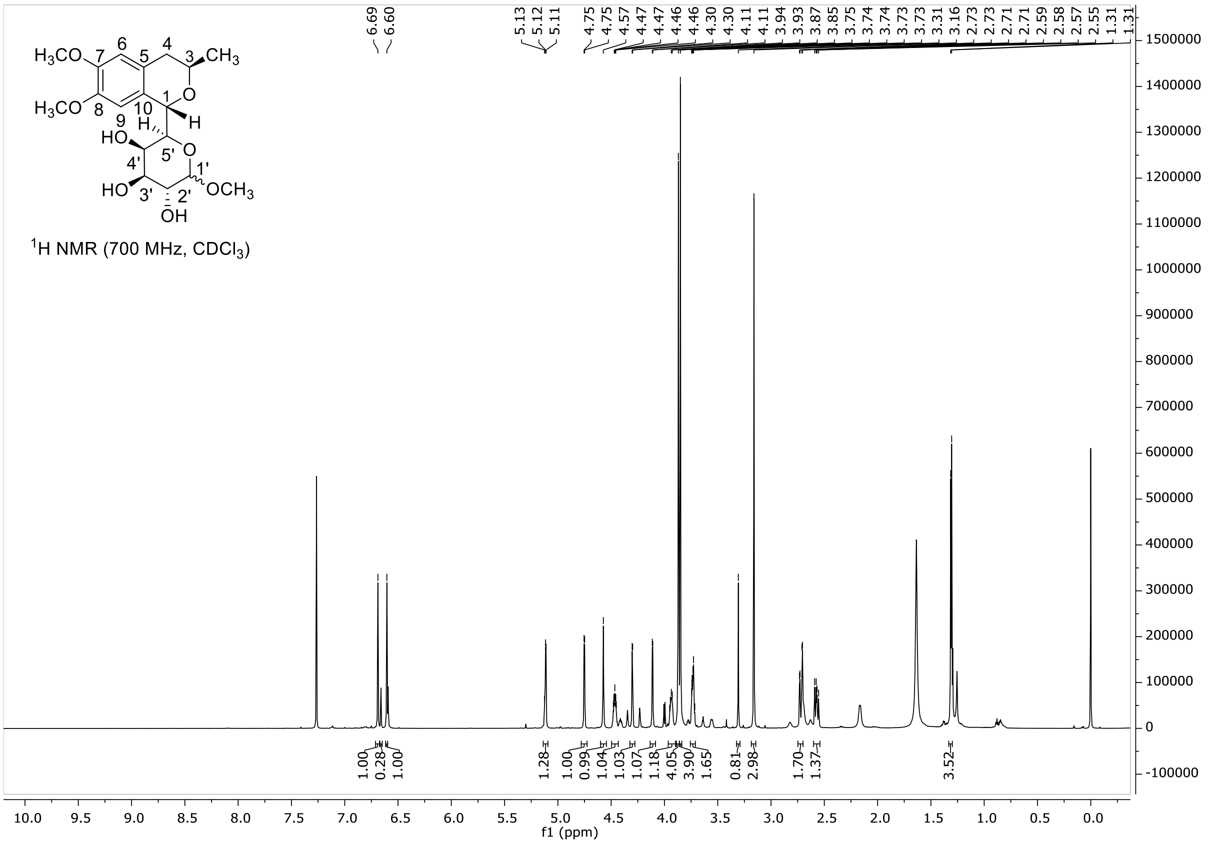

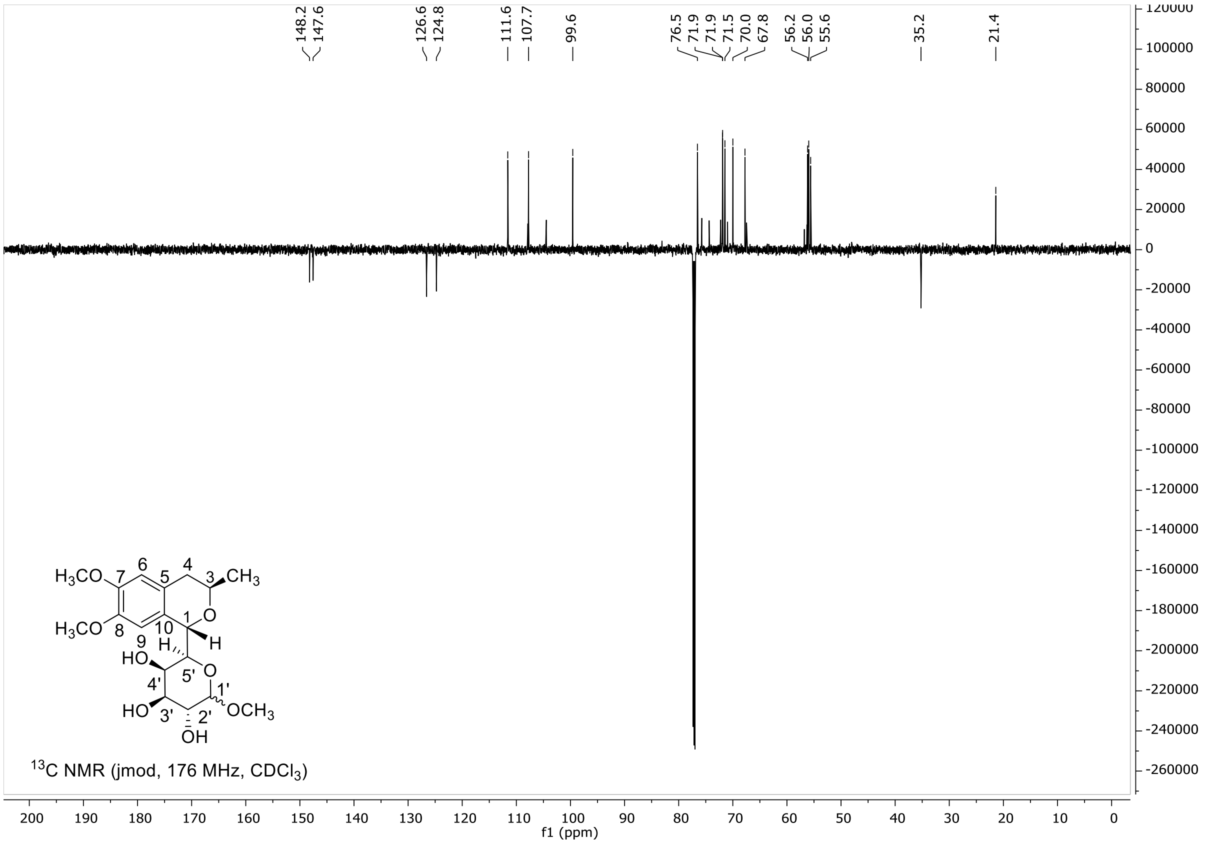

Compound 11α

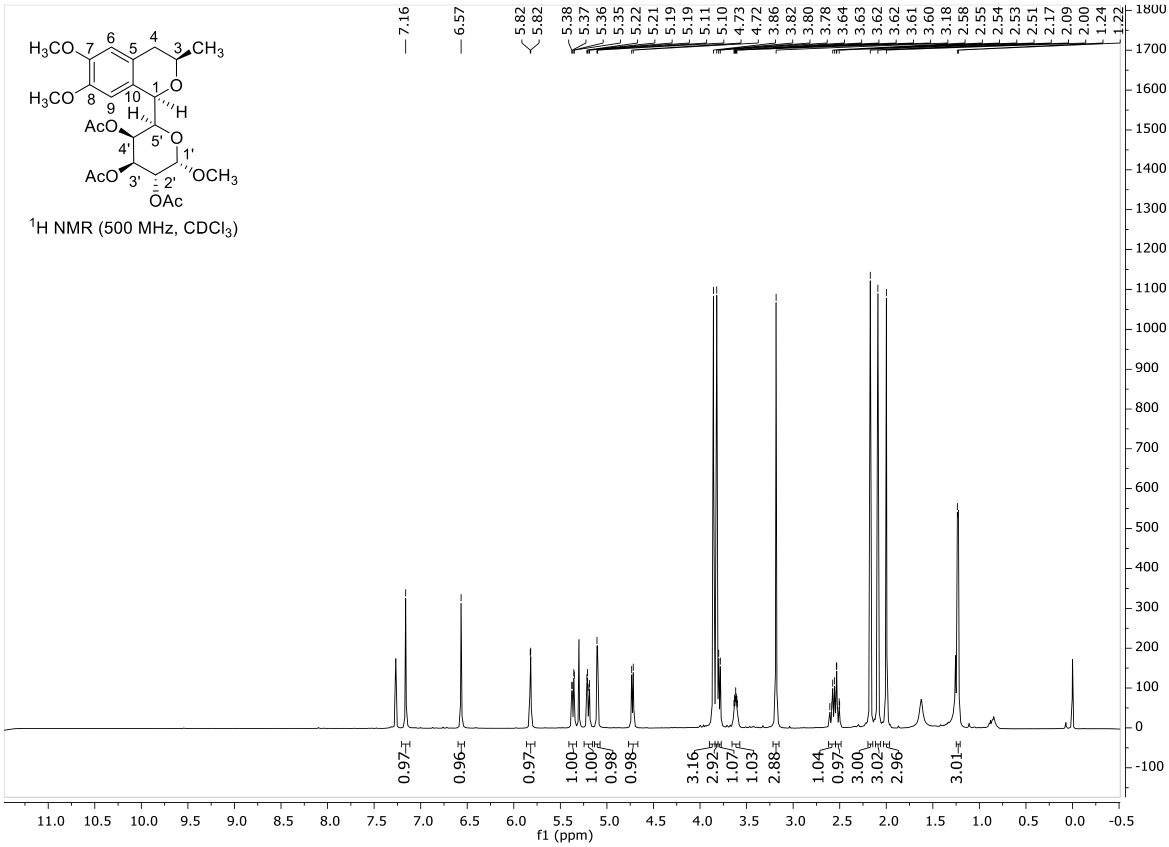

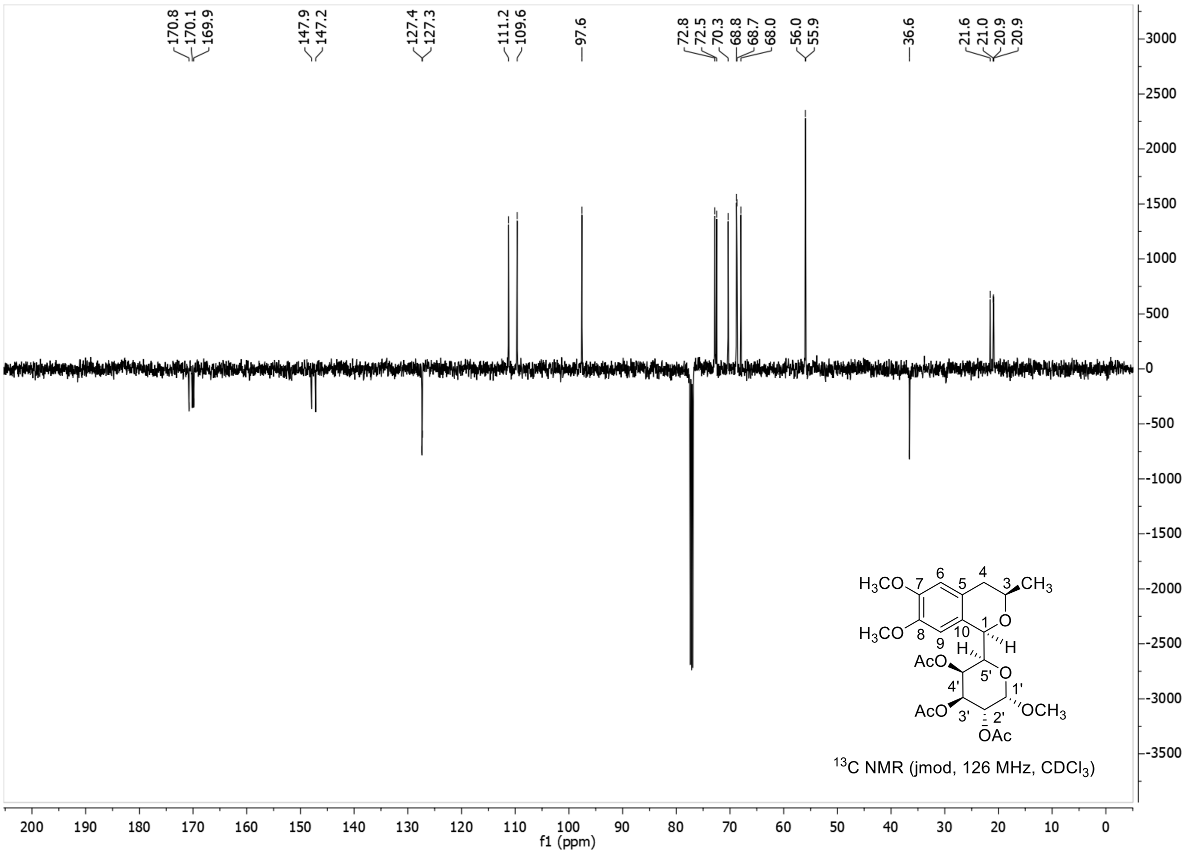

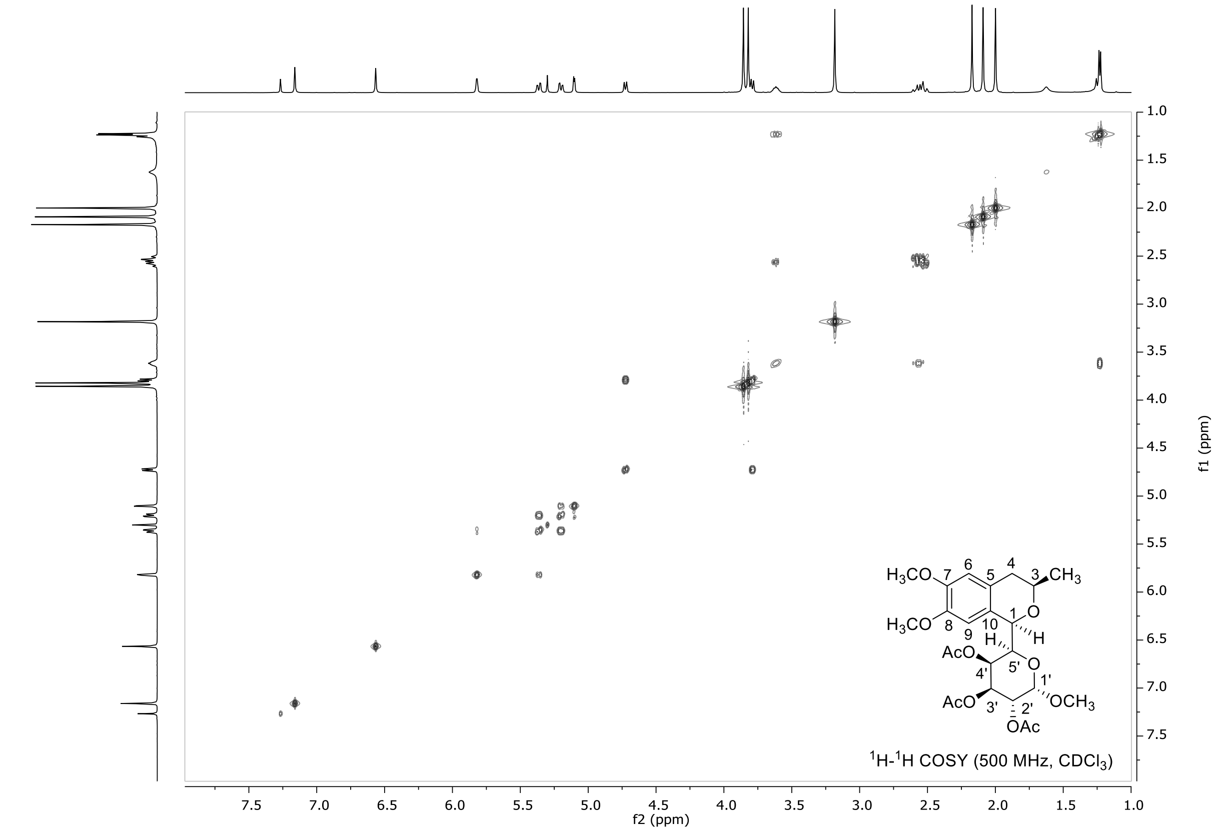

H3CO 7 6 5 4 3 CH3
H3CO 8 9 10 1 O
H H
AcO 4' 5' O 1'
AcO 3' 2' OCH3
OAc
1H-1H COSY (500 MHz, CDCl3)
f1 (ppm)
f2 (ppm)

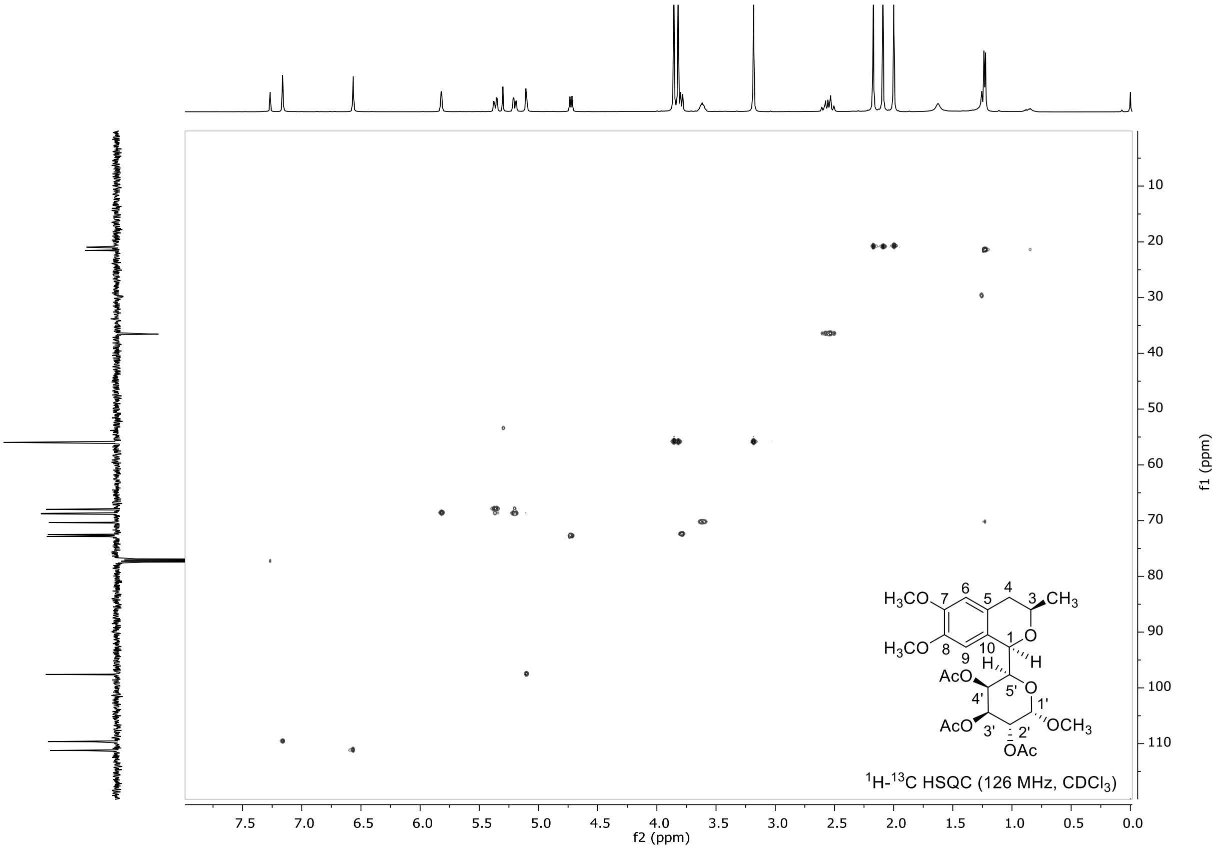

H3CO 7 6 5 4 3 CH3
H3CO 8 9 10 1 O
H H
AcO 4' 5' O 1'
AcO 3' 2' OCH3
OAc
1H-13C HSQC (126 MHz, CDCl3)
f1 (ppm)
f2 (ppm)

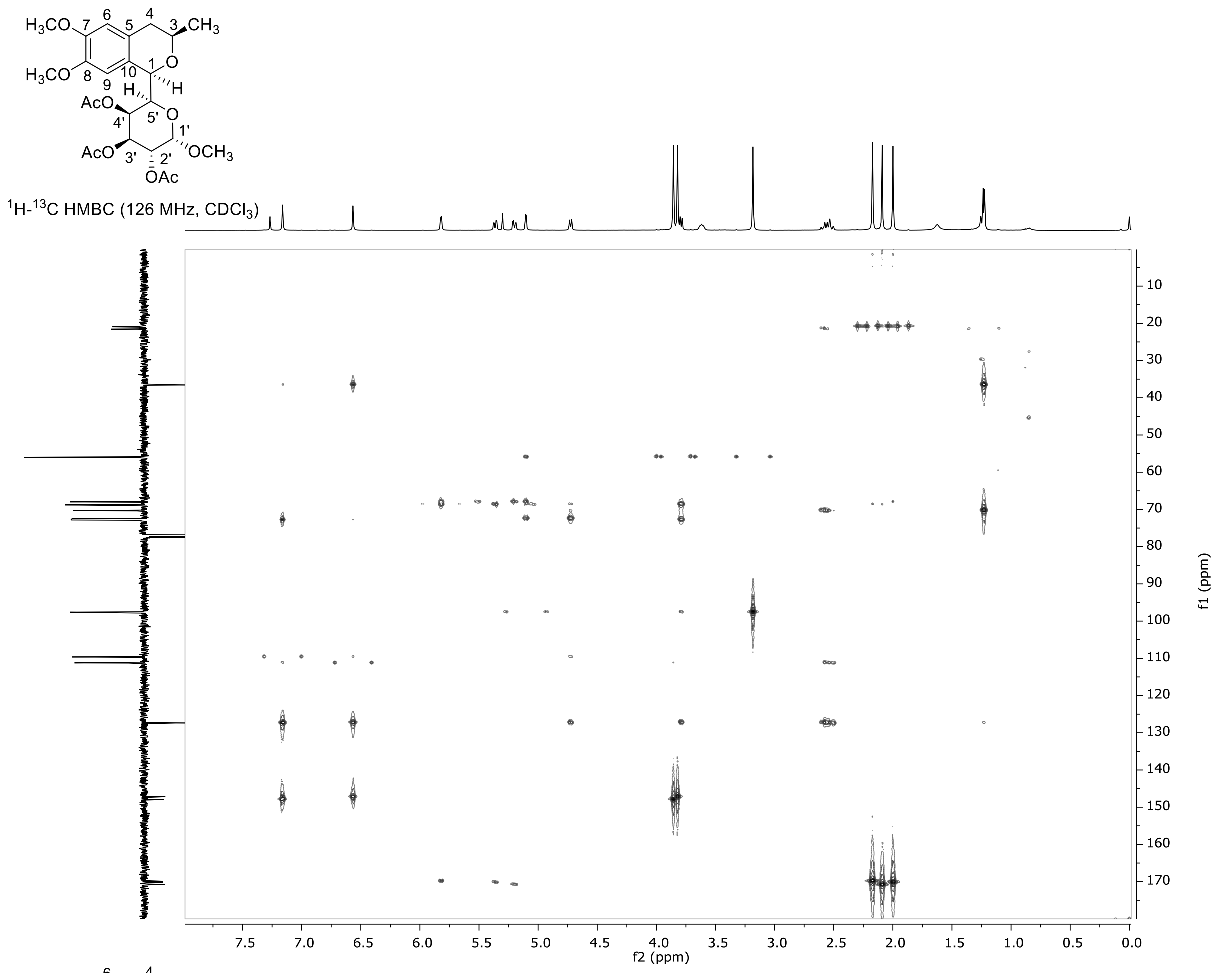
H3CO
H3CO
AcO
AcO
OAc
OCH3
CH3
1H-13C HMBC (126 MHz, CDCl3)
f2 (ppm)
f1 (ppm)

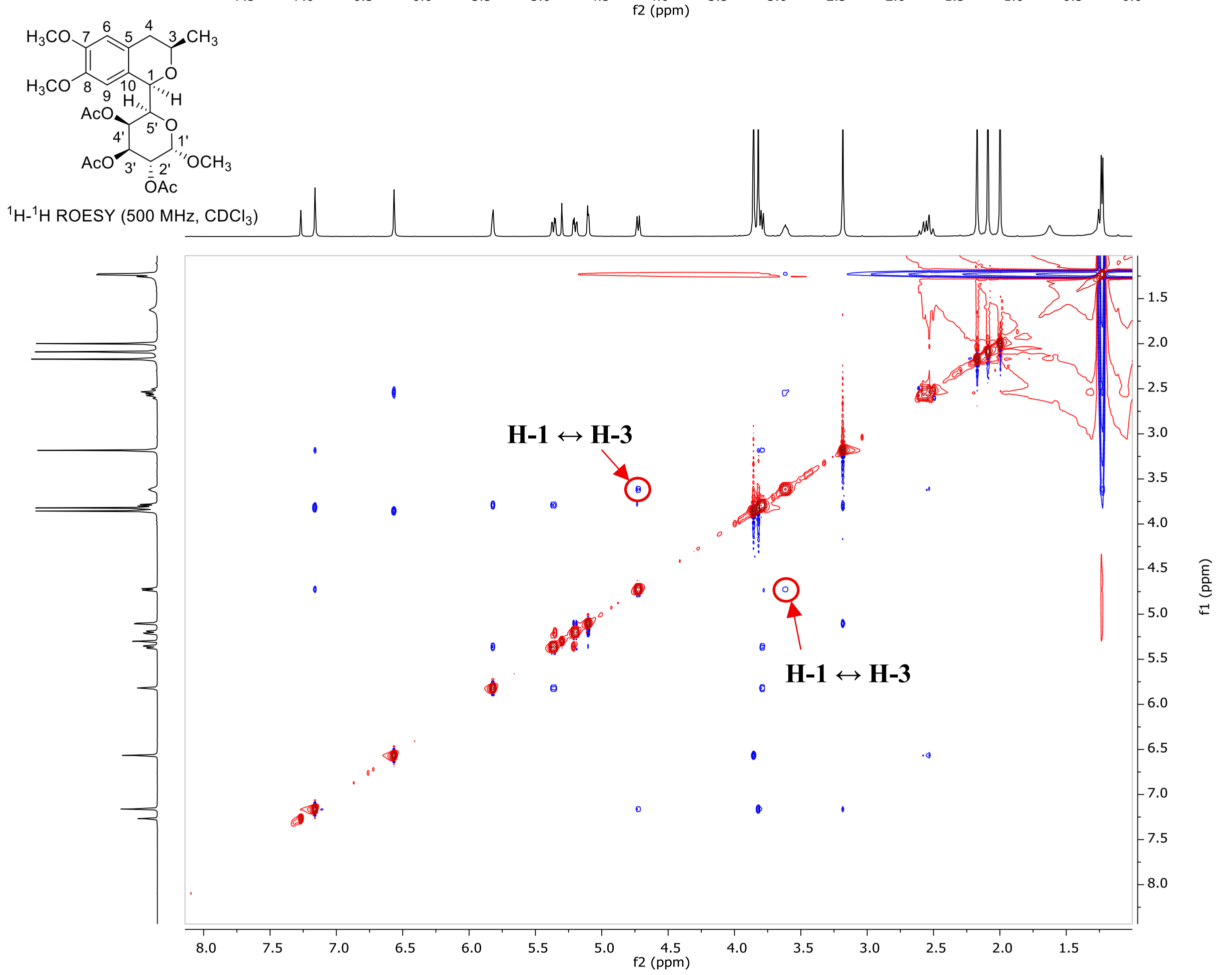
H3CO
H3CO
AcO
AcO
OAc
OCH3
CH3
1H-1H ROESY (500 MHz, CDCl3)
H-1 ↔ H-3
H-1 ↔ H-3
f2 (ppm)
f1 (ppm)

Compound 11β

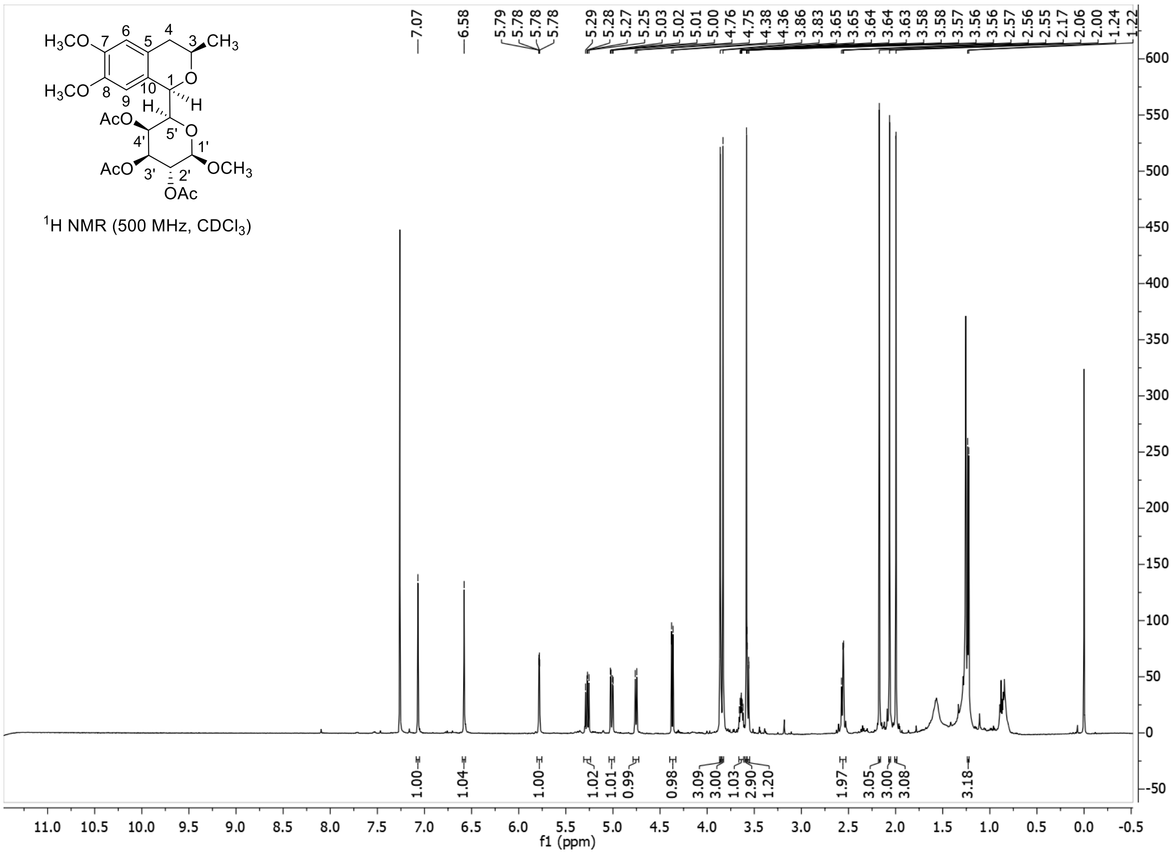

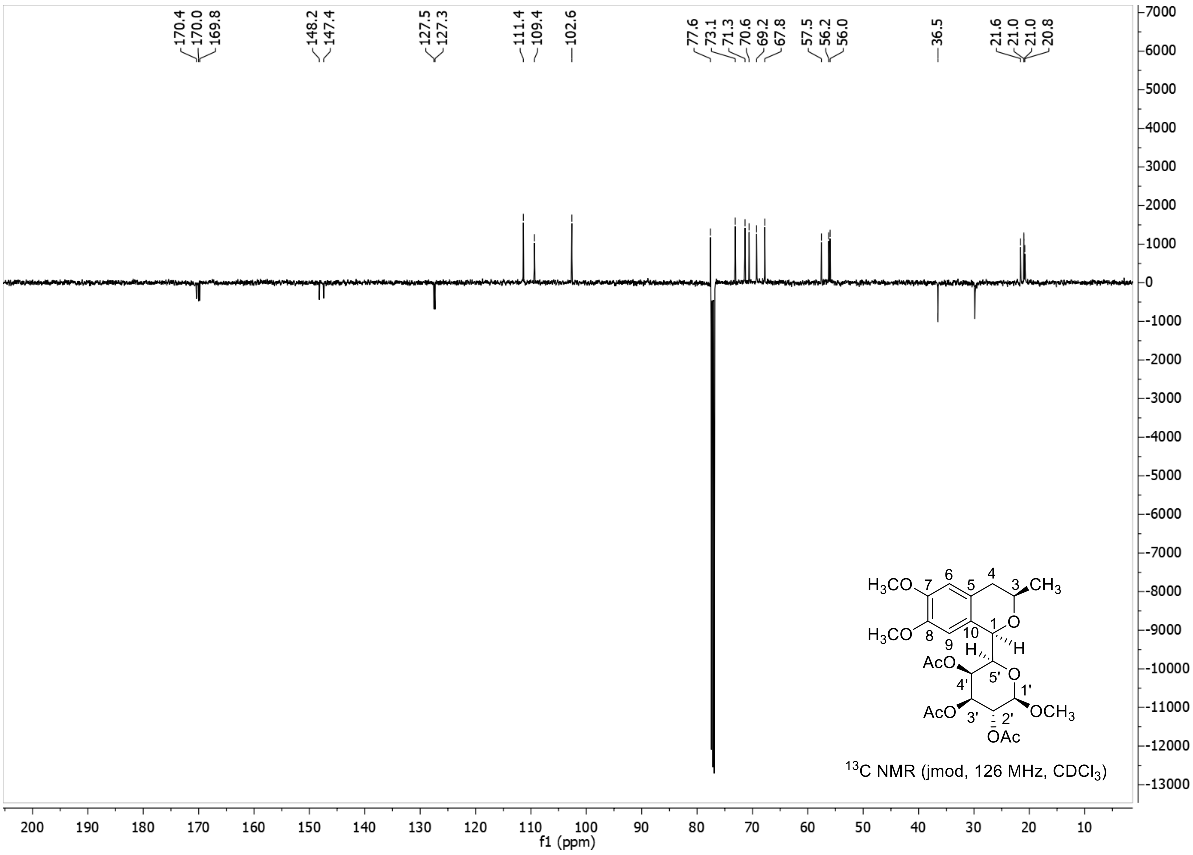

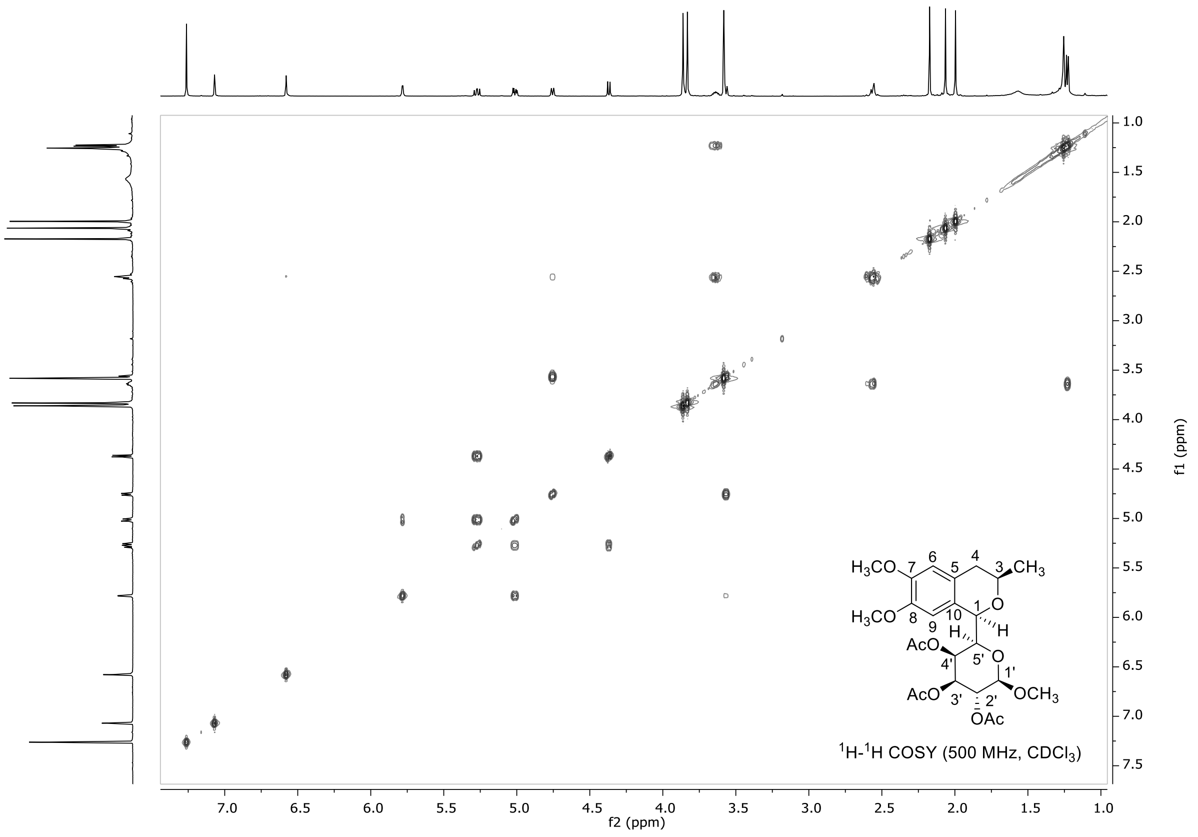
H3CO 7 6 5 4 3 CH3
H3CO 8 9 10 1 O
H H
AcO 4' 5' O 1'
AcO 3' 2' OCH3
OAc
1H-1H COSY (500 MHz, CDCl3)
f2 (ppm)
f1 (ppm)

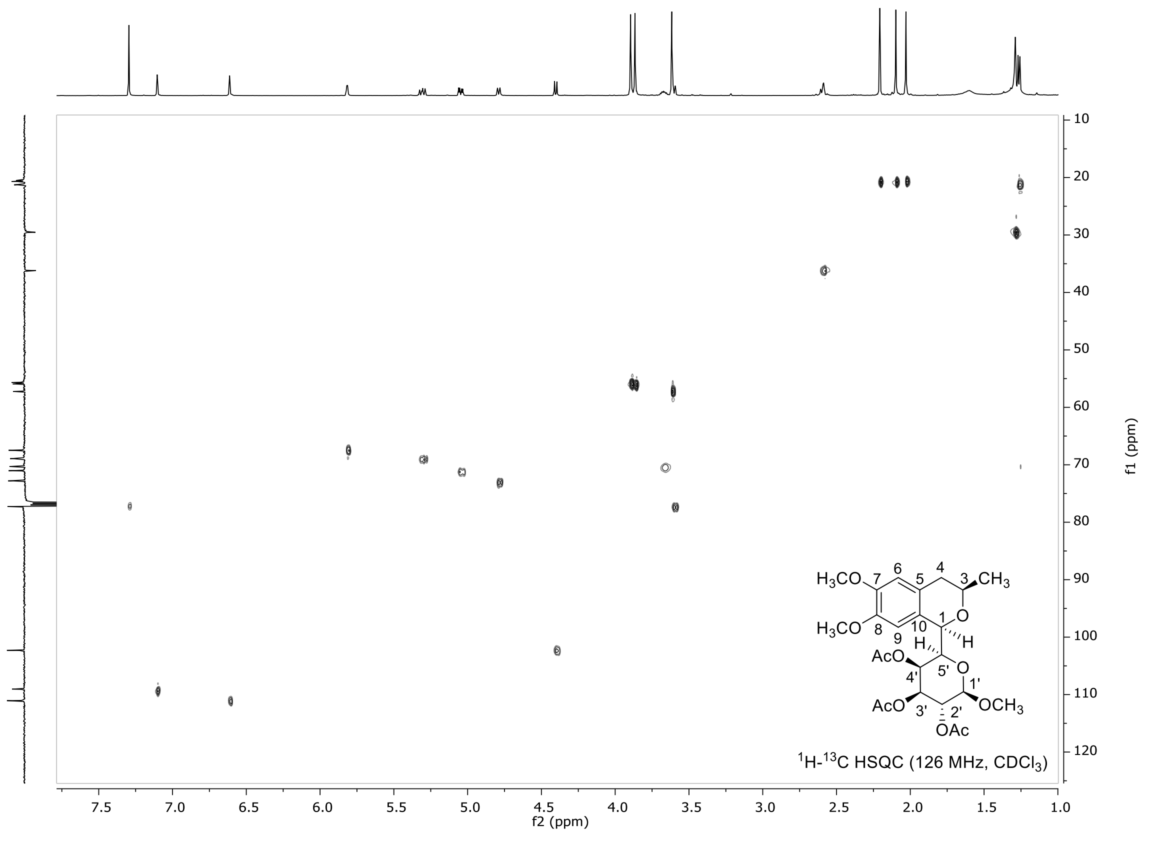
H3CO 7 6 5 4 3 CH3
H3CO 8 9 10 1 O
H H
AcO 4' 5' O 1'
AcO 3' 2' OCH3
OAc
1H-13C HSQC (126 MHz, CDCl3)
f2 (ppm)
f1 (ppm)

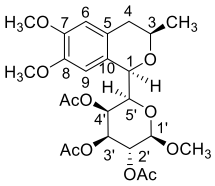

^{1}H-^{13}C HMBC (126 MHz, $CDCl_3$)

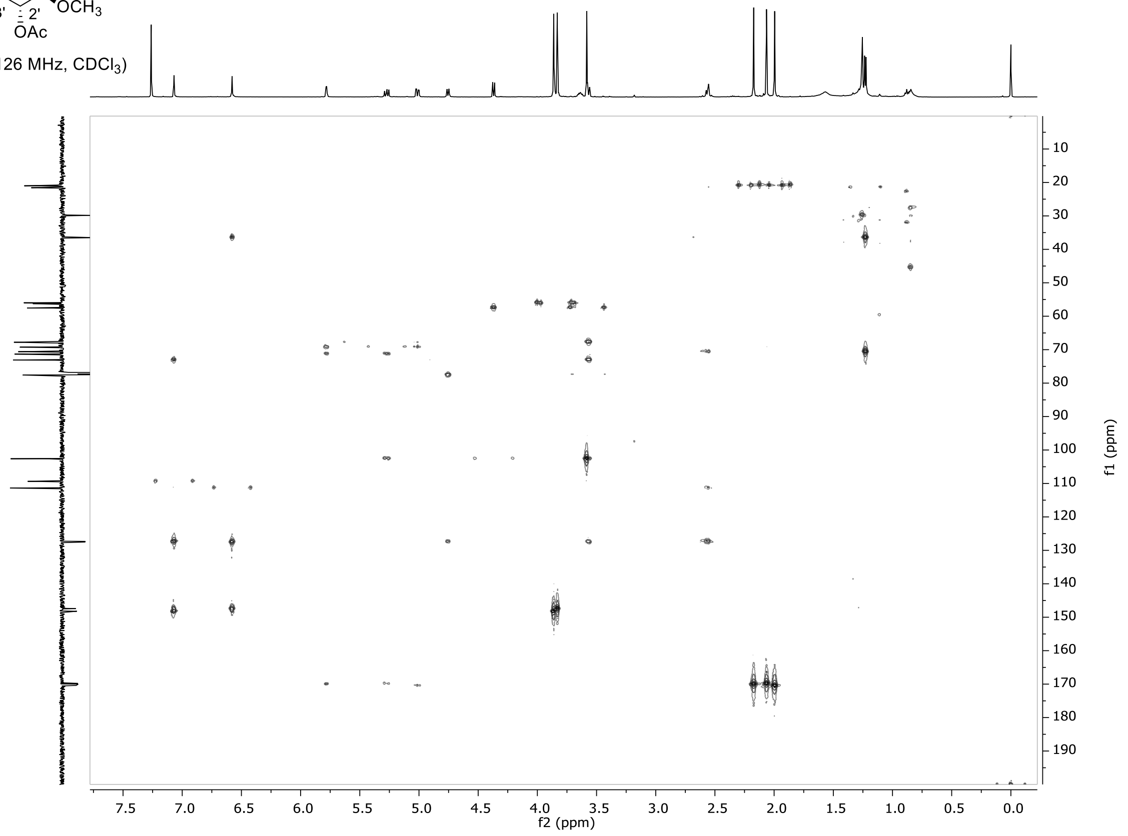

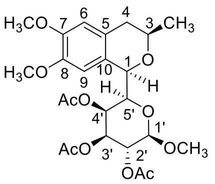

^{1}H-^{1}H ROESY (500 MHz, $CDCl_3$)

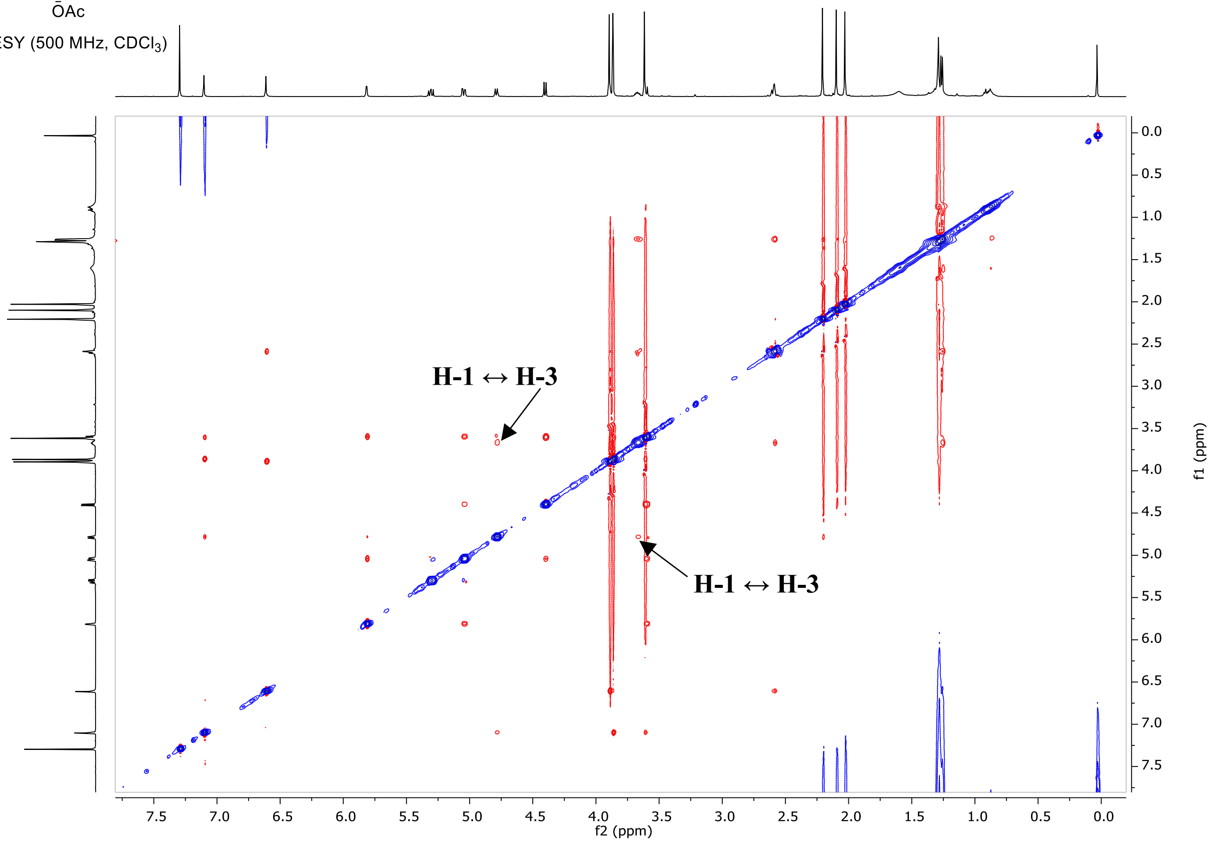

Compound 16

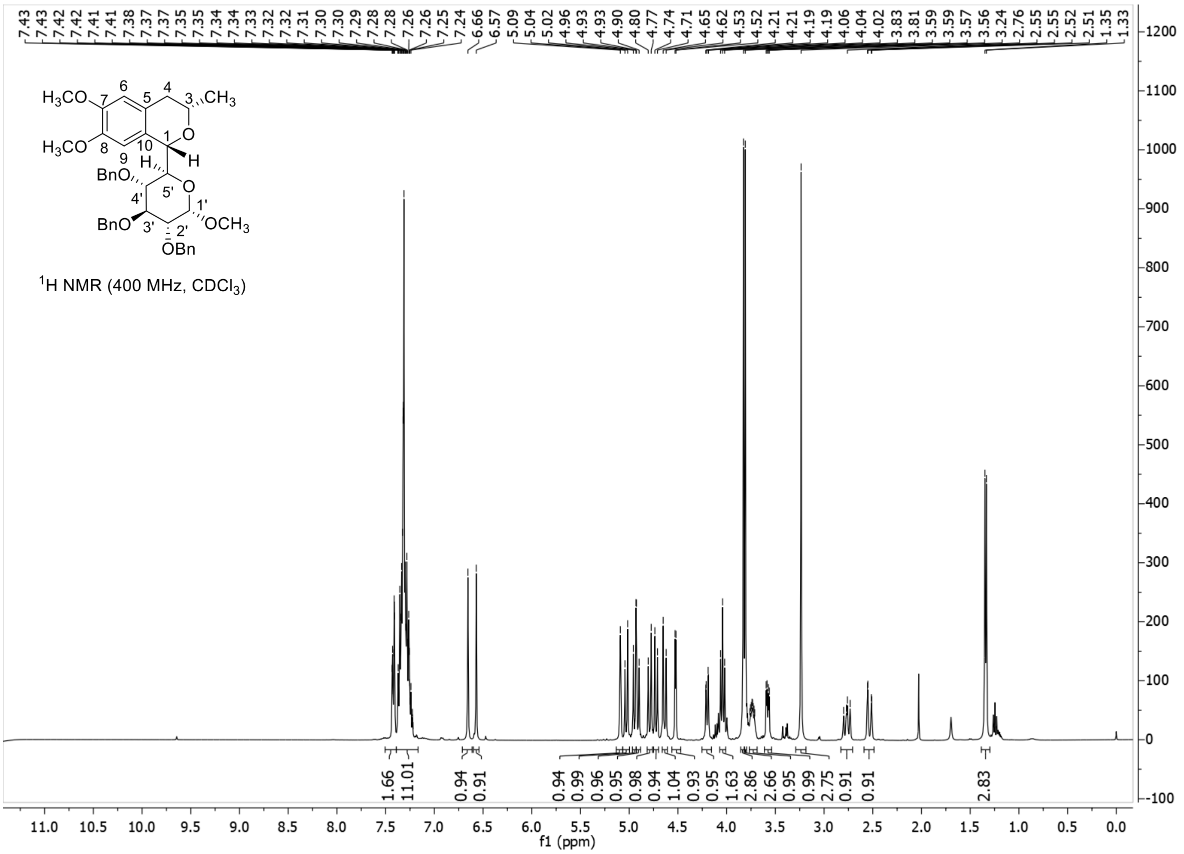

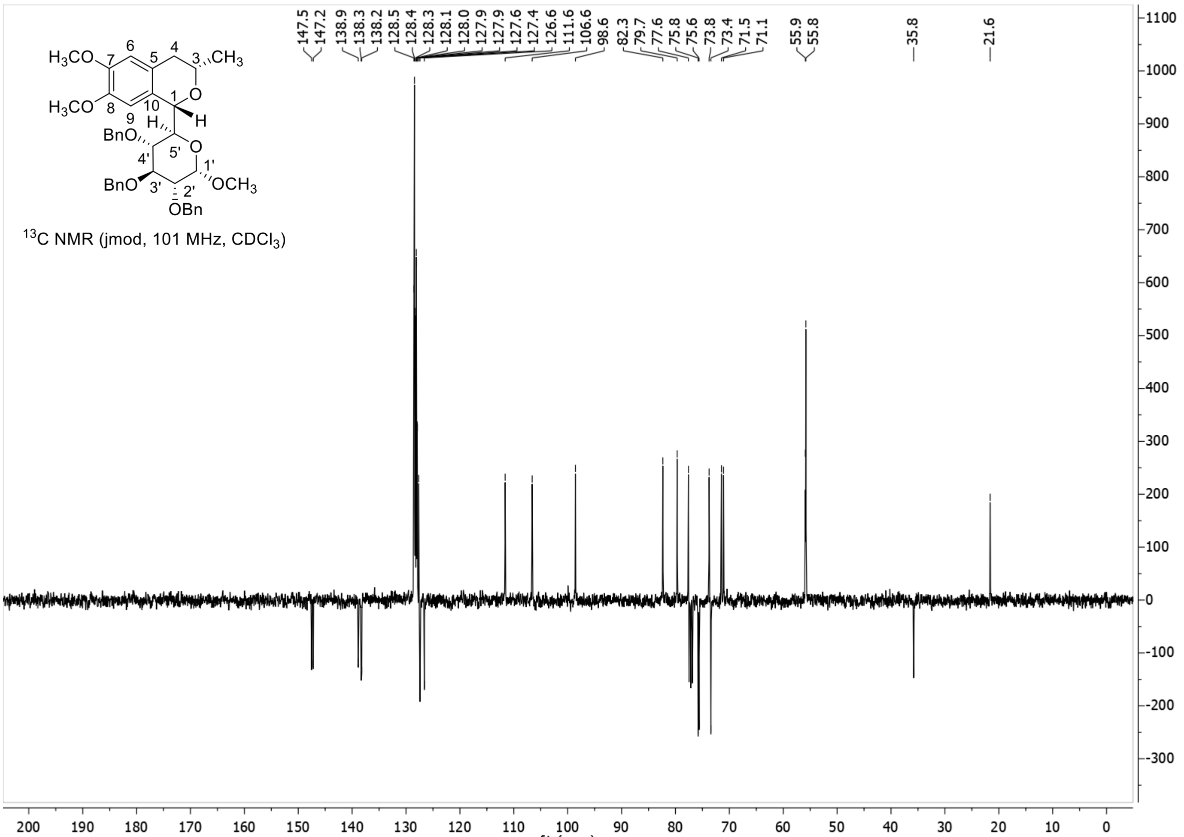

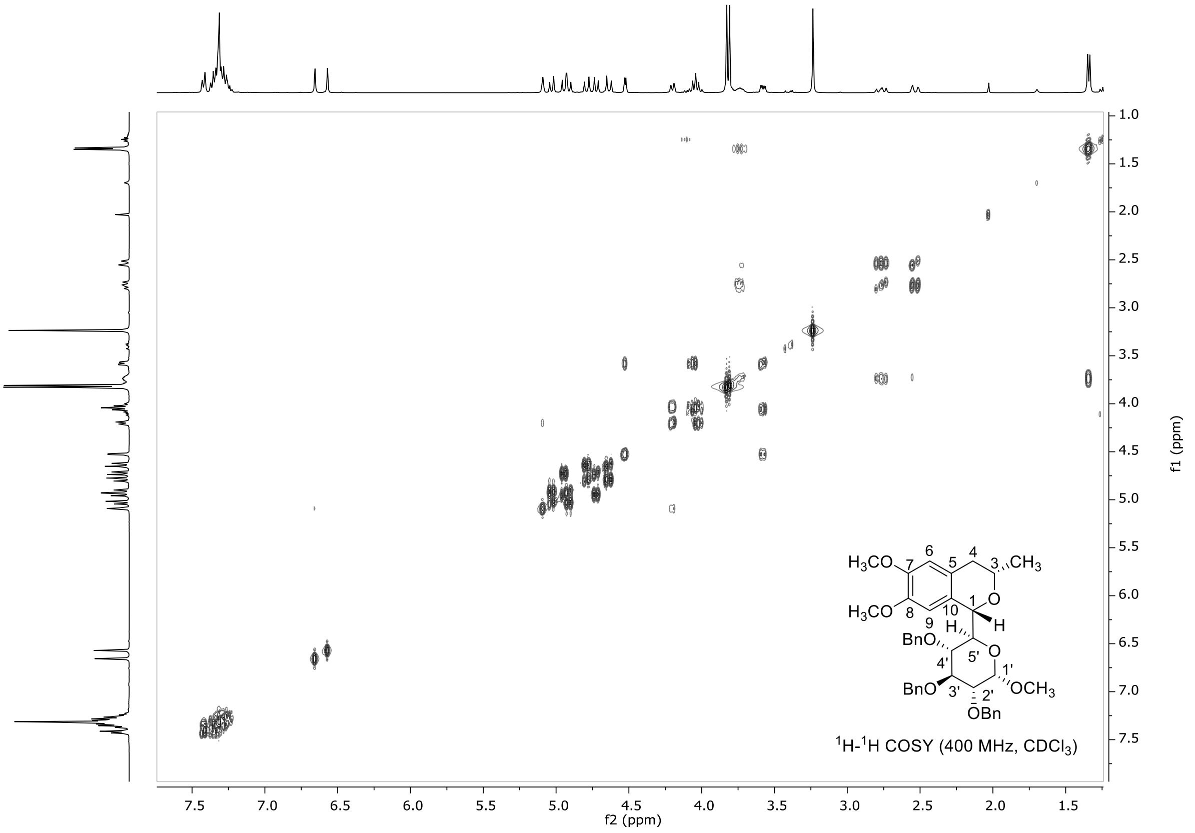
^{1}H-^{1}H COSY (400 MHz, CDCl$_3$)
H$_3$CO
H$_3$CO
CH$_3$
BnO
BnO
OBn
OCH$_3$
f2 (ppm)
f1 (ppm)

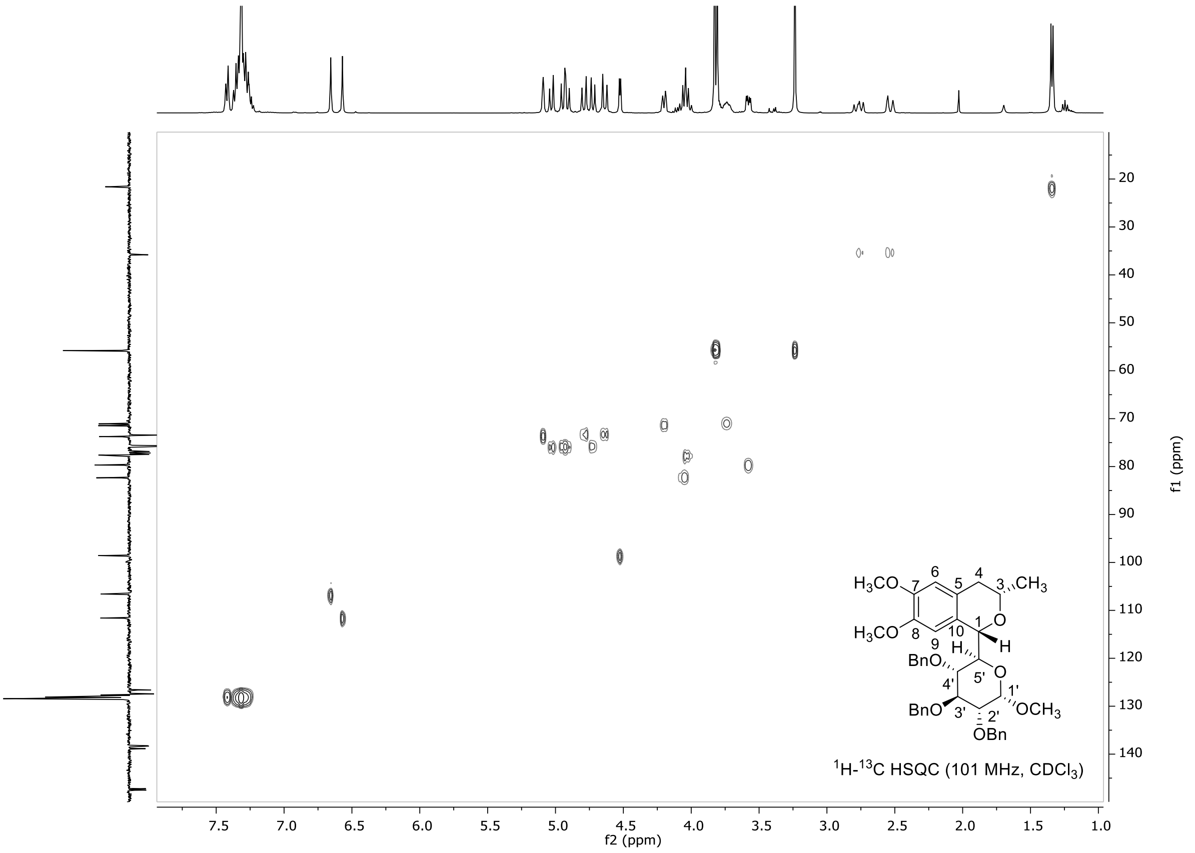
^{1}H-^{13}C HSQC (101 MHz, CDCl$_3$)
H$_3$CO
H$_3$CO
CH$_3$
BnO
BnO
OBn
OCH$_3$
f2 (ppm)
f1 (ppm)

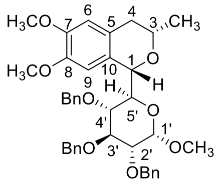

^{1}H-^{13}C HMBC (101 MHz, $CDCl_3$)

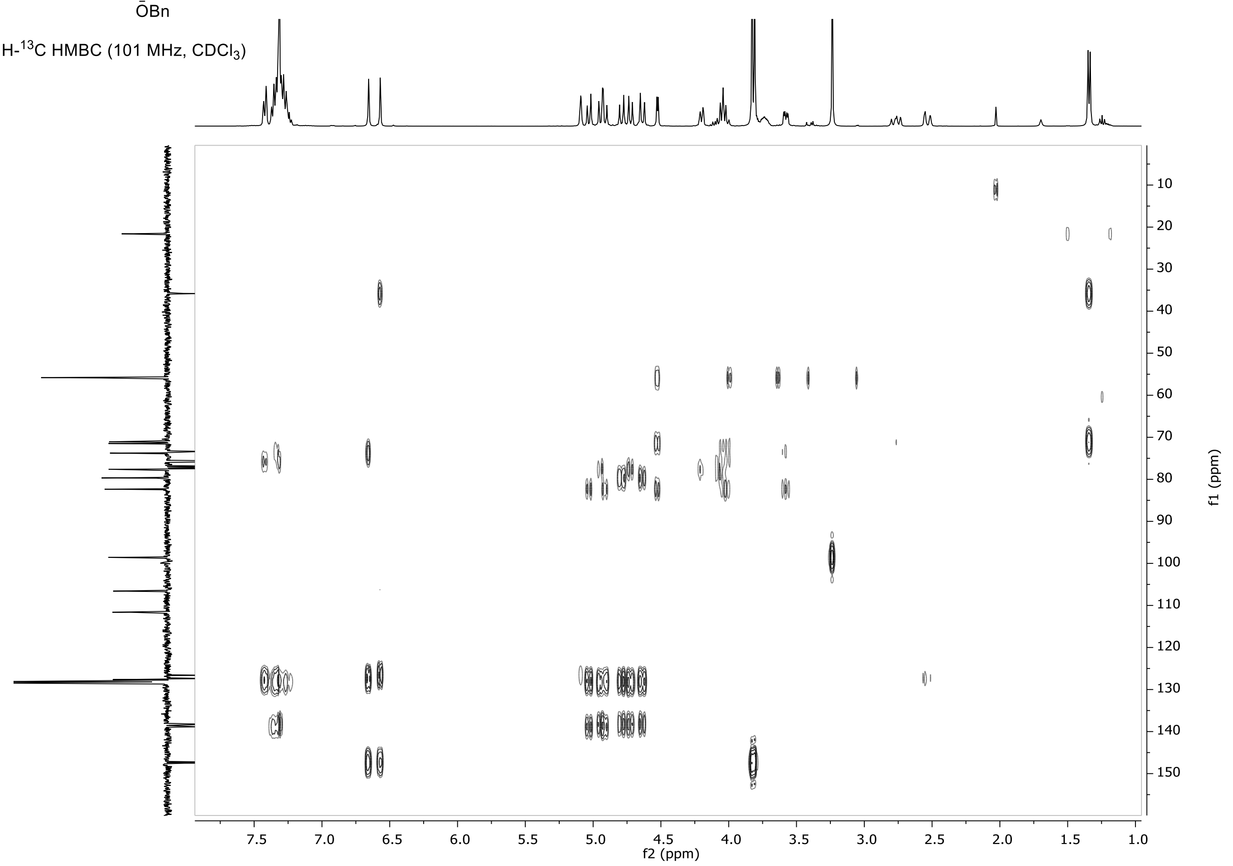

Compound 17

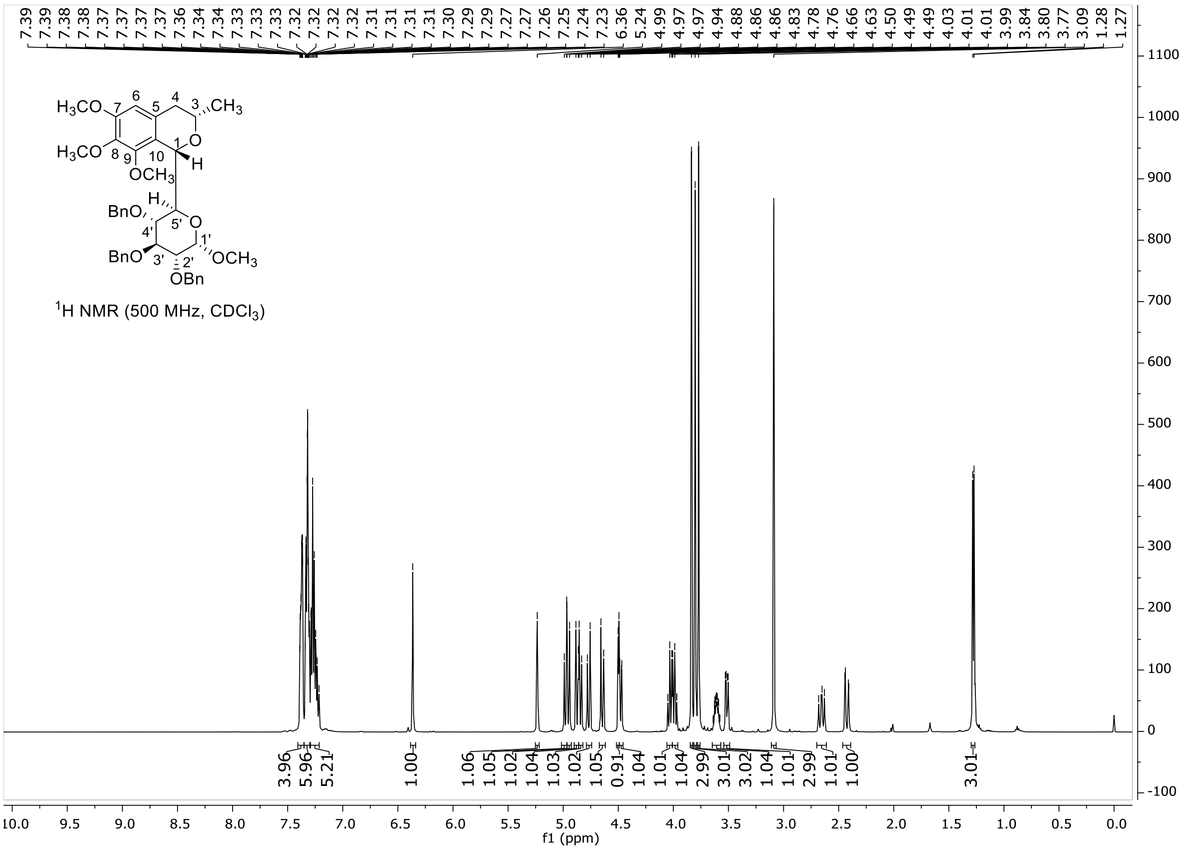

7.39 7.39 7.38 7.38 7.37 7.37 7.37 7.37 7.36 7.34 7.34 7.33 7.33 7.33 7.32 7.32 7.32 7.32 7.32 7.31 7.31 7.31 7.30 7.29 7.29 7.27 7.27 7.26 7.25 7.24 7.23 6.36 5.24 4.99 4.97 4.97 4.94 4.88 4.86 4.86 4.83 4.78 4.76 4.66 4.63 4.50 4.49 4.49 4.03 4.01 3.99 3.84 3.80 3.77 3.09 1.28 1.27
^{1}H NMR (500 MHz, CDCl$_3$)
3.96 5.96 5.21 1.00 1.06 1.05 1.02 1.04 1.03 1.02 1.05 0.91 1.04 1.01 1.04 2.99 3.01 3.02 1.04 1.01 2.99 1.01 1.00 3.01
f1 (ppm)

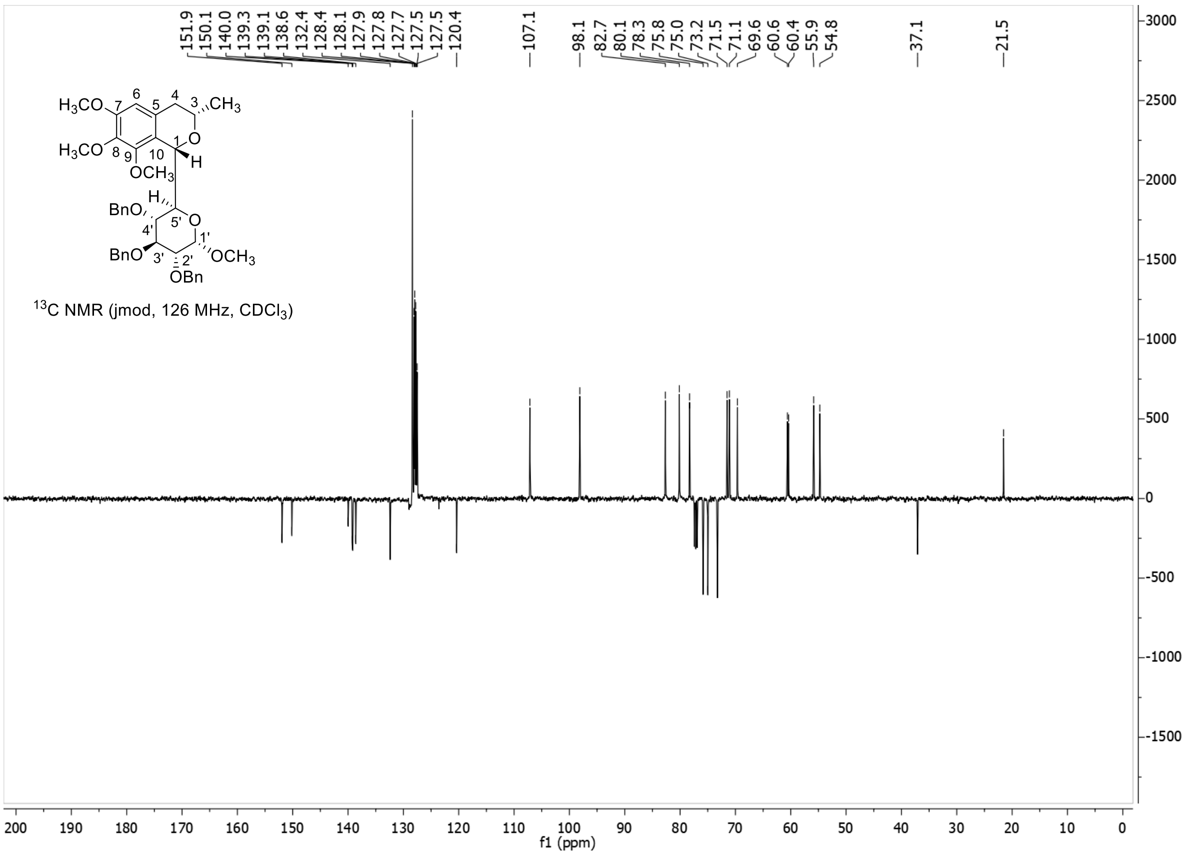

151.9 150.1 140.0 139.3 139.1 138.6 132.4 128.4 128.1 127.9 127.8 127.7 127.5 127.5 120.4 107.1 98.1 82.7 80.1 78.3 75.8 75.0 73.2 71.5 71.1 69.6 60.6 60.4 55.9 54.8 37.1 21.5
^{13}C NMR (jmod, 126 MHz, CDCl$_3$)
f1 (ppm)

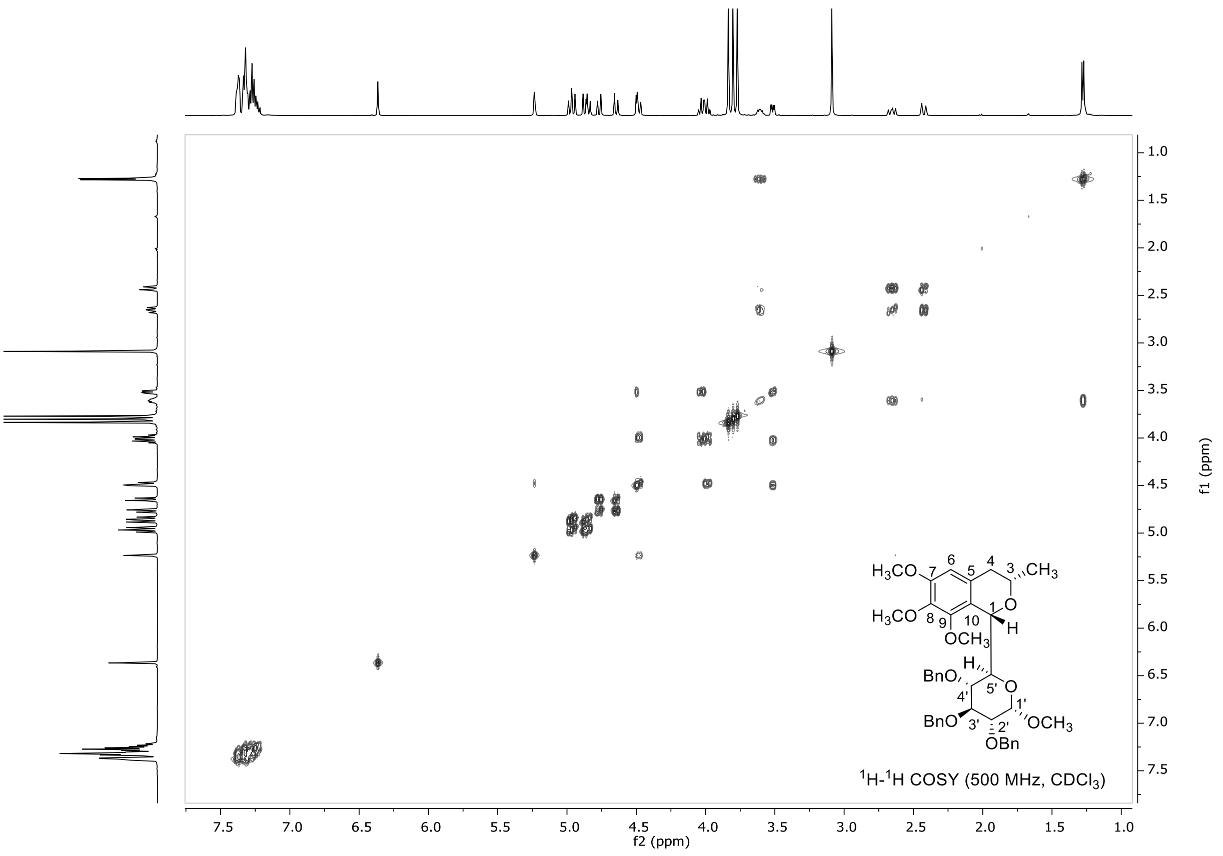

H3CO
H3CO
OCH3
CH3
BnO
BnO
OBn
OCH3
1H-1H COSY (500 MHz, CDCl3)
f2 (ppm)
f1 (ppm)

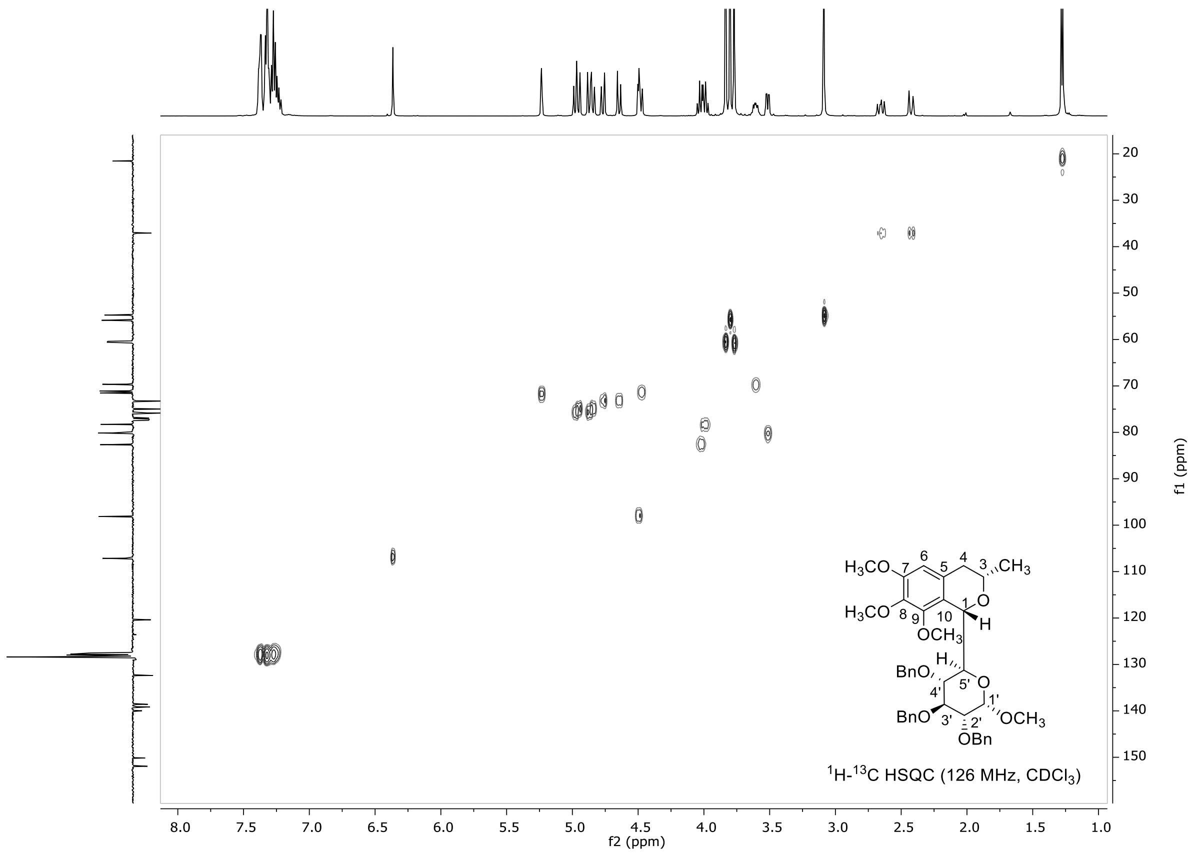

H3CO
H3CO
OCH3
CH3
BnO
BnO
OBn
OCH3
1H-13C HSQC (126 MHz, CDCl3)
f2 (ppm)
f1 (ppm)

Compound 18

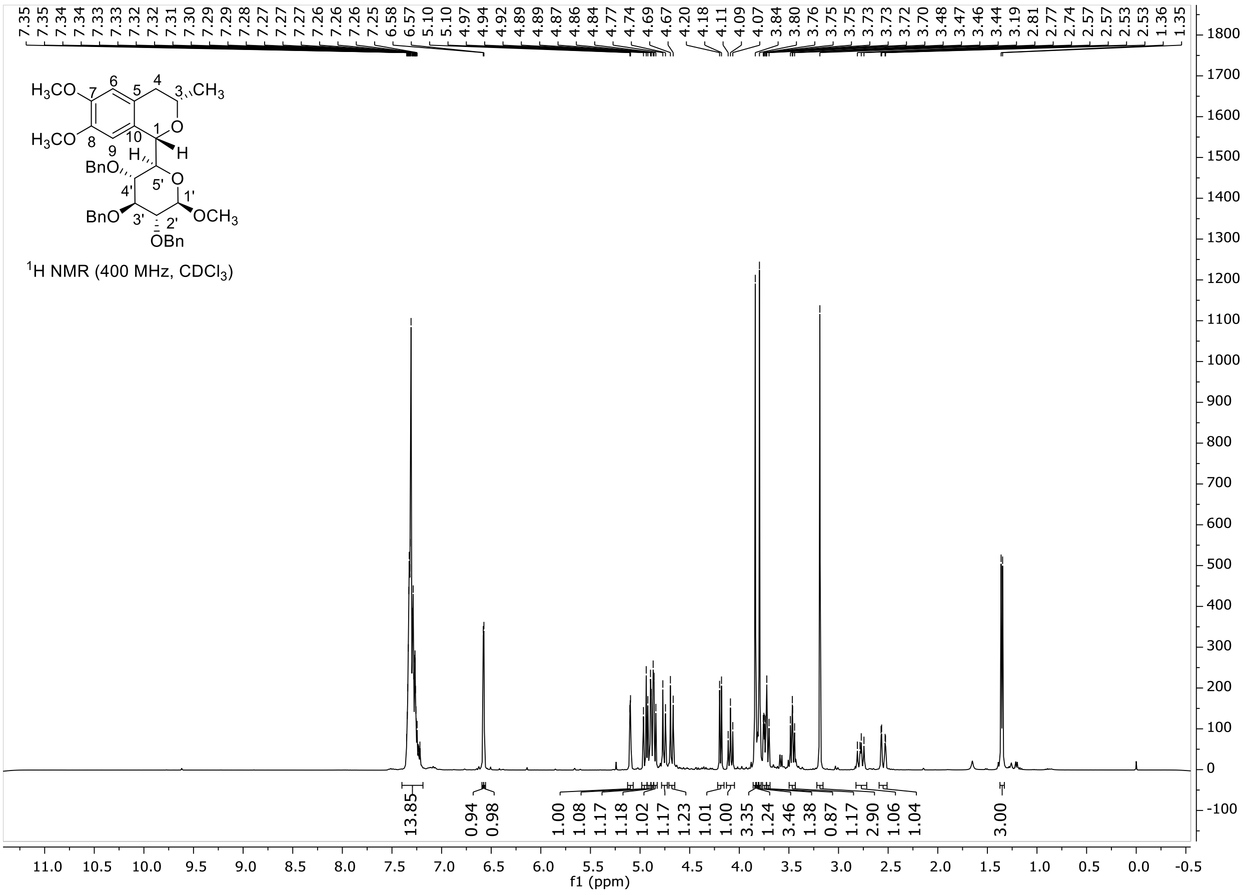

7.35 7.35 7.34 7.33 7.33 7.32 7.32 7.31 7.30 7.29 7.29 7.28 7.27 7.27 7.27 7.26 7.26 7.26 7.25 6.58 6.57 5.10 5.10 4.97 4.94 4.92 4.89 4.89 4.87 4.86 4.84 4.77 4.74 4.69 4.67 4.20 4.18 4.11 4.09 4.07 3.84 3.80 3.76 3.75 3.75 3.73 3.72 3.70 3.48 3.47 3.46 3.44 3.19 2.81 2.77 2.74 2.57 2.57 2.53 2.53 1.36 1.35
^{1}H NMR (400 MHz, $CDCl_3$)
13.85 0.94 0.98 1.00 1.08 1.17 1.18 1.02 1.17 1.23 1.01 1.00 3.35 1.24 3.46 1.38 0.87 1.17 2.90 1.06 1.04 3.00
f1 (ppm)

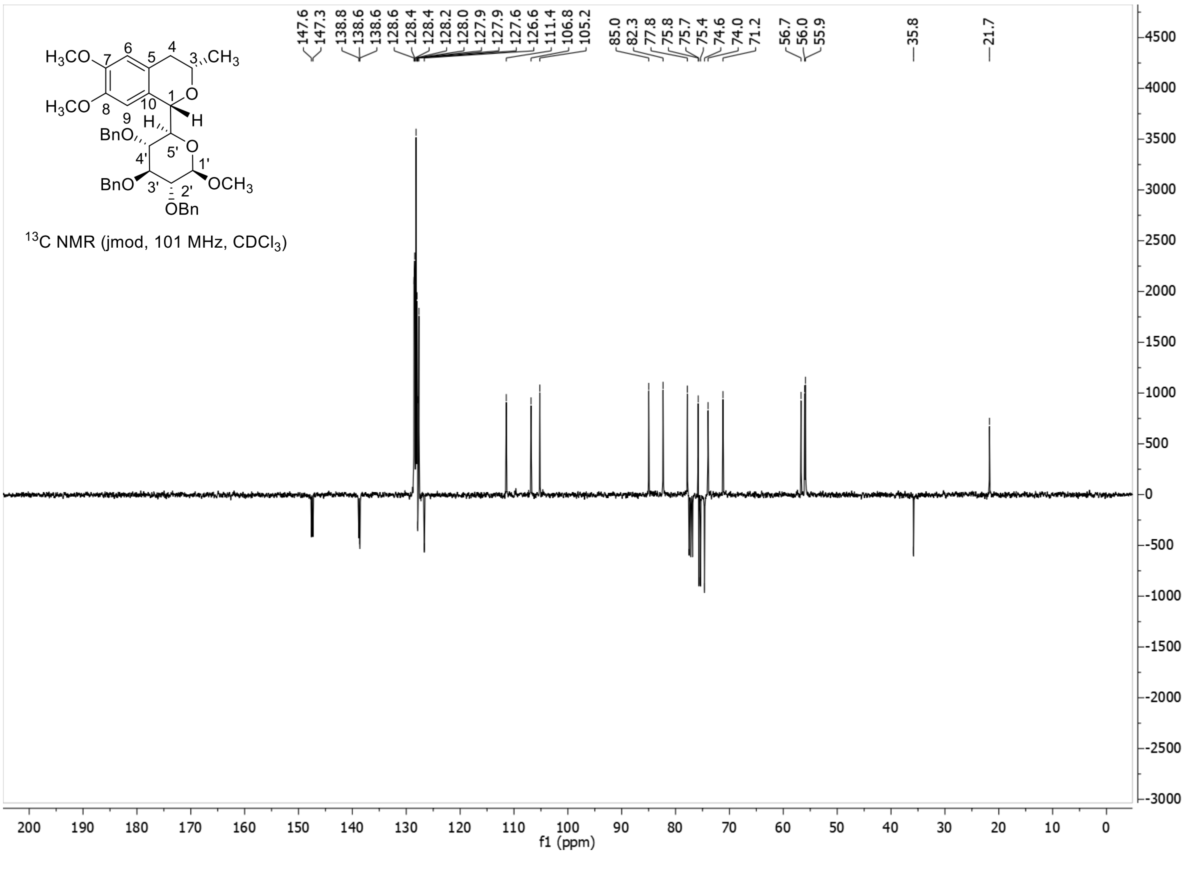

147.6 147.3 138.8 138.6 138.6 128.6 128.4 128.4 128.2 128.0 127.9 127.9 127.6 126.6 111.4 106.8 105.2 85.0 82.3 77.8 75.8 75.7 75.4 74.6 74.0 71.2 56.7 56.0 55.9 35.8 21.7
^{13}C NMR (jmod, 101 MHz, $CDCl_3$)
f1 (ppm)

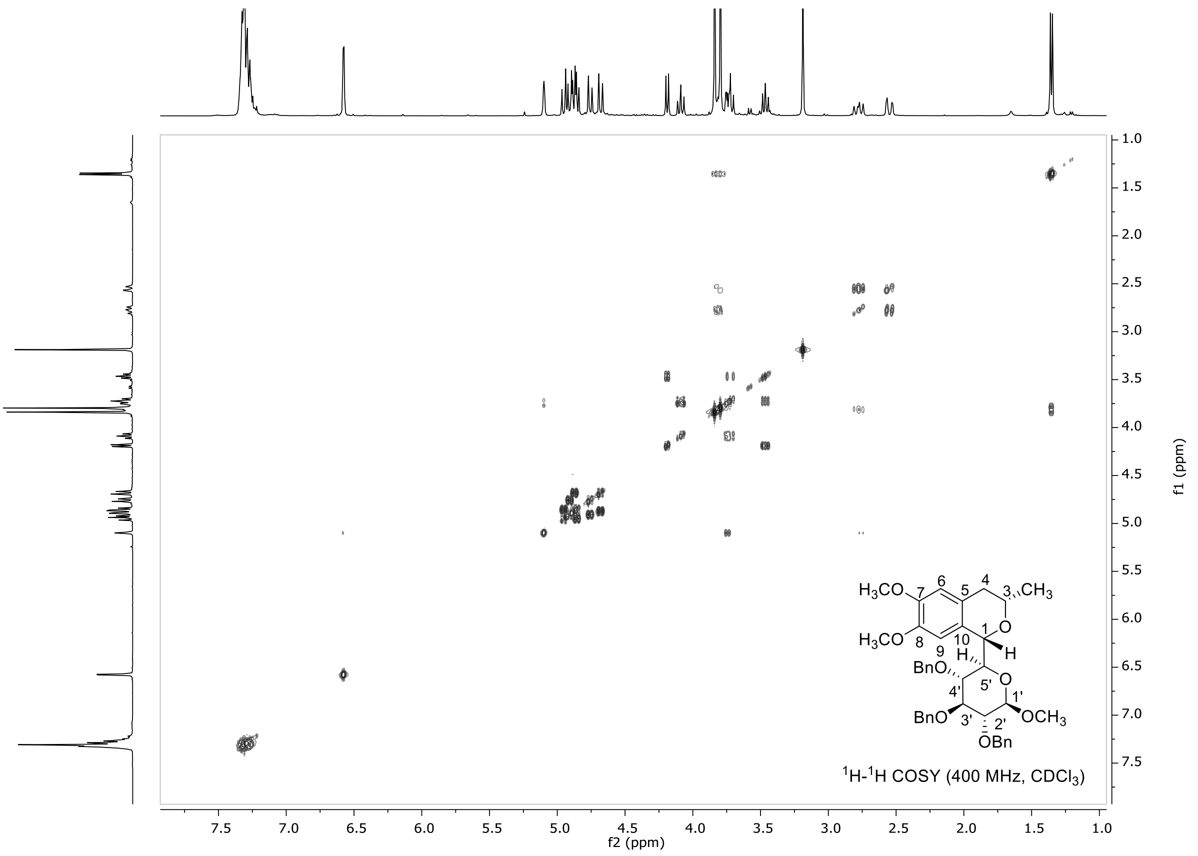
H3CO
H3CO
BnO
BnO
OBn
OCH3
CH3
1H-1H COSY (400 MHz, CDCl3)
f2 (ppm)
f1 (ppm)

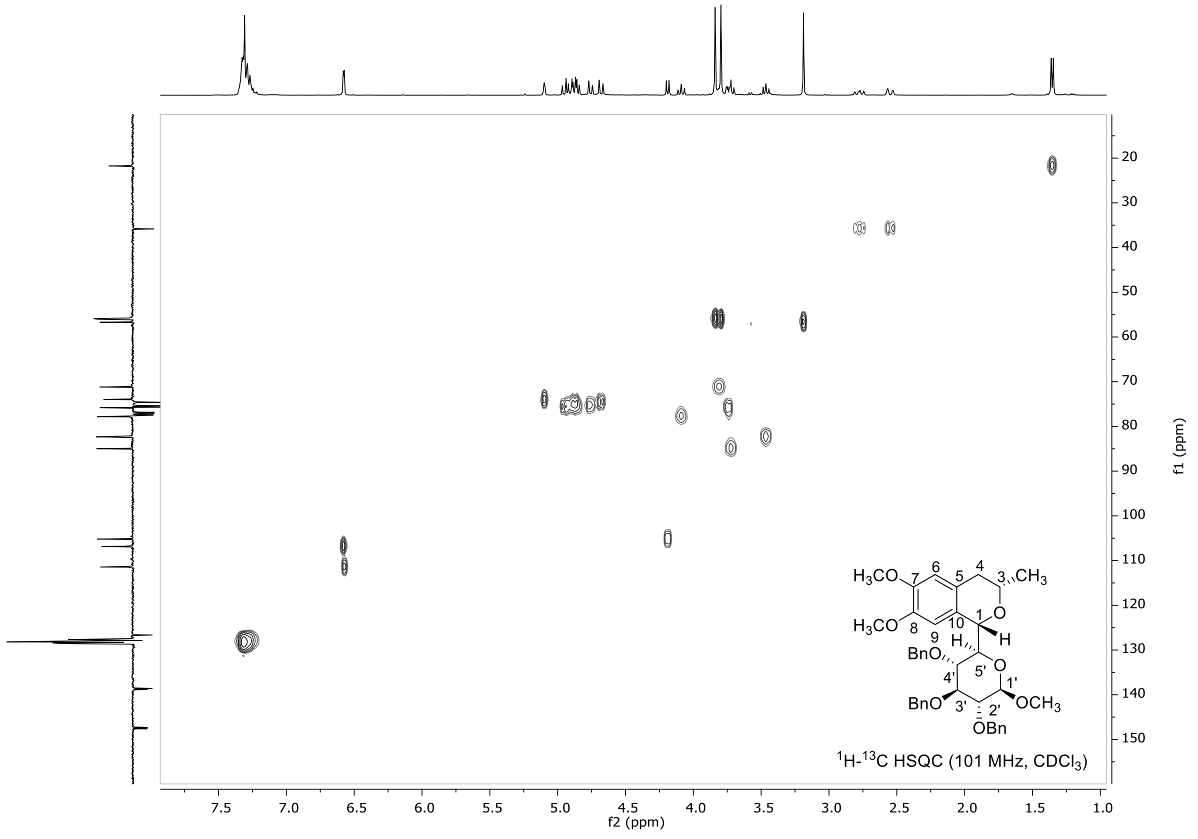
H3CO
H3CO
BnO
BnO
OBn
OCH3
CH3
1H-13C HSQC (101 MHz, CDCl3)
f2 (ppm)
f1 (ppm)

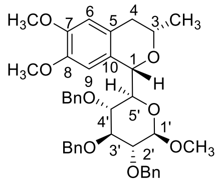

^{1}H-^{13}C HMBC (101 MHz, $CDCl_3$)

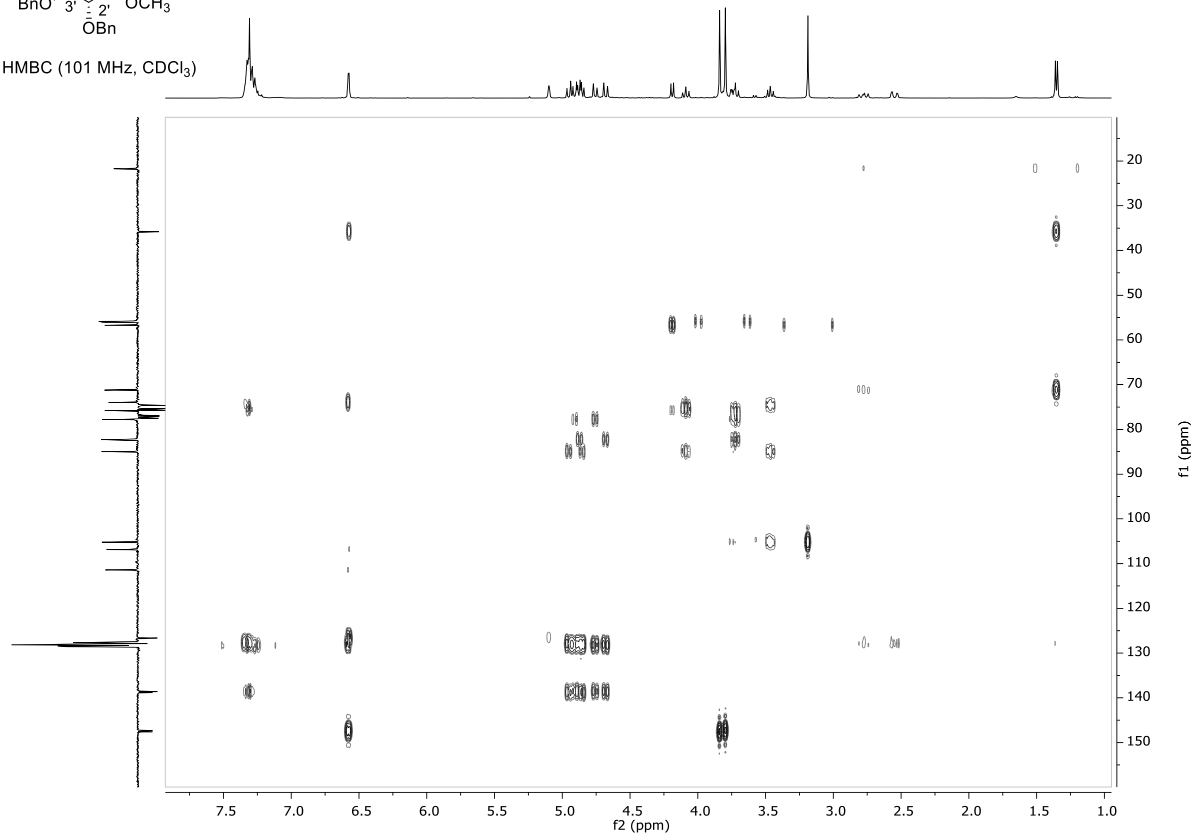

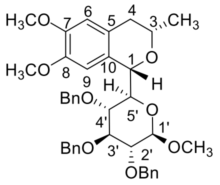

^{1}H-^{1}H ROESY (400 MHz, $CDCl_3$)

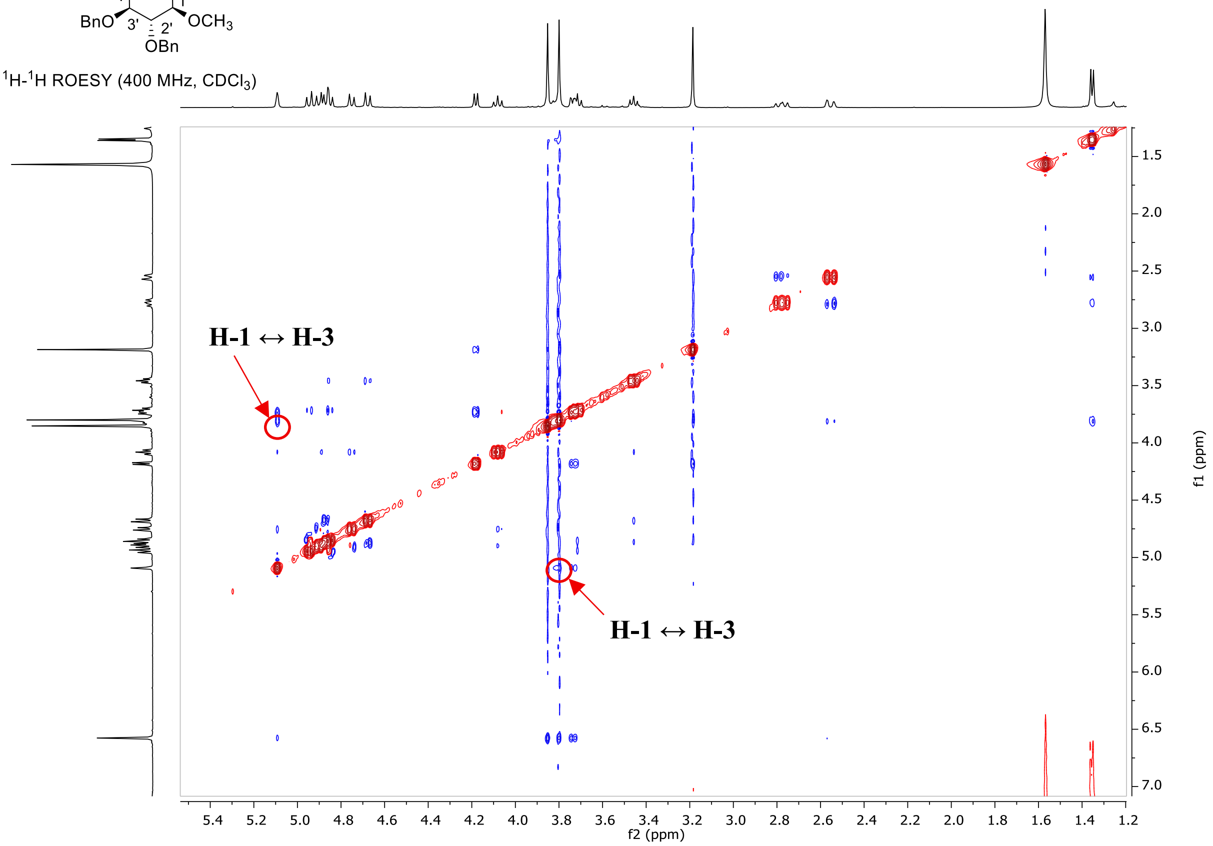

Compound 19

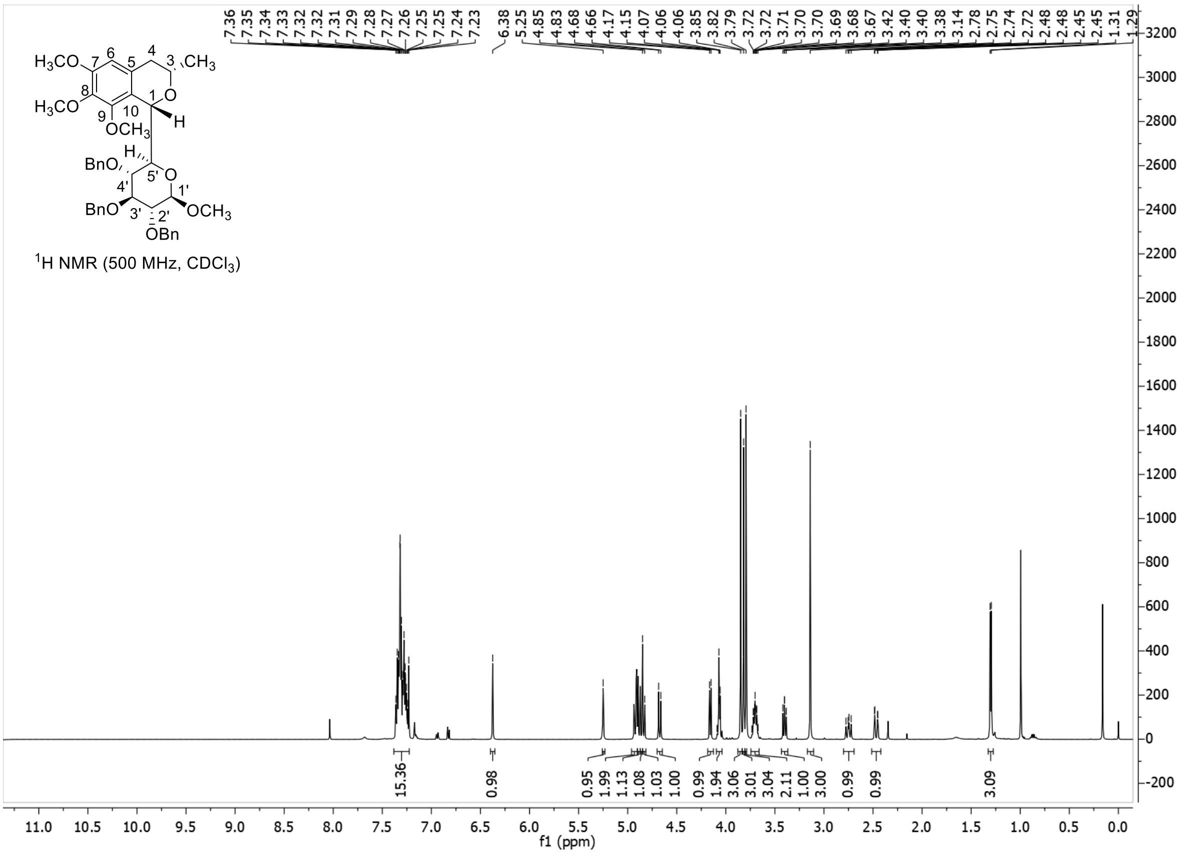
H3CO
H3CO
OCH3
BnO
BnO
OBn
OCH3
1H NMR (500 MHz, CDCl3)
f1 (ppm)

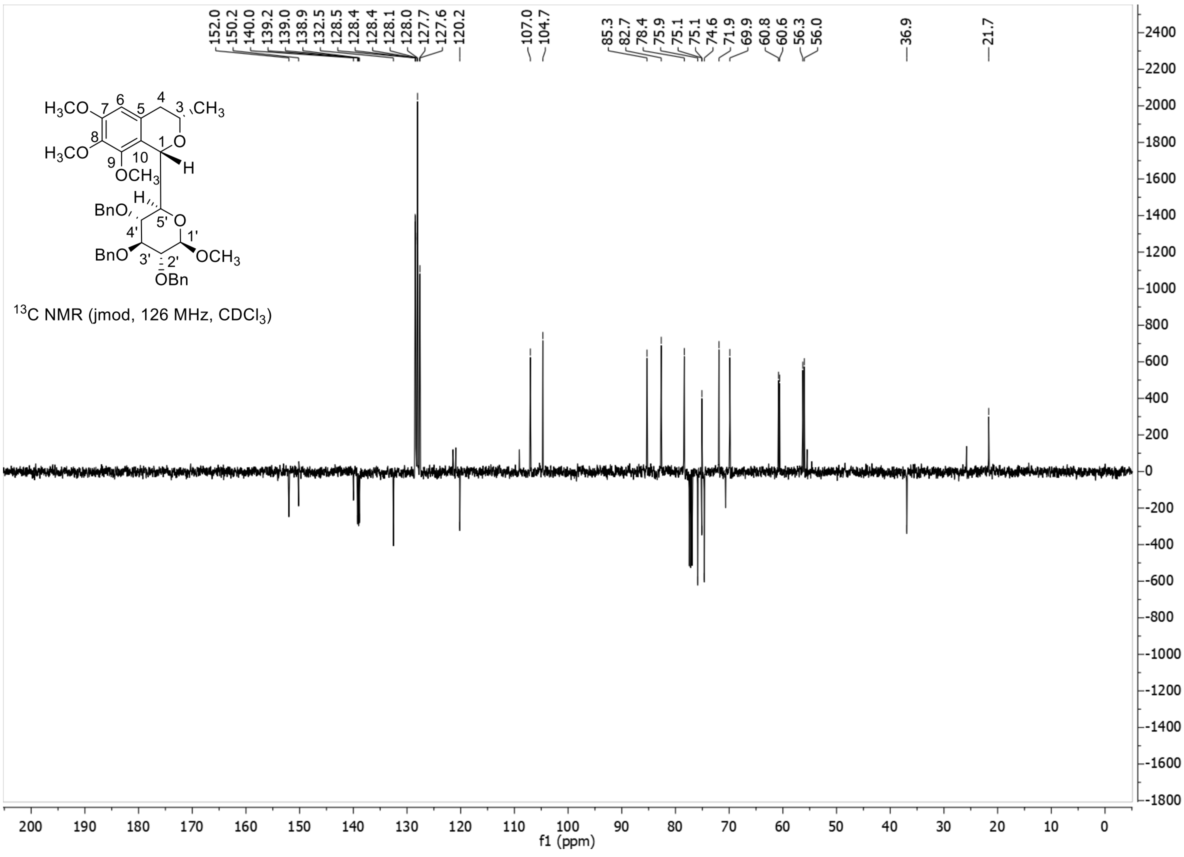
13C NMR (jmod, 126 MHz, CDCl3)
f1 (ppm)

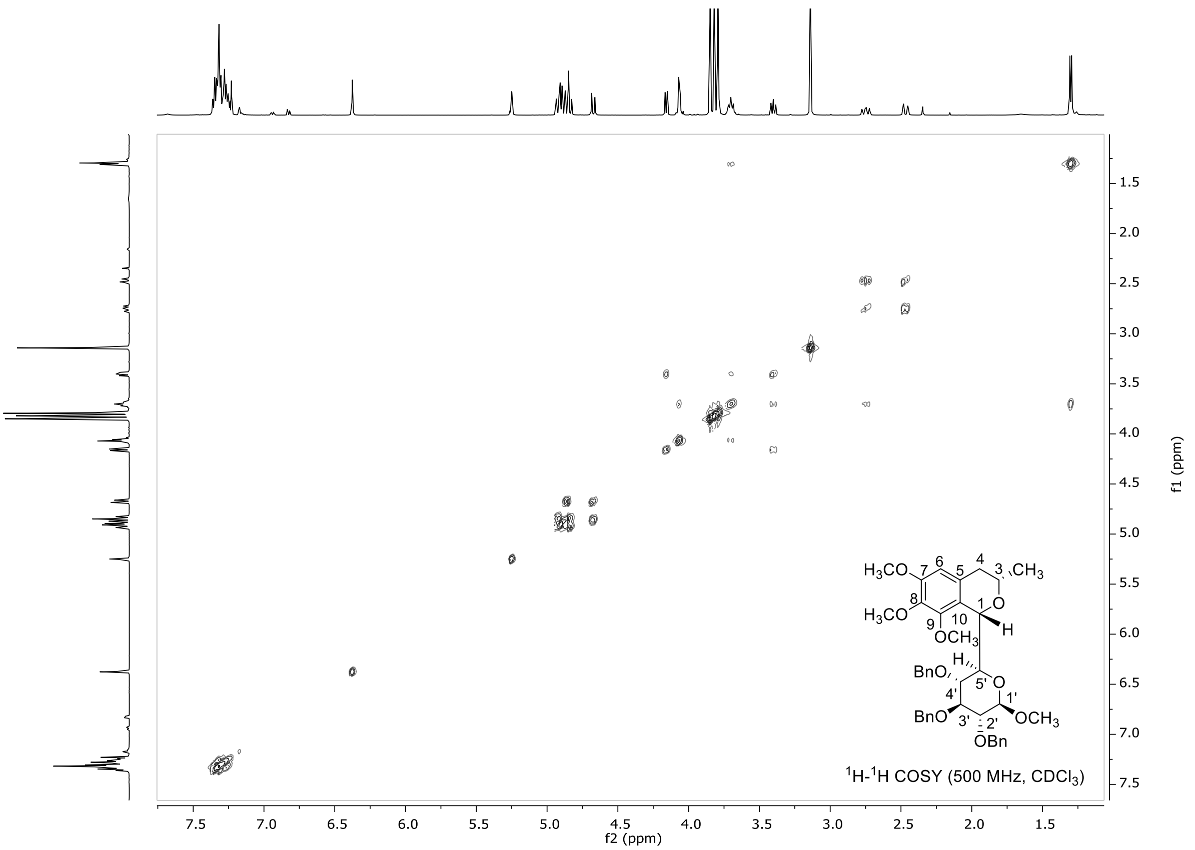
H3CO
H3CO
OCH3
CH3
H
BnO
BnO
OBn
OCH3
1H-1H COSY (500 MHz, CDCl3)
f2 (ppm)
f1 (ppm)

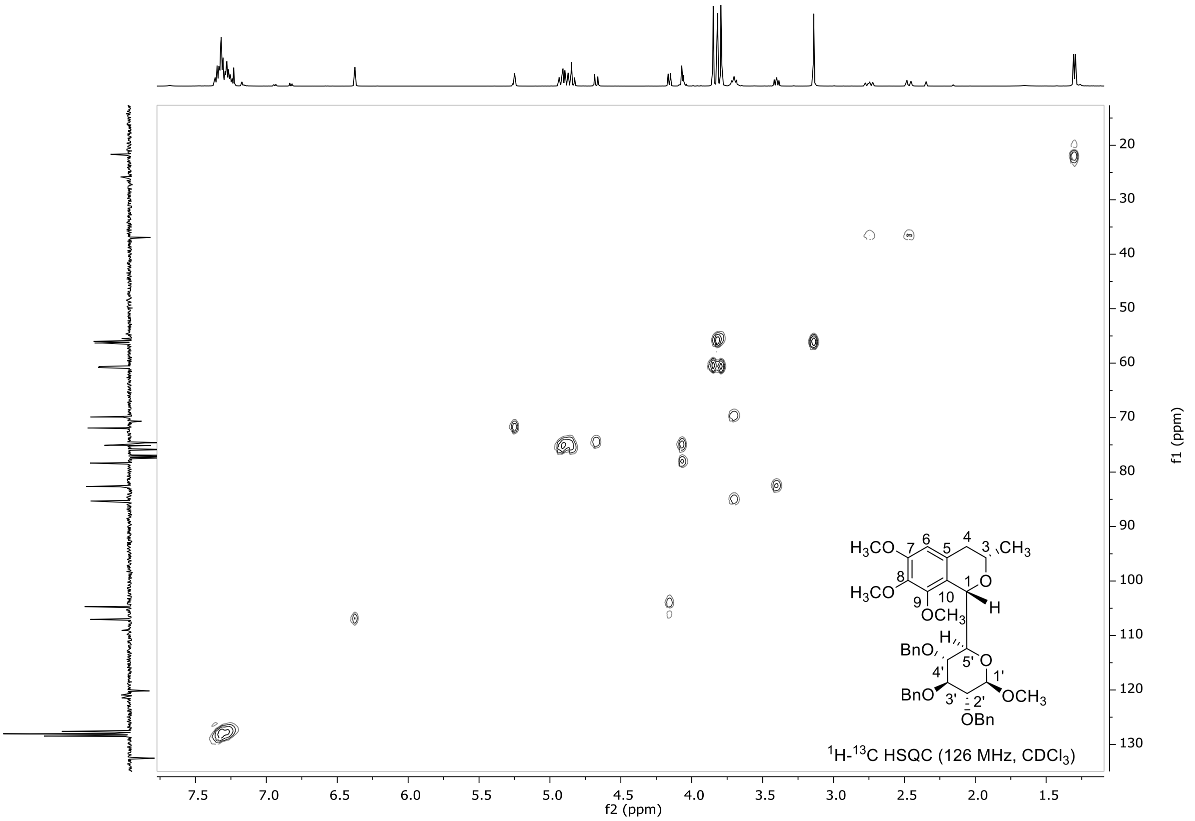
H3CO
H3CO
OCH3
CH3
H
BnO
BnO
OBn
OCH3
1H-13C HSQC (126 MHz, CDCl3)
f2 (ppm)
f1 (ppm)

Compound 20

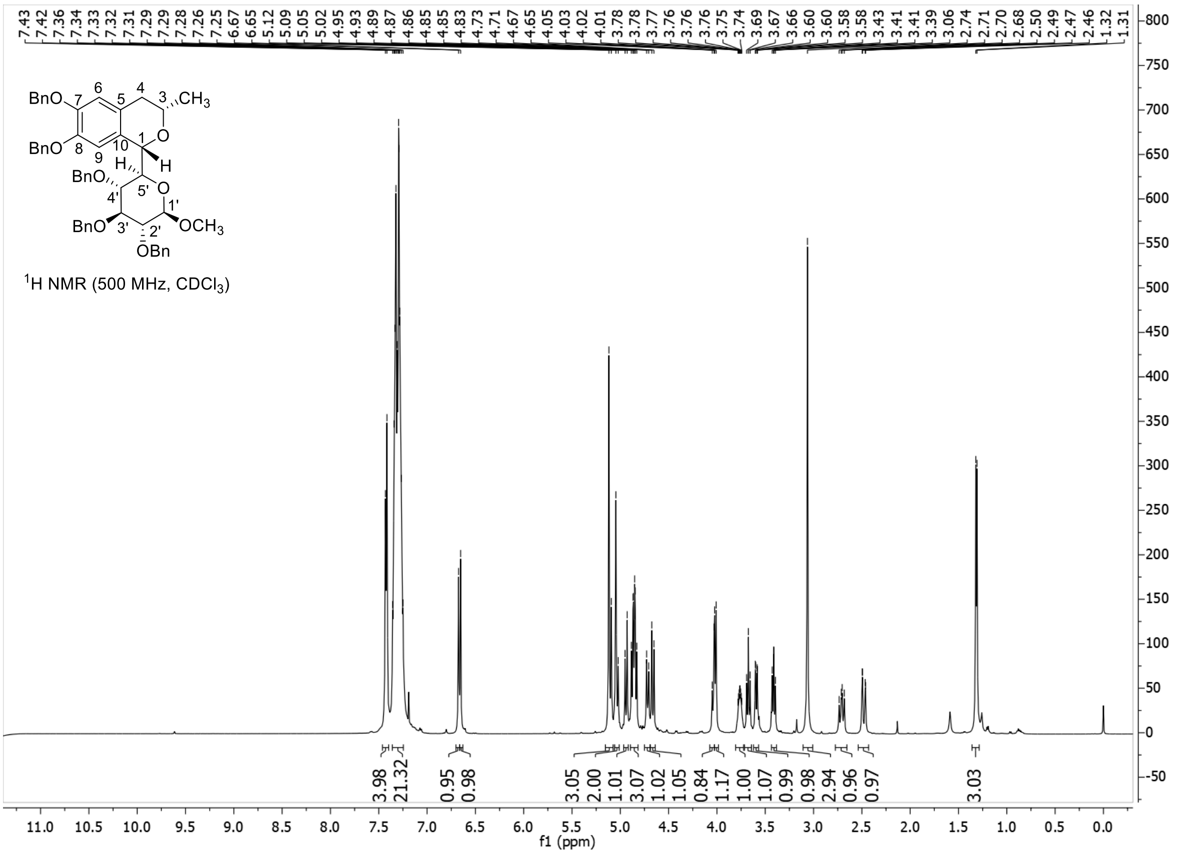

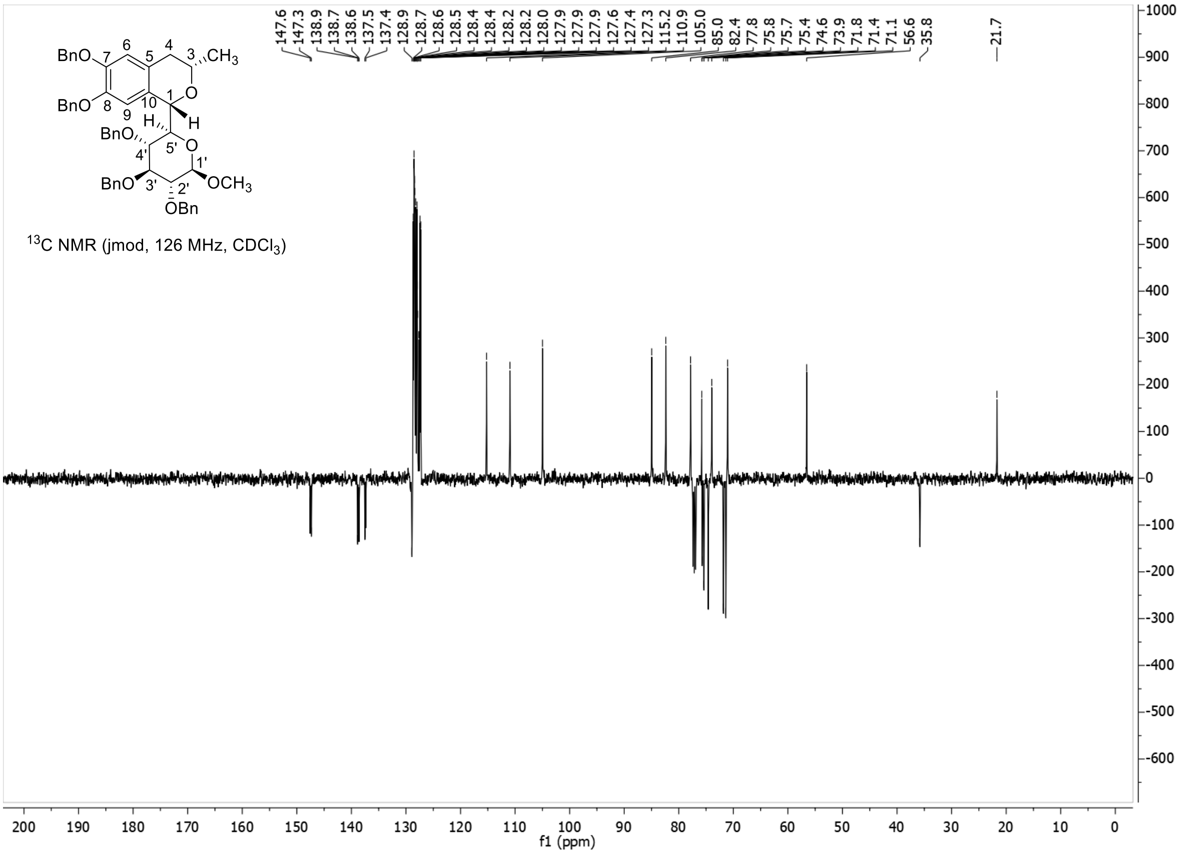

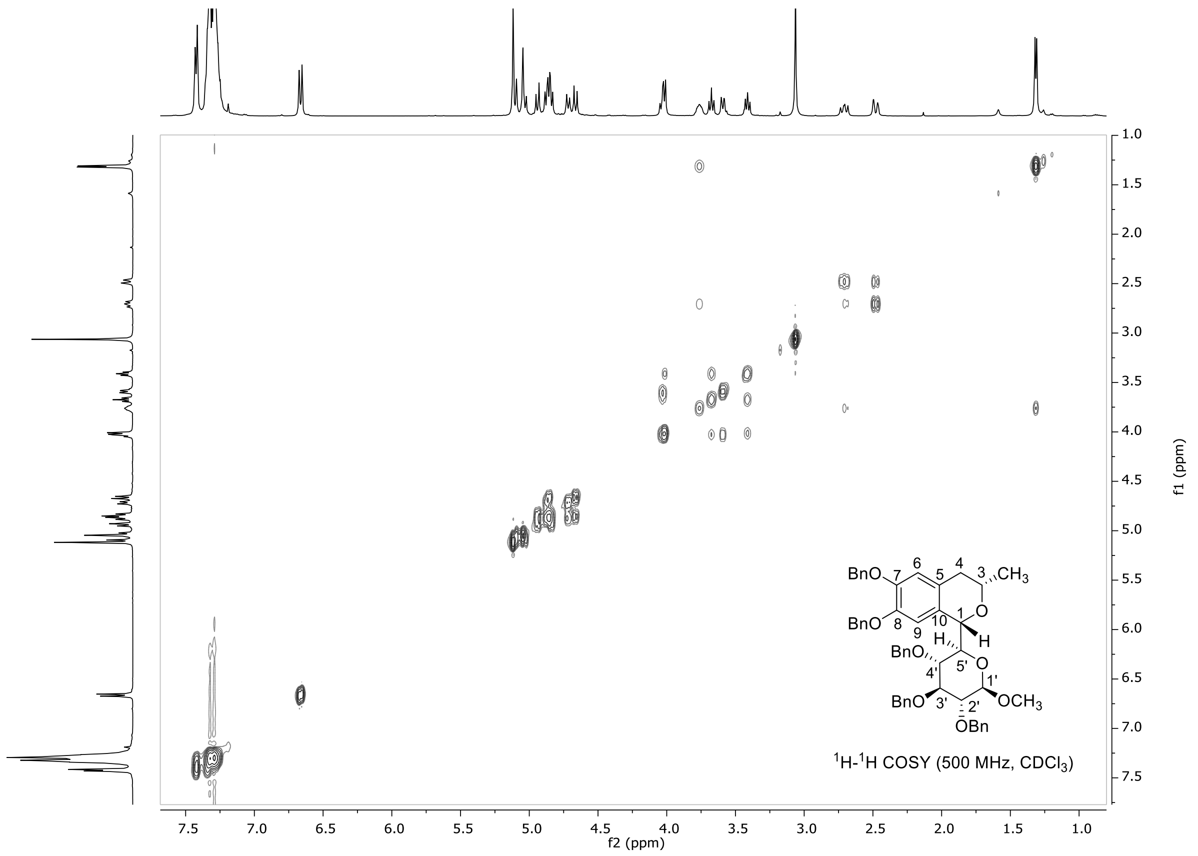
BnO
BnO
CH3
OCH3
OBn
1H-1H COSY (500 MHz, CDCl3)
f2 (ppm)
f1 (ppm)

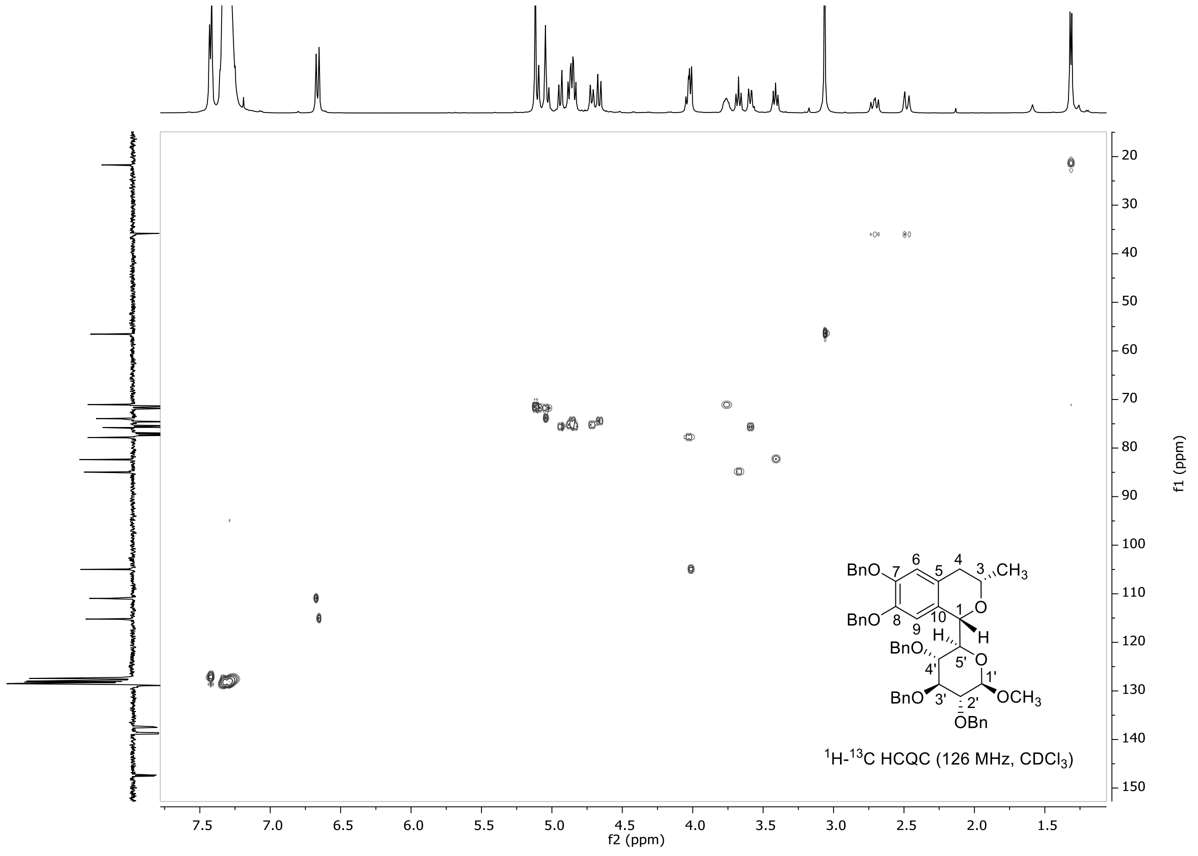
BnO
BnO
CH3
OCH3
OBn
1H-13C HCQC (126 MHz, CDCl3)
f2 (ppm)
f1 (ppm)

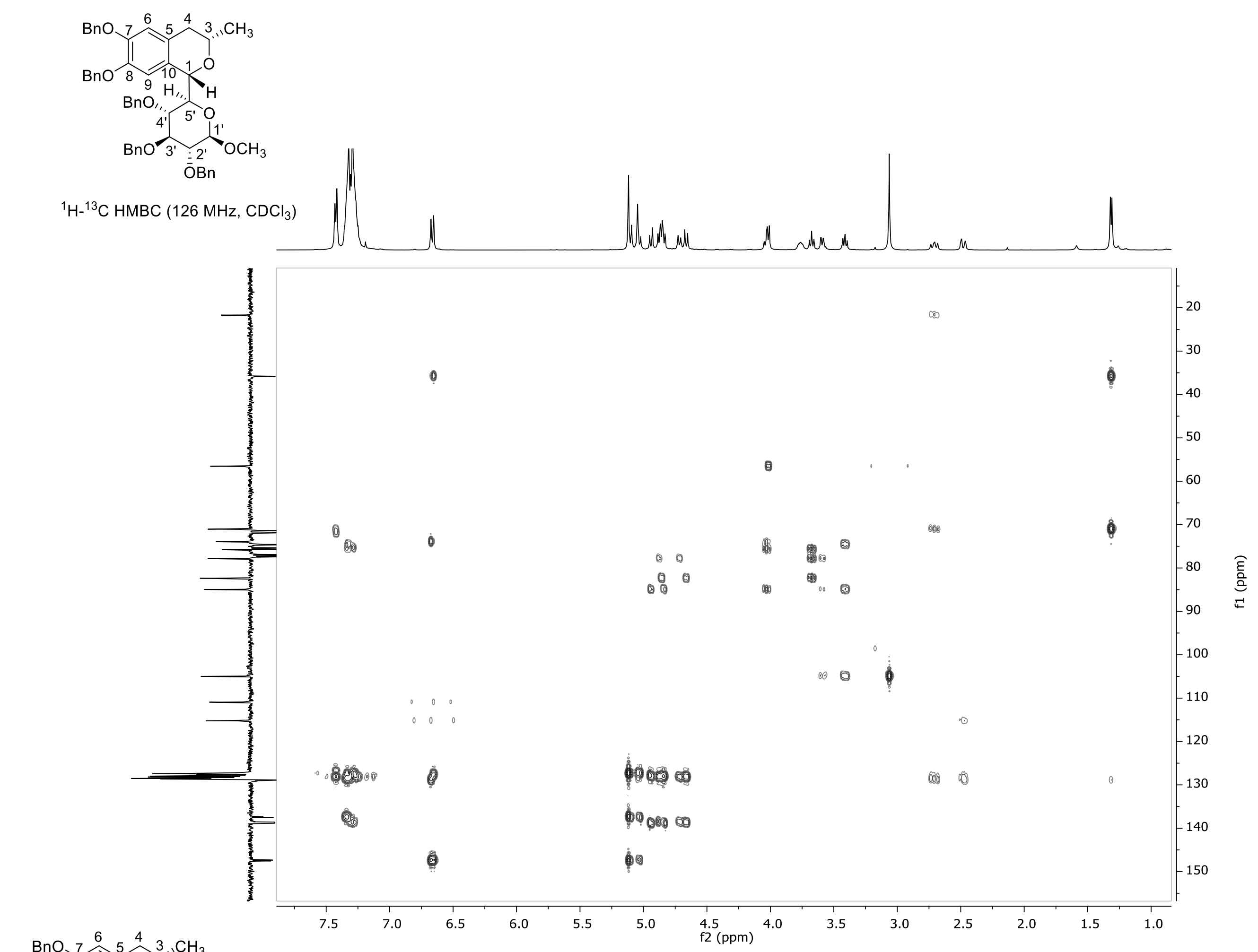
BnO
7
6
5
4
3
CH3
BnO
8
9
10
1
O
H
H
BnO
4'
5'
O
1'
BnO
3'
2'
OCH3
OBn
^{1}H-^{13}C HMBC (126 MHz, $CDCl_3$)
f2 (ppm)
f1 (ppm)

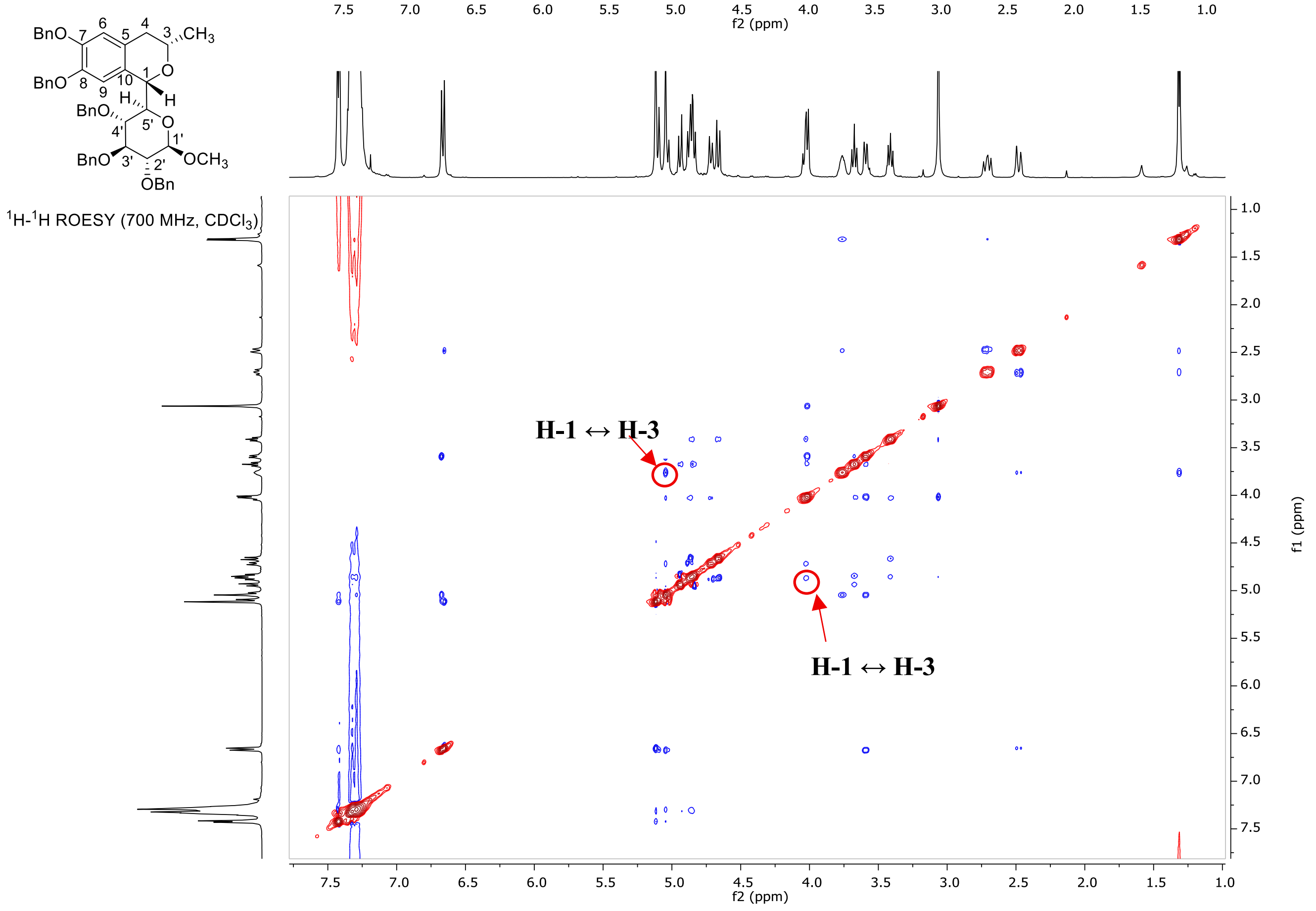
^{1}H-^{1}H ROESY (700 MHz, $CDCl_3$)
H-1 ↔ H-3
H-1 ↔ H-3
f2 (ppm)
f1 (ppm)

Compound 21

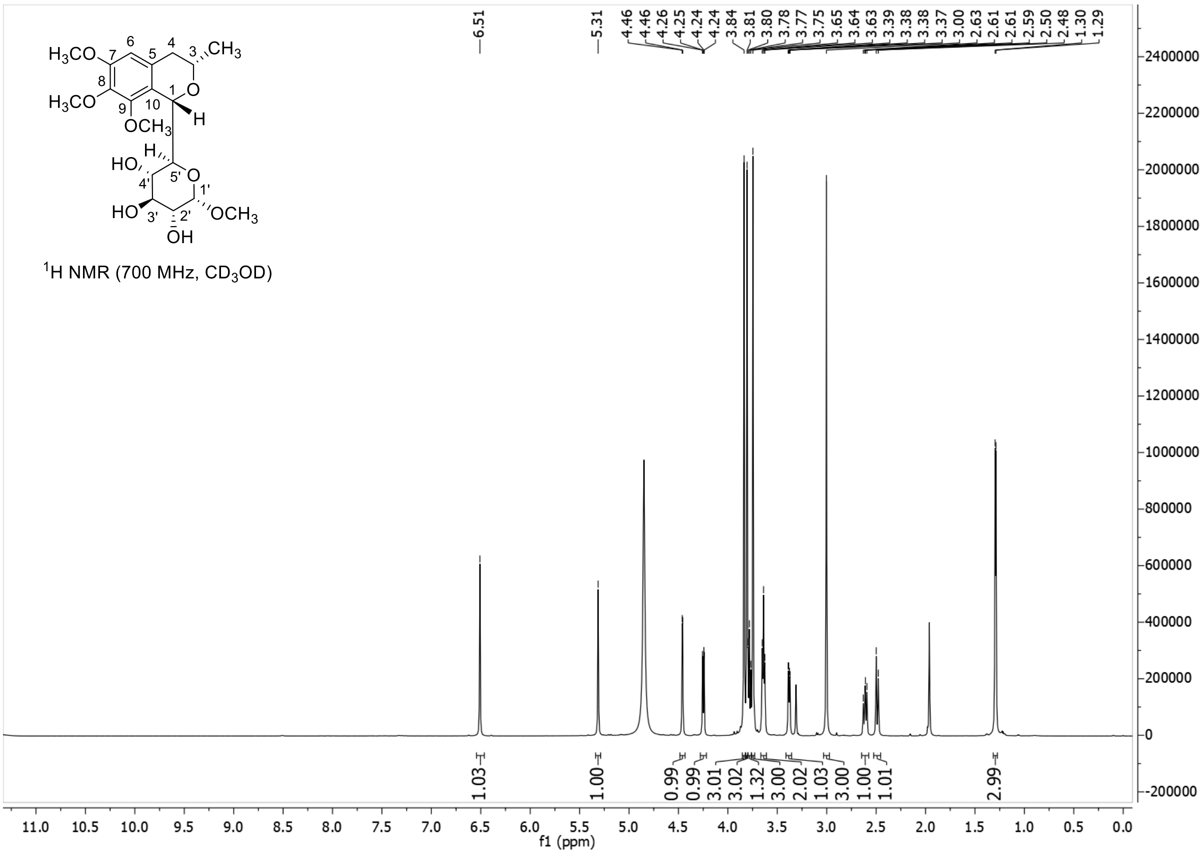

1H NMR (700 MHz, CD3OD)
6.51
5.31
4.46 4.46 4.26 4.25 4.24 4.24 3.84 3.81 3.80 3.78 3.77 3.75 3.65 3.64 3.63 3.39 3.38 3.38 3.37 3.00 2.63 2.61 2.61 2.59 2.50 2.48 1.30 1.29
1.03 1.00 0.99 0.99 3.01 3.02 1.32 3.00 2.02 1.03 3.00 1.00 1.01 2.99
f1 (ppm)

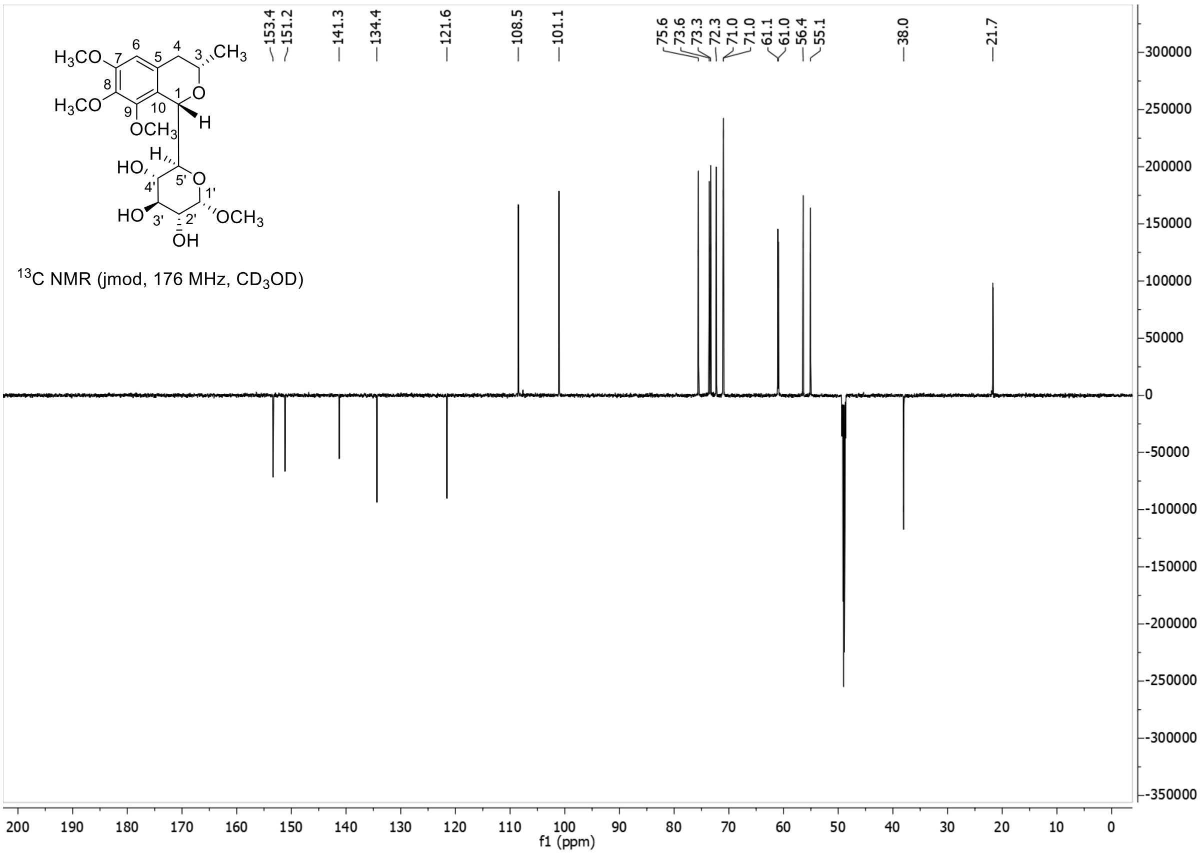

13C NMR (jmod, 176 MHz, CD3OD)
153.4 151.2 141.3 134.4 121.6 108.5 101.1 75.6 73.6 73.3 72.3 71.0 71.0 61.1 61.0 56.4 55.1 38.0 21.7
f1 (ppm)

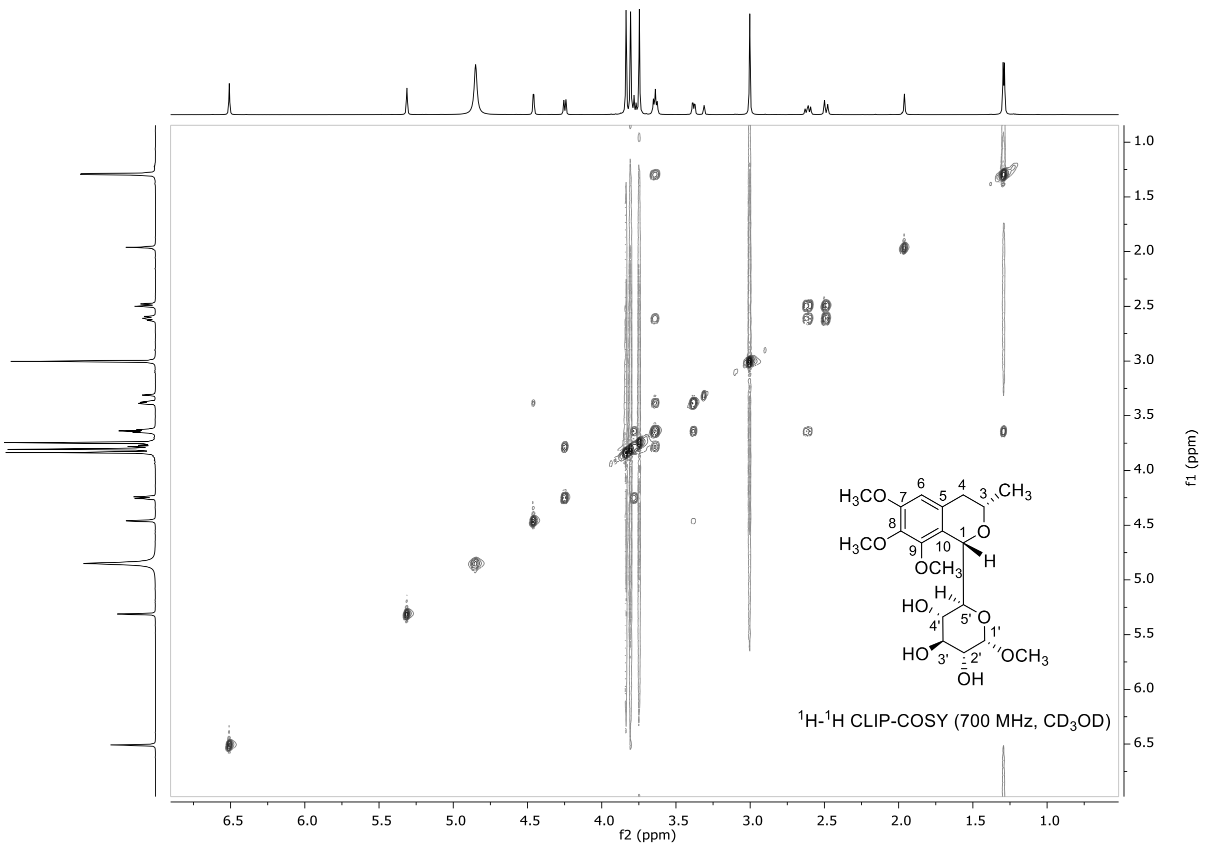

H3CO 7 6 5 4 3 CH3
8 O
H3CO 10 1
9 OCH3 H
H
HO 5' O
4' 1'
HO 3' 2' OCH3
OH
1H-1H CLIP-COSY (700 MHz, CD3OD)
f2 (ppm)
f1 (ppm)

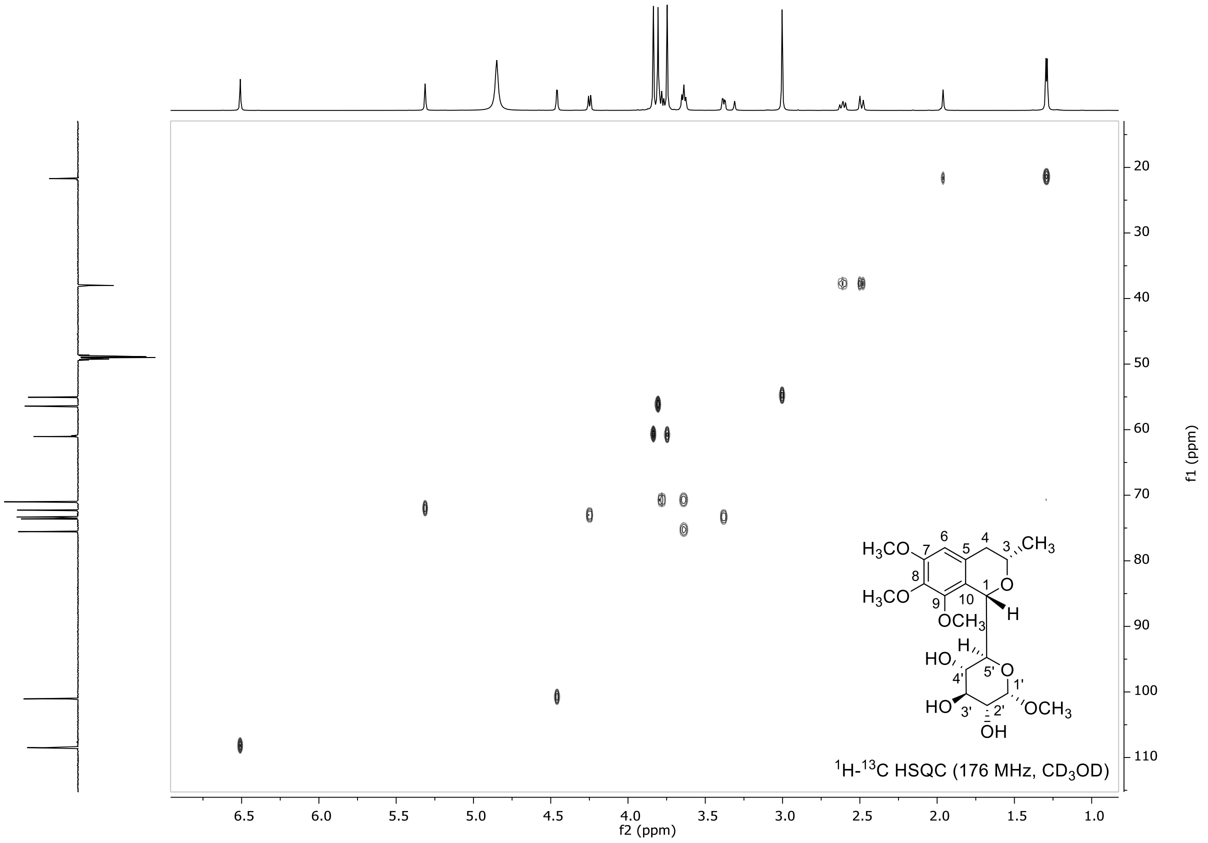

H3CO 7 6 5 4 3 CH3
8 O
H3CO 10 1
9 OCH3 H
H
HO 5' O
4' 1'
HO 3' 2' OCH3
OH
1H-13C HSQC (176 MHz, CD3OD)
f2 (ppm)
f1 (ppm)

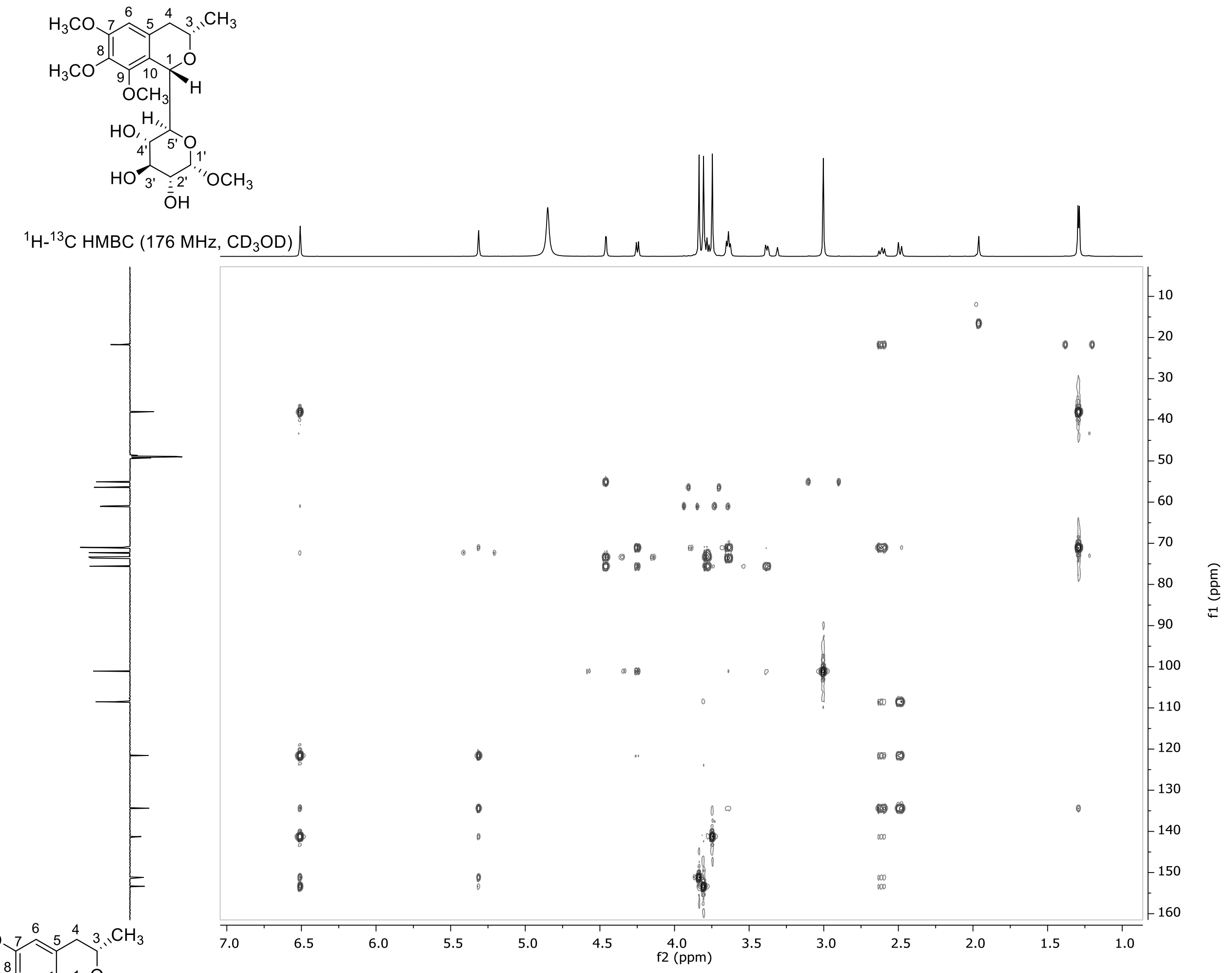
H3CO
H3CO
OCH3
HO
HO
OH
OCH3
CH3
H
H
O
O
1H-13C HMBC (176 MHz, CD3OD)
f2 (ppm)
f1 (ppm)

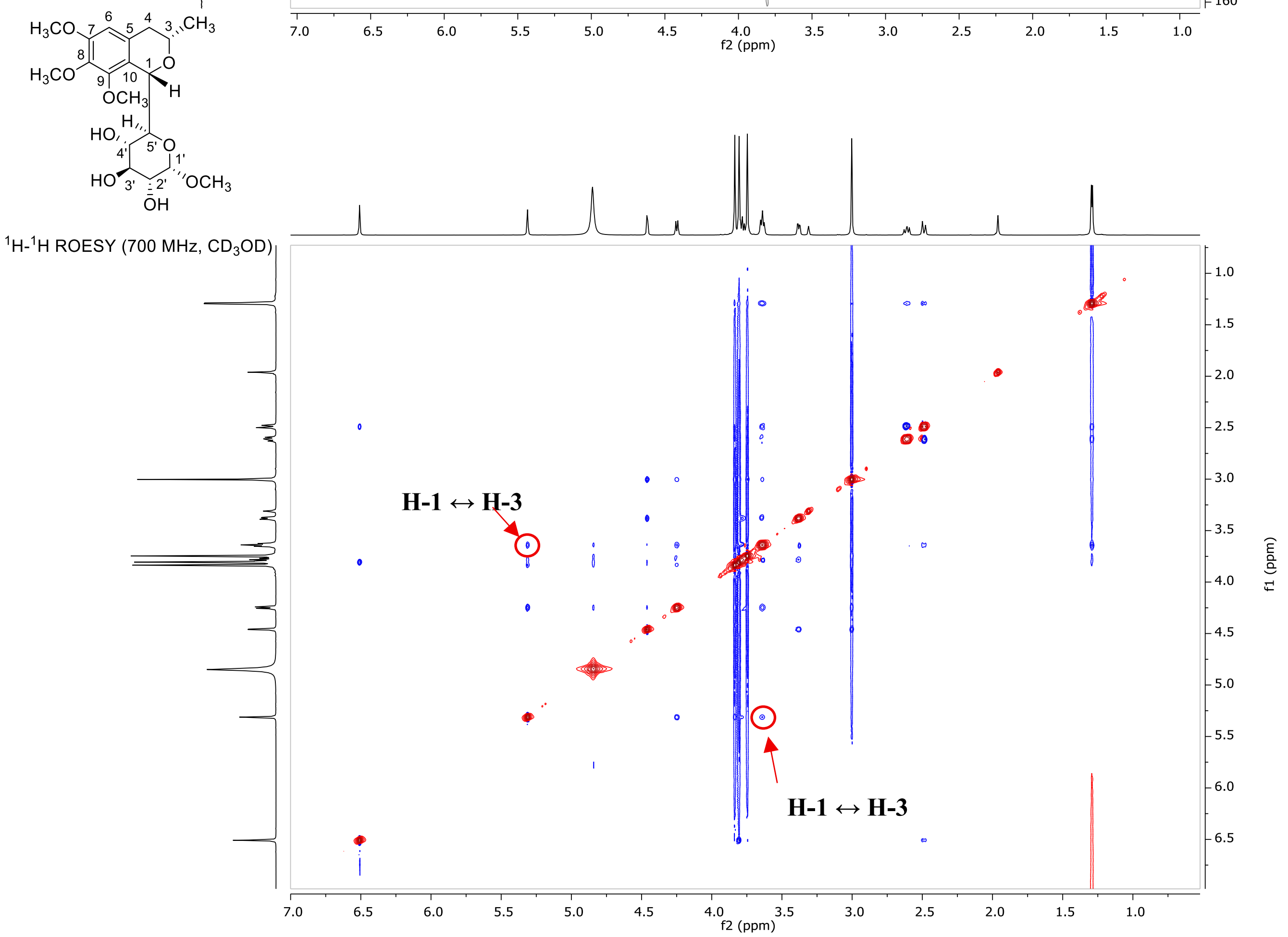
H3CO
H3CO
OCH3
HO
HO
OH
OCH3
CH3
H
H
O
O
1H-1H ROESY (700 MHz, CD3OD)
H-1 ↔ H-3
H-1 ↔ H-3
f2 (ppm)
f1 (ppm)

Compound 22

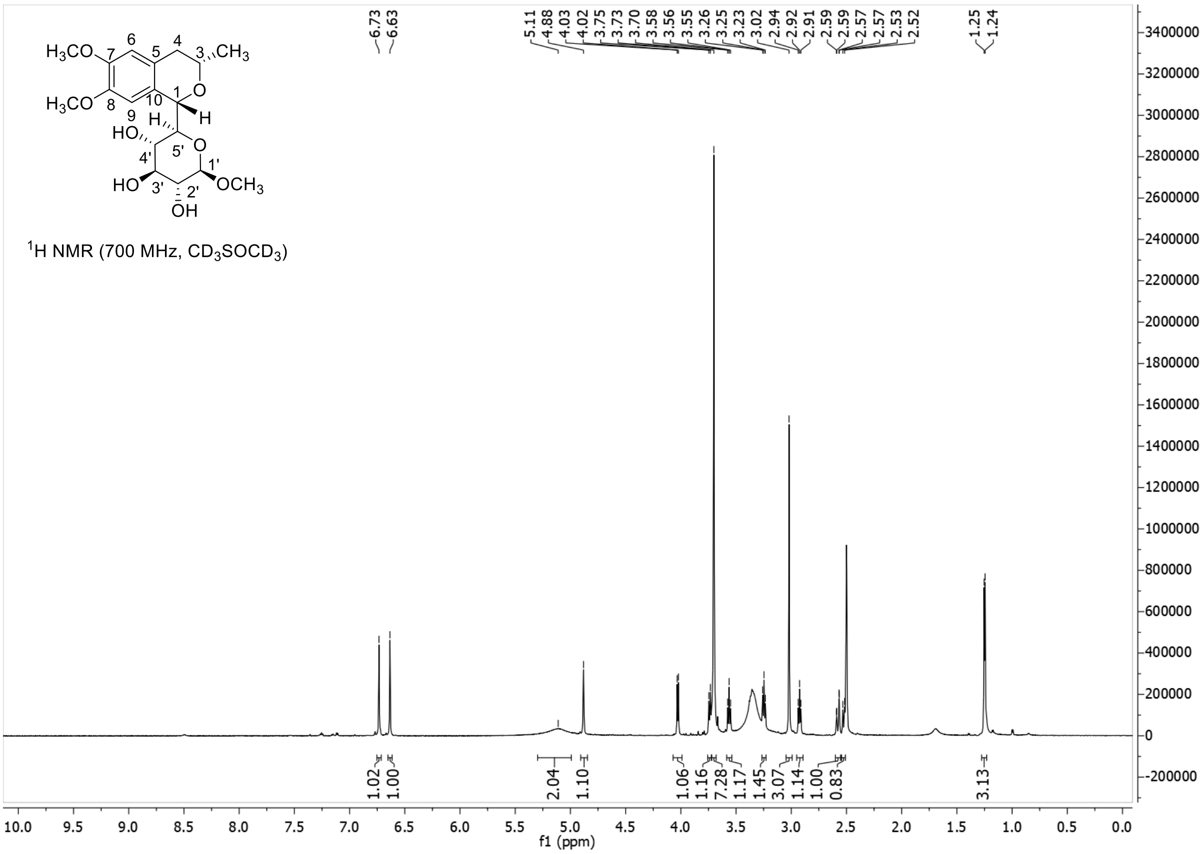

^{1}H NMR (700 MHz, CD_3SOCD_3)
6.73
6.63
5.11
4.88
4.03
4.02
3.75
3.73
3.70
3.58
3.56
3.55
3.26
3.25
3.23
3.02
2.94
2.92
2.91
2.59
2.59
2.57
2.57
2.53
2.52
1.25
1.24
1.02
1.00
2.04
1.10
1.06
1.16
7.28
1.17
1.45
3.07
1.14
1.00
0.83
3.13
f1 (ppm)

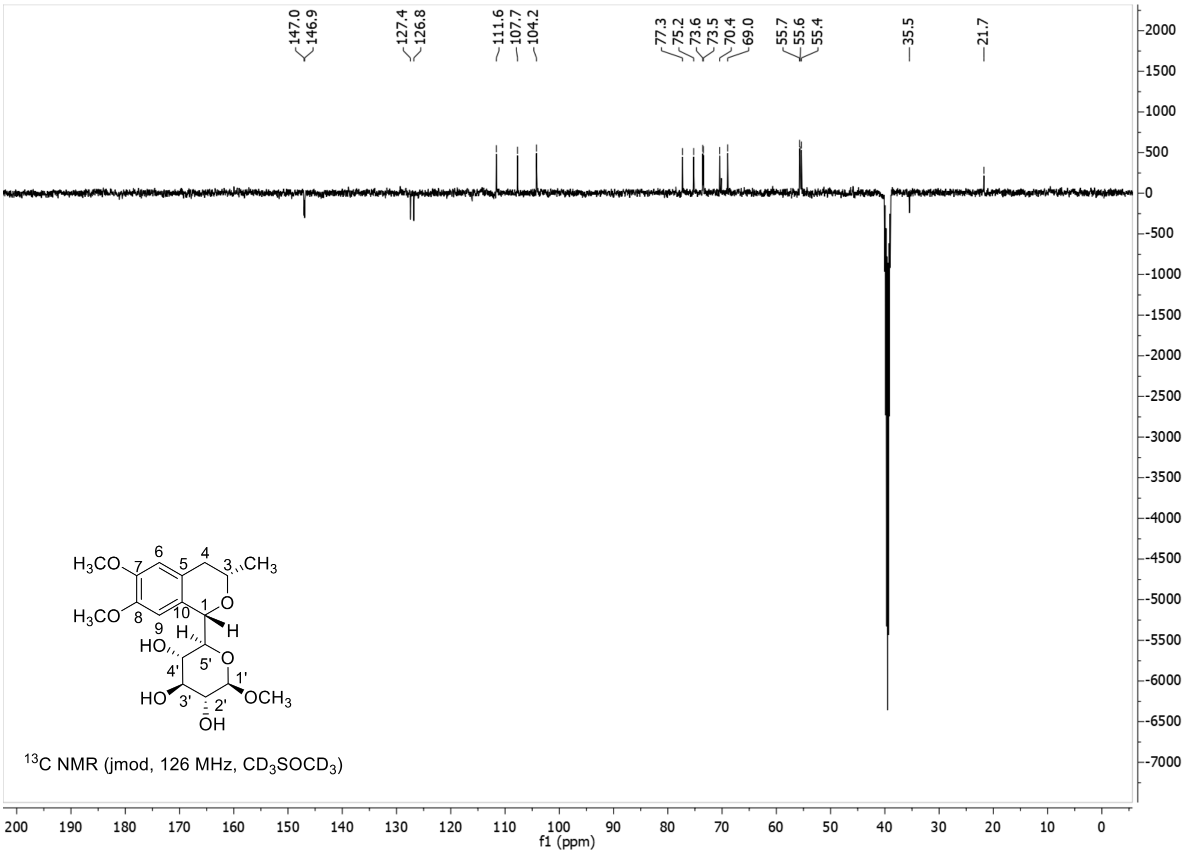

^{13}C NMR (jmod, 126 MHz, CD_3SOCD_3)
147.0
146.9
127.4
126.8
111.6
107.7
104.2
77.3
75.2
73.6
73.5
70.4
69.0
55.7
55.6
55.4
35.5
21.7
f1 (ppm)

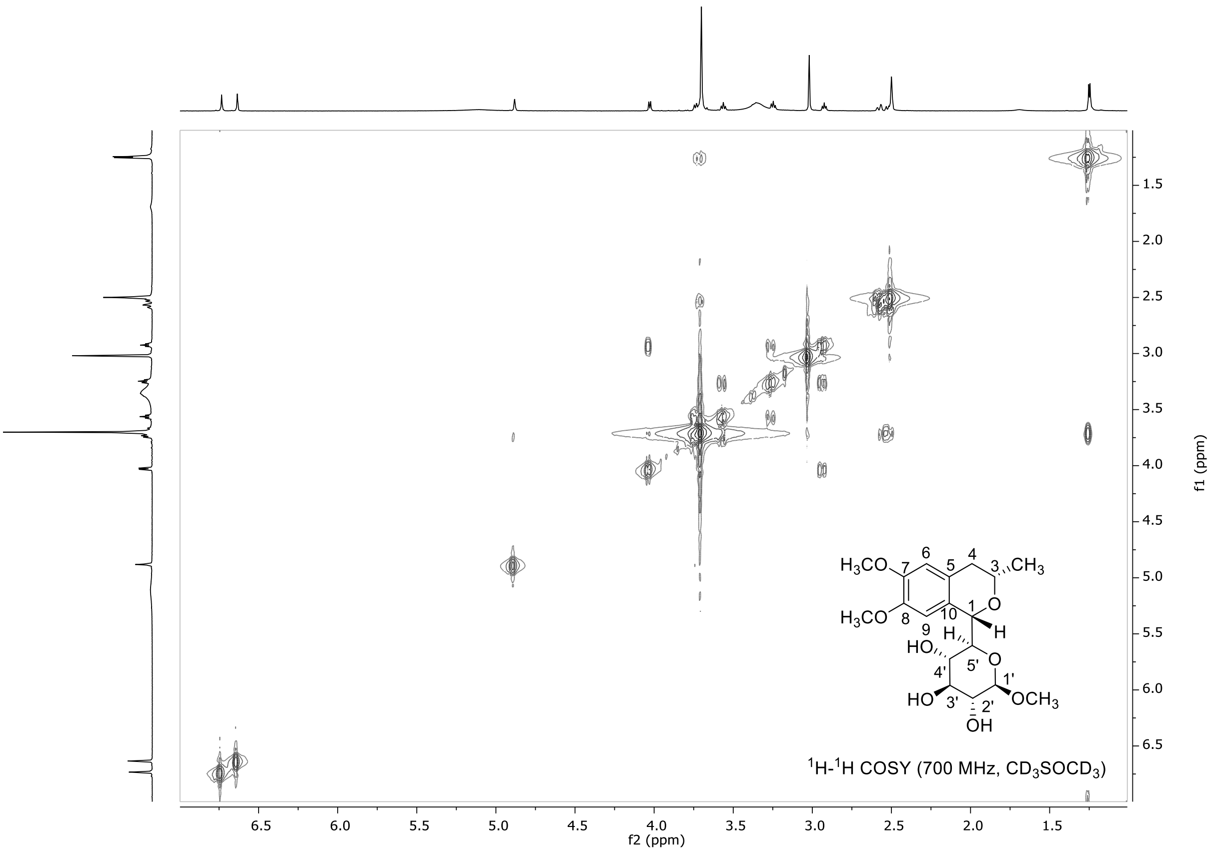

H3CO
H3CO
HO
HO
OH
OCH3
CH3
1H-1H COSY (700 MHz, CD3SOCD3)
f1 (ppm)
f2 (ppm)

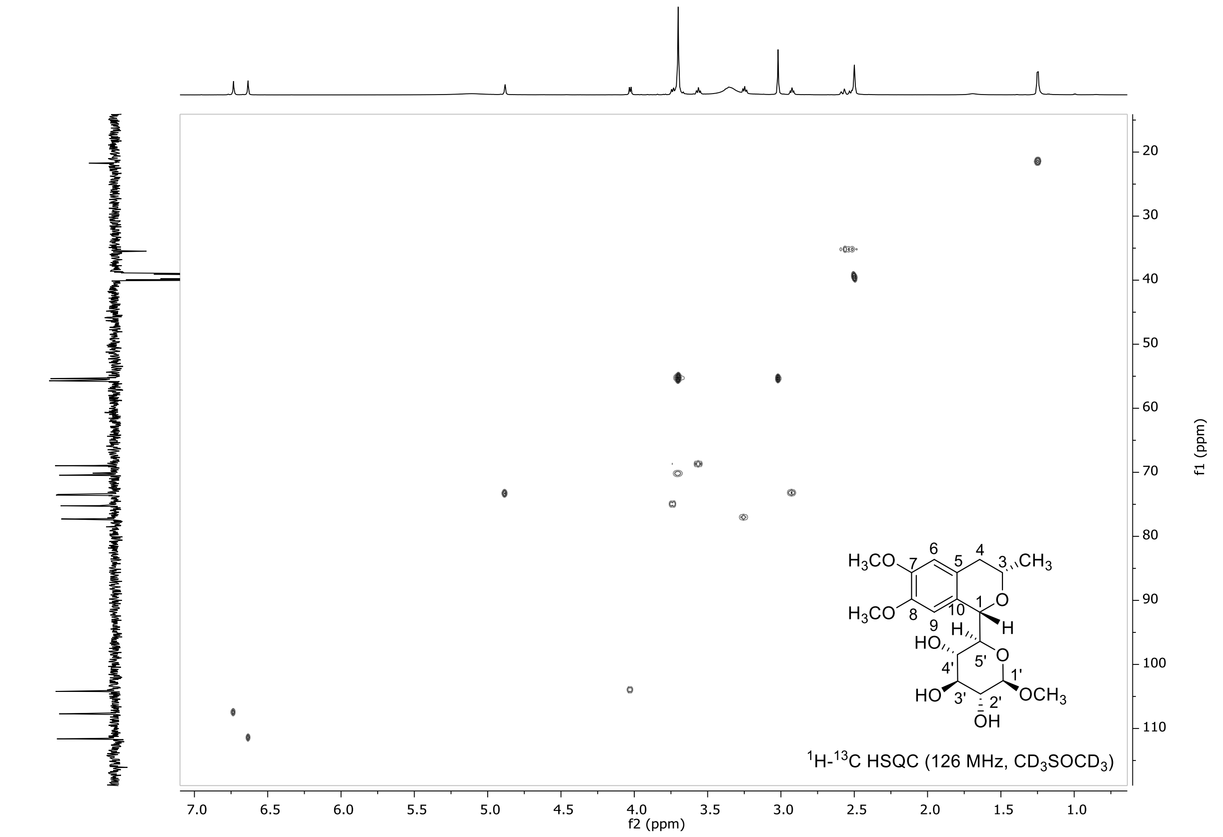

H3CO
H3CO
HO
HO
OH
OCH3
CH3
1H-13C HSQC (126 MHz, CD3SOCD3)
f1 (ppm)
f2 (ppm)

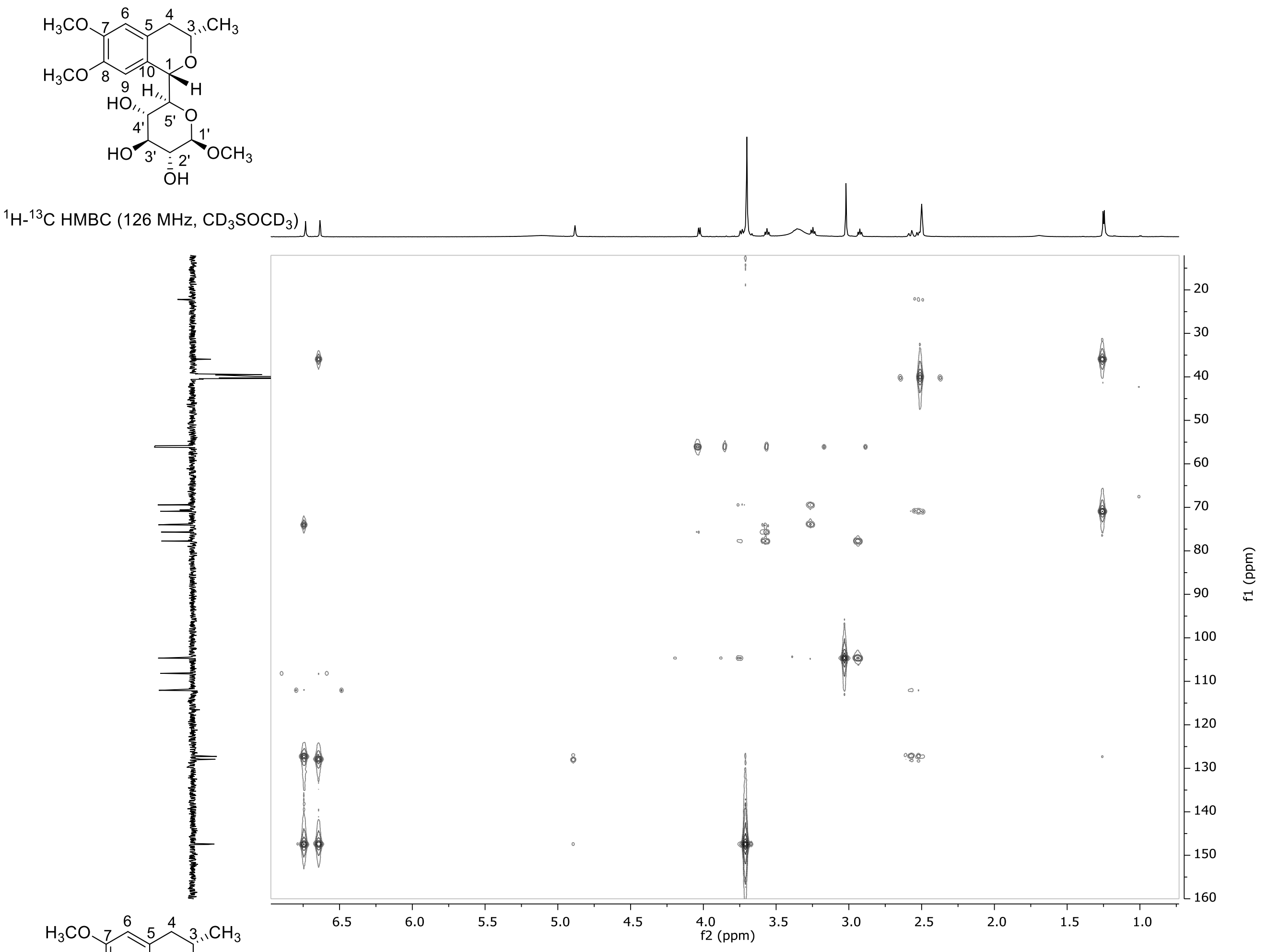

^{1}H-^{13}C HMBC (126 MHz, CD_3SOCD_3)
f2 (ppm)
f1 (ppm)

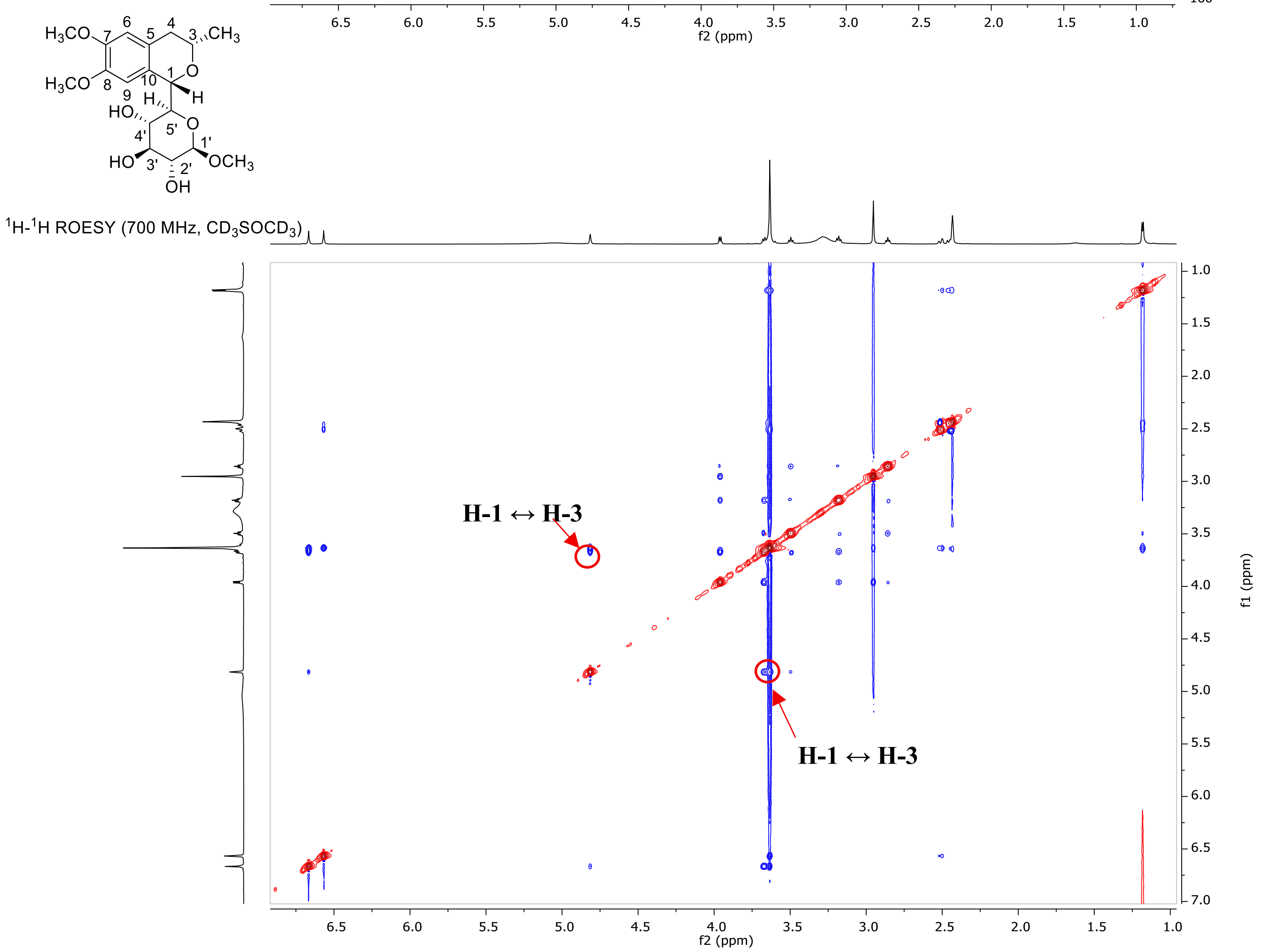

^{1}H-^{1}H ROESY (700 MHz, CD_3SOCD_3)
H-1 ↔ H-3
H-1 ↔ H-3
f2 (ppm)
f1 (ppm)

Compound 23

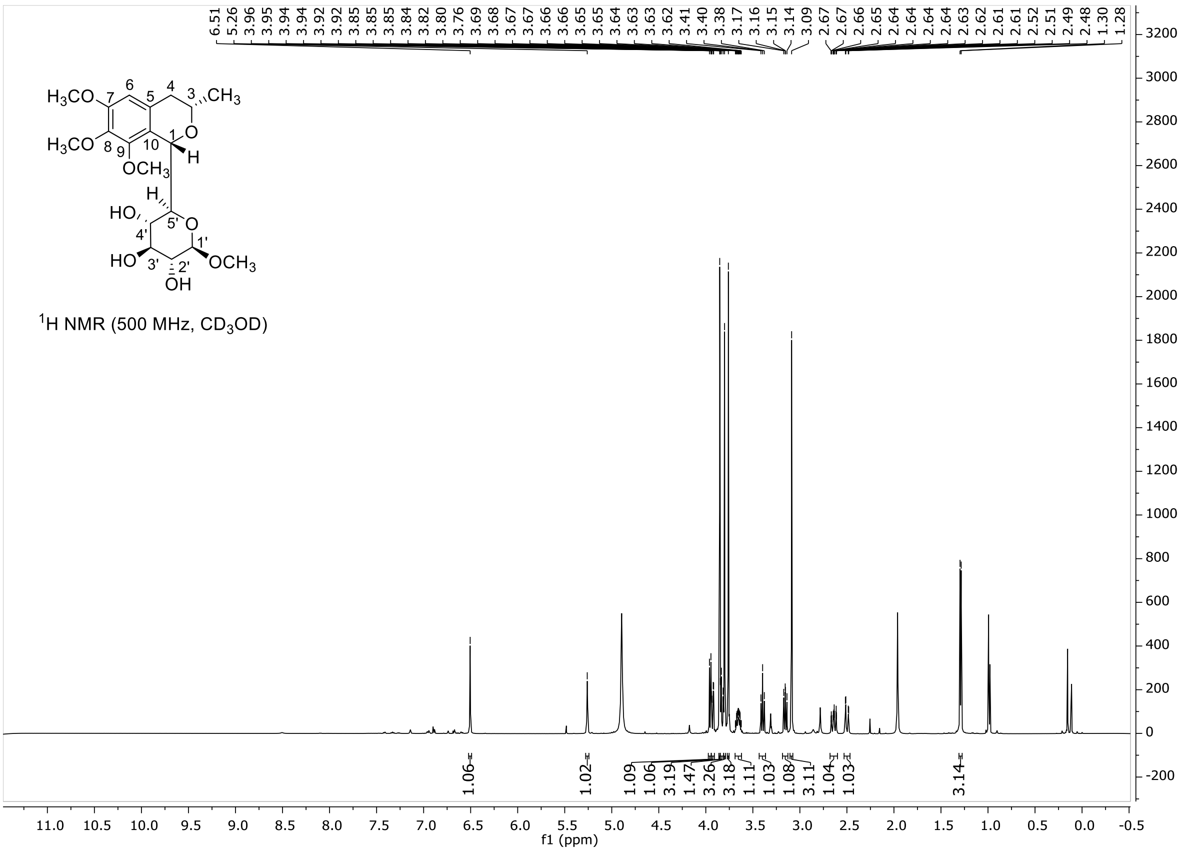

6.51 5.26 3.96 3.95 3.94 3.94 3.92 3.92 3.85 3.85 3.84 3.82 3.80 3.76 3.69 3.68 3.67 3.67 3.66 3.66 3.65 3.65 3.64 3.63 3.63 3.62 3.41 3.40 3.38 3.17 3.16 3.15 3.14 3.09 2.67 2.67 2.66 2.65 2.64 2.64 2.64 2.64 2.63 2.62 2.61 2.61 2.52 2.51 2.49 2.48 1.30 1.28
^{1}H NMR (500 MHz, CD$_3$OD)
1.06 1.02 1.09 1.06 3.19 1.47 3.26 3.18 1.11 1.03 1.08 3.11 1.04 1.03 3.14
f1 (ppm)

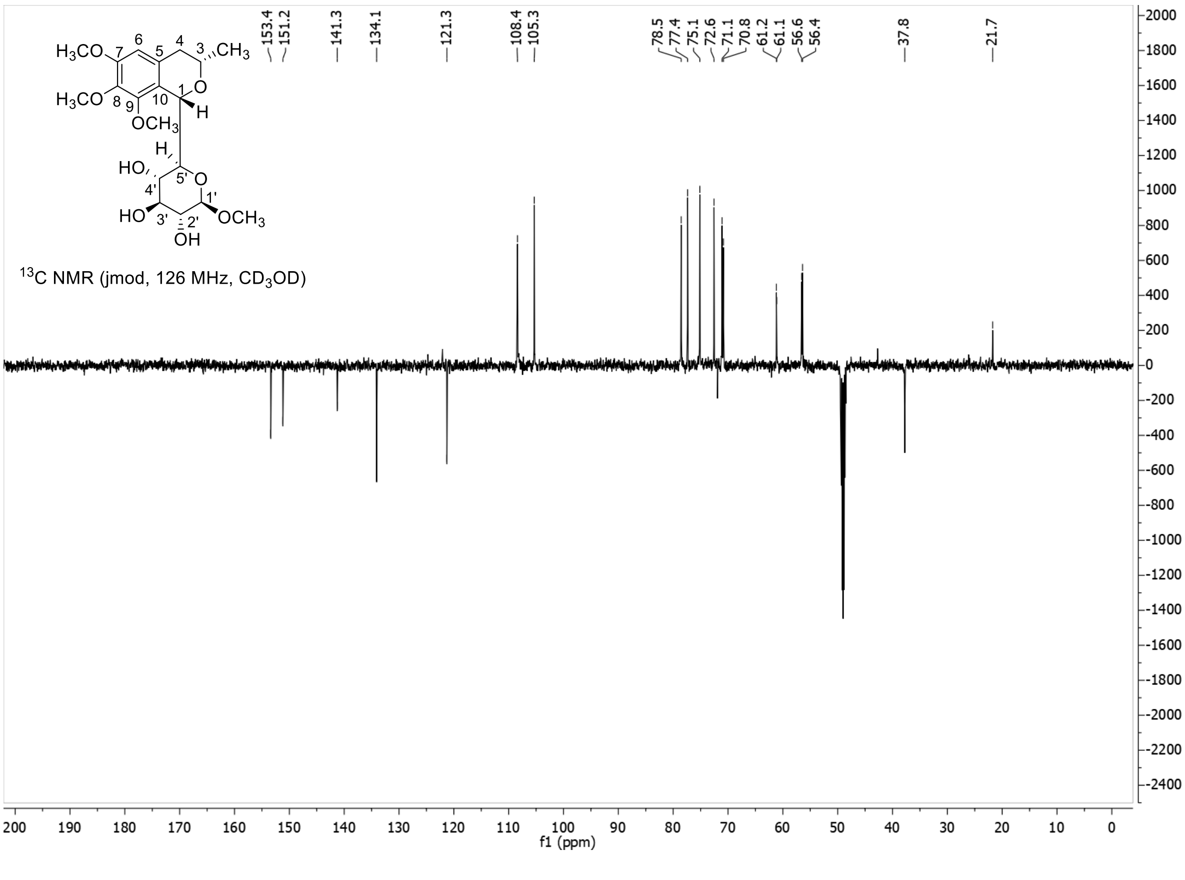

153.4 151.2 141.3 134.1 121.3 108.4 105.3 78.5 77.4 75.1 72.6 71.1 70.8 61.2 61.1 56.6 56.4 37.8 21.7
^{13}C NMR (jmod, 126 MHz, CD$_3$OD)
f1 (ppm)

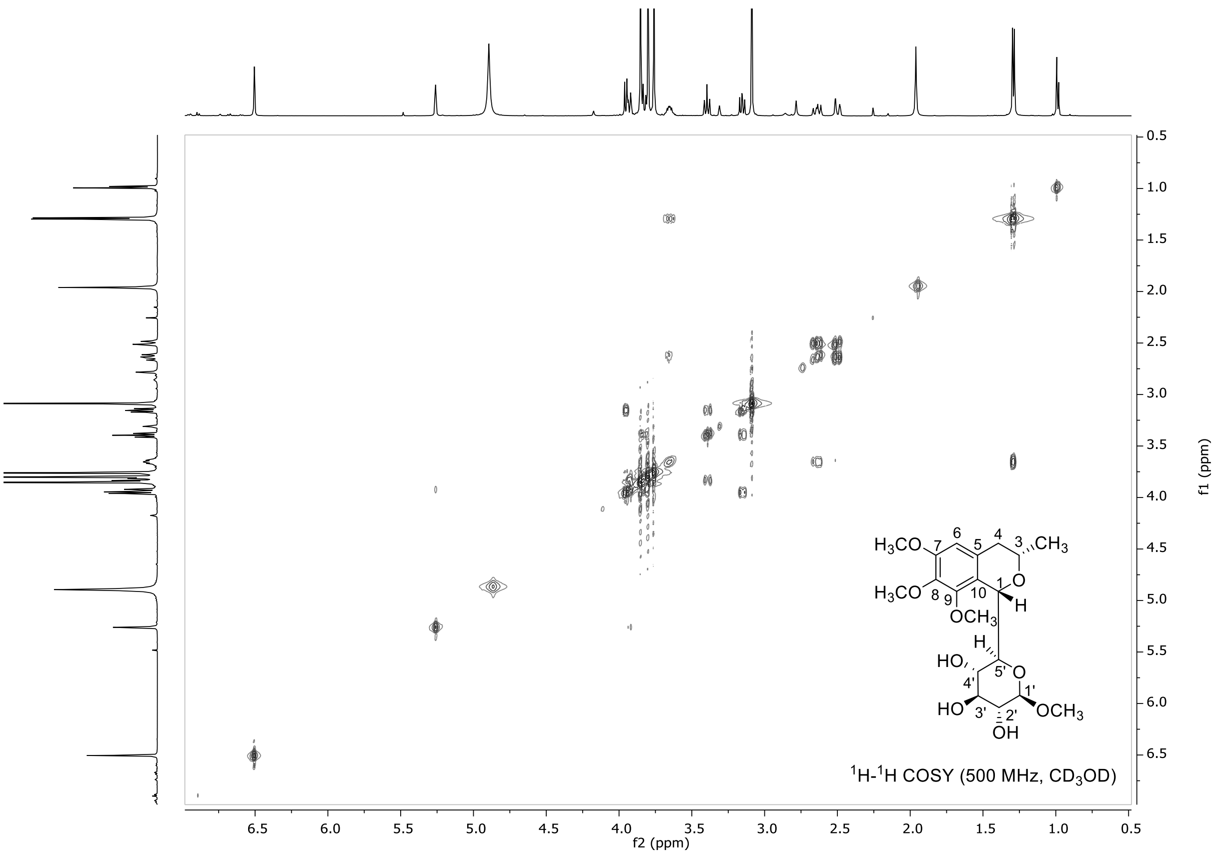

H3CO 7 6 5 4 3 CH3
O
H3CO 8 9 10 1 H
OCH3
H
HO 4' 5' O 1'
HO 3' 2' OCH3
OH
1H-1H COSY (500 MHz, CD3OD)
f2 (ppm)
f1 (ppm)

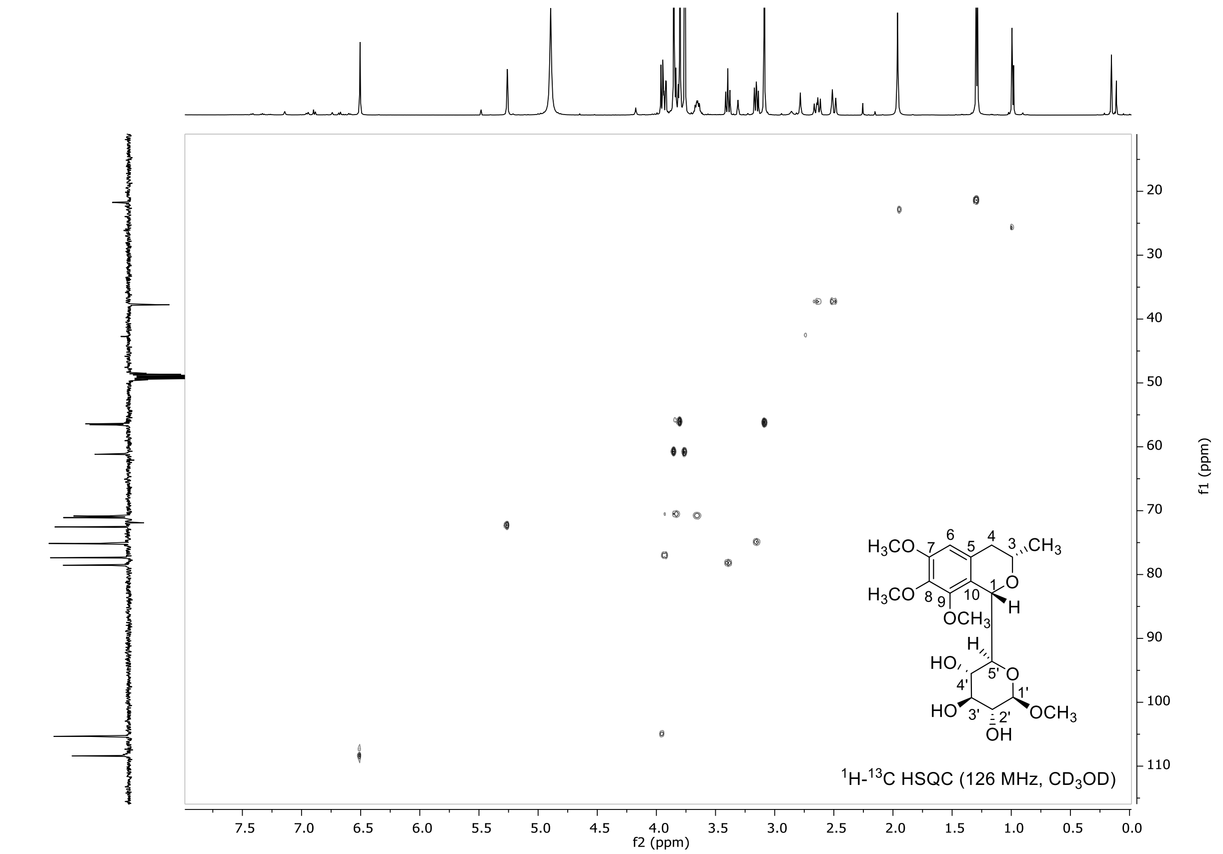

H3CO 7 6 5 4 3 CH3
O
H3CO 8 9 10 1 H
OCH3
H
HO 4' 5' O 1'
HO 3' 2' OCH3
OH
1H-13C HSQC (126 MHz, CD3OD)
f2 (ppm)
f1 (ppm)

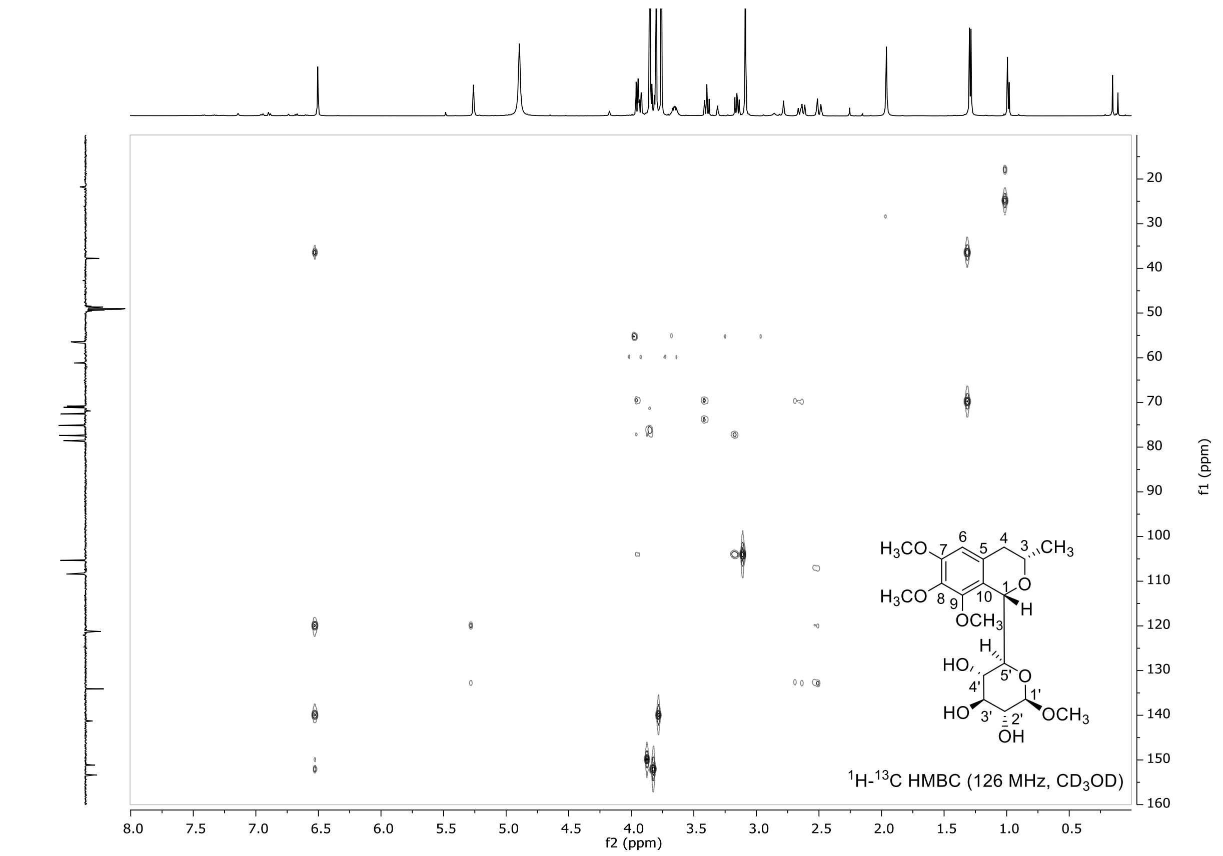
1H-13C HMBC (126 MHz, CD3OD)
f2 (ppm)
f1 (ppm)
H3CO
H3CO
OCH3
CH3
HO
HO
OH
OCH3

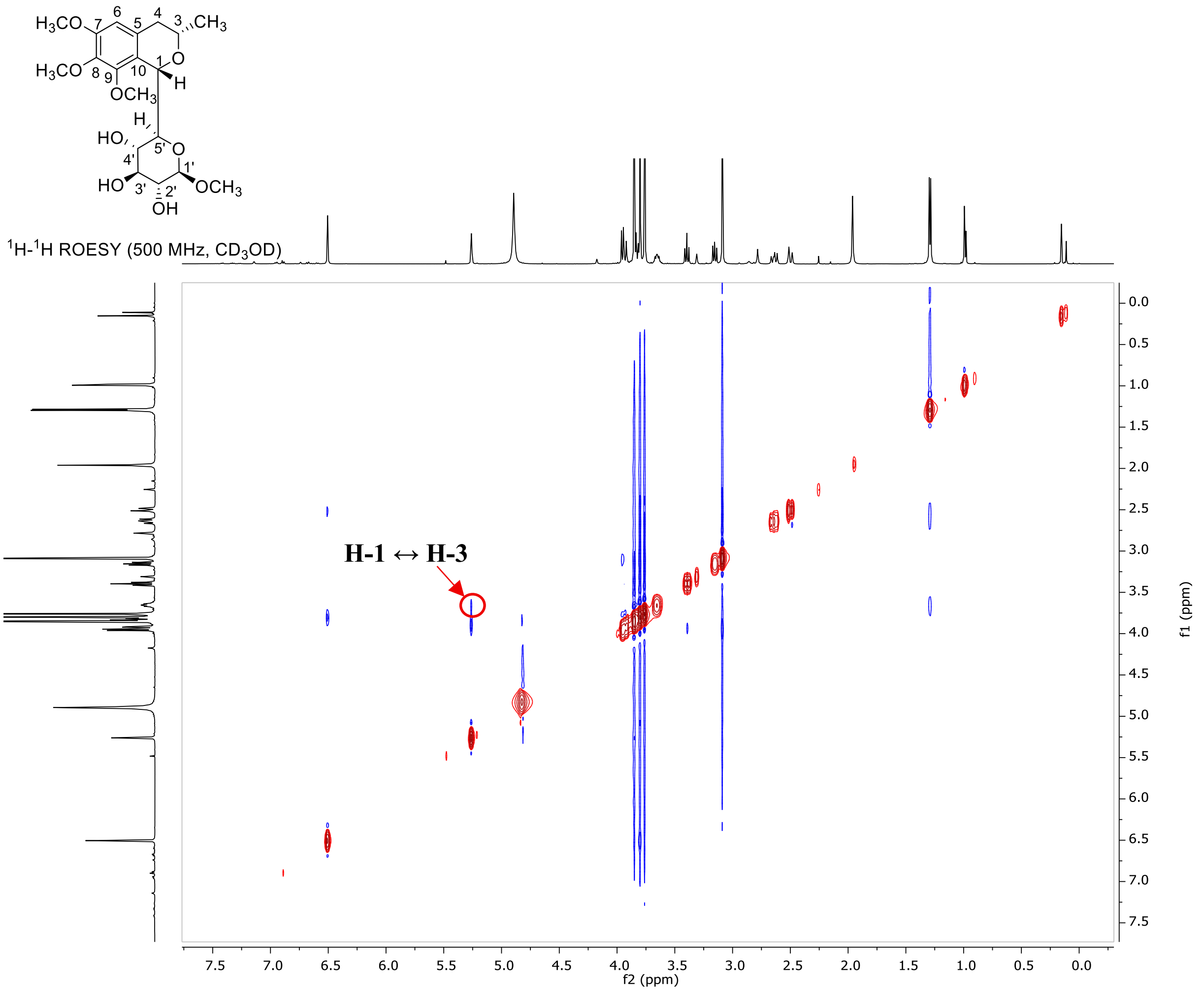
1H-1H ROESY (500 MHz, CD3OD)
H-1 ↔ H-3
f2 (ppm)
f1 (ppm)
H3CO
H3CO
OCH3
CH3
HO
HO
OH
OCH3

Compound 24

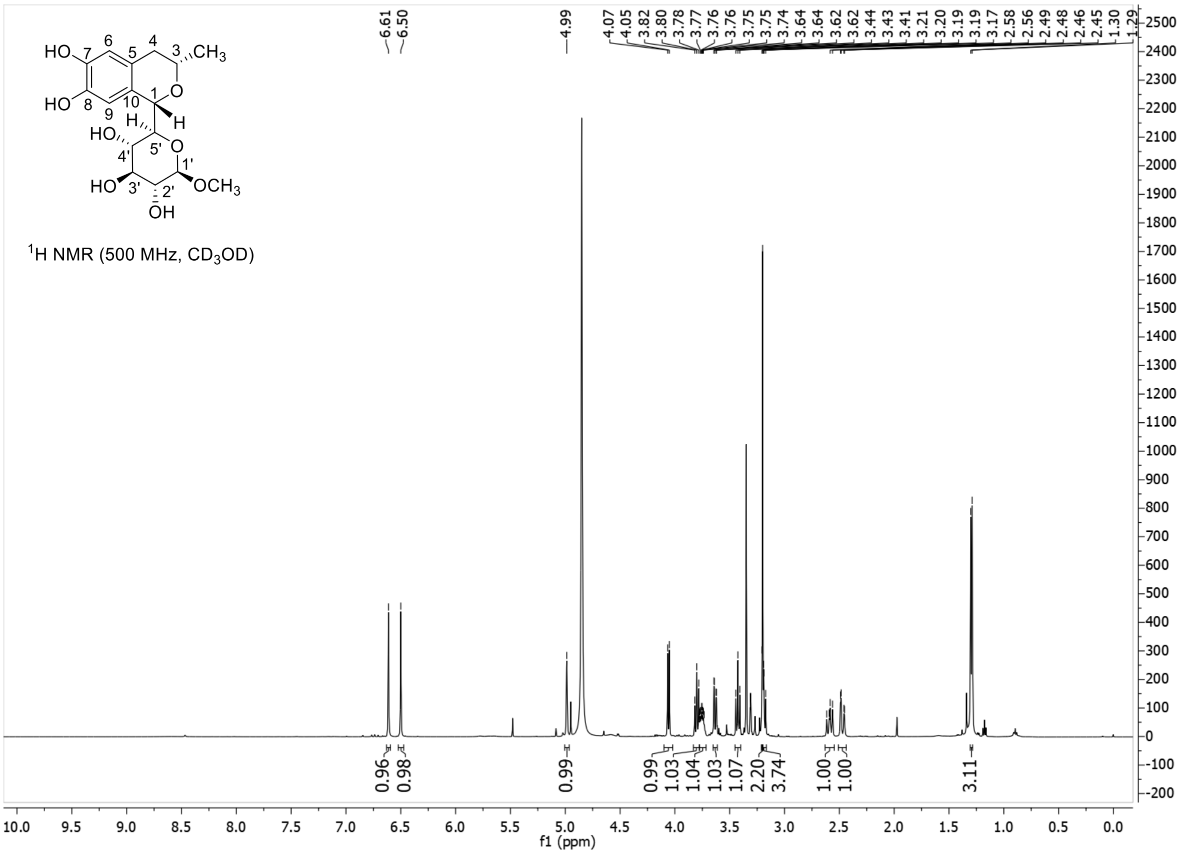

HO 7 6 5 4 3 CH3
HO 8 9 10 1 O H
H
HO 4' 5' O 1'
HO 3' 2' OCH3
OH
1H NMR (500 MHz, CD3OD)
6.61
6.50
4.99
4.07 4.05 3.82 3.80 3.78 3.77 3.76 3.76 3.75 3.75 3.74 3.64 3.64 3.62 3.62 3.44 3.43 3.41 3.21 3.20 3.19 3.19 3.17 2.58 2.56 2.49 2.48 2.46 2.45 1.30 1.29
0.96 0.98 0.99 0.99 1.03 1.04 1.03 1.07 2.20 3.74 1.00 1.00 3.11
f1 (ppm)

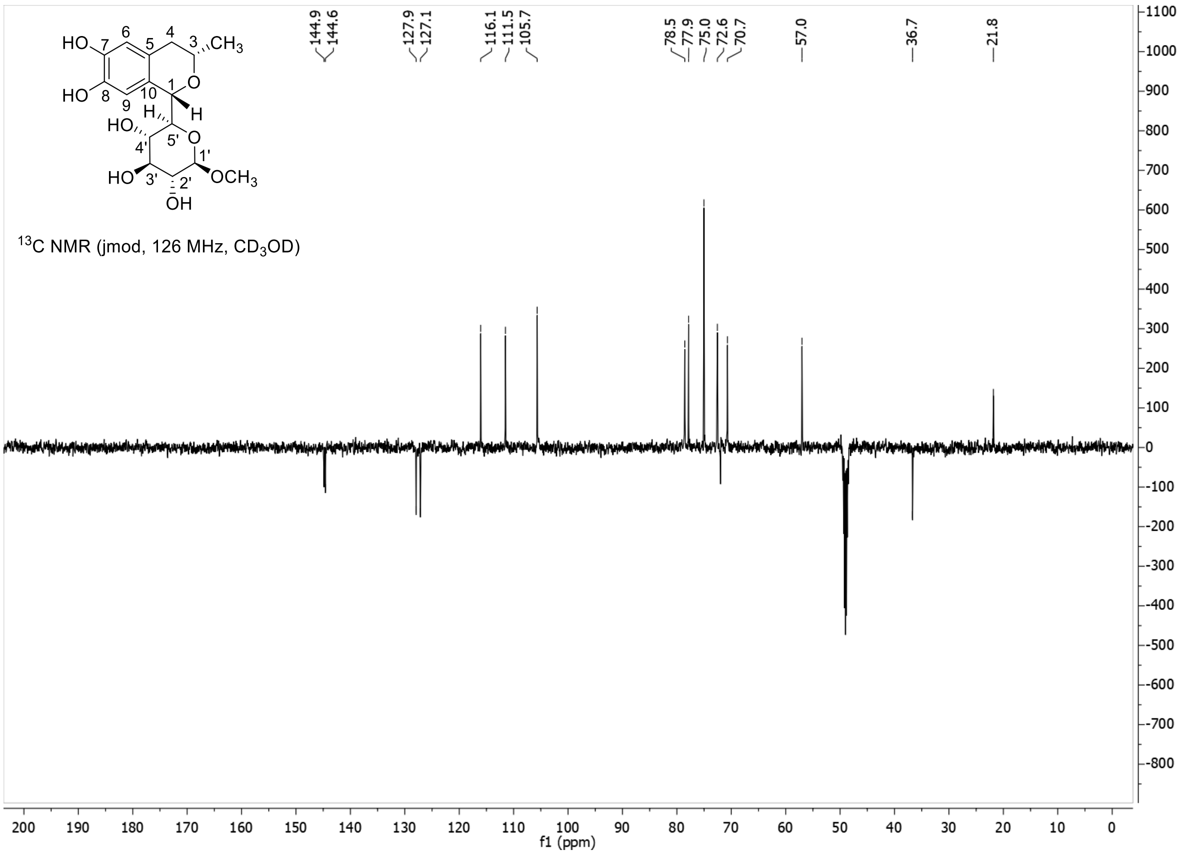

HO 7 6 5 4 3 CH3
HO 8 9 10 1 O H
H
HO 4' 5' O 1'
HO 3' 2' OCH3
OH
13C NMR (jmod, 126 MHz, CD3OD)
144.9 144.6
127.9 127.1
116.1 111.5 105.7
78.5 77.9 75.0 72.6 70.7
57.0
36.7
21.8
f1 (ppm)

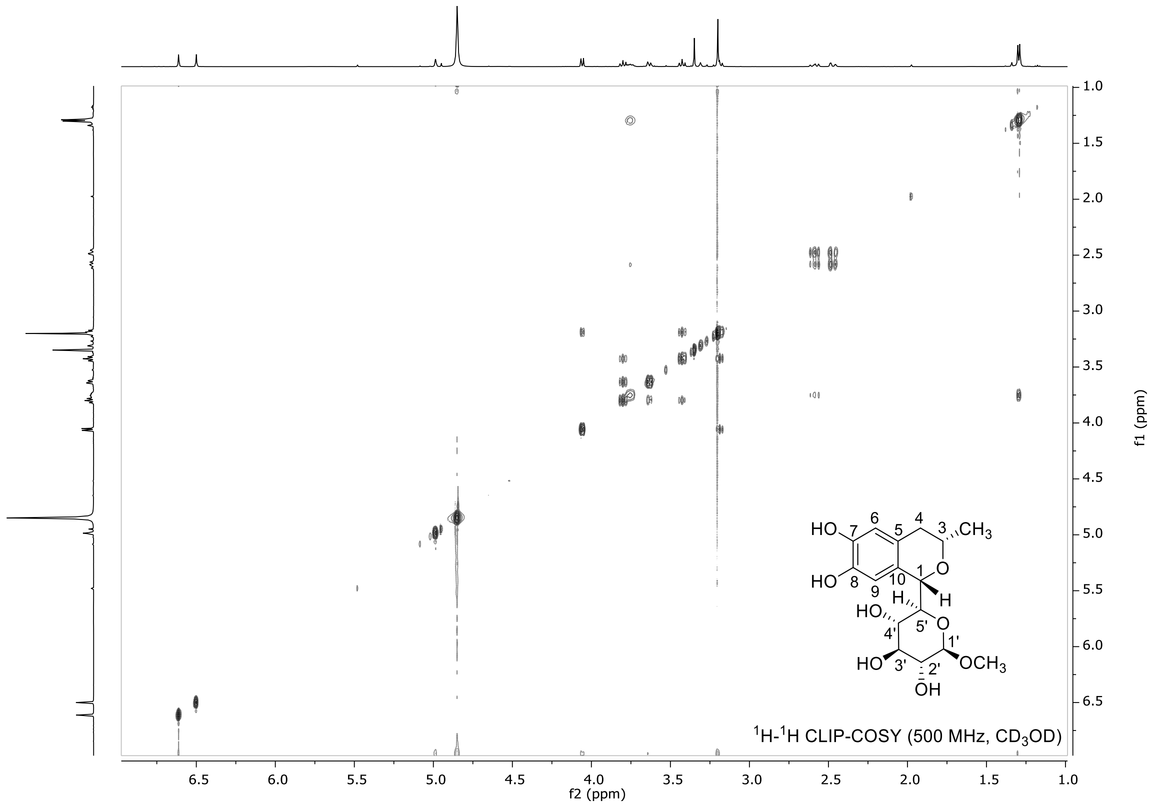

^{1}H-^{1}H CLIP-COSY (500 MHz, CD$_3$OD)
f2 (ppm)
f1 (ppm)

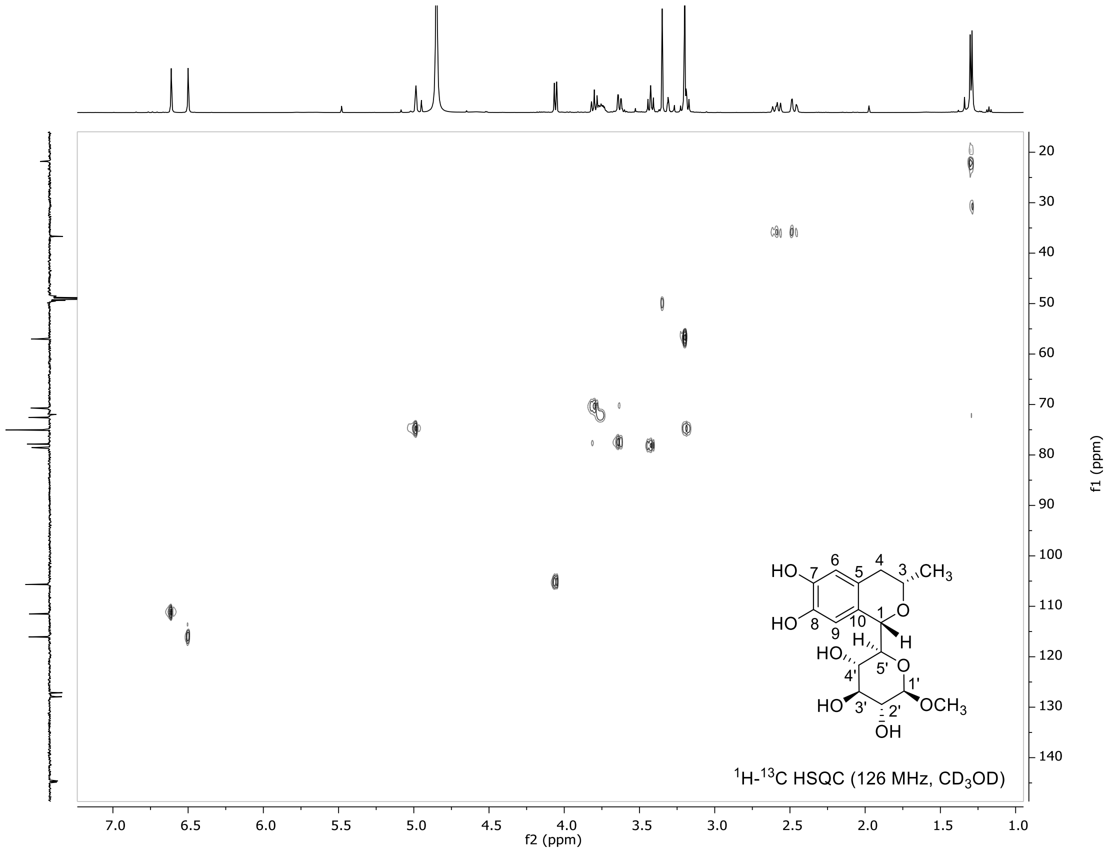

^{1}H-^{13}C HSQC (126 MHz, CD$_3$OD)
f2 (ppm)
f1 (ppm)

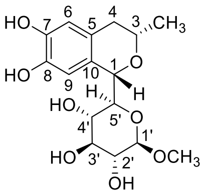

^1H-^{13}C HMBC (126 MHz, CD_3OD)

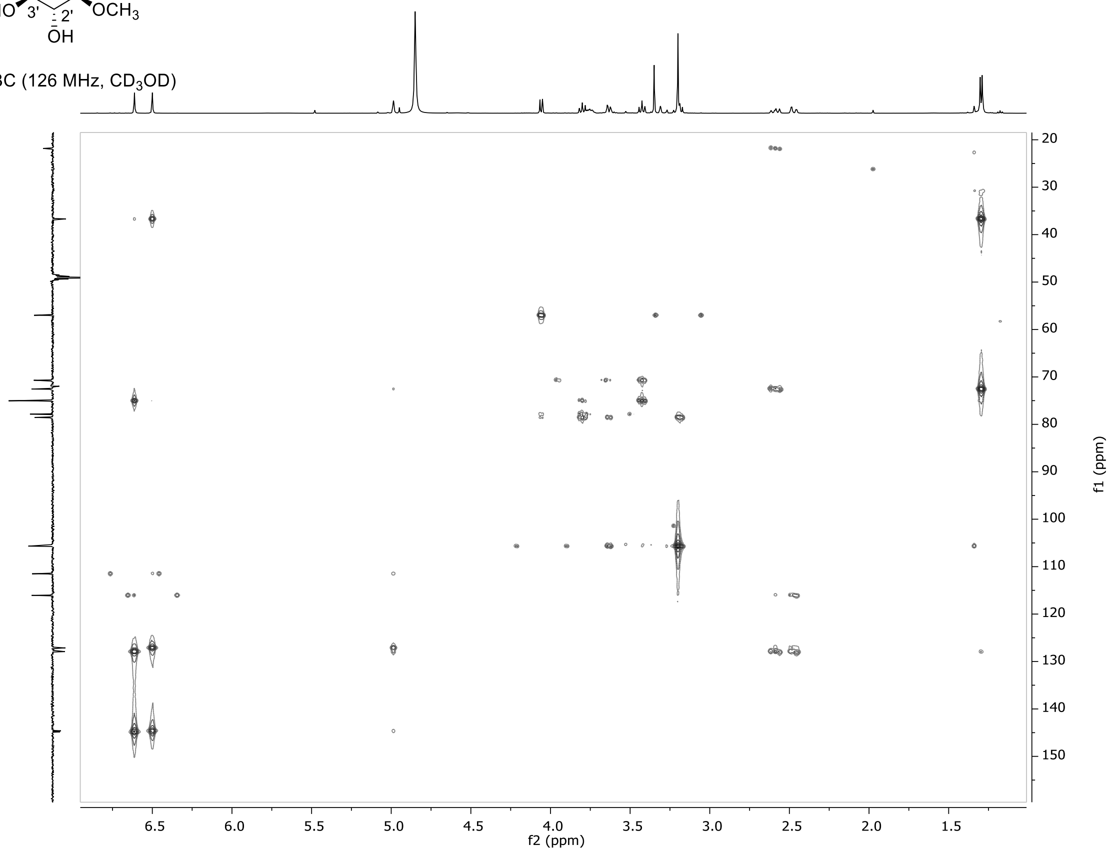

Compound 25

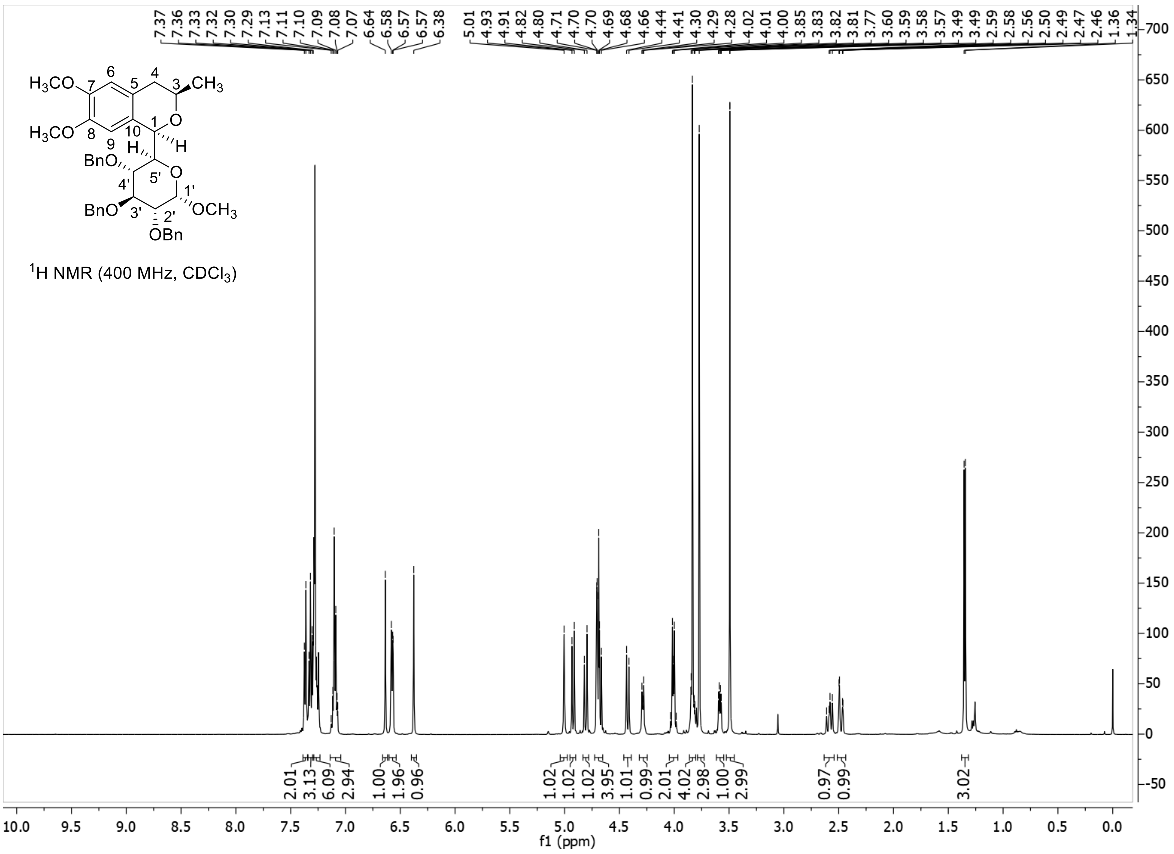

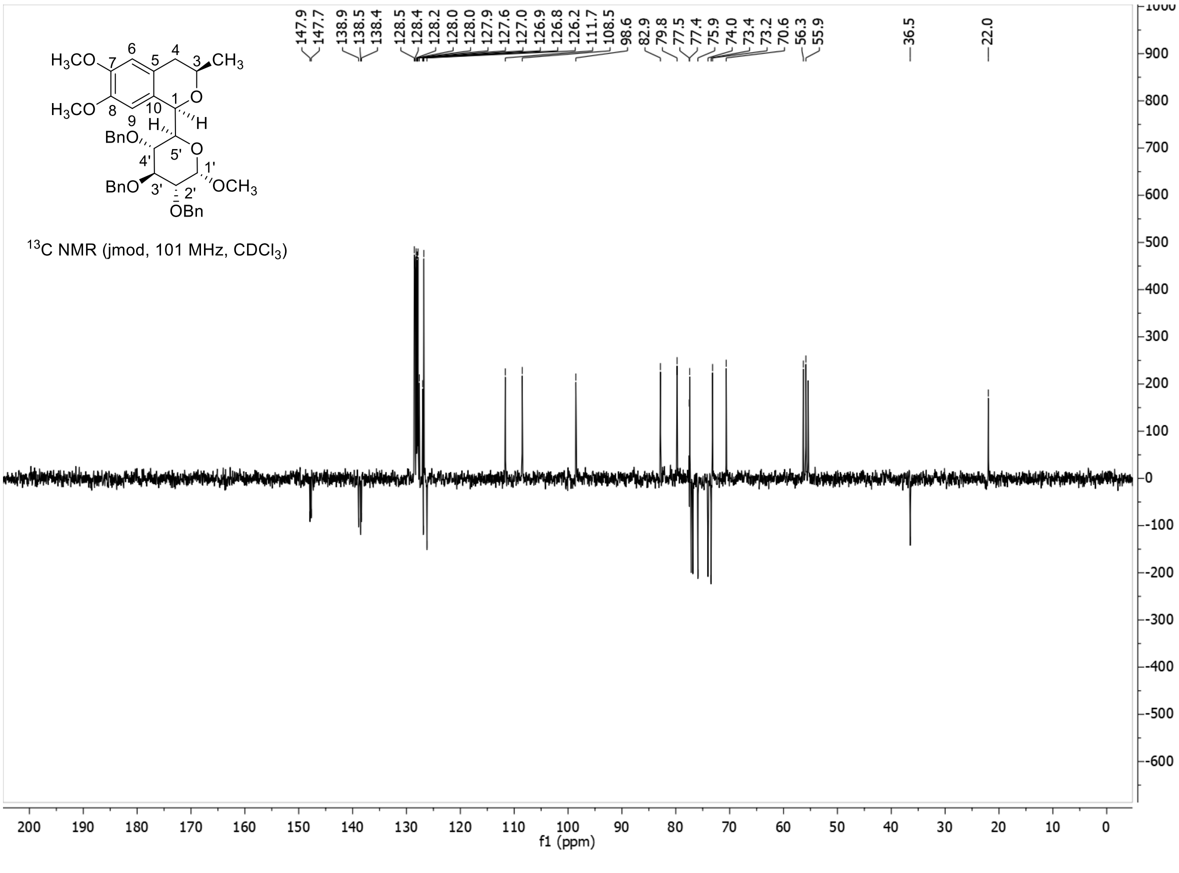

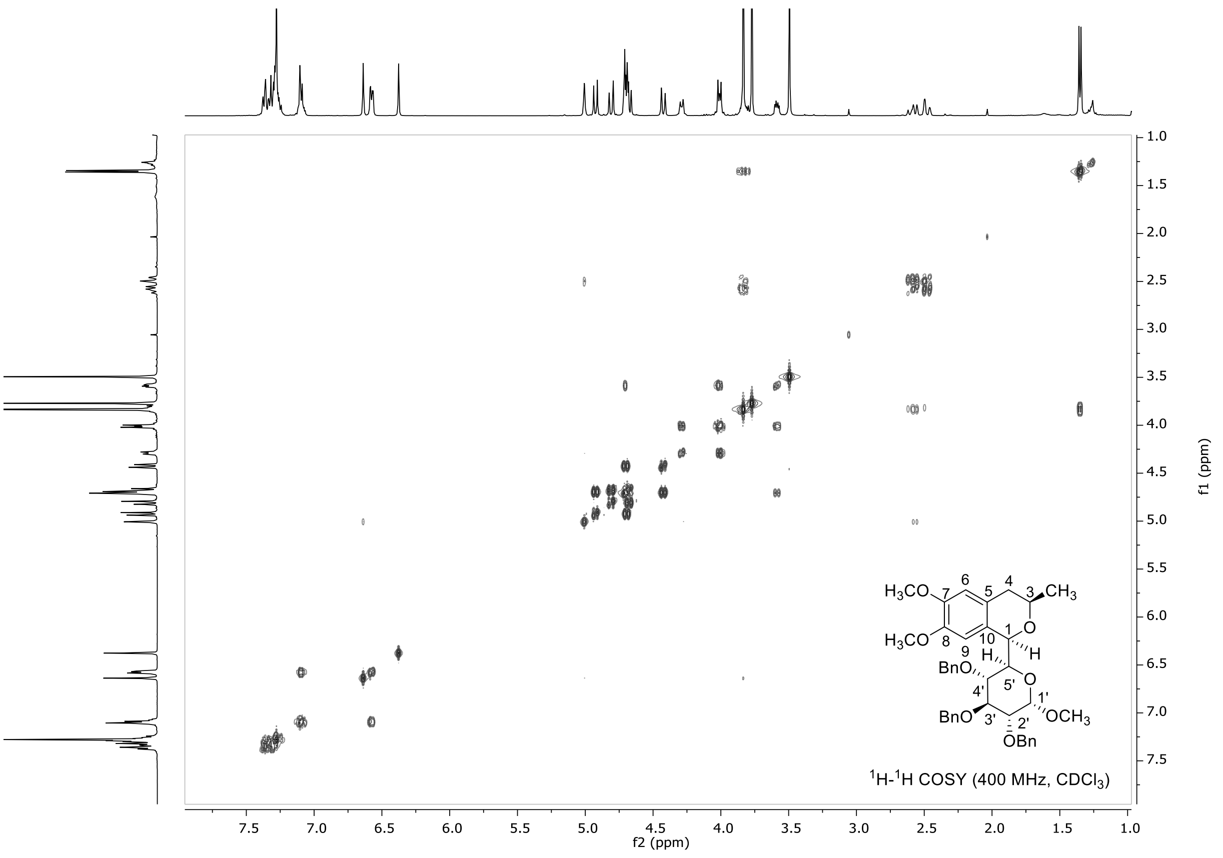
H3CO
H3CO
BnO
BnO
OBn
OCH3
CH3
1H-1H COSY (400 MHz, CDCl3)
f2 (ppm)
f1 (ppm)

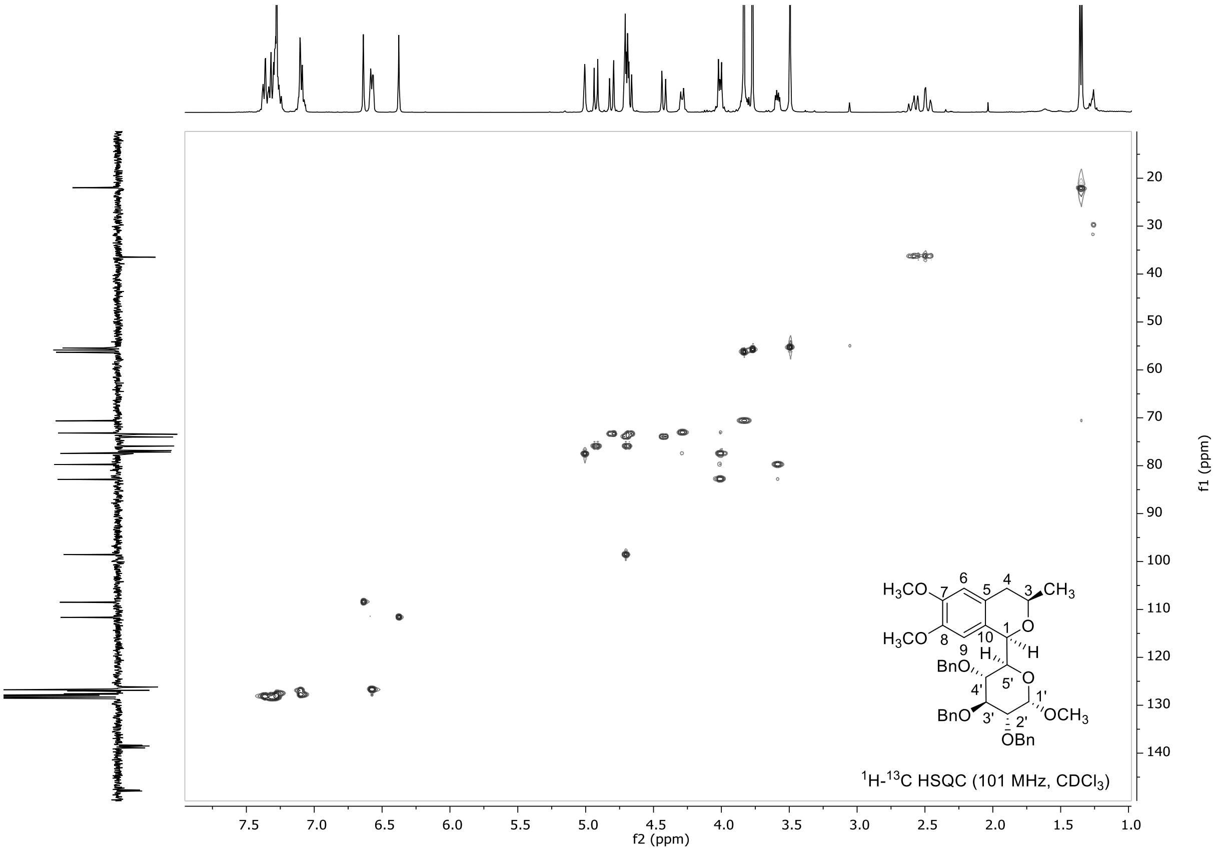
H3CO
H3CO
BnO
BnO
OBn
OCH3
CH3
1H-13C HSQC (101 MHz, CDCl3)
f2 (ppm)
f1 (ppm)

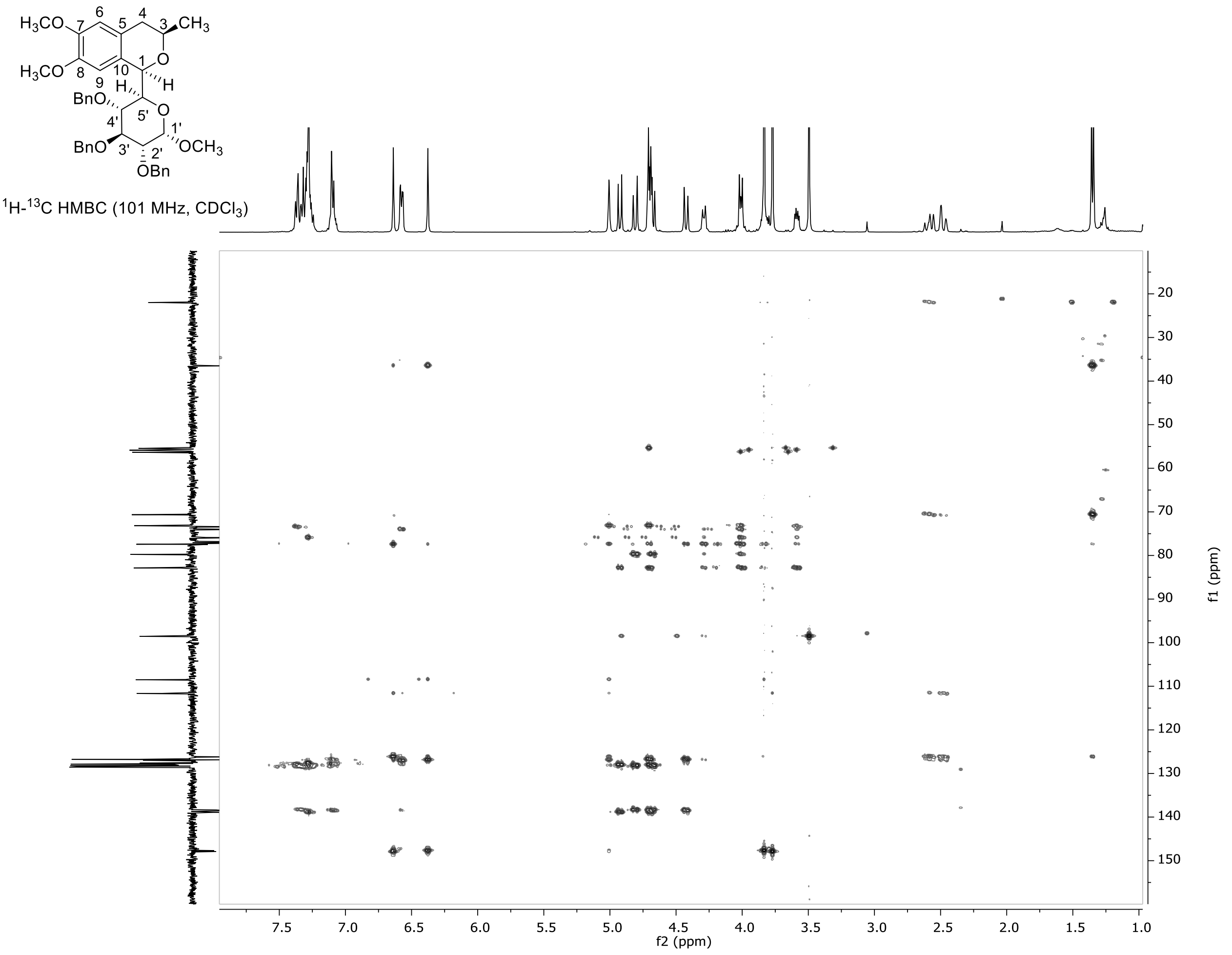
H3CO
H3CO
BnO
BnO
OBn
OCH3
CH3
1H-13C HMBC (101 MHz, CDCl3)
f2 (ppm)
f1 (ppm)

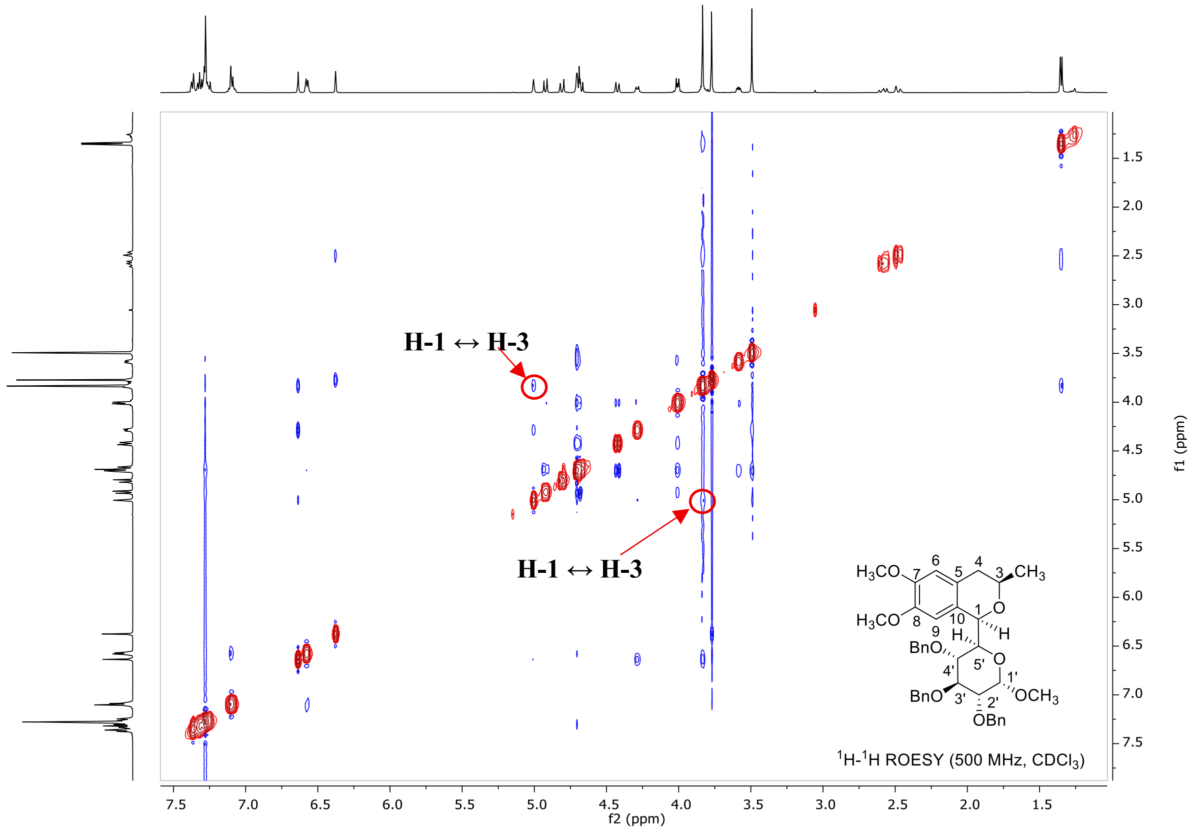
H-1 ↔ H-3
H-1 ↔ H-3
H3CO
H3CO
BnO
BnO
OBn
OCH3
CH3
1H-1H ROESY (500 MHz, CDCl3)
f2 (ppm)
f1 (ppm)

Compound 26

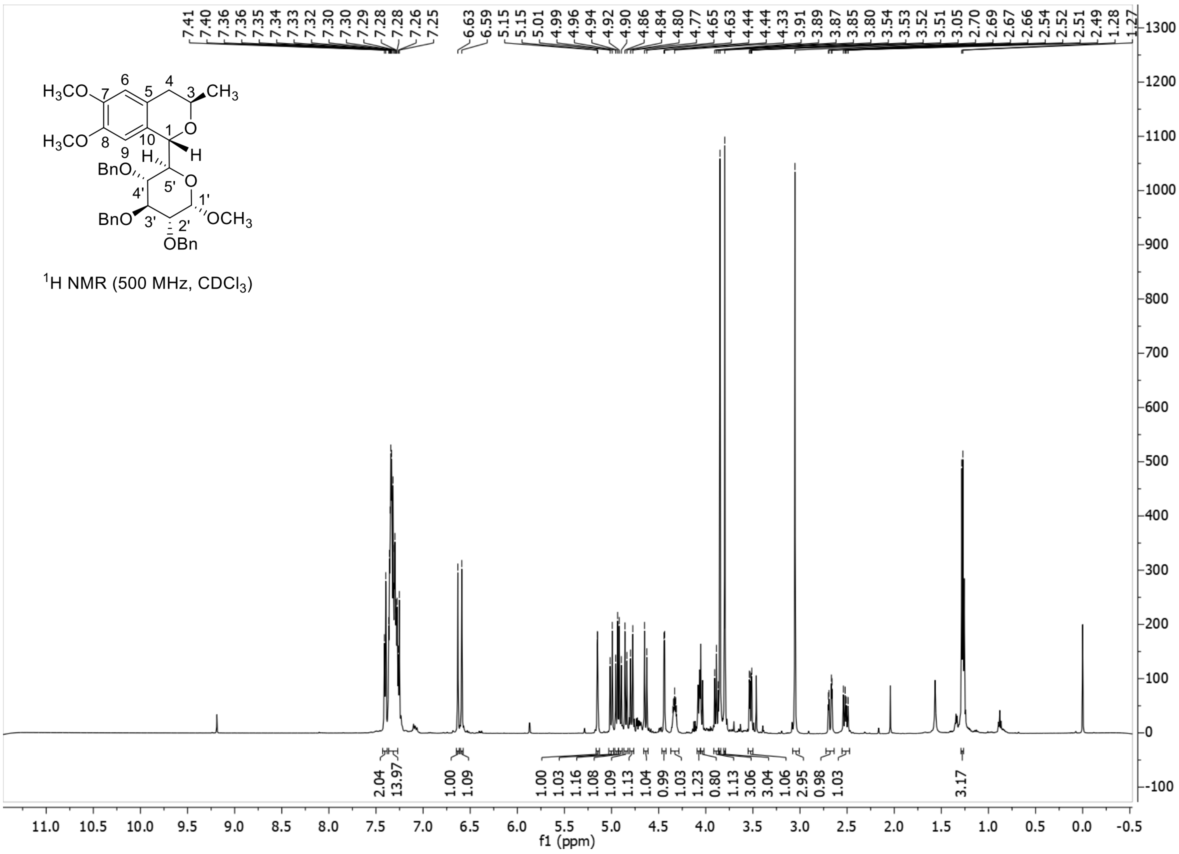

7.41 7.40 7.36 7.36 7.35 7.34 7.33 7.32 7.30 7.30 7.29 7.28 7.28 7.26 7.25 6.63 6.59 5.15 5.15 5.01 4.99 4.96 4.94 4.92 4.90 4.86 4.84 4.80 4.77 4.65 4.63 4.44 4.44 4.33 3.91 3.89 3.87 3.85 3.80 3.54 3.53 3.52 3.51 3.05 2.70 2.69 2.67 2.66 2.54 2.52 2.51 2.49 1.28 1.27
H_3CO H_3CO 6 7 8 9 10 5 4 3 1 CH_3 O H H BnO 4' 5' O 1' BnO 3' 2' OCH_3 OBn
^1H NMR (500 MHz, $CDCl_3$)
2.04 13.97 1.00 1.09 1.00 1.03 1.16 1.08 1.09 1.13 1.04 0.99 1.03 1.23 0.80 1.13 3.06 3.04 1.06 2.95 0.98 1.03 3.17
f1 (ppm)

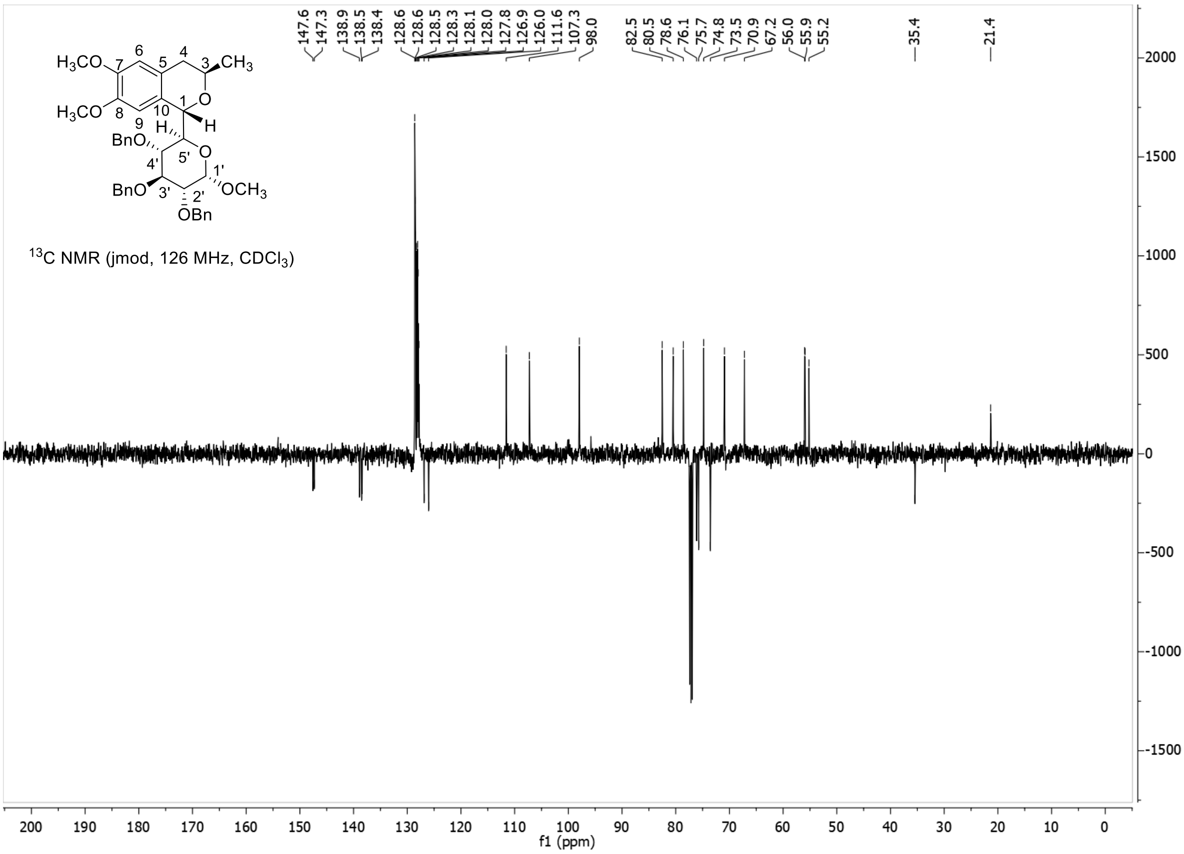

147.6 147.3 138.9 138.5 138.4 128.6 128.6 128.5 128.3 128.1 128.0 127.8 126.9 126.0 111.6 107.3 98.0 82.5 80.5 78.6 76.1 75.7 74.8 73.5 70.9 67.2 56.0 55.9 55.2 35.4 21.4
H_3CO H_3CO 6 7 8 9 10 5 4 3 1 CH_3 O H H BnO 4' 5' O 1' BnO 3' 2' OCH_3 OBn
^{13}C NMR (jmod, 126 MHz, $CDCl_3$)
f1 (ppm)

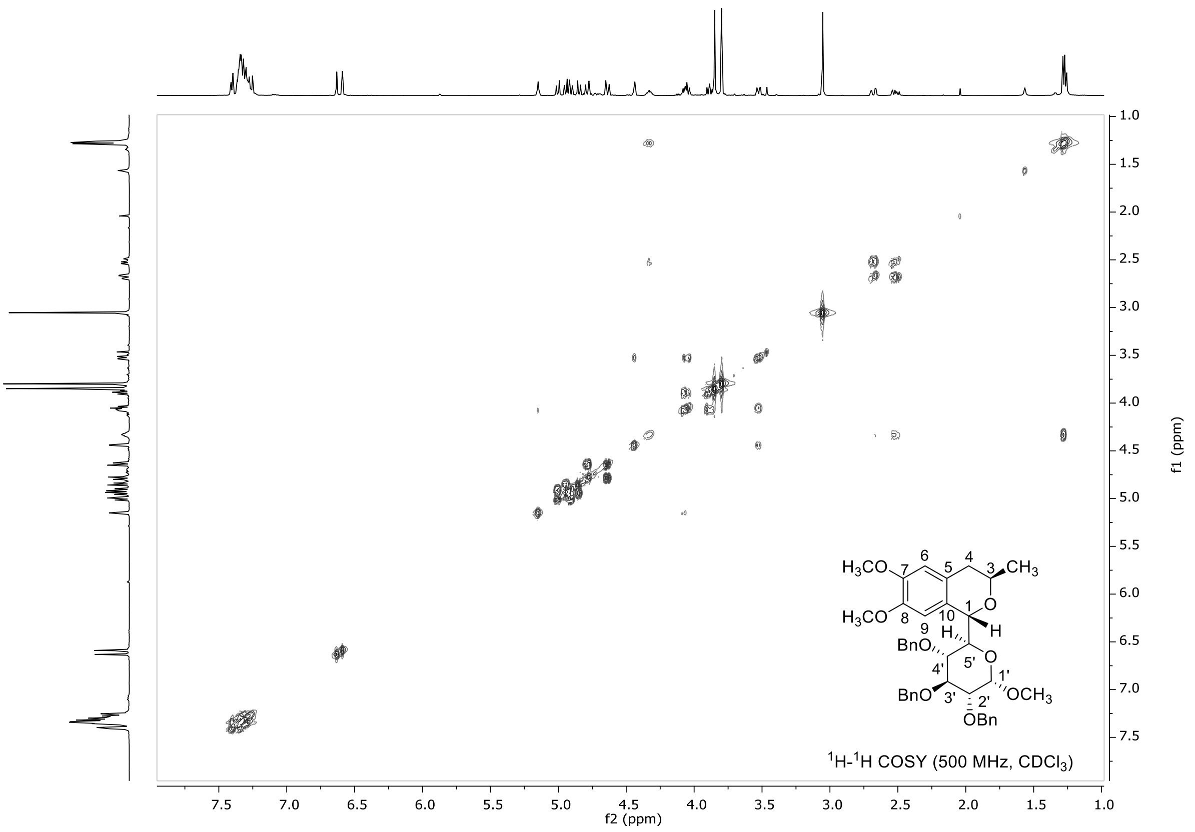

H3CO 7 6 5 4 3 CH3
H3CO 8 9 10 1 O H
H
BnO 4' 5' O 1'
BnO 3' 2' OCH3
OBn
1H-1H COSY (500 MHz, CDCl3)
f1 (ppm)
f2 (ppm)

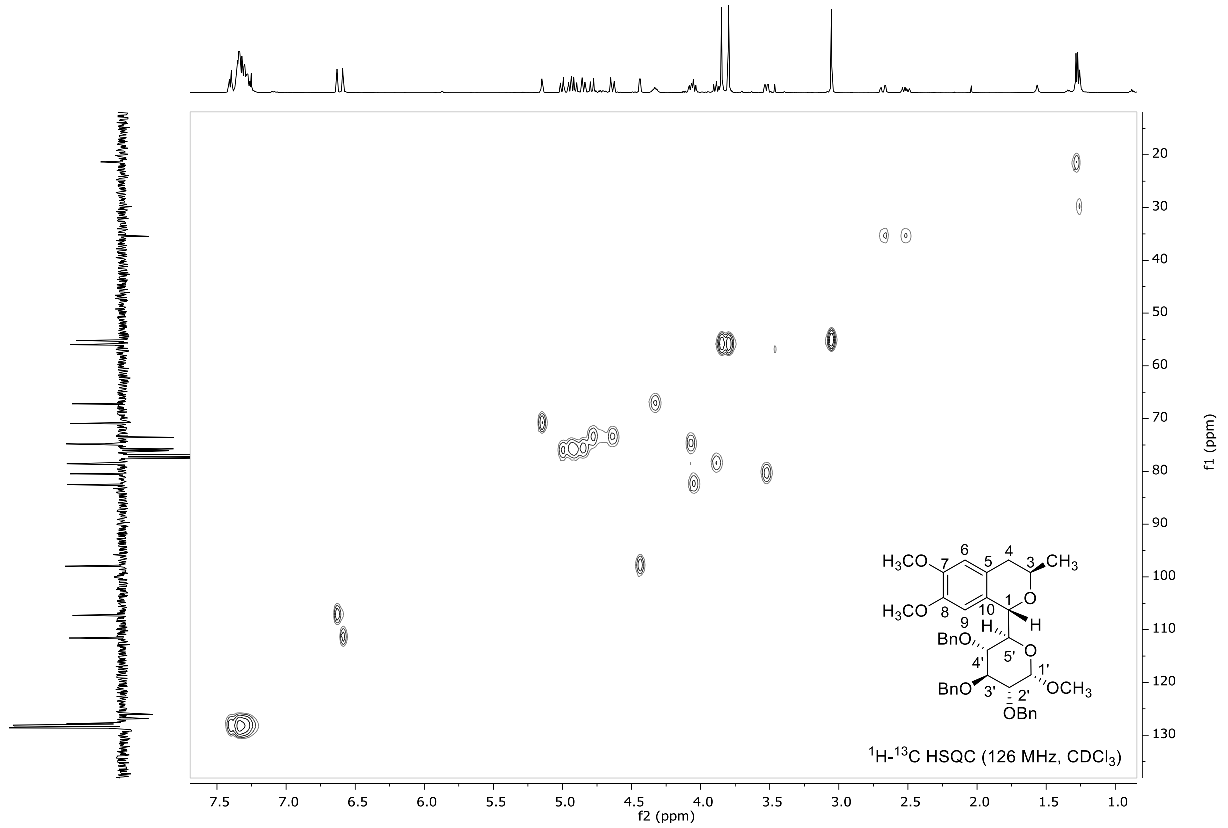

H3CO 7 6 5 4 3 CH3
H3CO 8 9 10 1 O H
H
BnO 4' 5' O 1'
BnO 3' 2' OCH3
OBn
1H-13C HSQC (126 MHz, CDCl3)
f1 (ppm)
f2 (ppm)

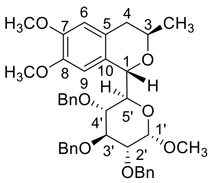

^1H-^1H ROESY (500 MHz, $CDCl_3$)

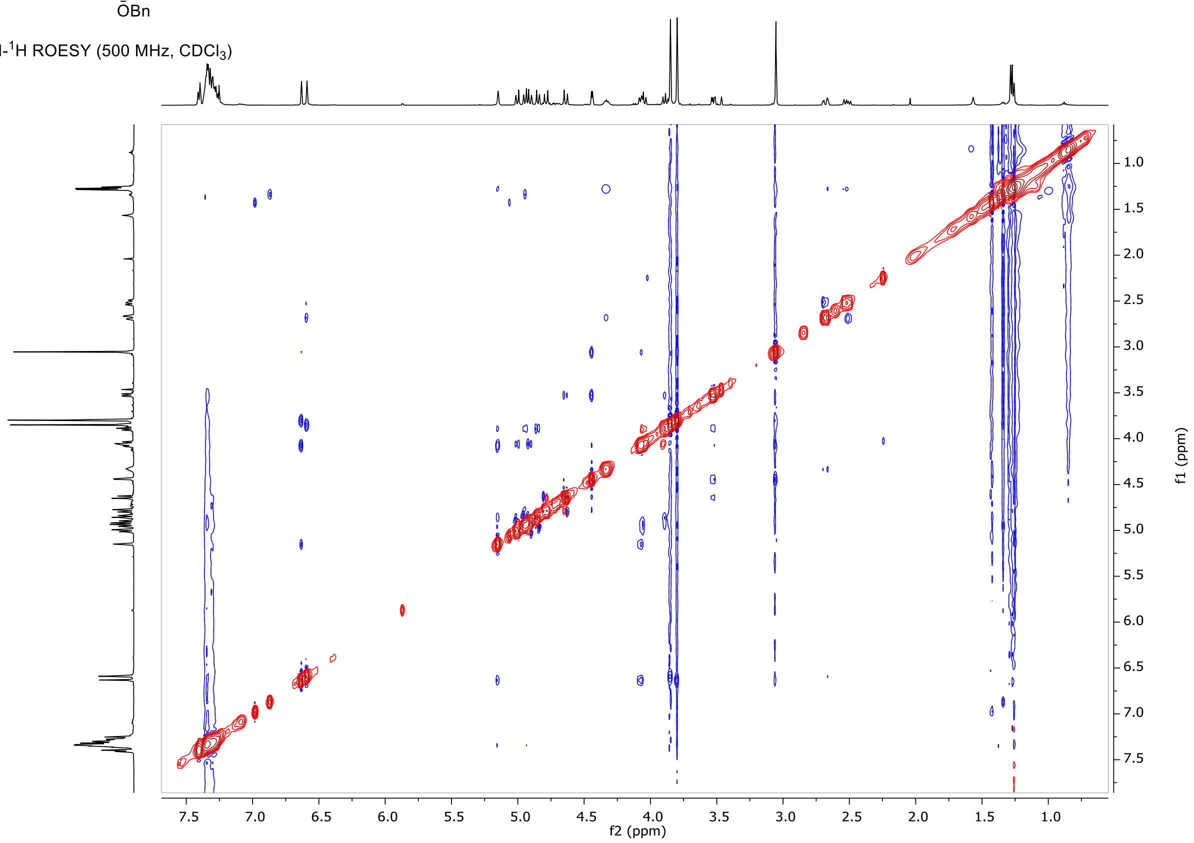

Compound 27

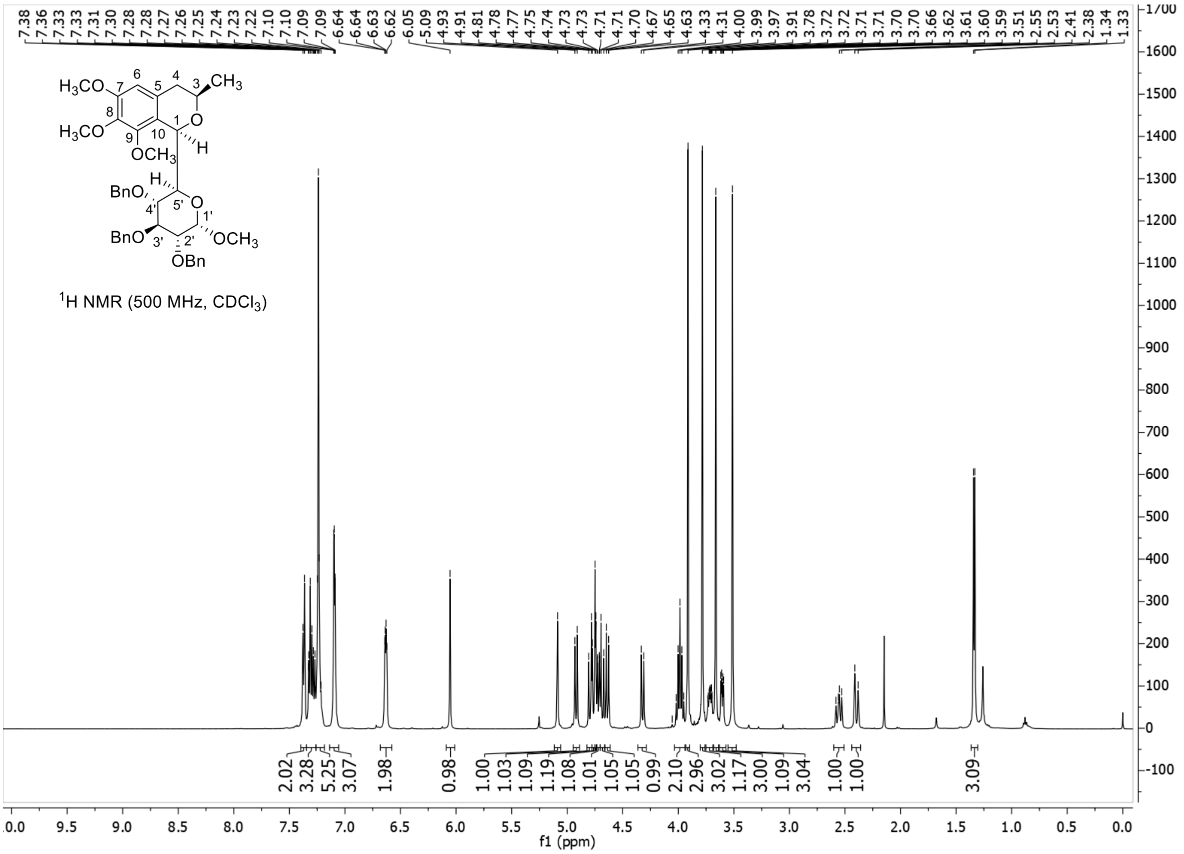

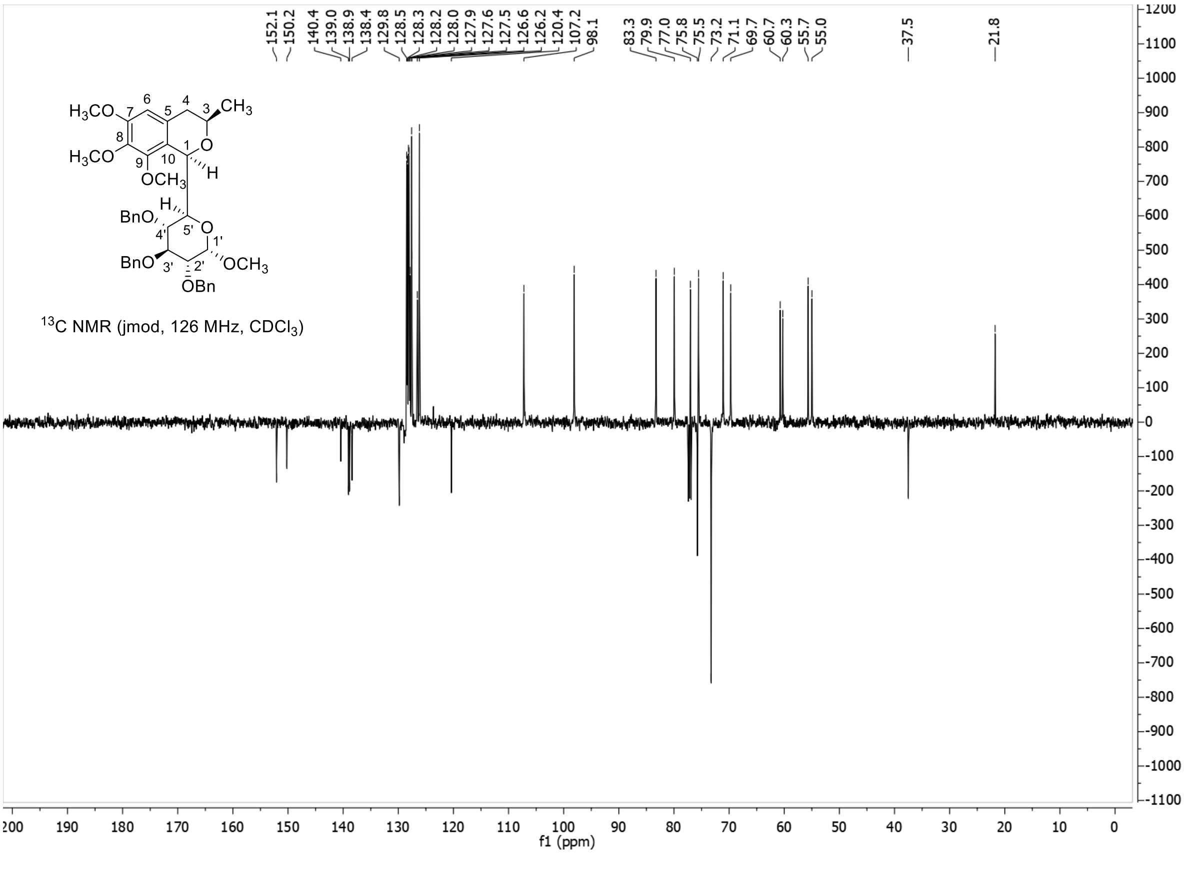

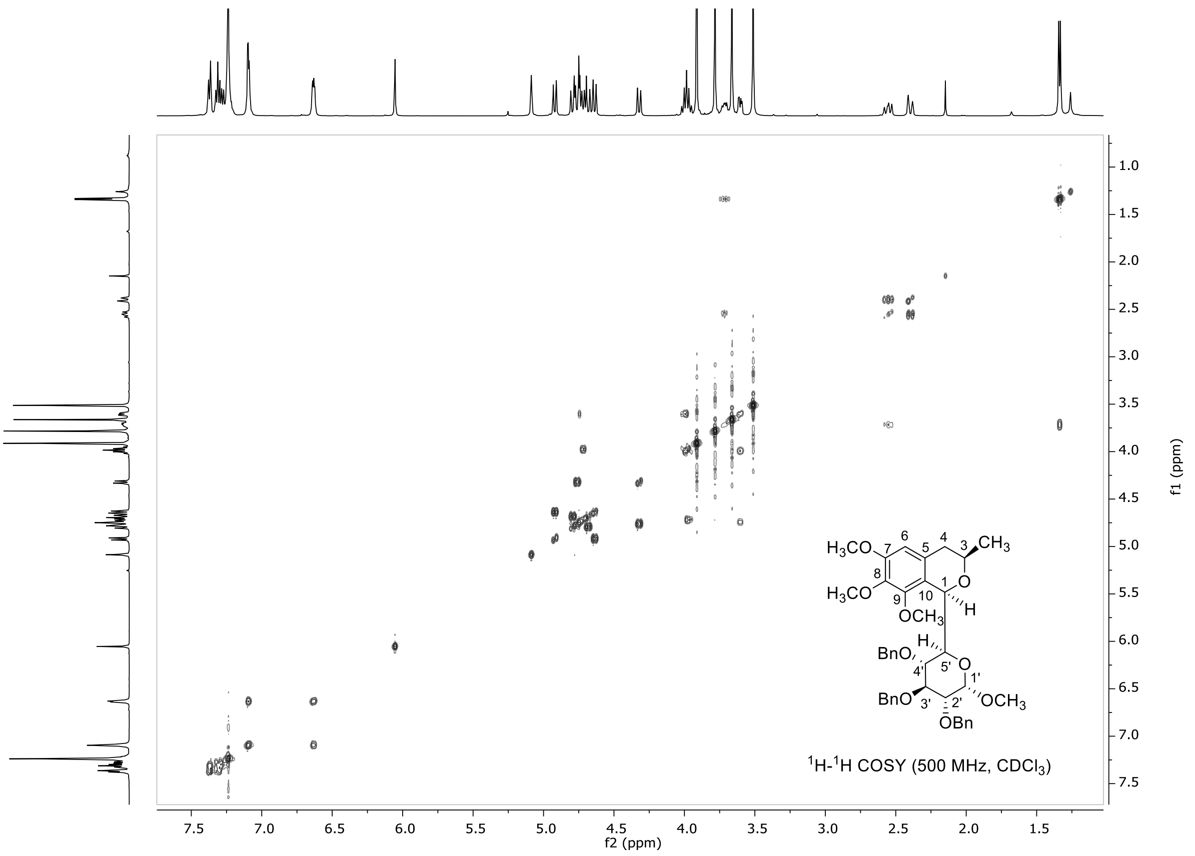
1H-1H COSY (500 MHz, CDCl3)
f1 (ppm)
f2 (ppm)
H3CO
H3CO
OCH3
CH3
BnO
BnO
OBn
OCH3

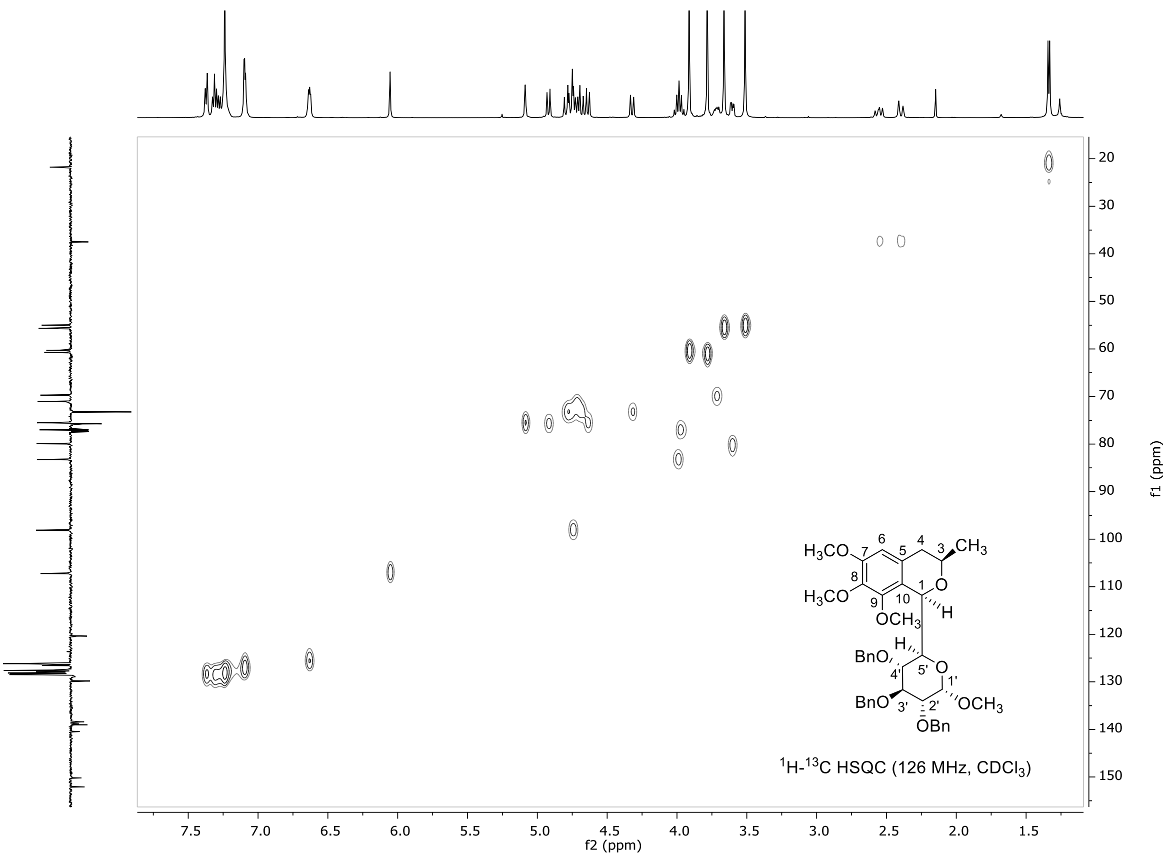
1H-13C HSQC (126 MHz, CDCl3)
f1 (ppm)
f2 (ppm)
H3CO
H3CO
OCH3
CH3
BnO
BnO
OBn
OCH3

Compound 28

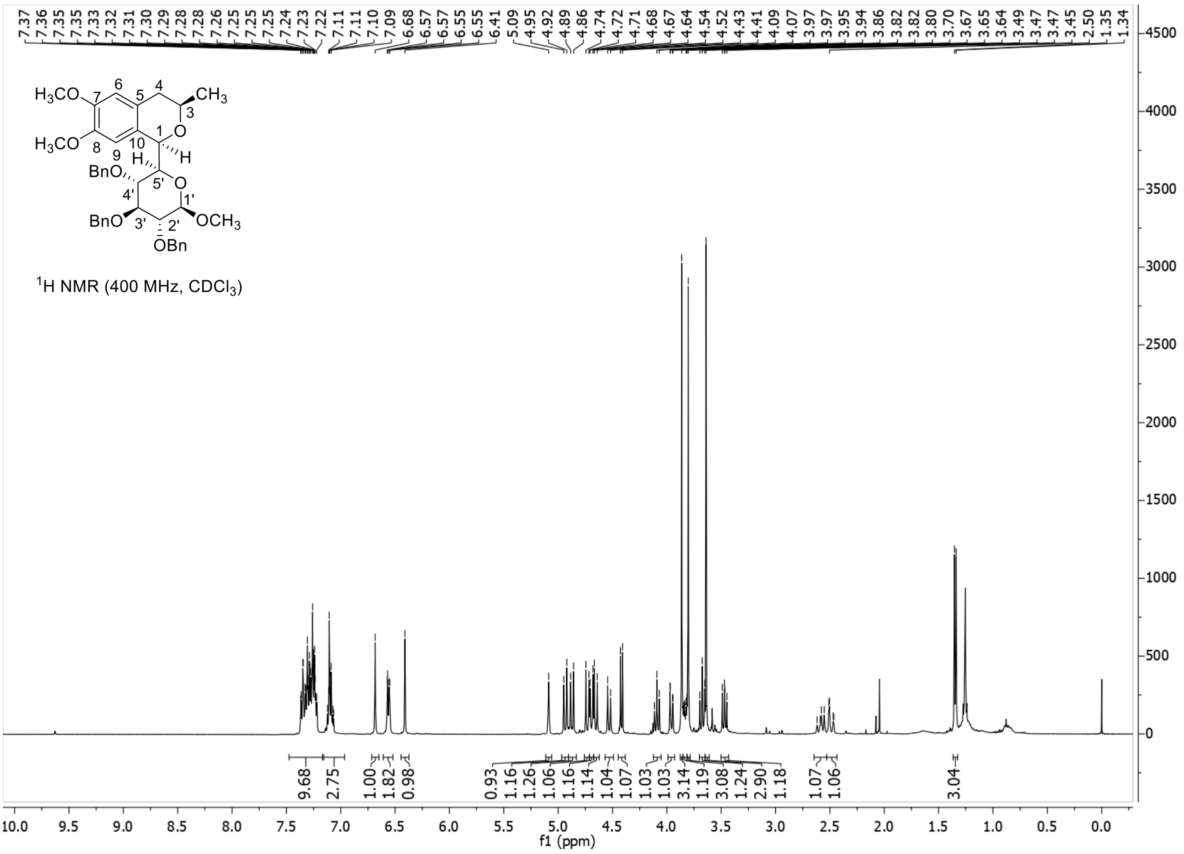

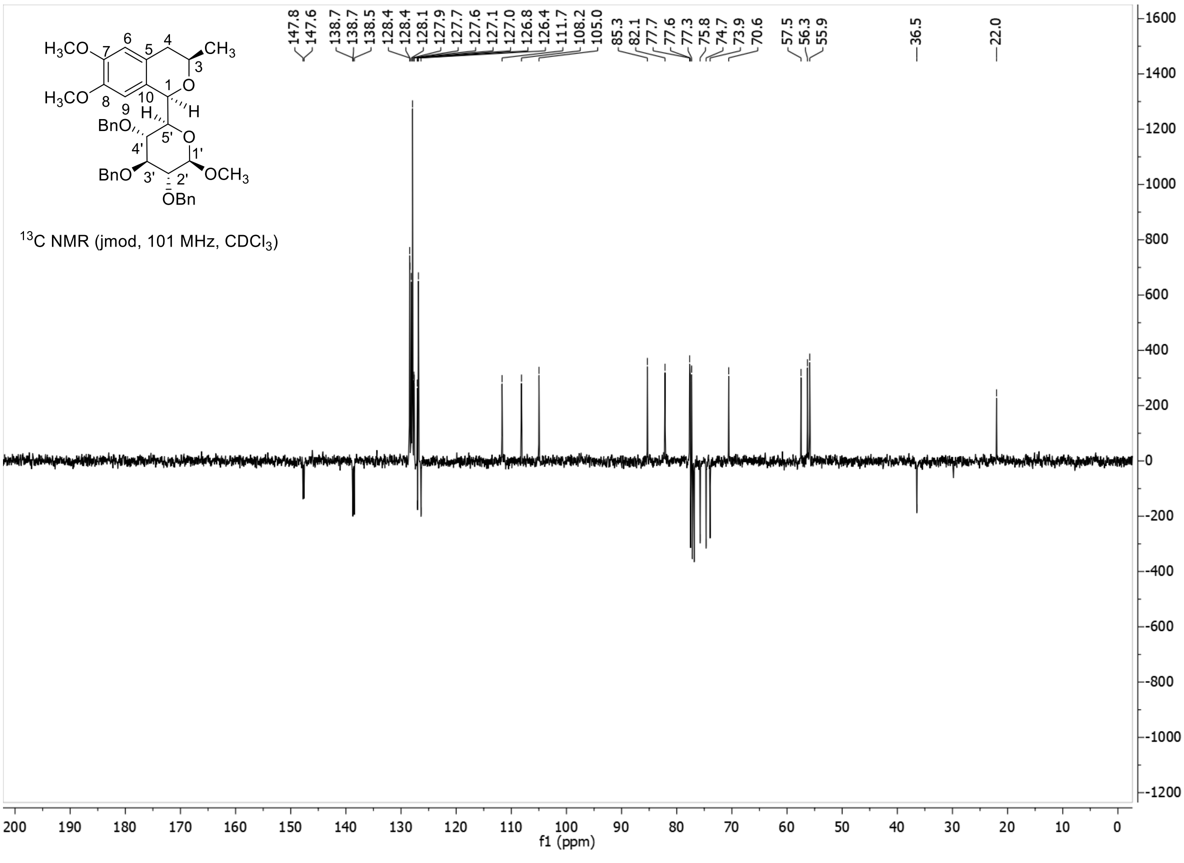

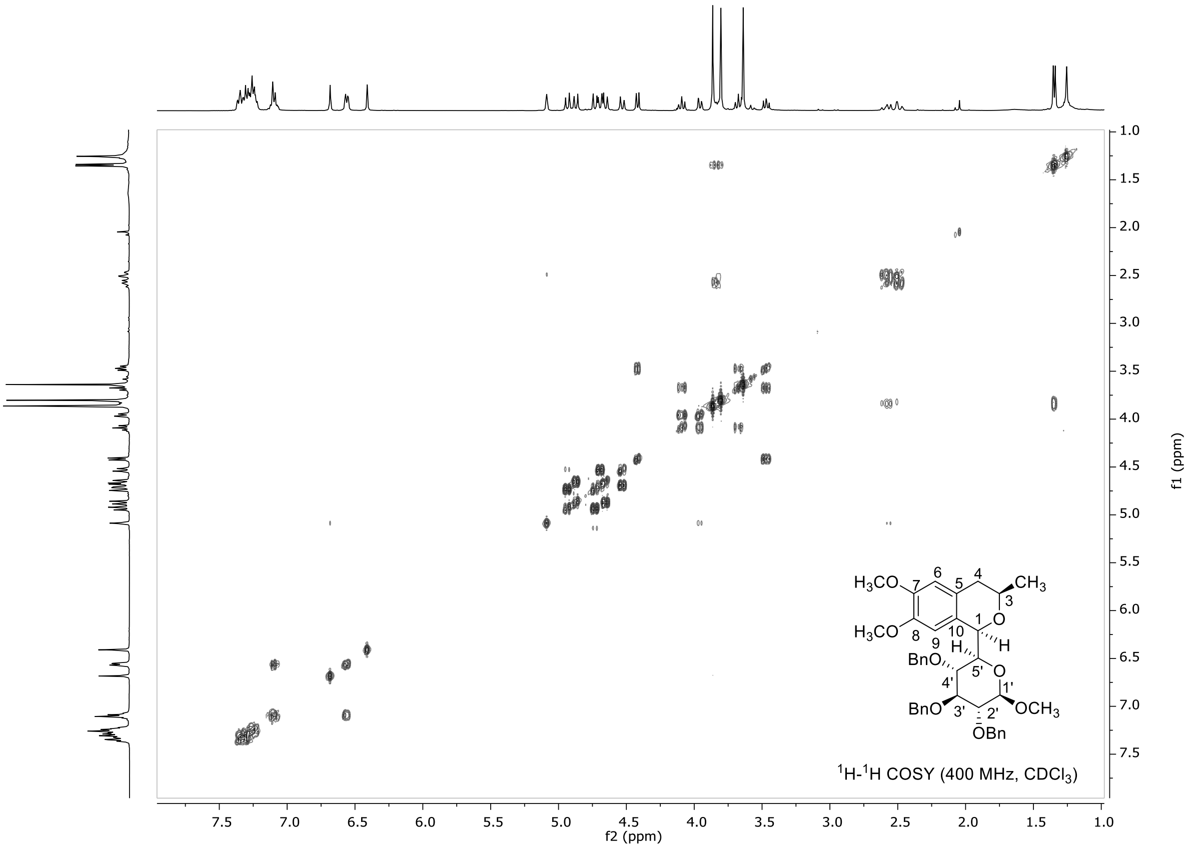

f1 (ppm)
f2 (ppm)
^{1}H-^{1}H COSY (400 MHz, CDCl$_3$)

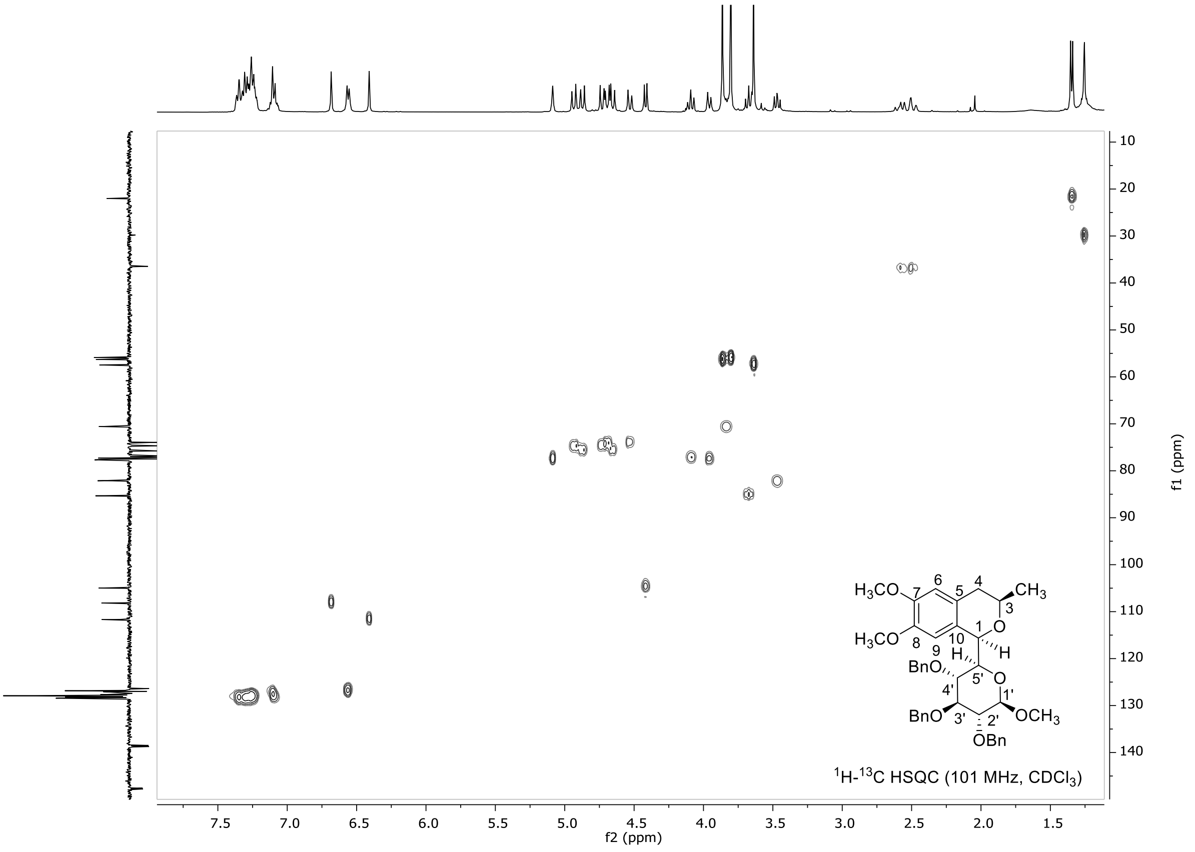

f1 (ppm)
f2 (ppm)
^{1}H-^{13}C HSQC (101 MHz, CDCl$_3$)

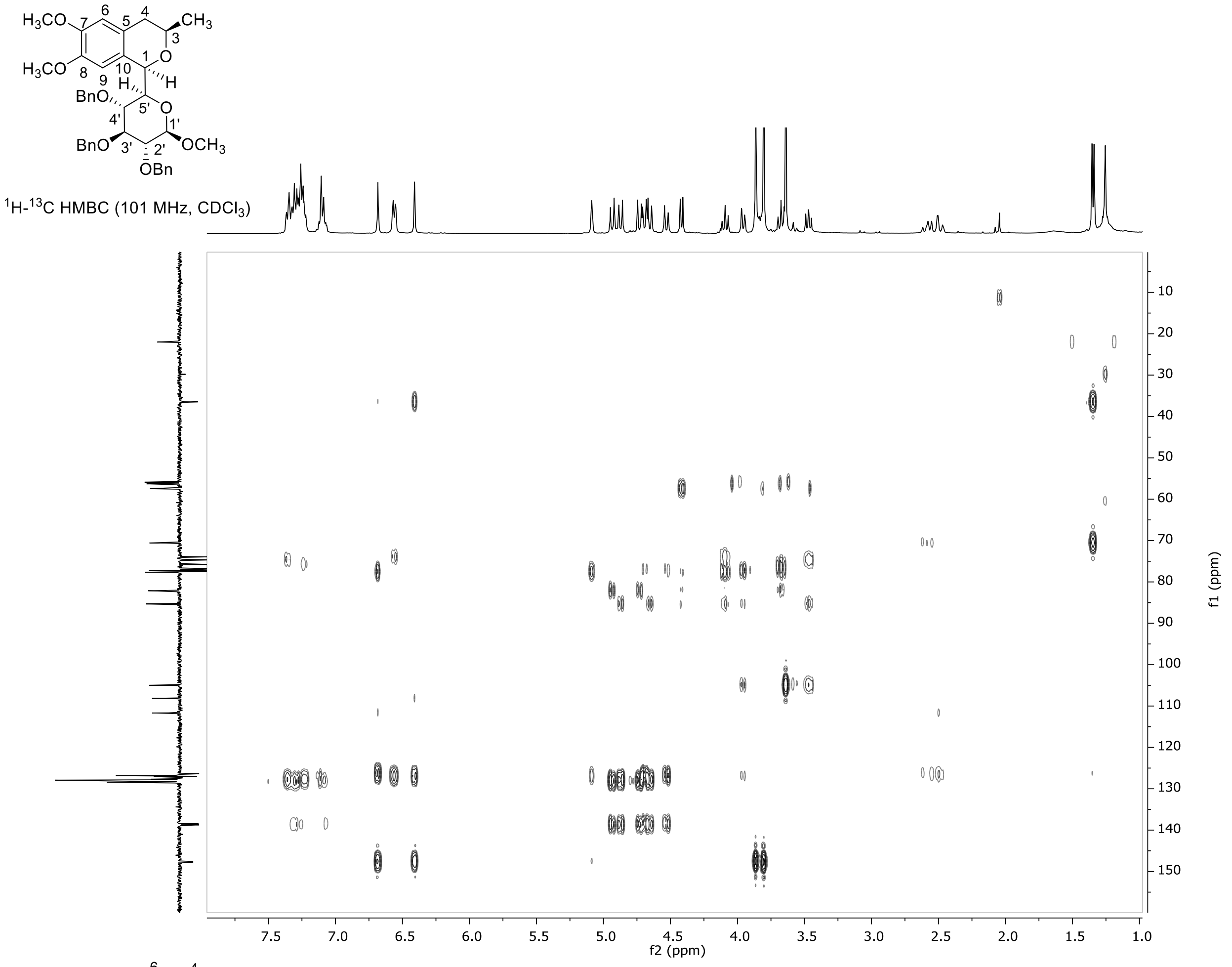

^{1}H-^{13}C HMBC (101 MHz, $CDCl_3$)

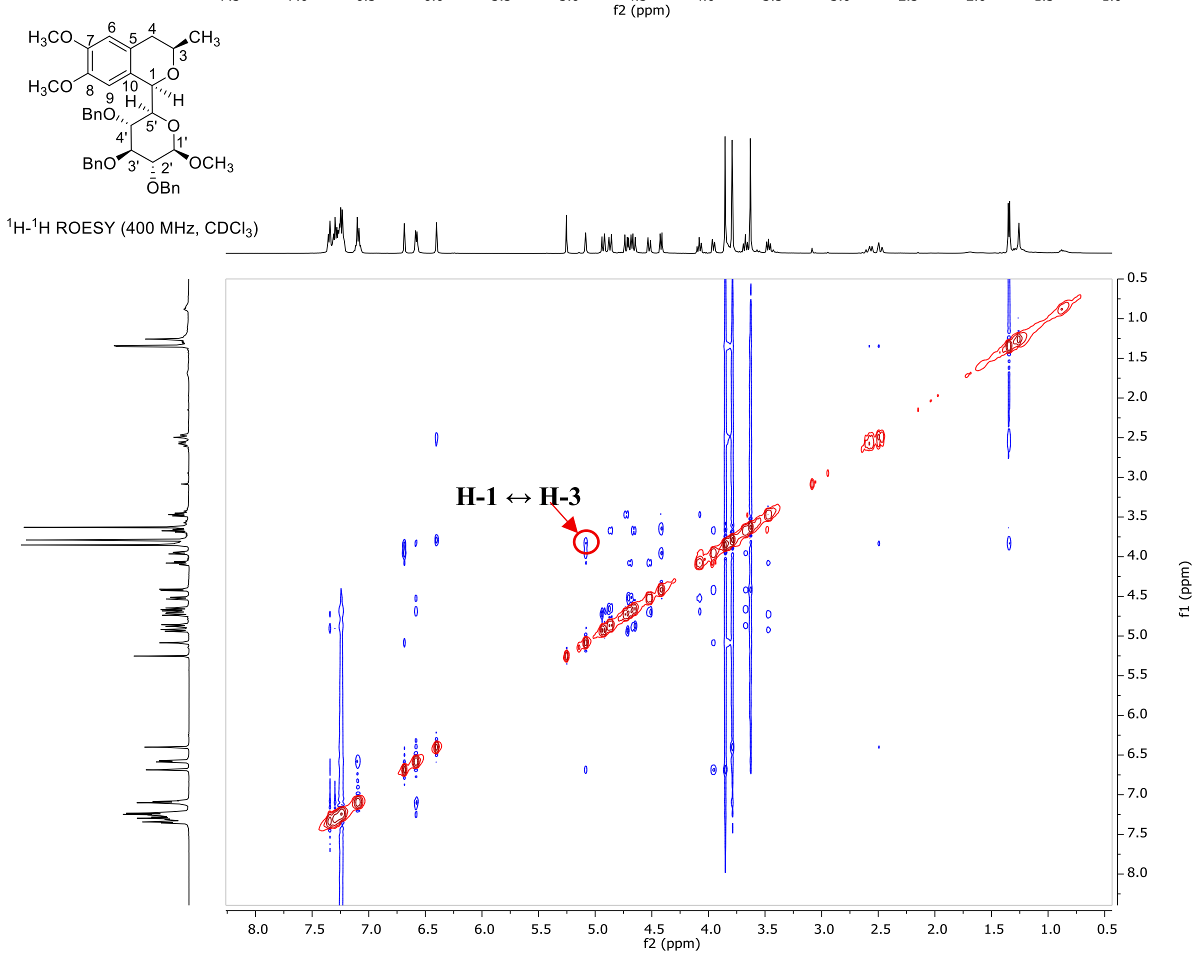

^{1}H-^{1}H ROESY (400 MHz, $CDCl_3$)

Compound 29

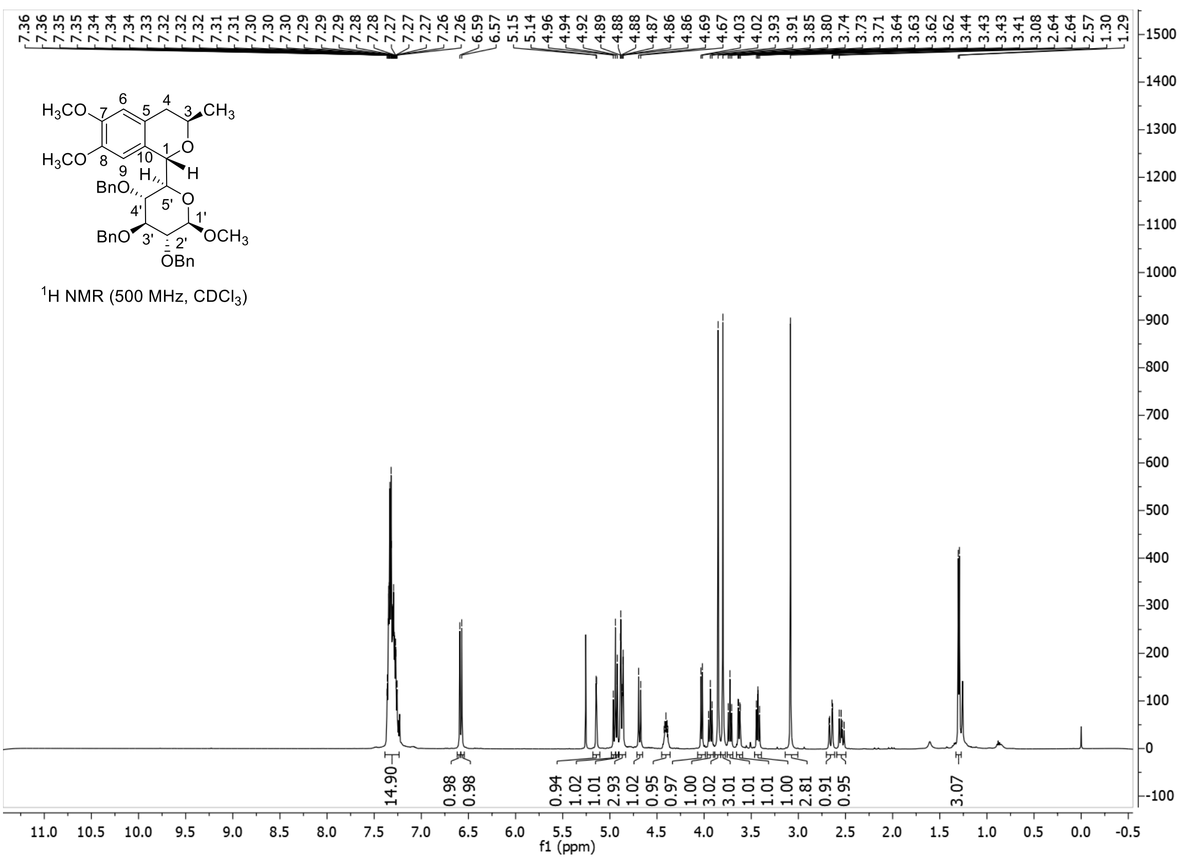
H3CO
H3CO
BnO
BnO
OBn
OCH3
CH3
1H NMR (500 MHz, CDCl3)
f1 (ppm)

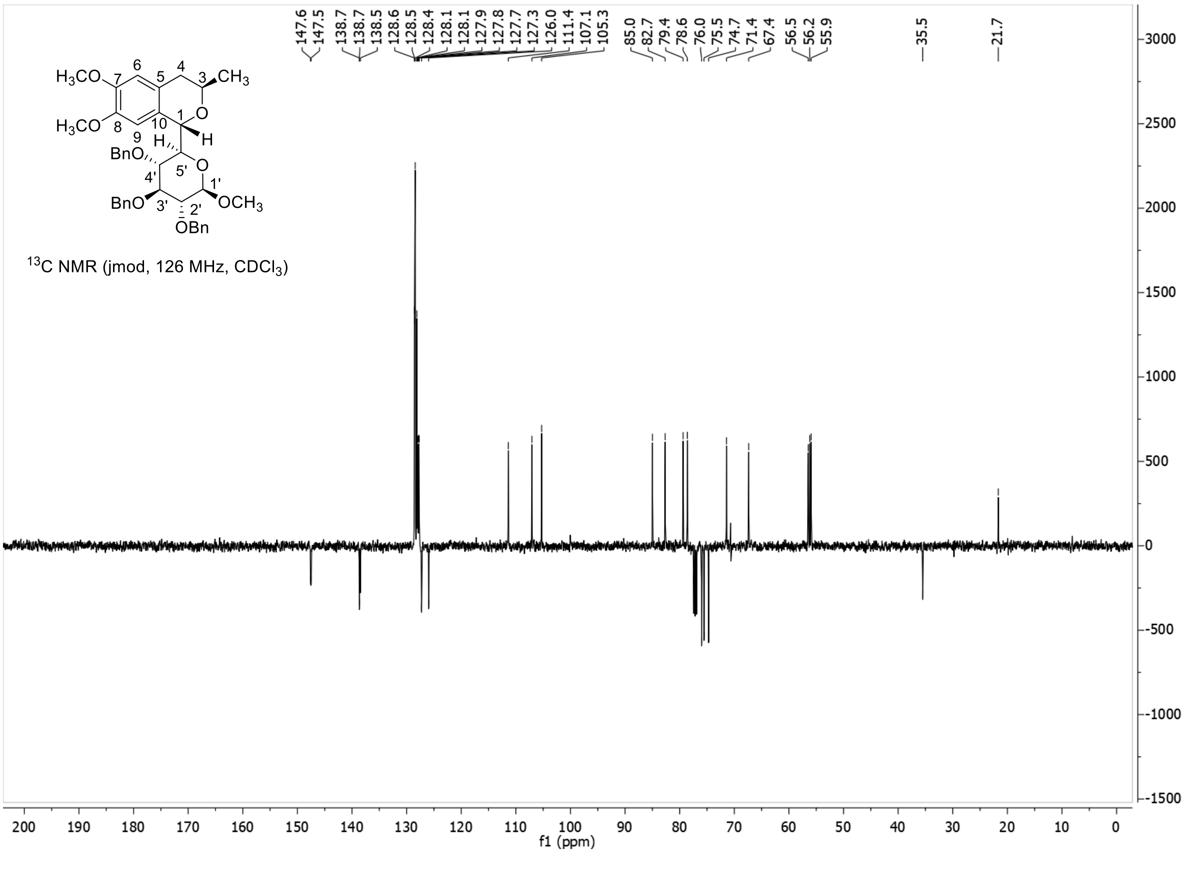
H3CO
H3CO
BnO
BnO
OBn
OCH3
CH3
13C NMR (jmod, 126 MHz, CDCl3)
f1 (ppm)

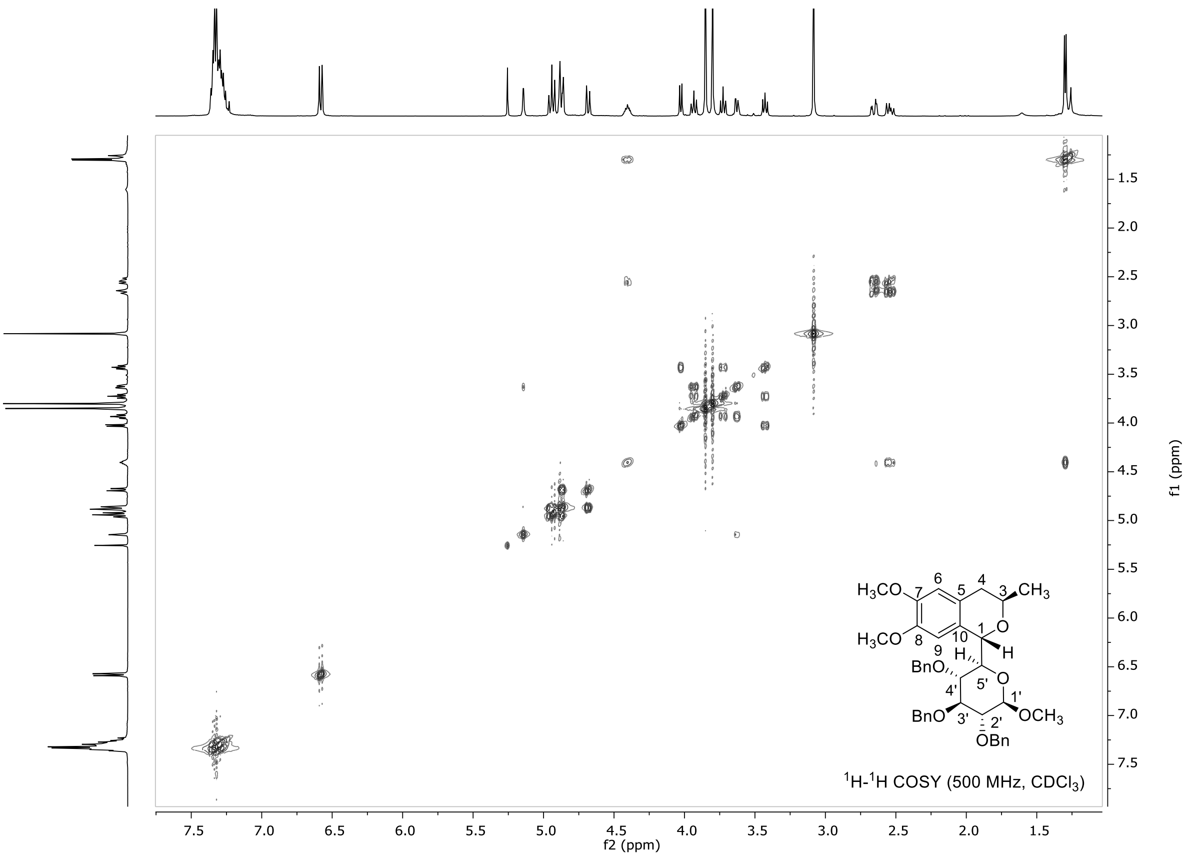
H3CO
H3CO
BnO
BnO
OBn
OCH3
CH3
1H-1H COSY (500 MHz, CDCl3)
f2 (ppm)
f1 (ppm)

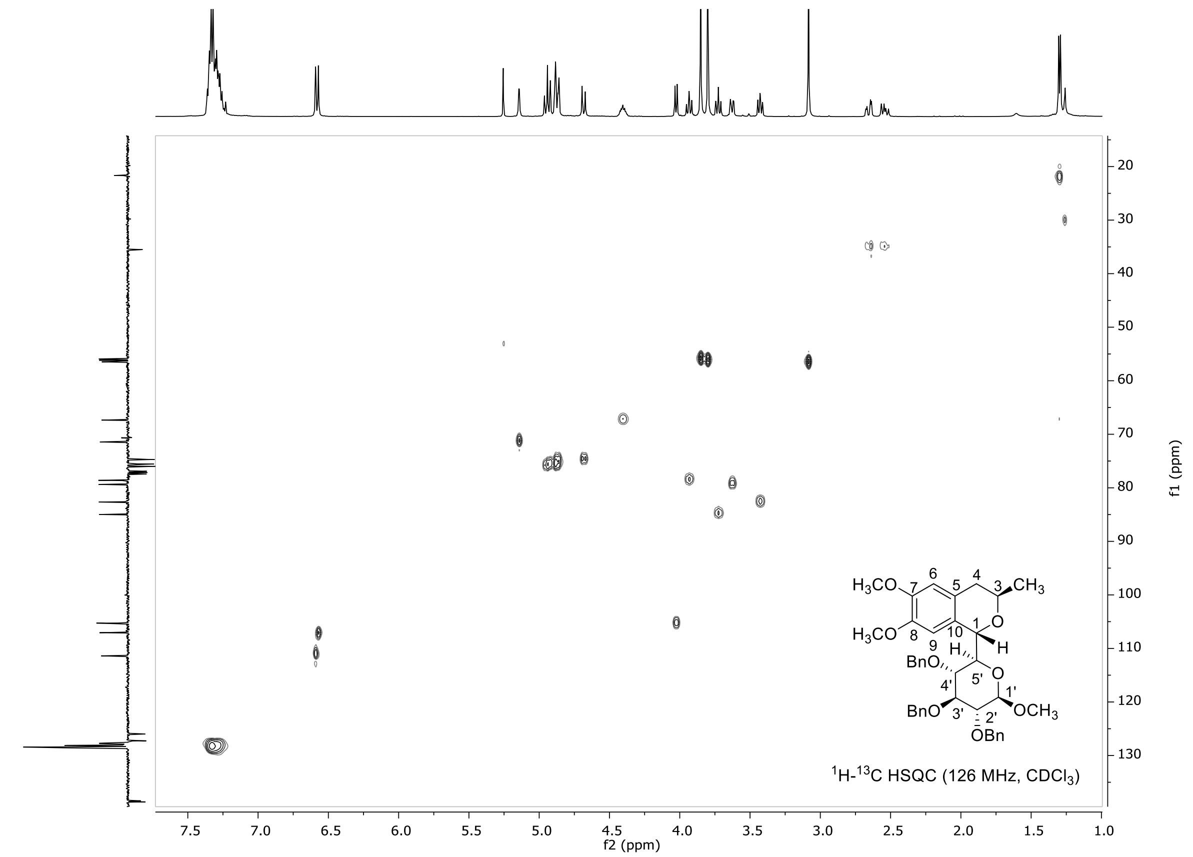
H3CO
H3CO
BnO
BnO
OBn
OCH3
CH3
1H-13C HSQC (126 MHz, CDCl3)
f2 (ppm)
f1 (ppm)

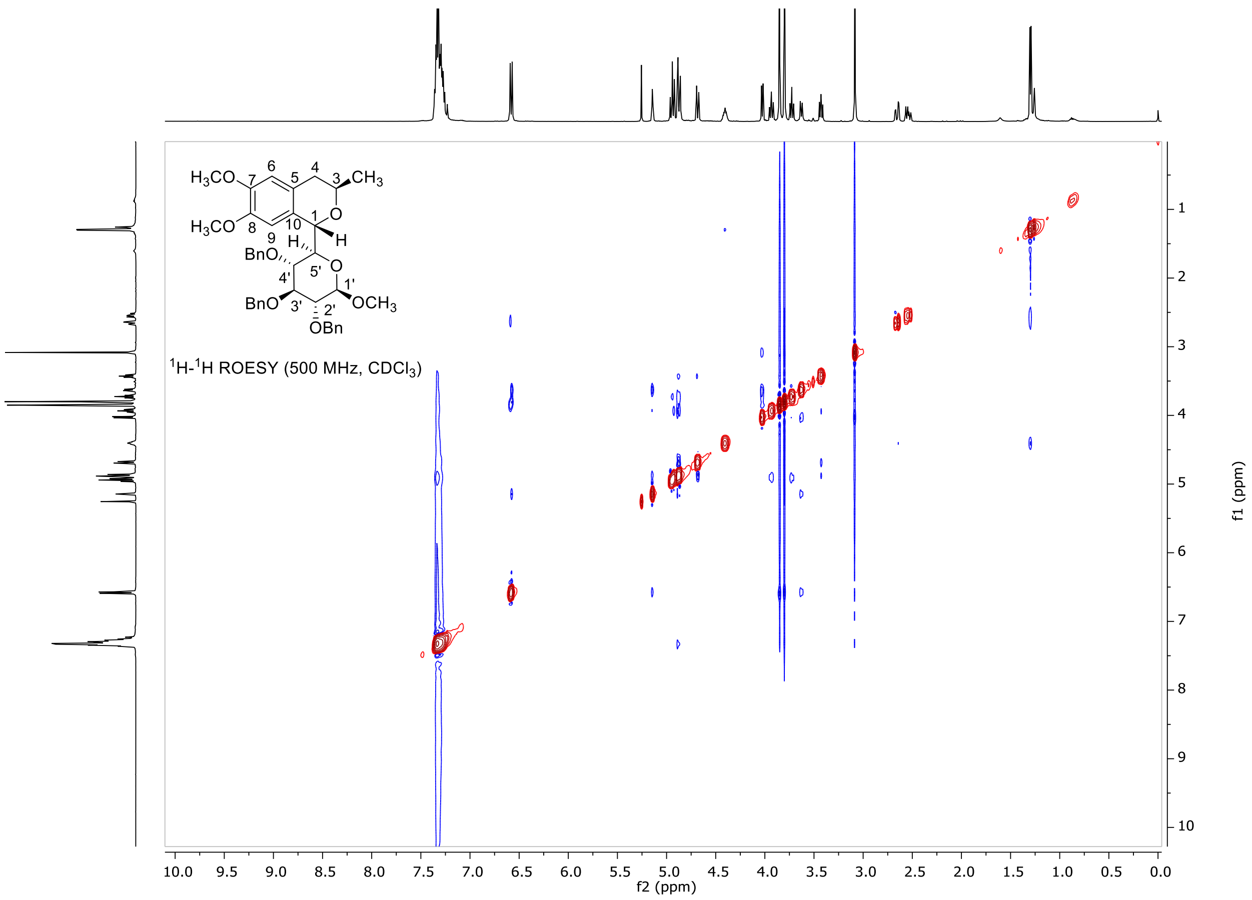
H3CO
H3CO
BnO
BnO
OBn
O
O
CH3
OCH3
H
H
1H-1H ROESY (500 MHz, CDCl3)
f2 (ppm)
f1 (ppm)

Compound 30

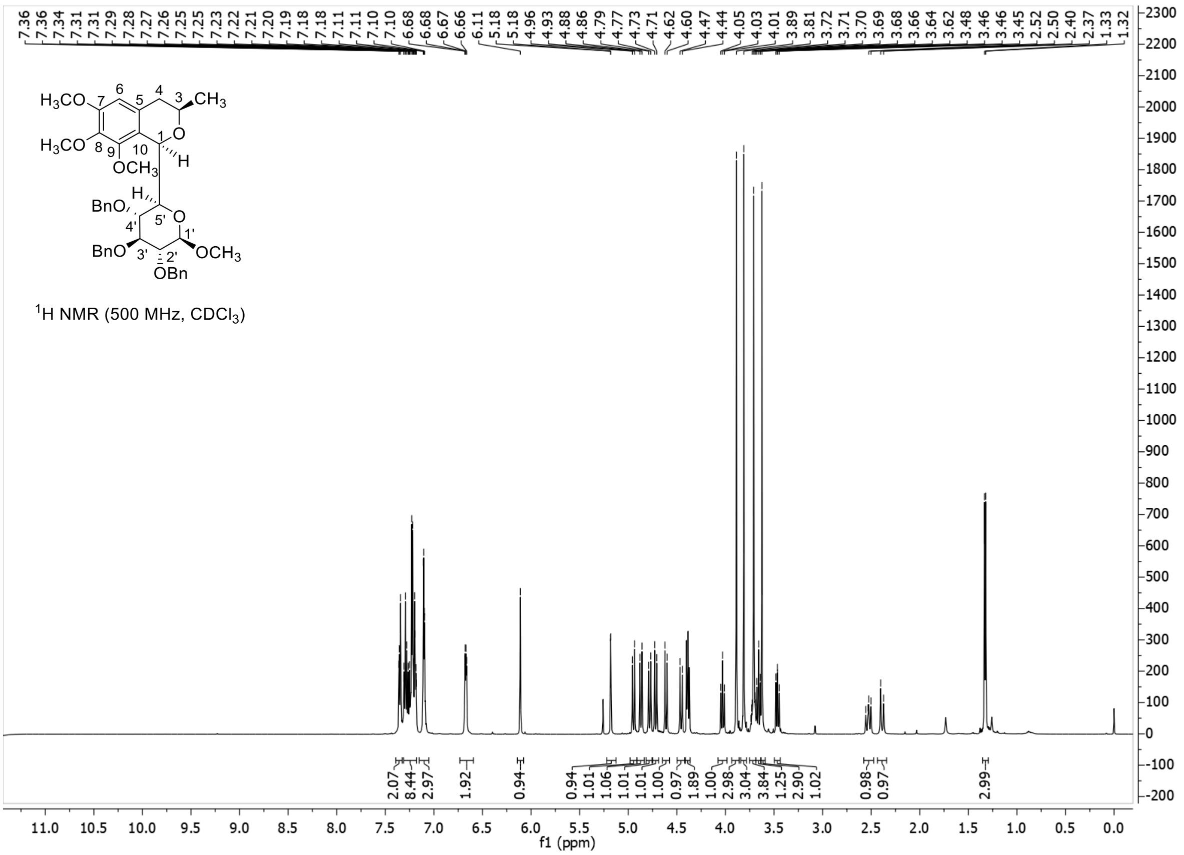

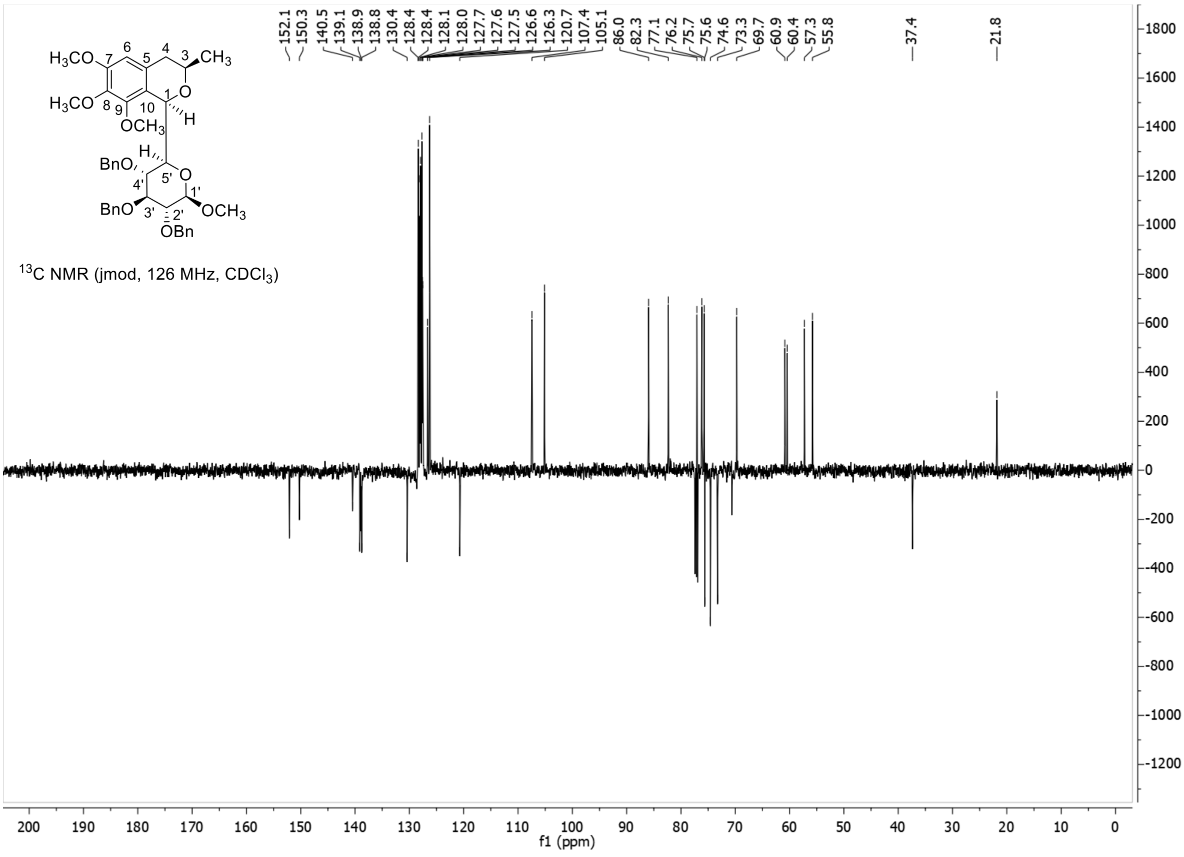

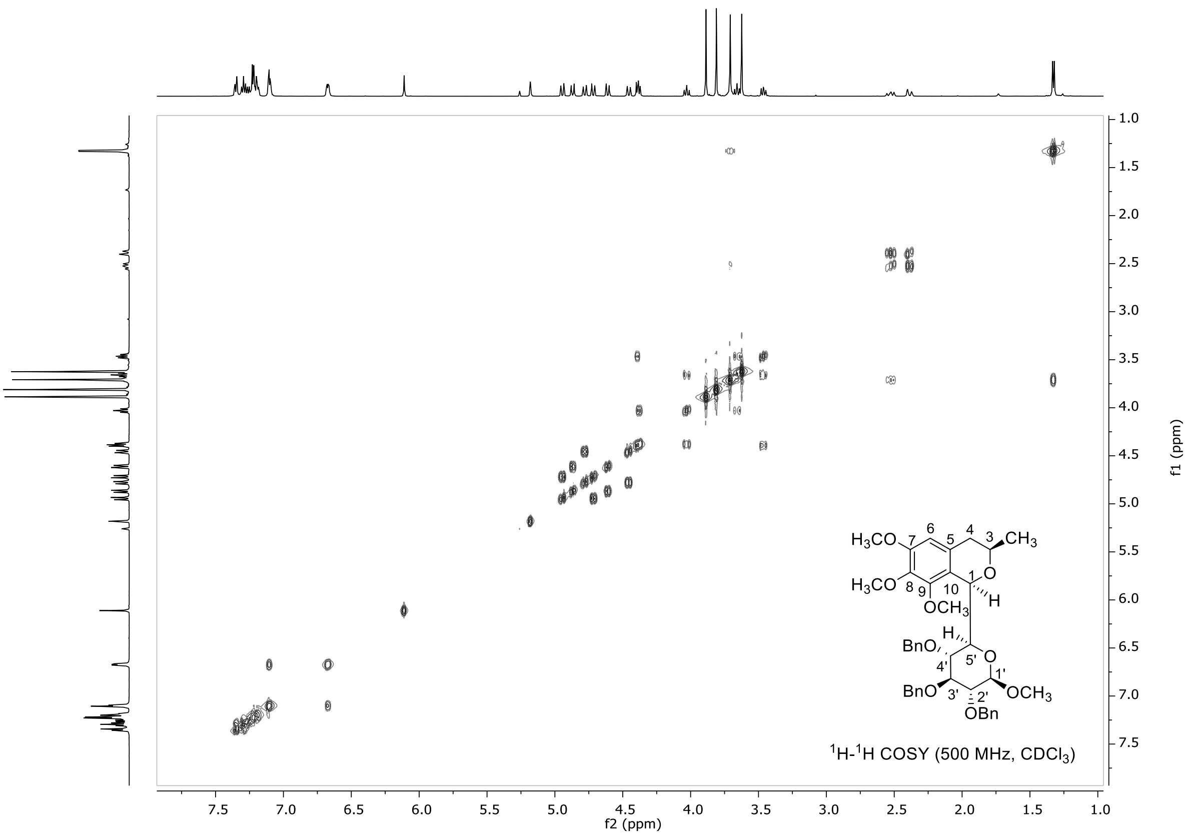
H3CO 7 6 5 4 3 CH3
H3CO 8 9 10 1 O H
OCH3
H
BnO 4' 5' O 1'
BnO 3' 2' OCH3
OBn
1H-1H COSY (500 MHz, CDCl3)
f1 (ppm)
f2 (ppm)

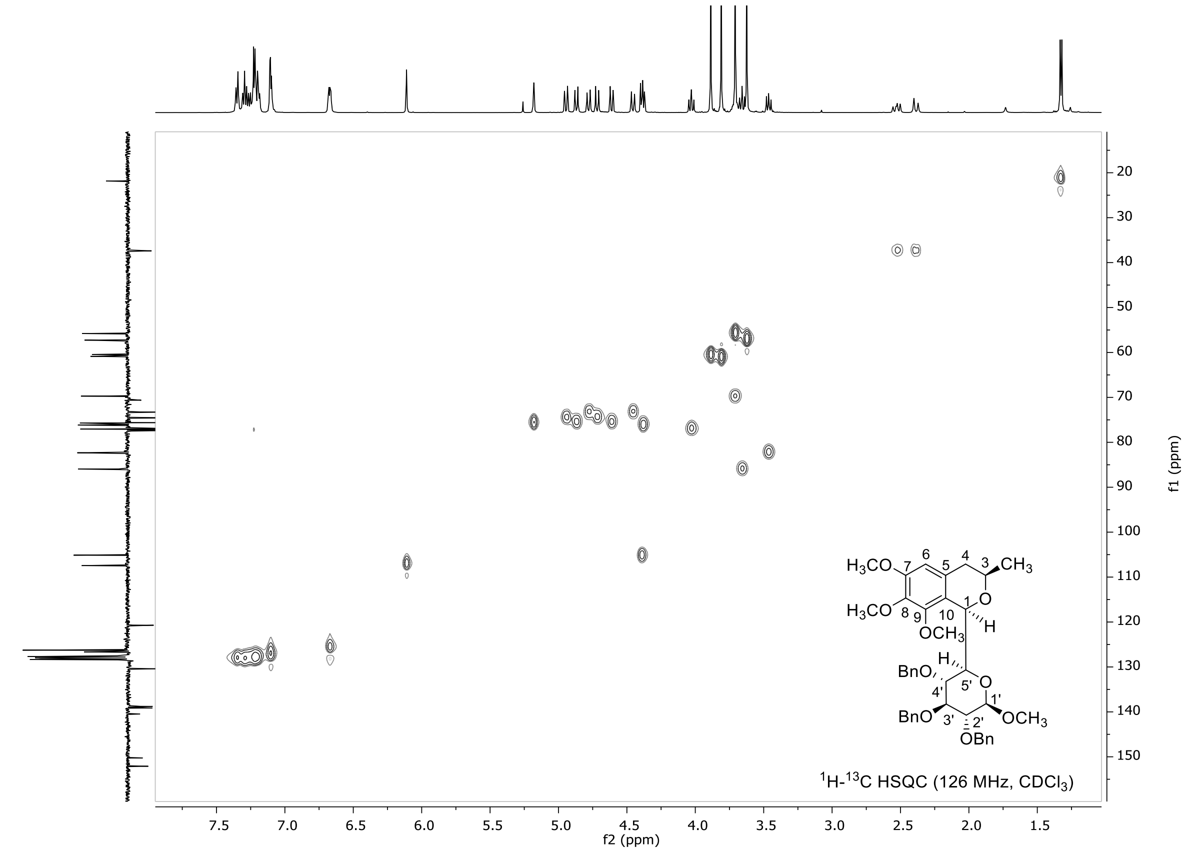
H3CO 7 6 5 4 3 CH3
H3CO 8 9 10 1 O H
OCH3
H
BnO 4' 5' O 1'
BnO 3' 2' OCH3
OBn
1H-13C HSQC (126 MHz, CDCl3)
f1 (ppm)
f2 (ppm)

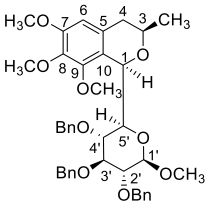

^{1}H-^{13}C HMBC (126 MHz, $CDCl_3$)

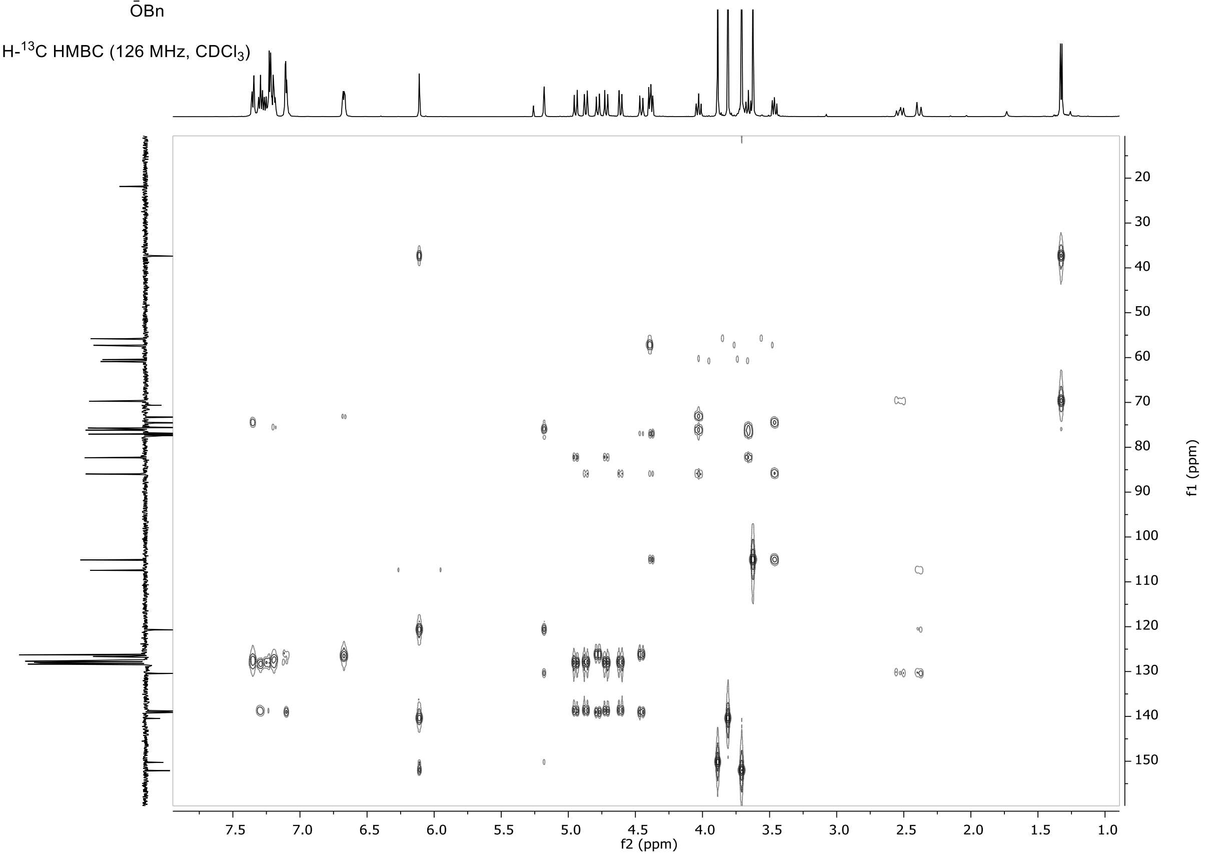

Compound 31

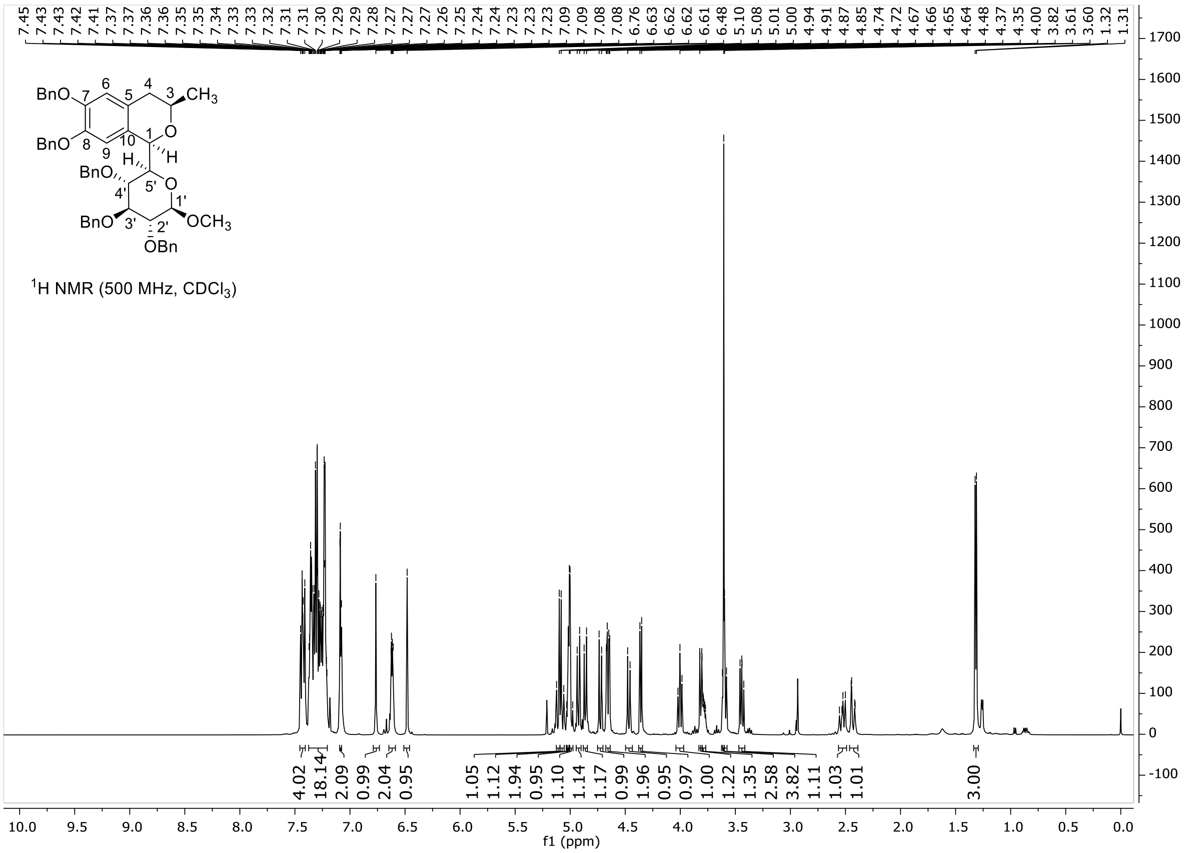
7.45 7.43 7.43 7.42 7.41 7.37 7.36 7.36 7.35 7.35 7.34 7.33 7.33 7.32 7.31 7.31 7.30 7.29 7.29 7.28 7.27 7.27 7.27 7.26 7.25 7.24 7.24 7.23 7.23 7.23 7.09 7.09 7.08 7.08 6.76 6.63 6.62 6.62 6.61 6.48 5.10 5.08 5.01 5.00 4.94 4.91 4.87 4.85 4.74 4.72 4.67 4.66 4.65 4.64 4.48 4.37 4.35 4.00 3.82 3.61 3.60 1.32 1.31
^{1}H NMR (500 MHz, $CDCl_3$)
4.02 18.14 2.09 0.99 2.04 0.95 1.05 1.12 1.94 0.95 1.10 1.14 1.17 0.99 1.96 0.95 0.97 1.00 1.22 1.35 2.58 3.82 1.11 1.03 1.01 3.00
f1 (ppm)

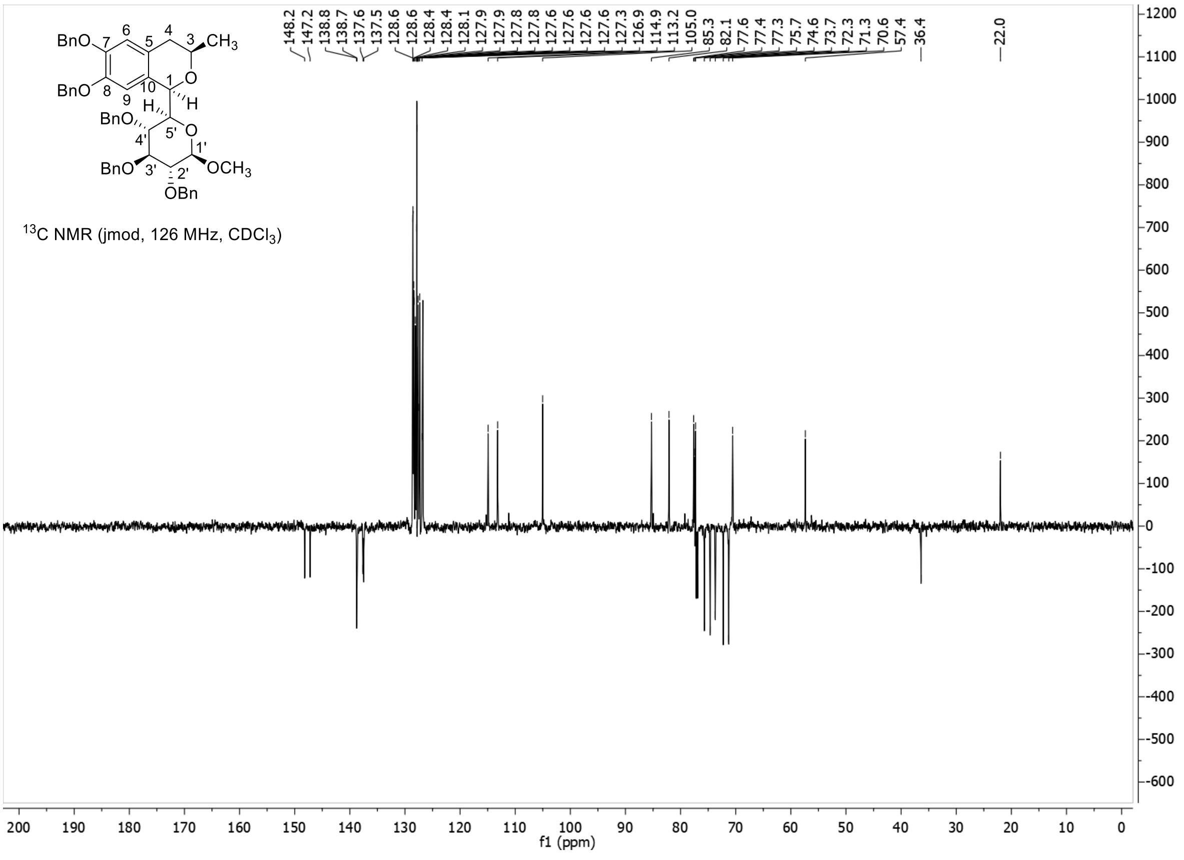
148.2 147.2 138.8 138.7 137.6 137.5 128.6 128.4 128.4 128.1 127.9 127.9 127.8 127.6 127.6 127.6 127.3 126.9 114.9 113.2 105.0 85.3 82.1 77.6 77.4 77.3 75.7 74.6 73.7 72.3 71.3 70.6 57.4 36.4 22.0
^{13}C NMR (jmod, 126 MHz, $CDCl_3$)
f1 (ppm)

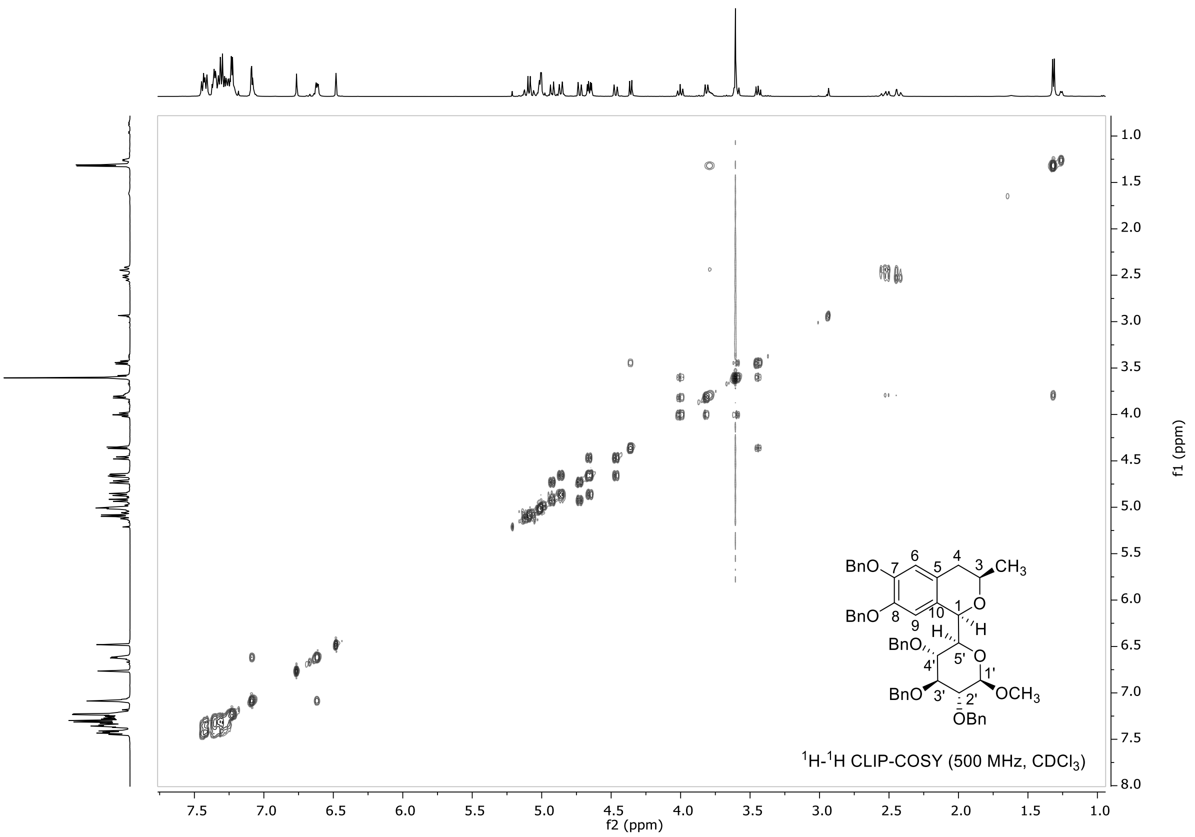
BnO
BnO
CH3
^{1}H-^{1}H CLIP-COSY (500 MHz, CDCl$_3$)
f2 (ppm)
f1 (ppm)

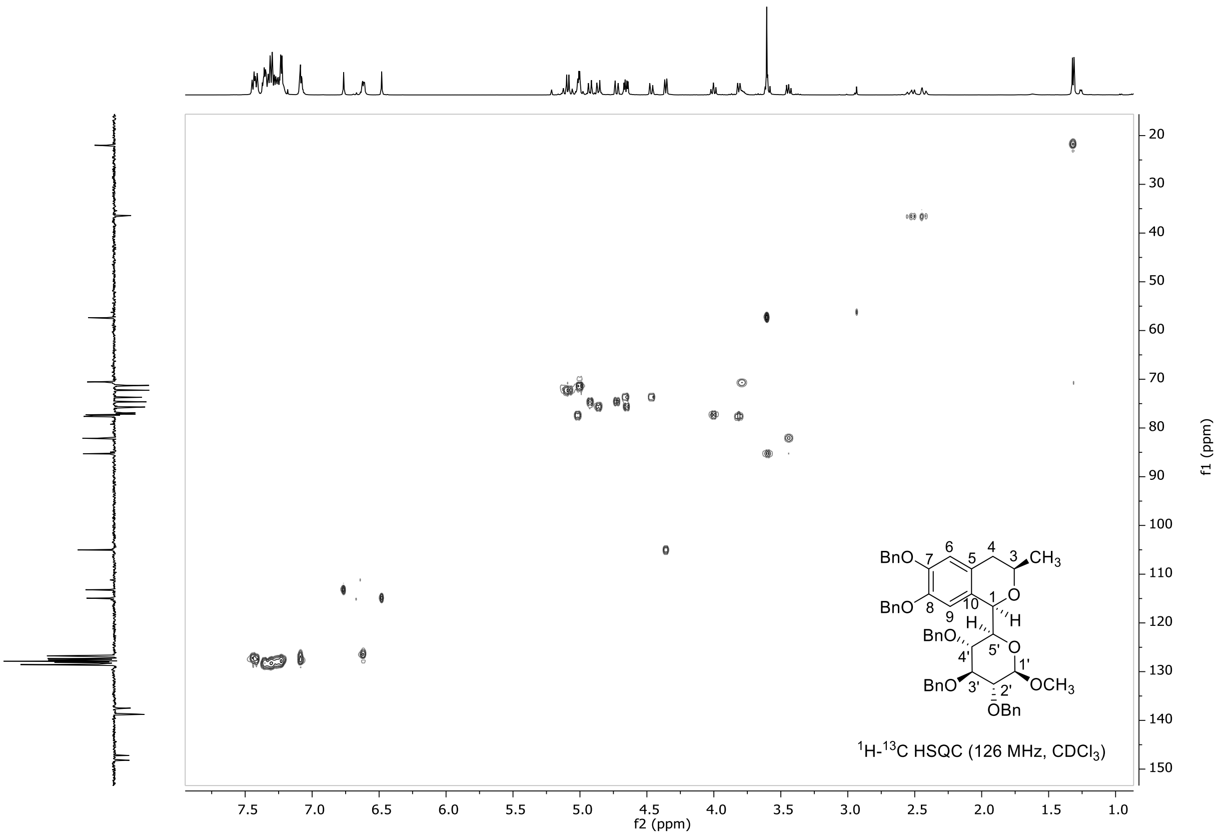
BnO
BnO
CH3
^{1}H-^{13}C HSQC (126 MHz, CDCl$_3$)
f2 (ppm)
f1 (ppm)

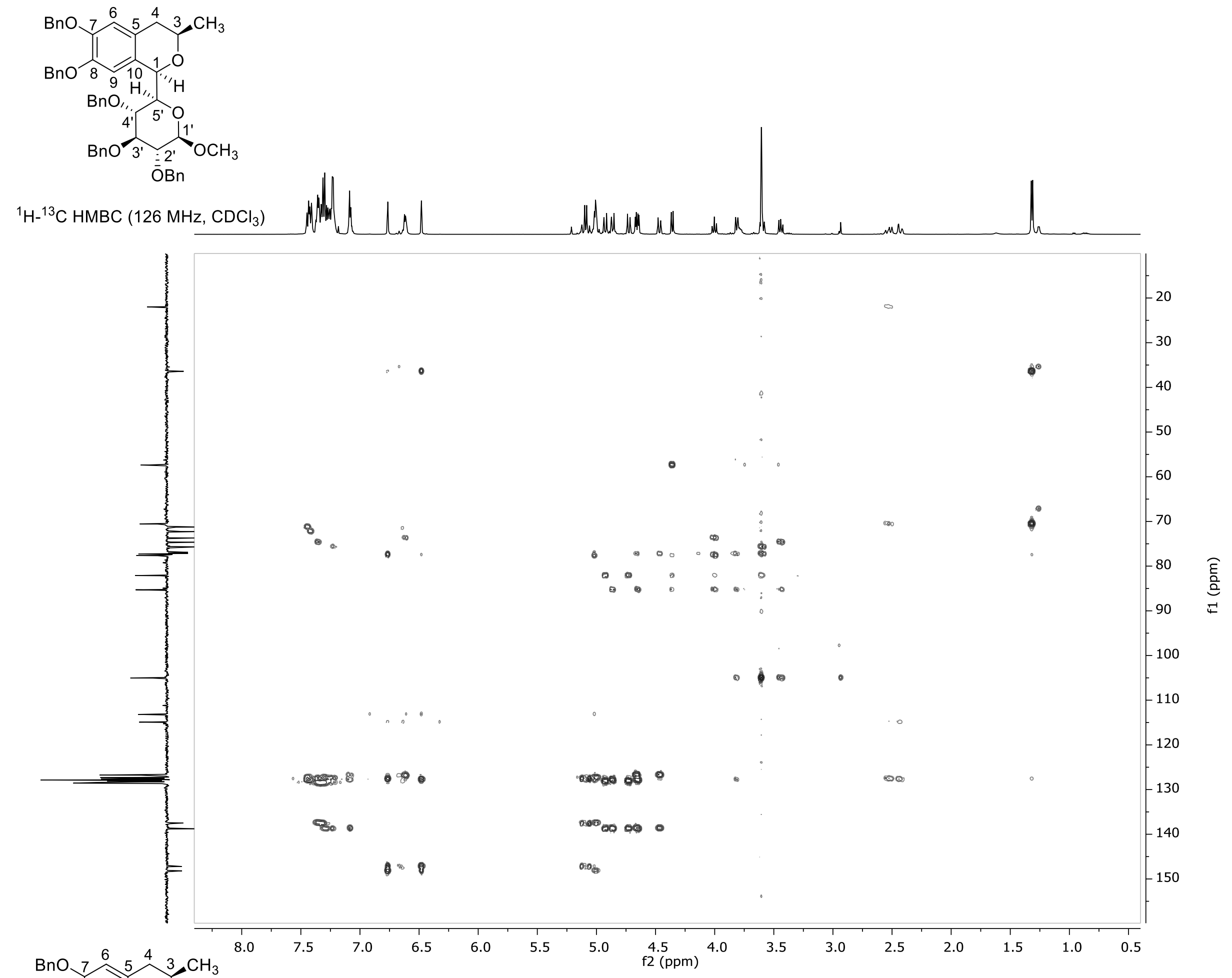

^{1}H-^{13}C HMBC (126 MHz, $CDCl_3$)

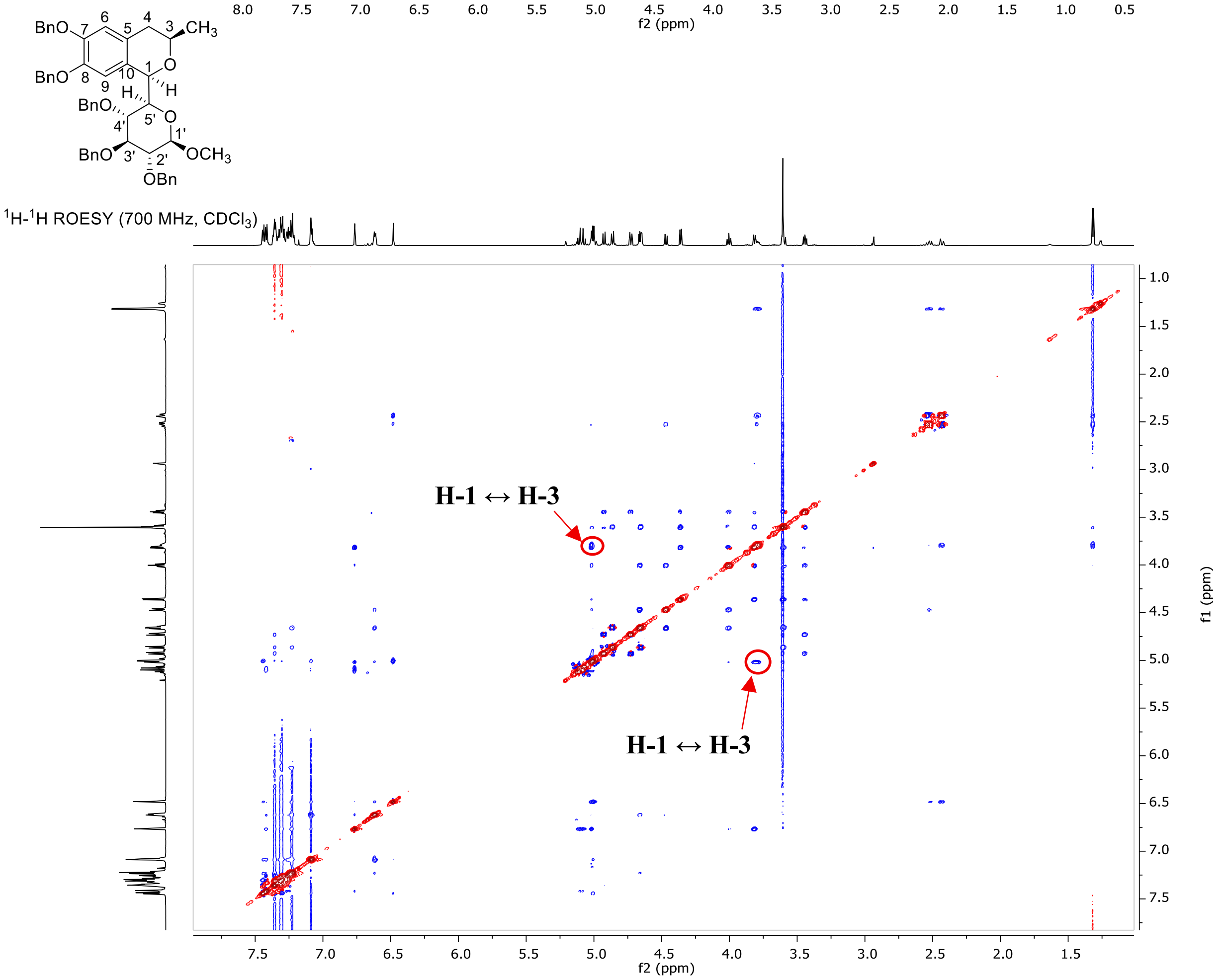

^{1}H-^{1}H ROESY (700 MHz, $CDCl_3$)

Compound 32

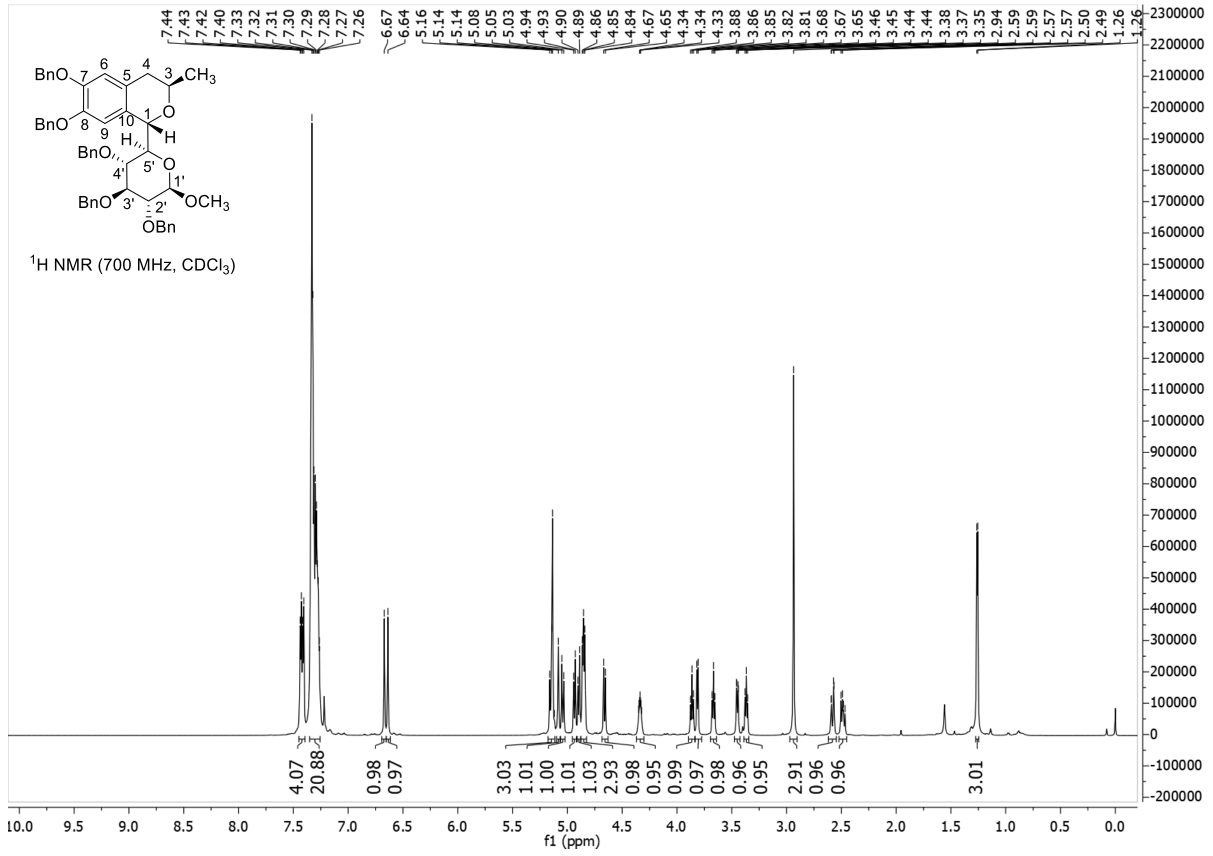

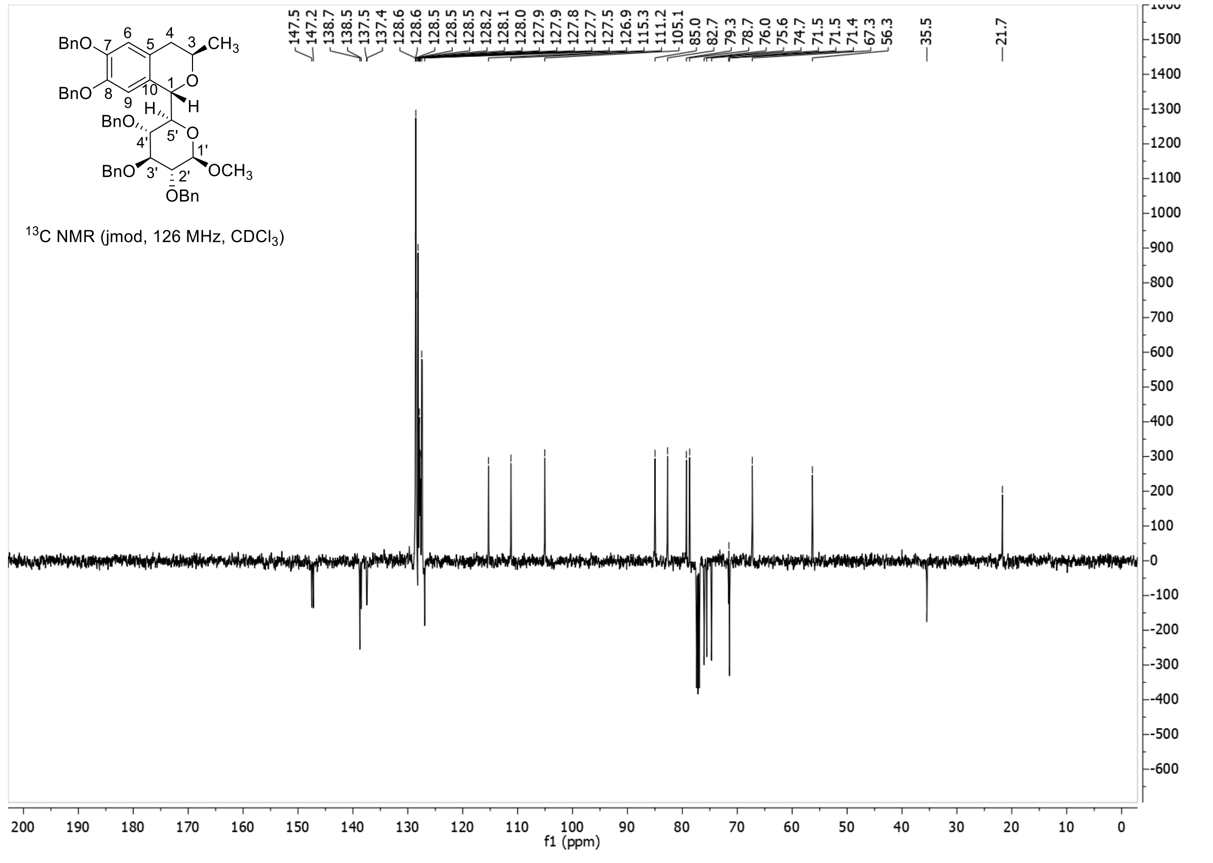

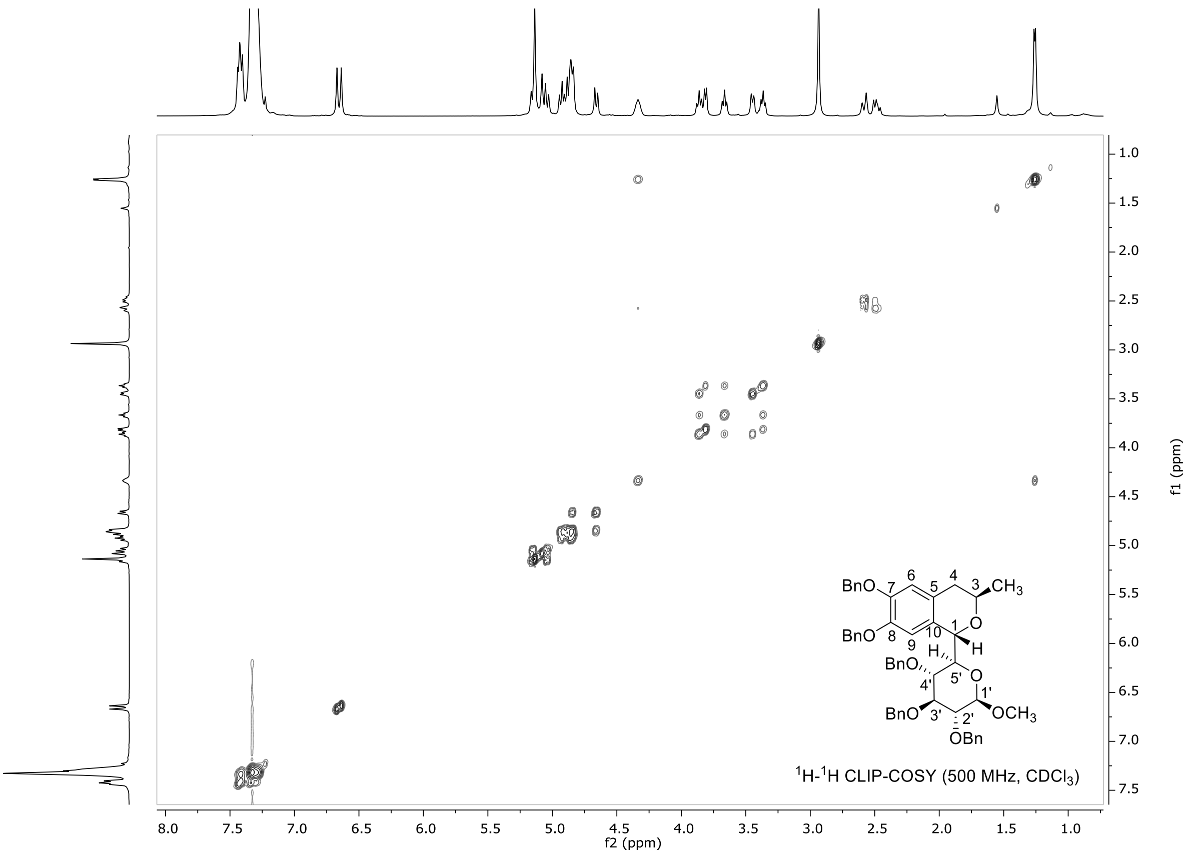

1H-1H CLIP-COSY (500 MHz, CDCl3)
f2 (ppm)
f1 (ppm)
BnO
BnO
OBn
OCH3
CH3

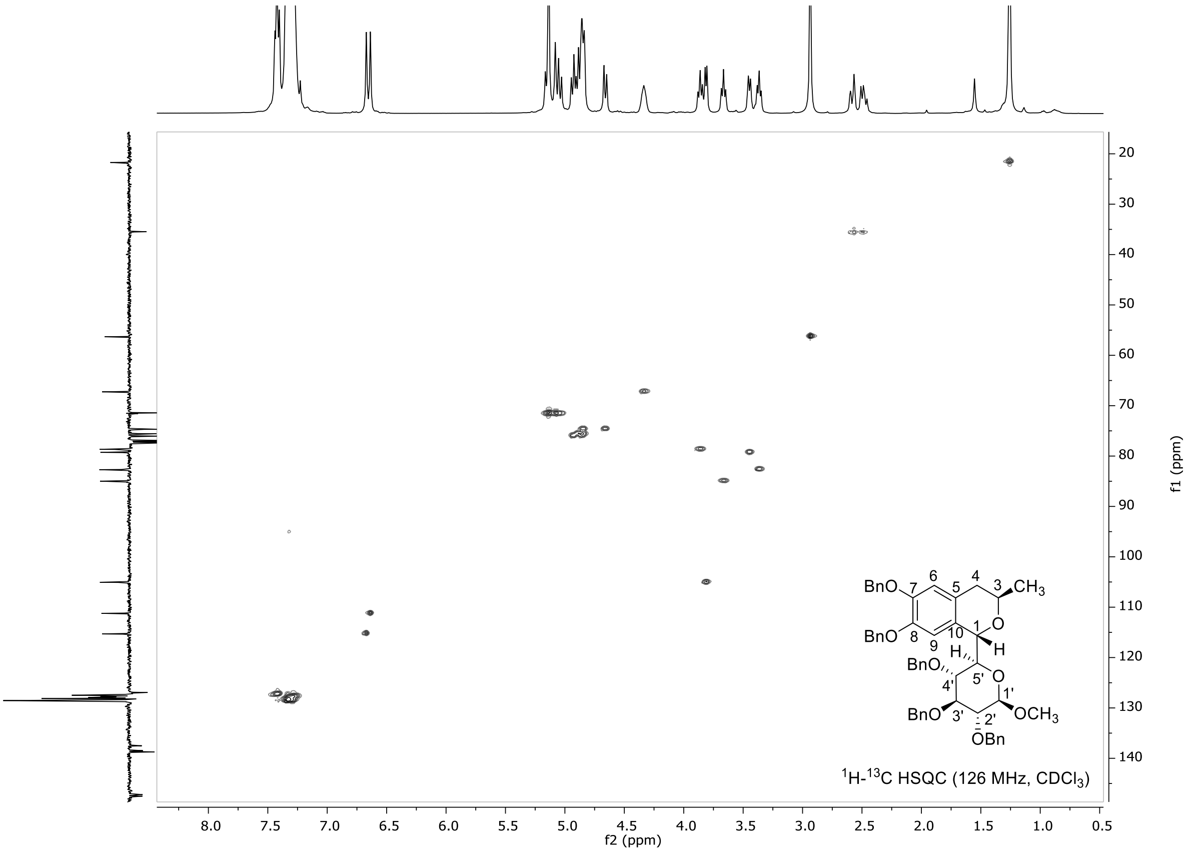

1H-13C HSQC (126 MHz, CDCl3)
f2 (ppm)
f1 (ppm)
BnO
BnO
OBn
OCH3
CH3

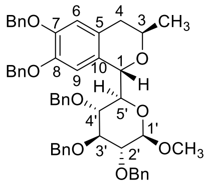

^1H-^1H ROESY (700 MHz, $CDCl_3$)

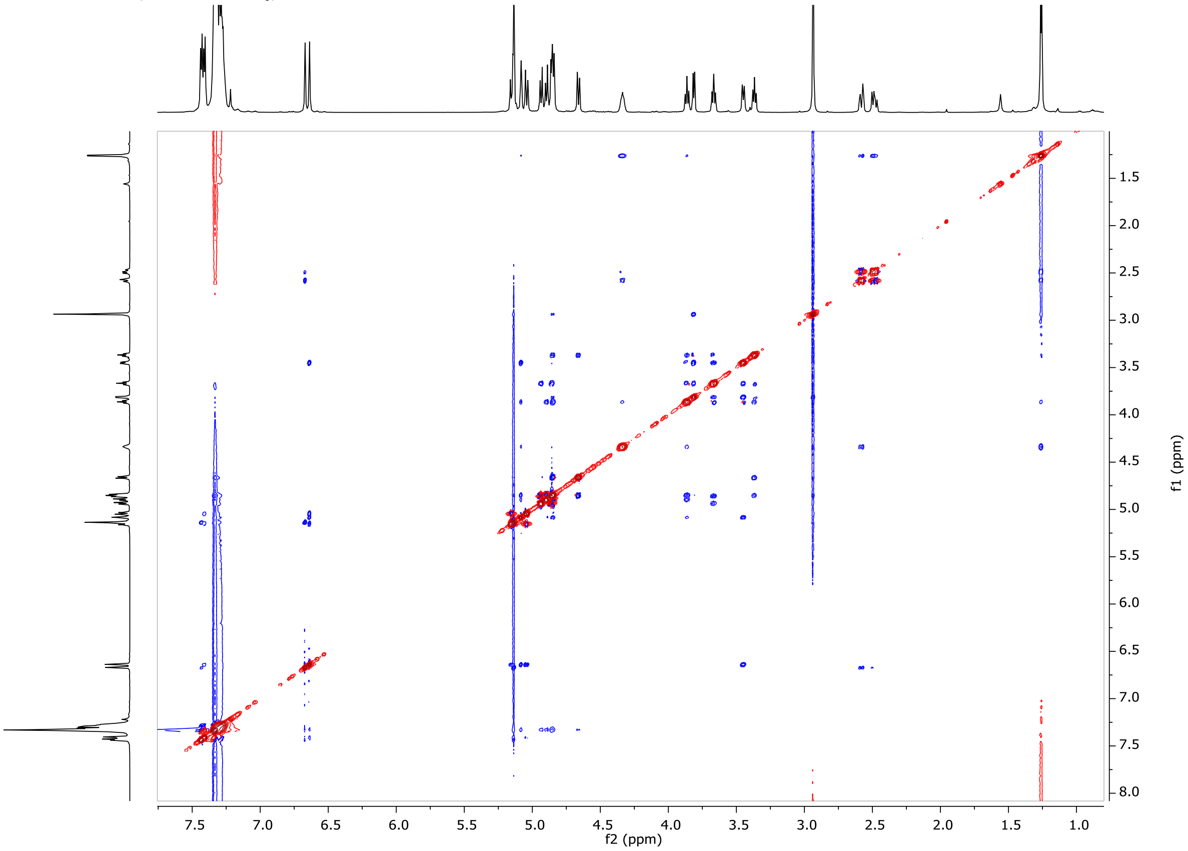

Compound 33

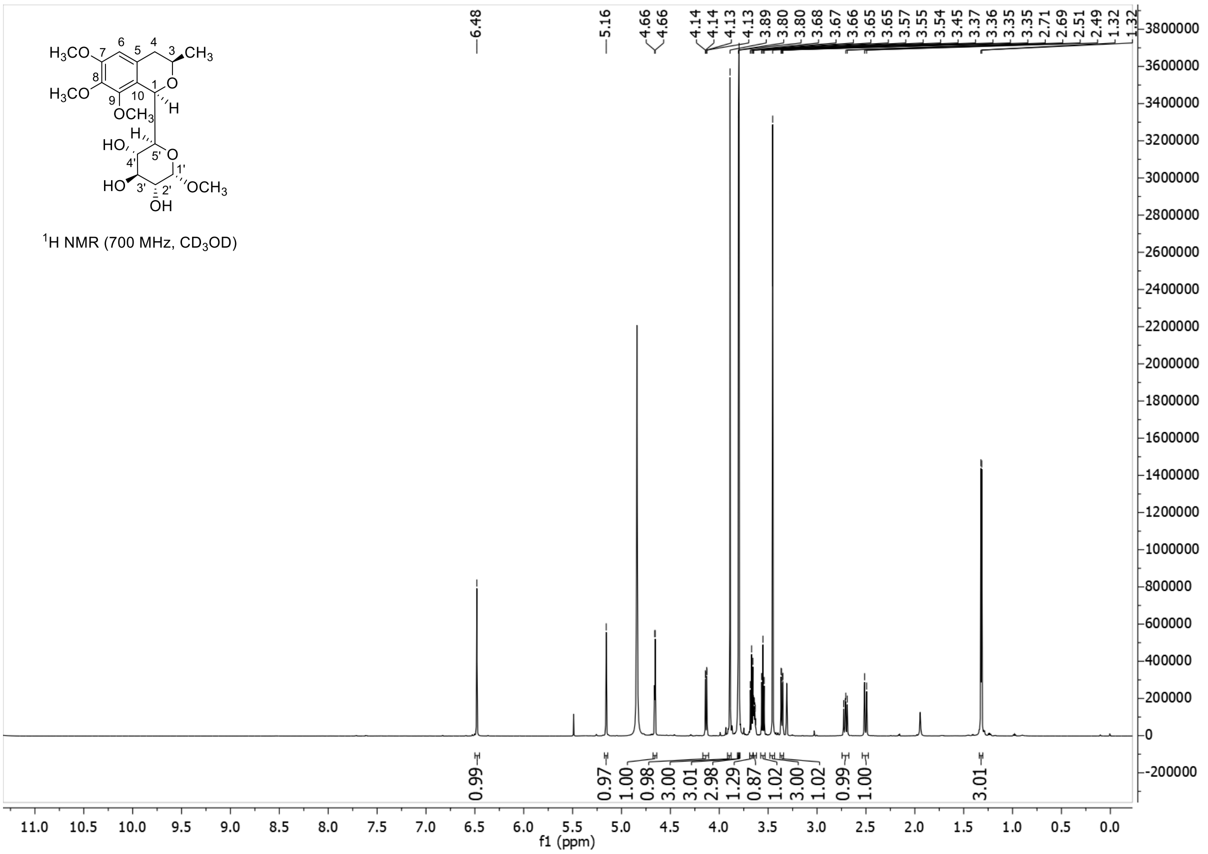

H3CO 7 6 5 4 3 CH3
8 H3CO 9 10 1 O H OCH3
H HO 4' 5' O 1'
HO 3' 2' OCH3
OH
1H NMR (700 MHz, CD3OD)
6.48
5.16
4.66 4.66
4.14 4.14 4.13 4.13 3.89 3.80 3.80 3.68 3.67 3.66 3.65 3.65 3.57 3.55 3.54 3.45 3.37 3.36 3.35 3.35 2.71 2.69 2.51 2.49 1.32 1.32
0.99
0.97 1.00 0.98 3.00 3.01 2.98 1.29 0.87 1.02 3.00 1.02 0.99 1.00
3.01
f1 (ppm)

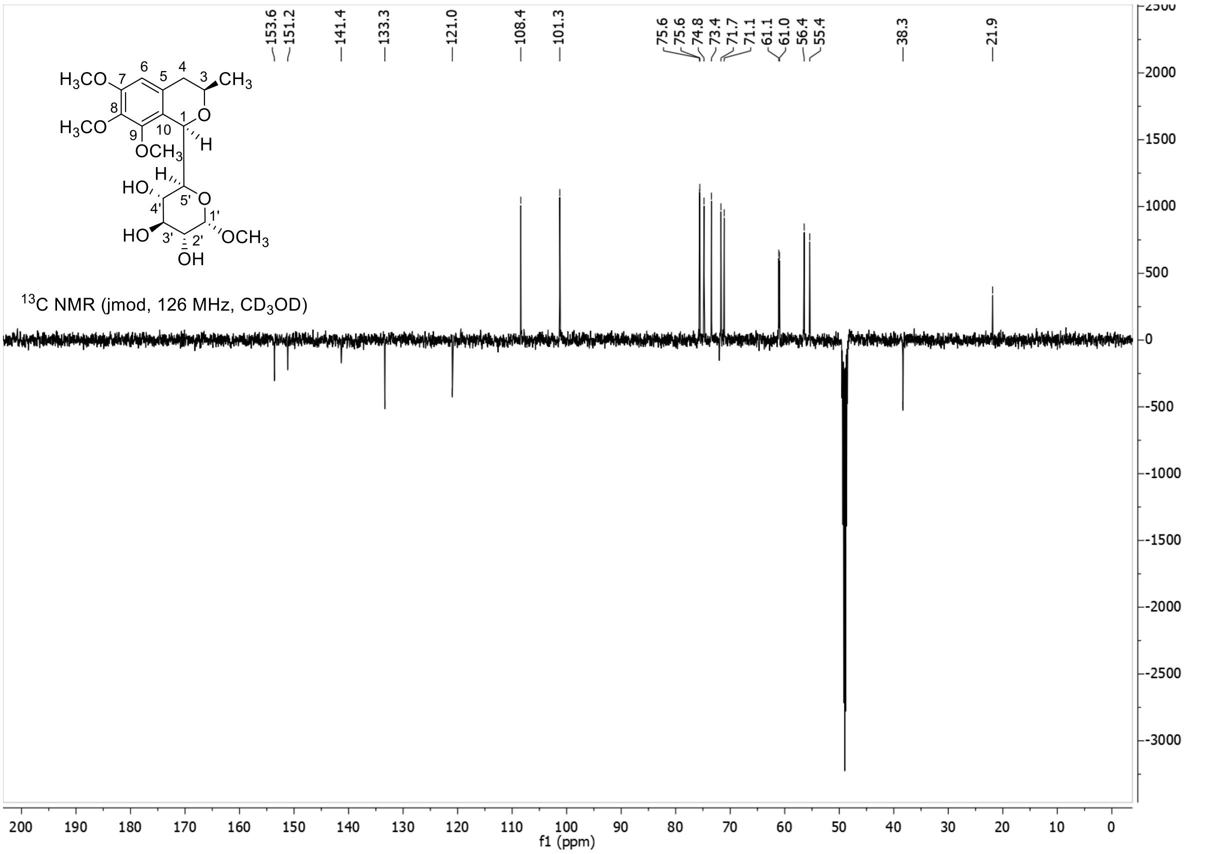

13C NMR (jmod, 126 MHz, CD3OD)
153.6 151.2
141.4
133.3
121.0
108.4
101.3
75.6 75.6 74.8 73.4 71.7 71.1 61.1 61.0 56.4 55.4
38.3
21.9
f1 (ppm)

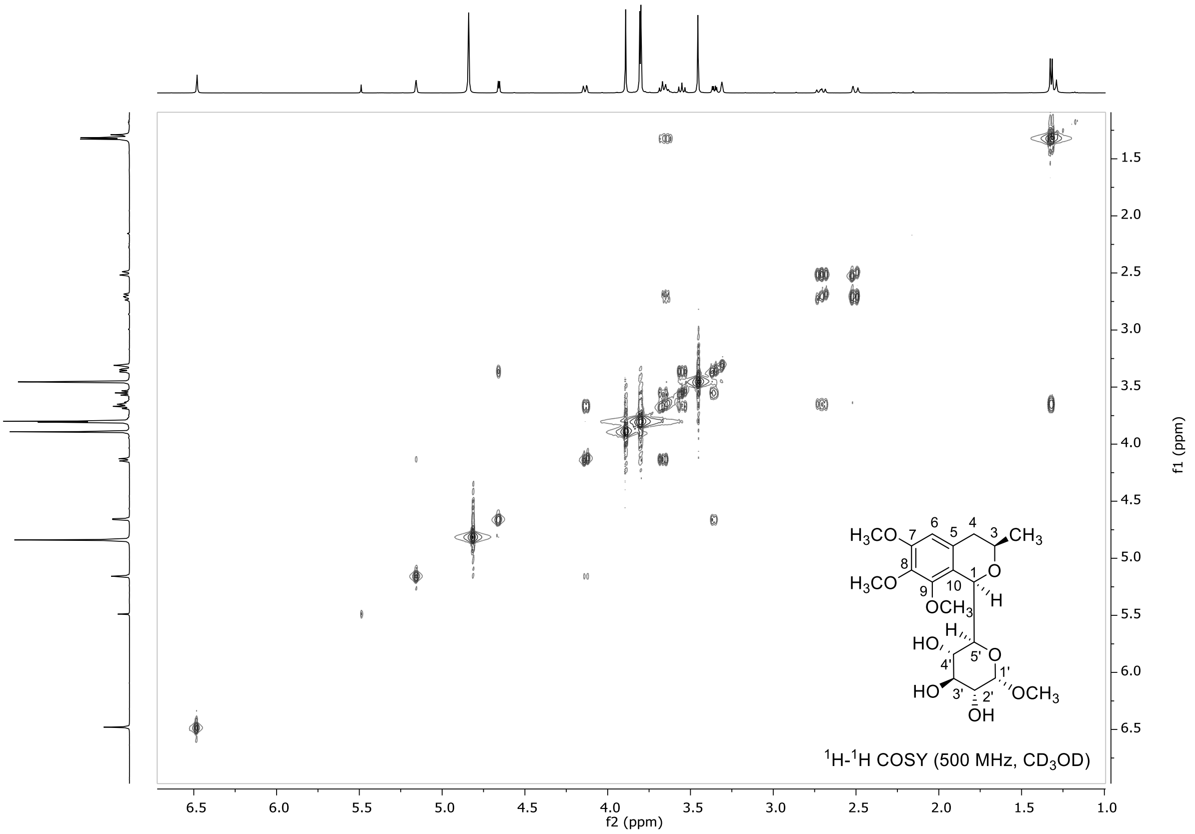

H3CO
H3CO
OCH3
H
HO
HO
OH
OCH3
1H-1H COSY (500 MHz, CD3OD)
f2 (ppm)
f1 (ppm)

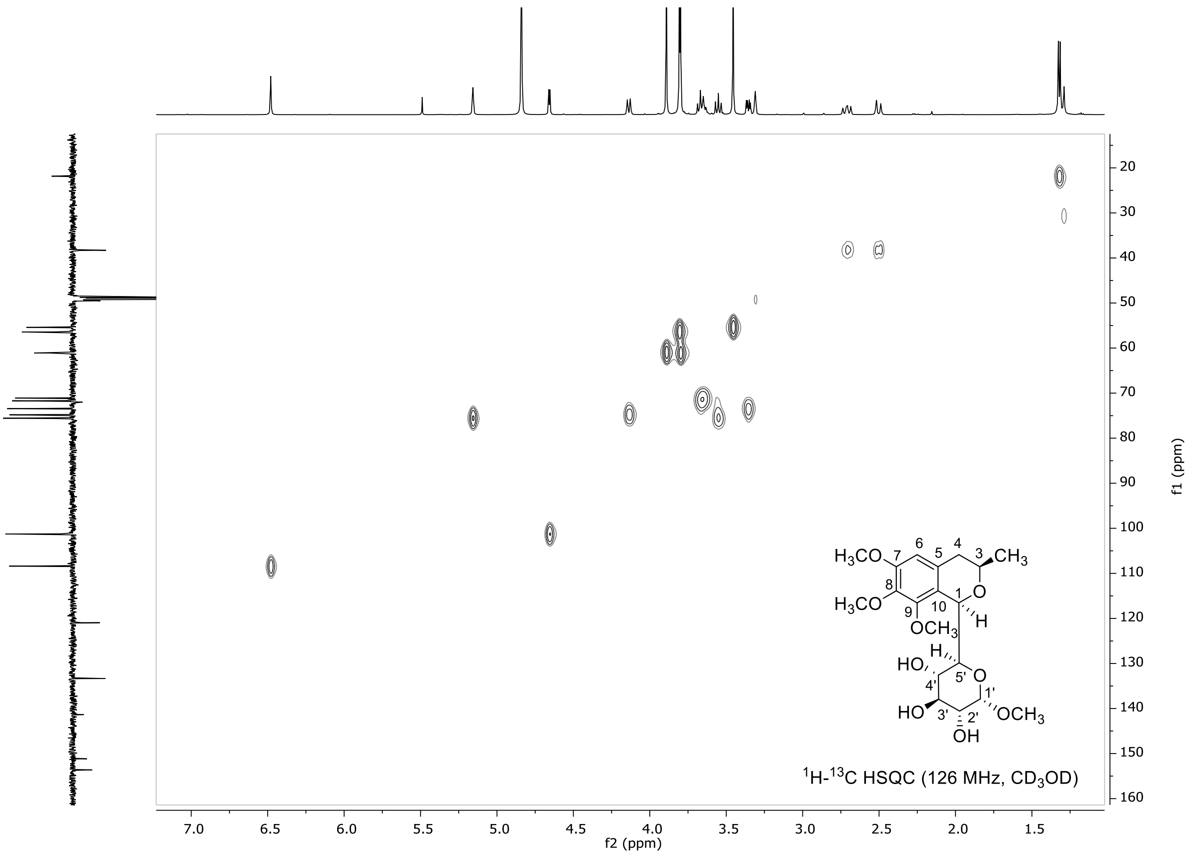

H3CO
H3CO
OCH3
H
HO
HO
OH
OCH3
1H-13C HSQC (126 MHz, CD3OD)
f2 (ppm)
f1 (ppm)

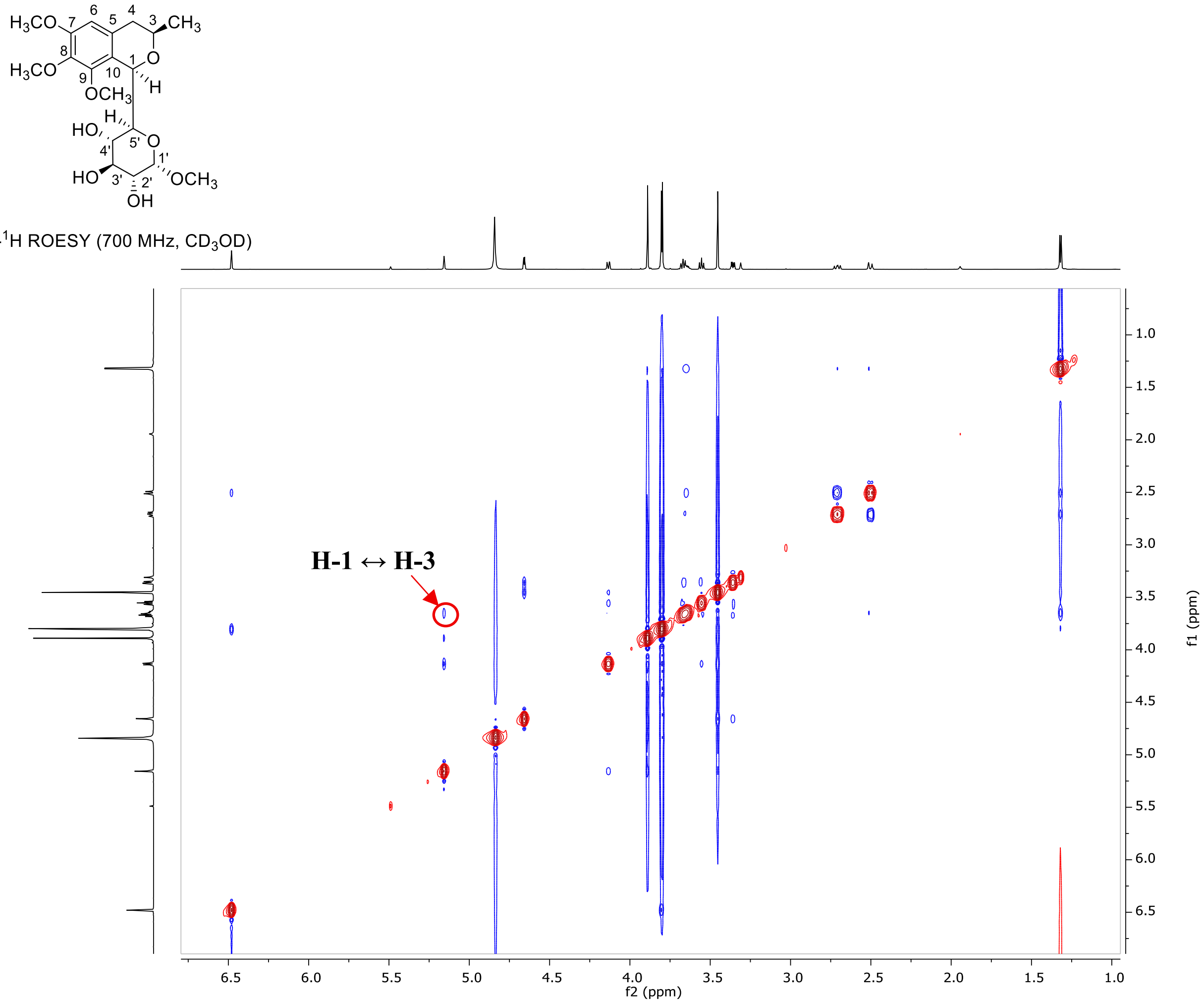

^1H-^1H ROESY (700 MHz, CD_3OD)

Compound 34

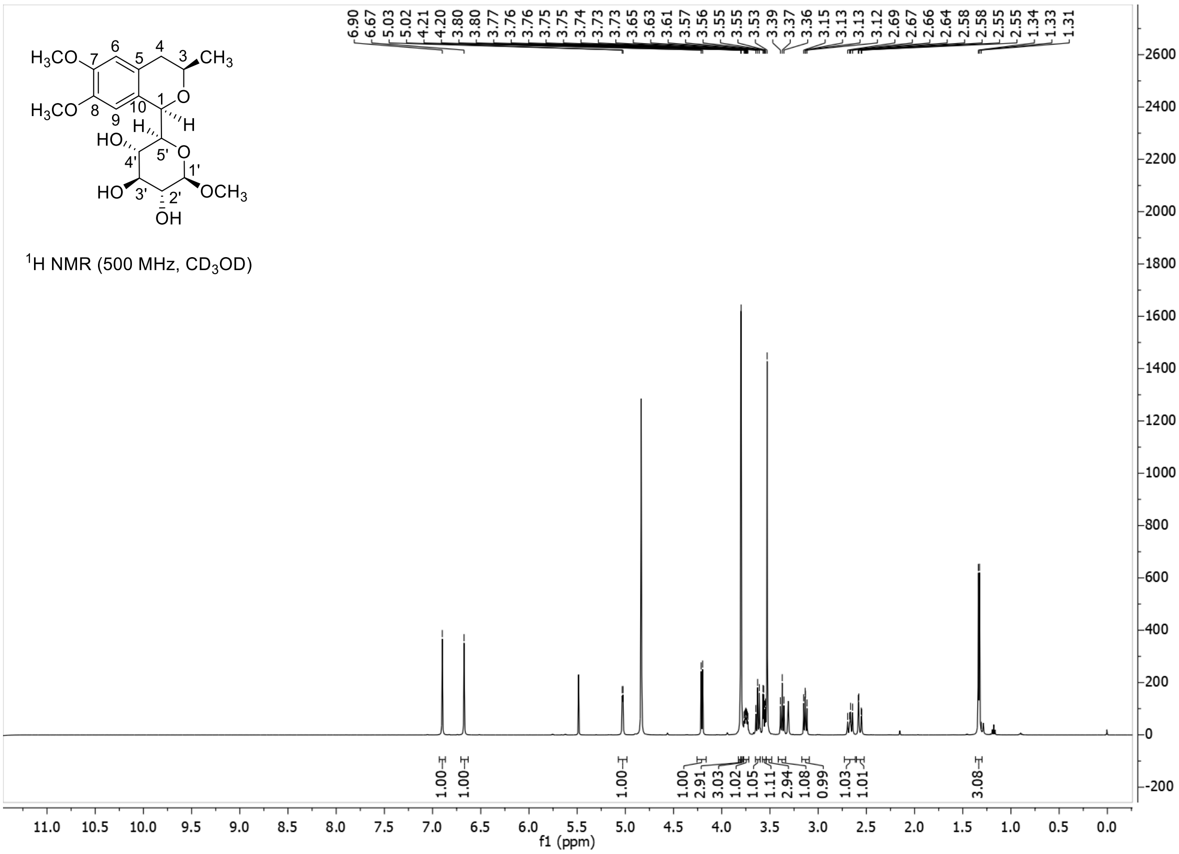

H3CO 7 6 5 4 3 CH3
H3CO 8 9 10 1 O H
H HO 5' O 1'
4' HO 3' 2' OCH3
OH
1H NMR (500 MHz, CD3OD)
6.90 6.67 5.03 5.02 4.21 4.20 3.80 3.80 3.77 3.76 3.76 3.75 3.75 3.74 3.73 3.73 3.65 3.63 3.61 3.57 3.56 3.55 3.55 3.53 3.39 3.37 3.36 3.15 3.13 3.13 3.12 2.69 2.67 2.66 2.64 2.58 2.58 2.55 2.55 1.34 1.33 1.31
1.00 1.00 1.00 1.00 2.91 3.03 1.02 1.05 1.11 2.94 1.08 0.99 1.03 1.01 3.08
f1 (ppm)

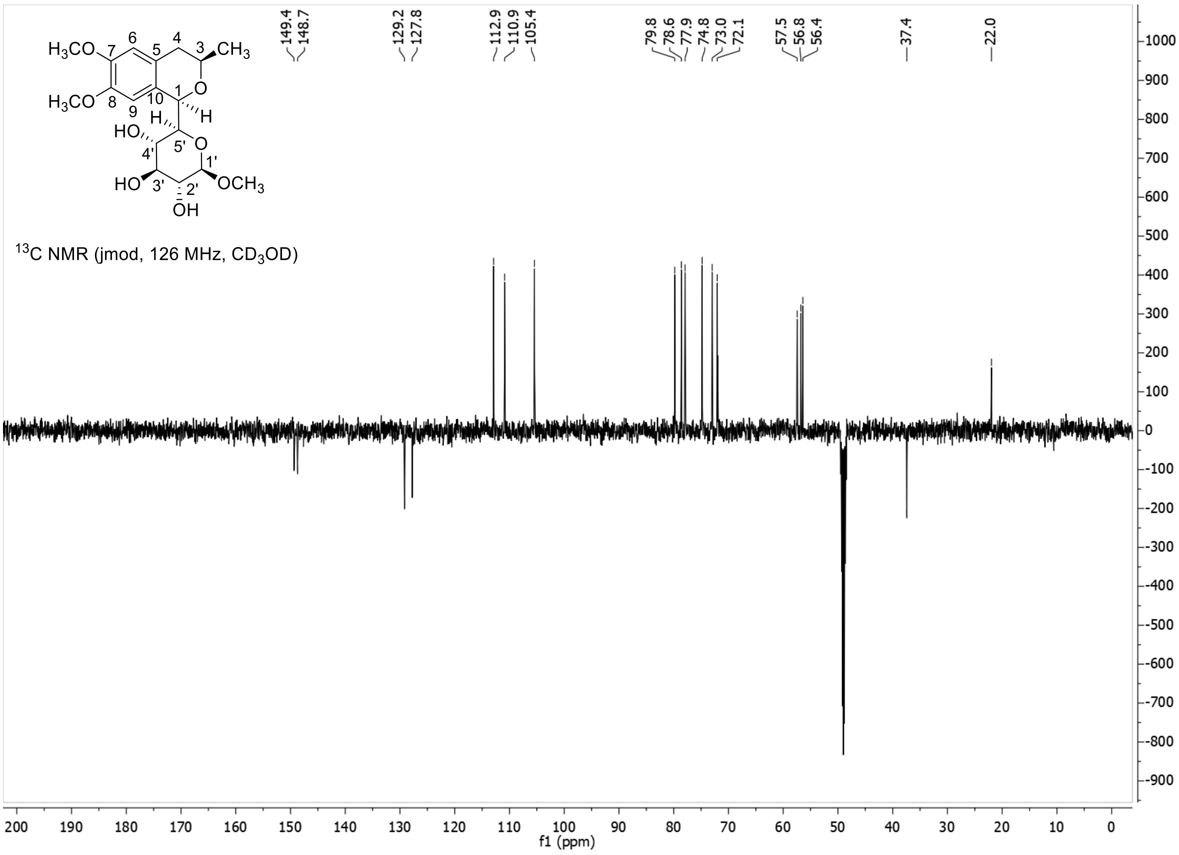

H3CO 7 6 5 4 3 CH3
H3CO 8 9 10 1 O H
H HO 5' O 1'
4' HO 3' 2' OCH3
OH
13C NMR (jmod, 126 MHz, CD3OD)
149.4 148.7 129.2 127.8 112.9 110.9 105.4 79.8 78.6 77.9 74.8 73.0 72.1 57.5 56.8 56.4 37.4 22.0
f1 (ppm)

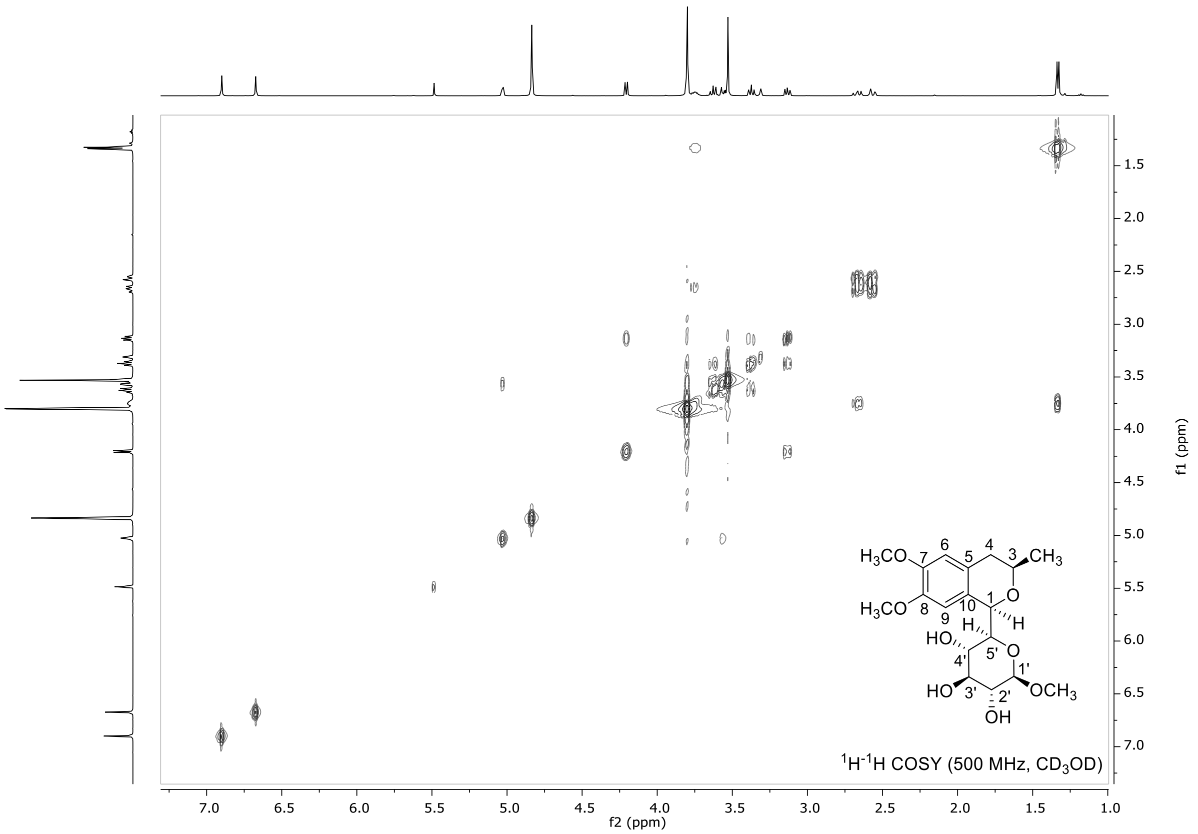
H3CO 7 6 5 4 3 CH3
H3CO 8 9 10 1 O H
H HO 4' 5' O 1'
HO 3' 2' OCH3
OH
1H-1H COSY (500 MHz, CD3OD)
f1 (ppm)
f2 (ppm)

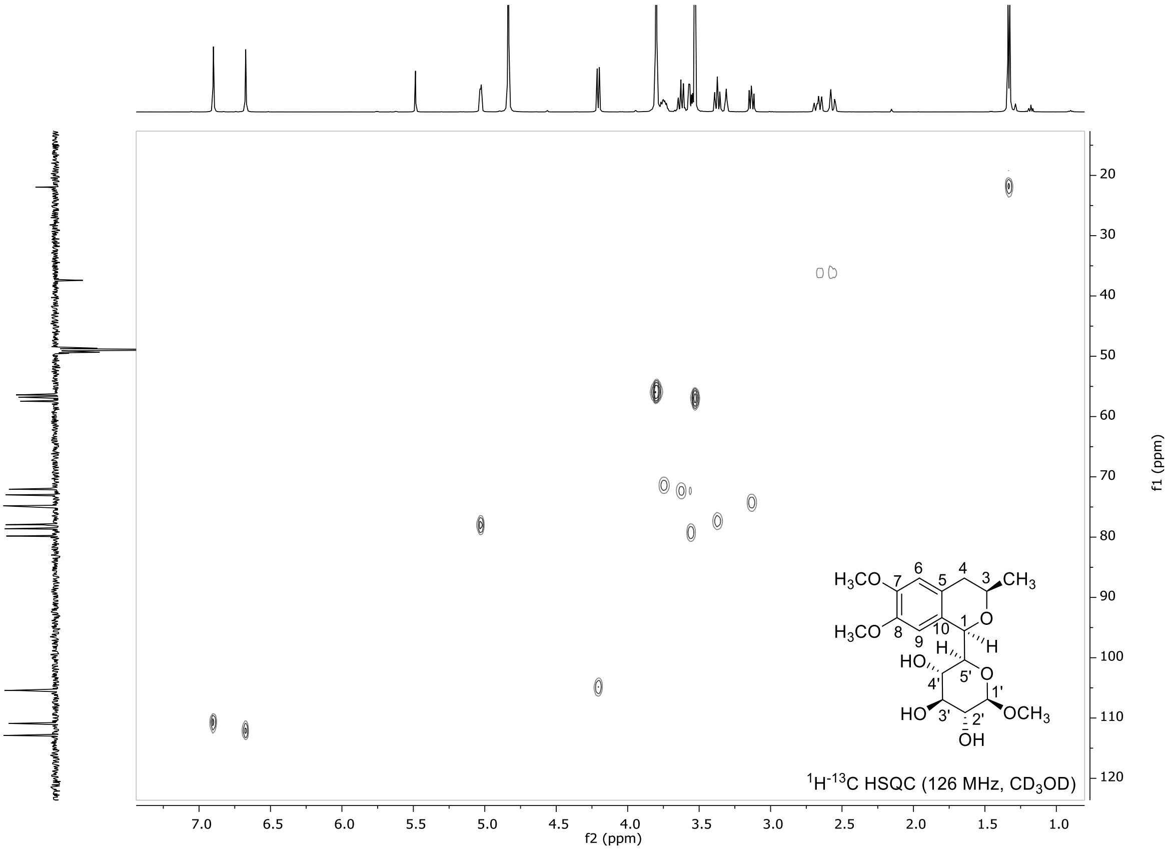
H3CO 7 6 5 4 3 CH3
H3CO 8 9 10 1 O H
H HO 4' 5' O 1'
HO 3' 2' OCH3
OH
1H-13C HSQC (126 MHz, CD3OD)
f1 (ppm)
f2 (ppm)

Compound 35

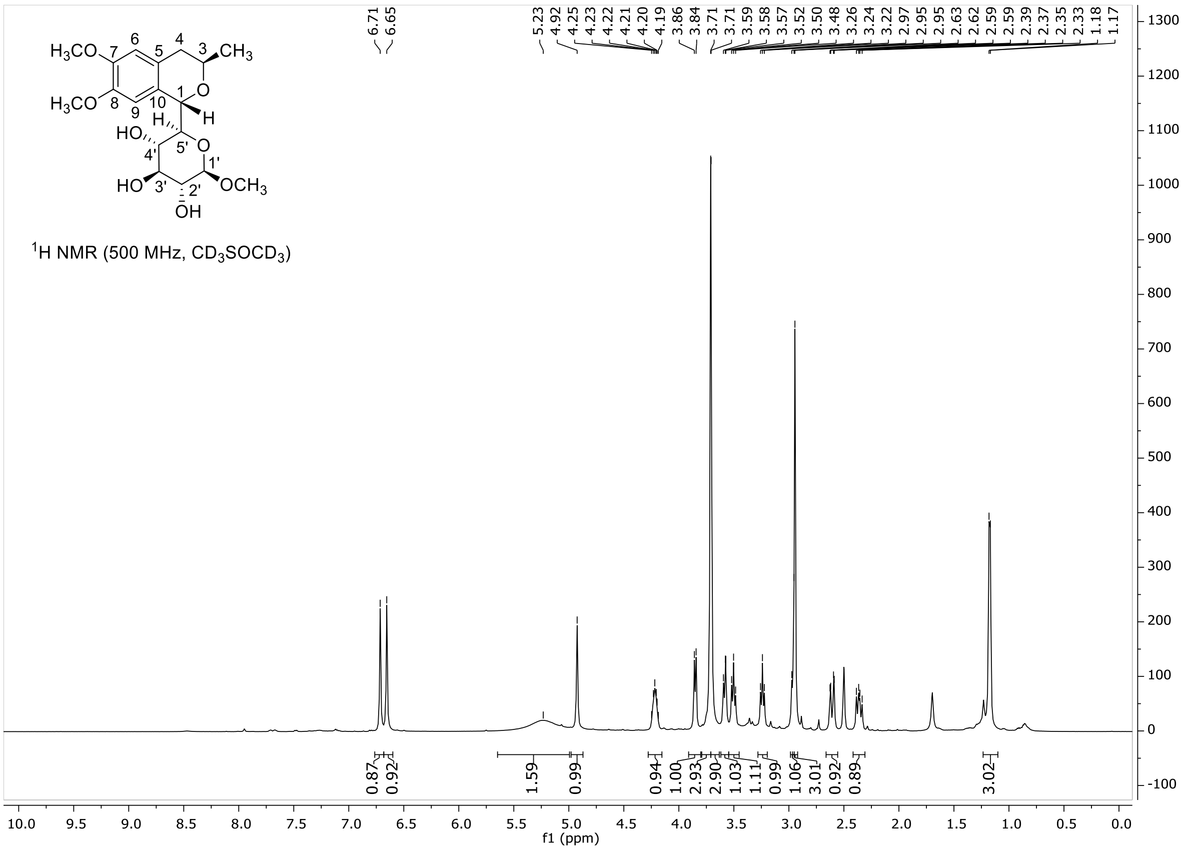

^{1}H NMR (500 MHz, CD$_3$SOCD$_3$)
f1 (ppm)

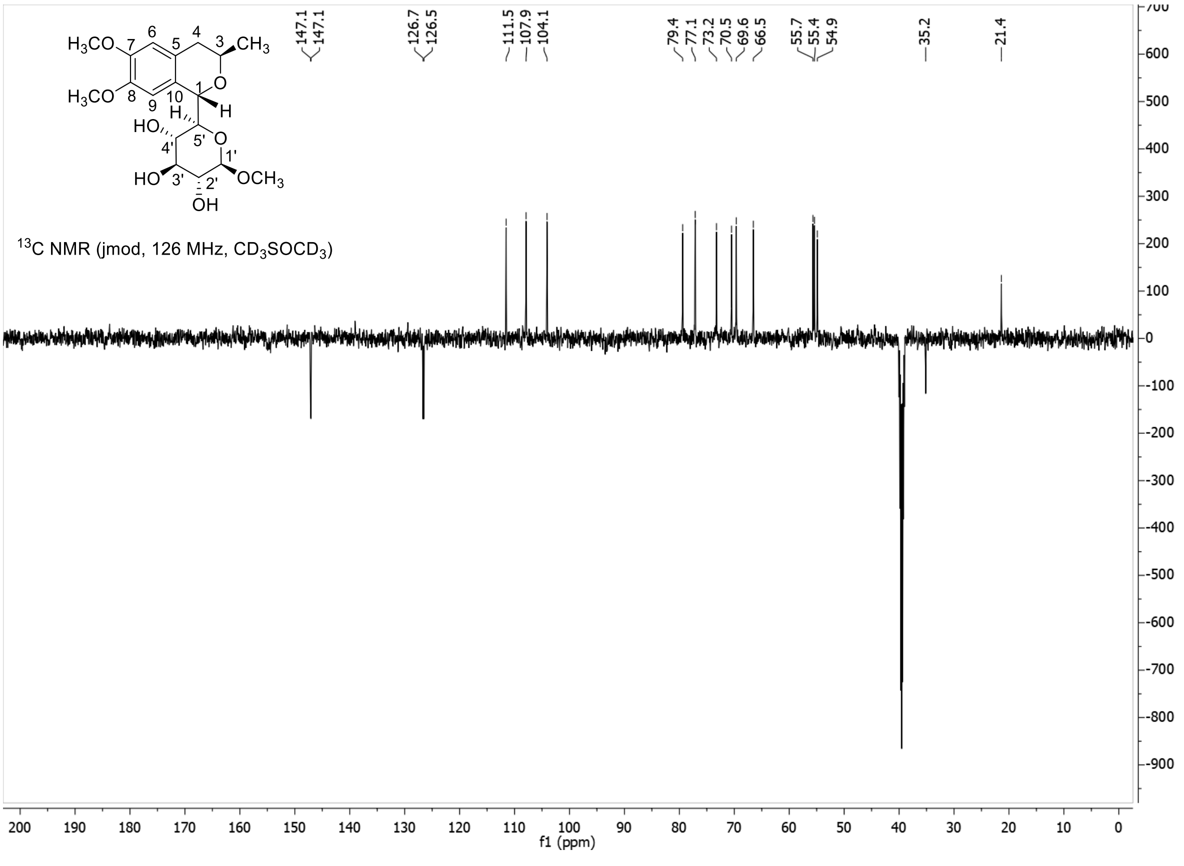

^{13}C NMR (jmod, 126 MHz, CD$_3$SOCD$_3$)
f1 (ppm)

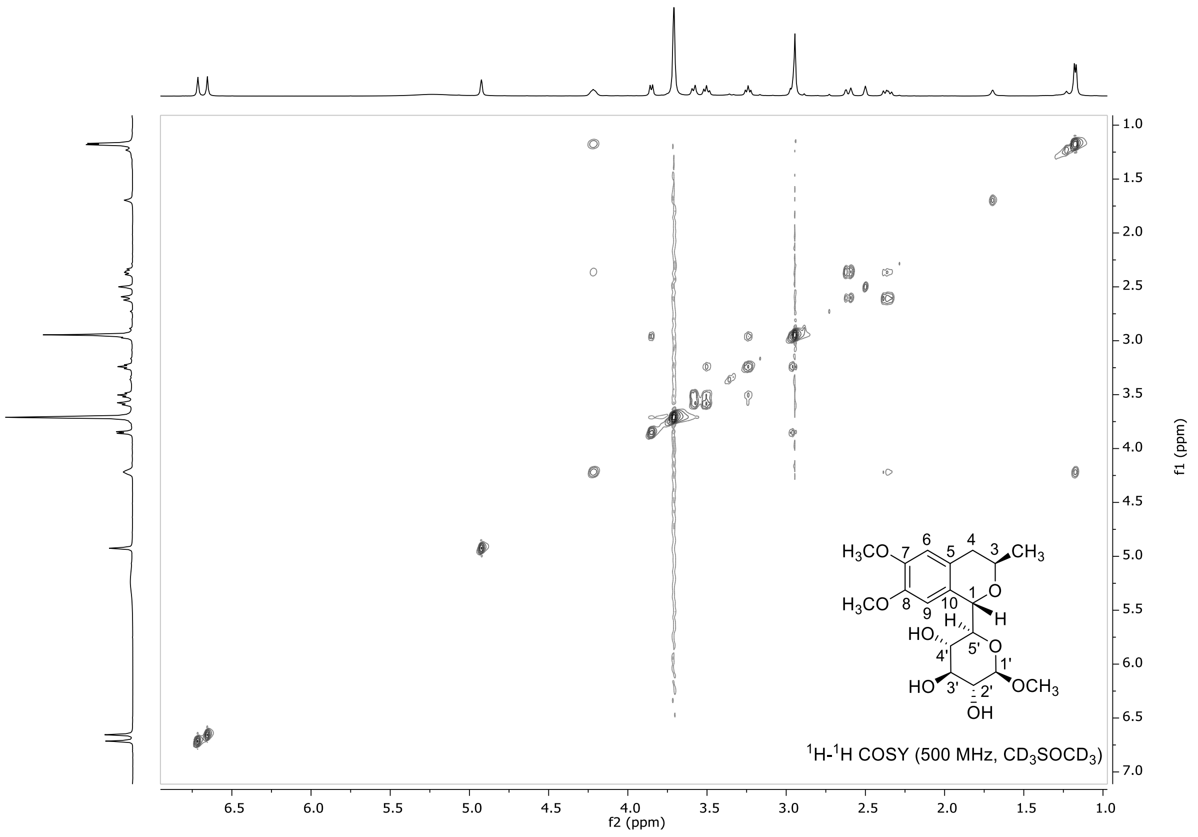
^{1}H-^{1}H COSY (500 MHz, CD_3SOCD_3)
f1 (ppm)
f2 (ppm)
H3CO
H3CO
HO
HO
OH
OCH3
CH3

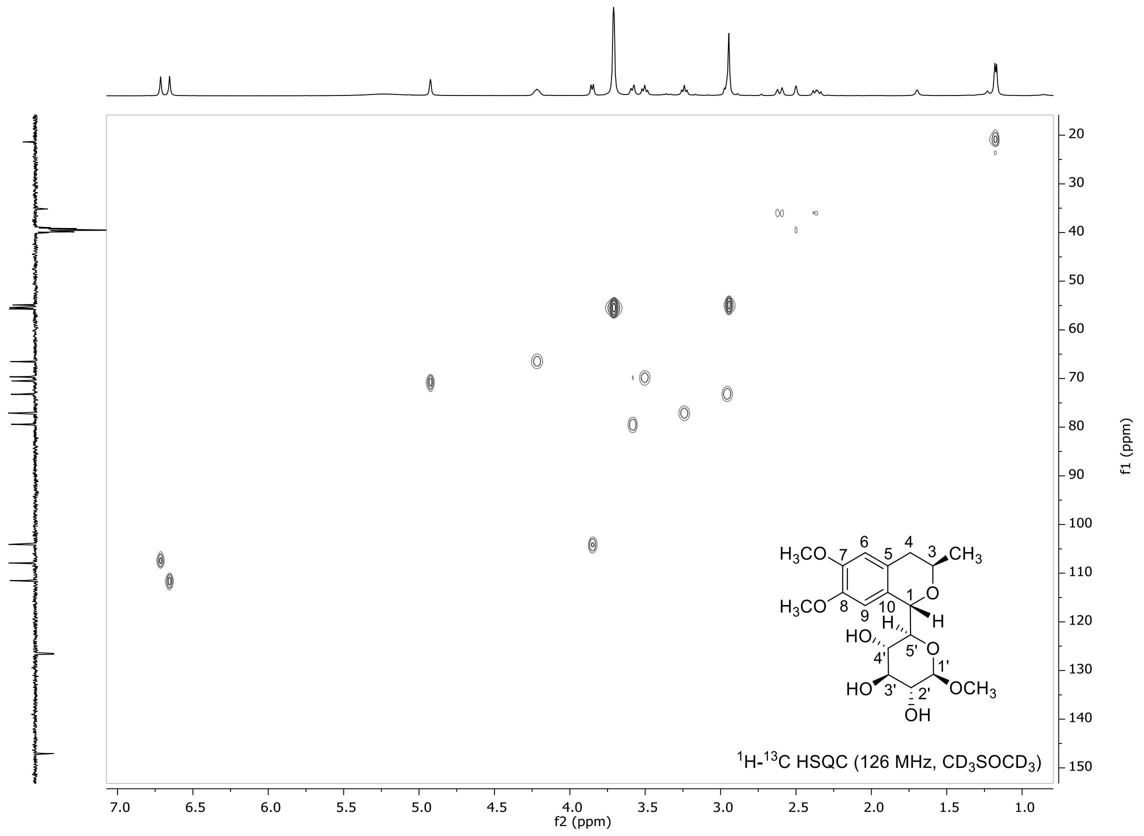
^{1}H-^{13}C HSQC (126 MHz, CD_3SOCD_3)
f1 (ppm)
f2 (ppm)
H3CO
H3CO
HO
HO
OH
OCH3
CH3

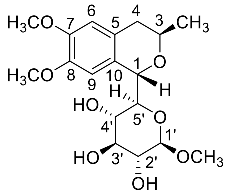

^{1}H-^{13}C HMBC (126 MHz, CD_3SOCD_3)

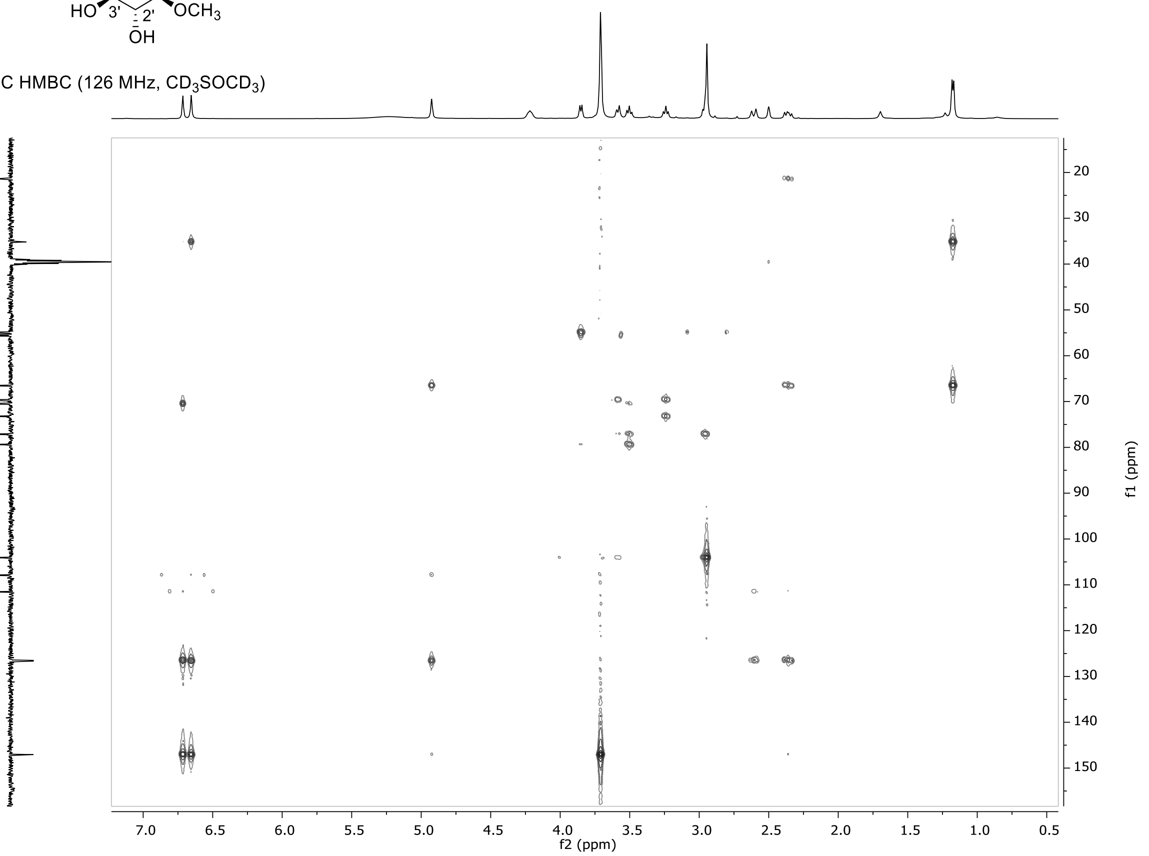

Compound 36

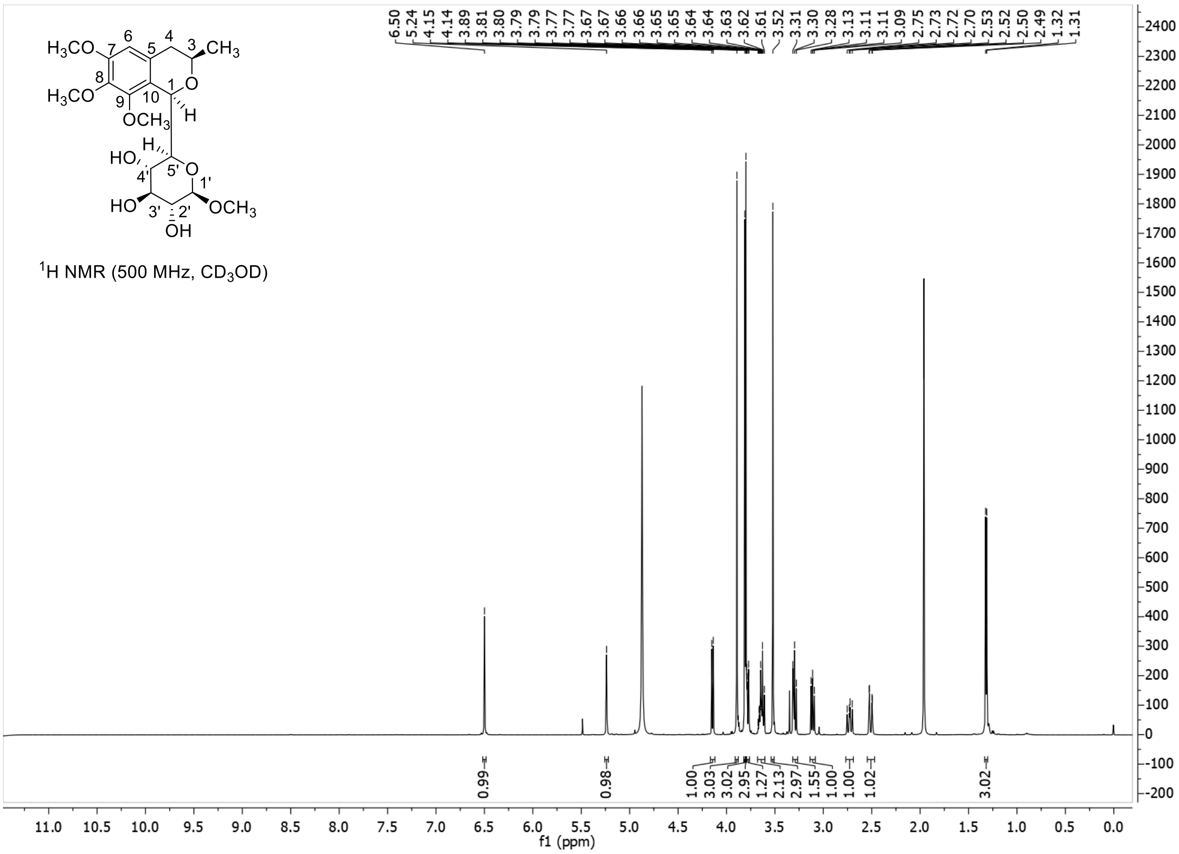
6.50 5.24 4.15 4.14 3.89 3.81 3.80 3.79 3.79 3.77 3.77 3.67 3.66 3.66 3.65 3.65 3.64 3.64 3.63 3.62 3.61 3.52 3.31 3.30 3.28 3.13 3.11 3.11 3.09 2.75 2.73 2.72 2.70 2.53 2.52 2.50 2.49 1.32 1.31
^{1}H NMR (500 MHz, CD_3OD)
0.99 0.98 1.00 3.03 3.02 2.95 1.27 2.13 2.97 1.55 1.00 1.02 3.02
f1 (ppm)

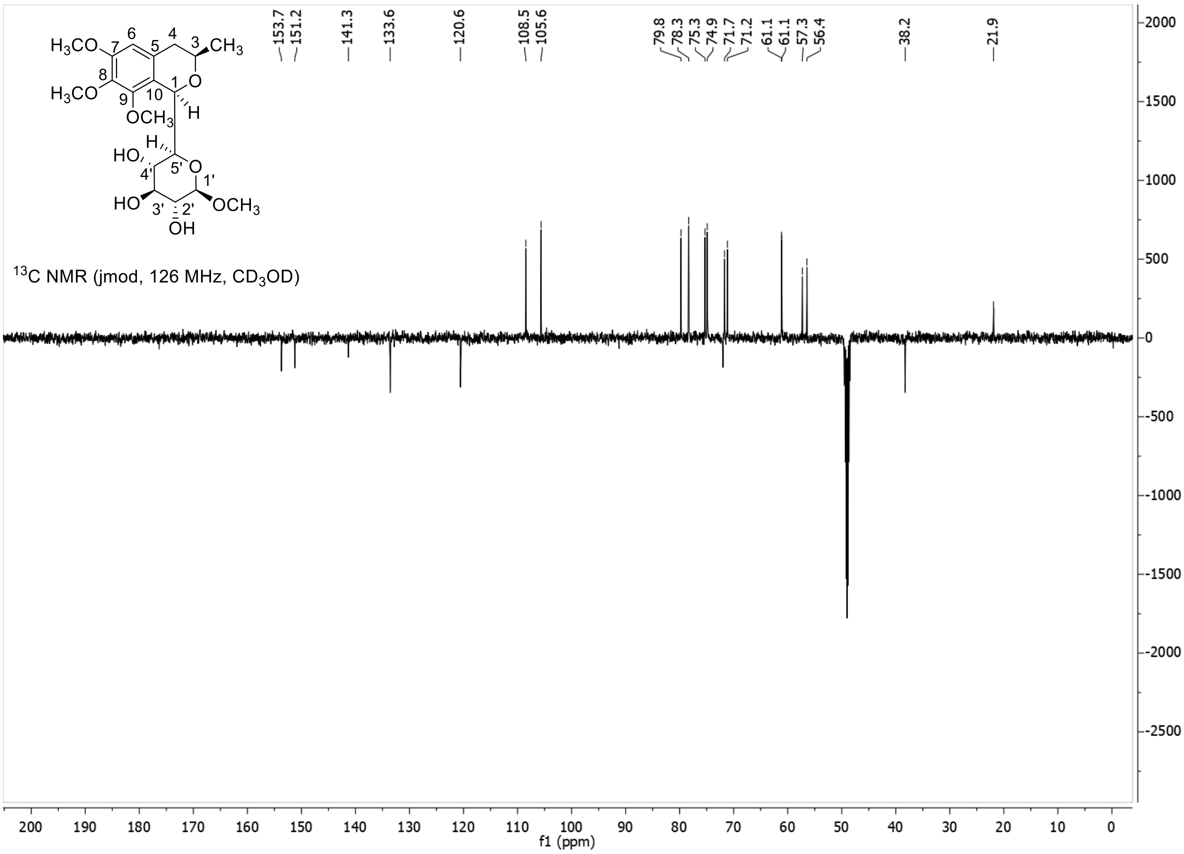
153.7 151.2 141.3 133.6 120.6 108.5 105.6 79.8 78.3 75.3 74.9 71.7 71.2 61.1 61.1 57.3 56.4 38.2 21.9
^{13}C NMR (jmod, 126 MHz, CD_3OD)
f1 (ppm)

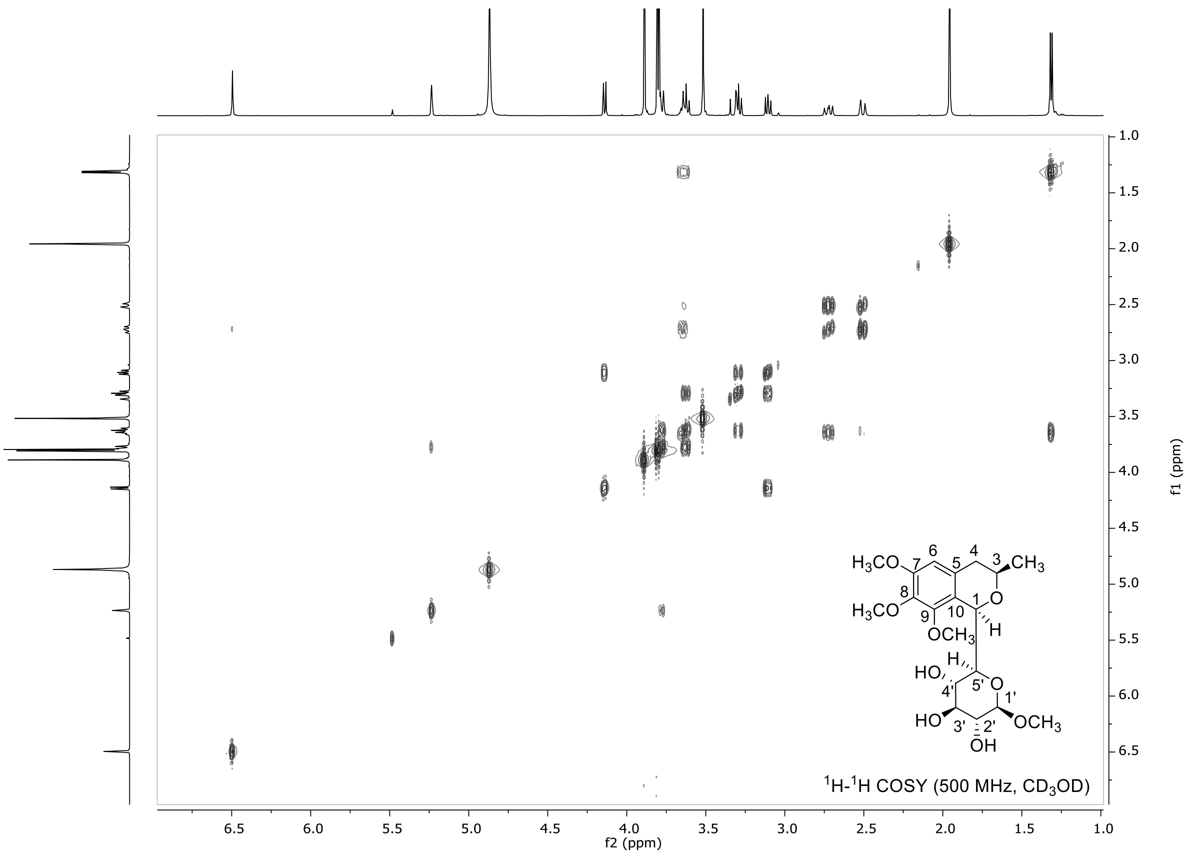
H3CO
H3CO
OCH3
H
HO
HO
OH
OCH3
CH3
O
1H-1H COSY (500 MHz, CD3OD)
f1 (ppm)
f2 (ppm)

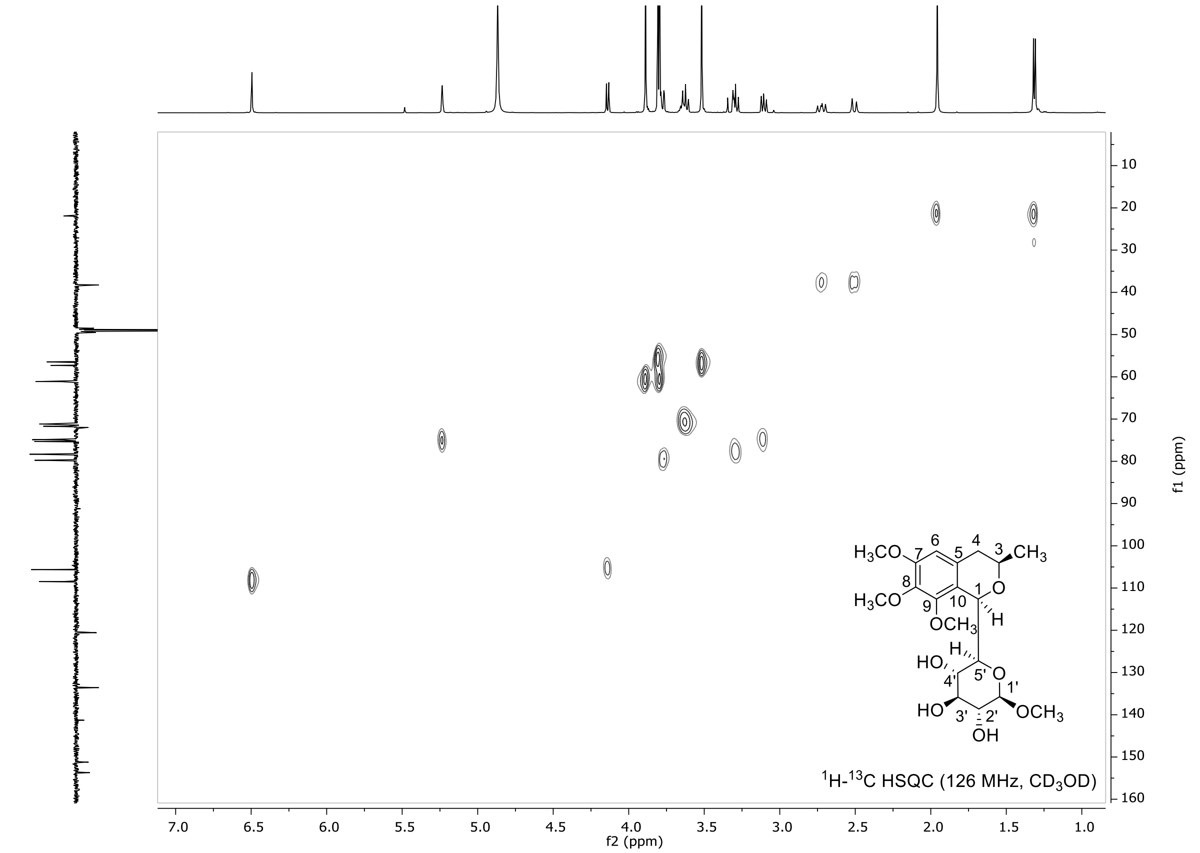
H3CO
H3CO
OCH3
H
HO
HO
OH
OCH3
CH3
O
1H-13C HSQC (126 MHz, CD3OD)
f1 (ppm)
f2 (ppm)

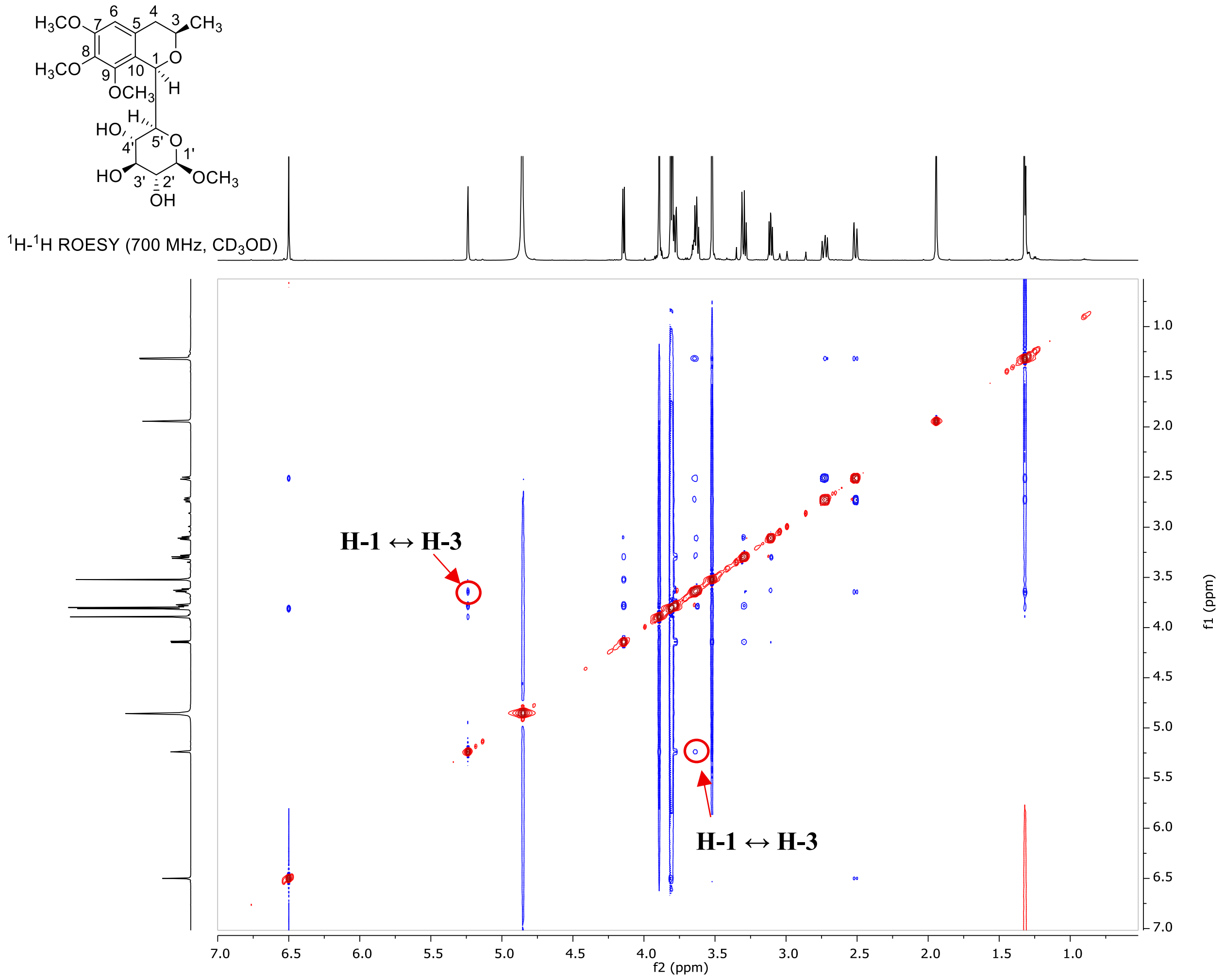

H3CO
H3CO
OCH3
HO
HO
OH
OCH3
CH3
H
1H-1H ROESY (700 MHz, CD3OD)
H-1 ↔ H-3
H-1 ↔ H-3
f2 (ppm)
f1 (ppm)

Compound 37

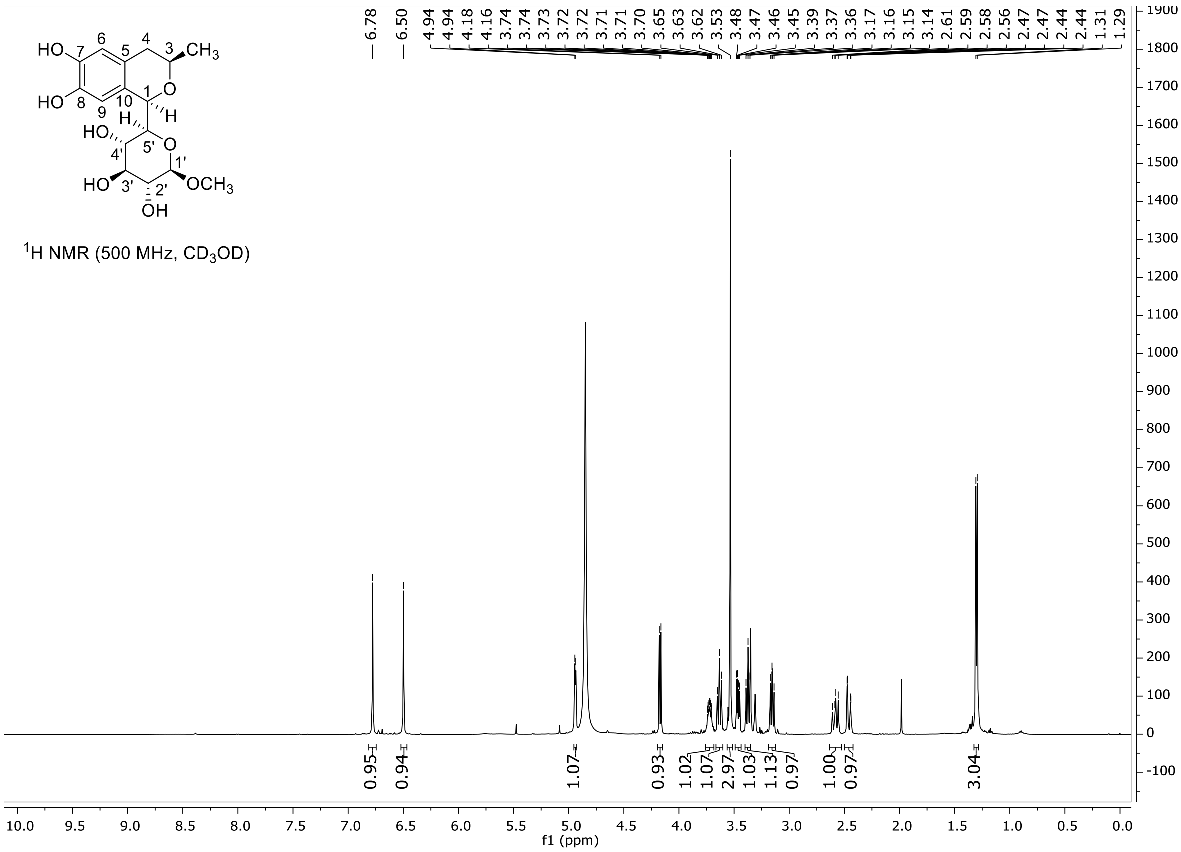

6.78
6.50
4.94
4.94
4.18
4.16
3.74
3.74
3.73
3.72
3.72
3.71
3.71
3.70
3.65
3.63
3.62
3.53
3.48
3.47
3.46
3.45
3.39
3.37
3.36
3.17
3.16
3.15
3.14
2.61
2.59
2.58
2.56
2.47
2.47
2.44
2.44
1.31
1.29
^{1}H NMR (500 MHz, CD_3OD)
0.95
0.94
1.07
0.93
1.02
1.07
2.97
1.03
1.13
0.97
1.00
0.97
3.04
f1 (ppm)

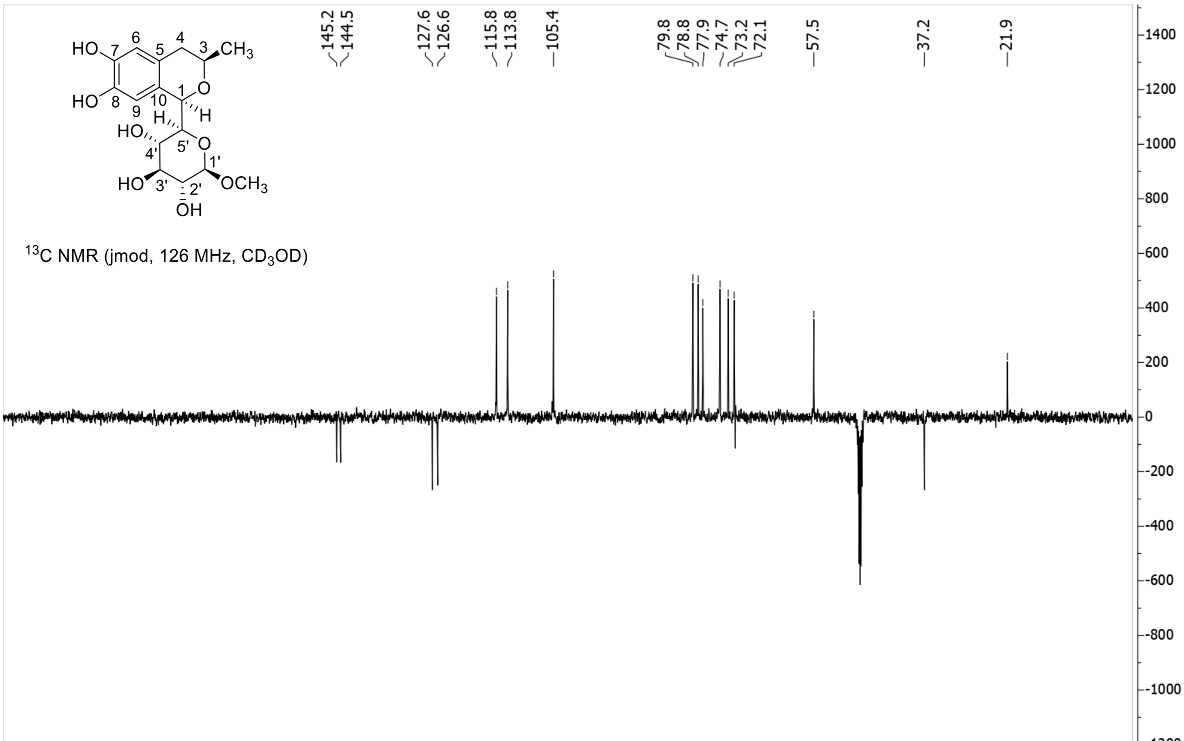

145.2
144.5
127.6
126.6
115.8
113.8
105.4
79.8
78.8
77.9
74.7
73.2
72.1
57.5
37.2
21.9
^{13}C NMR (jmod, 126 MHz, CD_3OD)
f1 (ppm)

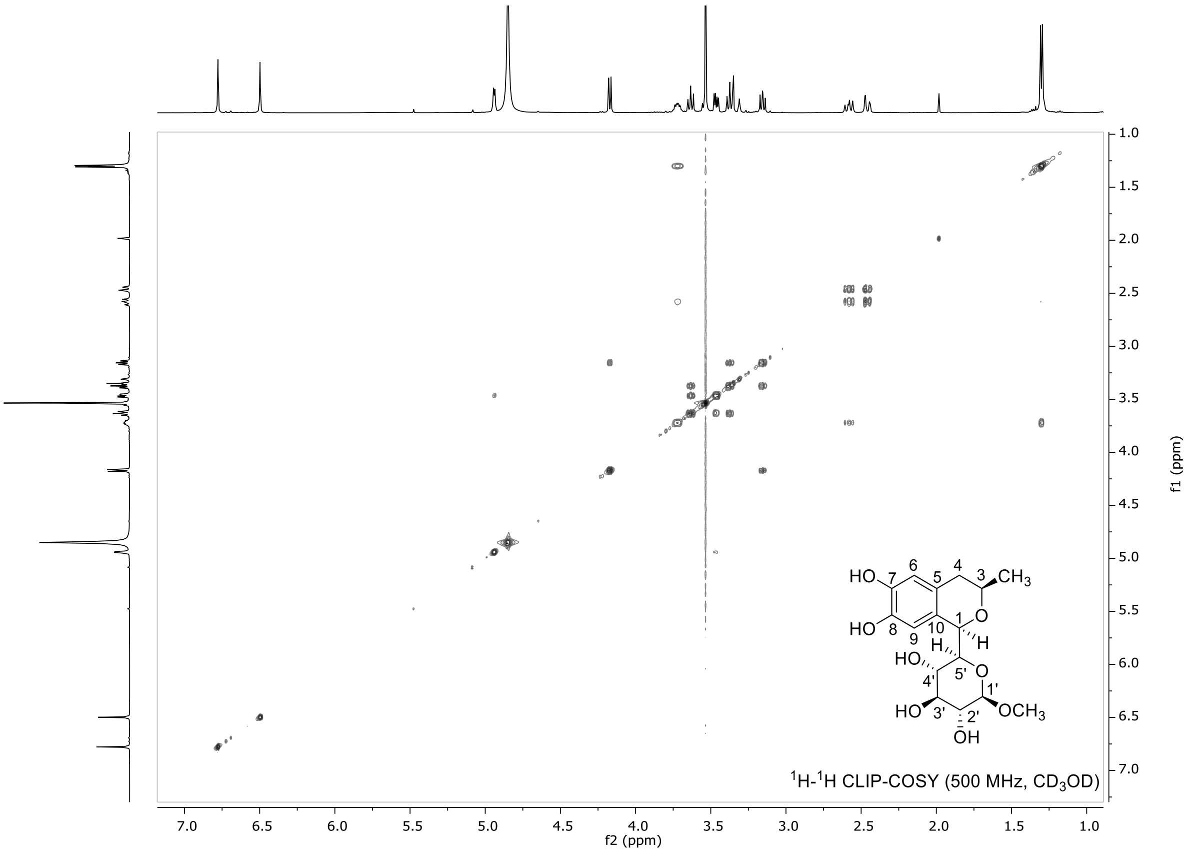
f1 (ppm)
f2 (ppm)
HO 7 6 5 4 3 CH3
HO 8 9 10 1 O H
H HO 5' O 4' 1'
HO 3' 2' OCH3
OH
1H-1H CLIP-COSY (500 MHz, CD3OD)

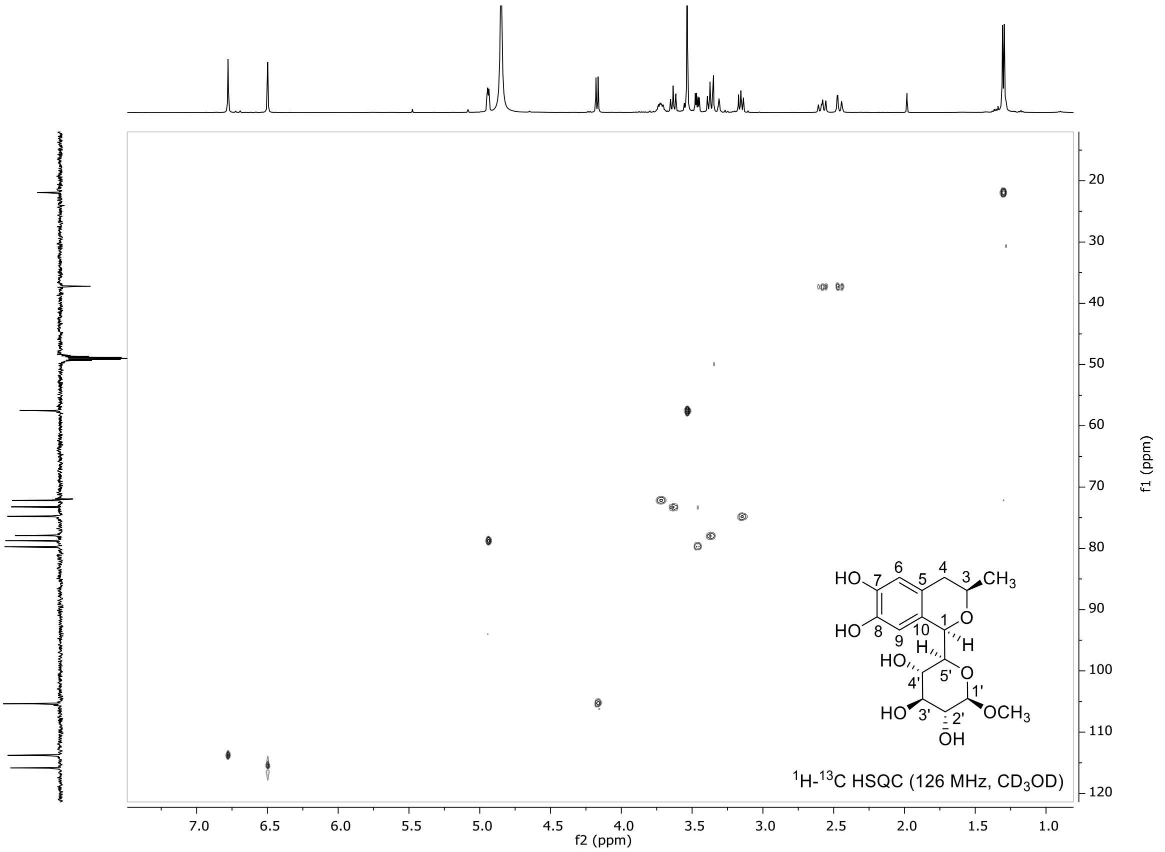
f1 (ppm)
f2 (ppm)
HO 7 6 5 4 3 CH3
HO 8 9 10 1 O H
H HO 5' O 4' 1'
HO 3' 2' OCH3
OH
1H-13C HSQC (126 MHz, CD3OD)

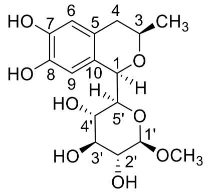

^{1}H-^{13}C HMBC (126 MHz, CD_3OD)

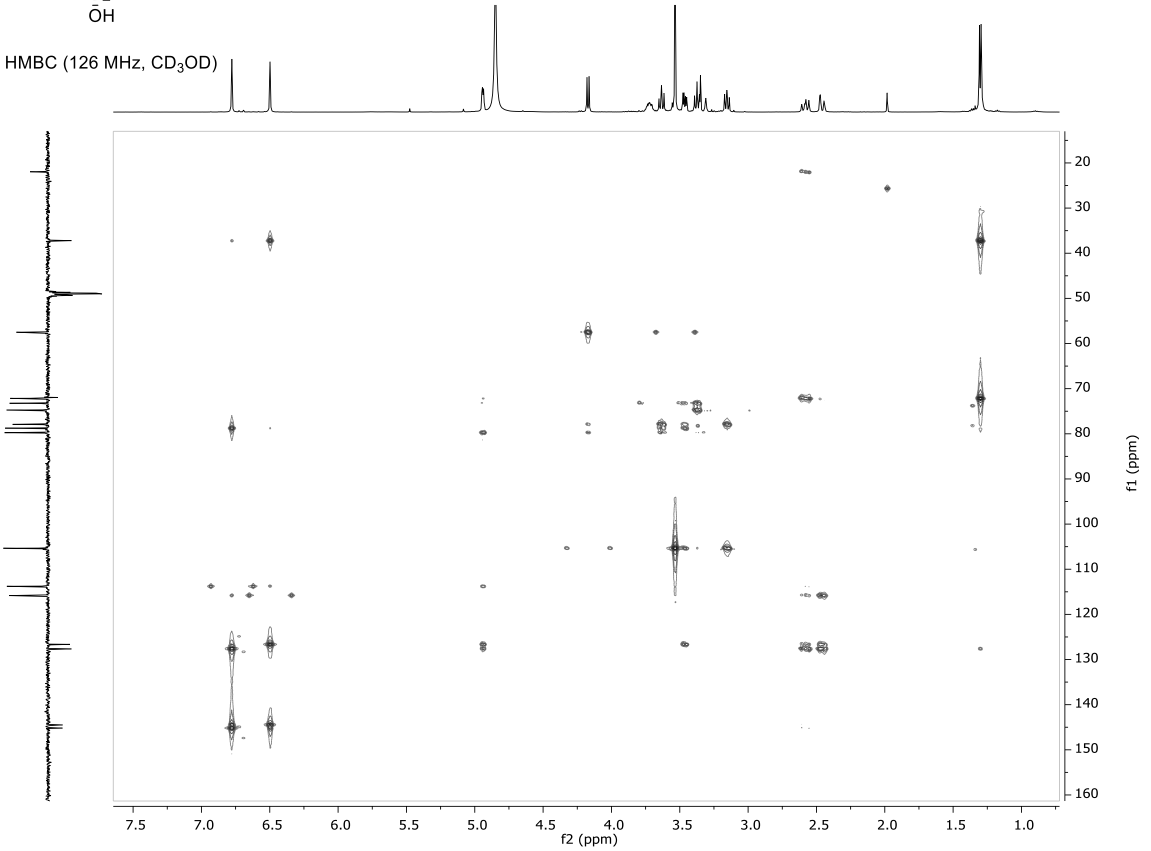

Compound 38.2

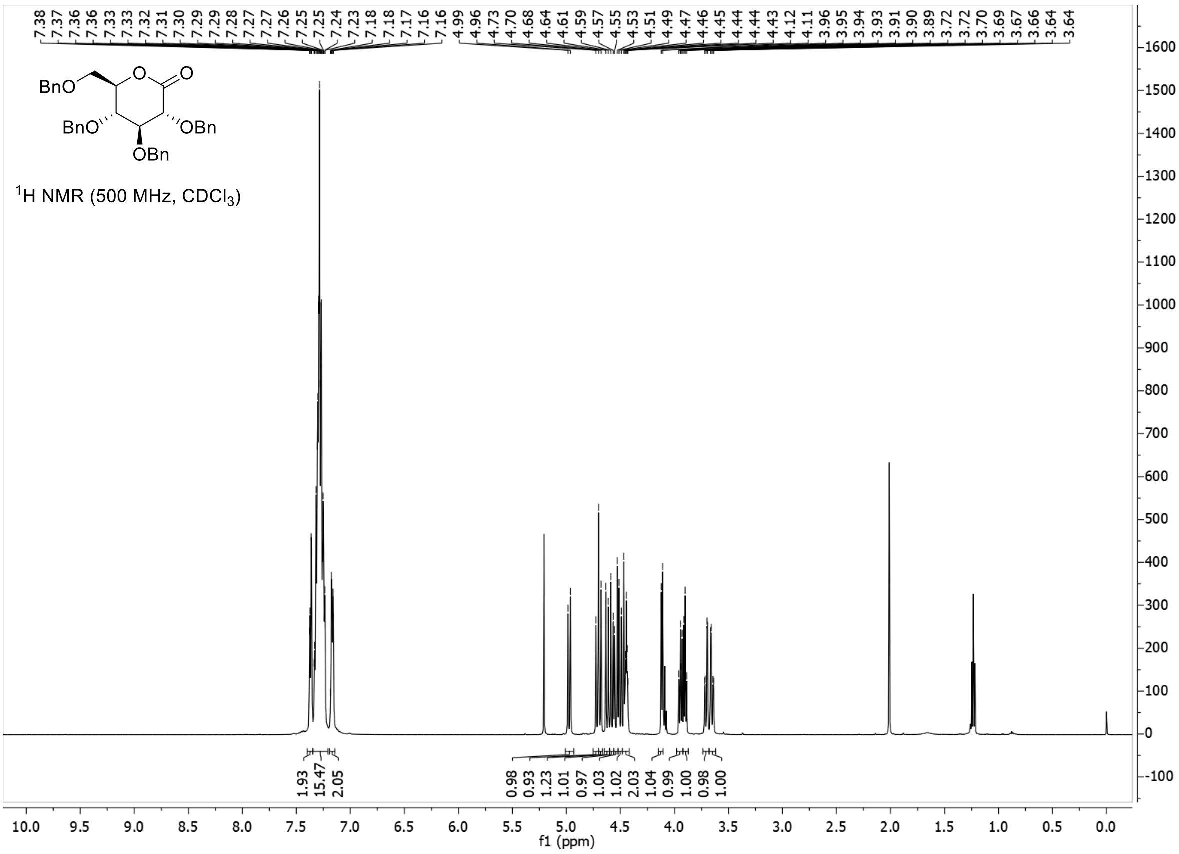

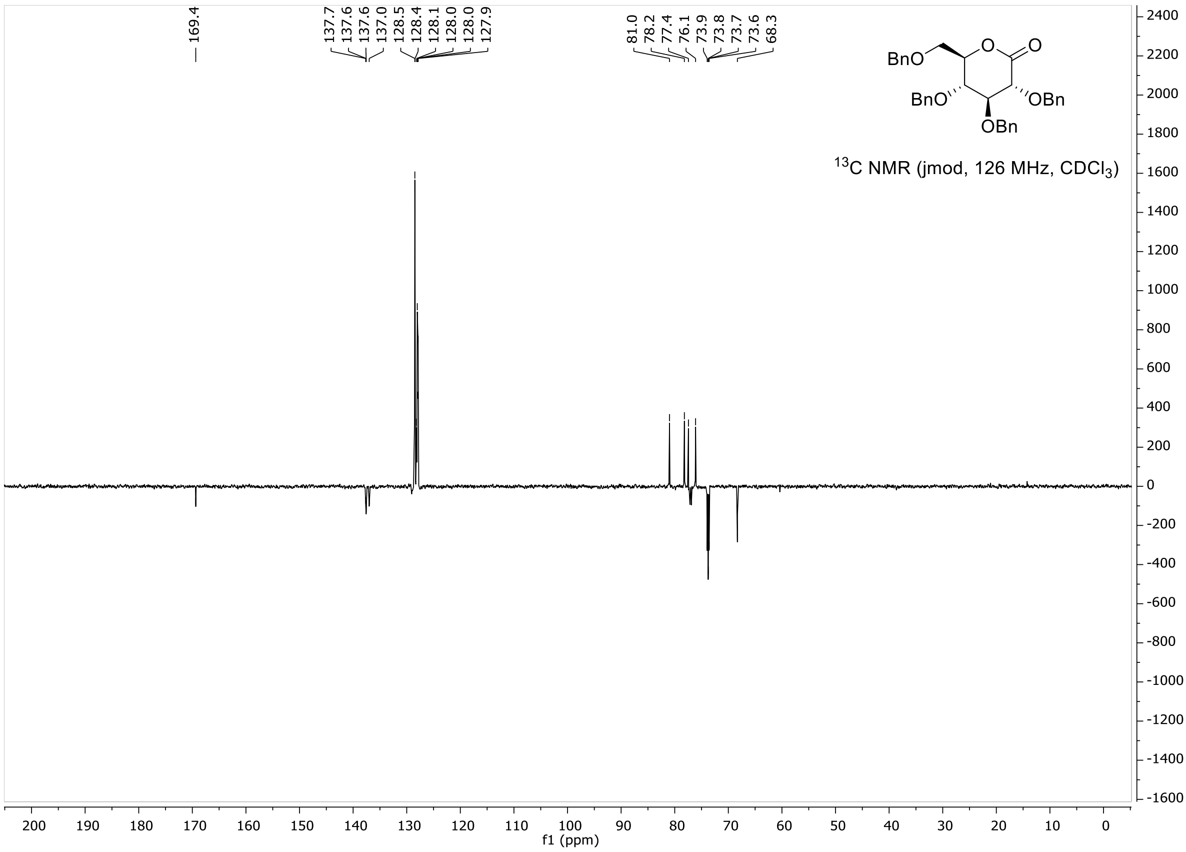

Compound 38.3

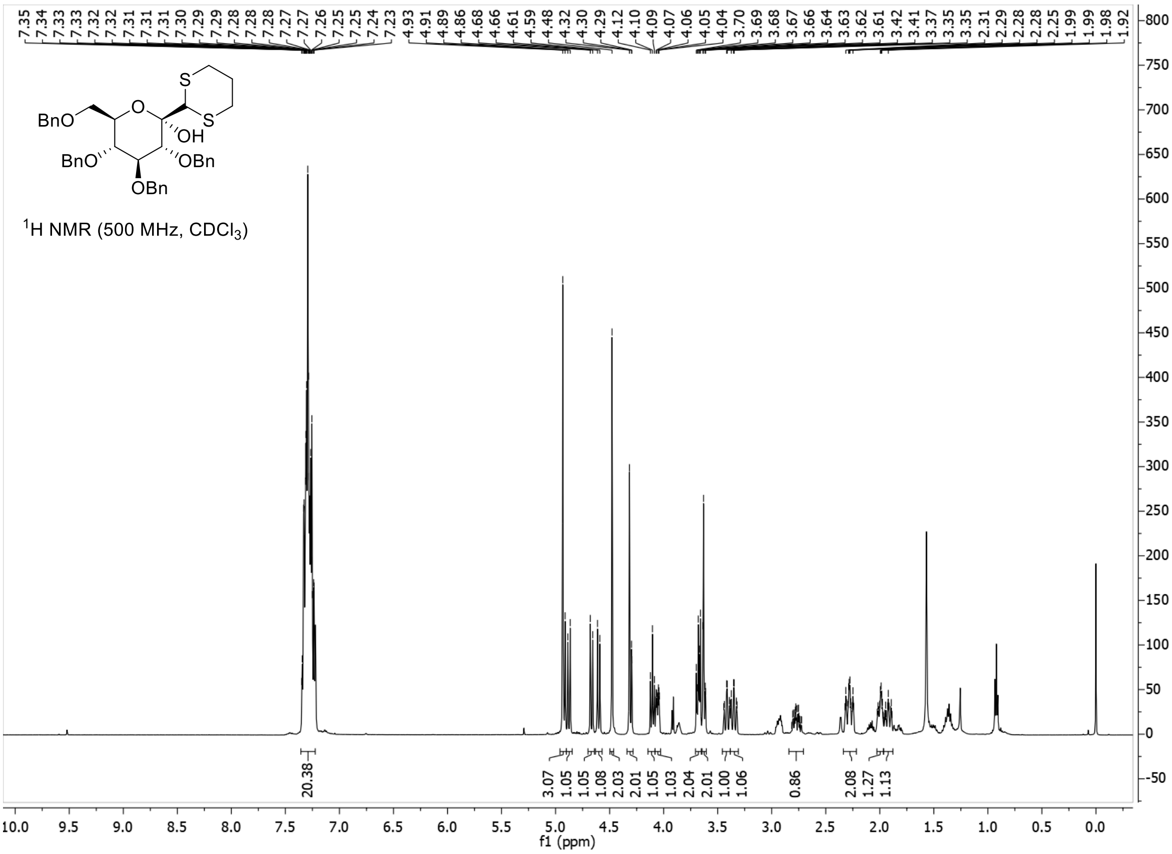

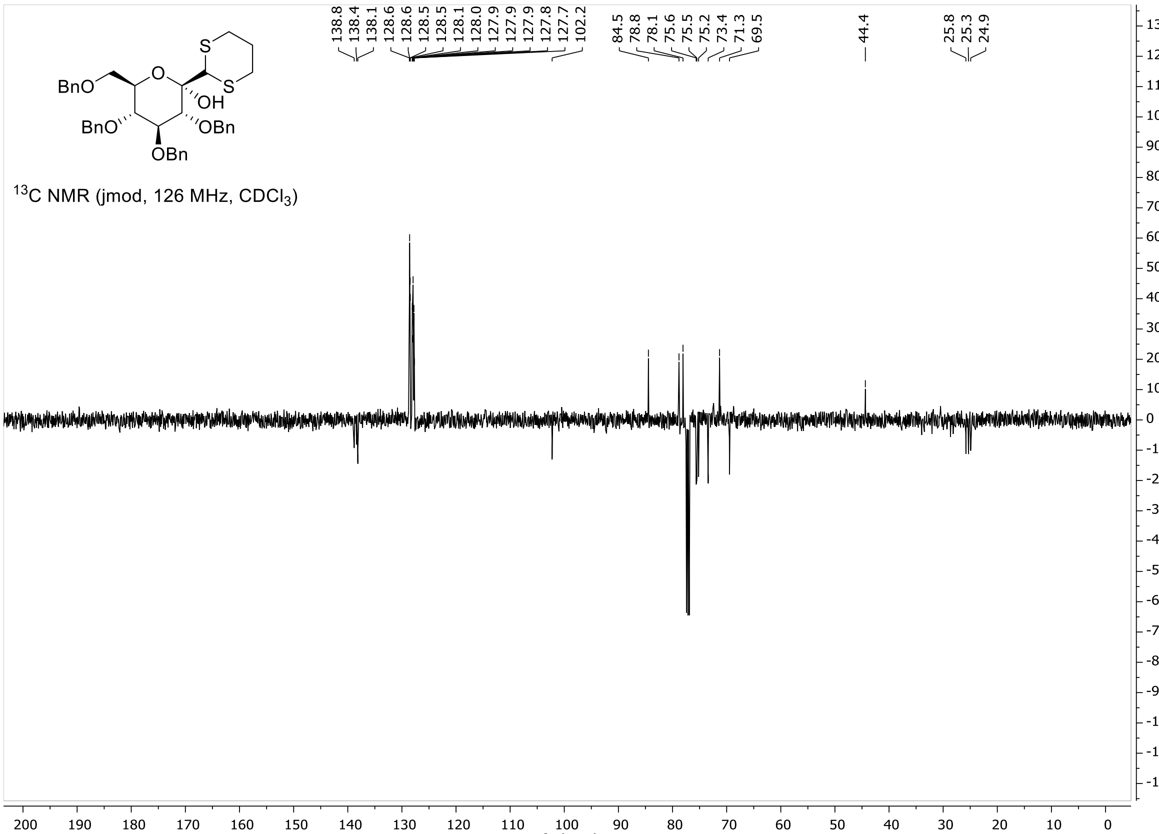

Compound 39

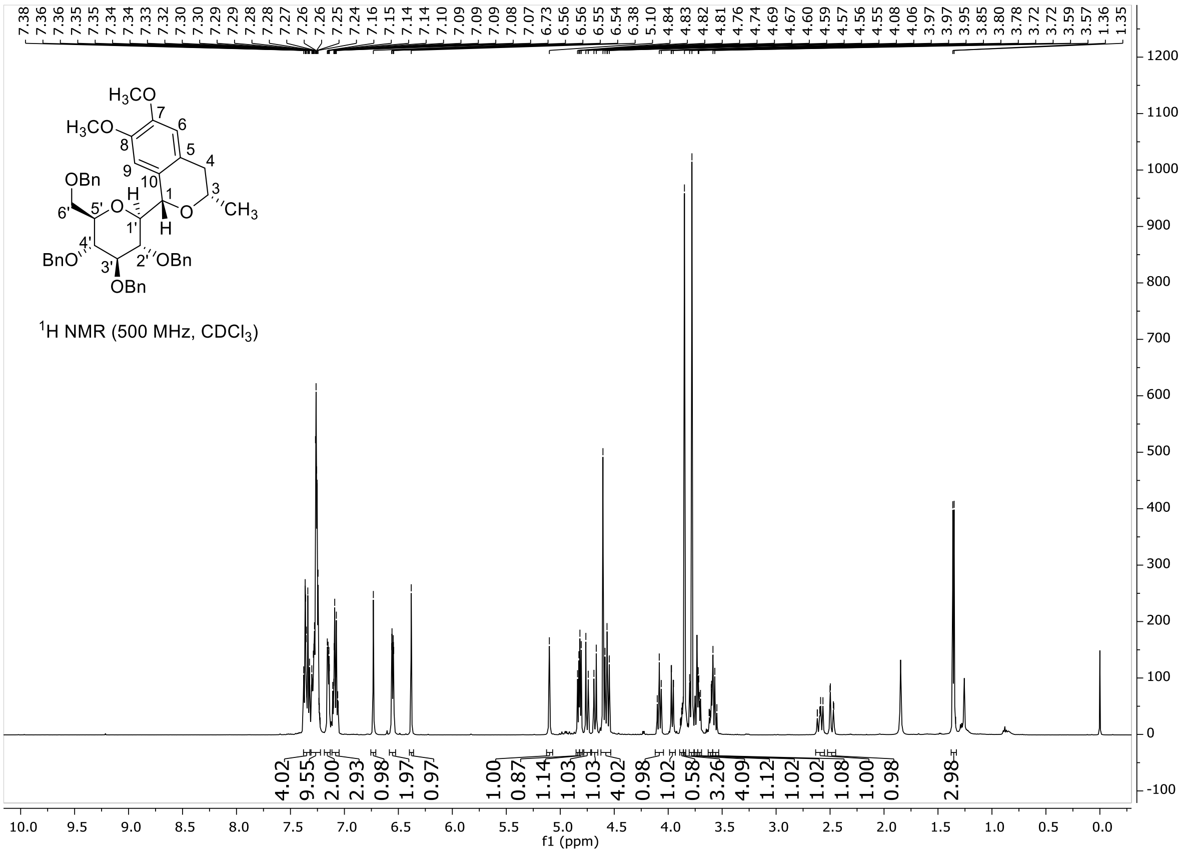

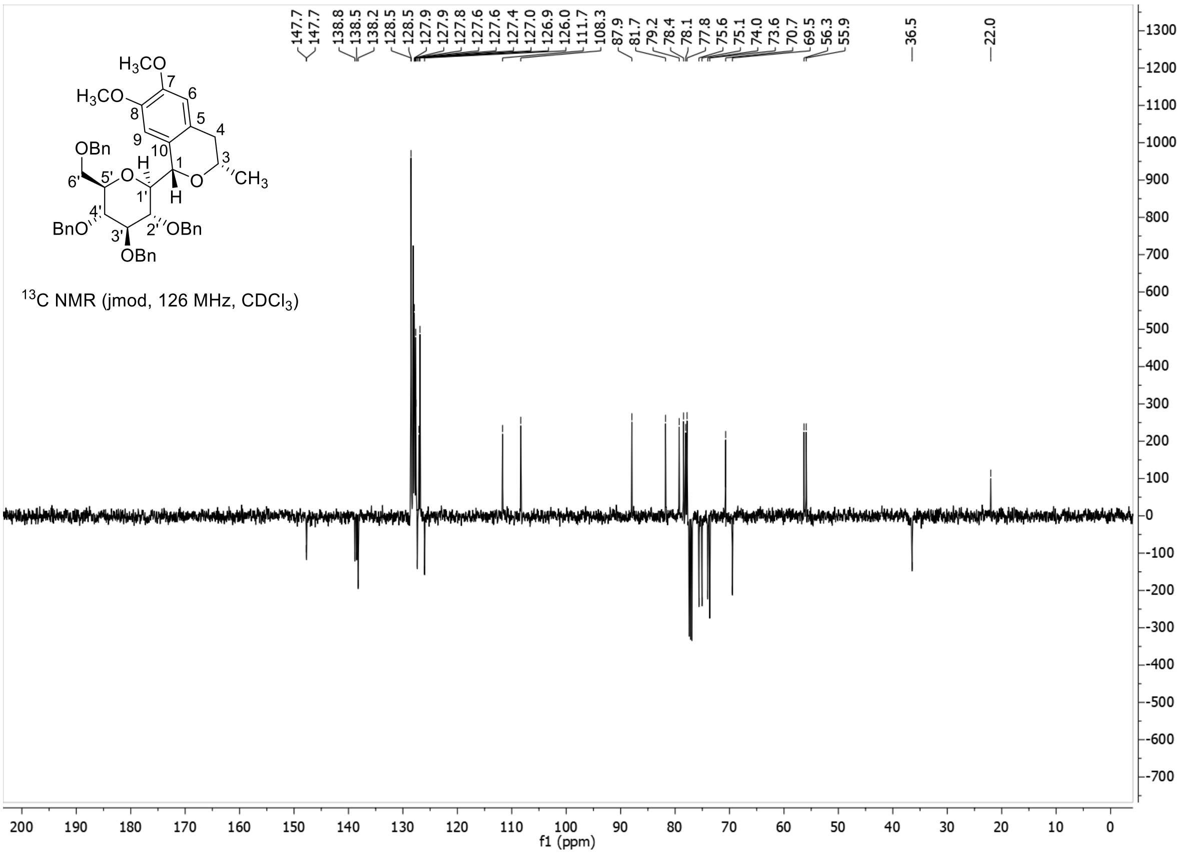

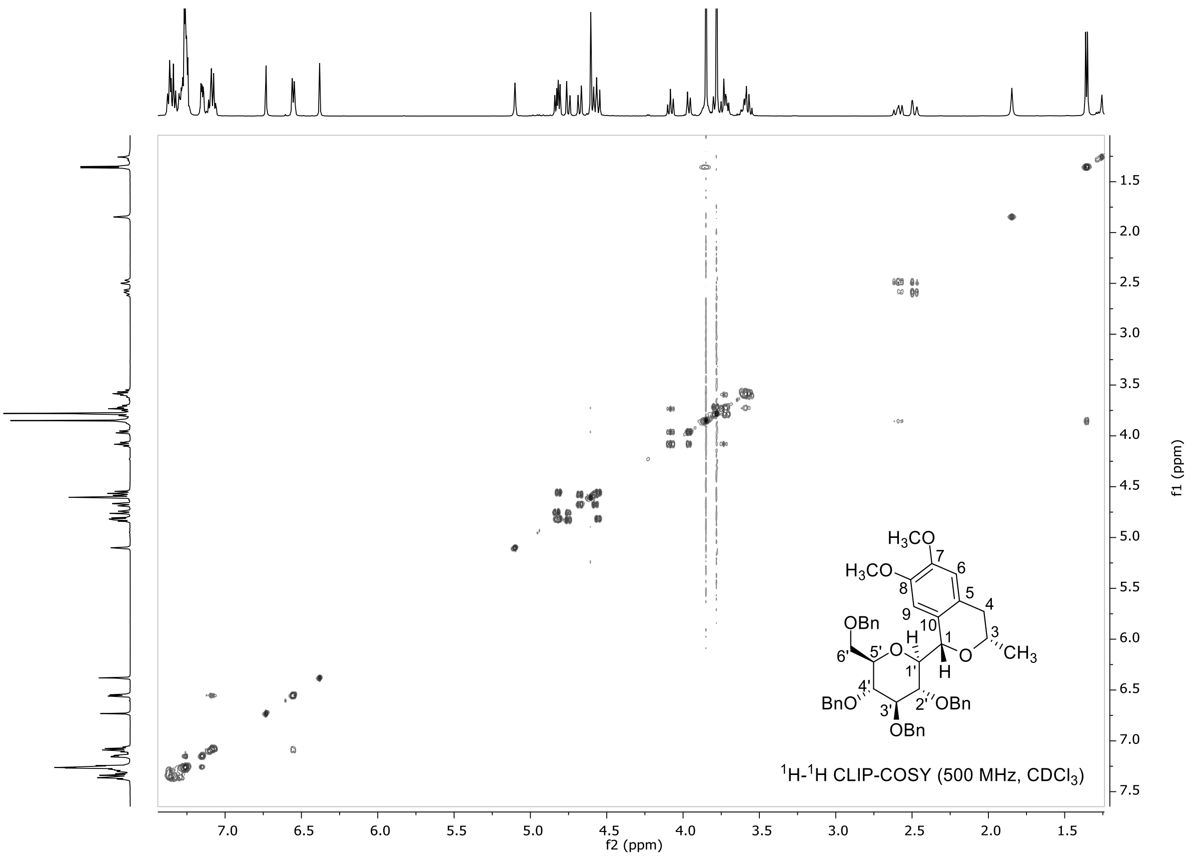
H3CO
H3CO
7
6
8
5
4
9
10
OBn
H
1
3
6'
5'
O
O
CH3
1'
H
4'
BnO
3'
2'
OBn
OBn
1H-1H CLIP-COSY (500 MHz, CDCl3)
f2 (ppm)
f1 (ppm)

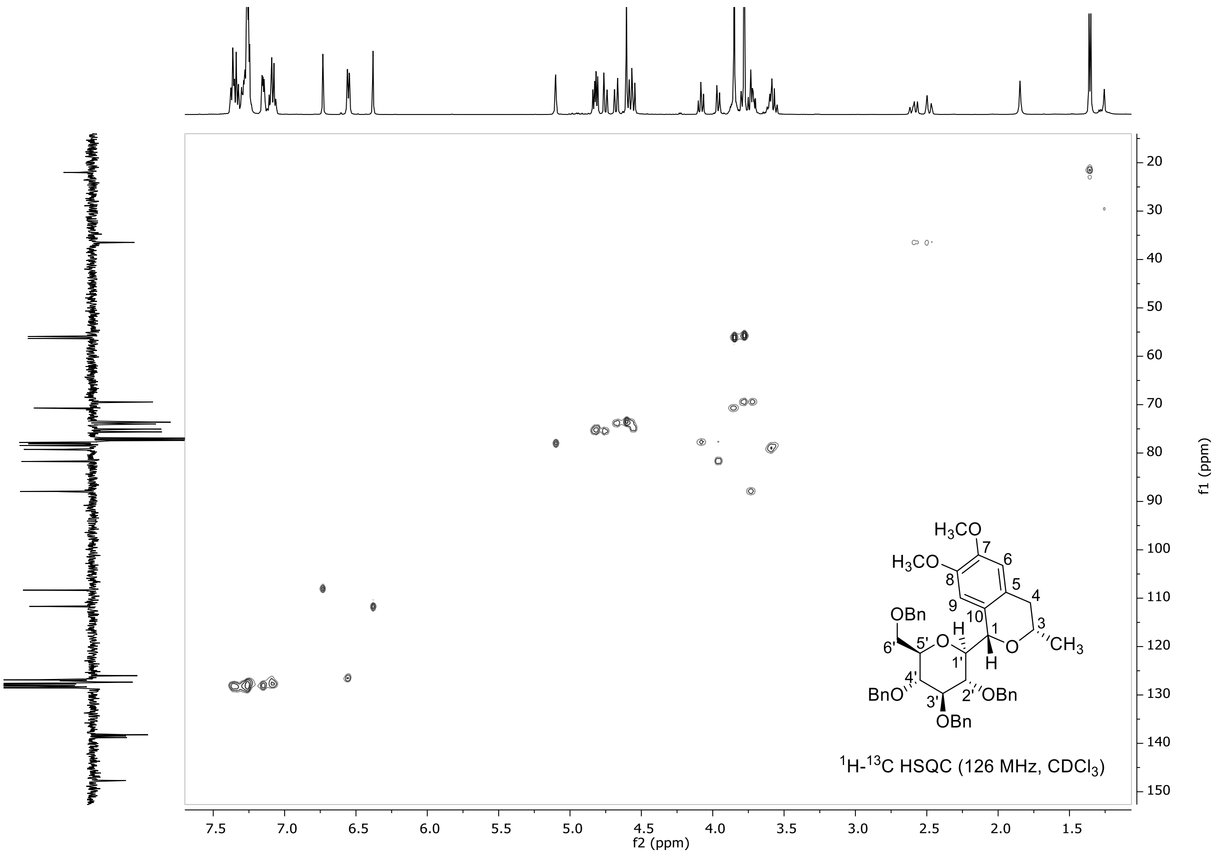
H3CO
H3CO
7
6
8
5
4
9
10
OBn
H
1
3
6'
5'
O
O
CH3
1'
H
4'
BnO
3'
2'
OBn
OBn
1H-13C HSQC (126 MHz, CDCl3)
f2 (ppm)
f1 (ppm)

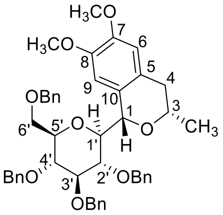

^1H-^{13}C HMBC (126 MHz, $CDCl_3$)

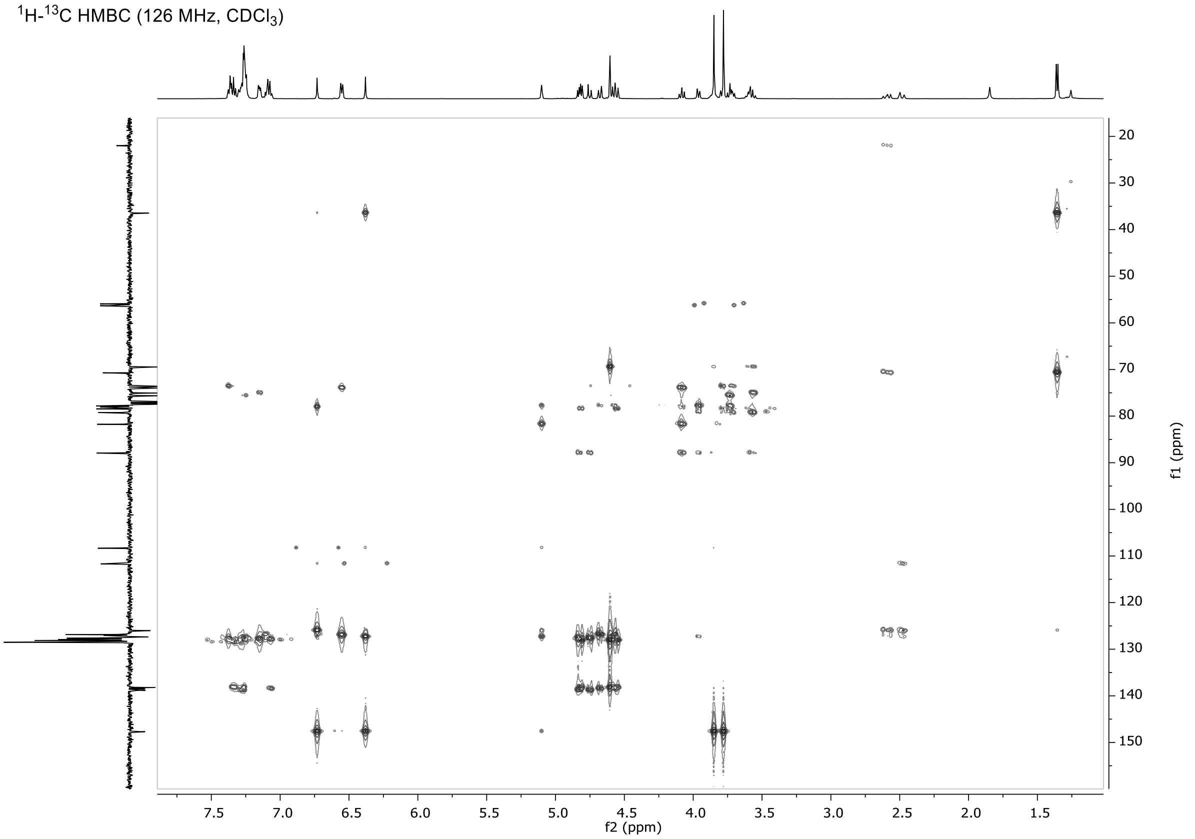

Compound 40

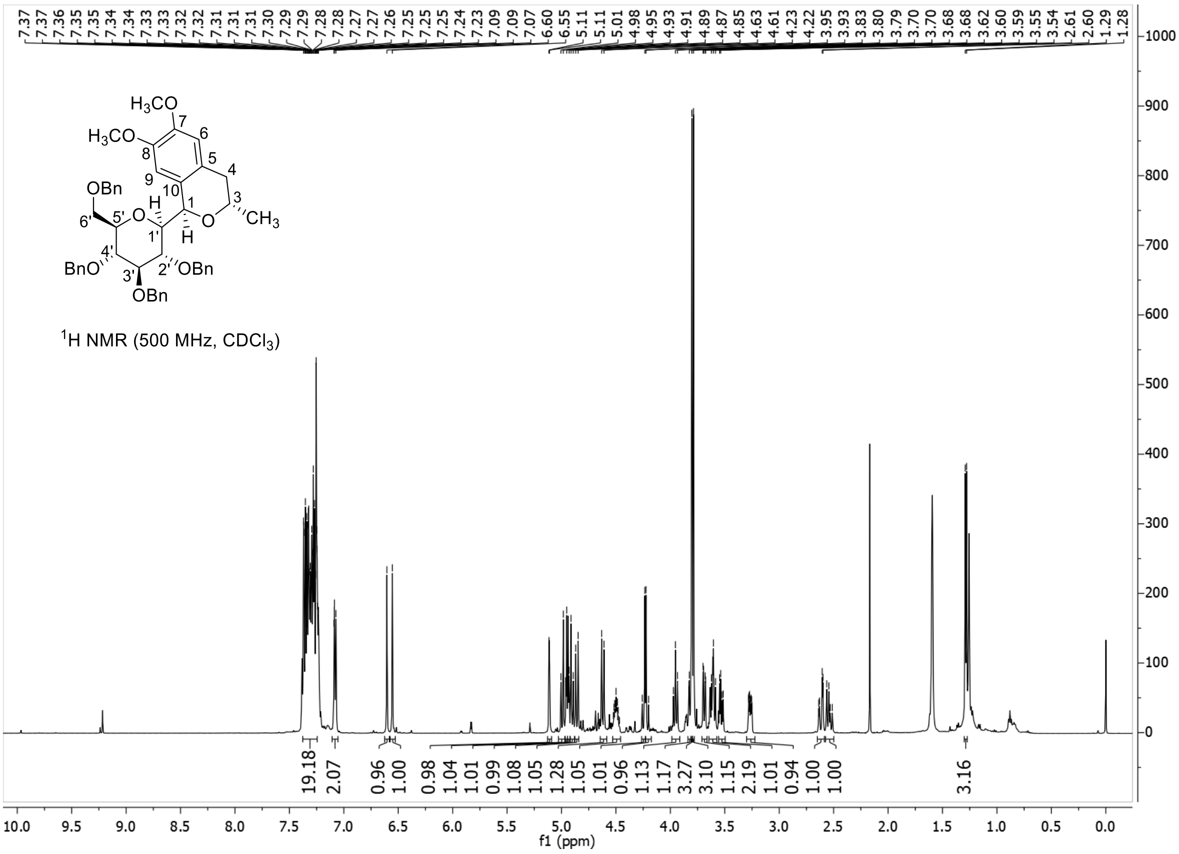
7.37 7.37 7.36 7.35 7.35 7.34 7.33 7.33 7.32 7.32 7.31 7.31 7.30 7.29 7.29 7.28 7.28 7.27 7.27 7.26 7.25 7.25 7.25 7.24 7.23 7.09 7.09 7.07 6.60 6.55 5.11 5.11 5.01 4.98 4.95 4.93 4.91 4.89 4.87 4.85 4.63 4.61 4.23 4.22 3.95 3.93 3.83 3.80 3.79 3.70 3.68 3.68 3.62 3.60 3.59 3.55 3.54 2.61 2.60 1.29 1.28
H_3CO
H_3CO
OBn
BnO
OBn
OBn
CH_3
^{1}H NMR (500 MHz, $CDCl_3$)
19.18 2.07 0.96 1.00 0.98 1.04 1.01 0.99 1.08 1.05 1.28 1.05 1.01 0.96 1.13 1.17 3.27 3.10 1.15 2.19 1.01 0.94 1.00 1.00 3.16
f1 (ppm)

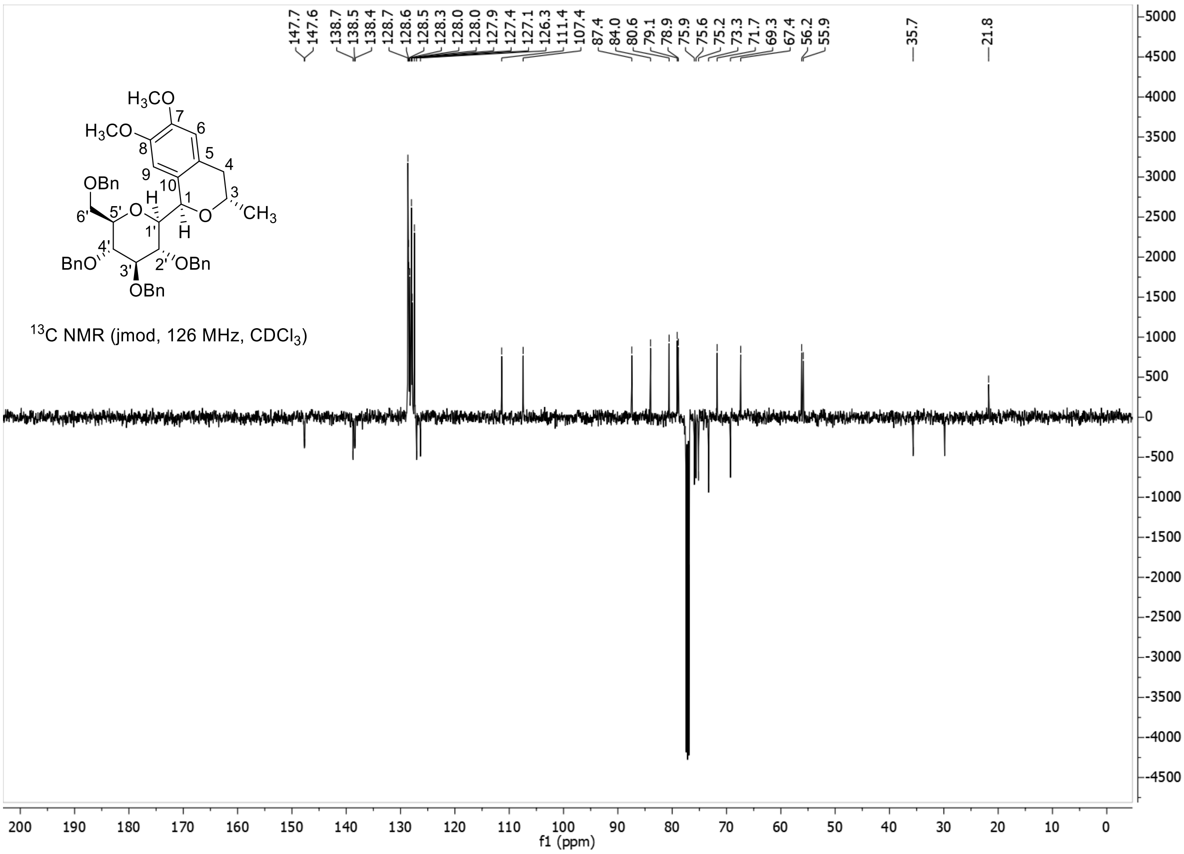
147.7 147.6 138.7 138.5 138.4 128.7 128.6 128.5 128.3 128.0 128.0 127.9 127.4 127.1 126.3 111.4 107.4 87.4 84.0 80.6 79.1 78.9 75.9 75.6 75.2 73.3 71.7 69.3 67.4 56.2 55.9 35.7 21.8
H_3CO
H_3CO
OBn
BnO
OBn
OBn
CH_3
^{13}C NMR (jmod, 126 MHz, $CDCl_3$)
f1 (ppm)

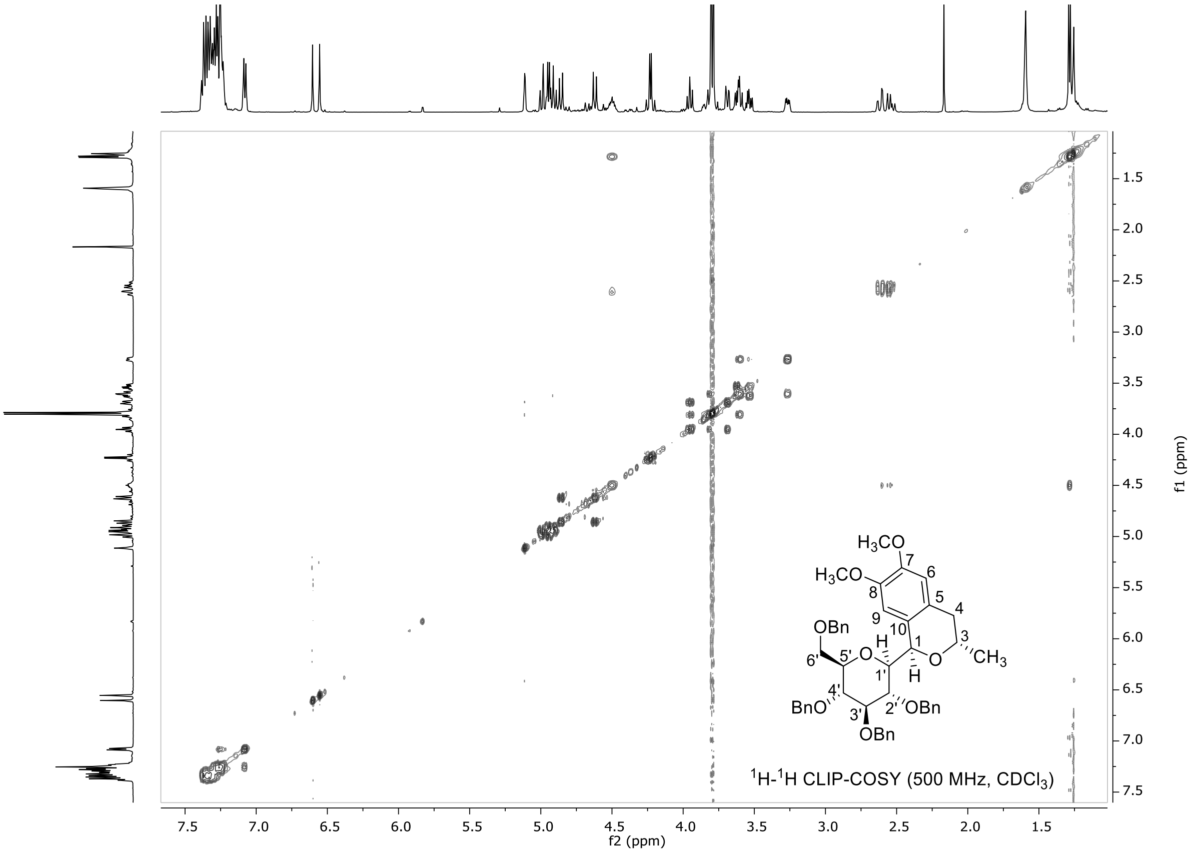
H3CO
H3CO
OBn
BnO
OBn
OBn
CH3
1H-1H CLIP-COSY (500 MHz, CDCl3)
f1 (ppm)
f2 (ppm)

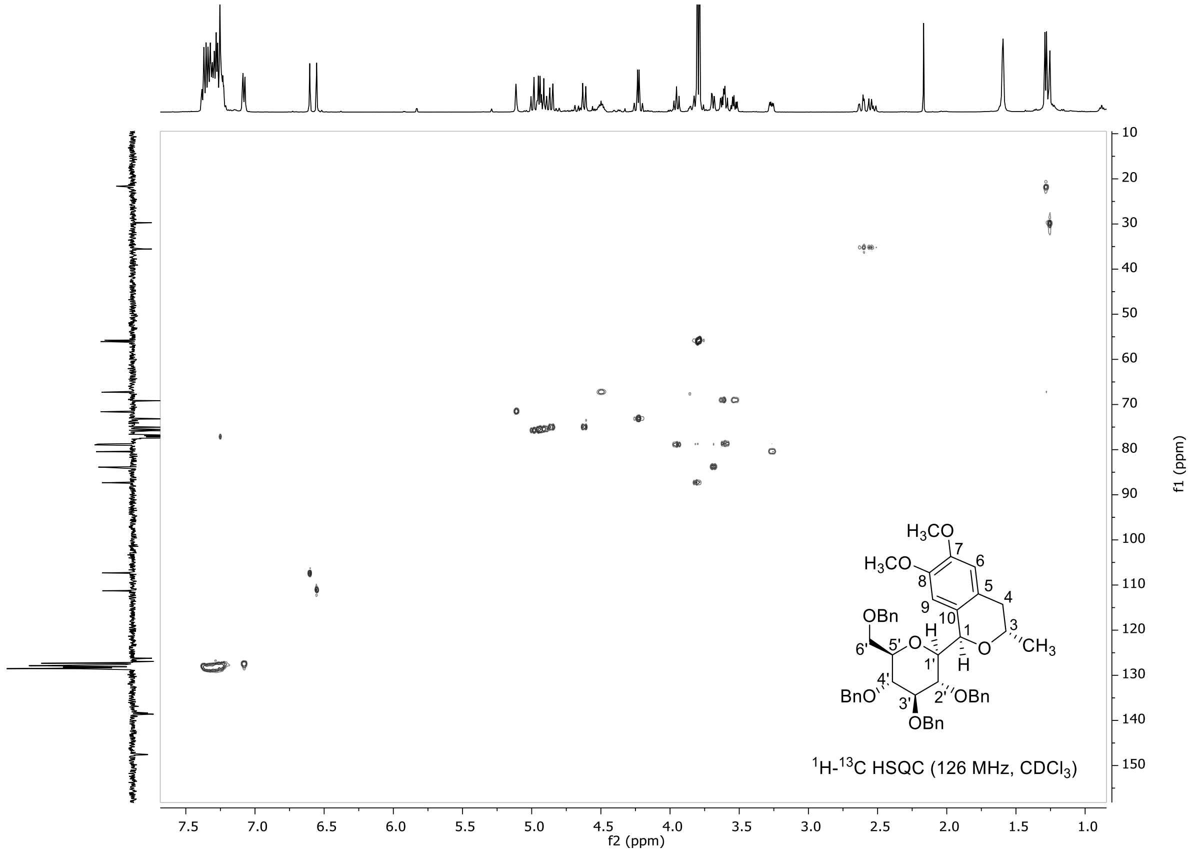
H3CO
H3CO
OBn
BnO
OBn
OBn
CH3
1H-13C HSQC (126 MHz, CDCl3)
f1 (ppm)
f2 (ppm)

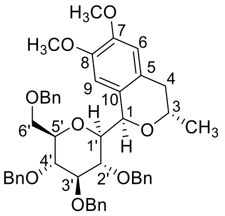

^1H-^1H ROESY (500 MHz, $CDCl_3$)

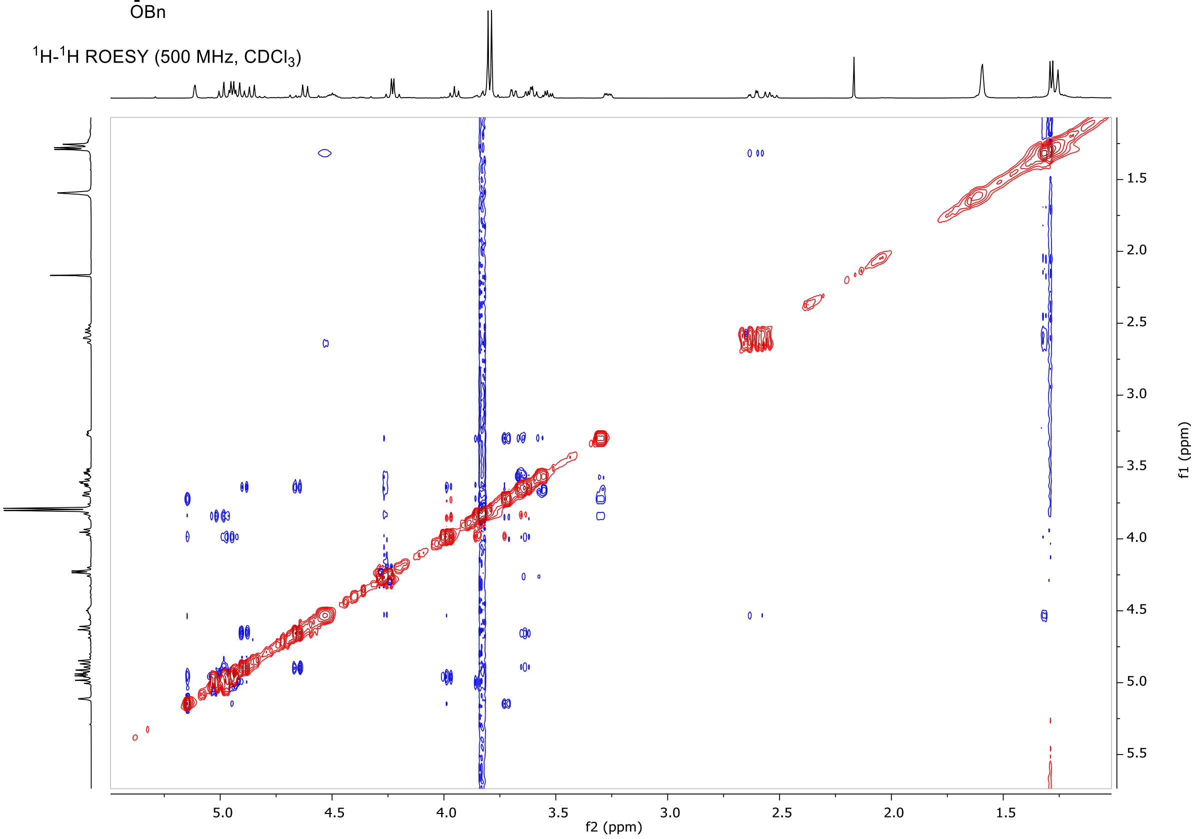

Compound 41

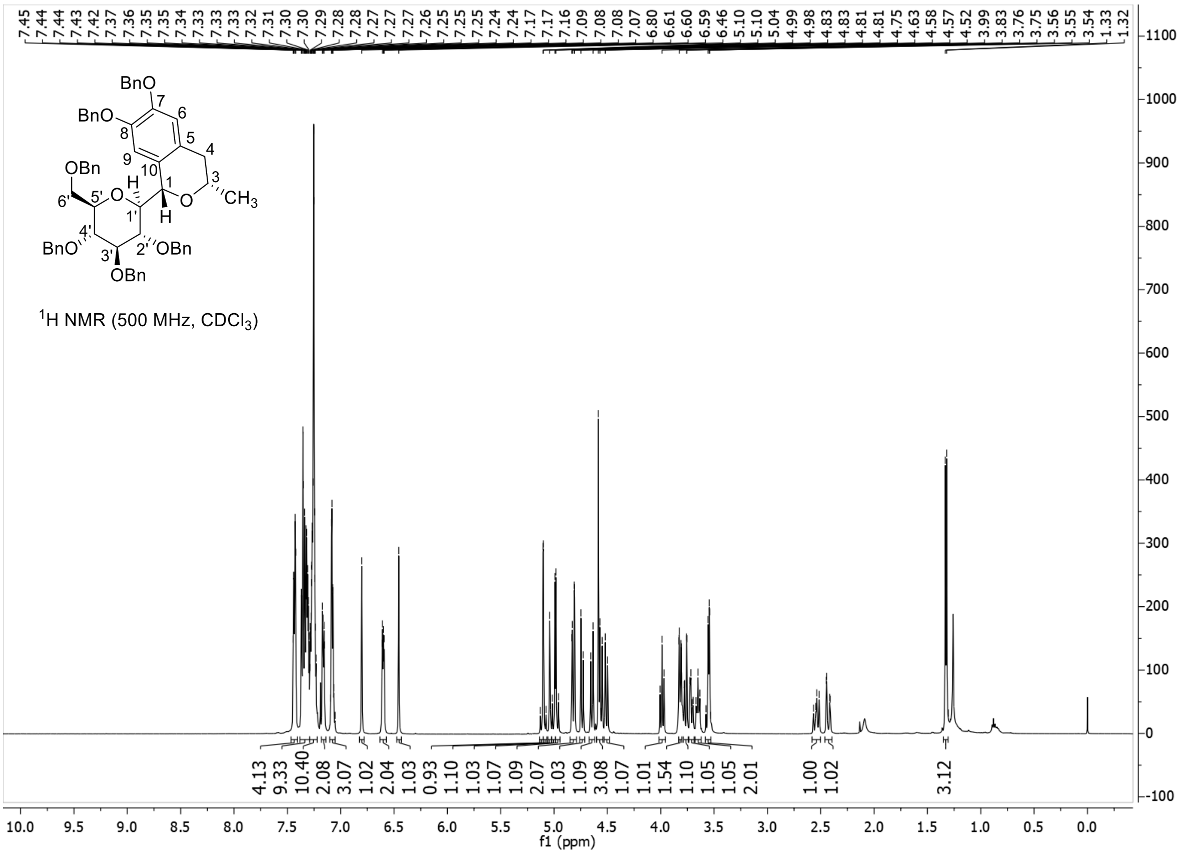

7.45 7.44 7.44 7.43 7.42 7.37 7.36 7.35 7.35 7.34 7.33 7.33 7.33 7.32 7.31 7.30 7.30 7.29 7.28 7.28 7.27 7.27 7.27 7.26 7.25 7.25 7.25 7.24 7.24 7.17 7.17 7.16 7.09 7.08 7.08 7.07 6.80 6.61 6.60 6.59 6.46 5.10 5.10 5.04 4.99 4.98 4.83 4.83 4.81 4.81 4.75 4.63 4.58 4.57 4.52 3.99 3.83 3.76 3.75 3.56 3.55 3.54 1.33 1.32
BnO BnO OBn BnO OBn OBn H H O O CH3
1H NMR (500 MHz, CDCl3)
4.13 9.33 10.40 2.08 3.07 1.02 2.04 1.03 0.93 1.10 1.03 1.07 1.09 2.07 1.03 1.09 3.08 1.07 1.01 1.54 1.10 1.05 1.05 2.01 1.00 1.02 3.12
f1 (ppm)

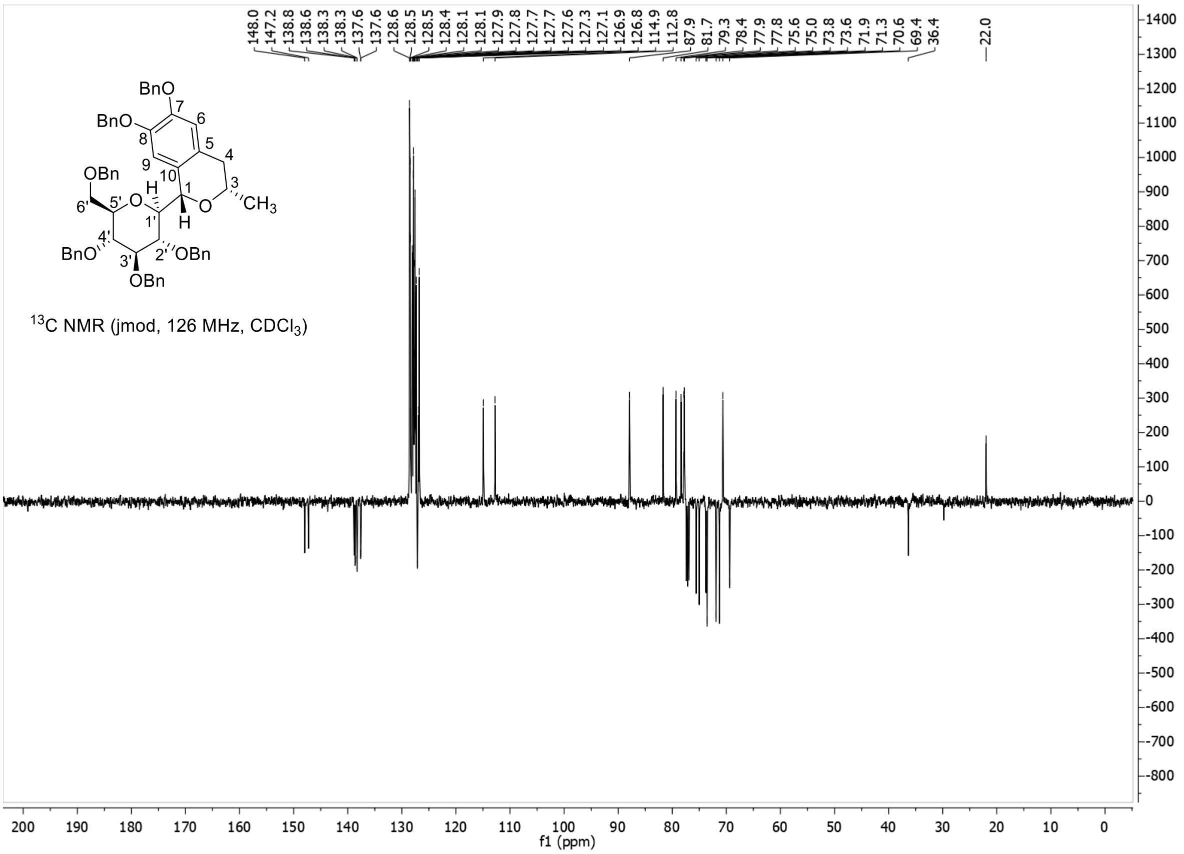

148.0 147.2 138.8 138.6 138.3 138.3 137.6 128.6 128.5 128.5 128.4 128.1 128.1 127.9 127.8 127.7 127.7 127.6 127.3 127.1 126.9 126.8 114.9 112.8 87.9 81.7 79.3 78.4 77.9 77.8 75.6 75.0 73.8 73.6 71.9 71.3 70.6 69.4 36.4 22.0
BnO BnO OBn BnO OBn OBn H H O O CH3
13C NMR (jmod, 126 MHz, CDCl3)
f1 (ppm)

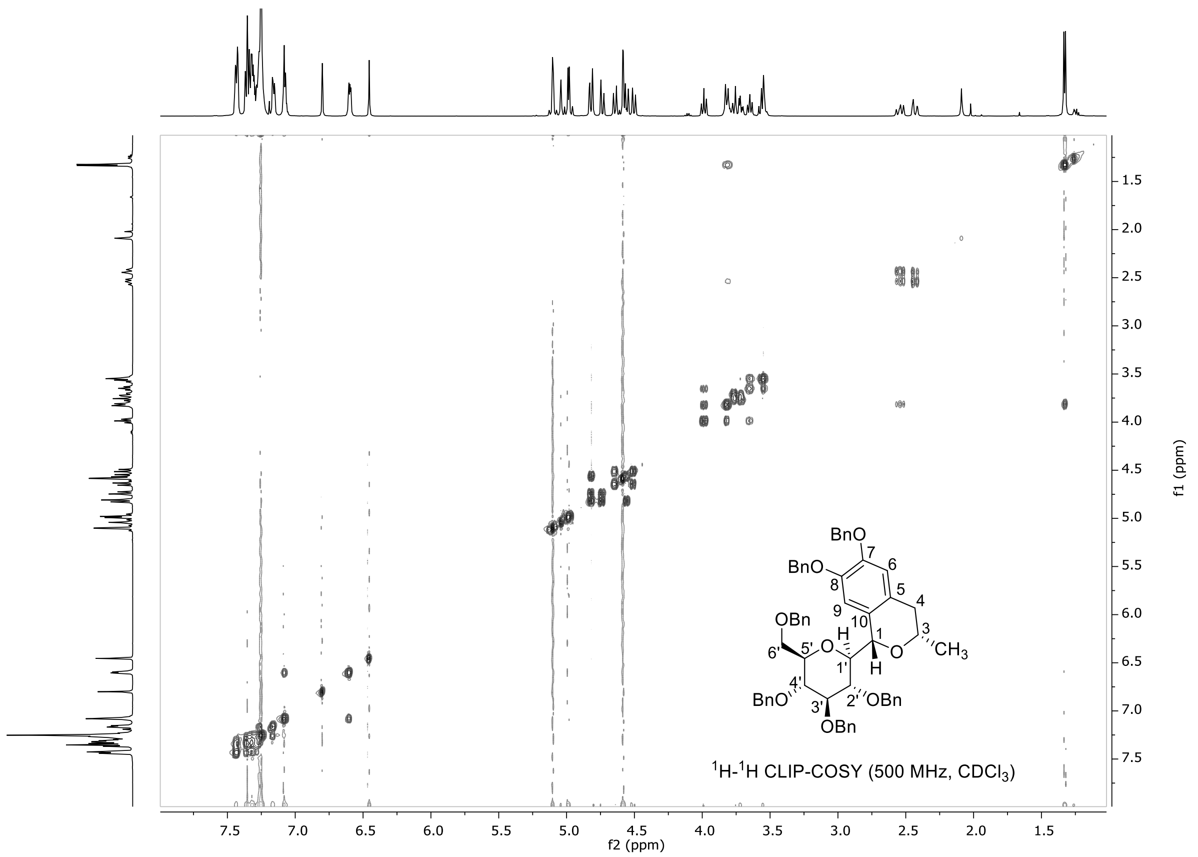

f1 (ppm)
f2 (ppm)
BnO
BnO
OBn
BnO
OBn
OBn
CH3
^{1}H-^{1}H CLIP-COSY (500 MHz, CDCl$_3$)

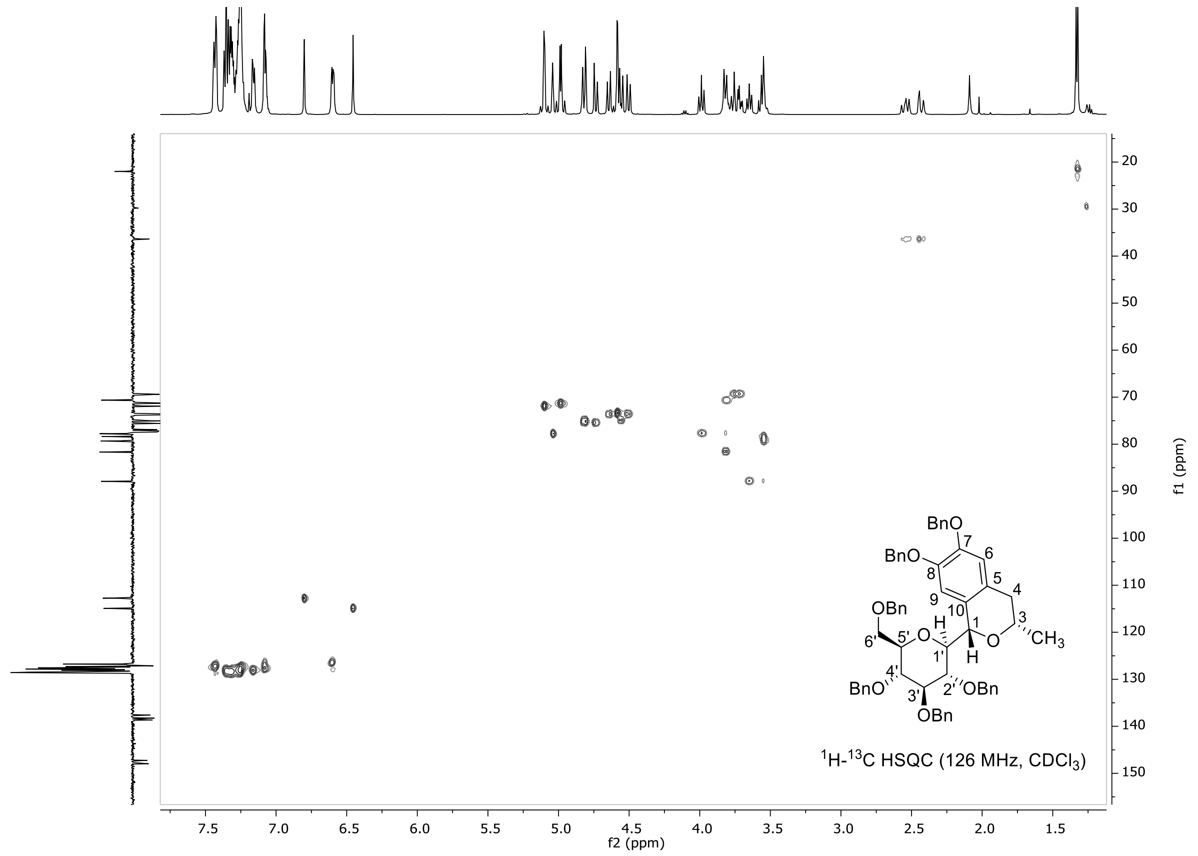

f1 (ppm)
f2 (ppm)
BnO
BnO
OBn
BnO
OBn
OBn
CH3
^{1}H-^{13}C HSQC (126 MHz, CDCl$_3$)

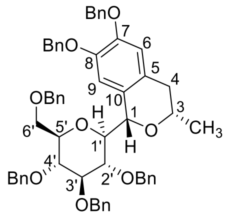

^{1}H-^{13}C HMBC (126 MHz, $CDCl_3$)

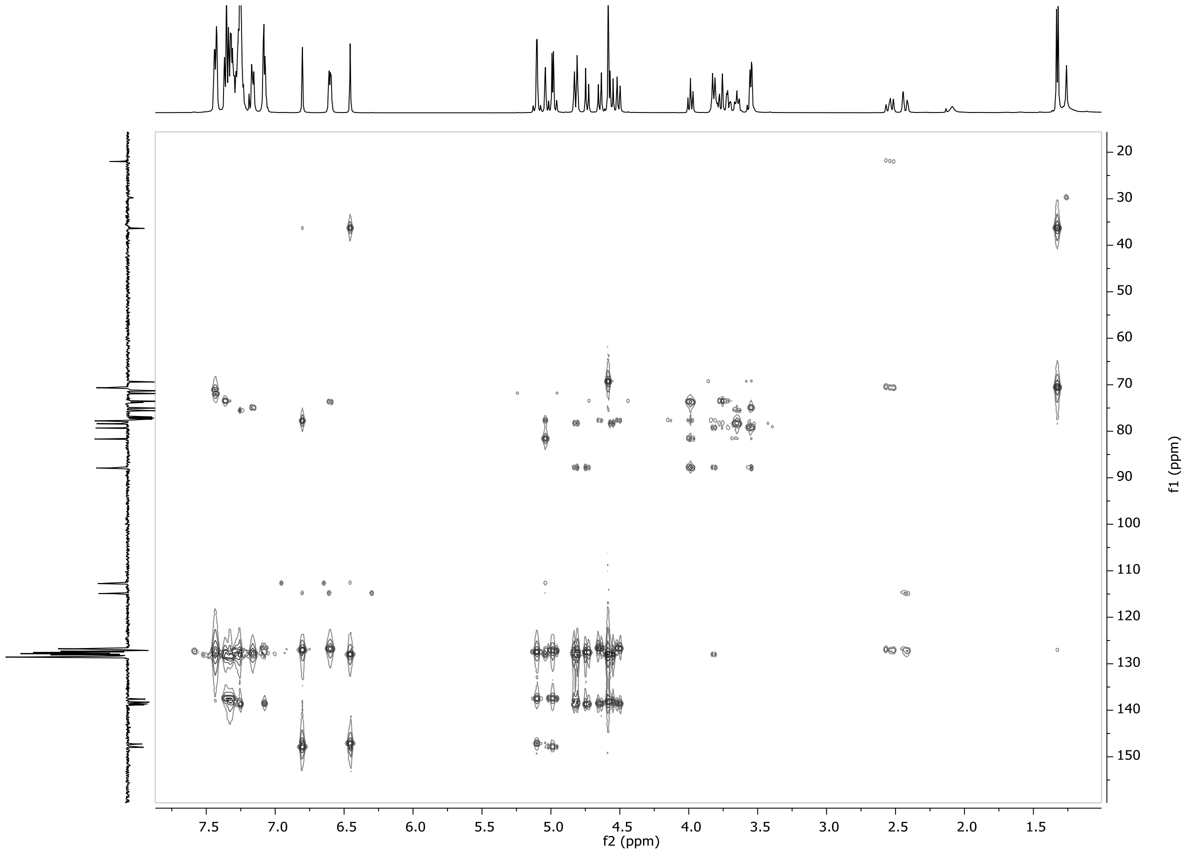

Compound 42

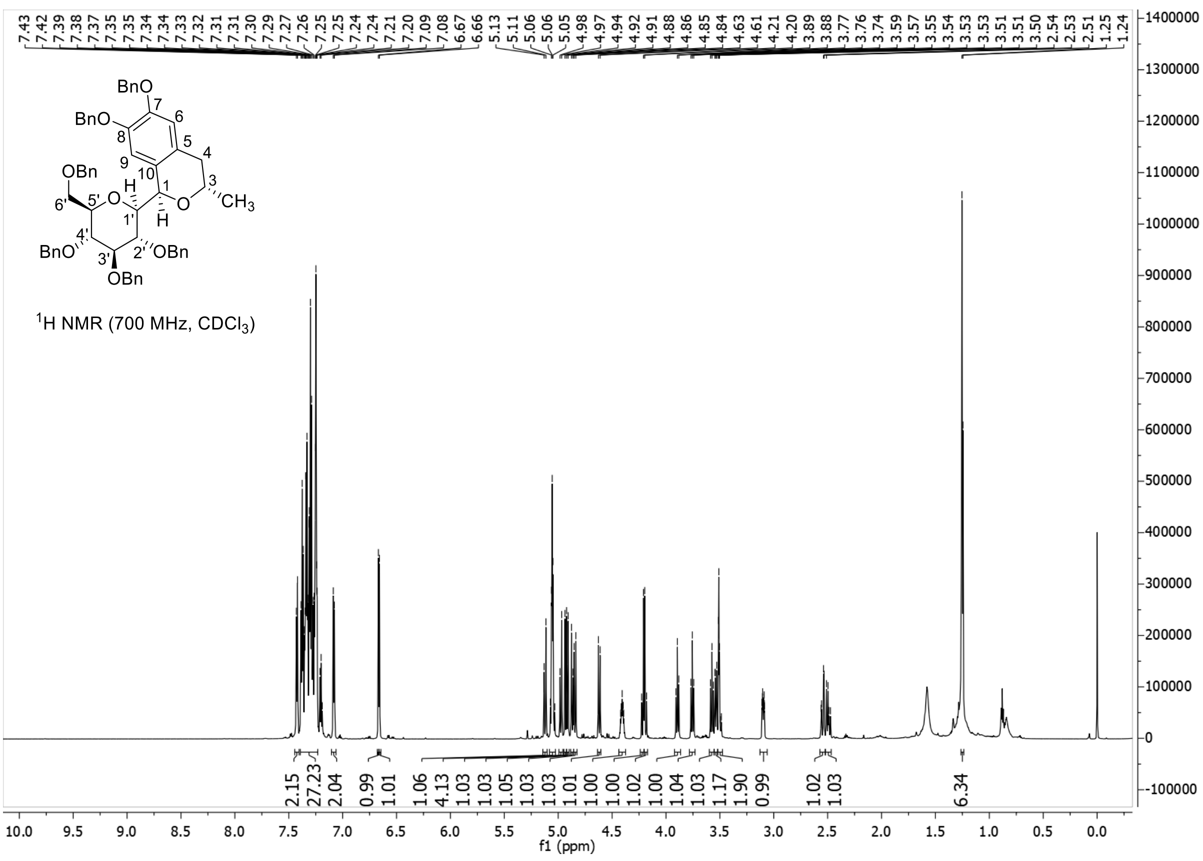

7.43
7.42
7.39
7.38
7.37
7.35
7.34
7.34
7.33
7.32
7.31
7.30
7.29
7.27
7.26
7.25
7.25
7.24
7.24
7.21
7.20
7.09
7.08
6.67
6.66
5.13
5.11
5.06
5.06
5.05
4.98
4.97
4.94
4.92
4.91
4.88
4.86
4.85
4.84
4.63
4.61
4.21
4.20
3.89
3.88
3.77
3.76
3.74
3.59
3.57
3.55
3.54
3.53
3.53
3.51
3.51
3.50
2.54
2.53
2.51
1.25
1.24
BnO
BnO
OBn
BnO
OBn
OBn
CH_3
^{1}H NMR (700 MHz, $CDCl_3$)
2.15
27.23
2.04
0.99
1.01
1.06
4.13
1.03
1.03
1.05
1.03
1.03
1.01
1.00
1.00
1.02
1.00
1.04
1.03
1.17
1.90
0.99
1.02
1.03
6.34
f1 (ppm)

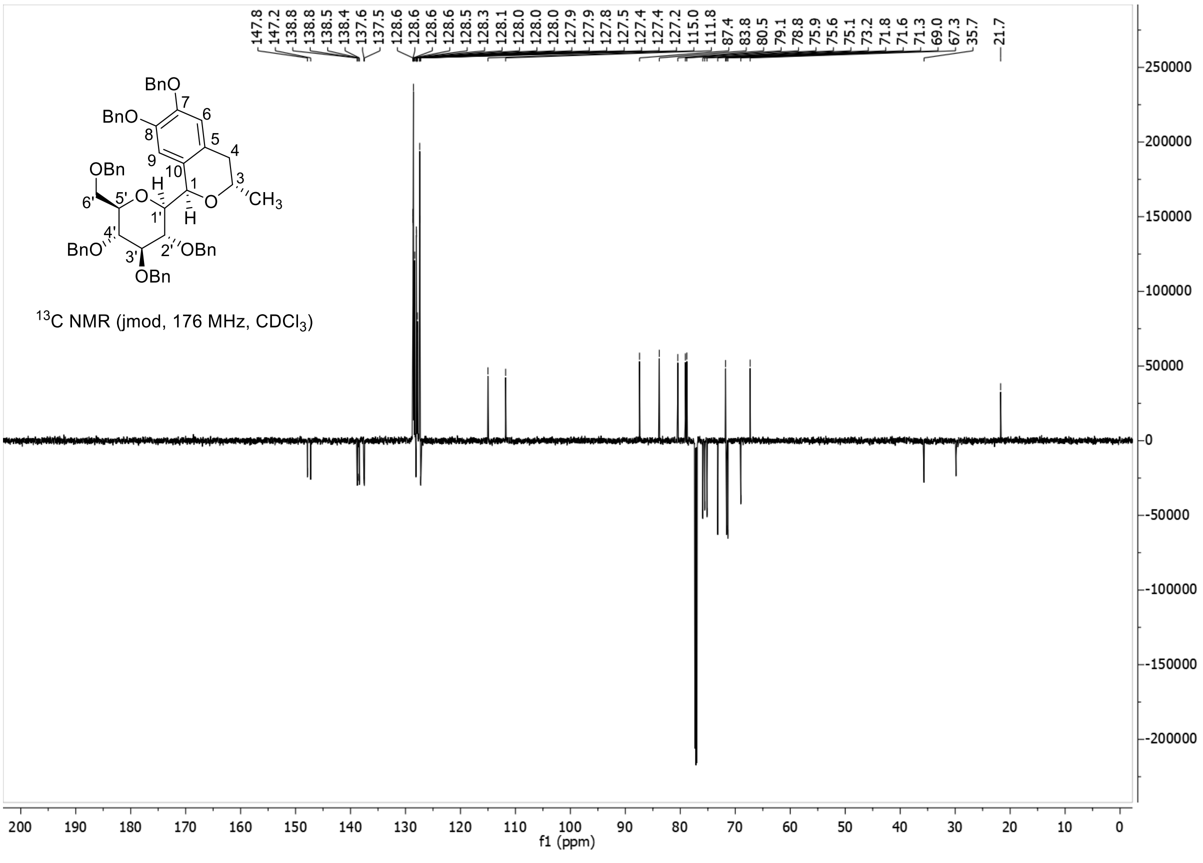

147.8
147.2
138.8
138.8
138.5
138.4
137.6
137.5
128.6
128.6
128.6
128.6
128.5
128.3
128.1
128.0
128.0
128.0
127.9
127.9
127.8
127.5
127.4
127.4
127.2
115.0
111.8
87.4
83.8
80.5
79.1
78.8
75.9
75.6
75.1
73.2
71.8
71.6
71.3
69.0
67.3
35.7
21.7
BnO
BnO
OBn
BnO
OBn
OBn
CH_3
^{13}C NMR (jmod, 176 MHz, $CDCl_3$)
f1 (ppm)

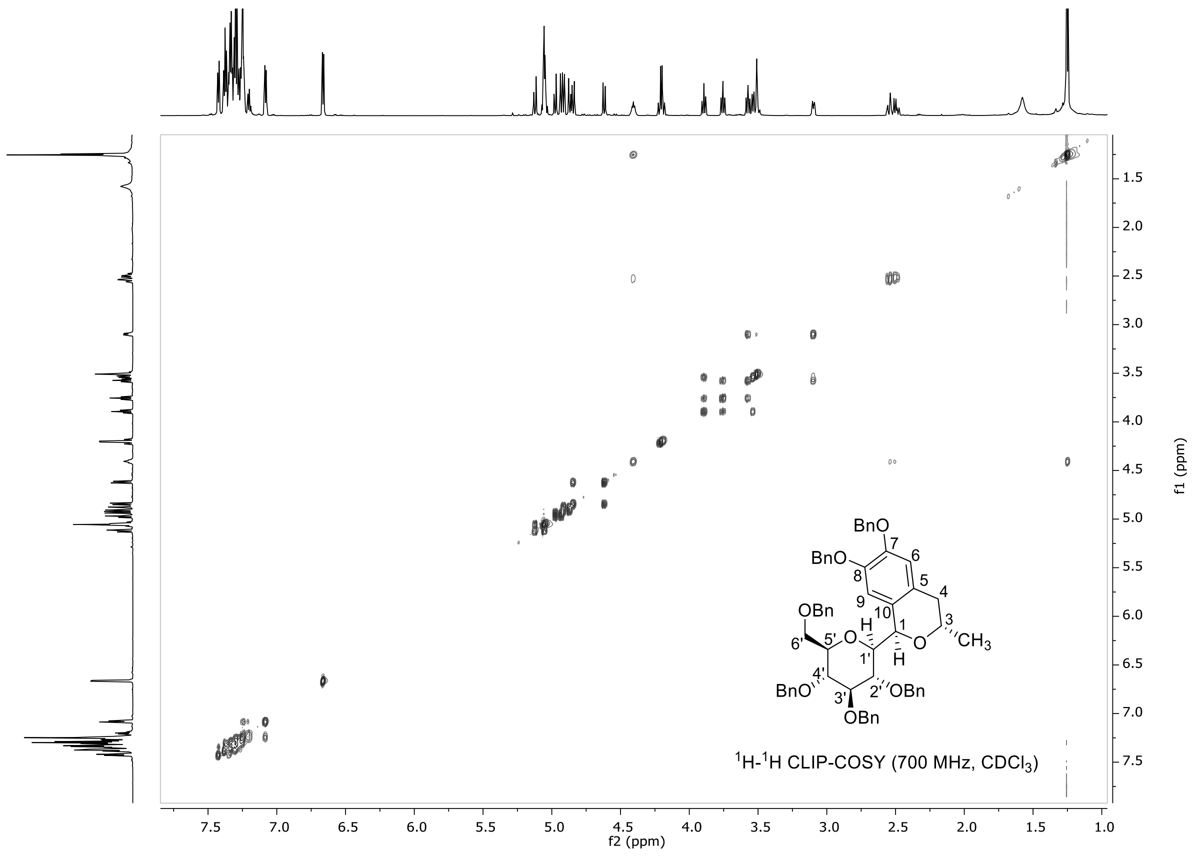
BnO
7
6
BnO
8
5
4
9
10
OBn
H
1
3
6'
5'
O
O
CH3
1'
H
4'
BnO
3'
2'
OBn
OBn
1H-1H CLIP-COSY (700 MHz, CDCl3)
f1 (ppm)
f2 (ppm)

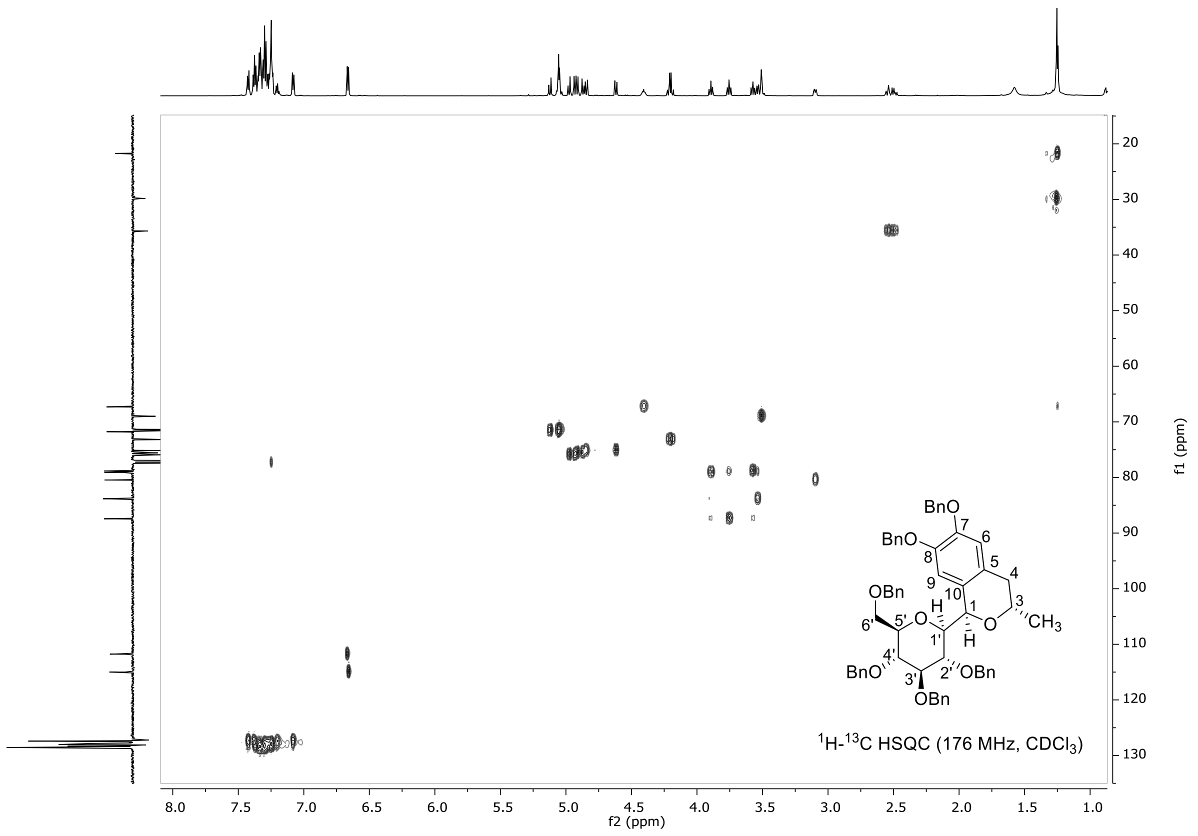
BnO
7
6
BnO
8
5
4
9
10
OBn
H
1
3
6'
5'
O
O
CH3
1'
H
4'
BnO
3'
2'
OBn
OBn
1H-13C HSQC (176 MHz, CDCl3)
f1 (ppm)
f2 (ppm)

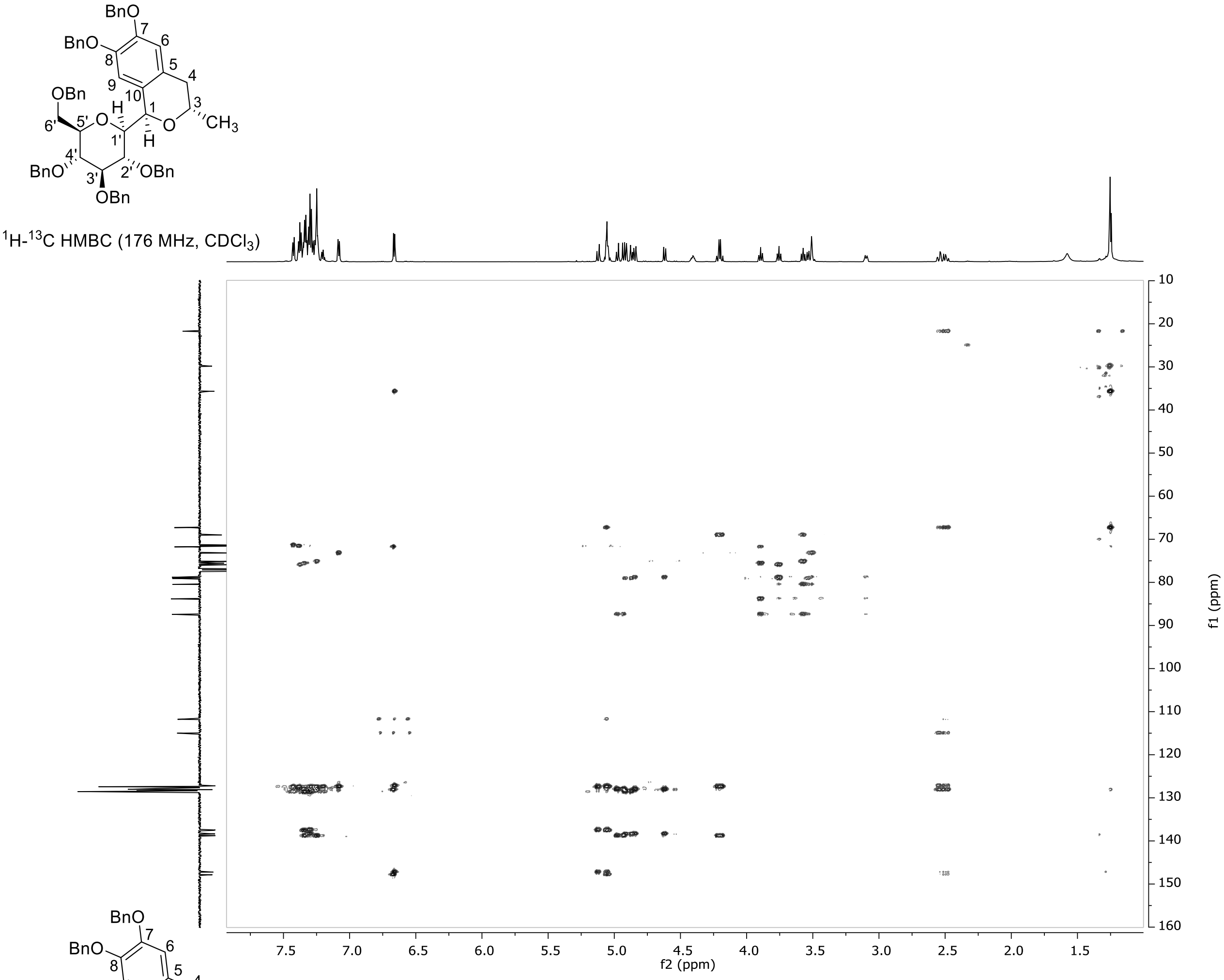

^{1}H-^{13}C HMBC (176 MHz, $CDCl_3$)

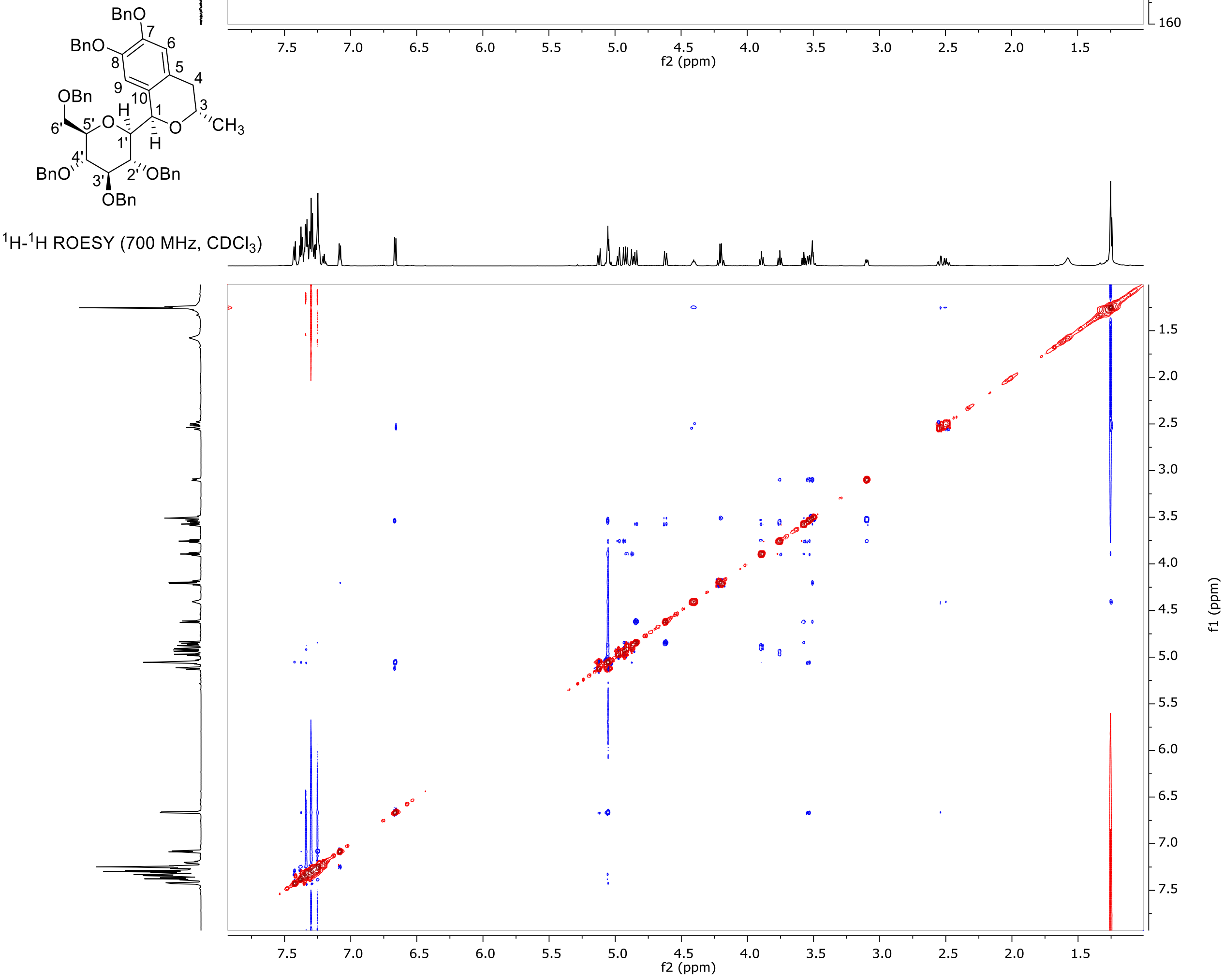

^{1}H-^{1}H ROESY (700 MHz, $CDCl_3$)

Compound 43

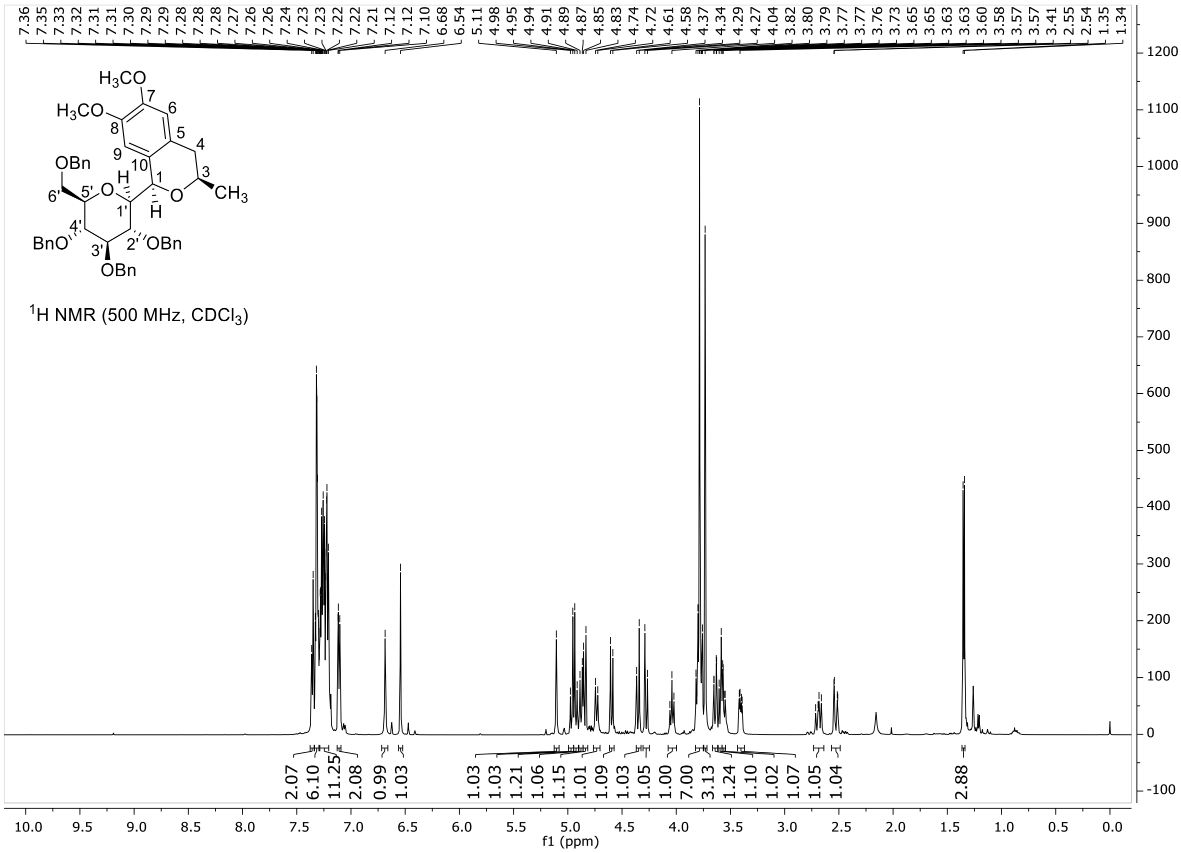

H3CO
H3CO
OBn
BnO
OBn
OBn
CH3
1H NMR (500 MHz, CDCl3)
f1 (ppm)

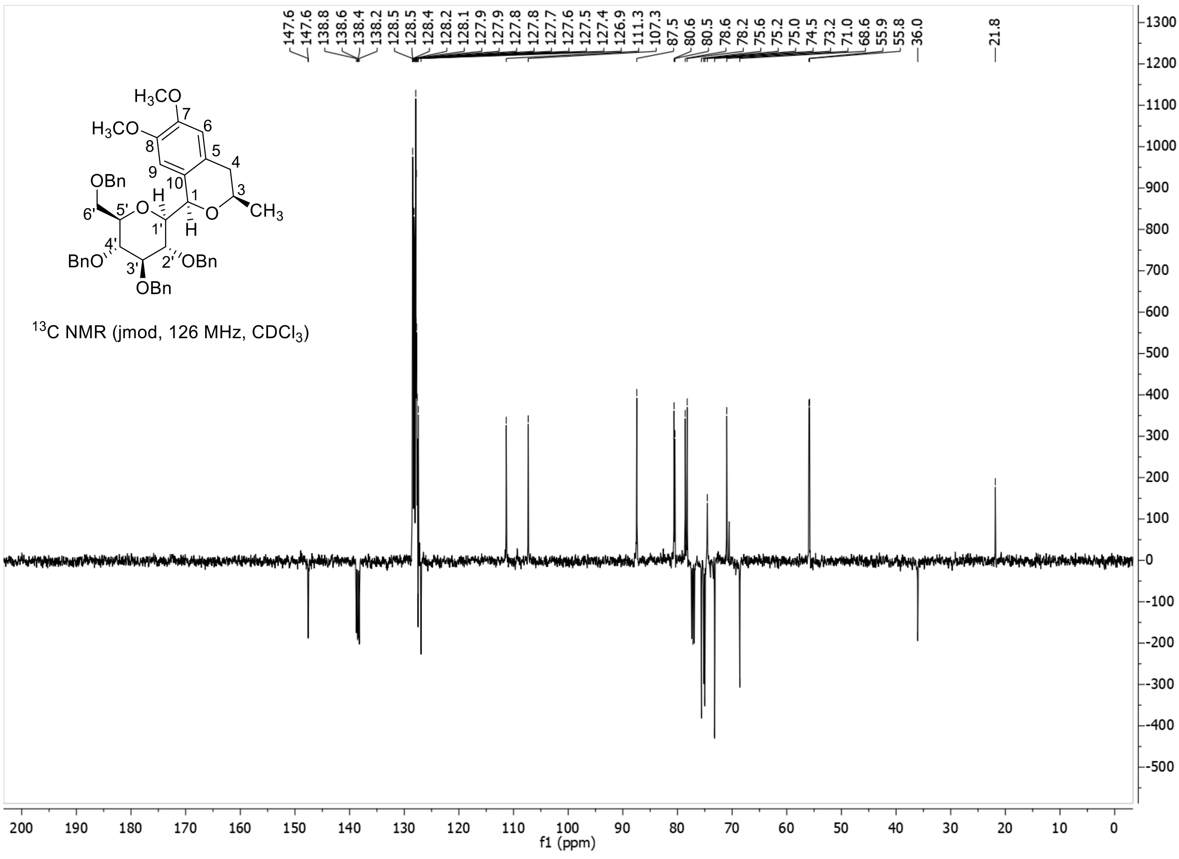

H3CO
H3CO
OBn
BnO
OBn
OBn
CH3
13C NMR (jmod, 126 MHz, CDCl3)
f1 (ppm)

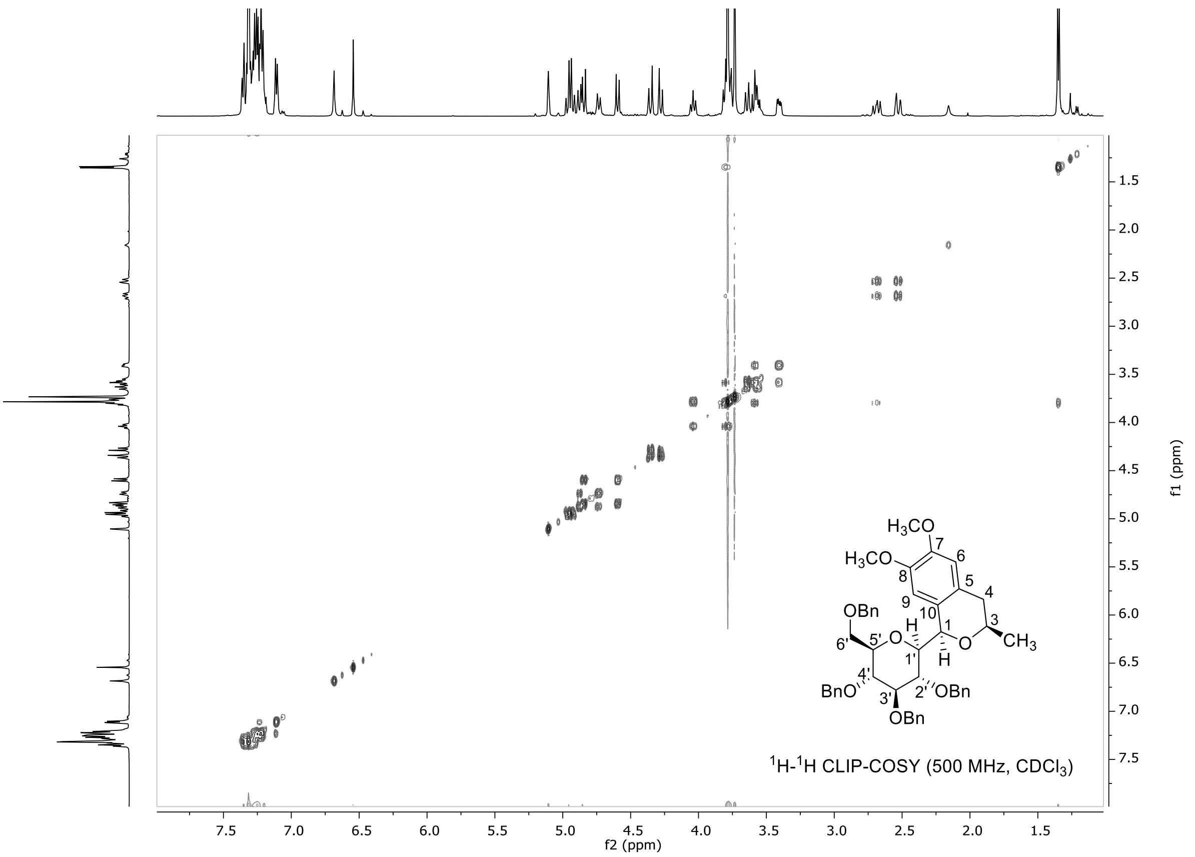

H3CO
7
6
H3CO
8
5
4
9
10
OBn
H
1
3
6'
5'
O
O
CH3
1'
H
4'
2'
BnO
OBn
3'
OBn
1H-1H CLIP-COSY (500 MHz, CDCl3)
f2 (ppm)
f1 (ppm)

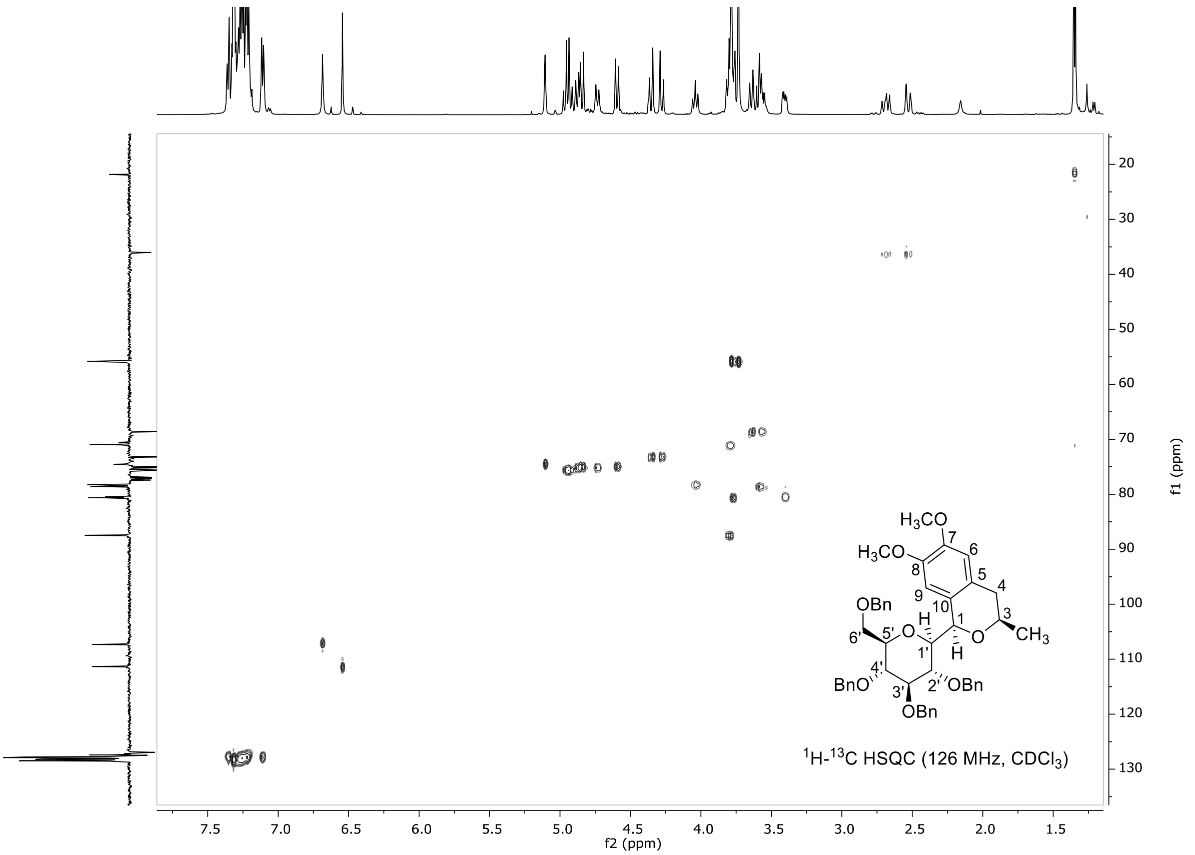

H3CO
7
6
H3CO
8
5
4
9
10
OBn
H
1
3
6'
5'
O
O
CH3
1'
H
4'
2'
BnO
OBn
3'
OBn
1H-13C HSQC (126 MHz, CDCl3)
f2 (ppm)
f1 (ppm)

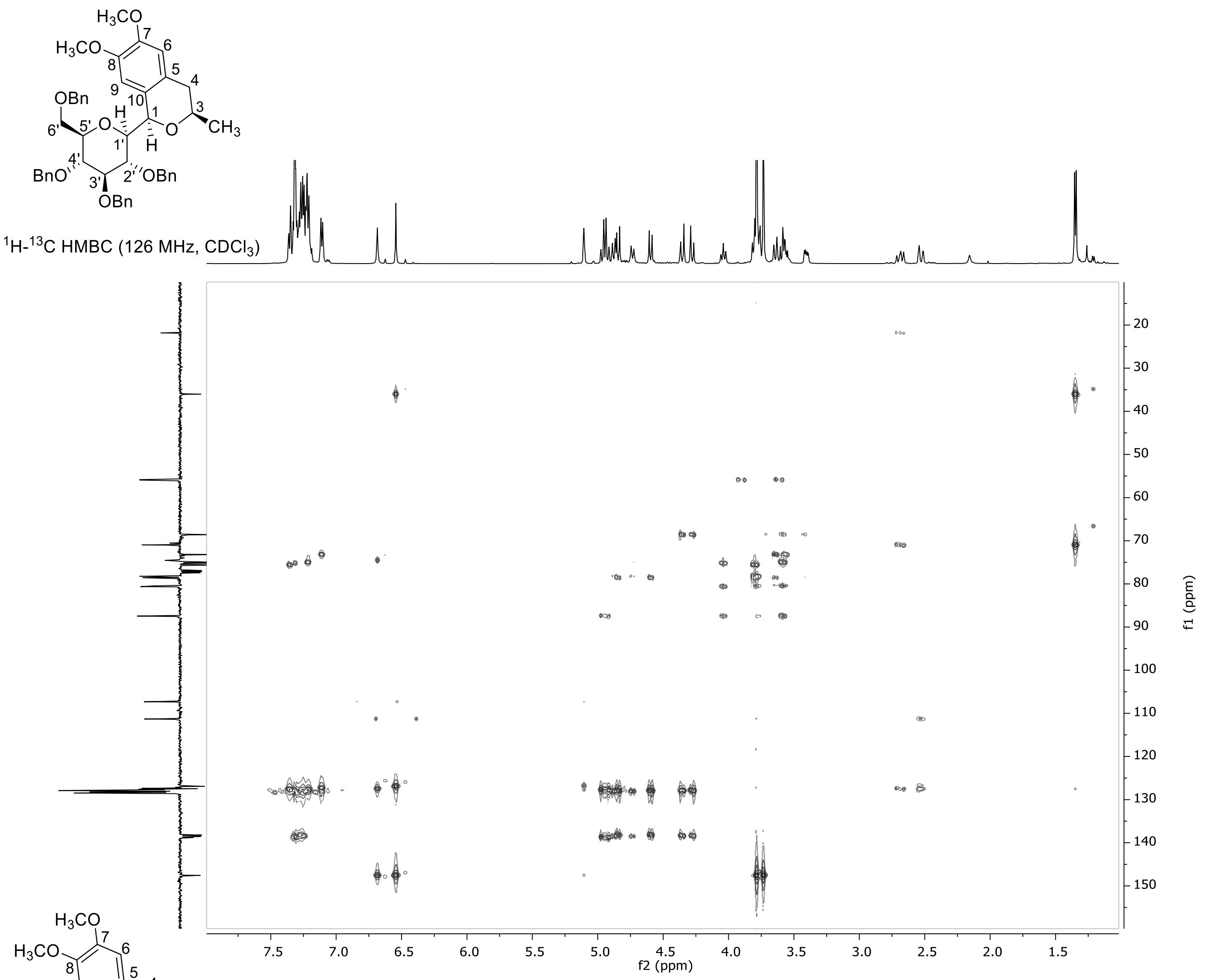
H3CO
H3CO
OBn
BnO
OBn
OBn
CH3
1H-13C HMBC (126 MHz, CDCl3)
f2 (ppm)
f1 (ppm)

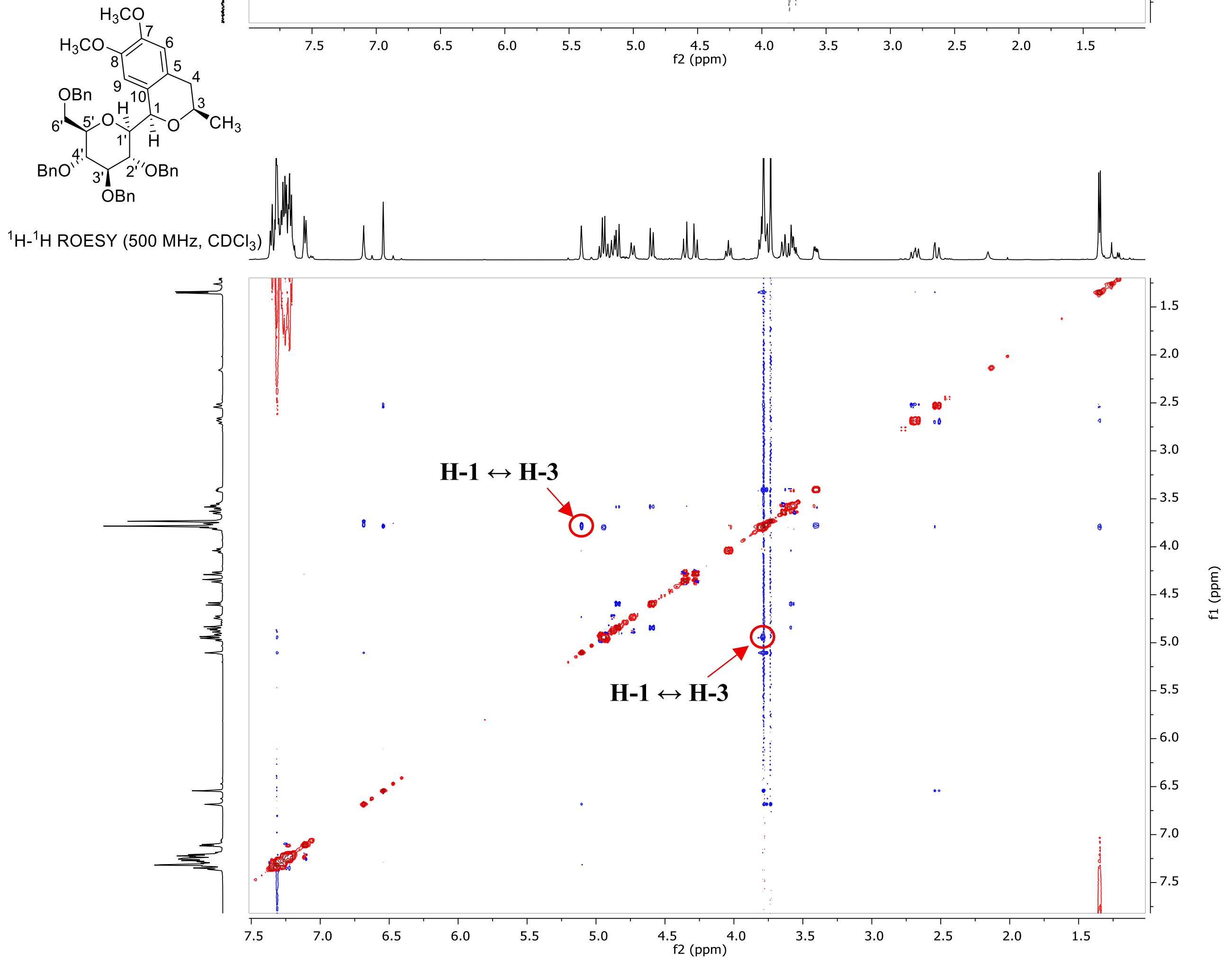
H3CO
H3CO
OBn
BnO
OBn
OBn
CH3
1H-1H ROESY (500 MHz, CDCl3)
H-1 ↔ H-3
H-1 ↔ H-3
f2 (ppm)
f1 (ppm)

Compound 44

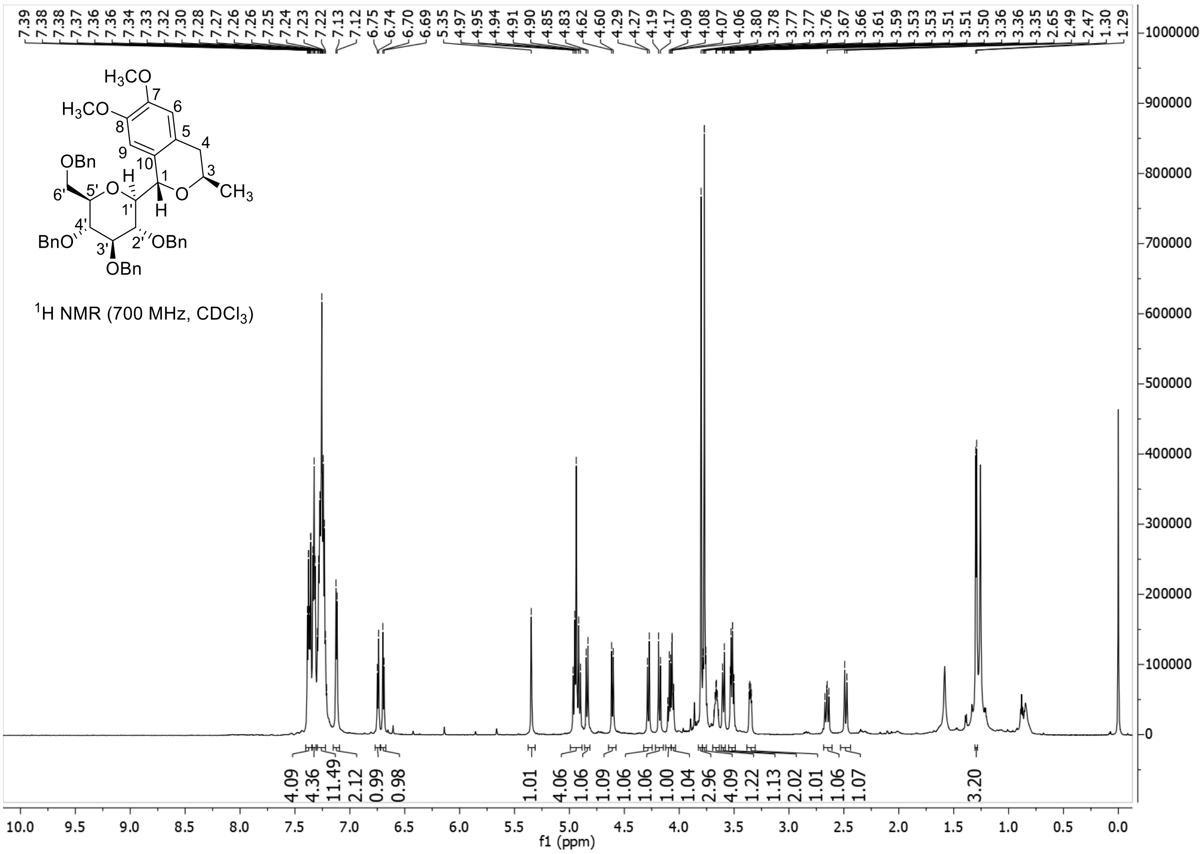

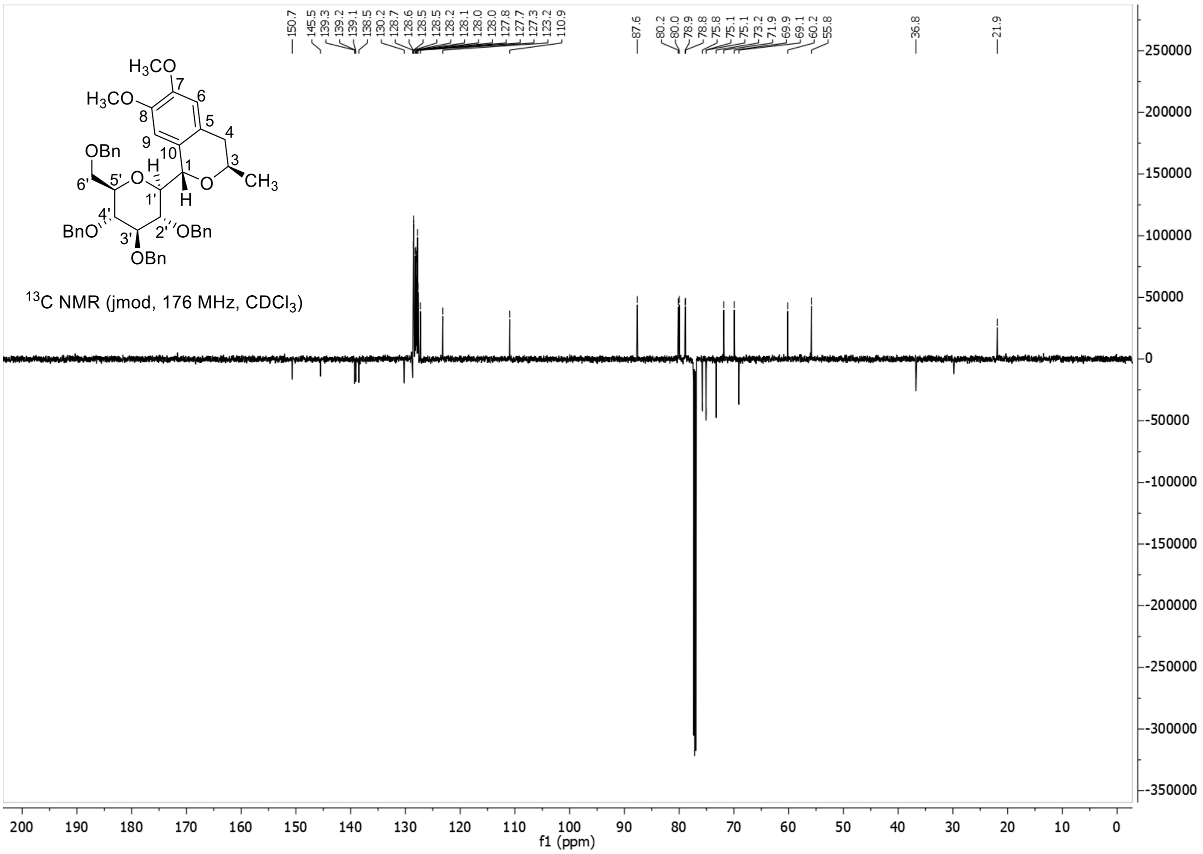

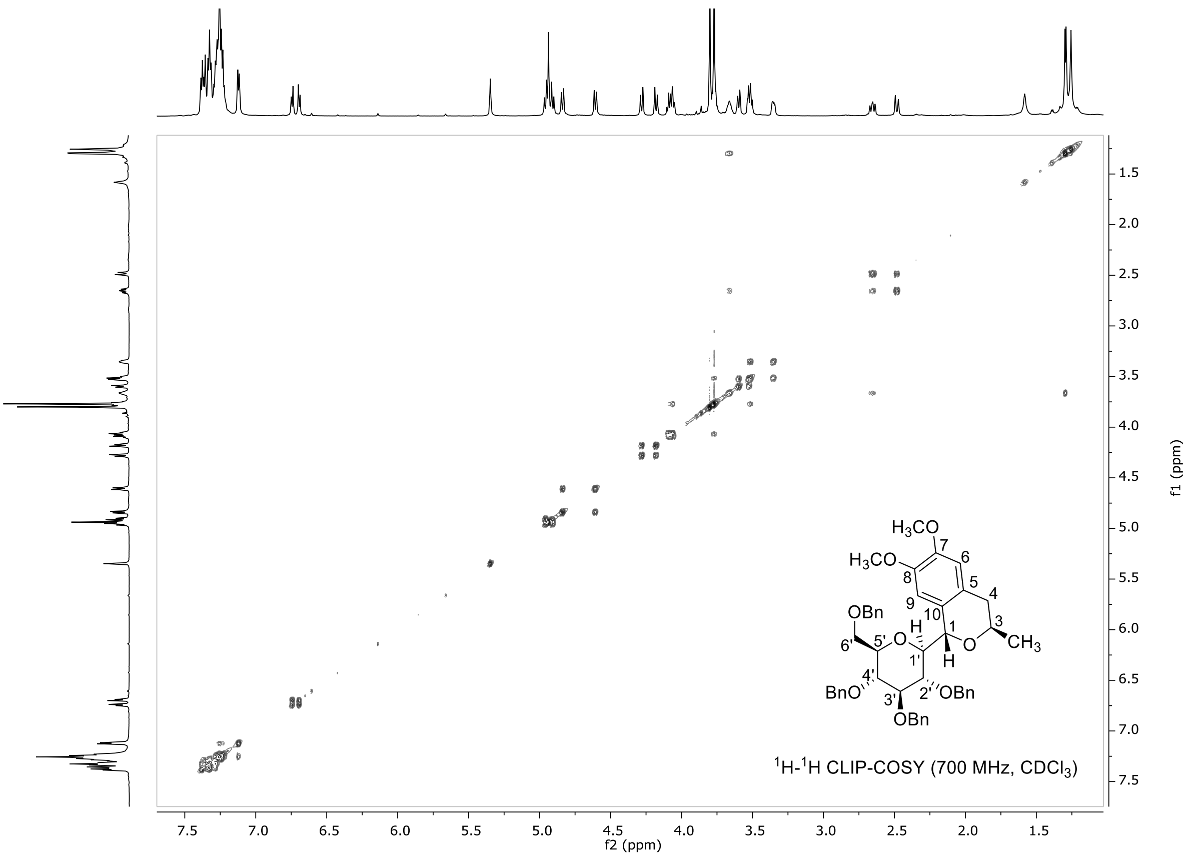
H3CO
H3CO
OBn
BnO
OBn
OBn
CH3
1H-1H CLIP-COSY (700 MHz, CDCl3)
f2 (ppm)
f1 (ppm)

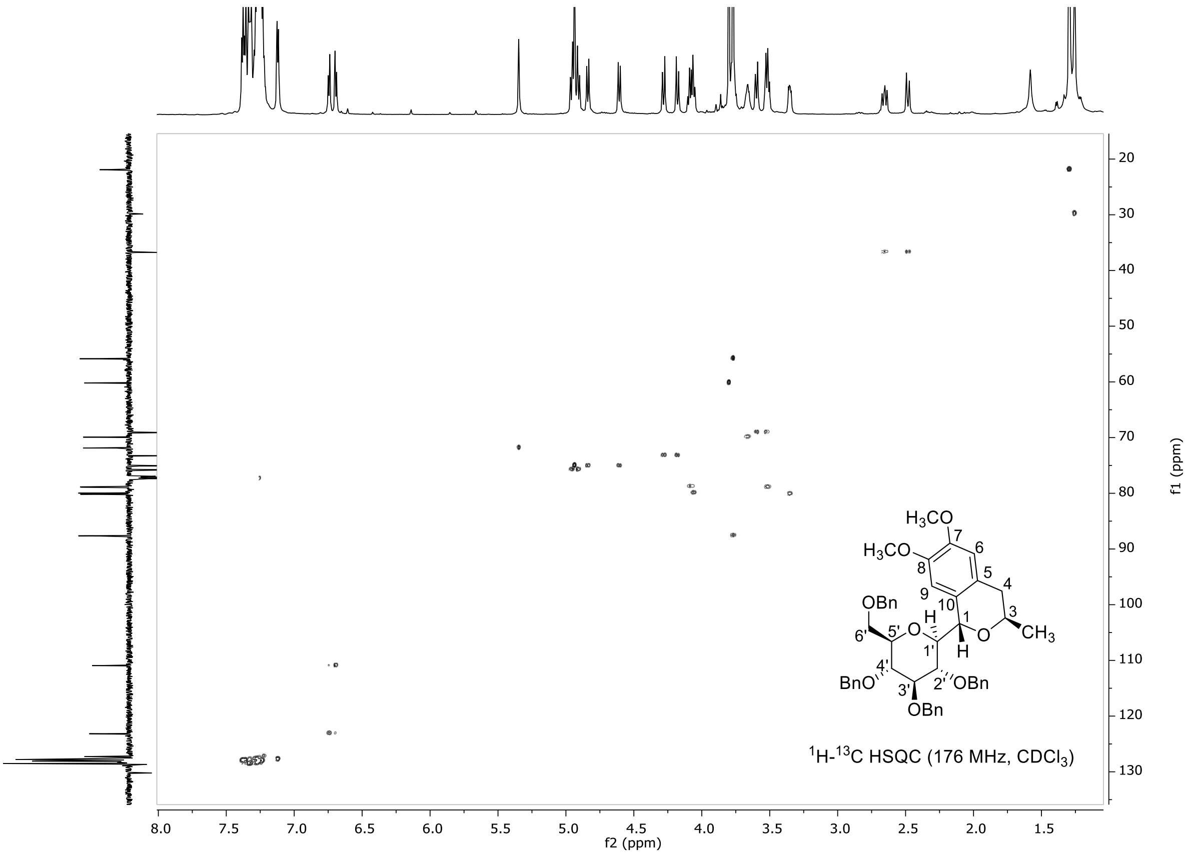
H3CO
H3CO
OBn
BnO
OBn
OBn
CH3
1H-13C HSQC (176 MHz, CDCl3)
f2 (ppm)
f1 (ppm)

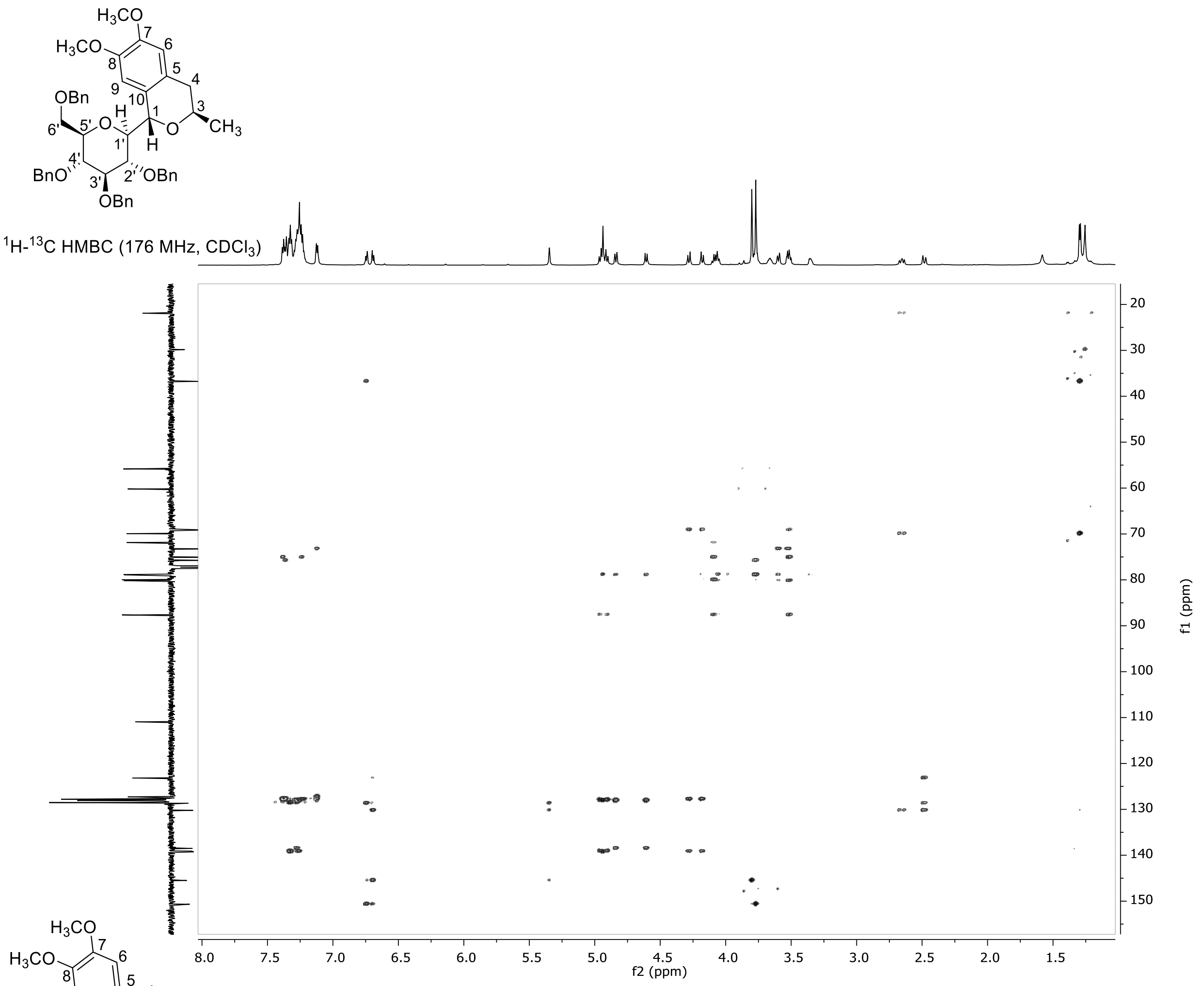

^{1}H-^{13}C HMBC (176 MHz, $CDCl_3$)

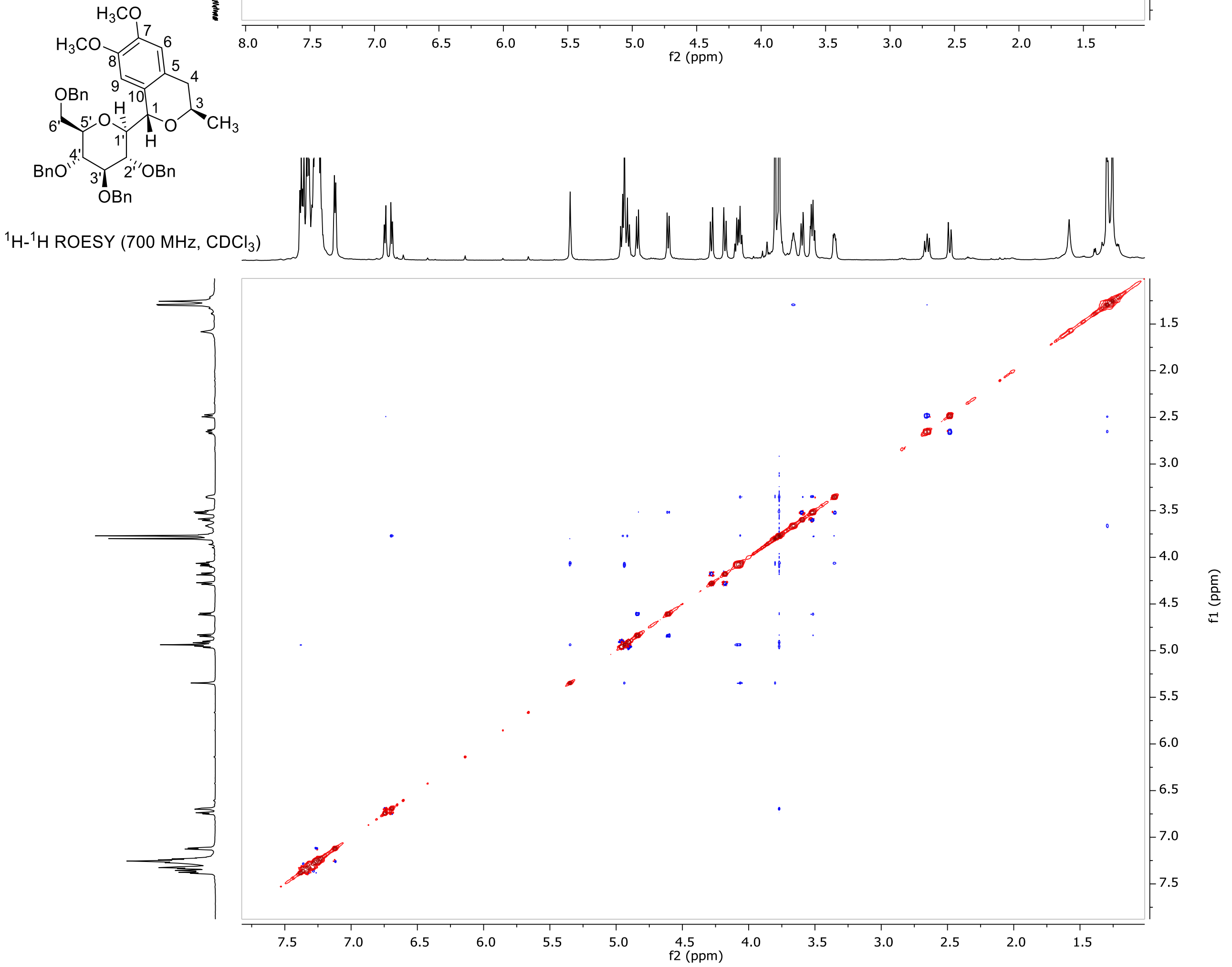

^{1}H-^{1}H ROESY (700 MHz, $CDCl_3$)

Compound 45

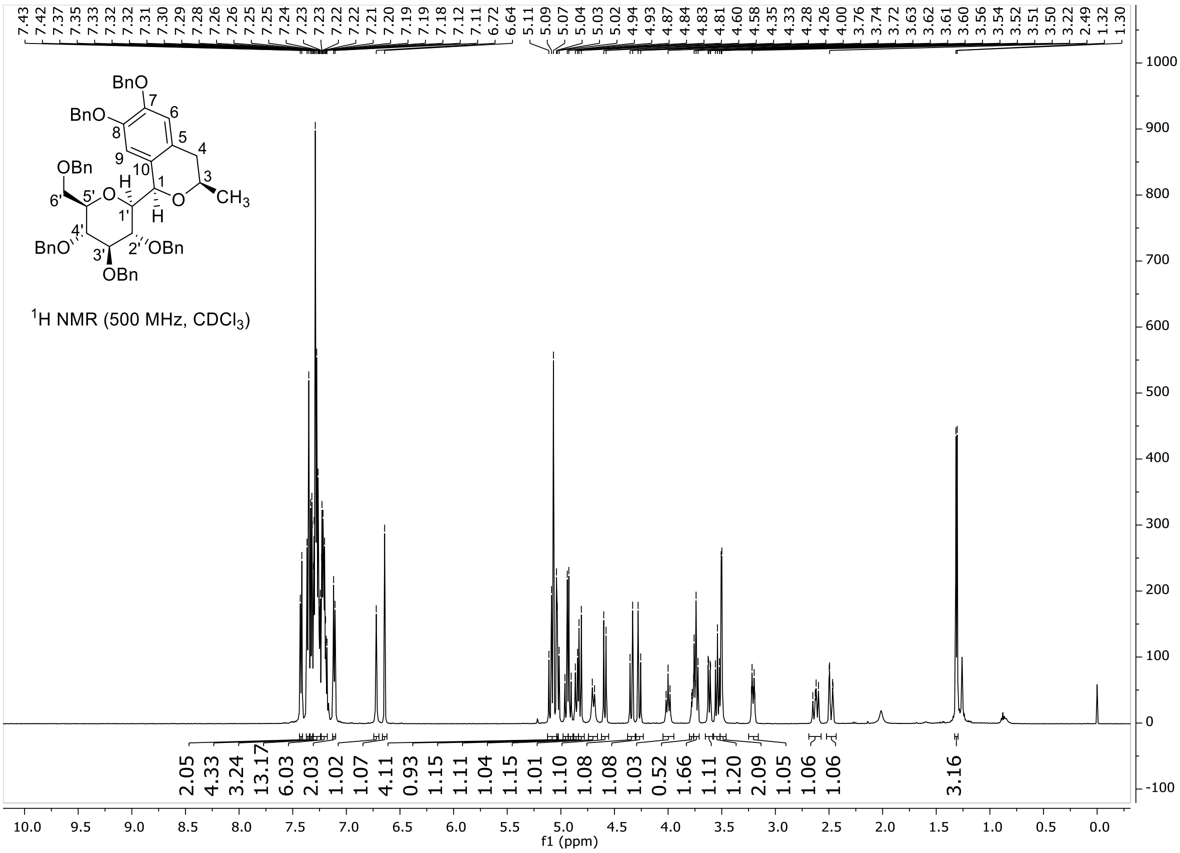

7.43 7.42 7.37 7.35 7.33 7.32 7.32 7.31 7.30 7.29 7.28 7.26 7.26 7.25 7.25 7.24 7.23 7.23 7.22 7.22 7.21 7.20 7.19 7.19 7.18 7.12 7.11 6.72 6.64 5.11 5.09 5.07 5.04 5.03 5.02 4.94 4.93 4.87 4.84 4.83 4.81 4.60 4.58 4.35 4.33 4.28 4.26 4.00 3.76 3.74 3.72 3.63 3.62 3.61 3.60 3.56 3.54 3.52 3.51 3.50 3.22 2.49 1.32 1.30
BnO
BnO
OBn
BnO
OBn
OBn
CH3
H
H
O
O
^{1}H NMR (500 MHz, CDCl$_3$)
2.05 4.33 3.24 13.17 6.03 2.03 1.02 1.07 4.11 0.93 1.15 1.11 1.04 1.15 1.01 1.10 1.08 1.08 1.03 0.52 1.66 1.11 1.20 2.09 1.05 1.06 1.06 3.16
f1 (ppm)

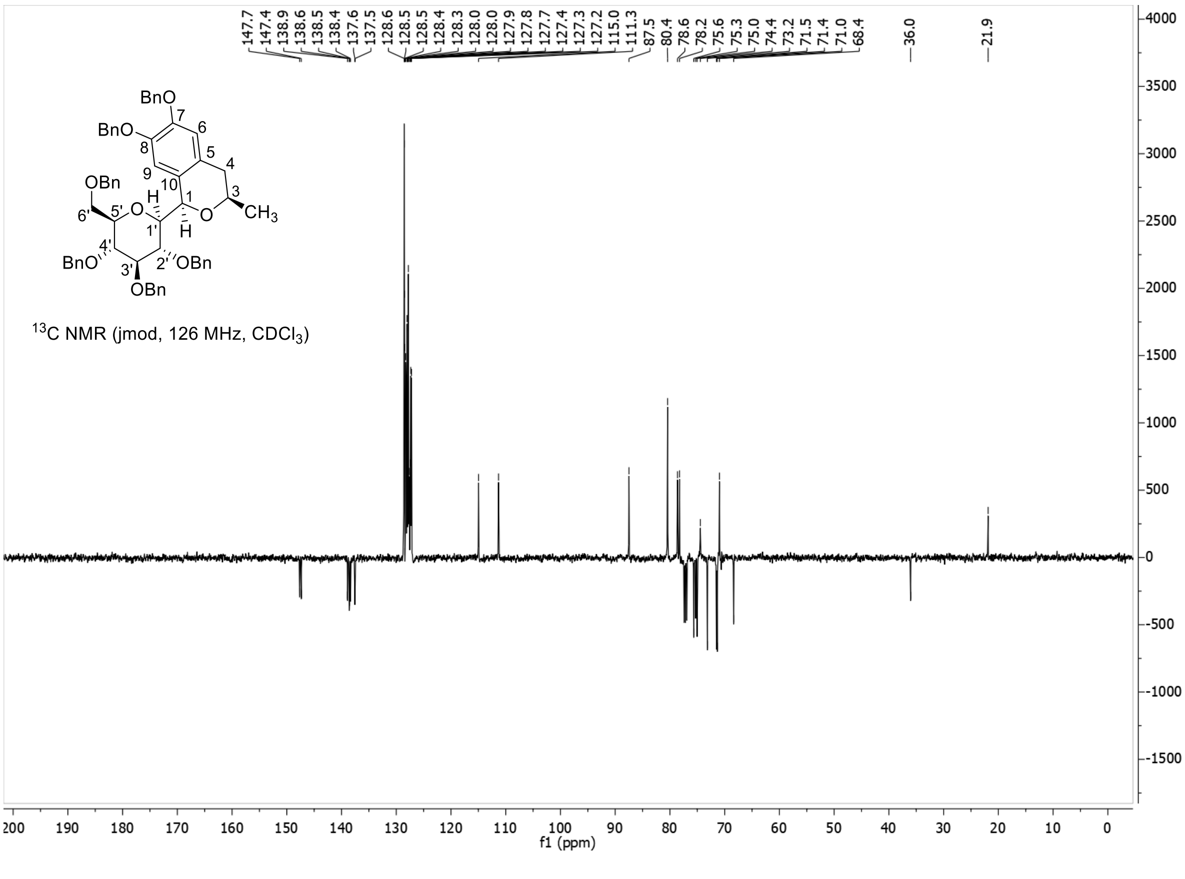

147.7 147.4 138.9 138.6 138.5 138.4 137.6 137.5 128.6 128.5 128.5 128.4 128.3 128.0 128.0 127.9 127.8 127.7 127.4 127.3 127.2 115.0 111.3 87.5 80.4 78.6 78.2 75.6 75.3 75.0 74.4 73.2 71.5 71.4 71.0 68.4 36.0 21.9
^{13}C NMR (jmod, 126 MHz, CDCl$_3$)
f1 (ppm)

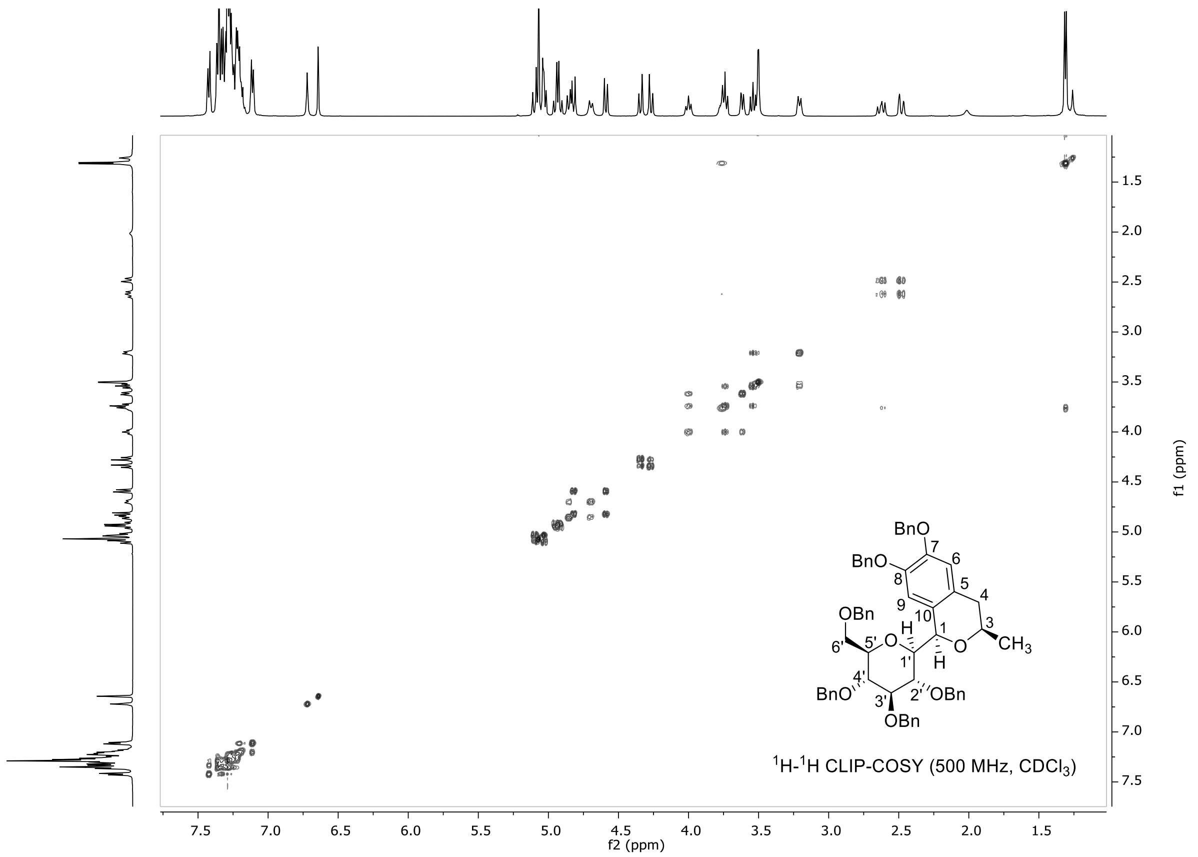
BnO
7
BnO
6
8
5
4
9
OBn
10
H
1
3
6'
5'
O
O
CH3
1'
H
4'
BnO
3'
2'
OBn
OBn
1H-1H CLIP-COSY (500 MHz, CDCl3)
f2 (ppm)
f1 (ppm)

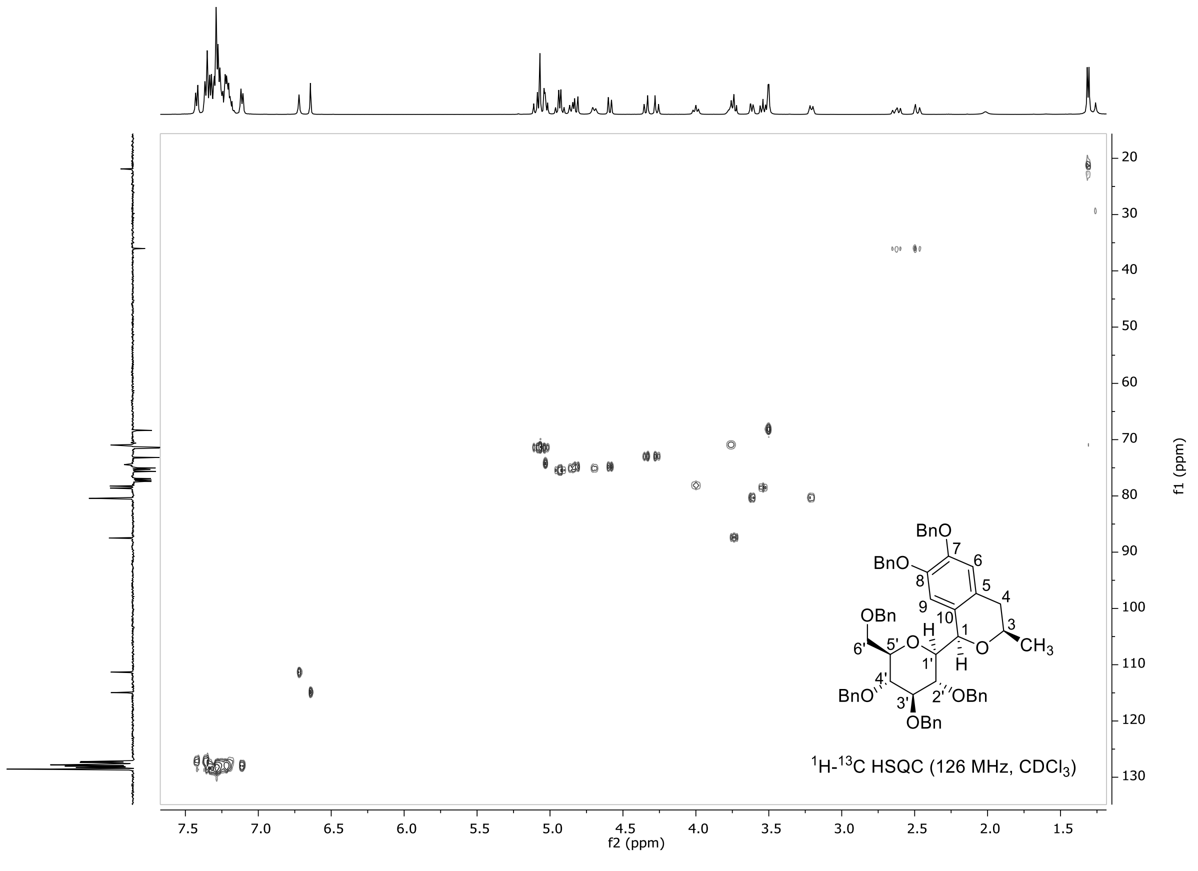
BnO
7
BnO
6
8
5
4
9
OBn
10
H
1
3
6'
5'
O
O
CH3
1'
H
4'
BnO
3'
2'
OBn
OBn
1H-13C HSQC (126 MHz, CDCl3)
f2 (ppm)
f1 (ppm)

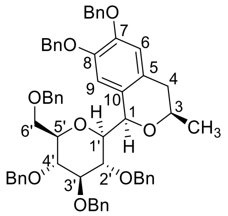

^{1}H-^{13}C HMBC (126 MHz, $CDCl_3$)

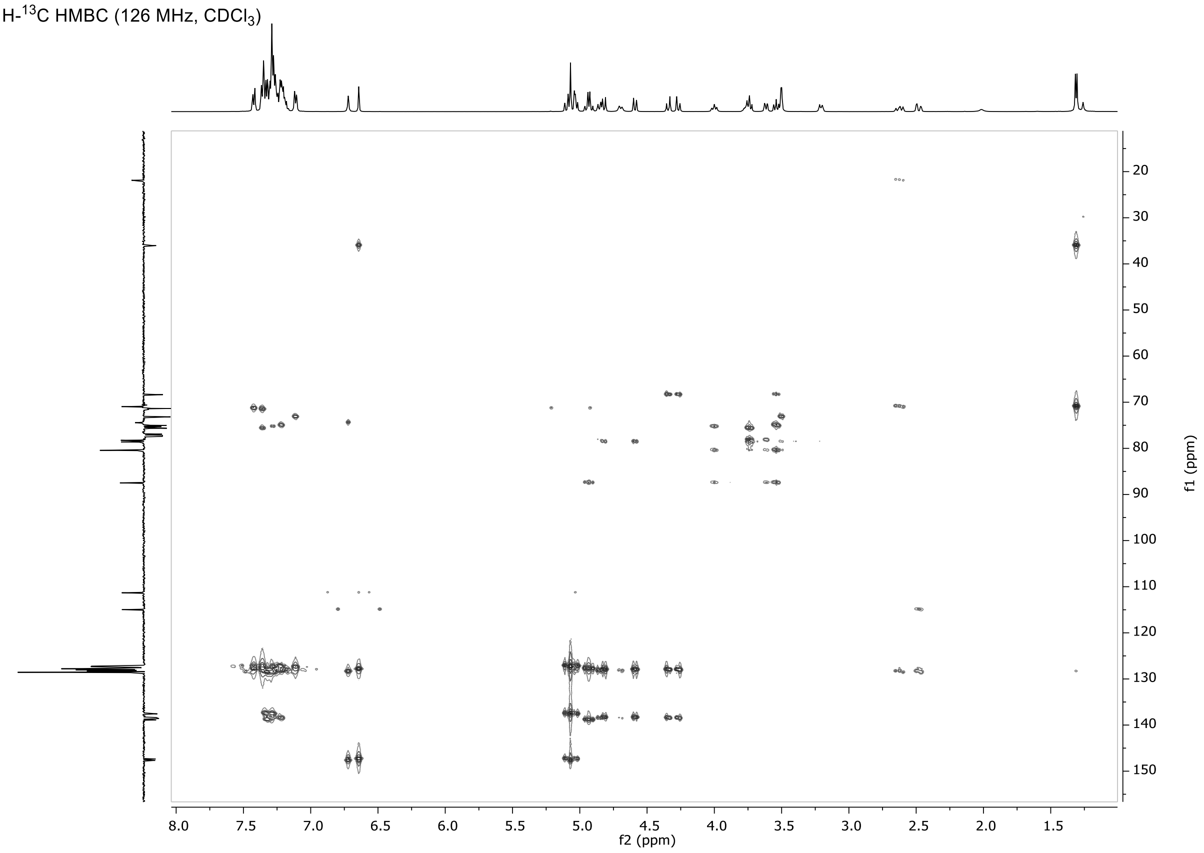

Compound 46

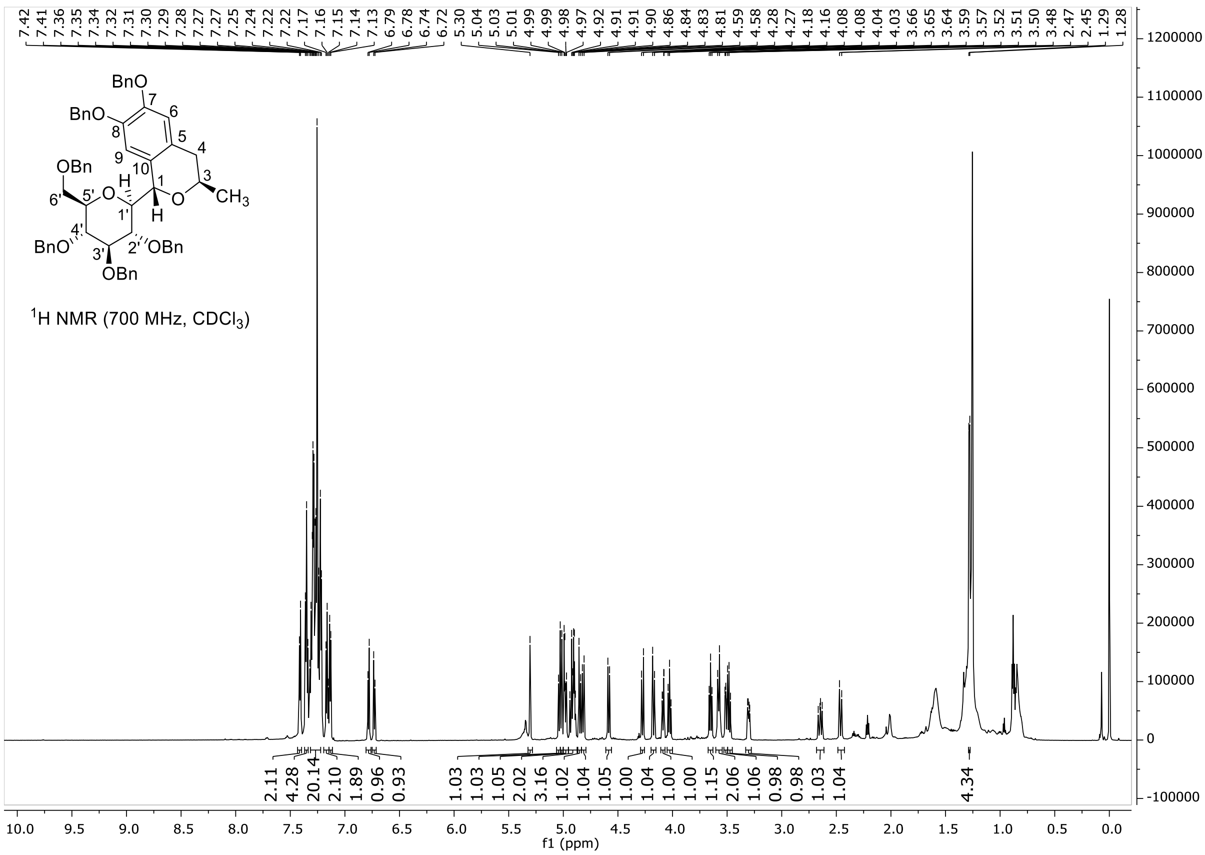

BnO
BnO
OBn
BnO
OBn
OBn
CH3
H
H
1H NMR (700 MHz, CDCl3)
f1 (ppm)

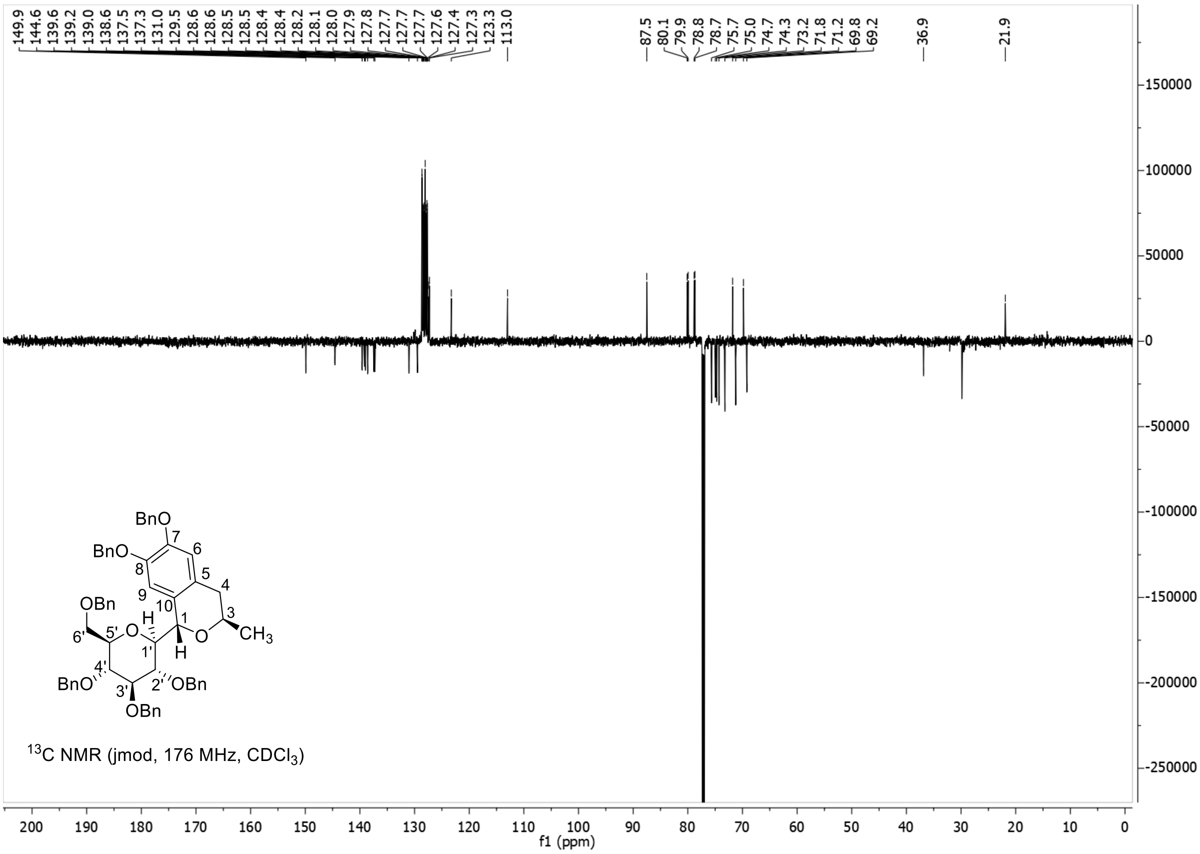

BnO
BnO
OBn
BnO
OBn
OBn
CH3
H
H
13C NMR (jmod, 176 MHz, CDCl3)
f1 (ppm)

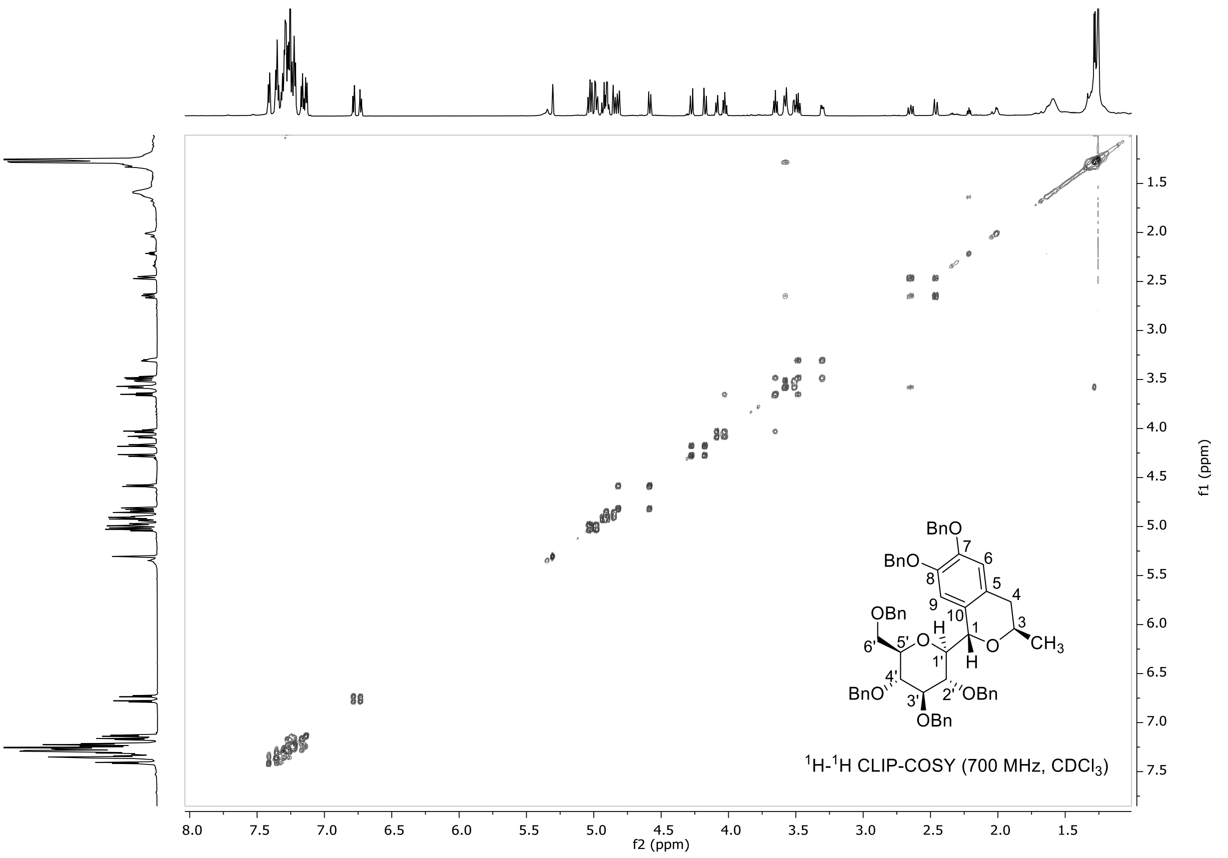
BnO
BnO
OBn
BnO
OBn
OBn
CH3
1H-1H CLIP-COSY (700 MHz, CDCl3)
f2 (ppm)
f1 (ppm)

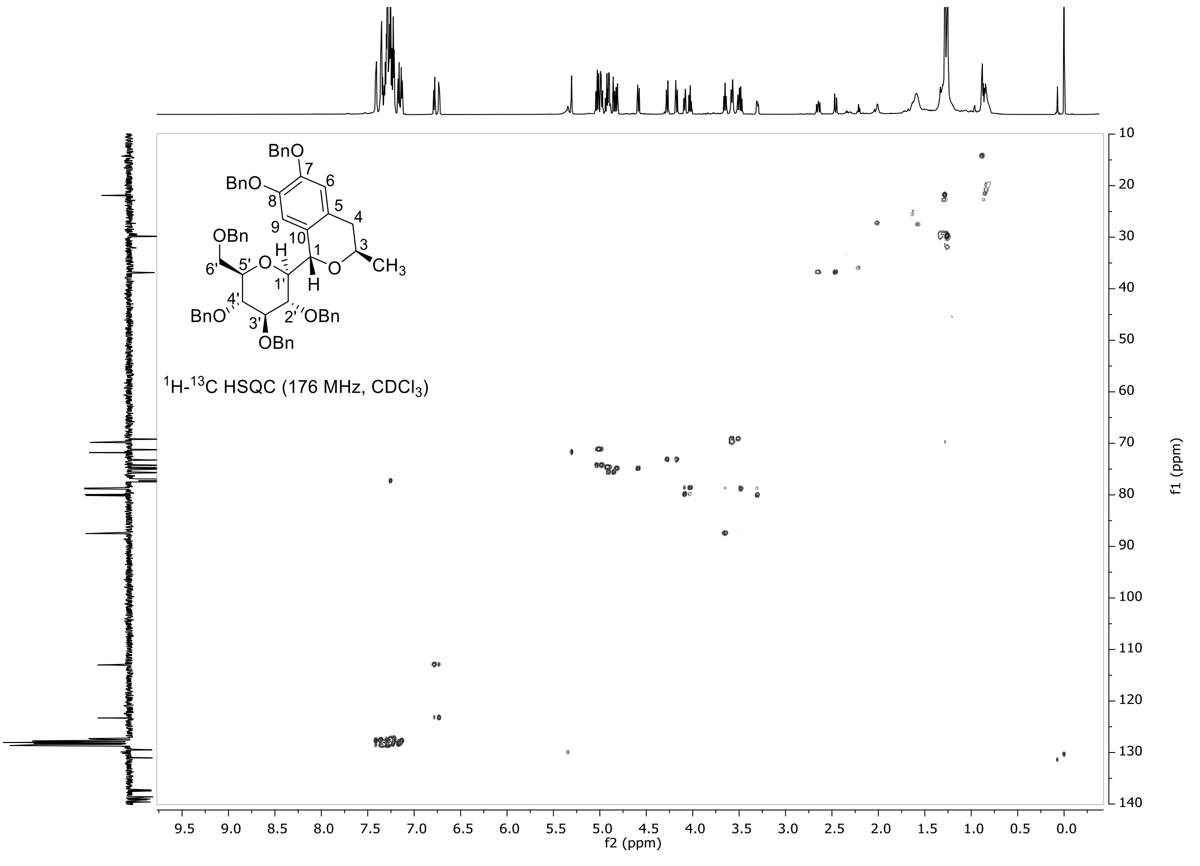
BnO
BnO
OBn
BnO
OBn
OBn
CH3
1H-13C HSQC (176 MHz, CDCl3)
f2 (ppm)
f1 (ppm)

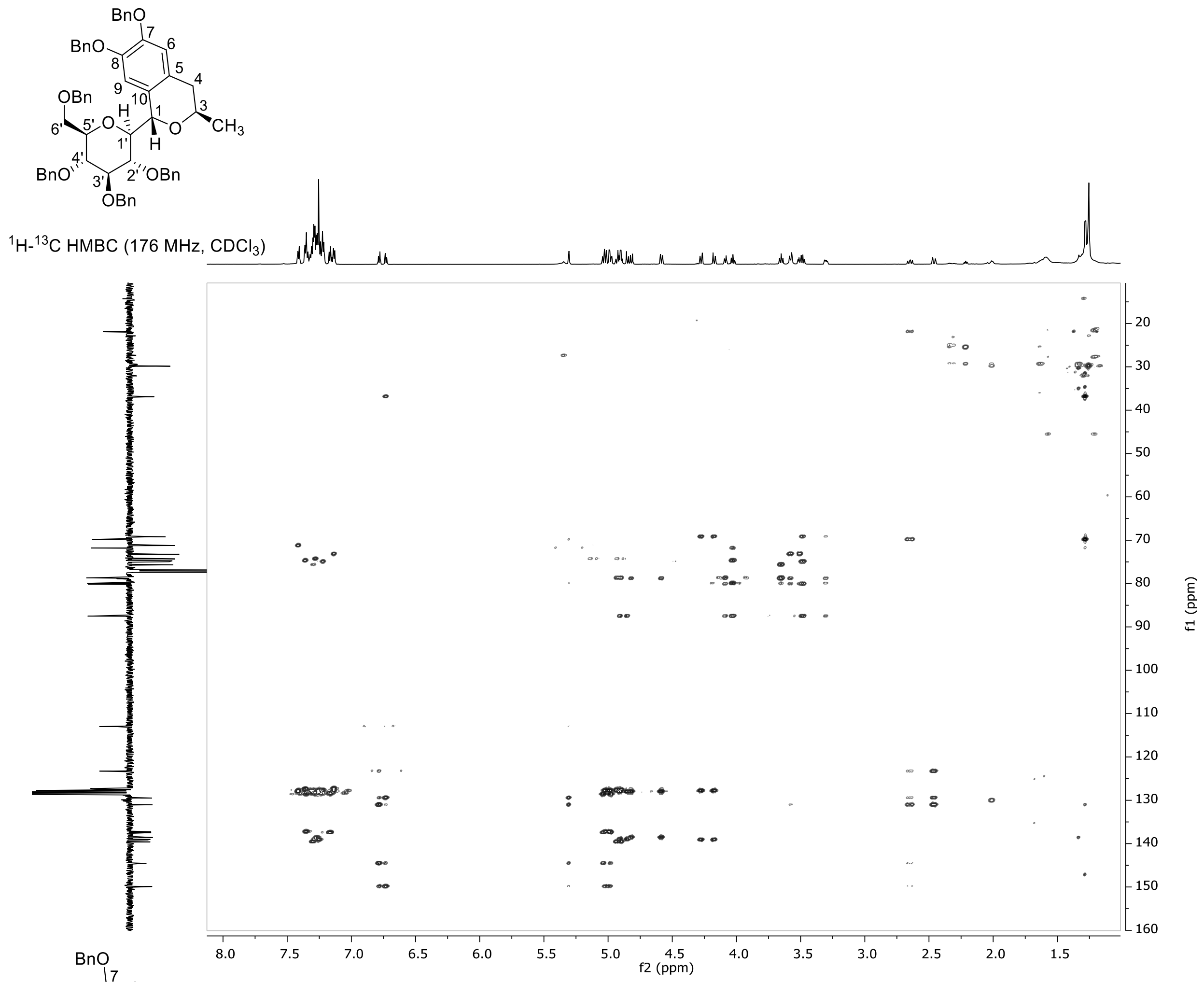

BnO
BnO
OBn
CH3
BnO
OBn
OBn
1H-13C HMBC (176 MHz, CDCl3)
f2 (ppm)
f1 (ppm)

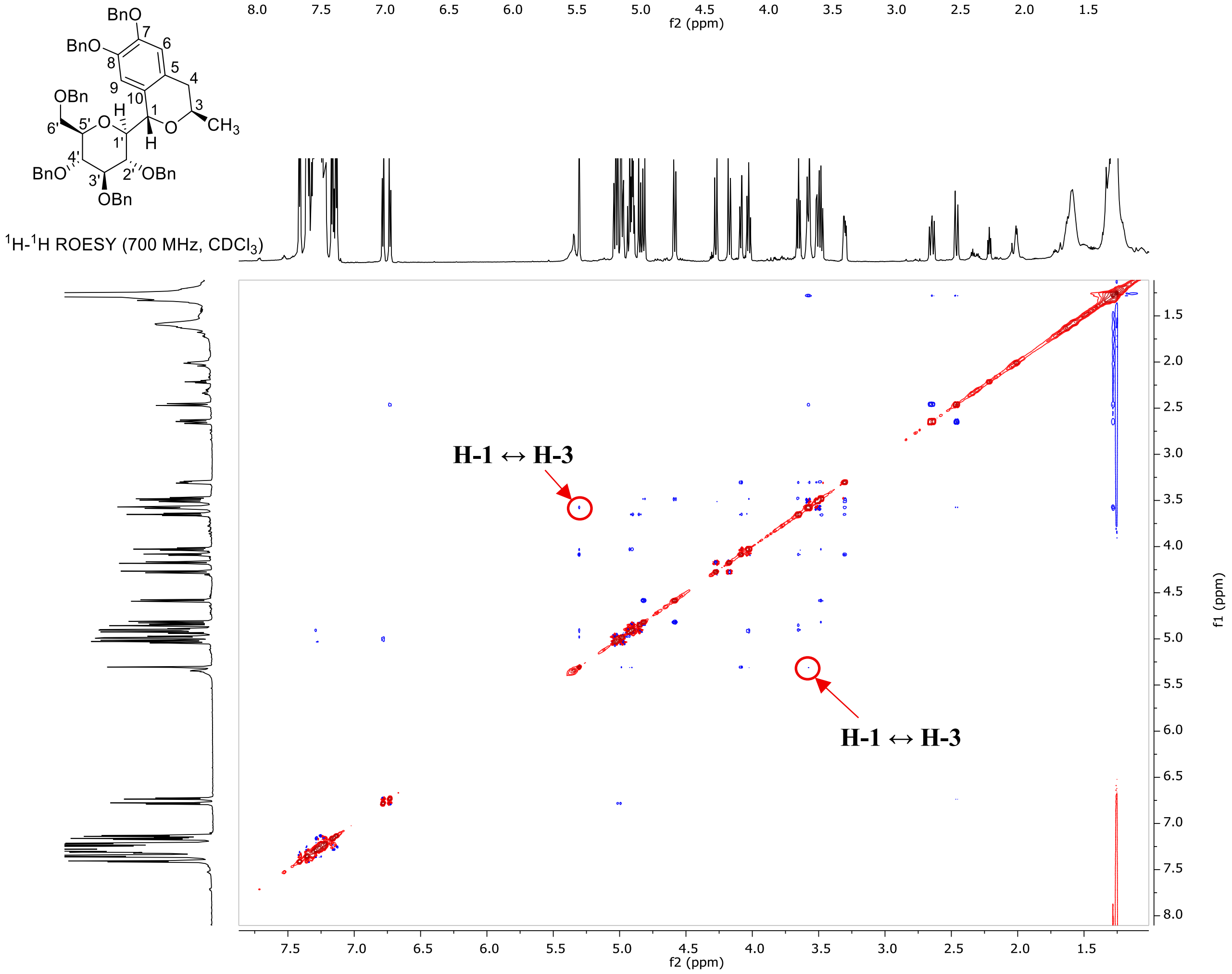

BnO
BnO
OBn
CH3
BnO
OBn
OBn
1H-1H ROESY (700 MHz, CDCl3)
H-1 ↔ H-3
H-1 ↔ H-3
f2 (ppm)
f1 (ppm)

Compound 47

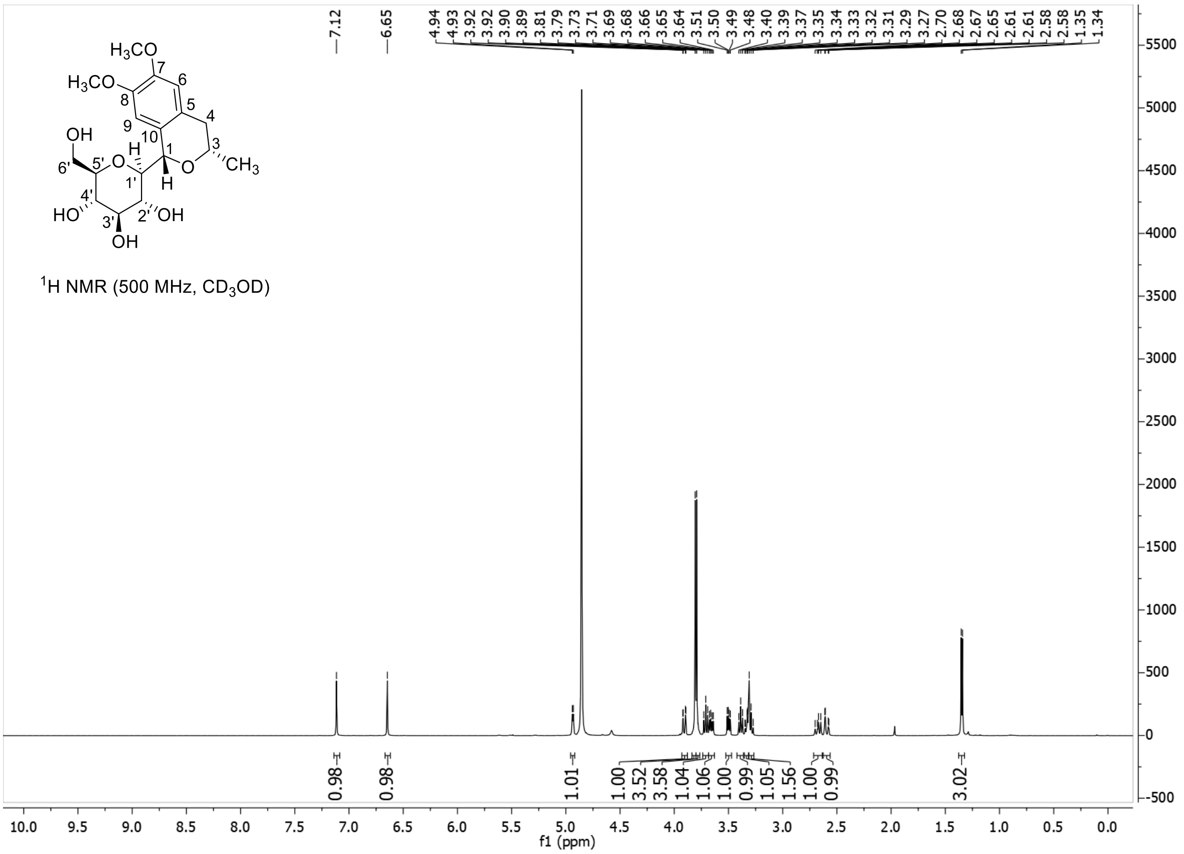

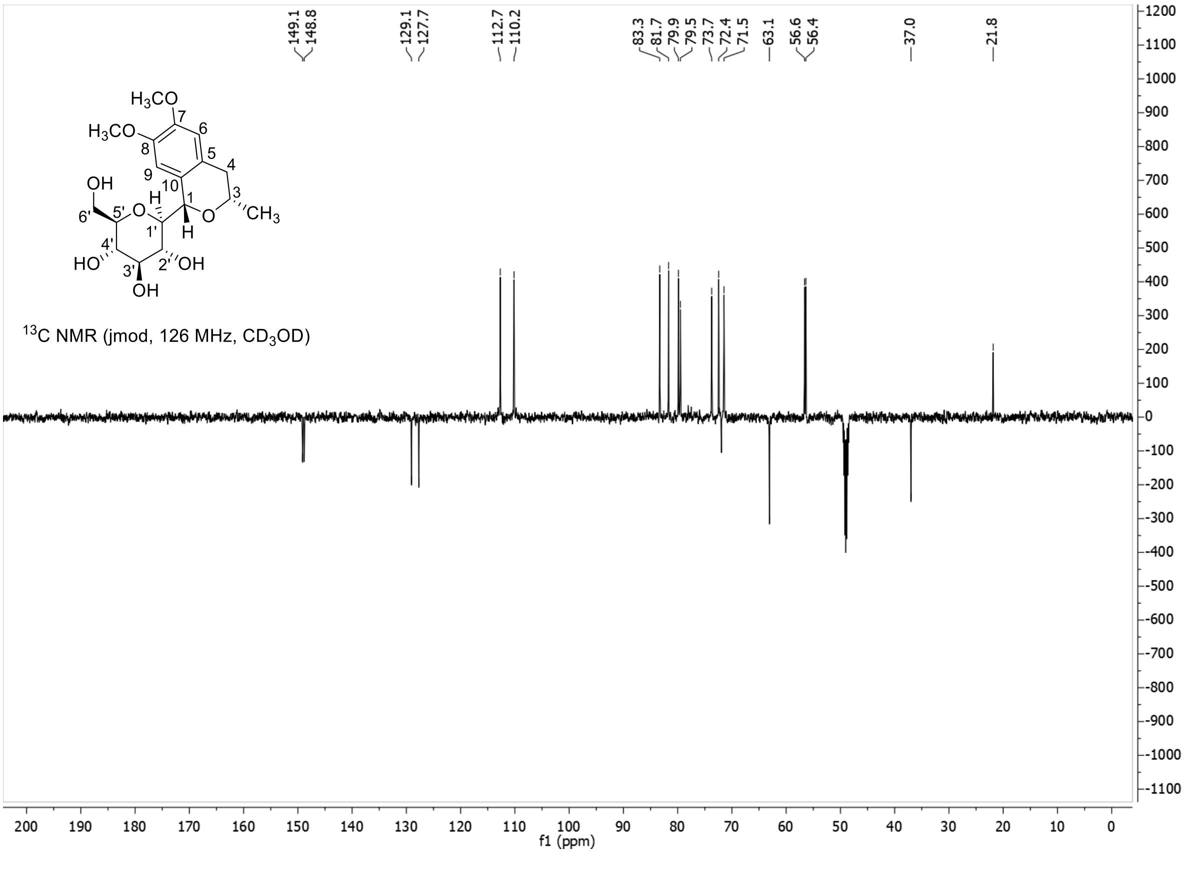

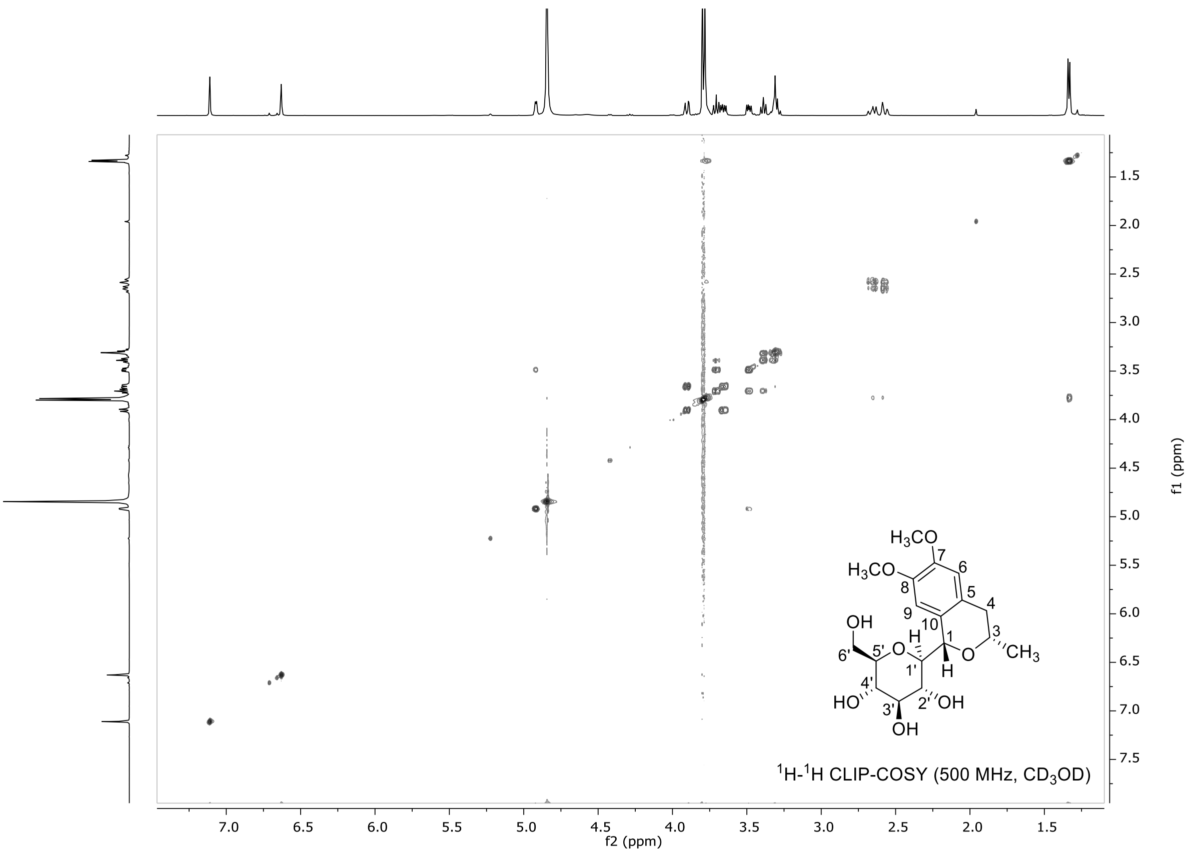
H3CO
H3CO
7
6
8
5
4
9
10
1
3
OH
H
6'
5'
O
1'
O
CH3
H
4'
HO
3'
2'
OH
OH
1H-1H CLIP-COSY (500 MHz, CD3OD)
f1 (ppm)
f2 (ppm)

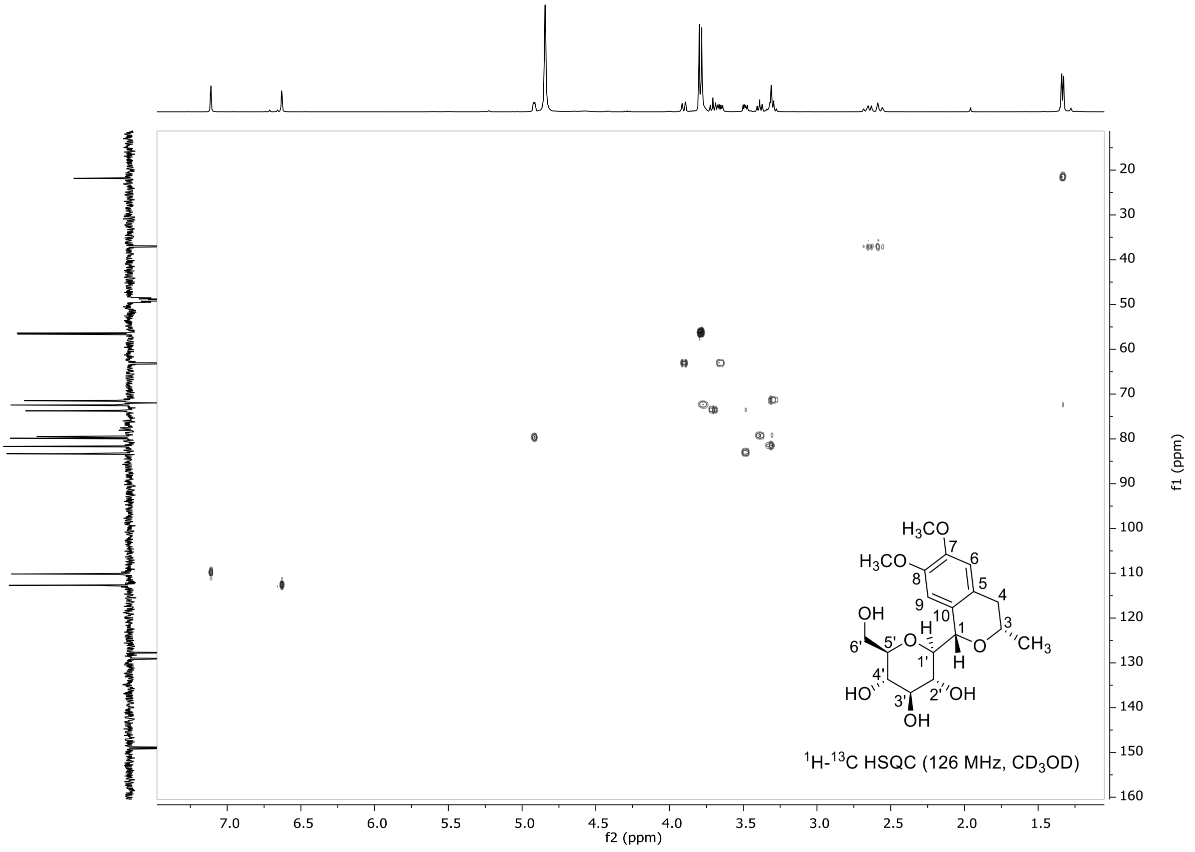
H3CO
H3CO
7
6
8
5
4
9
10
1
3
OH
H
6'
5'
O
1'
O
CH3
H
4'
HO
3'
2'
OH
OH
1H-13C HSQC (126 MHz, CD3OD)
f1 (ppm)
f2 (ppm)

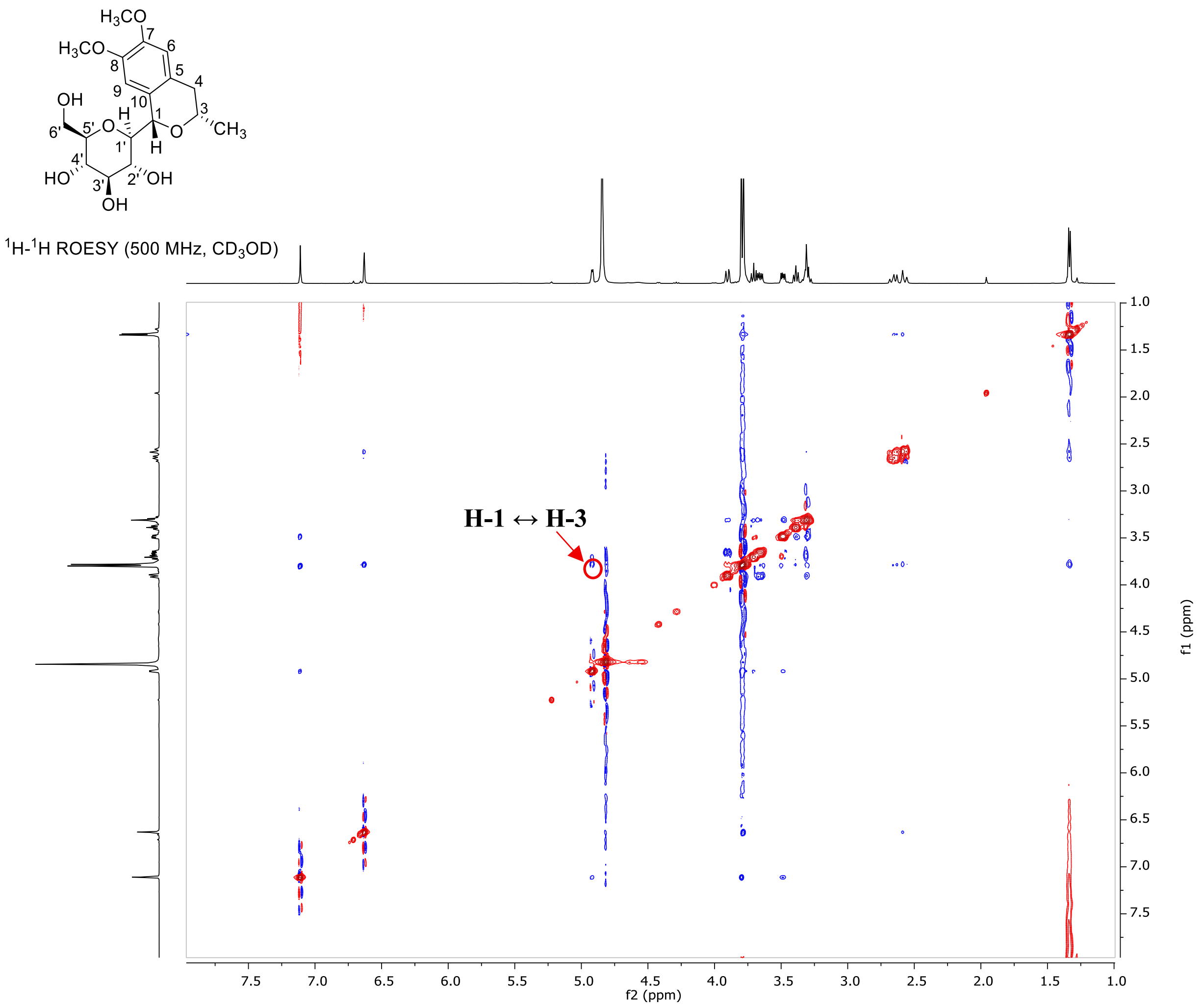
H3CO
H3CO
OH
HO
OH
OH
CH3
^{1}H-^{1}H ROESY (500 MHz, CD3OD)
H-1 ↔ H-3
f1 (ppm)
f2 (ppm)

Compound 48

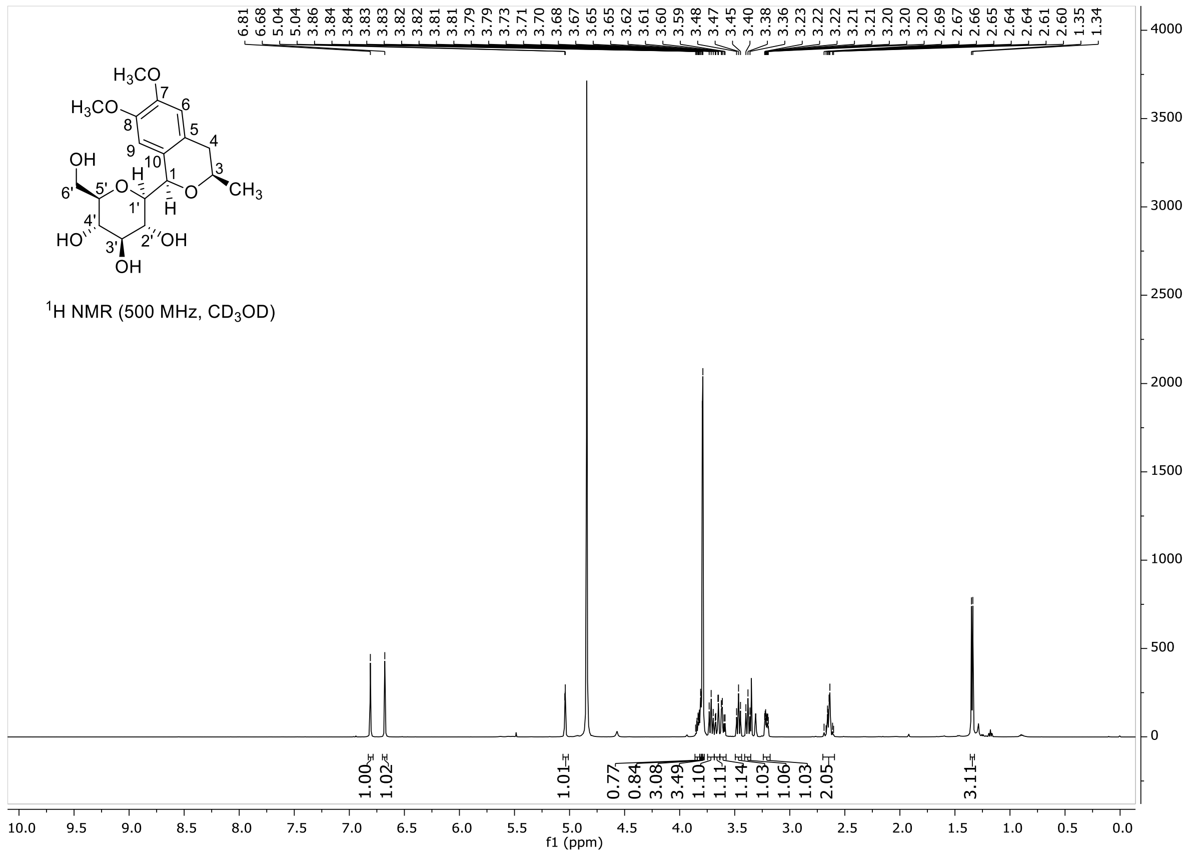

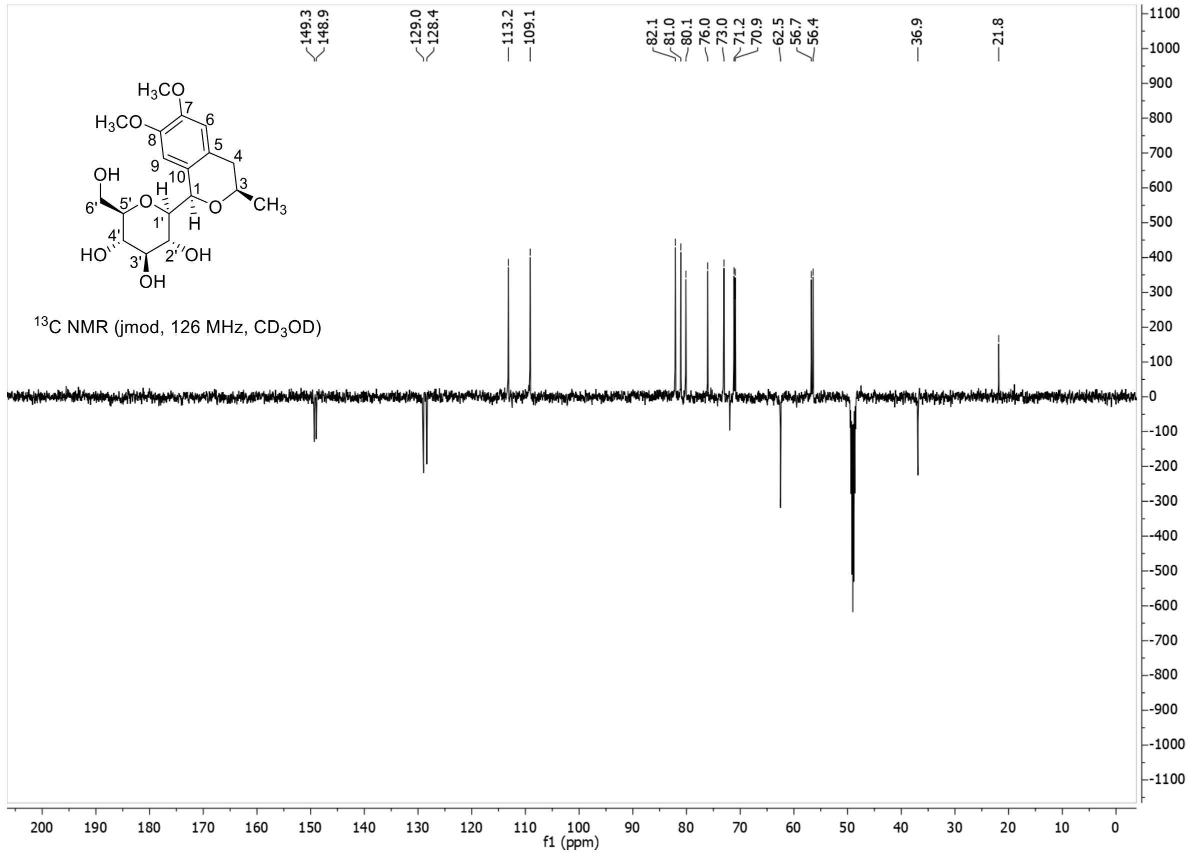

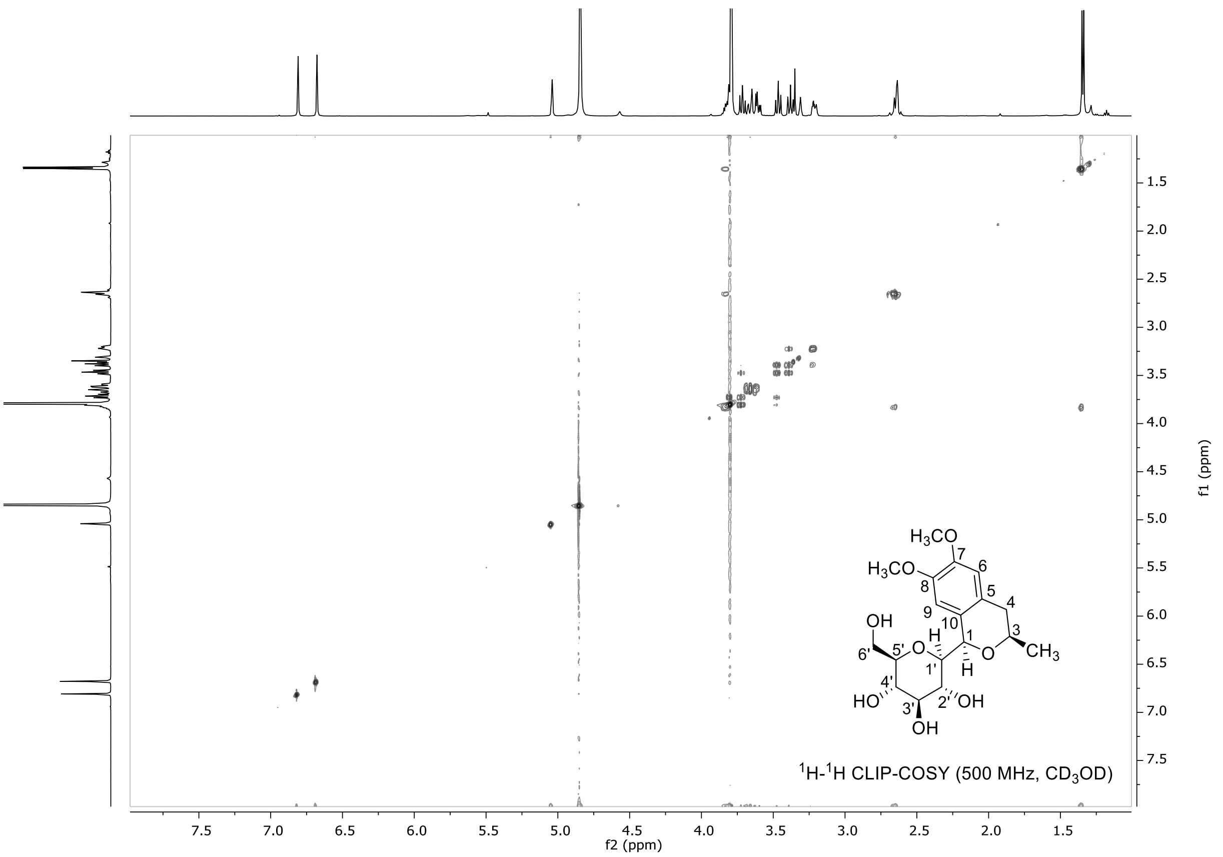

1H-1H CLIP-COSY (500 MHz, CD3OD)
f2 (ppm)
f1 (ppm)
H3CO
H3CO
OH
HO
OH
OH
CH3

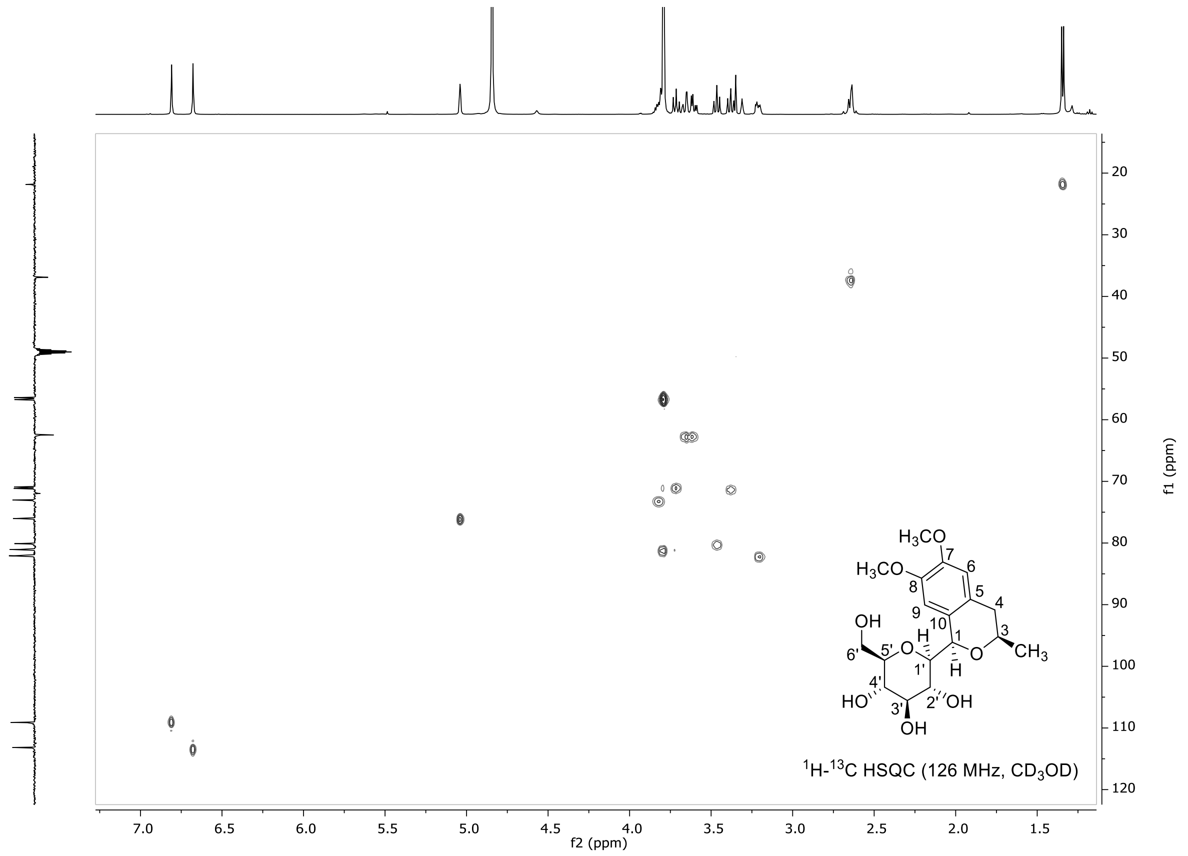

1H-13C HSQC (126 MHz, CD3OD)
f2 (ppm)
f1 (ppm)
H3CO
H3CO
OH
HO
OH
OH
CH3

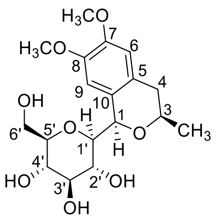

^{1}H-^{13}C HMBC (126 MHz, CD_3OD)

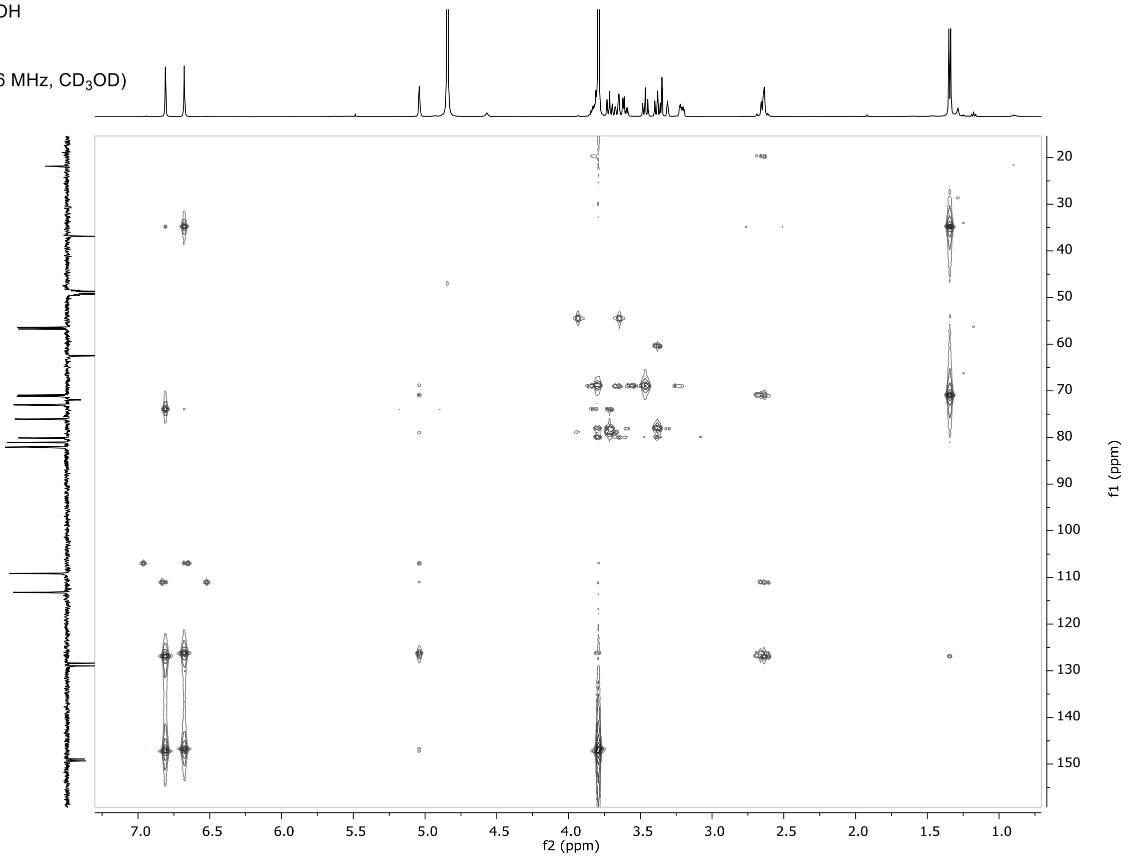

Compound 49

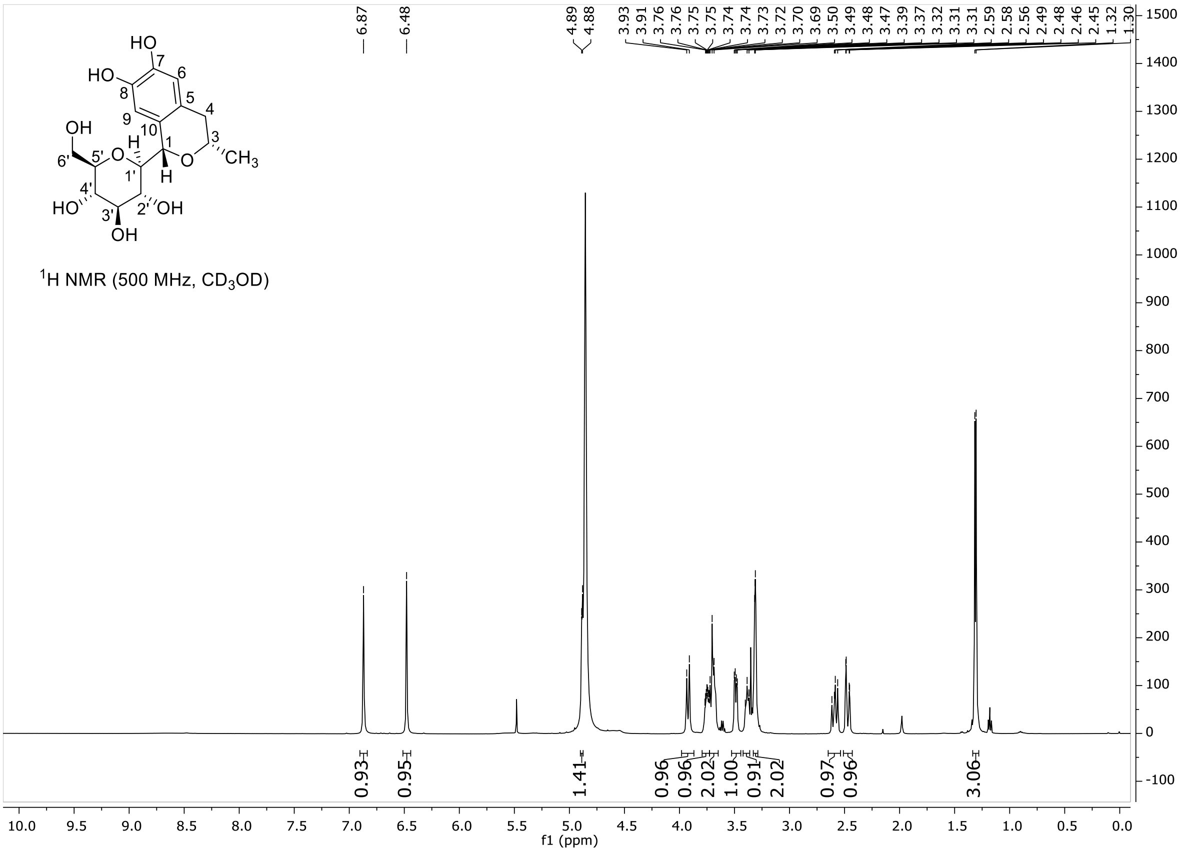

HO
HO
OH
O
H
H
O
CH3
HO
OH
OH
1H NMR (500 MHz, CD3OD)
6.87
6.48
4.89
4.88
3.93
3.91
3.76
3.76
3.75
3.75
3.74
3.74
3.73
3.72
3.70
3.69
3.50
3.49
3.48
3.47
3.39
3.37
3.32
3.31
3.31
2.59
2.58
2.56
2.49
2.48
2.46
2.45
1.32
1.30
0.93
0.95
1.41
0.96
0.96
2.02
1.00
0.91
2.02
0.97
0.96
3.06
f1 (ppm)

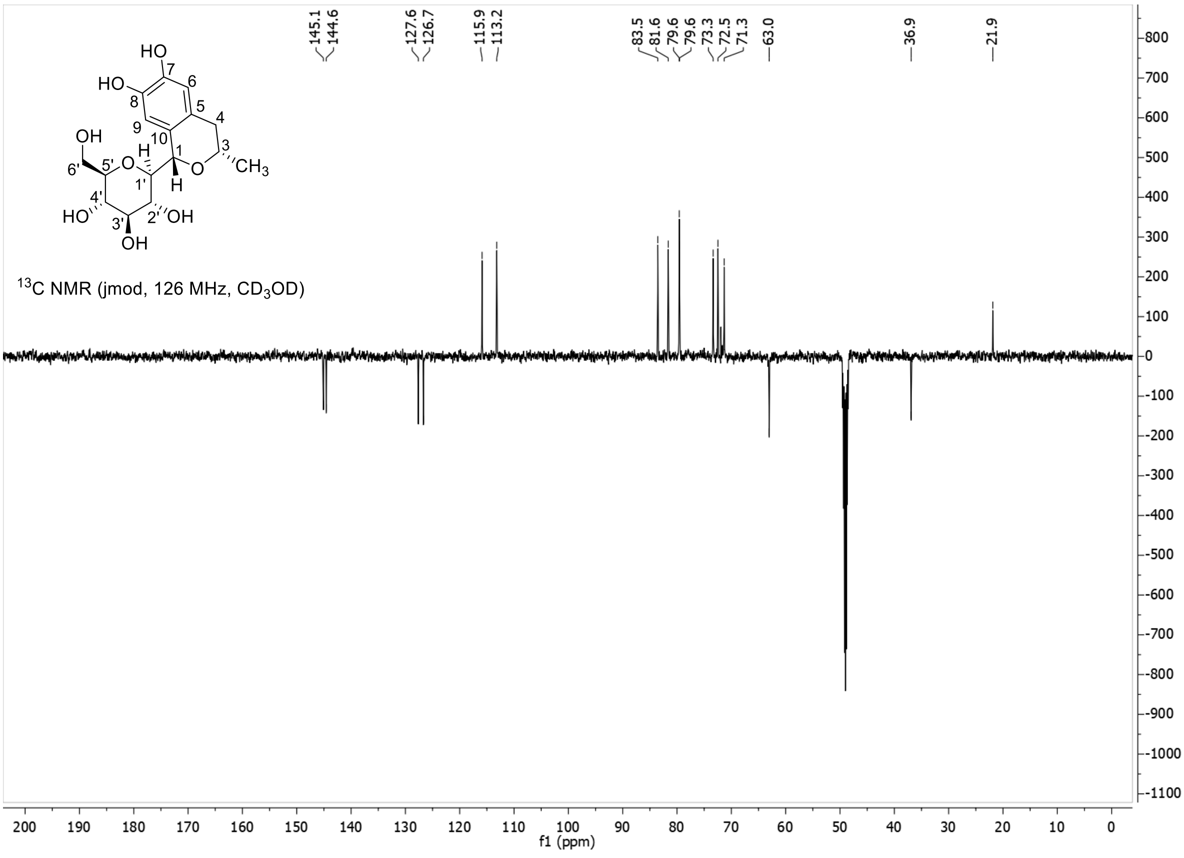

HO
HO
OH
O
H
H
O
CH3
HO
OH
OH
13C NMR (jmod, 126 MHz, CD3OD)
145.1
144.6
127.6
126.7
115.9
113.2
83.5
81.6
79.6
79.6
73.3
72.5
71.3
63.0
36.9
21.9
f1 (ppm)

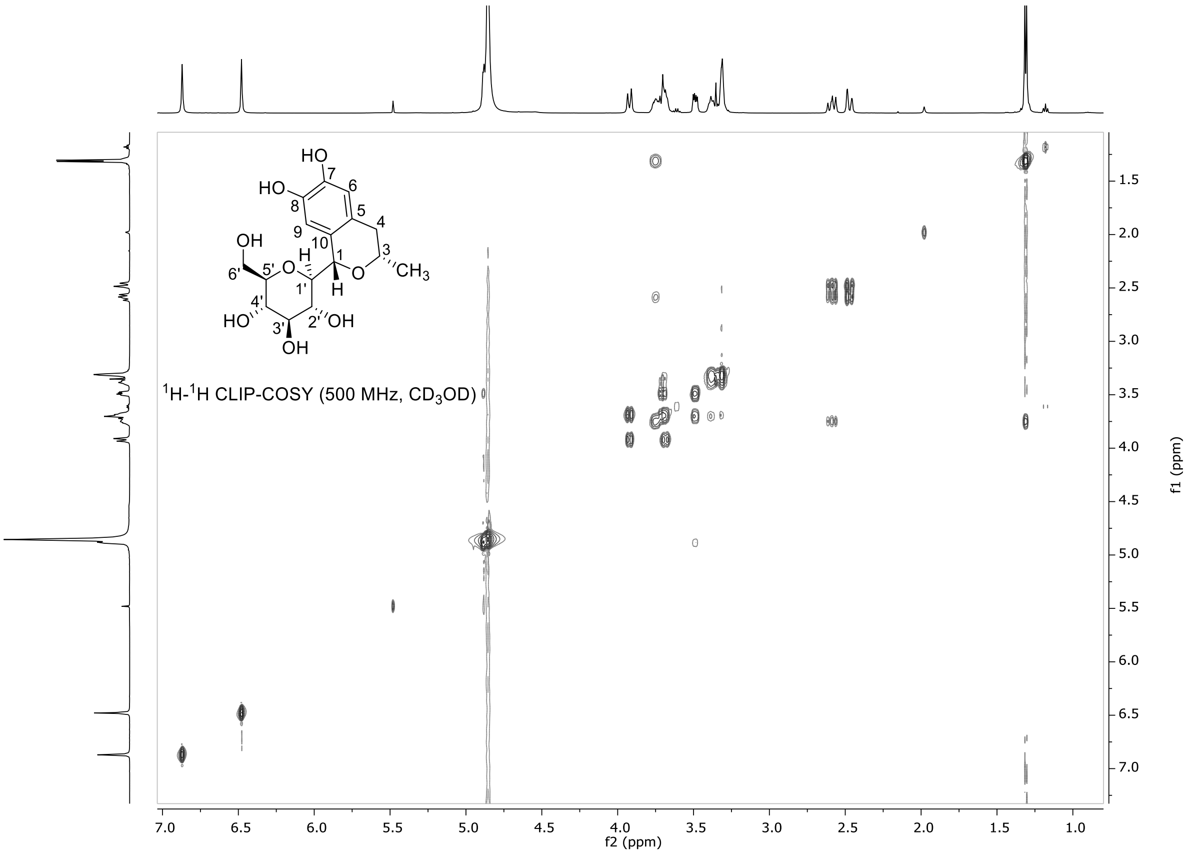
1H-1H CLIP-COSY (500 MHz, CD3OD)
f2 (ppm)
f1 (ppm)
HO
HO
OH
OH
HO
OH
OH
CH3

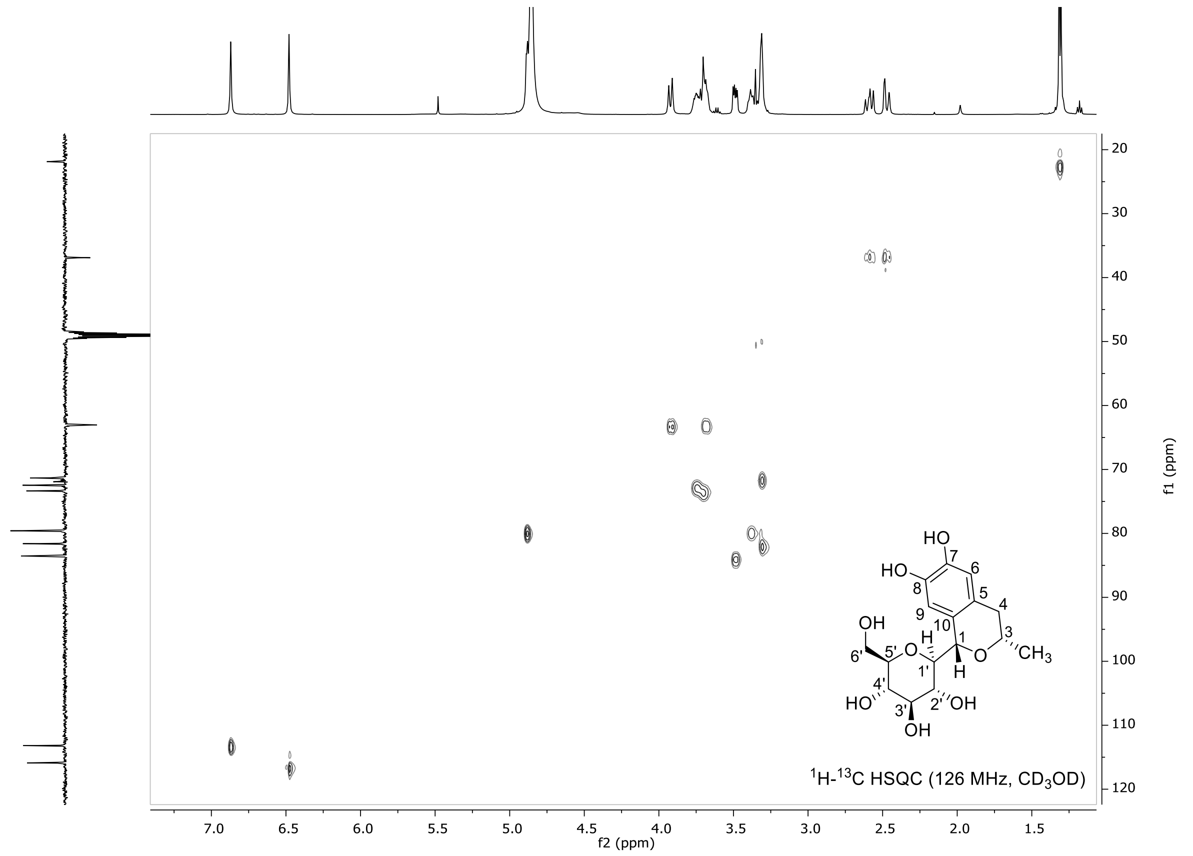
1H-13C HSQC (126 MHz, CD3OD)
f2 (ppm)
f1 (ppm)
HO
HO
OH
OH
HO
OH
OH
CH3

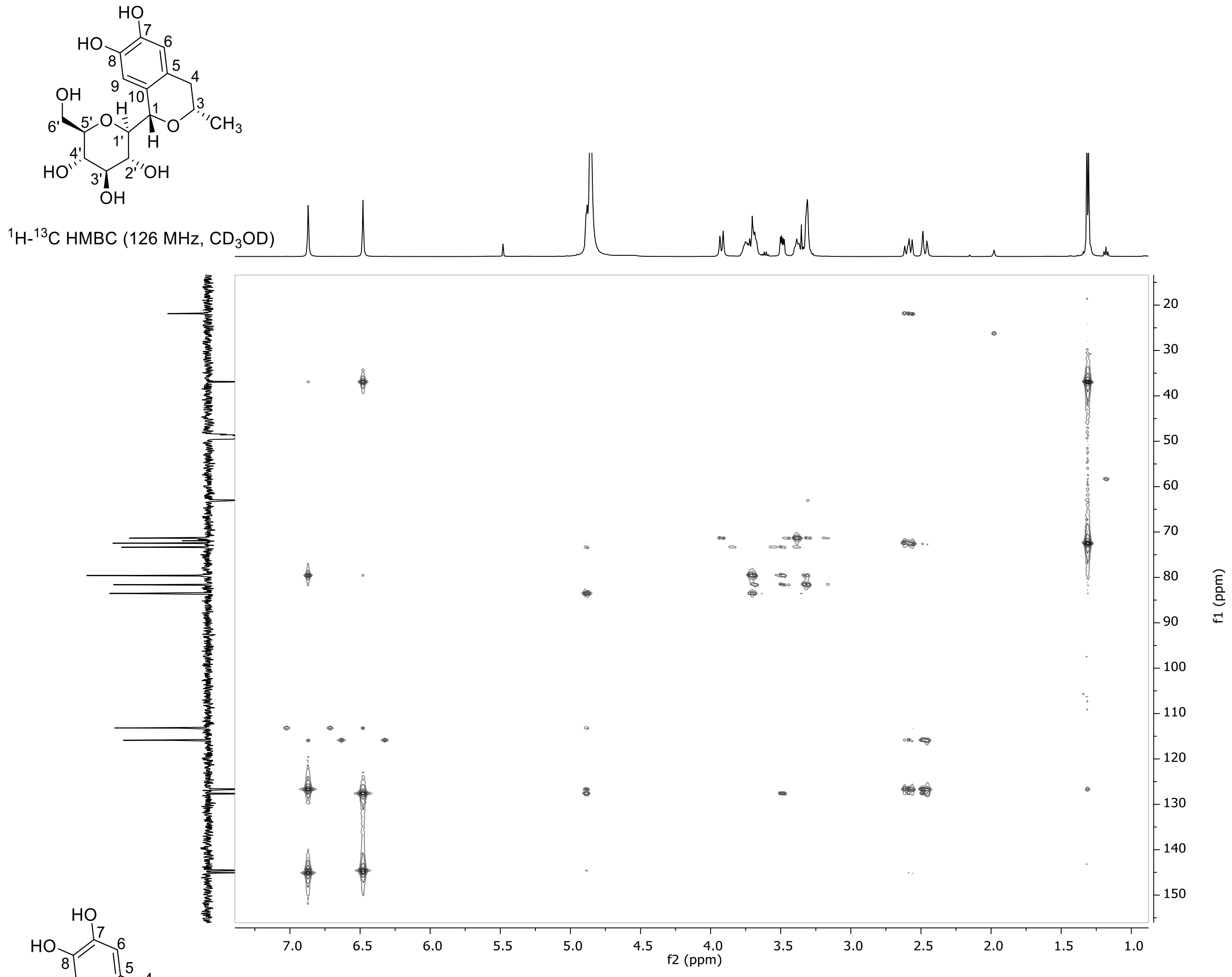
^1H-^{13}C HMBC (126 MHz, CD_3OD)
f2 (ppm)
f1 (ppm)

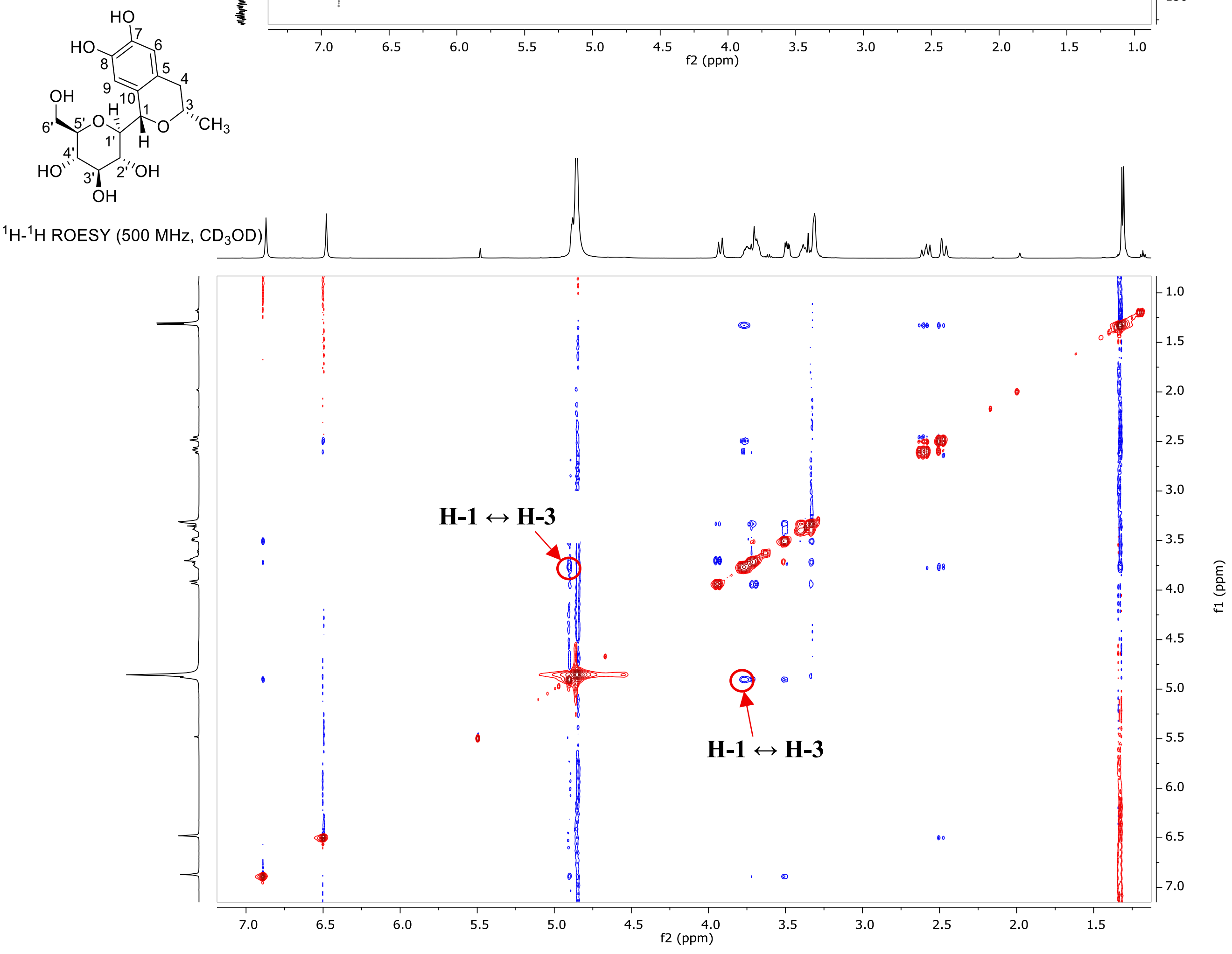
^1H-^1H ROESY (500 MHz, CD_3OD)
H-1 ↔ H-3
H-1 ↔ H-3
f2 (ppm)
f1 (ppm)

Compound 50

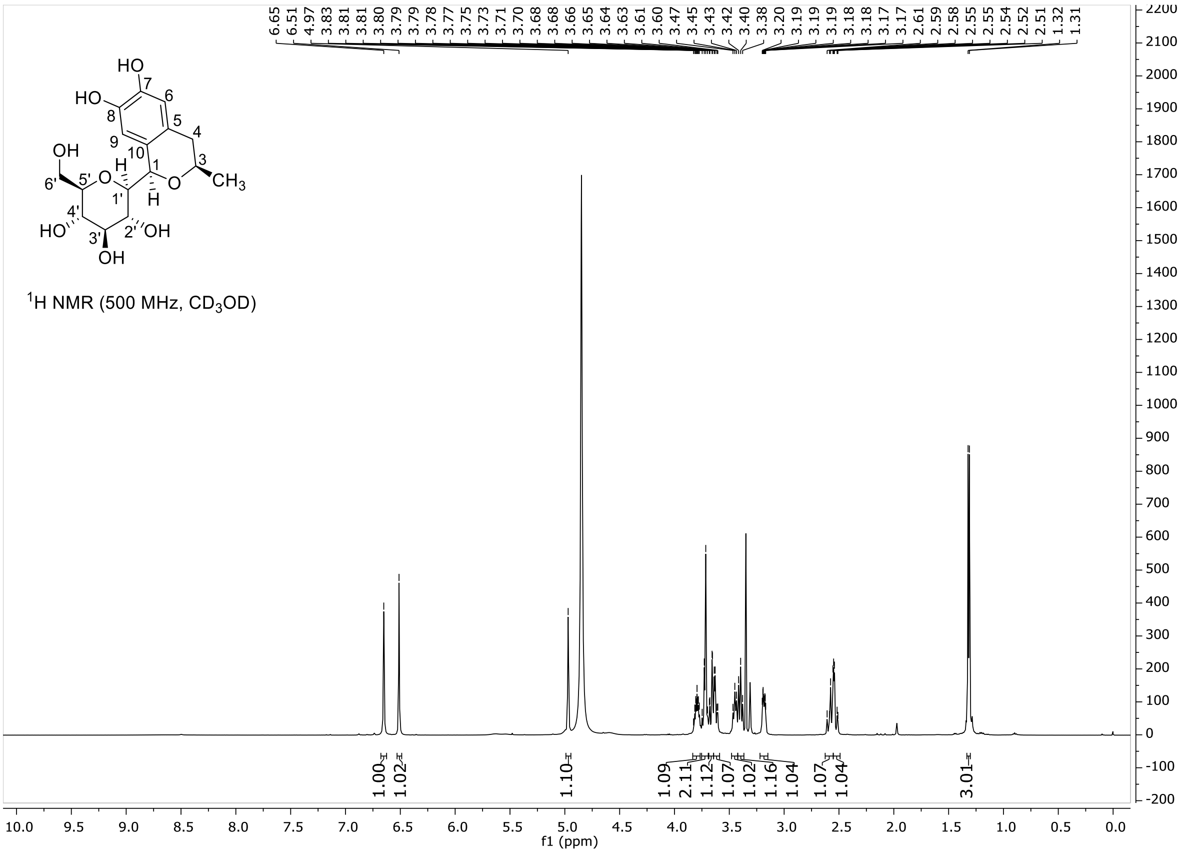

6.65 6.51 4.97 3.83 3.81 3.81 3.80 3.79 3.79 3.78 3.77 3.75 3.73 3.71 3.70 3.68 3.68 3.66 3.65 3.64 3.63 3.61 3.60 3.47 3.45 3.43 3.42 3.40 3.38 3.20 3.19 3.19 3.19 3.18 3.18 3.17 3.17 2.61 2.59 2.58 2.55 2.55 2.54 2.52 2.51 1.32 1.31
^{1}H NMR (500 MHz, CD_3OD)
1.00 1.02 1.10 1.09 2.11 1.12 1.07 1.02 1.16 1.04 1.07 1.04 3.01
f1 (ppm)

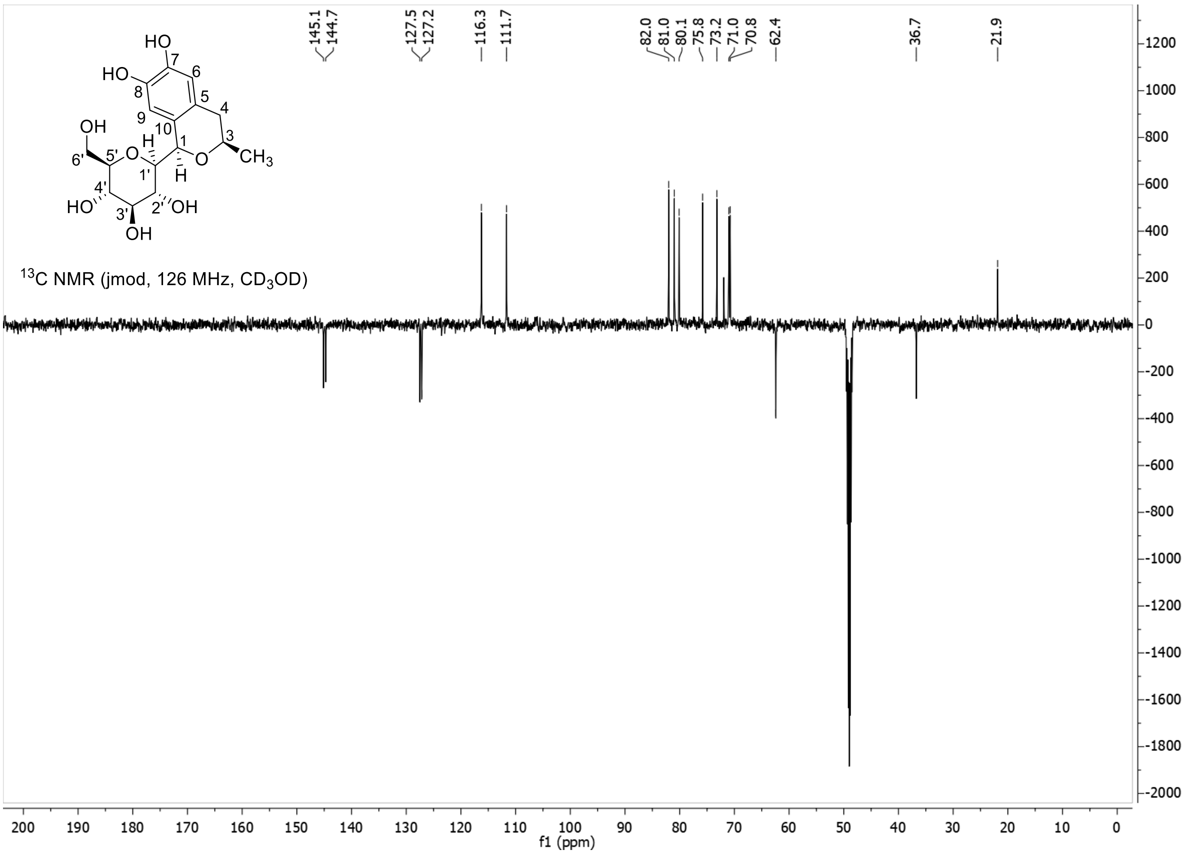

145.1 144.7 127.5 127.2 116.3 111.7 82.0 81.0 80.1 75.8 73.2 71.0 70.8 62.4 36.7 21.9
^{13}C NMR (jmod, 126 MHz, CD_3OD)
f1 (ppm)

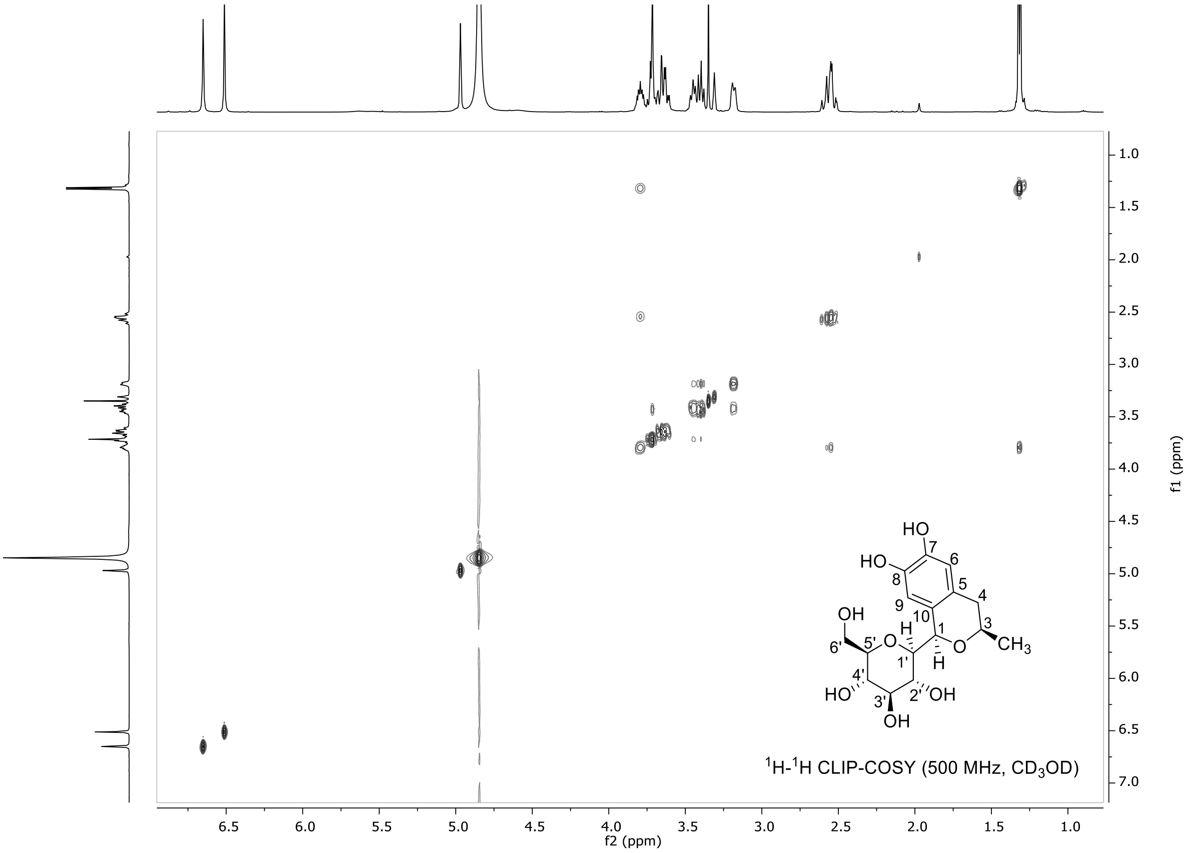
HO
7
6
HO
8
5
4
9
10
OH
H
1
3
6'
5'
O
1'
O
CH3
H
4'
HO
3'
2'
OH
OH
1H-1H CLIP-COSY (500 MHz, CD3OD)
f1 (ppm)
f2 (ppm)

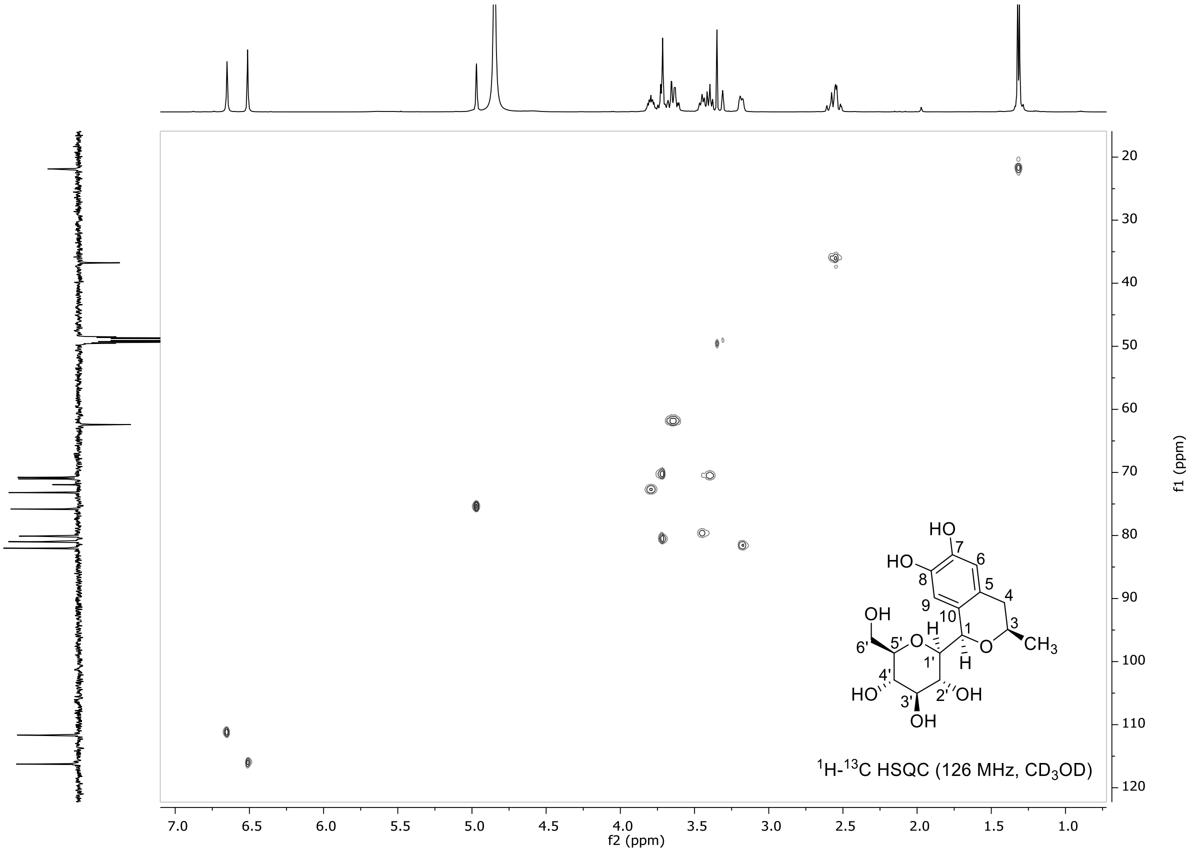
HO
7
6
HO
8
5
4
9
10
OH
H
1
3
6'
5'
O
1'
O
CH3
H
4'
HO
3'
2'
OH
OH
1H-13C HSQC (126 MHz, CD3OD)
f1 (ppm)
f2 (ppm)

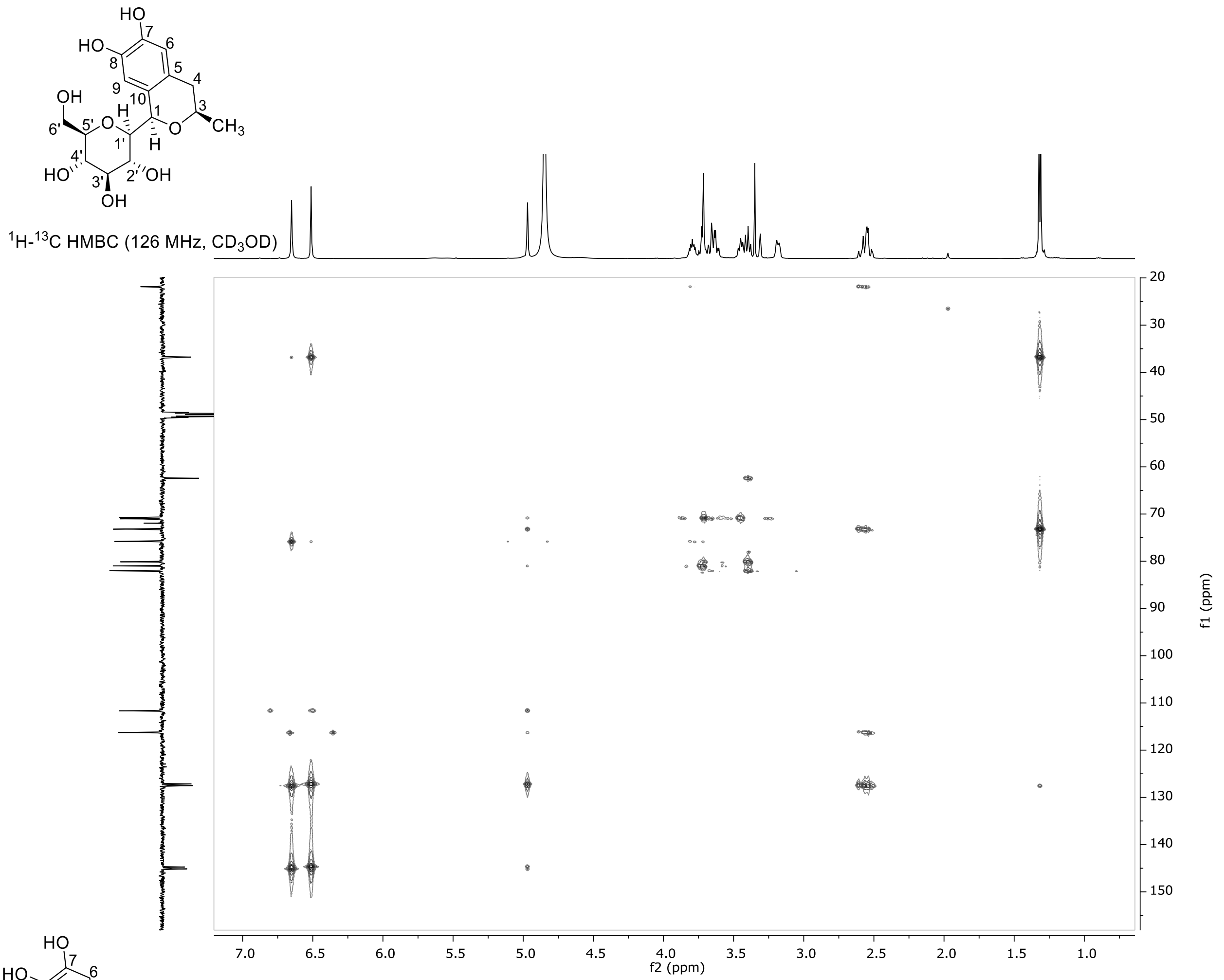
HO
HO
OH
HO
OH
OH
CH3
1H-13C HMBC (126 MHz, CD3OD)
f2 (ppm)
f1 (ppm)

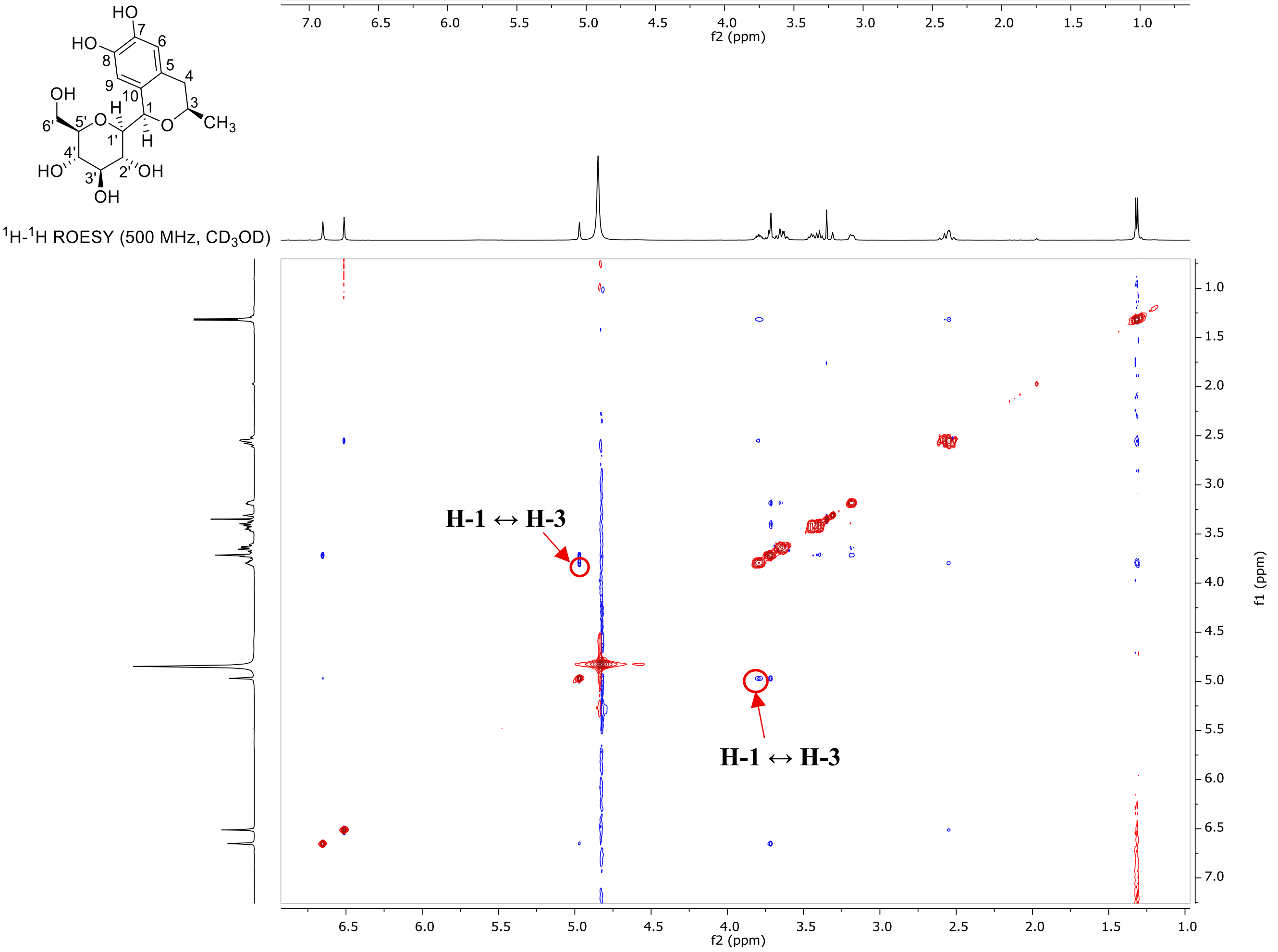
HO
HO
OH
HO
OH
OH
CH3
1H-1H ROESY (500 MHz, CD3OD)
H-1 ↔ H-3
H-1 ↔ H-3
f2 (ppm)
f1 (ppm)
